# Catalytic Enantioselective Diels–Alder Reaction of Dienes with Acyclic and α,β- and β,β-Disubstituted Enones

**DOI:** 10.1021/jacs.5c13725

**Published:** 2025-10-20

**Authors:** Jan Samsonowicz-Górski, Santanu Ghosh, Nobuya Tsuji, Markus Leutzsch, Georg Breitenbruch, Nils Nöthling, Philip Kraft, Benjamin List

**Affiliations:** † 28314 Max-Planck-Institut für Kohlenforschung, 45470 Mülheim an der Ruhr, Germany; ‡ Institute for Chemical Reaction Design and Discovery (WPI-ICReDD), 12810 Hokkaido University, Sapporo 001-0021, Japan; § 10784 Symrise AG, S&C Global Innovation Fragrances, 37603 Holzminden, Germany

## Abstract

Despite the wealth of methods to enantioselectively catalyze Diels–Alder reactions of α,β-unsaturated carbonyl dienophiles, until today, not a single example with an acyclic enone that is either β,β- or α,β-disubstituted has been reported. Herein, we disclose a general Brønsted acid-catalyzed enantioselective Diels–Alder reaction of various aliphatic acyclic disubstituted enones with hydrocarbon dienes featuring diverse steric and electronic properties. The established method has been employed to synthesize several enantiopure targets, including well-known fragrance components such as arborone and the damascones.

## Graphical abstract

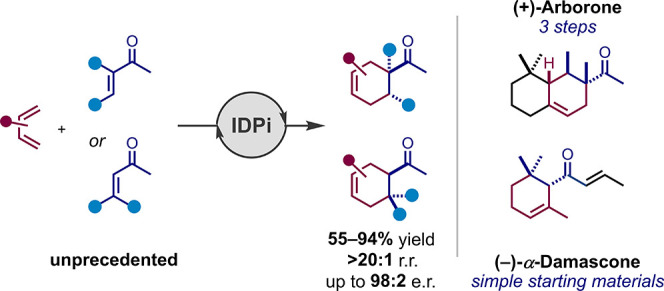

Over the past decades, numerous catalysts for enantioselective Diels–Alder reactions, including transition metal complexes, Lewis and Brønsted acids, and secondary amines have been developed.^1–11^ Particular attention has been given to the reactions of α,β-unsaturated ketone dienophiles due to their ready availability and synthetic utility in target-oriented synthesis (Figure 1A). Remarkably, not a single catalytic enantioselective ground state Diels–Alder reaction of an acyclic disubstituted enone has been described. The reason for this notable lack of examples is the drastically reduced dienophilicity of disubstituted enones caused by alkyl groups at both α- and β-positions. Unfavorable steric hindrance in these structures (i.e., the *cis*-β-methyl substituent interacting with the acyl group and hampering the reactive conformation), together with hyperconjugation, attenuates the reaction rate to the extent that product formation cannot be achieved (Figure 1B).^12,13^ Arguably, the previously used chiral Lewis acid catalysts have exhibited moderate acidity, largely due to the presence of Lewis basic and relatively bulky chiral ligands. Our design consequently featured three aspects using (a) the proton as the smallest Lewis acid, (b) a strong acid that counteracts the hyperconjugative depolarization of the dienophile, and (c) a confined acid for their proven ability to handle aliphatic unbiased substrates (Figure 1C).^14,15^ Here, we report the development of a widely applicable and highly stereoselective Diels–Alder reaction of simple dienes with aliphatic, acyclic enones, including unprecedented β,β- and α,β-disubstituted ones.

**Figure 1:**
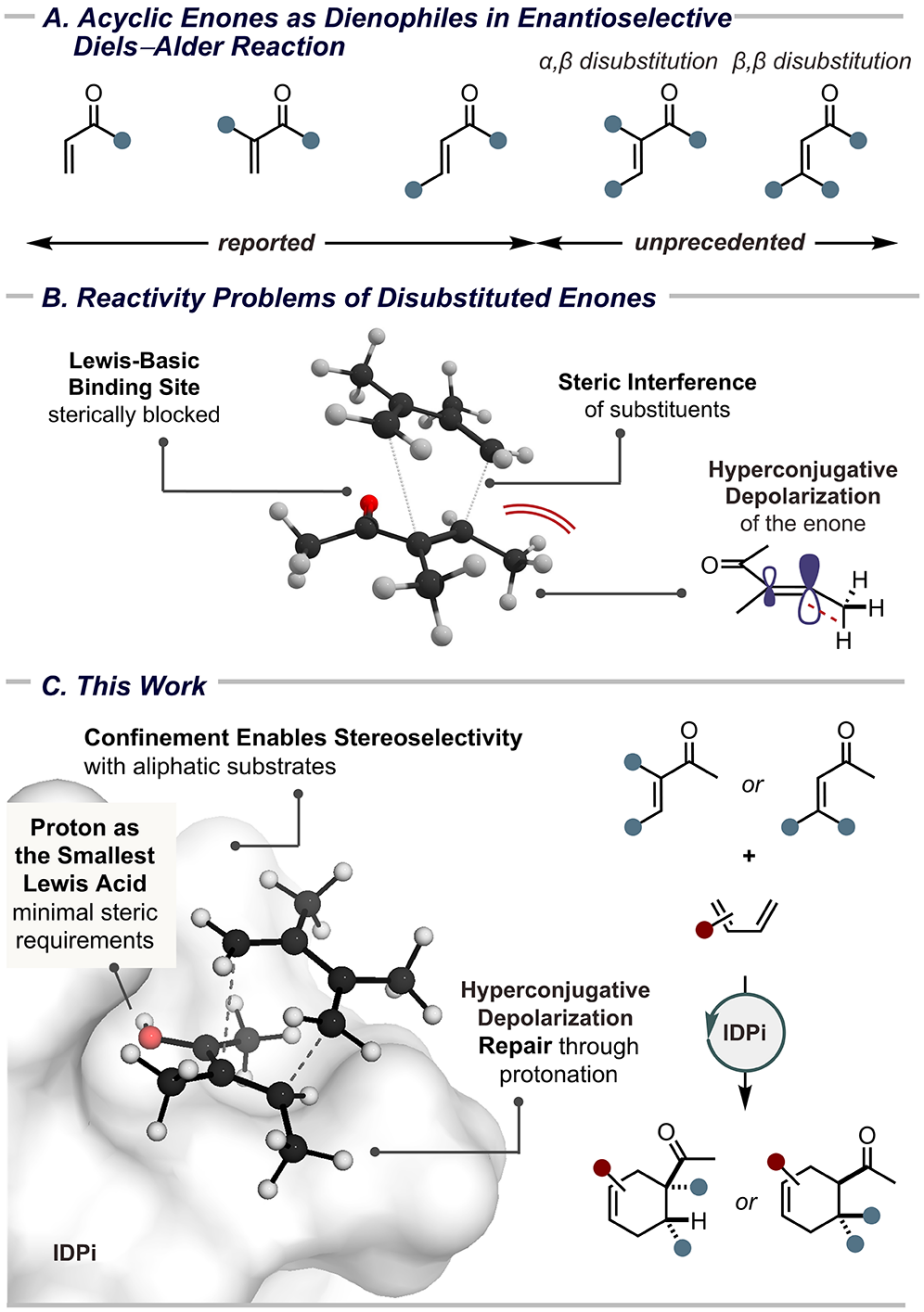
Acyclic enones in enantioselective Diels–Alder reactions.

Our work has been inspired by important terpenoid odorants, where odor profiles and thresholds are dependent on absolute configuration (Figure 2). (*S*)-α-Damascone ((−)-**1a**), for example, with a pleasant odor profile, is 65 times stronger than its enantiomer and inaccessible from simple achiral substrates.^16–19^ The situation is even more challenging for isocyclemones, which constitute a family of terpene-inspired bicyclic regio- and stereoisomers ubiquitous for 50 years in almost all segments of the commercial perfume world.^20–24^ As revealed by Corey and others, following a 15-step total synthesis, the characteristic woody-ambery smell is caused by a minor component arborone (+)-**1d** (instead of the most abundant isomer **1c**), while its enantiomer is virtually odorless.^16,25,26^ Surprisingly, no industrially applicable selective method for the synthesis of (+)-**1d** has been reported. Consequently, large amounts of inactive and poorly biodegradable side-products accumulate in the environment.^27^

**Figure 2:**
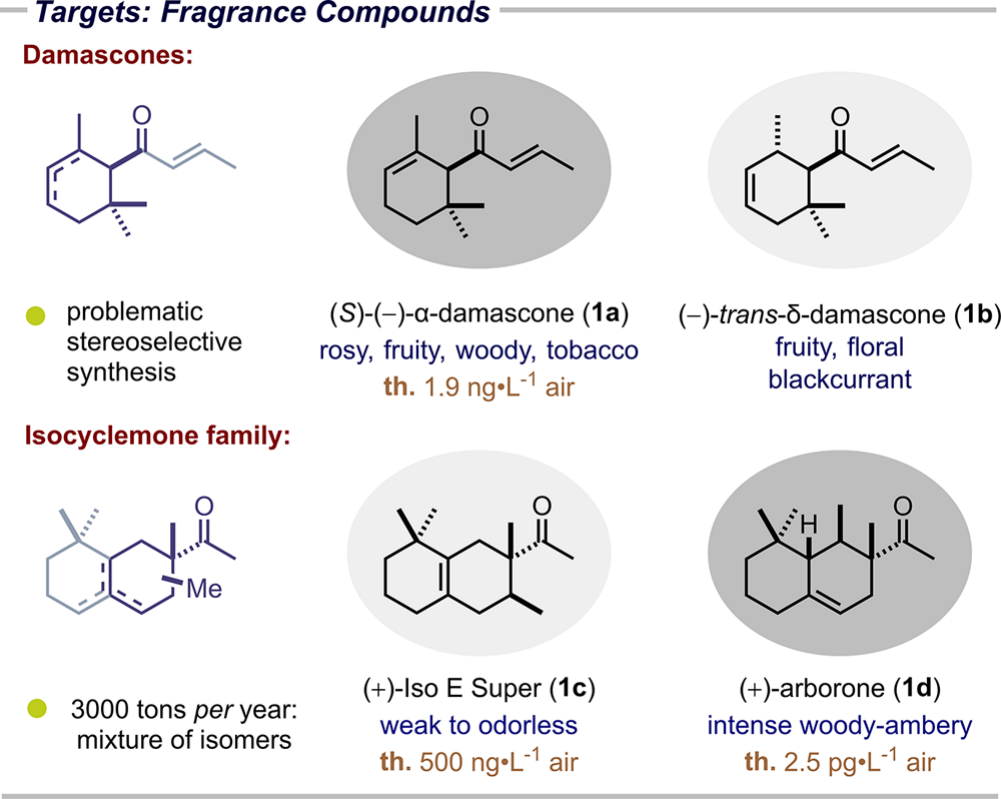
Synthetic targets of the reaction.

Technically, the isocyclemones are produced from cyclohexene *rac*-**4a** (precyclemone E), which is obtained in a Diels–Alder cycloaddition of enone **2a** with myrcene (**3a**), which was used as a model reaction (Table 1; see S.I. for details). Our investigation of an enantioselective Brønsted acid catalyzed version of this reaction revealed that IDPi **5d** featuring *para*-pentafluorosulfanyl phenyl groups at the 3,3′-positions of the binaphthyl catalyst backbone and triflyl group as the inner core gave promising results.^10,28^ Gratifyingly, highly regio- and stereoselective formation of the product was obtained at −60 °C using chloroform as a solvent after 6 days of reaction time. Anhydrous conditions were ensured by adding 5 Å molecular sieves, which prevent unselective side reactions.^29^ We were pleased to observe that, an *n*-perfluoropropyl sulfonyl group at the inner core (**5h**), afforded the most selective catalyst, delivering quantitative yield, >20:1 r.r., and 95:5 e.r. of the Diels–Alder product (Table 1).

**Table 1:**
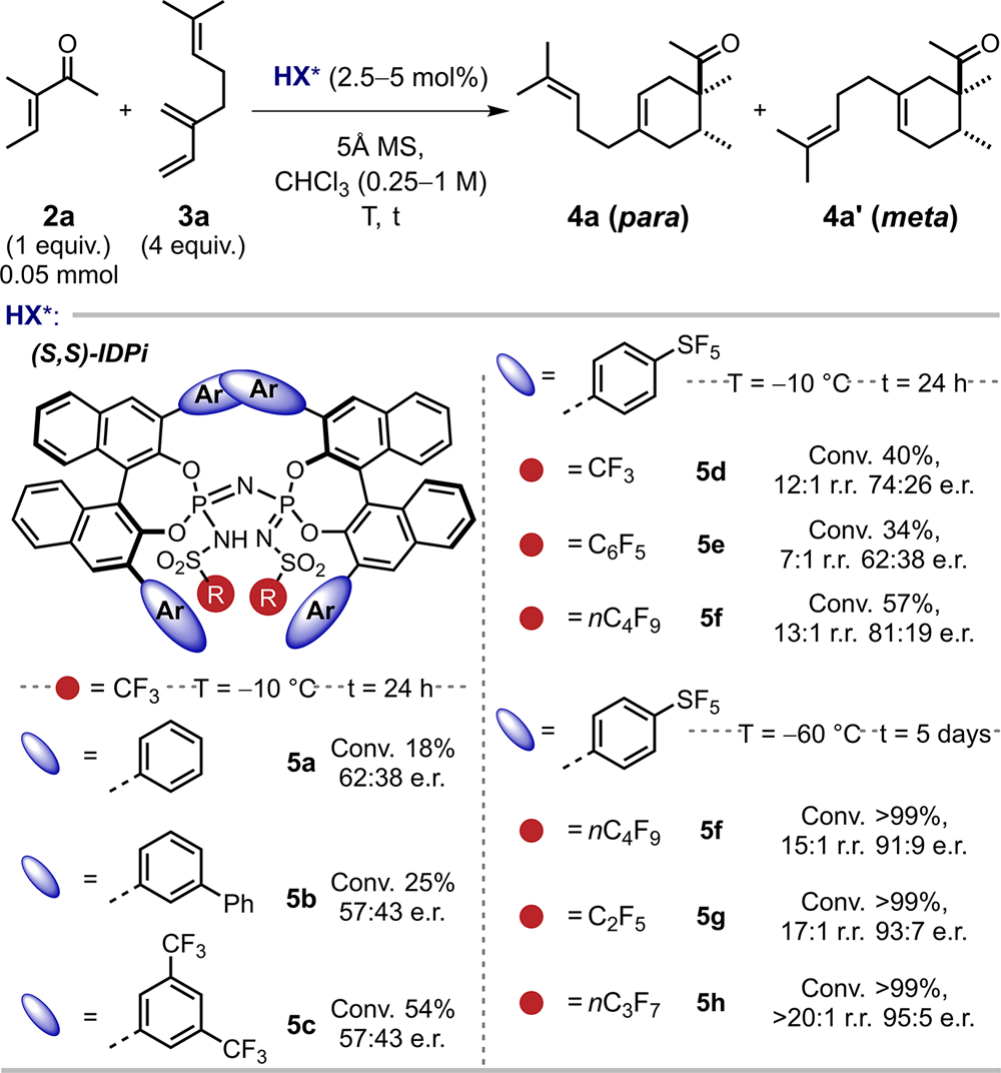
Reaction Development^a^

a Conversion (Conv.) and regioisomeric ratio (r.r.) were determined by ^1^H NMR; e.r. (enantiomeric ratio) was determined by chiral HPLC. For the IDPi-**5h** catalyst structure, see S.I. 22. Appendix 2 pg. 353.

Having established optimized reaction conditions, we investigated an array of enone dienophiles with diverse substitution patterns in the reaction with diene **3a** (Table 2). Gratifyingly, catalyst **5h** furnished high regio- and enantioselectivity irrespective of the alkyl substituents at the enone’s β-position, delivering products **4b**–**f** in up to 99% yield, 97:3 e.r., and >20:1 r.r. (Table 2). Remarkably, the method was also found suitable for the formation of C_5_–C_6_ and C_6_–C_7_ bicyclic systems (**4g**–**j**) present in *Euphorbia* diterpenoids from *exo*-acyl enones as starting materials.^30^

**Table 2:**
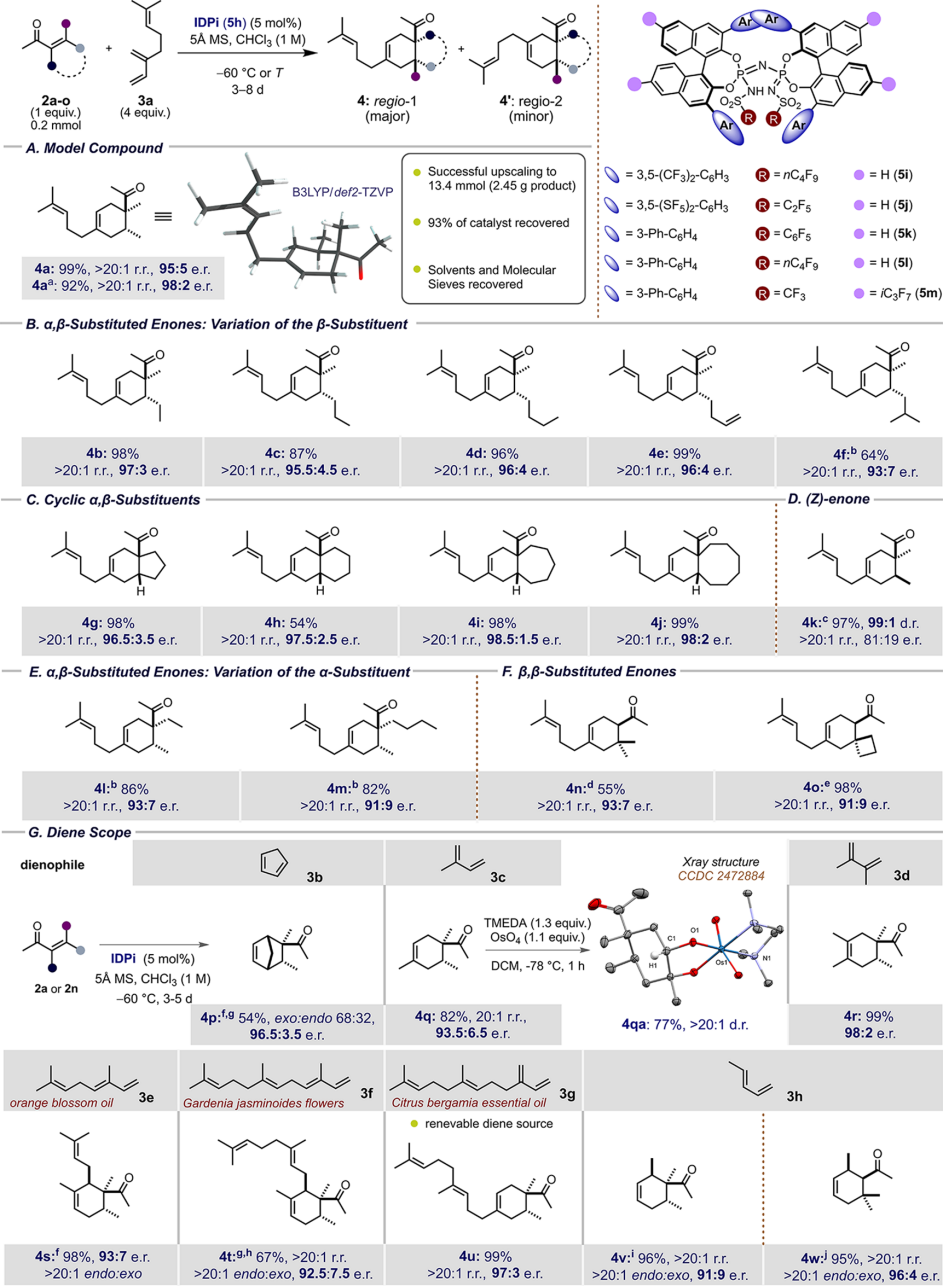
Reaction Scope

Yields of isolated products are provided. r.r. and e.r. values were determined using chromatographic methods (see S.I.). Relative configurations were assigned by NMR spectroscopy. The absolute configuration of compounds **4q** and **4r** were determined upon derivation to osmate esters (**4qa**, **4ra**) using anomalous dispersion in a single-crystal X-ray diffraction experiment,^32^ **4r** was derivatized to Mosher ester **4rb**.^38^ Absolute configuration of **4n** was established using the ECD technique. The absolute stereochemistry of **4w** was determined upon derivation and comparison of the [α]_D_^25^ value available in the literature (Scheme 1 and S.I.).

a Reaction at −90 °C with 8 equiv of diene.

b Solvent: pentane:CHCl_3_ 5:3 (v/v).

c Fed-batch reactor setup with constant addition of enone (see S.I. for details).

d Reaction at −40 °C, catalyst **5i**, isopropanol (*c* = 0.87 M).

e Reaction at −40 °C, catalyst **5j**.

f Catalyst **5k**.

g Reaction at −80 °C.

h Catalyst **5l**.

i Catalyst **5m**.

j Reaction at −40 °C, catalyst **5c** (2% mol), CyMe as solvent, HFIP (*c* = 0.57 M).

(*Z*)-Enones seem to be more challenging substrates for our reaction. For example, (*Z*)-**2a** readily isomerized to the corresponding (*E*)-isomer during the reaction, leading to poor diastereocontrol. This problem can be mitigated using a fed-batch reactor setup with a constant addition of (*Z*)-**2a** to the vessel (see S.I.). Product **4k** could be obtained with 97% yield, >99:1 d.r., and 81:19 e.r. Having optimized the diastereoselectivity of the reaction, we next turned our attention to enones with longer alkyl chains at the α-position, where the increased steric bulk hampers the reaction under standard conditions. Optimization of the solvent system enabled the synthesis of ketones **4l** and **4m** in respectable yields and enantioselectivites.

As mentioned before, although dozens of diverse and powerful approaches to stereoselective Diels–Alder reaction have been reported, not a single chiral catalyst has previously enabled handling β,β-disubstituted enones. Remarkably, we found that, upon addition of isopropanol to the reaction and using catalysts **5i** or **5j** under standard conditions, **3a** reacted with mesityl oxide (**2n**) and 1-cyclobutylidenepropan-2-one (**2o**) to form products **4n** and **4o** with high enantiomeric ratios.

Having established a significant dienophile scope, we turned our attention to dienes possessing miscellaneous steric and electronic properties. Initially, highly reactive cyclopentadiene (**3b**, *k*_rel_ = 1348)^31^ was harnessed in a stereoselective reaction; however, the *endo* to *exo* ratio remained moderate. 2,3-Dimethylbutadiene (**3d**, *k*_rel_ = 4.9) and less reactive isoprene (**3c**, *k*_rel_ = 2.2) still afforded the corresponding products with excellent yields (82–99%) and enantioselectivities (up to 98:2 e.r.) and provided the precursors of osmate esters **4qa** and **4ra** (in the S.I.), which were used in the assignment of absolute configuration.^32^ Subsequently, we investigated the transformations of naturally occurring dienes: ocimene (**3e**),^33^ (*E*,*E*)-α-farnesene (**3f**),^16,34,35^ and (*E*,*E*)-β-farnesene (**3g**),^16,36^ accessible from renewable sources.^16,37^ To our delight, terpene-derived products **4s**–**u** were obtained with excellent regio-, diastereo-, and enantioselectivities.

Subsequently, we turned our attention to differences of olfactory properties between enantiomers. It was interesting to study the enantiomeric pairs of compounds **4q** and **4r**, both sterically congested cyclohexenes with an acetyl group as an osmophore (Table 3).^39,40^ Interestingly, **4q** possessed an herbal-resinous, terpenic odor with the warm, pleasantly peppery, earthy-musty, slightly woody-green character of 4-terpineol and floral facts in the direction of lilly of the valley. In contrast, *ent*-**4q** has a pronounced minty-anisic character. In the 3-methyl homologous series, enantiomer **4r** is again more terpenic. Gratifyingly, *ent*-**4r** is more violet in character, this time in the direction of α-ionone, with the creamy-woody orris note and earthy facets accompanied by a green, fruity-herbal aspects of fig leaves and the tropical green of β-cyclocitral. While of limited interest to the perfumer, these are impressive examples for the enantioselectivity of odor perception.

**Table 3:**
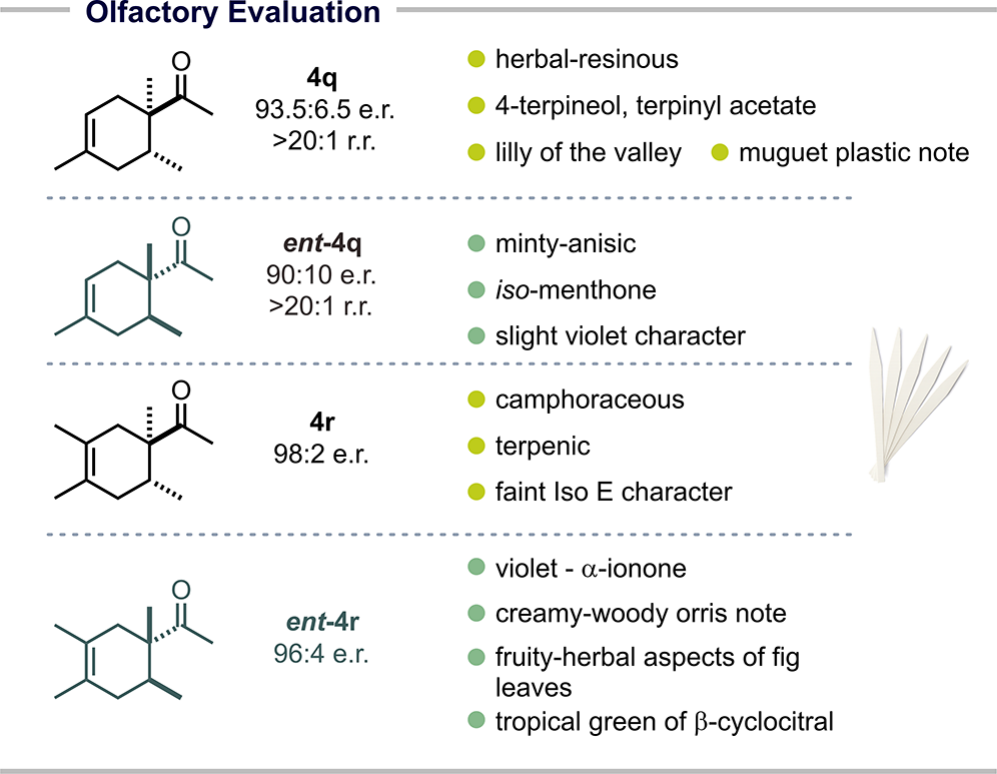
Olfactory Evaluation

Analysis of solutions in DPG (dipropylene glycol) were carried out on paper blotter over a period of 24 h, with smelling fresh and at 4 h, 8 h, and 24 h (see S.I. Appendix 4).

Lastly, of equal importance, the stereoselective cycloaddition to piperylene (**3h**, *k*_rel_= 3.3) yielded product **4v** with 91:9 e.r. upon introducing acidifying perfluoroisopropyl substituents at the 6,6′-positions of the catalyst backbone (**5m**). However, with mesityl oxide (**2n**), the formation of **4w** was not observed. We reasoned that a polar protic solvent may have a beneficial effect on the formation of **4w** (as observed for compound **4n**). Gratifyingly, by replacing chloroform with methylcyclohexane and upon addition of HFIP (1,1,1,3,3,3-hexafluoroisopropanol), the reaction furnished **4w** in 95% yield with 96:4 e.r. The synthesis was successfully conducted on a 2 mmol scale, and the catalyst was recovered (87% yield) with full catalytic activity upon reacidification.

In order to understand the reaction mechanism, we performed intramolecular competition ^12^C/^13^C NMR kinetic isotope effect studies (KIE) at natural abundance.^41^ As a model reaction, we used the synthesis of compound **4r** under various conditions: (1) the thermal reaction, (2) the AlCl_3_-catalyzed reaction, and (3) our model reaction conditions with catalyst **5h** (Figure 3). These relative KIE studies suggest that the reaction might proceed via a concerted asynchronous mechanism with the bond between C_5_ and C_6_ being formed “first”, which is expected for these kinds of systems.^42^

**Figure 3:**
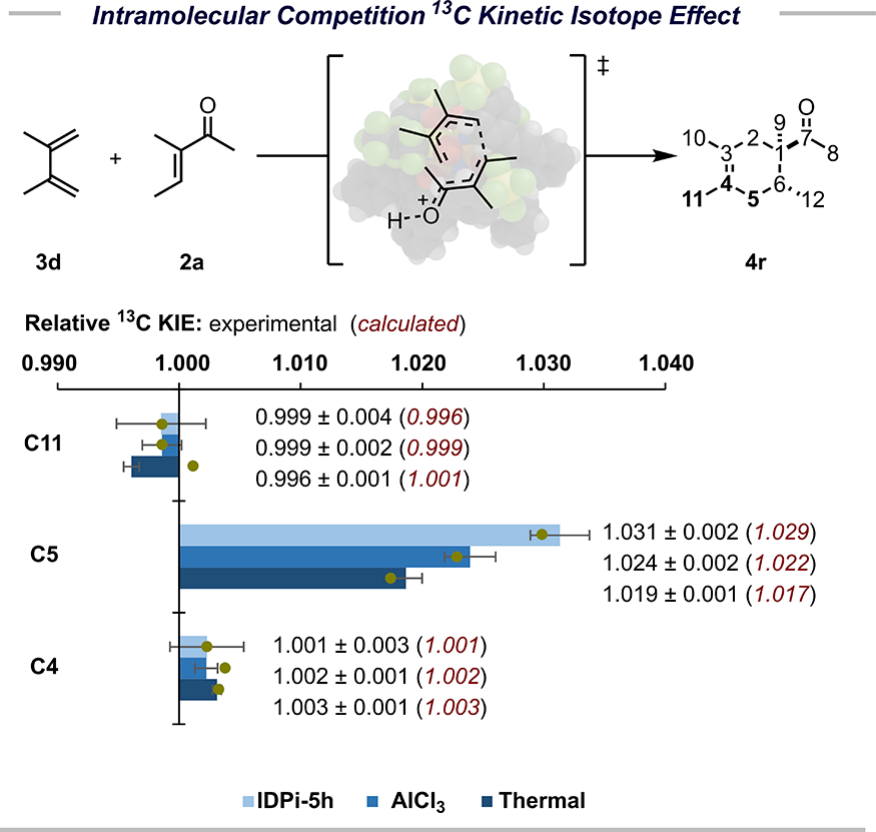
Intramolecular competition ^13^C KIE at natural abundance and calculation results using cycloaddition of **2a** and **3d** as a model.

To further analyze both the reaction mechanism and origins of regio- and enantioselectivity, we performed DFT studies of the reaction between 3-methyl-pent-3-enone (**2a**) and isoprene (**3c**) catalyzed by IDPi **5h**. This reaction proceeds via transition state **TS**_**maj**_ throughout asynchronous cycloaddition (Figure 4A). The plot of the intrinsic reaction coordinates (IRCs) against electronic energy and bond length clearly depicts the asynchronicity of the reaction: formation of the C_5_–C_6_ bond takes place at **TS**_**maj**_, while the other C_2_–C_1_ bond length remains at 2.88 Å. The IRC reaction path exhibits a detectable shoulder after **TS**_**maj**_, corresponding to the spontaneous formation of the C_2_–C_1_ bond (Figure 4B). Based on this mechanism, we optimized transition states of the reaction between **2a** and **3d** to discover that calculated KIE values are in excellent agreement with the experiment results (Figure 3).

**Figure 4:**
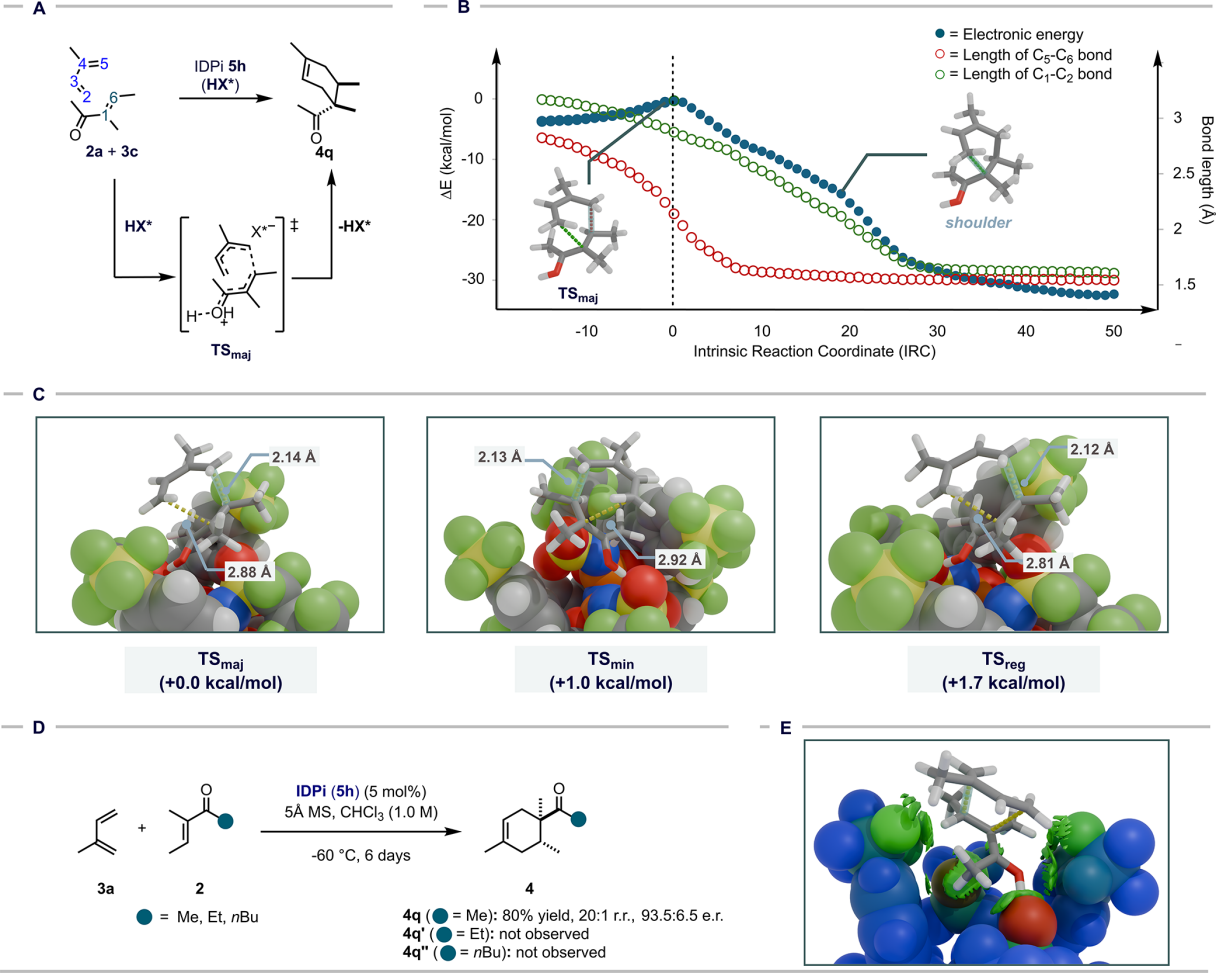
Investigation of the reaction mechanism of the IDPi-**5h**-catalyzed stereo- and regioselective Diels–Alder reaction. (A) Summary of the reaction mechanism. (B) IRC plot against the electronic energy profile and bond length of C–C bonds. The geometry optimization and electronic energies were obtained at the r^2^SCAN-3c level of theory. Only cationic substrates are depicted for clarity. (C) Comparison of transition states leading to the major isomer (**TS**_**maj**_), the minor isomer (**TS**_**min**_), and the regioisomer (**TS**_**reg**_). The free energies were obtained at the SMD­(CHCl_3_)-ωB97M-V/def2-TZVPP//r^2^SCAN-3c level of theory.^48–51^ (D) Reactivity comparison between methyl, ethyl, and butyl ketone: necessity of the methyl group for binding of enone in the catalytic pocket of IDPi **5h**. (E) IGMH plot of noncovalent interactions between reactants and the catalyst in **TS**_**maj**_.

To gain deeper insights into the origin of the enantio- and regioselectivities, we calculated the transition states leading to the minor enantiomer (**TS**_**min**_) and the regioisomer (**TS**_**reg**_) (Figure 4C). The energy difference between **TS**_**maj**_ and **TS**_**min**_ was 1.0 kcal/mol, which aligns well with the experimental outcome (93.5:6.5 e.r.; 1.1 kcal/mol). The energy difference between **TS**_**maj**_ and **TS**_**reg**_ was 1.7 kcal/mol, also in good agreement with the experimental observation (>20:1 r.r.; >1.2 kcal/mol). The origin of the enantioselectivity appears to arise from substrate packing in the microenvironment offered by the catalyst anion; **TS_maj_** is more stable than **TS_min_**, likely due to favorable noncovalent interactions, as supported by a series of decomposition analyses (see S.I.).^43,44^ In contrast, the binding modes of **TS_maj_** and **TS_reg_** are similar with the energy difference mainly arising from the difference in the substrate energies. Regardless of the isomers, all reaction paths displayed an asynchronous concerted mechanism, as indicated by the bond lengths. The methyl group of the ketone consistently anchors toward the catalytically active site, while other substituents point outward, explaining the scope of the transformation. In contrast to reactive methyl ketone **2a**, ones with longer alkyl groups drastically deteriorate the reactivity (Figure 4D). This orientation can be attributed to the multiple noncovalent interactions between polarized C–H bonds and the Lewis basic catalyst anions, visualized using the independent gradient model based on the Hirschfeld partition (IGMH) method (Figure 4E).^45–47^

Having obtained all desired Diels–Alder cycloadducts and elucidated the origins of the selectivity, we focused on the synthetic objectives. First, we used enantioenriched **4w** as a starting material in the enantioselective synthesis of damascones (Scheme 1).^16,52–54^ As expected, a rhodium-salt-catalyzed isomerization of the endocyclic double bond (to trisubstituted olefin **6w**) followed by an aldolization-crotonization sequence furnished products (−)-**1a** and (−)-*cis*-**1b** with high enantioselectivity (95:5 e.r.). Base-catalyzed epimerization of the α-tertiary stereocenter of product **4w**, followed by the above-mentioned reaction sequence, yielded *trans*-**1b** in 67% yield and 95:5 e.r. Notably, (−)-(1*S*,2*R*)-*trans*-**1b** with an e.r. of >90:10 was recently shown to be inherently biodegradable (>60% of the material is degraded within 60 days according to the OECD 301F protocol), which speaks for the use of this material in enantioenriched form.^55,56^

Furthermore, (+)-Iso E Super (**1c**) was obtained from ketone **4a** with 96% yield and 95:5 e.r. via cyclization catalyzed by NaHSO_4_. Finally, we investigated potassium bisulfate catalyzed isomerization of the endocyclic double bond.^57^ The obtained 1:1 mixture of **6a** and **4a** was submitted to the IDPi-**5c**-catalyzed cyclization to obtain (+)-arborone (**1d**) in 20% yield and 94:6 e.r in a mixture with other cyclization products **1c**–**f**, which were isolated and characterized (see S.I.).^58,59^

**Scheme 1:**
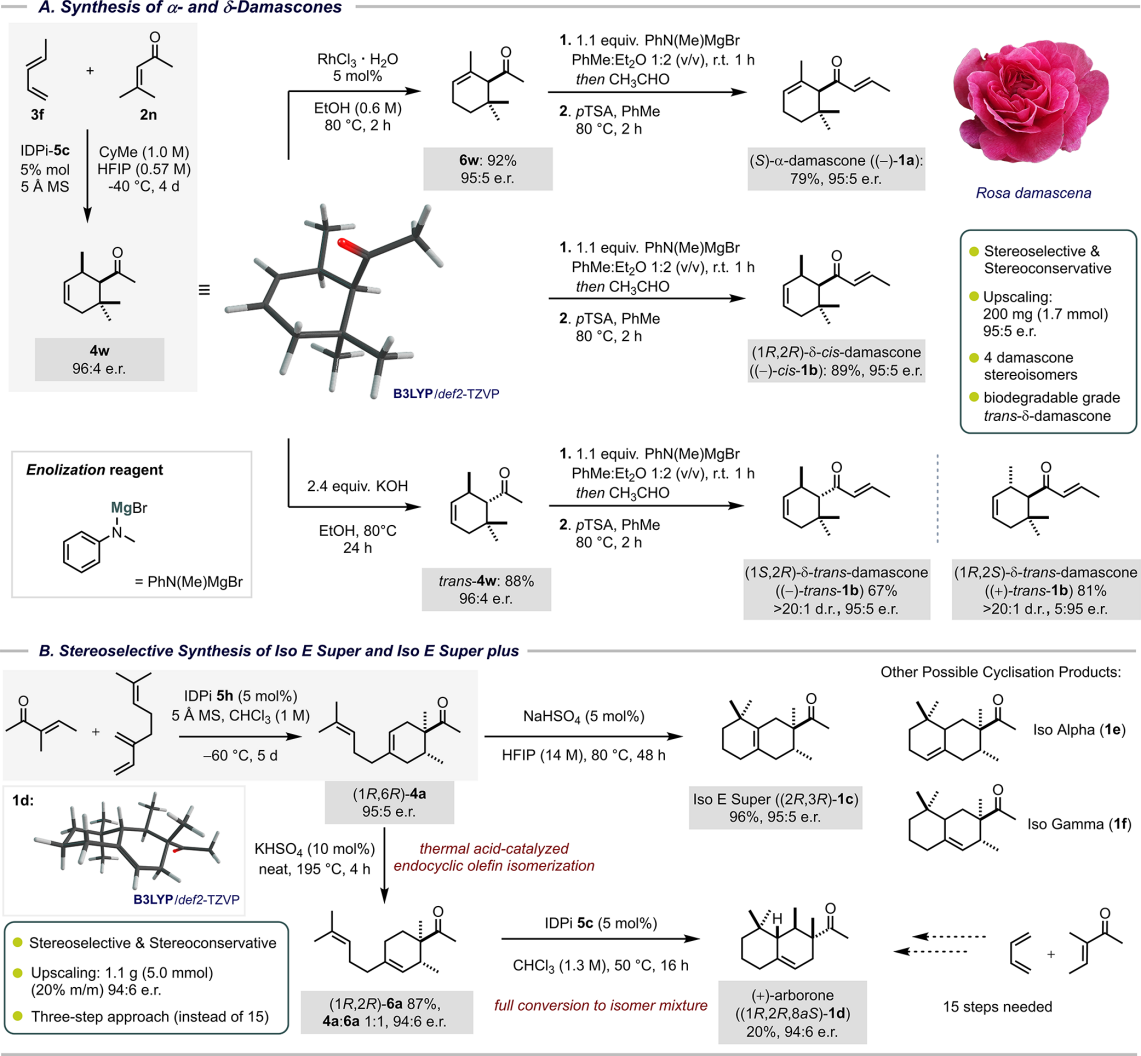
Synthetic Application of the Method

a See S.I. for details.

In summary, almost 100 years after the publication of the original reaction by Diels and Alder, the stereoselective cycloaddition of sterically demanding dienophiles and an array of dienes was successfully performed using confined chiral Brønsted acids as catalysts. Additionally, a library of terpene-inspired compounds was obtained including enantioenriched arborone, one of the most valuable fragrances; here, it was synthesized with the shortest pathway and highest yield reported so far. We also successfully synthesized various enantiopure damascone isomers including a biodegradable grade δ-damascone, which is appreciated for its odor profile, intensity, and cost-effectiveness in application.

## Supplementary Material



## Notes

Experimental details, materials, NMR spectra, HRMS data, GC and HPLC chromatograms, ECD spectra, mechanistic and computation studies details and Cartesian coordinates of calculated structures (PDF)

## Abbreviations

CPA: chiral phosphoric acid
DSI: disulfonimide
IDP: imidodiphosphate
iIDP: imidodiphosphates
IDPi: imidodiphosphorimidates
e.r.: enantiomer ratio
r.r.: regioisomer ratio
HPLC: high performance liquid chromatography
NMR: nuclear magnetic resonance
S.I.: Supporting Information
*p*TSA: *para*-Toluenesulfonic acid
HFIP: 1,1,1,3,3,3-hexafluoroisopropanol
GC: gas chromatography
DPG: dipropylene glycol
