# Supplementary material for: Catalytic Enantioselective Diels–Alder Reaction of Dienes with Acyclic and α,β- and β,β-Disubstituted Enones
Source: J Am Chem Soc. 2025 Oct 20;147(43):39011–9. doi: 10.1021/jacs.5c13725 (PMC12576814; doi:10.1021/jacs.5c13725)
Supplement: Supplementary file 1 [file ja5c13725_si_002.pdf]

Supporting Information

Catalytic Asymmetric Diels–Alder Reaction of Dienes with Acyclic, α,β- and β,β-Disubstituted Enones

Jan Samsonowicz-Górski, Santanu Ghosh, Nobuya Tsuji, Markus Leutzsch, Georg Breitenbruch, Nils Nöthling, Philip Kraft and Benjamin List*

1. Contents

2. Materials and Instrumentation 5
3. Synthesis of Enones 7
 3.1. General procedure for the synthesis of (*Z*)-2a,2b,2q' and 2q'': 7
 3.2. Procedure for the synthesis of enones 2c-f 10
 3.3. Procedure for the synthesis of enones 2g-j 12
 3.4. Procedure for the synthesis of enones 2l-m 14
 3.5. Procedure for the synthesis of enone 2o: 16
4. Synthesis of (*E,E*)-α-Farnesene (3f)[11,12] 16
5. General procedure for the racemic Diels-Alder reaction 18
 5.1. Alumina Chloride Catalysed Diels-Alder Reaction (4a-s,v,w) 18
 5.2. IDPi catalyzed synthesis of racemates: 4t and 4u 19
6. General procedure for the catalytic enantioselective Diels-Alder reaction 20
7. Syntheses of IsoESuper and IsoESuper plus(Arborone) 40
 7.1. Isomerisation of the Endocyclic Double Bond of PrecyclemoneE (4a) 40
 7.2. Cyclisation of Precyclemone E to IsoESuper 41
 7.3. Synthesis of the Enantioenriched IsoESuper plus (Arborone) 43
  7.3.1. Achiral Lewis acid catalysis: 43
  7.3.2. IDPi-catalyzed variant: 43

7.3.3. Purification procedure: ... 43
7.4. Preparative HPLC Methods and achiral chromatographic data. ... 47
7.4.1. Method for isolation of 6a ... 47
7.4.2. Method for separation of Isocyclemone isomers 1c-f ... 48
7.4.3. Chromatographic data for separated isomers (achiral separations) ... 51
8. Synthesis of Damascones ... 56
8.1. Synthesis of α-Damascone Precursors ... 56
8.1.1. The isomerization of the Endocyclic Double Bond[16] ... 56
8.1.2. Epimerisation of 4w to *trans*-4w[17] ... 56
8.2. Synthesis of Damascones via Aldolization-crotonization Approach[18] ... 57
9. Synthesis of IDPi catalysts ... 61
9.1. Sulfonamide Synthesis ... 61
9.2. Phosphazine Synthesis ... 64
9.3. General Procedure for BINOL synthesis 10a-f ... 66
9.4. General procedure for the Synthesis of an IDPi Catalysts ... 68
10. Upscaling Experiments ... 71
10.1. The Upscaling of Synthesis of 4a ... 71
10.2. The Upscaling of Synthesis of 4w ... 71
11. Catalyst recovery Experiments ... 73
11.1. The Recovery of IDPi-5h (for synthesis of 4a) ... 73
11.2. The Recovery of IDPi-5c (for synthesis of 4w) ... 74
12. Absolute Configuration determination by CD ... 75
12.1. Calculation of CD Spectra using DFT Methods ... 75
12.2. CD spectrum calculation ... 75
12.3. CD experiments for determination of the absolute configuration of Diels-Alder products 4n: ... 76
13. Absolute Configuration determination by Mosher ester analysis[33] ... 78
13.1. Synthesis of Mosher ester of 4r ... 78
13.2. Analysis of Mosher esters ... 85

14. Absolute Configuration determination by single crystal X-ray diffraction (SC-XRD) 91
14.1. Syntheses of osmate esters for structural analyses 91
14.2. Structural analyses of the single crystals of osmate esters 92
14.2.1. Analysis of 4qa 92
14.2.2. Analysis of 4ra 99
15. Intramolecular Competition Kinetic Isotope Effect (KIE) Experiments 106
15.1. Introduction to Experimental Mechanistic Investigation of Diels-Alder Cycloaddition 106
15.2. NMR assignment data of compound 4r in CD_2Cl_2 106
15.3. NMR data acquisition and processing methods 107
15.4. Sample Preparation 108
15.4.1. The Synthetic procedures: 108
15.4.2. The preparation of NMR samples: 109
15.5. NMR Integrals and KIE Results 110
15.5.1. Overview of obtained KIE data 110
15.5.2. Sample 1 – Thermal Reaction (4r-1) 112
15.5.3. Sample 2 – $AlCl_3$-catalysed cycloaddition (4r-2) 114
15.5.4. Sample 3 – IDPi-5c catalyzed cycloaddition (4r-3) 116
15.5.5. Sample 4 – IDPi-5h catalyzed cycloaddition (4r-4) 118
16. Computational studies 120
16.1. Method 120
16.2. Results and Discussion 120
Cartesian coordinates of the optimized structures 129
17. References: 203
18. NMR spectra 206
18.1. Enones: 206
18.2. (*E*,*E*)-α-farnesene 220
18.3. Diels-Alder Cycloaddition Products (4a-w) 221

18.4. Derivation products 246
18.5. Synthetic Targets 249
18.5.1. Sulfonamides 264
18.5.2. Phosphazine 8e 266
18.5.3. (*S*)-BINOL 267
18.6. IDPi Catalysts 269
18.7. Mosher esters 276
18.8. Osmate esters 287
19. HPLC and GC traces 289
20. Data for structural analyses of the single crystals of osmate esters 346
20.1. X-ray Crystal Structure Analysis of 4qa: 346
20.2. X-ray Crystal Structure Analysis of 4ra: 348
21. Appendix 1: Screening Table 350
22. Appendix 2: Space-filling model of the IDPi-5h 352
23. Appendix 3: Setup of the Preparative HPLC 353
24. Appendix 4: Olfactory analysis 354

2. Materials and Instrumentation

Unless otherwise stated, oven-dried (80 °C) or flame-dried glassware was used to conduct the experiments. All reactions were performed in anhydrous solvents under an argon atmosphere, applying standard Schlenk techniques. Dry argon (purity >99.5%) was purchased from Air Liquide.

Thin-layer chromatography (TLC) on silica gel pre-coated glass plates (SIL G-25 UV254, 0.25 mm, Macherey-Nagel) or plastic sheets (0.2 mm, Macherey-Nagel) was used to monitor the progress of reactions. Visualization was done by UV light at 254 nm and/or by staining with p-anisaldehyde and/or permanganate. Preparative thin-layer chromatography was performed using SIL G-25 UV254 with a 0.25 mm SiO_2 layer on glass plates. Column chromatography was performed using Merck silica gel (60 Å, 230−400 mesh, particle size 0.040–0.063 mm). Technical grade solvents were used for elution, which was accelerated using compressed air. All isolated yields are reported unless otherwise specified.

Chemicals were purchased from various commercial sources (ABCR, Acros Organics, Alfa Aesar, Fluorochem, Sigma-Aldrich, TCI, Carbolution, Apollo Chemicals) as reagent grade and used without further purification unless otherwise stated. Triethylamine (Et_3N) was distilled over $LiAlH_4$ and stored under argon prior to use. Previously reported compounds were synthesized from commercially available starting materials following literature procedures.

Solvents used as reaction media ($CHCl_3$, CH_2Cl_2, Et_2O, THF, toluene, MeCy, acetonitrile, dioxane, pentane, CyMe) were distilled using appropriate drying agents in the technical department of the Max-Planck-Institut für Kohlenforschung, stored under an argon atmosphere, and used in reactions. Spectroscopy-grade acetonitrile was purchased from Romil Ltd.

Nuclear Magnetic Resonance (NMR) spectra of ^{1}H, ^{13}C, ^{19}F, and ^{31}P nuclei were recorded on Bruker AVNeo-600 or AVNeo-500 spectrometers in $CDCl_3$, CD_3CN, methanol-d_4, or CD_2Cl_2. Resonance multiplicity is described as s (singlet), d (doublet), t (triplet), q (quartet), p (pentet), hept (heptet), m (multiplet), and br (broad). Chemical shifts (δ) are reported in ppm, and coupling constants (*J*) in Hz. The data were processed using Bruker TOPSPIN or MestReNova software suites. All spectra were recorded at 298 K unless otherwise noted.

The residual deuterated solvent signal relative to tetramethylsilane (TMS) was used as the internal reference in ^{1}H NMR spectra (δ, ppm: $CDCl_3$ 7.26; CD_2Cl_2 5.32; CD_3CN 1.96; methanol-d_4 3.34). ^{13}C NMR chemical shifts (δ, ppm) are also reported relative to TMS using solvent resonances as internal standards ($CDCl_3$ 77.16; CD_2Cl_2 53.84; CD_3CN 118.26; methanol-d_4 49.00). ^{19}F and ^{31}P NMR spectra are reported relative to CCl_3F (δ = 0 ppm) and H_3PO_4 (δ = 0 ppm), respectively.

High-resolution mass spectrometry (HRMS) was performed on a Finnigan MAT 95 (EI) or a Bruker APEX III FTMS (7 T magnet, ESI). Electron impact (EI) mass spectrometry was conducted using a Finnigan MAT 8200 (70 eV) or MAT 8400 (70 eV) spectrometer. The ionization method and mode of detection are indicated in the individual entries.

Enantiomeric ratios (e.r.) were determined by chiral Gas Chromatography (GC) or High-Performance Liquid Chromatography (HPLC). GC analyses, conducted in the GC department of the Max-Planck-Institut für Kohlenforschung, used HP 6890 and 5890 series instruments (split-mode capillary injection system, flame ionization detector (FID), hydrogen as the carrier gas). Conditions are provided in the individual experiments in the form: injection temperature [°C], time gradient in °C/min, 'iso' (isothermal separation), and flame temperature.

HPLC analyses were performed on Shimadzu LC-20AB liquid chromatographs (reversed phase, SIL-20ACHT autosampler, CTO-20AC column oven, SPD-M20A diode array detector) using Daicel columns with chiral stationary phases. The column type and solvent mixture (HPLC-grade) are indicated for each experiment.

Specific rotations $[\alpha]D^{25}$ were recorded using an Autopol IV polarimeter (Rudolph Research Analytical) at 25 °C with a sodium lamp (D line, λ = 589 nm). Measurements were carried out in an acid-resistant 1 mL cell (50 mm length), and concentrations (c, g/100 mL) are reported in $CHCl_3$.

Electronic Circular Dichroism (ECD) spectra were recorded on a J-1100 CD spectrometer (JASCO) using a 2.0 mm Light Path QS High Precision Cell quartz cuvette (Hellma Analytics). Measurements were performed at 20 °C using a thermostated sample holder. Scans were performed from 600 to 180 nm at a scan speed of 100 nm/min with a data pitch of 0.5 nm. The slit width was 1.0 nm. The high-tension voltage did not exceed 700 V

during measurements within the analyzed wavelength range. The solvent spectrum was subtracted from the final spectra. The analysis region used was 205–265 nm. Spectra were recorded in duplicate.

Abbreviations

e.r. = enantiomer ratio, d.r. = diastereomer ratio, r.r. = regioisomer ratio, TLC = thin layer chromatography, THF = tetrahydrofuran, MTBE = methyl tert-butyl ether, CyMe = methylcyclohexane, IDPi = imidodiphosphorimidate,

3. Synthesis of Enones

3.1. General procedure for the synthesis of (*Z*)-2a,2b,2q' and 2q'':

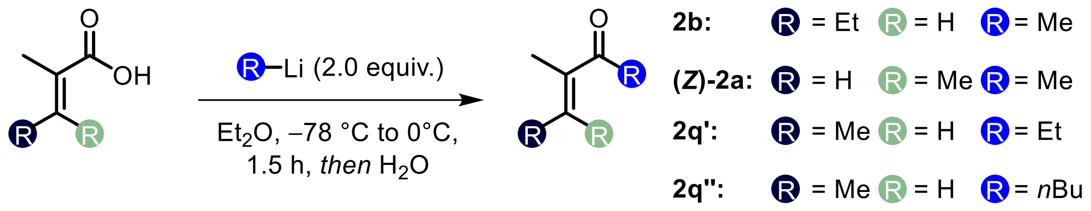

Enones **(*Z*)-2a**, **2b**, **2q′**, and **2q″** were prepared according to a modified literature procedure.[1] A flame-dried two-neck round-bottom flask equipped with a magnetic stirring bar was charged with a solution of the carboxylic acid (29.9 mmol, 1.0 equiv.) in anhydrous Et_2O (35 mL). The mixture was cooled in a dry ice–acetone bath. A solution of methyllithium in Et_2O (1.6 M, 2.0 equiv.) was then added dropwise over 10 minutes at −78 °C under a positive pressure of argon. After 30 minutes, the cooling bath was removed, and the resulting white suspension was allowed to warm to 0 °C over 1 hour. Upon reaction completion, distilled water was added, and the aqueous phase was extracted with Et_2O (3 × 20 mL). The combined organic layers were washed with brine, dried over anhydrous Na_2SO_4, and concentrated under reduced pressure (keeping the water bath temperature below 20 °C due to the volatility of the enone). The crude residue was purified by flash column chromatography on silica gel using 2–4% Et_2O in pentane as the eluent.

2a and **2n** were purchased from commercial source and distilled prior to use. All pure enones were stored under atmosphere of argon in flame-dried glassware.

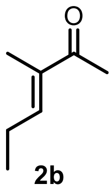

2b

(*E*)-3-methylhex-3-en-2-one (**2b**)

The enone **2b** was prepared according to the general procedure, the desired product was obtained as colorless liquid (1.69 g, 29%). **^{1}H NMR** (501 MHz, $CDCl_3$): δ 6.64–6.57 (m, 1H), 2.30 (s, 3H), 2.24 (qd, *J* = 7.5, 1.0 Hz, 2H), 1.76 (q, *J* = 1.1 Hz, 3H), 1.08 (t, *J* = 7.6 Hz, 3H); **^{13}C NMR** (126 MHz, $CDCl_3$): δ 200.1, 145.3, 137.3, 25.5, 22.5, 13.2, 11.1; HRMS (GC-EI) (m/z) calculated for $C_7H_{12}O$ $[M]^+$: 112.088265, found: 112.088450.

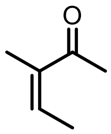

(*Z*)-2a

(*Z*)-3-methylpent-3-en-2-one (**(*Z*)-2a**)

The enone **2k** was prepared according to the general procedure, the desired product was obtained as colorless liquid (2.29 g, 85%). **^{1}H NMR** (501 MHz, $CDCl_3$): 5.86–5.81 (m, 1H), 2.23 (d, *J* = 1.0 Hz, 3H), 1.89 (p, *J* = 1.5 Hz, 3H), 1.86 (m, 3H); **^{13}C NMR** (126 MHz, $CDCl_3$): δ 203.0, 136.4, 133.6, 30.2, 21.0, 15.8. Spectral characteristics consistent with those previously reported.[2]

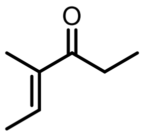

2q'

(*E*)-4-methylhex-4-en-3-one (2q')

The enone **2q'** was prepared according to the general procedure (using ethyllithium solution in benzene). Because of the enone volatility, the residual benzene was distilled out using Vigreux column before purification by silica gel chromatography. Further, product was purified from solvent residue by Kugelrohr distillation: p = 66 mbar, T = 80 °C.[3] The desired product was obtained as colorless liquid (0.26 g, 22%). **^{1}H NMR** (501 MHz, $CDCl_3$): δ 6.72–6.70 (m, 1H), 2.63 (q, *J* = 7.3 Hz, 2H), 1.82 (dt, *J* = 6.9, 0.9 Hz, 3H), 1.75 (t, *J* = 1.5 Hz, 3H), 1.06 (t, *J* = 7.3 Hz, 3H).**^{13}C NMR** (126 MHz, $CDCl_3$): δ 202.4, 138.1, 136.7, 30.3, 14.8, 11.1, 8.9. Spectral characteristics consistent with those previously reported.[4]

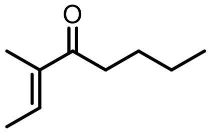

2q''

(*E*)-3-methyloct-2-en-4-one (**2q''**)

The enone **2q''** was prepared according to the general procedure (from butyllithium), the desired product was obtained as colorless liquid (2.31 g, 69%). **^{1}H NMR** (501 MHz, $CDCl_3$): δ 6.71 (qq, *J* = 6.9, 1.5 Hz, 1H), 2.64 – 2.58 (m, 2H), 1.83 (dq, *J* = 6.9, 1.1 Hz, 3H), 1.75 (p, *J* = 1.1 Hz, 3H), 1.63 – 1.48 (m, 2H), 1.36 – 1.23 (m, 2H), 0.89 (t, *J* = 7.4 Hz, 3H); **^{13}C NMR** (126 MHz, $CDCl_3$): δ 202.2, 138.4, 136.9, 37.0, 27.3, 22.7, 14.8, 14.0, 11.1. **HRMS**: (GC-EI) (m/z) calculated for $C_9H_{16}O$ $[M+Na]^+$: 163.109334, found: 163.109490.

3.2. Procedure for the synthesis of enones 2c-f

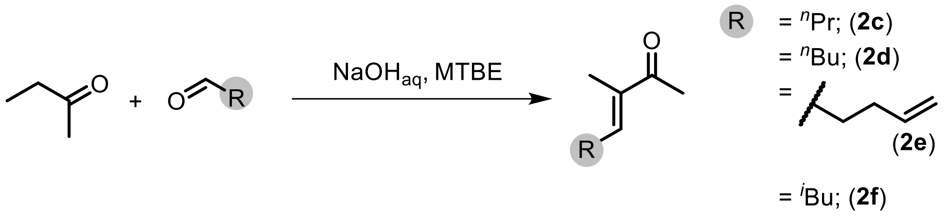

A 250 mL round-bottom flask (equipped with a Teflon-coated magnetic stirring bar) was charged with an aqueous NaOH solution (1.4 M, 100 mL for 28.1 mmol). To this solution, 2-butanone (4.0 equiv.) was added at 0 °C, and the mixture was stirred vigorously. Subsequently, a premixed solution of the corresponding aldehyde in MTBE (6.0 M) was added dropwise over 30 minutes. The reaction mixture was then stirred at room temperature for 24 hours. After completion, the reaction mixture was diluted with Et_2O and treated with 10% aqueous HCl. The organic layer was separated, dried over anhydrous Na_2SO_4, and concentrated under reduced pressure. The crude product was purified by column chromatography on silica gel using 2–6% Et_2O in pentane as the eluent, affording the product as a colorless oil.

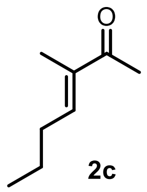

2c

(*E*)-3-methylhept-3-en-2-one (**2c**)

The title enone **2c** was prepared using the general procedure, in 33.3 mmol scale, the desired product was obtained as a colorless oil (0.45 g, 11%). **^{1}H NMR** (501 MHz, $CDCl_3$): δ 6.64–6.61 (m, 1H), 2.30 (s, 3H), 2.22 (q, *J* = 7.4 Hz, 2H), 1.76 (br. s, 3H), 1.50 (h, *J* = 7.4 Hz, 2H), 0.96 (t, *J* = 7.4 Hz, 3H); **^{13}C NMR** (126 MHz, $CDCl_3$): δ 200.1, 143.8, 137.9, 31.3, 25.6, 22.1, 14.1, 11.3; **HRMS**: (GC-EI) (m/z) calculated for $C_8H_{14}O$ $[M]^+$: 126.103915, found: 126.104160.

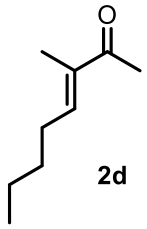

2d

(*E*)-3-methyloct-3-en-2-one (2d)

The title compound **2d** was prepared according to the general procedure mentioned above, in 28.1 mmol scale, the desired product was obtained as a colorless oil (0.705 g, 18%). **^{1}H NMR** (501 MHz, $CDCl_3$): δ 6.62 (t, *J* = 7.3 Hz, 1H), 2.30 (s, 3H), 2.26–2.22 (m, 2H), 1.76 (brs, 3H), 1.48–1.42 (m, 2H), 1.40–1.32 (m, 2H), 0.93 (t, *J* = 7.3 Hz, 3H); **^{13}C NMR** (126 MHz, $CDCl_3$): δ 200.1, 144.0, 137.8, 30.9, 29.0, 25.6, 22.6, 14.0, 11.2; **HRMS**: (GC-EI) (m/z) calculated for $C_9H_{16}O$ $[M]^+$: 140.119565, found: 140.119810.

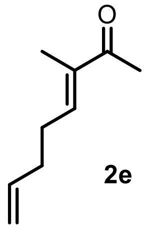

2e

(*E*)-3-methylocta-3,7-dien-2-one (2e)

The title compound **2e** was prepared according to the general procedure, in 28.1 mmol scale, the desired product was obtained as colorless oil (0.51 g, 13%). **^{1}H NMR** (501 MHz, $CDCl_3$): δ 6.61 (td, *J* = 7.1, 1.6 Hz, 1H), 5.86–5.78 (m, 1H), 5.09–5.01 (m, 2H), 2.37–2.31 (m, 2H), 2.30 (s, 3H), 2.25–2.21 (m, 2H), 1.77 (s, 3H); **^{13}C NMR** (126 MHz, $CDCl_3$): δ 200.0, 142.8, 138.1, 137.5, 115.7, 32.7, 28.5, 25.6, 11.4; **HRMS**: (GC-EI) (m/z) calculated for $C_9H_{14}O$ $[M]^+$:138.103915, found:138.104070.

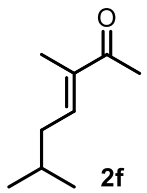

2f

(*E*)-3,6-dimethylhept-3-en-2-one (2f)

The title compound **2f** was prepared according to the general procedure, in 28 mmol scale, the desired product was obtained as colorless oil (1.76 g, 42%). **^{1}H NMR** (501 MHz, $CDCl_3$): δ 6.65 – 6.61 (m, 1H), 2.24 (s, 3H), 2.12 – 2.04 (m, 2H), 1.77 – 1.66 (m, 4H), 0.88 (d, *J* = 6.6 Hz, 6H).; **^{13}C NMR** (126 MHz, $CDCl_3$): δ 199.7, 142.6, 138.0, 38.0, 28.2, 25.3, 22.3, 11.1.; Spectral data were in agreement with literature.[5]

3.3. Procedure for the synthesis of enones 2g-j

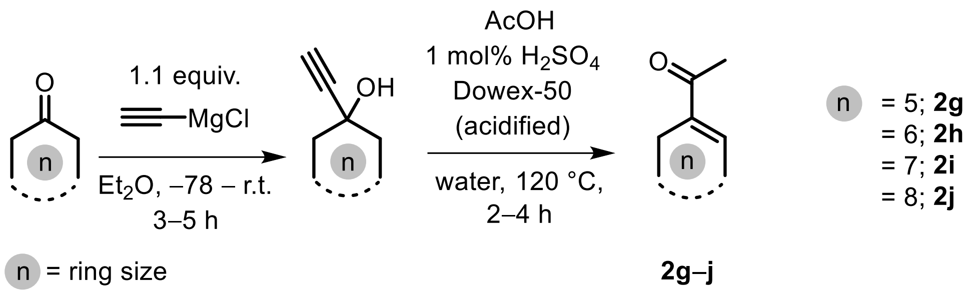

Step-1

A flame-dried Schlenk flask under argon, equipped with a magnetic stirring bar, was charged with a solution of the cyclic ketone (10 mmol) in THF (0.5 M). To this solution, at 0 °C, ethynylmagnesium chloride (1.1 equiv.) was added. Stirring was continued for an additional 3–5 hours at room temperature. Upon completion, the reaction mixture was treated with saturated aqueous NH_4Cl and extracted with Et_2O (3 × 30 mL). The combined organic layers were dried over anhydrous Na_2SO_4 and concentrated under reduced pressure. ^1H NMR analysis of the crude reaction mixture consistently indicated quantitative yield. The crude product was used directly in the next step without further purification.

Step 2[6]

Following a literature procedure, a mixture of crude 1-ethynylcycloalkanol, acetic acid (6.0 equiv.), water (1 mL per 10 mmol of alcohol), Dowex-50 resin (1.0 g per 10 mmol of substrate), and a catalytic amount of H_2SO_4 (one drop) was stirred in a round-bottom flask at 120 °C for 2–4 hours. Upon completion, the reaction mixture was allowed to cool to room temperature, diluted with Et_2O, and the resin was filtered off. The filtrate was then treated with 1 N aqueous NaOH to neutralize excess acid. The organic layer was separated, washed with brine, dried over anhydrous Na_2SO_4, and evaporated. The residue was purified by flash column chromatography using 4–6% Et_2O in hexane as the eluent, and then distilled.

2h was purchased from commercial source and distilled prior to use.

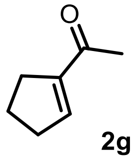

2g

1-(cyclopent-1-en-1-*yl*)ethan-1-one (2g)

The title compound **2f** was prepared according to the general procedure, in 56 mmol scale, the desired product was obtained as colorless oil (2.1 g, 34%). The compound was carefully distilled prior to use: T = 110 °C, p = 60 mbar. **^{1}H NMR** (501 MHz, $CDCl_3$): δ 6.76 – 6.71 (m, 1H), 2.60 – 2.47 (m, 4H), 2.31 (s, 3H), 1.93 (p, *J* = 7.7 Hz, 2H); **^{13}C NMR** (126 MHz, $CDCl_3$): δ 196.8, 146.1, 144.4, 33.9, 30.5, 26.7, 22.9.; Spectral data were in agreement with those reported in literature.[7]

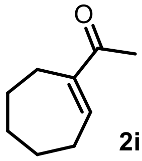

2i

1-(cyclohept-1-en-1-*yl*)ethan-1-one (2i)

The title compound **2h** was prepared using general procedure mentioned above, in 17.8 mmol scale, the desired product was obtained as colorless oil (1.32 g, 54%). **^{1}H NMR** (501 MHz, $CDCl_3$): δ 7.07 (t, *J* = 6.6 Hz, 1H), 2.50–2.48 (m, 2H), 2.36–2.33 (m, 2H), 2.29 (s, 3H), 1.80–1.75 (m, 2H), 1.57–1.53 (m, 2H), 1.48–1.42 (m, 2H); **^{13}C NMR** (126 MHz, $CDCl_3$): δ 199.4, 146.8, 145.7, 32.4, 29.3, 26.2, 26.0, 25.5, 25.4; **HRMS**: (ESI- +) (m/z) calculated for $C_9 H_{15}O$ $[M\ ]^{+}$: 139.111740, found: 139.111980.

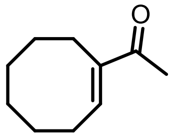

2j

(*E*)-1-(cyclooct-1-en-1-yl)ethan-1-one (2j)

The title compound **2i** was prepared according to general procedure, in 11.9 mmol scale, the desired product was obtained as colorless oil (0.783 g, 43%). **^{1}H NMR** (501 MHz, $CDCl_3$): δ 6.87 (t, *J* = 8.3 Hz, 1H), 2.45–2.43 (m, 2H), 2.36–2.32 (m, 2H), 2.30 (s, 3H), 1.65–1.60 (m, 2H), 1.55–1.50 (m, 2H), 1.48–1.3 (m, 4H); **^{13}C NMR** (126 MHz, $CDCl_3$): δ 199.2, 143.7, 143.3, 29.3, 29.2, 27.7, 26.7, 26.3, 25.6, 23.5; **HRMS**: (GC-EI) (m/z) calculated for $C_{10}H_{16}O$ $[M^{+\bullet}]$: 152.119565, found: 152.119850.

3.4. Procedure for the synthesis of enones 2l-m

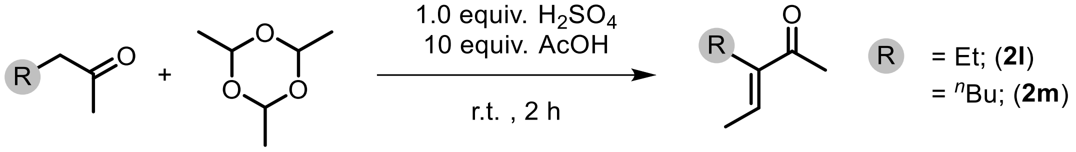

In a round-bottom flask equipped with a magnetic stirring bar and maintained under argon atmosphere, a mixture of the ketone and paraldehyde (1.0 equiv.) in acetic acid (7 mL) was treated dropwise with sulfuric acid at 0 °C. The resulting mixture was stirred at room temperature for 2 hours, then cooled to 0 °C before work-up. The reaction mixture was poured into an ice-cold 2 N aqueous NaOH solution and extracted with Et_2O (3 × 30 mL). The combined organic phases were washed with saturated aqueous NaCl, dried over anhydrous Na_2SO_4, and the solvent was evaporated. The crude product was first purified by column chromatography using 2–5% Et_2O in pentane as the eluent. The isolated product was then distilled under reduced pressure.

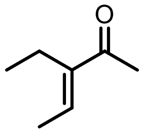

2l

(*E*)-3-ethylpent-3-en-2-one (2l)
The title compound **2i** was prepared according to general procedure, in 12 mmol scale, the desired product was obtained as colorless oil (1.36 g, 25%) distilled: T = 45 °C, p = 70 mbar. **^{1}H NMR** (501 MHz, $CDCl_3$): δ 6.72 (q, J = 7.0 Hz, 1H), 2.34 (m, 2H), 2.31 (s, Hz, 3H), 1.90 (d, *J* = 6.9 Hz, 3H), 0.95 (t, *J* = 7.5 Hz, 3H).; **^{13}C NMR** (126 MHz, $CDCl_3$): δ 199.4, 145.0, 138.1, 25.7, 18.5, 14.6, 13.5.; Spectral data remained in agreement with these reported in literature.[8]

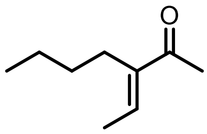

2m

(*E*)-3-butylpent-3-en-2-one (2m)
The title compound **2m** was prepared according to general procedure, in 23 mmol scale, the desired product was obtained as colorless oil (1.2 g, 37%) distilled: T = 65 °C, p = 5 mbar, (oil bath temperature 160 °C). **^{1}H NMR** (501 MHz, $CDCl_3$): δ 6.65 (q, J = 7.0 Hz,

1H), 2.21 (s, 5H), 1.80 (d, J = 6.9 Hz, 3H), 1.35 – 1.10 (m, 4H), 0.83 (t, J = 6.8 Hz, 3H).; **^{13}C NMR** (126 MHz, $CDCl_3$): δ 199.6, 143.8, 138.4, 31.3, 25.8, 25.0, 23.0, 14.8, 14.1.; Spectral data and boiling point in agreement with literature.[9]

3.5. Procedure for the synthesis of enone 2o:

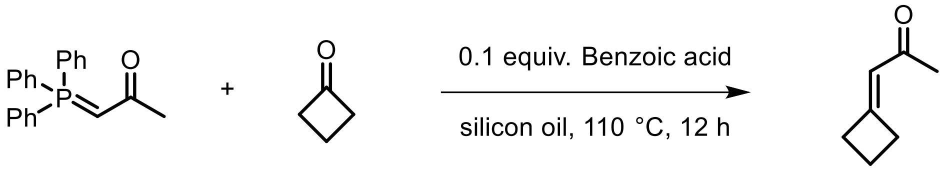

1.0 equiv. 1.1 equiv. **2o**

In a flame-dried Schlenk tube (equipped with a magnetic stirring bar) and maintained under an argon atmosphere, a solution of 1-(triphenylphosphoranylidene)-2-propanone (15 mmol, 5.0 g) in silicone oil (8 mL) was heated to 110 °C. At this temperature, benzoic acid (191.8 mg, 1.6 mmol) and cyclobutanone (17 mmol, 1.18 g) were added, and the mixture was stirred at 110 °C for 12 hours. The product was distilled directly from the reaction mixture and further purified by column chromatography on silica gel using 2–5% Et_2O in pentane as the eluent.

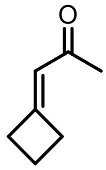

2o

1-cyclobutylidenepropan-2-one (2o)

The title compound **2o** was prepared according to the abovementioned procedure, in 15 mmol scale, the desired product was obtained as colorless liquid (0.73 g, 42%). **^{1}H NMR** (501 MHz, $CDCl_3$): 5.91 (t, *J* = 2.3 Hz, 1H), 3.17 – 3.08 (m, 2H), 2.85 (tt, *J* = 8.0, 2.0 Hz, 2H), 2.13 (d, *J* = 6.9 Hz, 3H), 2.13 – 2.08 (m, 2H); **^{13}C NMR** (126 MHz, $CDCl_3$): δ 198.0, 166.2, 122.4, 34.2, 32.7, 30.0, 17.7.; The obtained spectral data are in agreement with literature.[10]

4. Synthesis of (*E*,*E*)-α-Farnesene (3f)[11,12]

Step 1: Synthesis of α-methyl sulfolene

A 100 mL Young tube equipped with a magnetic stirring bar was charged with a 4:1 (v/v) mixture of HFIP (20 mL) and distilled water (5 mL). The stirred mixture was degassed by freeze–pump–thaw cycles and backfilled with argon. Isoprene (13 mmol) was then added, followed by sodium metabisulfite (65 mmol, 5.0 equiv.). The tube was sealed, and

the reaction mixture was stirred at 100 °C overnight. After cooling to room temperature, the mixture was concentrated under reduced pressure to remove HFIP and unreacted isoprene. The remaining aqueous phase was diluted with brine and extracted with ethyl acetate (3 × 30 mL). The combined organic layers were dried over anhydrous Na_2SO_4 and concentrated under reduced pressure to afford the sulfolene product, which crystallized as a creamy-white solid (a characteristic exothermic effect was observed). The crude crystals were used in the next step without further purification.

Step 2: Low-temperature alkylation

A solution of crude α-methyl sulfolene (1.8 g, 13 mmol) and DMPU (3.8 mL, 32 mmol) in dry THF (60 mL) under an argon atmosphere was cooled to −105 °C using an ethanol/liquid nitrogen bath. Then, 2.0 M n-BuLi in hexane (3.50 mL, 7.00 mmol) was added dropwise while maintaining the internal temperature below −90 °C. The mixture was stirred for 10 minutes before geranyl bromide (2.8 mL, 14.06 mmol) was added in one portion. The solution was allowed to warm slowly to room temperature and stirred for 2 hours. The reaction mixture was concentrated in vacuo, redissolved in Et_2O (100 mL), stirred at room temperature for 10 minutes, and dried over anhydrous Na_2SO_4. The mixture was filtered through Celite and concentrated again under reduced pressure. The crude product was purified by silica gel column chromatography using hexanes:ethyl acetate (8:2) as the eluent. TLC monitoring was performed using permanganate staining. Fractions with Rf = 0.47 were collected, and the compound was used directly in the next step.

Step 3: Retro-[4+1] reaction

A dry Schlenk tube under an argon atmosphere was charged with the sulfolene from the previous step (2.88 g, 10.72 mmol) and dry, degassed toluene (20 mL, stored over molecular sieves). Anhydrous sodium bicarbonate (21.44 mmol, 2.0 equiv.) was added, and the tube was sealed tightly. The reaction mixture was heated to 110 °C and stirred for 4 hours. Upon completion, the crude reaction mixture was purified by chromatography over activated alumina (Al_2O_3), and the product was eluted with pentane. The solvent was removed under reduced pressure, and the residue was distilled (T = 32–35 °C, p = 0.2 mbar) to afford (*E*,*E*)-α-farnesene as a colorless oil (786 mg, 3.84 mmol, 29.6% yield over three steps).

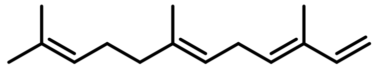

6f

(*E*,*E*)-α-Farnesene (3f)

^{1}H NMR (501 MHz, $CDCl_3$): δ 6.38 (dd, *J* = 17.4, 10.7 Hz, 1H), 5.46 (t, *J* = 7.4 Hz, 1H), 5.17 – 5.06 (m, 3H), 4.93 (d, *J* = 10.7 Hz, 1H), 2.84 (t, *J* = 7.3 Hz, 2H), 2.07 (t, *J* = 7.4 Hz, 2H), 2.00 (dd, *J* = 9.0, 6.1 Hz, 2H), 1.77 (s, 3H), 1.73 – 1.67 (m, 3H), 1.64 (s, 3H), 1.60 (s, 3H). **^{13}C NMR** (126 MHz, $CDCl_3$): δ 141.7, 135.9, 133.9, 132.0, 131.6, 124.4, 122.2, 110.7, 39.8, 27.4, 26.8, 25.8, 17.8, 16.3, 11.8. Boiling point: lit.: 30 – 32 °C, p = 0.19 mbar, observed: 33 – 36 °C, p = 0.21 mbar. Experimental data remained in agreement with literature.[13]

5. General procedure for the racemic Diels-Alder reaction

5.1. Alumina Chloride Catalysed Diels-Alder Reaction (4a-s,v,w)

A flame-dried Young tube (equipped with a magnetic stirring bar, flame-dried, and filled with argon) was charged with the enone (1.0 equiv., 5 mmol) in toluene (5 mL). The flask was then placed in a dry ice–acetone bath and cooled below −40 °C. The diene (2.5 equiv., 12.5 mmol) was added, and the reaction mixture was further cooled to −78 °C. Finely powdered anhydrous $AlCl_3$ (5–10 mol%) was then added in small portions. The reaction mixture was stirred for 1–3 hours in the closed tube under an argon atmosphere, while

maintaining the temperature with the dry ice–acetone bath. Reaction progress was monitored by TLC (8% Et_2O in pentane as eluent, *p*-anisaldehyde stain; product typically appeared as a green spot with Rf between 0.3 and 0.6). If no product formation was observed, the mixture was gradually warmed using an ice bath (0 °C) or to room temperature (23–27 °C). Upon completion, Et_2O (10 mL) was added, followed by 5% aqueous HCl (20 mL). The layers were separated, and the aqueous phase was extracted with Et_2O (3 × 20 mL). The combined organic layers were dried over anhydrous Na_2SO_4 and concentrated under reduced pressure. The crude oil was purified by flash column chromatography using Et_2O–pentane mixtures as eluents. A gradient elution was applied: pure pentane was first used to wash out residual toluene, diene, and diene oligomers, followed by 97:3 pentane:Et_2O to elute the desired product.

Note: The products are volatile; therefore, the rotary evaporator bath temperature was kept below 30 °C to prevent significant yield loss during solvent removal under vacuum.

5.2. IDPi catalyzed synthesis of racemates: 4t and 4u

The cycloadducts of the farnesene isomers could not be obtained using the general racemate preparation protocol. Therefore, compounds **4t** and **4u** were prepared using racemic IDPi-**5c**, following the general procedure for the catalytic enantioselective Diels–Alder reaction (see **Section 5**, next page).

6. General procedure for the catalytic enantioselective Diels-Alder reaction

As an example, the reaction on a 0.2 mmol scale is described. To obtain successful and reproducible results, it is essential to follow all instructions carefully. A freshly flame-dried and filled with argon 2 mL GC vial (equipped with a magnetic stirring bar that was also flame-dried and stored under inert, dry conditions) was charged with IDPi (**5h** or as specified in the individual entry, 5 mol%, 0.05 equiv., 0.01 mmol), 5 Å molecular sieves (50 mg, vacuum-dried at 120 °C for 4 days, p = 10^{-5} mbar), and anhydrous $CHCl_3$ (1 M, 200 µL, water content <15 ppm). Next, the enone (**2a–q**, 1.0 equiv., 0.2 mmol), previously distilled and stored under inert conditions (or alternatively dried in solution in Et_2O over anhydrous Na_2SO_4 before distillation), was added. The reaction vial was sealed, wrapped with parafilm, and cooled to approximately −80 °C using a dry ice bath, until the mixture became a jelly-like semi-solid. After 15 minutes, the diene (4.0 equiv., 0.8 mmol) was added via syringe through the septum. The vial cap was further sealed with a layer of grease and parafilm. The reaction vial was then placed in a cryostat and stirred at the specified temperature for the indicated time. Upon completion, the catalyst was quenched by the addition of dry Et_3N (20 µL), and the mixture was stirred for 10 minutes at the reaction temperature. The reaction mixture was then allowed to warm to room temperature and was directly purified by flash column chromatography on silica gel using 1–3% Et_2O in pentane as the eluent.

Note: The products are volatile; therefore, during solvent removal, the rotary evaporator bath temperature was kept below 30 °C to prevent significant yield loss under vacuum.

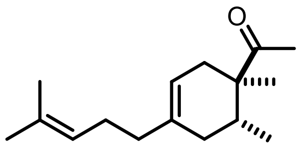

4a

1-((1*R*,6*R*)-1,6-dimethyl-4-(4-methylpent-3-en-1-yl)cyclohex-3-en-1-yl)ethan-1-one (4a)

The title compound was prepared from 19.6 mg of corresponding enone according to the representative procedure at –60 °C for 5 days. The product was purified by column chromatography using 1–3% Et_2O in pentane as eluent and was obtained as colorless oil (46.7 mg, 0.19 mmol, 99% yield, 95:5 e.r., >20:1 r.r.). The compound was also prepared at –80 °C for 7 d, and the desired product **4a** was obtained in 92% yield, 98:2 e.r., >20:1 r.r. **^{1}H NMR** (501 MHz, $CDCl_3$): δ 5.30 – 5.28 (m, 1H), 5.04 – 5.00 (m, 1H), 2.36 (dd, *J*

= 17.2, 2.5 Hz, 1H), 2.17 – 2.08 (m, 4H), 2.02 (q, *J* = 7.3 Hz, 2H), 1.98 – 1.94 (m, 0.5 H), 1.94 – 1.87 (m, 3H), 1.74 (ddt, *J* = 17.2, 4.7, 1.6 Hz, 1H), 1.63 (t, *J* = 1.4 Hz, 3H), 1.59 (ddd, *J* = 8.6, 2.1, 1.0 Hz, 0H), 1.55 (d, *J* = 1.5 Hz, 3H), 0.93 (s, 3H), 0.77 (d, *J* = 6.8 Hz, 3H).; **^{13}C NMR** (126 MHz, $CDCl_3$): δ 214.3, 136.4, 131.4, 124.2, 118.1, 50.3, 37.4, 34.6, 34.1, 32.9, 26.4, 25.7, 25.2, 17.7, 16.3, 15.6; **HRMS**: (GC-EI) (*m*/*z*) calculated for $C_{16}H_{26}O$ $[M]^+$: 234.197815, found: 234.197830; The enantiomeric ratio was determined by GC on a chiral column: BGB-176/BGB-15 0,25/0,25df G/618, temp.: 220 / 100, 450 min iso 6/min 240, 3 min iso / 350, Gas: 0.60 bar H_2 gas; t_R^1 = 400.39 min., t_R^2 = 404.76 min., e.r. = 4.5:95.5; or with HPLC: column with chiral stationary phase: AD-3R, eluent (isocratic) 40:60 (*v*/*v*) as eluent, flow 1 mL/min, column temperature: 25 °C, t_R^1 = 10.27 min., t_R^2 = 12.46 min., e.r. = 2:98; **$[\alpha]_D^{25}$** = –5.000 (c = 0.038, CH_2Cl_2).

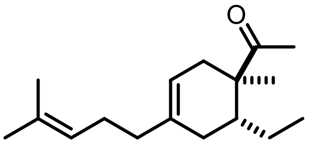

4b

1-((1*R*,6*R*)-6-ethyl-1-methyl-4-(4-methylpent-3-en-1-yl)cyclohex-3-en-1-yl)ethan-1-one (4b)

The title compound was prepared from 22.4 mg of corresponding enone according to the representative procedure at –60 °C for 6 d. The product was purified by column chromatography using 1–3% Et_2O in pentane as eluent and was obtained as colorless oil (49.6 mg, 0.19 mmol, 98%, 97:3 e.r., >20:1 r.r.). **^{1}H NMR** (501 MHz, $CDCl_3$): δ 5.35–5.33 (m, 1H), 5.09–5.06 (m, 1H), 2.41–2.37 (m, 1H), 2.14 (s, 3H), 2.15–2.04 (m, 3H), 1.99–1.97 (m, 2H), 1.92–1.86 (m, 1H), 1.74 (dd, *J* = 17.1, 4.8 Hz, 1H), 1.68 (brs, 3H), 1.61 (s, 3H), 1.65–1.55 (m, 1H), 1.25–1.17 (m, 1H), 1.09–1.01 (m, 1H), 0.98 (s, 3H), 0.92 (t, *J* = 7.4 Hz, 3H); **^{13}C NMR** (126 MHz, $CDCl_3$): δ 214.8, 136.4, 131.6, 124.3, 118.2, 51.0, 40.3, 37.6, 35.3, 30.7, 26.5, 25.9, 25.4, 24.1, 17.9, 15.8, 12.5; **HRMS**: (ESIpos) (m/z) calculated for $C_{17}H_{28}ONa$ $[M+Na]^+$: 271.203234, found: 271.203060; The enantiomeric ratio was determined by GC on a chiral column: BGB-176/BGB-15 0,25/0,25df G/618, temp.: 220/ 110, 350 min. iso 240, 5 min. iso / 350, Gas: 0.60 bar H_2 gas; Retention times: t_R^1 = 267.94 min., t_R^2 = 290.61 min., e.r. = 96:4; or with HPLC: column with chiral stationary phase: AD-3R, eluent (isocratic): water:acetonitrile 40:60 (*v*/*v*) as eluent, flow 1 mL/min, column temperature: 25 °C, t_R^1 = 10.58 min., t_R^2 = 14.22 min., e.r. = 3:97; **$[\alpha]_D^{25}$** = –11.429 (*c* = 0.37, $CHCl_3$).

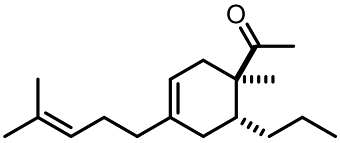

4c

1-((1*R*,6*R*)-1-methyl-4-(4-methylpent-3-en-1-yl)-6-propylcyclohex-3-en-1-yl)ethan-1-one (4c)

The title compound was prepared from 25.2 mg of the corresponding enone according to the representative procedure at –60 °C for 6 days. The product was purified by column chromatography using 1–3% Et_2O in pentane as eluent and was obtained as colorless oil (45.8 mg, 0.18 mmol, 87% yield, 95.5:4.5 e.r., >20:1 r.r.). **^{1}H NMR** (501 MHz, $CDCl_3$): δ 5.36 – 5.33 (m, 1H), 5.07 (t, J = 7.1 Hz, 1H), 2.39 (d, *J* = 17.2 Hz, 1H), 2.14 (s, 3H), 2.07 (t, *J* = 7.5 Hz, 3H), 1.96 (t, *J* = 7.7 Hz, 3H), 1.75 (dd, *J* = 17.0, 4.7 Hz, 1H), 1.68 (s, 3H), 1.60 (s, 4H), 1.52 – 1.39 (m, 1H), 1.22 (dt, *J* = 13.9, 7.3 Hz, 1H), 1.11 – 1.03 (m, 2H), 0.98 (s, 3H), 0.89 (t, *J* = 7.3 Hz, 3H).; **^{13}C NMR** (126 MHz, $CDCl_3$): δ 214.8, 136.5, 131.7, 124.3, 118.2, 50.9, 38.0, 37.6, 35.3, 33.6, 31.2, 26.5, 25.9, 25.4, 20.9, 17.9, 15.8, 14.4.; **HRMS**: (ESIpos) (*m*/*z*) calculated for $C_{18}H_{30}ONa$ $[M+Na]^+$: 285.218884, found: 285.218790; The enantiomeric ratio was determined by HPLC: column with chiral stationary phase: AD-3R, eluent (isocratic): water:acetonitrile 40:60 (*v*/*v*) as eluent, flow 1 mL/min, column temperature: 25 °C, t_R^1 = 13.01 min., t_R^2 = 17.07 min., e.r. = 4.5:95.5; **$[\alpha]_D^{25}$** = –6.677 (*c* = 0.03, CH_2Cl_2).

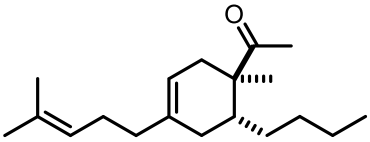

4d

1-((1*R*,6*R*)-6-butyl-1-methyl-4-(4-methylpent-3-en-1-yl)cyclohex-3-en-1-yl)ethan-1-one (4d)

The title compound was prepared from 28 mg of the corresponding enone according to the representative procedure at –60 °C for 7 days. The product was purified by column chromatography using 1–3% Et_2O in pentane as eluent and was obtained as colorless oil (53.1 mg, 0.19 mmol, 96% yield, 96:4 e.r., >20:1 r.r.). **^{1}H NMR** (501 MHz, $CDCl_3$): δ 5.35 – 5.33 (m, 1H), 5.11 – 5.06 (m, 1H), 2.42 – 2.35 (m, 1H), 2.14 (s, 3H), 2.10 – 2.05 (m, 3H), 2.02 – 1.93 (m, 3H), 1.74 (dd, *J* = 17.2, 4.7 Hz, 1H), 1.71 – 1.66 (m, 3H), 1.61 (m, 4H), 1.39 (d, *J* = 5.4 Hz, 1H), 1.31 (ddd, *J* = 11.3, 8.9, 4.8 Hz, 1H), 1.28 – 1.23 (m, 1H), 1.19 (dd, *J* = 10.9, 5.5 Hz, 1H), 1.14 – 1.03 (m, 1H), 0.98 (s, 3H), 0.88 (t, *J* = 7.1

Hz, 3H).; ^{13}**C NMR** (126 MHz, $CDCl_3$): δ 214.8, 136.5, 131.7, 124.3, 118.2, 50.9, 38.3, 37.6, 35.3, 31.3, 31.1, 30.1, 26.5, 25.9, 25.4, 23.0, 17.9, 15.8, 14.2.; **HRMS**: (GC-EI) (m/z) calculated for $C_{19}H_{32}O$ $[M]^+$: 276.244765, found: 276.244760; The enantiomeric ratio was determined by HPLC: column with chiral stationary phase: AD-3R, eluent (isocratic): water:acetonitrile 40:60 (*v*/*v*) as eluent, flow 1 mL/min, column temperature: 25 °C, $t_R{}^1$ = 12.59 min., $t_R{}^2$ = 14.16 min., e.r. = 4:96; $[\alpha]_D^{25}$ = –11.667 (*c* = 0.09, CH_2Cl_2).

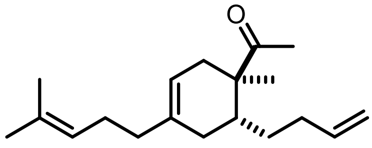

4e

1-((1R,6R)-6-(but-3-en-1-yl)-1-methyl-4-(4-methylpent-3-en-1-yl)cyclohex-3-en-1-yl)ethan-1-one (4e)

The title compound was prepared from 27.6 mg of the corresponding enone according to the representative procedure at –60 °C for 8 days. The product was purified by column chromatography using 1–3% Et_2O in pentane as eluent and was obtained as colorless oil (54.7 mg, 0.19 mmol, 99% yield, 96:4 e.r., >20:1 r.r.). ^{1}**H NMR** (501 MHz, $CDCl_3$): δ 5.78 – 5.74 (m, 1H), 5.35 (ddt, *J* = 4.6, 3.0, 1.5 Hz, 1H), 5.07 (ddt, *J* = 6.9, 5.5, 1.4 Hz, 1H), 5.01 (dq, *J* = 17.1, 1.7 Hz, 1H), 4.96 (ddt, *J* = 10.2, 2.2, 1.2 Hz, 1H), 2.44 – 2.36 (m, 1H), 2.26 – 2.16 (m, 1H), 2.14 (s, 3H), 2.11 – 2.04 (m, 2H), 2.03 – 1.92 (m, 3H), 1.76 (ddt, *J* = 17.1, 4.8, 1.6 Hz, 1H), 1.70 – 1.66 (m, 3H), 1.60 (d, *J* = 1.3 Hz, 4H), 1.31 – 1.11 (m, 2H), 0.99 (s, 3H).; ^{13}**C NMR** (126 MHz, $CDCl_3$): δ 214.6, 138.6, 136.3, 131.7, 124.3, 118.2, 115.0, 50.8, 37.5, 35.2, 31.8, 31.0, 30.6, 26.5, 25.9, 25.4, 17.9, 15.9 (one carbon is missing due to the overlap); **HRMS**: (GC-EI) (*m*/*z*) calculated for $C_{19}H_{30}O$ $[M]^+$: 274.229115, found: 274.229120; The enantiomeric ratio was determined by HPLC: column with chiral stationary phase: AD-3R, eluent (isocratic): water:acetonitrile 40:60 (*v*/*v*) as eluent, flow 1 mL/min, column temperature: 25 °C, $t_R{}^1$ = 12.59 min., $t_R{}^2$ = 14.16 min., e.r. = 4:96; $[\alpha]_D^{25}$ = –16.364 (*c* = 0.07 , CH_2Cl_2).

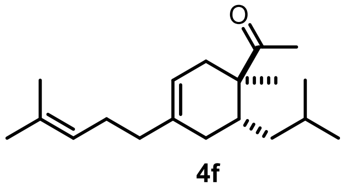

4f

1-((1*R*,6*R*)-6-isobutyl-1-methyl-4-(4-methylpent-3-en-1-yl)cyclohex-3-en-1-*yl*)ethan-1-one (4f)

The title compound was prepared from 28 mg of the corresponding enone according to the representative procedure, using 0.2 mL of pentane:$CHCl_3$ 3:5 (*v*/*v*) mixture as reaction medium, at –60 °C for 8 days. The product was purified by column chromatography using 1–3% Et_2O in pentane as eluents and was obtained as colorless oil (35.6 mg, 0.13 mmol, 64% yield, 93:7 e.r., >20:1 r.r.). **^{1}H NMR**: (501 MHz, $CDCl_3$) δ 5.34 – 5.33 (m, 1H), 5.07 – 5.05 (m, 1H), 2.40 (dq, *J* = 17.4, 2.6 Hz, 1H), 2.12 (s, 3H), 2.06 (dtd, *J* = 12.8, 5.3, 2.6 Hz, 4H), 1.95 (t, *J* = 7.7 Hz, 2H), 1.78 – 1.69 (m, 1H), 1.66 (d, *J* = 1.7 Hz, 3H), 1.61 – 1.57 (m, 4H), 1.09 (ddd, *J* = 14.2, 10.8, 3.7 Hz, 1H), 0.96 (s, 3H), 0.91 – 0.83 (m, 7H), 0.79 (ddd, *J* = 13.0, 10.6, 2.2 Hz, 1H). **^{13}C NMR**: (126 MHz, $CDCl_3$) δ 214.6, 136.4, 131.6, 124.3, 118.2, 50.8, 40.5, 37.5, 35.7, 35.2, 31.3, 26.5, 25.8, 25.3, 25.2, 24.5, 21.3, 17.8, 15.7. **HRMS** (GC-EI) (*m*/*z*) calculated for $C_{19}H_{32}O$ $[M]^+$: 276.244765, found: 276.244970; The enantiomeric ratio was determined by HPLC: column with chiral stationary phase: AD-3R, eluent (isocratic): water:acetonitrile 40:60 (*v*/*v*) as eluent, flow 1 mL/min, column temperature: 25 °C, t_R^1 = 12.29 min., t_R^2 = 17.91 min., e.r. = 7:93; **$[\alpha]_D^{25}$** = –3.529 (*c* = 0.69, $CHCl_3$).

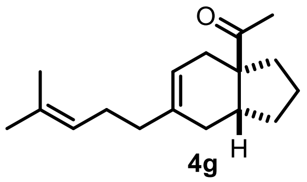

4g

1-((3a*R*,7a*R*)-6-(4-methylpent-3-en-1-yl)-1,2,3,4,7,7a-hexahydro-3aH-inden-3a-yl)ethan-1-one (4g)

The title compound was prepared from 22 mg of the corresponding enone according to the representative procedure at –80 °C for 7 days. The product was purified by column chromatography using 1–3% Et_2O in pentane as eluent and was obtained as colorless oil (48.2 mg, 0.19 mmol, 98%, 96.5:3.5 e.r., >20:1 r.r.). **^{1}H NMR** (501 MHz, $CDCl_3$): δ 5.37–5.35 (m, 1H), 5.07–5.04 (m, 1H), 2.46–2.37 (m, 2H), 2.17–2.11 (m, 1H), 2.14 (s, 3H), 2.08–2.05 (m, 2H), 2.00–1.93 (m, 3H), 1.92–1.86 (m, 1H), 1.86–1.69 (m, 3H), 1.69–1.60 (m, 1H), 1.65 (brs, 3H), 1.61–1.54 (m, 1H), 1.57 (brs, 3H), 1.46–1.38 (m, 1H); **^{13}C NMR** (126 MHz, $CDCl_3$): δ 213.0, 136.7, 131.5, 124.4, 118.0, 56.7, 39.9, 37.7, 36.2, 31.2, 29.8, 29.0, 26.5, 25.8, 25.8, 21.4, 17.8. At spectra of the product isolated from a chemical reaction, the signals of minor regioisomer can be observed. **HRMS**: (GC-EI) (*m*/*z*) calculated for $C_{17}H_{26}O$ $[M]^+$: 246.197815, found: 246.197920; The enantiomeric ratio was determined by GC on a chiral column; GC column: Lipodex-E 0.25/df G/645, temp.: 220/90 900 min iso/350, t_R^1 = 829.98 min., t_R^2 = 875.69 min., e.r. = 4:96; or by HPLC: column with chiral stationary phase: AD-3R, eluent (isocratic): water:acetonitrile

30:70 (*v*/*v*) as eluent, flow 1 mL/min, column temperature: 25 °C, t_R^1 = 7.05 min., t_R^2 = 8.79 min., e.r. = 96.5:3.5; $[\alpha]_D^{25}$ = +32.743 (*c* = 0.226, $CHCl_3$, 96:4 e.r.).

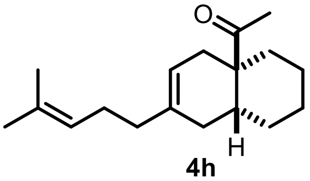

4h

1-((4a*R*,8a*R*)-7-(4-methylpent-3-en-1-yl)-1,3,4,5,8,8a-hexahydronaphthalen-4a(2H)-yl)ethan-1-one (4h)

The title compound was prepared from 24.8 mg of the corresponding enone according to the representative procedure at –60 °C for 8 days. The product was purified by column chromatography using 1–3% Et_2O in pentane as eluent and was obtained as colorless oil (28.1 mg, 0.11 mmol, 54% yield, 97.5:2.5 e.r., >20:1 r.r.). **^{1}H NMR** (501 MHz, $CDCl_3$): δ 5.32–5.30 (m, 1H), 5.05–5.02 (m, 1H), 2.25 (m, 2H), 2.19–2.11 (m, 2H), 2.14 (s, 3H), 2.04–1.98 (m, 2H), 1.94–1.88 (m, 2H), 1.71–1.66 (m, 1H), 1.66 (m, 3H), 1.63–1.58 (m, 1H), 1.58 (s, 3H), 1.55–1.41 (m, 6H), 1.37–1.25 (m, 2H); **^{13}C NMR** (126 MHz, $CDCl_3$): δ 214.2, 136.1, 131.5, 124.4, 117.6, 50.9, 37.6, 34.6, 33.0, 32.8, 28.7, 27.5, 26.5, 25.9, 25.2, 25.0, 22.0, 17.8; **HRMS**: (ESIpos) (*m*/*z*) calculated for $C_{18}H_{28}ONa$ $[M+Na]^+$: 283.203234, found: 283.203130; The enantiomeric ratio was determined by HPLC: column with chiral stationary phase: AD-3R, eluent (isocratic): water:acetonitrile 30:70 (*v*/*v*) as eluent, flow 1 mL/min, column temperature: 25 °C, t_R^1 = 8.38 min., t_R^2 = 12.09 min., e.r. = 97.5:2.5; $[\alpha]_D^{25}$ = +38.307 (*c* = 0.225, CH_2Cl_2).

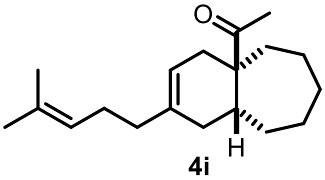

4i

1-((4a*R*,9a*R*)-2-(4-methylpent-3-en-1-yl)-1,4,5,6,7,8,9,9a-octahydro-4aH-benzo[7]annulen-4a-yl)ethan-1-one (4i)

The title compound was prepared from 27.6 mg of the corresponding enone according to the representative procedure at –60 °C for 7 days. The product was purified by column chromatography using 1–3% Et_2O in pentane as eluent and was obtained as colorless oil (54 mg, 0.19 mmol, 98% yield, 98.5:1.5 e.r., >20:1 r.r.). **^{1}H NMR** (501 MHz, $CDCl_3$): δ 5.34 – 5.33 (m, 1H), 5.05–5.01 (m, 1H), 2.40–2.33 (m, 2H), 2.15 (s, 3H), 2.06–2.00 (m, 4H), 1.93–1.88 (m, 2H), 1.80–1.74 (m, 1H), 1.73–1.68 (m, 3H), 1.66 (brs, 3H), 1.58 (s, 3H), 1.57–1.54 (m, 2H), 1.53–1.34 (m, 5H); **^{13}C NMR** (126 MHz, $CDCl_3$): δ 213.6,

136.9, 131.5, 124.4, 118.5, 53.3, 37.6, 37.0, 36.1, 35.2, 32.8, 32.7, 29.3, 27.5, 26.5, 25.9, 25.0, 23.4, 17.8.; **HRMS**: (GC-EI) (m/z) calculated for $C_{19}H_{30}O$ $[M]^+$: 274.229115, found: 274.229400; The enantiomeric ratio was determined by HPLC: column with chiral stationary phase: AD-3R, eluent (isocratic): water:acetonitrile 40:60 (*v*/*v*) as eluent, flow 1 mL/min, column temperature: 25 °C, t_R^1 = 20.65 min., t_R^2 = 28.25 min., e.r. = 98.5:1.5; **$[\alpha]_D^{25}$** = +34.146 (*c* = 0.246, 97:3 e.r. $CHCl_3$).

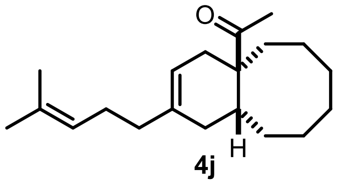

4j

1-((4a*R*,10a*R*)-2-(4-methylpent-3-en-1-yl)-1,5,6,7,8,9,10,10a-octahydrobenzo[8]annulen-4a(4H)-yl)ethan-1-one (4j)

The title compound was prepared from 30.4 mg of the corresponding enone according to the representative procedure at –60 °C for 6 days. The product was purified by column chromatography using 1–3% Et_2O in pentane as eluent and was obtained as colorless oil (57.5 mg, 0.20 mmol, 99% yield, 98:2 e.r., >20:1 r.r.). **^{1}H NMR** (501 MHz, $CDCl_3$) δ 5.34 – 5.33 (m, 1H), 5.02–4.99 (m, 1H), 2.60–2.57 (m, 1H), 2.37 (dd, *J* = 18.0, 5.6 Hz, 1H), 2.27–2.22 (m, 1H), 2.10 (s, 3H), 2.11–2.06 (m, 1H), 2.02–1.97 (m, 2H), 1.92–1.84 (m, 2H), 1.69–1.65 (m, 4H), 1.63–1.55 (m, 10H), 1.53–1.34 (m, 4H), 1.27–1.22 (m, 1H); **^{13}C NMR** (126 MHz, $CDCl_3$) δ 213.6, 137.9, 131.5, 124.4, 118.6, 53.2, 37.7, 35.6, 34.1, 32.1, 32.0, 31.0, 28.8, 28.1, 26.4, 25.9, 25.7, 25.2, 22.6, 17.8; **HRMS** (GC-EI) (m/z) calculated for $C_{20}H_{32}O$ $[M]^+$: 288.244765, found: 288.244950; The enantiomeric ratio was determined by 2D-RP-HPLC: 1D: column: 50 mm Eclipse PAH 1.8 µm 4.6 mm i.D. eluent (isocratic): acetonitrile:water 70:30, flow: 1 mL/min, column temperature 35 °C, chromatogram observed at 220 nm; 2D: column: Chiralcel OJ-3R 4.6 i.D., eluent (isocratic): methanol:water 85:15 (*v*/*v*), flow: 1 mL/min, column oven temperature: 25 °C, chromatograms were monitored at 220 nm; retention times (2D): t_R^1 = 7.74 min., t_R^2 = 9.34 min., e.r. = 2:98; **$[\alpha]_D^{25}$** = +56.678 (*c* = 0.307, 95:5 e.r. $CHCl_3$).

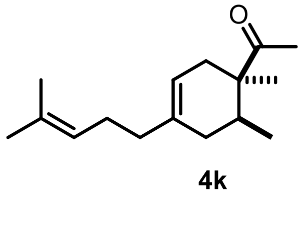

4k

1-((1*R*,6*S*)-1,6-dimethyl-4-(4-methylpent-3-en-1-yl)cyclohex-3-en-1-yl)ethan-1-one (4k)

The product was obtained using a fed-batch reactor setup with a syringe pump. Catalyst **5h** (0.05 equiv., 12 mg) and dry 5 Å molecular sieves (100 mg) were placed in a 5 mL screw-cap vial (equipped with a magnetic stirring bar, flame-dried, evacuated, and filled with argon). The vial was then evacuated and refilled with argon once more. After sealing the vial thoroughly, chloroform (0.5 mL) was added to form a suspension of the molecular sieves in the catalyst solution. The vial was sealed with parafilm and cooled to below −50 °C in a dry ice bath. Myrcene (1.0 mmol, 168 μL) was then added slowly. The vial cap was connected via a cannula to a "dry pressure equilibration vial" (prepared in the same manner, but containing only dried molecular sieves under argon). This equilibration vial was connected to the argon line (see **Fig. S1**). Both vials were placed in a cryostat set to −70 °C for one hour. Meanwhile, a solution of (Z)-enone (0.125 mmol, 15 μL) was prepared in a mixture of pentane (185 μL) and chloroform (300 μL). This solution was transferred to a Hamilton syringe, which was loaded into a syringe pump. The syringe cannula was connected to the reaction vial and left under these conditions for 30 minutes. Subsequently, the dry pressure equilibration vial was removed, and the reaction vial cap and cannula were sealed with grease. The syringe pump was activated (flow rate: 0.05 μL/min), and the reaction vial was maintained at −70 °C until the entire starting material had been added. Work-up was carried out according to the general procedure. The title compound was purified by column chromatography using 1–3% Et_2O in pentane as the eluent and was obtained as a colorless oil (28.4 mg, 0.12 mmol, 97% yield, 99:1 d.r.; 81:19 e.r., >20:1 r.r.). **^{1}H NMR** (501 MHz, $CDCl_3$): δ 5.33 – 5.31 (m, 1H), 5.09 – 5.05 (m, 1H), 2.46 (dt, *J* = 18.0, 2.1 Hz, 1H), 2.35 – 2.25 (m, 1H), 2.11 (s, 3H), 2.09 – 2.01 (m, 3H), 1.96 (t, *J* = 7.7 Hz, 2H), 1.84 – 1.74 (m, 1H), 1.73 – 1.65 (m, 5H), 1.60 (d, *J* = 1.4 Hz, 3H), 1.08 (s, 3H), 0.76 (d, *J* = 6.9 Hz, 3H). (Half of integrals – due to the diastereotopic proton peaks overlapping with other peaks); **^{13}C NMR**: (126 MHz, $CDCl_3$) δ 213.9, 134.3, 131.5, 124.4, 118.3, 49.2, 37.6, 33.6, 32.9, 29.2, 26.6, 25.9, 24.9, 22.8, 17.9, 16.8. **HRMS:** (GC-EI) (m/z) calculated for $C_{16}H_{26}O$ $[M]^+$: 234.197815, found: 234.197830; The diastereomeric and enantiomeric ratios were determined by GC on a chiral column; GC column: BGB-176/BGB-15 0.25/0.25df G/618, temp.: 220/100 450 min iso 6/min 240, 3 min iso/350, t_R^1 = 336,02 min., t_R^2 = 342,92 min., e.r. = 81:19; **$[\alpha]_D^{25}$** = -5.556 (*c* = 0.72, $CHCl_3$)

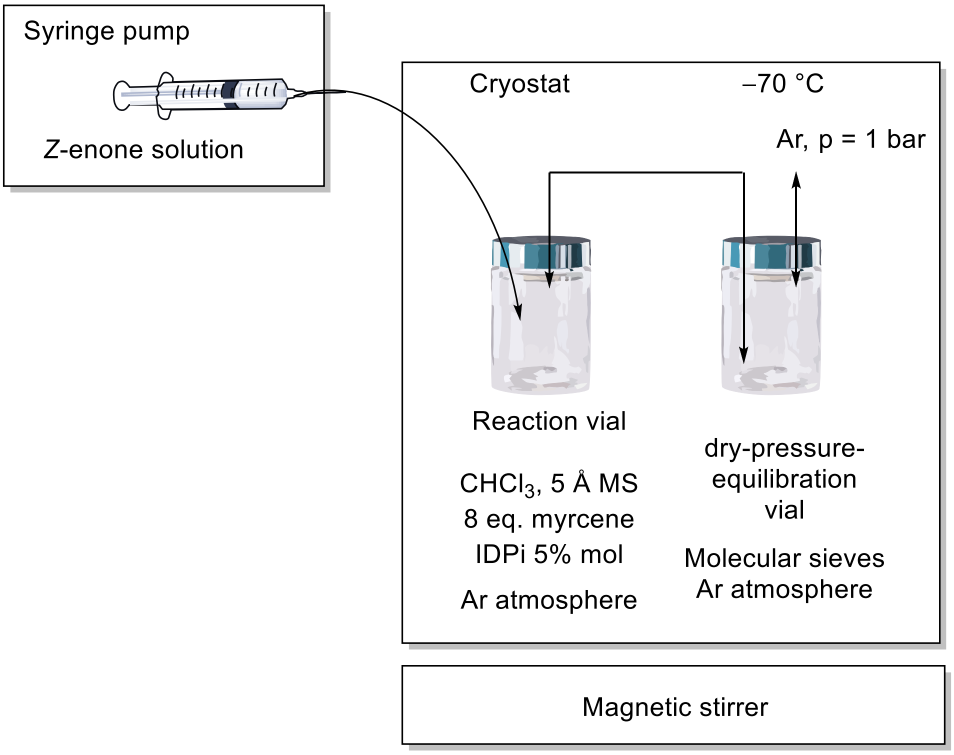

Figure S1. The fed-batch reactor set-up.

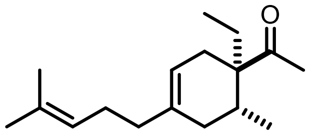

4l

1-((1*R*,6*R*)-1-ethyl-6-methyl-4-(4-methylpent-3-en-1-yl)cyclohex-3-en-1-yl)ethan-1-one (4l)

The title compound was prepared from 22.4 mg of corresponding enone according to the representative procedure at –60 °C for 8 days using chloroform:pentane 5:3 (*v*/*v*) mixture as a solvent. The product was purified by column chromatography using 1–3% Et_2O in pentane as eluent and was obtained as colorless oil (42.9 mg, 0.17 mmol, 86% yield, 93:7 e.r., >20:1 r.r.). **^{1}H NMR** (501 MHz, $CDCl_3$): δ 5.38 – 5.32 (m, 1H), 5.07 – 4.99 (m, 1H), 2.55 – 2.46 (m, 1H), 2.29 – 2.19 (m, 1H), 2.11 (s, 3H), 2.02 (q, *J* = 8.0 Hz, 3H), 1.91 (t, *J* = 6.3 Hz, 2H), 1.83 (s, 1H), 1.66 (t, *J* = 1.4 Hz, 3H), 1.62 – 1.53 (m, 5H), 1.51 – 1.40 (m, 1H), 0.87 (d, *J* = 7.0 Hz, 3H), 0.72 (t, *J* = 7.5 Hz, 3H). **^{13}C NMR** (126 MHz, $CDCl_3$): δ 213.4, 135.9, 131.5, 124.4, 118.6, 53.9, 37.7, 34.3, 31.6, 29.0, 28.2, 26.5, 25.9, 25.7, 17.8, 15.4, 8.6. **HRMS**: (GC-EI) (m/z) calculated for $C_{17}H_{28}O$ $[M]^+$: 248.213465, found: 248.213550. The enantiomeric ratio was determined by HPLC: column with chiral stationary phase: AD-3R, eluent (isocratic): water: acetonitrile 50:50 (*v*/*v*) as eluent, flow 1 mL/min, column temperature: 25 °C, t_R^1 = 32.70 min., t_R^2 = 36.32 min., e.r. = 7:93; **$[\alpha]_D^{25}$** = –3.636 (*c* = 0.45, $CHCl_3$).

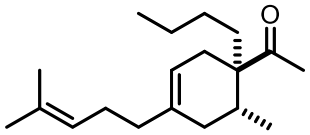

4m

1-((1*R*,6*R*)-1-butyl-6-methyl-4-(4-methylpent-3-en-1-yl)cyclohex-3-en-1-yl)ethan-1-one (4m)

The title compound was prepared from 28 mg of corresponding enone according to the representative procedure at –60 °C for 8 days using chloroform:pentane 5:3 (*v*/*v*) mixture as a solvent. The product was purified by column chromatography using 1–3% Et_2O in pentane as eluent and was obtained as colorless oil (45.8 mg, 0.17 mmol, 82% yield, 91:9 e.r., >20:1 r.r.). **^{1}H NMR** (501 MHz, $CDCl_3$): δ 5.35 (m, 1H), 5.05 – 5.02 (m, 1H), 2.53 – 2.46 (m, 1H), 2.23 (t, *J* = 7.0 Hz, 1H), 2.11 (d, *J* = 2.5 Hz, 3H), 2.02 (q, *J* = 7.4 Hz, 3H),

1.90 (d, J = 7.6 Hz, 2H), 1.82 (d, J = 17.8 Hz, 1H), 1.66 (s, 3H), 1.64 – 1.49 (m, 5H), 1.45 – 1.31 (m, 1H), 1.24 (dq, J = 9.8, 4.2 Hz, 2H), 1.14 – 0.95 (m, 2H), 0.87 (ddd, J = 12.1, 7.4, 2.5 Hz, 6H). **^{13}C NMR** (126 MHz, $CDCl_3$): δ 213.5, 135.8, 131.5, 124.4, 118.7, 53.7, 37.7, 35.7, 34.3, 31.9, 29.4, 26.5, 26.3, 25.9, 25.7, 23.6, 17.9, 15.4, 14.1. **HRMS**: (GC-EI) (m/z) calculated for $C_{19}H_{32}O$ $[M]^+$: 276.244765, found: 276.244860. The enantiomeric ratio was determined by HPLC: column with chiral stationary phase: AD-3R, eluent (isocratic): water:acetonitrile 45:55 (*v*/*v*) as eluent, flow 1 mL/min, column temperature: 25 °C, t_R^1 = 33.78 min., t_R^2 = 40.08 min., e.r. = 9:91; **$[\alpha]_D^{25}$** = +12.000 (c = 0.67, CH_2Cl_2).

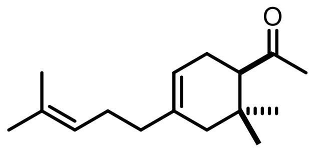

4n

(*R*)-1-(6,6-dimethyl-4-(4-methylpent-3-en-1-yl)cyclohex-3-en-1-yl)ethan-1-one (4n)

A 2 mL flame-dried GC vial equipped with a magnetic stirring bar was charged with IDPi-**5i** (5 mol%, 0.05 equiv., 0.009 mmol), 5 Å molecular sieves (50 mg), and $CHCl_3$ (200 µL). To this suspension, isopropanol (13.3 µL, 0.17 mmol, 1.0 equiv.) and mesityl oxide **2n** (1.0 equiv., 0.174 mmol) were added, and the reaction vial was cooled to −80 °C. After 10 minutes, myrcene (60 µL, 0.35 mmol, 2.0 equiv.) was added through the septum of the reaction vial. The vial was then maintained at −40 °C for 5 days. The reaction was quenched with Et_3N (20 µL) and purified directly by flash column chromatography on silica gel using 1–3% Et_2O in pentane as the eluent, affording the desired product **4n** (26 mg, 0.11 mmol, 64% yield, 93:7 e.r., >20:1 r.r.). **^{1}H NMR** (600 MHz, $CDCl_3$): δ 5.38 – 5.32 (m, 1H), 5.12 – 5.05 (m, 1H), 2.54 (dd, J = 9.8, 5.6 Hz, 1H), 2.17 (s, 3H), 2.13 – 2.02 (m, 3H), 1.97 – 1.85 (m, 3H), 1.74 – 1.66 (m, 4H), 1.59 (d, J = 15.0 Hz, 4H), 1.01 (s, 3H), 0.92 (s, 3H); **^{13}C NMR** (151 MHz, $CDCl_3$): δ 213.0, 137.1, 131.8, 124.6, 118.4, 55.6, 44.5, 38.0, 32.7, 32.5, 29.8, 26.8, 26.5, 26.1, 22.1, 18.1; **HRMS**: (GC-EI) (m/z) calculated for $C_{16}H_{26}O$ $[M]^+$: 234.197815, found: 234.197850; The enantiomeric ratio was determined by HPLC: column with chiral stationary phase: IG-3R 4.6 mm i.D., eluent (isocratic): water:acetonitrile 40:60 (*v*/*v*) as eluent, flow 1 mL/min, column temperature: 25 °C, λ = 220 nm, t_R^1 = 11.54 min., t_R^2 = 13.89 min., e.r. = 93:7. **$[\alpha]_D^{25}$** = –22.857 (c = 0.42, heptane).

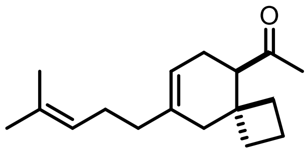

4o

(*R*)-1-(8-(4-methylpent-3-en-1-yl)spiro[3.5]non-7-en-5-yl)ethan-1-one (4o)

The title compound was prepared from 22 mg of the corresponding enone according to the representative procedure at –40 °C for 5 days using IDPi-**5j** as catalyst (5% mol, 0.05 equiv.). The product was purified by column chromatography using 1–3% Et_2O in pentane as eluent and was obtained as colorless oil (48.3 mg, 0.19 mmol, 98% yield, 91:9 e.r., >20:1 r.r.). **^{1}H NMR** (501 MHz, $CDCl_3$) δ 5.33 – 5.32 (m, 1H), 5.13 – 5.04 (m, 1H), 2.61 (t, J = 5.3 Hz, 1H), 2.29 – 2.14 (m, 4H), 2.12 (s, 3H), 2.06 (q, J = 7.8 Hz, 2H), 2.00 – 1.86 (m, 4.5H), 1.74 (m, 2.5H), 1.69 – 1.65 (m, 4H), 1.60 (s, 3H). **^{13}C NMR** (126 MHz, $CDCl_3$) δ 211.4, 137.0, 131.8, 124.7, 118.9, 55.3, 40.2, 39.5, 38.0, 33.2, 31.5, 30.8, 26.8, 26.1, 25.0, 18.1, 15.9. **HRMS** (GC-EI) (m/z) calculated for $C_{17}H_{26}O$ $[M]^+$: 246.197815, found: 246.197760; The enantiomeric ratio was determined by GC on a chiral column; GC column: BGB-178/BGB-15 0.25/0.25df G/615, temp.: 220/100 496 min iso /350, t_R^1 = 456.08 min., t_R^2 = 466.48 min., e.r. = 9:91; **$[\alpha]_D^{25}$** = –15.385 (*c* = 0.44, CH_2Cl_2)

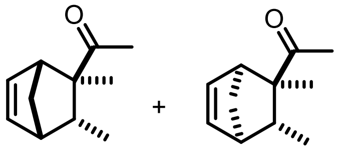

exo *endo*

4p

1-((1*R*,2*S*,3*R*,4*S*)-2,3-dimethylbicyclo[2.2.1]hept-5-en-2-yl)ethan-1-one and 1-((1*S*,2*S*,3*R*,4*R*)-2,3-dimethylbicyclo[2.2.1]hept-5-en-2-yl)ethan-1-one (4p)

The title compound was prepared from 19.6 mg of the corresponding enone according to the representative procedure at –80 °C for 8 days using CH_2Cl_2 (1.0 M) as solvent and in the presence of IDPi-**5k**. The product was purified by column chromatography using 1–3% Et_2O in pentane as eluents and was obtained as colorless oil (17.7 mg, 0.11 mmol, 54% yield, 96.5:3.5 e.r. (*exo*), 56.5:43.5 e.r. (*endo*), 68:32 *exo*:*endo*).**^{1}H NMR** for *exo* diastereoisomer (501 MHz, $CDCl_3$): δ 6.23 (dd, *J* = 5.8, 2.9 Hz, 1H), 6.19 (dd, *J* = 5.8, 3.1 Hz, 1H), 3.00 (s, 1H), 2.66 (s, 1H), 2.51 (qd, *J* = 7.2, 3.5 Hz, 1H), 2.18 (s, 3H), 1.41–1.38 (m, 1H), 1.26–1.20 (m, 1H), 0.91 (s, 3H), 0.82 (d, *J* = 7.3 Hz, 3H); **^{1}H NMR** for

endo diastereoisomer (501 MHz, $CDCl_3$): δ 6.16 (dd, *J* = 5.8, 3.1 Hz, 1H), 6.02 (dd, *J* = 5.8, 2.7 Hz, 1H), 2.71 (s, 1H), 2.39 (s, 1H), 2.14 (qd, *J* = 7.3, 2.0 Hz, 1H), 2.08 (s, 3H), 1.40 (m, 1H), 1.26–1.20 (m, 1H), 1.20 (s, 3H), 1.05 (d, *J* = 7.3 Hz, 3H); **^{13}C NMR** (for both *exo, endo*- diastereoisiomers) (126 MHz, $CDCl_3$): δ 213.5, 138.8, 137.2, 136.1, 134.6, 58.5, 58.2, 52.2, 50.4, 50.0, 48.9, 48.0, 44.2, 38.5, 37.4, 26.5, 26.2, 21.0, 18.3, 17.4, 15.4 (due to low concentration carbonyl signal for the endo isomer was missing); **HRMS**: (GC-EI) (*m/z*) calculated for $C_{11}H_{16}O$ $[M]^+$: 164.119565, found: 164.119660; The enantiomeric ratio was determined by GC on a chiral column: BGB-176/BGB-15 0,25/0,25df G/615, temp.: 220/80, 60 min iso 6/min 230, 3 min iso / 350, Gas: 0.50 bar H_2 gas;, $t_R{}^1$(*exo*) = 37.8 min., $t_R{}^2$(*exo*) = 40.21 min., e.r.*exo* = 96.5:3.5. NMR spectra identical that reported in the literature.[14]

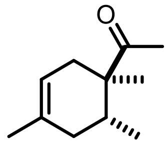

4q

1-((1*R*,6*R*)-1,4,6-trimethylcyclohex-3-en-1-yl)ethan-1-one (4q)

The title compound was prepared from 19.6 mg of the corresponding enone according to the representative procedure at –60 °C for 5 days. The product was purified by column chromatography using 1–3% Et_2O in pentane as eluent and was obtained as colorless oil (27.3 mg, 0.16 mmol, 82% yield, 93.5:6.5 e.r., 20:1 r.r.). **^{1}H NMR** (501 MHz, $CDCl_3$): δ 5.31 – 5.30 (m, 1H), 2.37 (dt, *J* = 17.1, 2.6 Hz, 1H), 2.23 – 2.11 (m, 4H), 2.01 – 1.92 (m, 1H), 1.79 – 1.68 (m, 1H), 1.66 – 1.57 (m, 4H), 0.97 (s, 3H), 0.80 (d, *J* = 6.8 Hz, 3H); **^{13}C NMR** (126 MHz, $CDCl_3$): δ 214.6, 132.8, 118.3, 50.3, 36.0, 34.7, 32.9, 25.4, 23.4, 16.4, 15.8; **HRMS**: **(GC-EI)** (m/z) calculated for $C_{11}H_{18}O$ $[M]^+$: 166.135215, found: 166.135270; The enantiomeric ratio was determined by GC on a Chiral column: Hydrodex-gamma-TBDAc-CD 0,251/dt G/624, temp.: 220/60 120 min iso 8/min 220 3min iso/350, Gas: 0.50 bar H_2 gas;, $t_R{}^1$ = 101.17 min., $t_R{}^2$ = 106.7 min., e.r. = 93.5:6.5; **$[\alpha]_D^{25}$** = –3.478 (*c* = 0.14, heptane).

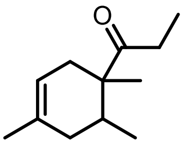

4q'

***rac*-1-(1,4,6-trimethylcyclohex-3-en-1-yl)propan-1-one**

The title compound was prepared according to the representative procedure for racemate synthesis using 86.9 mg (0.77 mmol, 0.1 mL) of the corresponding enone. Product was purified by column chromatography using 1–3% Et_2O in pentane as eluent and was obtained as colorless oil (63 mg, 0.35 mmol, 45% yield, 10:3 r.r.). The number of chemical shifts may differ due to the two regioisomers obtained. ^{1}H NMR integrals are not necessarily expressed in natural numbers because of overlaps of signals from two regioisomers. **^{1}H NMR** (501 MHz, $CDCl_3$): δ 5.32 – 5.30 (m, 1H), 5.15 (m, 0.29H), 2.74 – 2.67 (m, 0.28H), 2.61 – 2.32 (m, 3.79H), 2.24 – 2.14 (m, 0.89H), 2.00 – 1.89 (m, 1.5H), 1.80 – 1.71 (m, 1.24H), 1.68 – 1.49 (m, 5H), 1.07 – 0.99 (m, 8H), 0.82 – 0.76 (m, 4H). **^{13}C NMR** (126 MHz, $CDCl_3$): δ 217.2, 216.8, 132.9, 126.5, 119.7, 118.6, 50.1, 36.2, 35.1, 34.6, 33.2, 32.7, 31.62, 30.50, 30.48, 30.2, 27.3, 23.8, 23.5, 23.4, 16.9, 16.6, 16.4, 16.1, 16.0, 8.5, 8.5. **HRMS**: (ESIpos) (m/z) calculated for $C_{12}H_{21}O$ $[M]^+$: 181.158690, found: 181.158760.

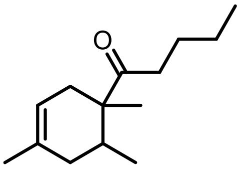

4q''

***rac*-1-(1,4,6-trimethylcyclohex-3-en-1-yl)pentan-1-one**

The title compound was prepared according to the representative procedure for racemate synthesis using 260.7 mg (1.86 mmol, 0.3 mL) of the corresponding enone. Product was purified by column chromatography using 1–3% Et_2O in pentane as eluent and was obtained as colorless oil (117 mg, 0.562 mmol, 30% yield, 10:1.7 r.r.). The number of chemical shifts may differ due to the two regioisomers obtained. The integrals are not necessarily expressed in natural numbers because of overlaps of signals from two regioisomers. **^{1}H NMR** (501 MHz, $CDCl_3$): δ 5.31 – 5.29 (m, 1H), 5.14 – 5.13 (m, 0.13H), 2.73 – 2.67 (m, 0.15H), 2.55 – 2.33 (m, 3.63H), 2.23 – 2.13 (m, 1H), 2.01 – 1.88 (m, 1.3H), 1.79 – 1.66 (m, 1.21H), 1.68 – 1.57 (m, 4.37H), 1.55 – 1.48 (m, 1.66H), 1.33 –

1.22 (m, 3H), 1.06 – 0.95 (m, 3.68H), 0.90 – 0.86 (m, 3.75H), 0.80 – 0.76 (m, 3.58H). **^{13}C NMR** (126 MHz, $CDCl_3$): δ 216.4, 216.1, 132.7, 131.9, 126.4, 119.6, 118.5, 52.0, 51.0, 50.0, 49.7, 41.9, 41.3, 39.2, 36.9, 36.6, 36.1, 35.2, 35.1, 34.8, 34.6, 34.4, 32.9, 32.5, 32.38, 32.36, 31.4, 31.3, 29.7, 29.2, 27.2, 26.3, 26.2, 26.0, 25.9, 23.7, 23.33, 23.27, 23.0, 23.0, 22.6, 22.6, 18.1, 16.7, 16.5, 16.3, 16.2, 16.1, 15.9, 15.8, 15.5, 14.2, 14.1, 14.0, 13.1, 11.3. **HRMS**: (ESIpos) (m/z) calculated for $C_{14}H_{24}O$ $[M]^+$: 231.171934, found: 231.172140.

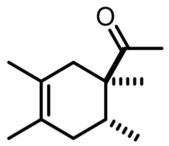

4r

1-((1*R*,6*R*)-1,3,4,6-tetramethylcyclohex-3-en-1-yl)ethan-1-one (4r)

The title compound was prepared from 19.6 mg of the corresponding enone according to the representative procedure at –60 °C for 5 days. The product was purified by column chromatography using 1–3% Et_2O in pentane as eluent and was obtained as colorless oil (36 mg, 0.2 mmol, 99% yield, 98:2 e.r.). **^{1}H NMR** (501 MHz, $CDCl_3$): δ 2.39 – 2.31 (m, 1H), 2.12 (s, 4H), 2.00 – 1.91 (m, 1H), 1.70 – 1.62 (m, 2H), 1.61 – 1.56 (m, 6H), 0.96 (s, 3H), 0.78 (d, J = 6.8 Hz, 3H); **^{13}C NMR** (126 MHz, $CDCl_3$): δ 214.4, 124.3, 122.9, 51.2, 41.0, 37.6, 33.1, 25.4, 19.1, 18.8, 16.3, 15.7; **HRMS**: (GC-EI) (m/z) calculated for $C_{12}H_{20}O$ $[M]^+$: 180.150865, found: 180.150750; The enantiomeric ratio was determined by GC on a Chiral column: Hydrodex-gamma-TBDAc-CD 0,251/dt G/624, temp.: 220/65 150 min iso 8/min 230 3min iso/350, Gas: 0.60 bar H_2 gas;, t_R^1 = 188.82 min., t_R^2 = 211.79 min.,, e.r. = 2:98; **$[\alpha]_D^{25}$** = –16.832 (c = 0.202, $CHCl_3$).

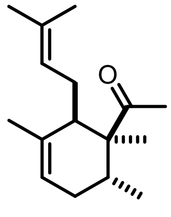

4s

1-((1*S*,2*R*,6*R*)-1,3,6-trimethyl-2-(3-methylbut-2-en-1-yl)cyclohex-3-en-1-yl)ethan-1-one (4s)

The title compound was prepared from 19.6 mg of the corresponding enone according to the representative procedure at –80 °C for 8 days in the presence of IDPi-**5k** as catalyst.

The product was purified by column chromatography using 1–3% Et_2O in pentane as eluent and was obtained as colorless oil (45.8 mg, 0.19 mmol, 98% yield, 93:7 e.r., >20:1 d.r., >20:1 r.r.). **^{1}H NMR** (501 MHz, $CDCl_3$): 5.30 – 5.26 (m, 1H), 5.03 – 4.98 (m, 1H), 2.29 (dt, *J* = 10.6, 6.5 Hz, 1H), 2.19 – 2.10 (m, 1H), 2.09 (s, 3H), 1.98 – 1.85 (m, 3H), 1.72 (q, *J* = 1.9 Hz, 3H), 1.64 (d, *J* = 1.6 Hz, 3H), 1.53 (s, 3H), 1.04 (s, 3H), 0.83 (d, *J* = 6.5 Hz, 3H).; **^{13}C NMR** (126 MHz, $CDCl_3$): δ 213.1, 135.2, 132.3, 124.5, 121.0, 53.5, 50.5, 32.6, 31.0, 27.5, 26.5, 26.2, 24.2, 18.2, 17.6, 17.0; **HRMS**: (GC-EI) (m/z) calculated for $C_{16}H_{26}O$ $[M]^+$: 234.197815, found: 234.197860; The enantiomeric ratio was determined by HPLC on a chiral stationary phase; HPLC column: IG-3R, solvent system: CH_3CN/H_2O = 60/40, flow rate: 1.0 mL/min., temp.: 25 °C, 220 nm, t_R^1 = 8.6 min., t_R^2 = 10.6 min., e.r. = 7:93; **$[\alpha]_D^{25}$** = +136.620 (*c* = 0.142, $CHCl_3$).

NMR analysis to confirm the relative stereochemistry of the desired product

NMR data analysis supports the following structure

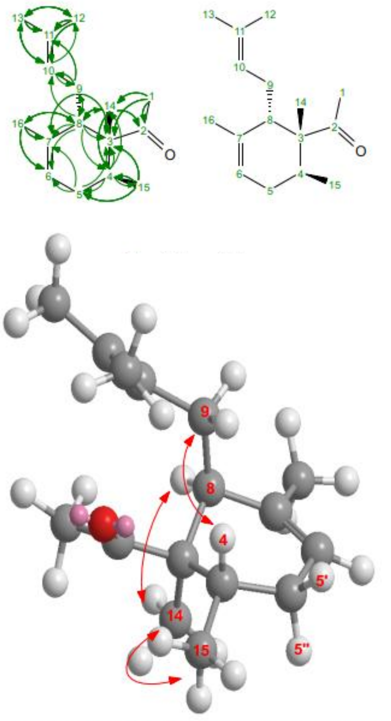

Atom	δ (ppm)	J	COSY	HSQC	HMBC	NOESY
1 C	27.26			1		
H3	2.09			1	2, 3, 14	14
2 C	212.84				1, 14	
3 C	53.28				1, 4, 5', 8, 9, 14, 15	
4 C	26.21			4	8, 14, 15	
H	2.29		5', 5", 15	4	3, 5, 14, 15	9, 15
5 C	32.37			5', 5"	4, 15	
H'	2.13		4, 5", 6, 16	5	3, 6, 7	6, 15
H"	1.55		4, 5', 6, 16	5		6, 14, 15
6 C	120.71			6	5', 8, 16	
H	5.28		5', 5", 16	6		5', 5", 16
7 C	134.89				5', 8, 9, 16	
8 C	50.22			8	9, 14, 16	
H	1.88		9	8	3, 4, 6, 7, 9, 10, 14, 16	14, 16
9 C	30.70			9	8, 10	
H2	1.95		8, 10, 12, 13	9	3, 7, 8, 10, 11	4, 12, 16
10 C	124.26			10	8, 9, 12, 13	
H	5.00		9, 12, 13	10	9, 12, 13	13
11 C	132.05				9, 12, 13	
12 C	17.98			12	10, 13	
H3	1.53		9, 10	12	10, 11, 13	9
13 C	25.90			13	10, 12	
H3	1.64		9, 10	13	10, 11, 12	10
14 C	16.76			14	1, 4, 8	
H3	1.04	6.50(15)		14	2, 3, 4, 8	1, 5", 8
15 C	17.37			15	4	
H3	0.82	6.50(14)	4	15	3, 4, 5	4, 5', 5"
16 C	23.92			16	8	
H3	1.72		5', 5", 6	16	6, 7, 8	6, 8, 9

Table S1. Peak table for the product **4o**, COSY, HSQC, HMBC, NOESY signals for the assignment of relative stereochemistry. (Analysis was carried out using racemic sample).

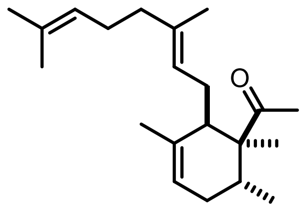

4t

1-((1*S*,2*R*,6*R*)-2-((*E*)-3,7-dimethylocta-2,6-dien-1-yl)-1,3,6-trimethylcyclohex-3-en-1-yl)ethan-1-one (4t)

The title compound was prepared from 19.6 mg of the corresponding enone and 81.7 mg (2 equiv., 0.4 mmol) of **3f**, according to the representative procedure at –80 °C for 8 days in the presence of IDPi-**5l** as catalyst. The product was purified by column chromatography using 1–3% Et_2O in pentane as eluent and was obtained as colorless oil (40.6 mg, 0.13 mmol, 67% yield, 92.5:7.5 e.r., >20:1 d.r., >20:1 r.r.). The integrals are not necessarily expressed in natural numbers because of peak overlaps. **^{1}H NMR** (501 MHz, $CDCl_3$): δ 5.29 – 5.27 (m, 1H), 5.10 – 5.06 (m, 1H), 5.05 – 5.02 (m, 1H), 2.30 (dt, *J* = 10.6, 6.6 Hz, 1H), 2.17 – 2.11 (m, 1H), 2.10 (s, 3H), 2.07 – 2.00 (m, 2.5H), 2.00 – 1.86 (m, 4.5H), 1.72 (q, *J* = 1.9 Hz, 3H), 1.67 (d, *J* = 1.4 Hz, 3H), 1.59 (d, *J* = 1.2 Hz, 3H), 1.53 (d, *J* = 1.3 Hz, 3H), 1.04 (s, 3H), 0.82 (d, *J* = 6.5 Hz, 3H). **^{13}C NMR** (126 MHz, $CDCl_3$): δ 212.8, 135.6, 134.9, 131.5, 124.5, 124.1, 120.7, 53.4, 50.2, 40.0, 32.4, 30.6, 27.3, 26.7, 26.3, 25.8, 24.0, 17.8, 17.4, 16.8, 16.3. **HRMS**: (GC-ESI) (m/z) calculated for $C_{21}H_{34}O$ $[M]^+$: 302.260415, found: 302.260430; The enantiomeric ratio was determined by HPLC on a chiral stationary phase; HPLC column: AD-3R, solvent system: CH_3CN : H_2O 50 : 50 (*v*/*v*), flow rate: 1.0 mL/min., temp.: 25 °C, 220 nm, λ = 210 nm, t_R^1 = 51.32 min., t_R^2 = 55.75 min., e.r. = 7.5:92.5; **$[\alpha]_D^{25}$** = +55.213 (*c* = 0.082 M, $CDCl_3$).

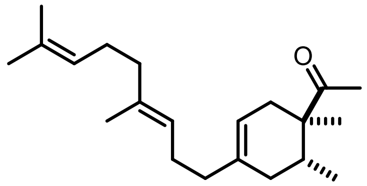

4u

1-((1*R*,6*R*)-4-((*E*)-4,8-dimethylnona-3,7-dien-1-yl)-1,6-dimethylcyclohex-3-en-1-yl)ethan-1-one (4u)

The title compound was prepared from 19.6 mg of the corresponding enone according to the representative procedure at –60 °C for 5 days. The product was purified by column

chromatography using 1–3% Et_2O in pentane as eluent and was obtained as colorless oil (60.1 mg, 0.2 mmol, 99% yield, >20:1 r.r., 97:3 e.r.). **^{1}H NMR:** (501 MHz, $CDCl_3$): δ 5.35 – 5.30 (m, 1H), 5.10 – 5.05 (m, 2H), 2.44 – 2.35 (m, 1H), 2.18 – 2.12 (m, 4H), 2.10 – 2.01 (m, 5H), 2.01 – 1.92 (m, 5H), 1.81 – 1.73 (m, 1H), 1.68 – 1.65 (m, 3H), 1.59 (s, 6H), 0.97 (s, 3H), 0.80 (d, *J* = 6.8 Hz, 3H). **^{13}C NMR**: (126 MHz, $CDCl_3$) δ 214.2, 136.2, 135.0, 131.1, 124.2, 123.9, 117.9, 50.1, 39.6, 37.2, 34.5, 34.0, 33.0, 26.6, 26.1, 25.6, 25.1, 17.5, 16.2, 15.9, 15.4. **HRMS**: (GC-ESI) (m/z) calculated for $C_{21}H_{34}O$ $[M]^+$: 302.260415, found: 302.260410; The enantiomeric ratio was determined by HPLC on a chiral stationary phase; HPLC column: AD-3R, solvent system: CH_3CN : H_2O 60 : 40 (*v*/*v*), flow rate: 1.0 mL/min., temp.: 25 °C, λ = 210 nm, t_R^1 = 29.17 min., t_R^2 = 38.40 min., e.r. = 3:97; **$[\alpha]_D^{25}$** = -4.000 (*c* = 0.033 M, $CDCl_3$).

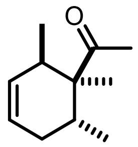

4v

1-((1*S*,2*R*,6*R*)-1,2,6-trimethylcyclohex-3-en-1-yl)ethan-1-one (4v)

The title compound was prepared from 19.6 mg of the corresponding enone according to the representative procedure at –80 °C for 8 days in the presence of catalyst IDPi-**5m** and piperylene (**3h**) as a diene. The product was purified by column chromatography using 1–3% Et_2O in pentane as eluent and was obtained as colorless oil (31.8 mg, 0.19 mmol, 96% yield, 91:9 e.r., >20:1 r.r., >20:1 *endo*:*exo*). **^{1}H NMR:** (501 MHz, $CDCl_3$): δ 5.62 – 5.51 (m, 2H), 2.24 (dt, *J* = 10.5, 6.4 Hz, 1H), 2.14 – 2.04 (m, 5H), 1.62 (ddd, *J* = 18.5, 10.6, 2.1 Hz, 1H), 1.14 (s, 3H), 0.85 (d, *J* = 6.5 Hz, 3H), 0.81 (d, *J* = 7.1 Hz, 3H); **^{13}C NMR** (126 MHz, $CDCl_3$): δ 212.6, 130.3, 124.9, 52.7, 39.1, 31.9, 27.0, 26.6, 18.2, 17.1, 16.8.; **HRMS**: (GC-EI) (m/z) calculated for $C_{11}H_{18}O$ $[M]^+$: 166.135215, found: 166.135360; The enantiomeric ratio was determined by GC on a Chiral column: BGB-178/BGB-15 0,25/0,25df G/615, temp.: 220/55, 240 min iso 8/min 230, 3 min iso/350, Gas: 0.50 bar H_2 gas;, t_R^1 = 142.9 min., t_R^2 = 160.8 min., e.r. = 91:9; **$[\alpha]_D^{25}$** = +167.509 (*c* = 0.139 M, $CHCl_3$).

NMR analysis to confirm the relative stereochemistry of the desired product

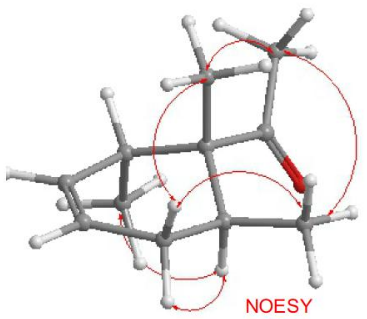

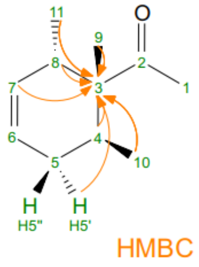

Major						
Atom	δ (ppm)	J	COSY	HSQC	HMBC	NOESY
1C	26.84			1		
H3	2.11			1	2	9, 11
2C	212.47				1, 4, 8, 9	
3C	52.48				H5', 4, 7, 8, 9, 10, 11	
4C	26.46			4	H5', 6, 8, 9, 10	
H	2.23	6.60(10), 10.60(H5"), 6.30(H5')	H5", H5', 10	4	2, 3, 5, 8, 10	H5', 10, 11
5C	31.69			H5", H5', 5', 5"	4, 6, 7, 10	
H5" H	1.62	2.20(7), 10.60(4), -18.30(H5')	H5', 4, 6, 7, 10	5		9, 10
H5' H	2.08	6.30(4), -18.30(H5")	H5", 4, 6, 7	5	3, 4, 6, 7, 10	4
6C	124.7			6	H5', 5", 8	
H	5.54	10.00(7)	H5", H5', 7	6	4, 5, 8	
7C	130.12			7	H5', 8, 11	
H	5.58	10.00(6), 4.50(8), 2.20(H5")	H5", H5', 6, 8	7	3, 5, 8	11
8C	38.93			8	4, 6, 7, 9, 11	
H	2.12	7.10(11), 4.50(7)	7, 11	8	2, 3, 4, 6, 7, 9, 11	
9C	16.67			9	8	
H3	1.13			9	2, 3, 4, 8	H5", 1, 10
10C	16.92			10	H5', 4	
H3	0.85	6.60(4)	H5", 4	10	3, 4, 5	H5", 4, 9
11C	17.99			11	8	
H3	0.81	7.10(8)	8	11	3, 7, 8	1, 4, 7

Table S2. Peak table for the product **4v**, COSY, HSQC, HMBC, NOESY signals for the assignment of relative stereochemistry. (Analysis was carried out using racemic sample).

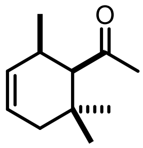

4w

1-((1*R*,2*R*)-2,6,6-trimethylcyclohex-3-en-1-yl)ethan-1-one (4w)

A 2 mL flame-dried GC vial equipped with a magnetic stirring bar was charged with IDPi-**5c** (2 mol%, 0.017 mmol, 0.02 equiv.), 5 Å molecular sieves (200 mg), and methylcyclohexane (CyMe, 1 mL). To this suspension, 1,1,1,3,3,3-hexafluoroisopropanol (HFIP, 0.57 mmol, 0.65 equiv., 60 µL; to get concentration in CyMe: c = 0.57 M) and mesityl oxide **2n** (1.0 equiv., 0.87 mmol, 100 µL) were added. The reaction vial was then cooled to −80 °C. After 10 minutes, piperylene **3h** (2.0 equiv., 1.74 mmol, 174 µL) was added via syringe through the septum. The vial was then placed in a cryostat set to −40 °C and stirred for 4 days. The reaction mixture was then treated with Et_3N (approx. 40 µL) and stirred for an additional 10 minutes. The vial was allowed to warm to room temperature and the crude mixture was purified directly by flash column chromatography on silica gel using 1–3% Et_2O in pentane as the eluent, affording the desired product **4w** in 95% yield (137 mg, 0.82 mmol, 96:4 e.r., >20:1 r.r., >20:1 d.r.). **^{1}HNMR** (501 MHz, $CDCl_3$): δ 5.67 (ddt, *J* = 10.1, 5.1, 2.6 Hz, 1H), 5.43 – 5.36 (m, 1H), 2.59 (d, *J* = 6.3 Hz, 1H), 2.53 – 2.44 (m, 1H), 2.17 – 2.11 (m, 4H), 1.65 (ddt, *J* = 17.8,

3.2, 1.6 Hz, 1H), 1.00 – 0.89 (m, 9H). **^{13}C NMR** (126 MHz, $CDCl_3$): δ 212.7, 129.0, 126.2, 60.7, 36.1, 35.0, 32.3, 30.3, 29.3, 29.0, 17.9. **HRMS** (GC-ESI) (m/z) calculated for $C_{11}H_{18}O$ $[M]^+$: 166.135215, found: 166.135260; The enantiomeric ratio was determined by GC on a Chiral column: BGB-176/BGB-30 0,25/0,25df, temp.: 220/55, 80 min 1/min 220, 5 min iso/350, Gas: 0.50 bar He gas; t_R^1 = 24.18 min., t_R^2 = 25.27 min., e.r. = 97:3; **$[\alpha]_D^{25}$** = -221.5 (*c* = 0.048 M, $CDCl_3$).

Enantiomer of compound **4w** (*ent*-**4w**) was synthesized using exactly the same procedure with (*R*,*R*)-IDPi-**5c** as colorless oil (106.7 mg, 0.64 mmol, 74%, 4:96 e.r., >20:1 d.r., >20:1 r.r.).

7. Syntheses of IsoESuper and IsoESuper plus(Arborone)

7.1. Isomerisation of the Endocyclic Double Bond of PrecyclemoneE (4a)

A Young-Schlenk tube equipped with a magnetic stirring bar was flame-dried and filled with argon. The tube was then charged with anhydrous $KHSO_4$ (10 mol%, 48 mg) and compound **4a** (840 mg, 3.6 mmol). It was sealed tightly and heated at 190 °C for 4 hours. The reaction progress was monitored by ^{1}H NMR spectroscopy. When the ratio of the enocyclic isomer **6a** to **4a** reached approximately 7:10, the reaction was allowed to cool to room temperature and was directly subjected to purification by flushing through a 1 cm layer of silica using 5% Et_2O in pentane as the eluent, followed by exhaustive solvent evaporation. The resulting mixture of isomers was used directly in the subsequent cyclization step. For spectroscopic characterization, the product **6a** was isolated from the reaction mixture via preparative HPLC using a 100 mm Zorbax RX-Sil column (1.8 µm, 4.6 mm) with a mixture of iso-hexane:MTBE (99.5:0.5, v/v) as the eluent, at a flow rate of 1 mL/min and a temperature of 25 °C (described in Section 6.4.1.). The final product was obtained as a colorless liquid.

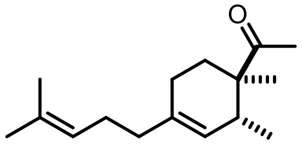

6a

1-((1*R*,2*R*)-1,2-dimethyl-4-(4-methylpent-3-en-1-yl)cyclohex-3-en-1-yl)ethan-1-one (6a)

^{1}HNMR (501 MHz, $CDCl_3$): δ 5.17 – 5.15 (m, 1H), 5.08 – 5.05 (m, 1H), 2.70 (dtd, *J* = 12.2, 6.2, 3.8 Hz, 1H), 2.14 (s, 3H), 2.06 (q, *J* = 7.6 Hz, 2H), 2.00 – 1.89 (m, 4H), 1.77 (ddd, *J* = 12.8, 9.1, 6.6 Hz, 1H), 1.67 (d, *J* = 1.5 Hz, 3H), 1.62 – 1.50 (m, 4H), 0.97 (s, 3H), 0.82 (d, *J* = 7.2 Hz, 3H). **^{13}CNMR** (126 MHz, $CDCl_3$): δ 214.4, 135.6, 131.6, 126.1, 124.3, 50.1, 37.3, 34.5, 31.5, 26.6, 25.8, 25.4, 25.3, 17.8, 16.8, 15.9. **HRMS**: (GC-EI) (m/z) calculated for $C_{16}H_{26}O$ $[M]^+$:234.197815, found: 234.198030; The enantiomeric excess of the product was determined by GC on a Chiral column: BGB-176/BGB-15 0,25/0,25df G/618, temp.: 220/100 450 min iso 6/min 240, 5 min iso/350, Gas: 0.50 bar H_2 gas; t_R^1 = 386.18 min., t_R^2 = 391.83 min., e.r. = 94:6;. Preparative HPLC (achiral): t_R = 128.19 min at third cycle.

7.2. Cyclisation of Precyclemone E to IsoESuper

A 2 mL pressure vial equipped with a magnetic stirring bar was flame-dried, evacuated, and filled with argon. The vial was then charged with wet $KHSO_4$ (5 mol%, 2.5 mg), HFIP (0.26 mL), and compound **4a** (100 mg, 3.6 mmol). The vial was sealed tightly and heated at 80 °C for 4 hours. The reaction progress was monitored by TLC (6% Et_2O in pentane, visualized with *p*-anisaldehyde stain: product appeared as a strong yellow spot; starting material as a green spot). Upon complete consumption of the starting material, the reaction mixture was allowed to cool to room temperature and was directly purified by column chromatography on silica gel using 5% Et_2O in pentane as the eluent to afford product **1c** as colorless oil (96.3 mg, 3.45 mmol, 96%, 95:5 e.r. if enantioenriched **4a** 95:5 e.r. was used).

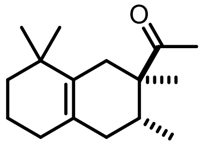

1c

1-((2*R*,3*R*)-2,3,8,8-tetramethyl-1,2,3,4,5,6,7,8-octahydronaphthalen-2-yl)ethan-1-one – IsoESuper, Isocyclemone (1c)

^{1}H NMR (600 MHz, $CDCl_3$): δ 2.28 (dp, *J* = 16.4, 2.5 Hz, 1H), 2.14 (s, 3H), 2.11 (dp, *J* = 9.3, 6.8 Hz, 1H), 1.97 – 1.90 (m, 1H), 1.88 – 1.77 (m, 2H), 1.76 – 1.71 (m, 1H), 1.60 – 1.49 (m, 3H), 1.49 – 1.39 (m, 2H), 0.96 (s, 3H), 0.93 (d, *J* = 0.6 Hz, 3H), 0.93 (s, 3H), 0.77 (d, *J* = 6.8 Hz, 3H). **^{13}C NMR** (151 MHz, $CDCl_3$): δ 214.9, 132.1, 126.0, 50.9, 39.7, 36.9, 34.3, 33.6, 32.6, 30.9, 28.0, 27.3, 25.6, 19.4, 16.4, 15.0. **HRMS**: (GC-EI) (m/z) calculated for $C_{16}H_{26}O$ $[M]^{+}$: 234.197815, found: 234.197810; The enantiomeric excess of the product was determined by GC on a Chiral column: BGB-176/BGB-15 0,25/0,25df G/618, 30 m column length, temp.: 220/117 94 min iso 8/min 240 /350, Gas: 0.70 bar H_2 gas;, t_R^1 = 76.47 min., t_R^2 = 78.04 min., e.r. = 92:8. Preparative HPLC (achiral): t_R = 221.26 min at tenth cycle.

NMR analysis to confirm the structure and relative stereochemistry of the desired product

Atom	J	δ (ppm)	HSQC	COSY	HMBC	NOESY
1 C		25.573	1			
H3		2.140	1		2	15
2 C		214.855			1, 4, 15	
3 C		50.976			4, 5ax, 8ax, 8eq, 15, 16	
4 C		32.623	4		5ax, 5eq, 8ax, 8eq, 15, 16	
H	5.9(5eq), 6.8(16), 9.3(5ax)	2.106	4	5ax, 5eq, 16	2, 3, 5, 6, 8, 15, 16	8ax, 16
5 C		36.889	5ax, 5eq		4, 16	
Hax	17.6(5eq), 9.3(4)	1.522	5	4, 5eq, 8eq	3, 4, 6, 7, 9, 16	15, 16
Heq	5.9(4), 17.6(5ax)	1.937	5	4, 5ax, 8eq	4, 6, 7, 16	16
6 C		126.026			4, 5ax, 5eq, 8ax, 8eq, 9	
7 C		132.109			5ax, 5eq, 8ax, 8eq, 11, 13, 14	
8 C		34.254	8ax, 8eq		4, 15	
Hax		2.278	8	8eq, 9	3, 4, 6, 7, 15	4, 13
Heq		1.736	8	5ax, 5eq, 8ax, 9	3, 4, 6, 7, 15	13, 14, 15
9 C		30.919	9		5ax, 10, 11	
H2		1.808	9	8ax, 8eq, 10	6	
10 C		19.353	10		11	
H2		1.569	10	9, 11	9, 11, 12	14
11 C		39.687	11		10, 13, 14	
H2		1.441	11	10	7, 9, 10, 12, 13, 14	13, 14
12 C		33.630			10, 11, 13, 14	
13 C		28.008	13		11, 14	
H3		0.960	13		7, 11, 12, 14	8ax, 8eq, 11
14 C		27.261	14		11, 13	
H3		0.928	14		7, 11, 12, 13	8eq, 10, 11
15 C		15.037	15		4, 8ax, 8eq	
H3	0.6(?)	0.934	15		2, 3, 4, 8	1, 5ax, 8eq, 16
16 C		16.390	16		4, 5ax, 5eq	
H3	6.8(4)	0.766	16	4	3, 4, 5	4, 5ax, 5eq, 15

Table S3. Peak table for the product **1c**, COSY, HSQC, HMBC, NOESY signals for the assignment of relative stereochemistry (Analysis was carried out using racemic sample and structure depicts only relative stereochemistry).

7.3. Synthesis of the Enantioenriched IsoESuper plus (Arborone)

7.3.1. Achiral Lewis acid catalysis:

In a flame-dried Young tube equipped with a magnetic stirring bar, evacuated and filled with argon, a mixture of isomers containing **6a** (1.0 equiv., 8.53 mmol, 2.0 g) was dissolved in dry 2-methyltetrahydrofuran (4 mL, 40 mmol). The resulting solution was cooled to below 10 °C using an ice–water bath. $SnCl_4$ (0.4 equiv., 3.41 mmol, 0.4 mL) was then added in one portion, resulting in the precipitation of a white solid. The tube was sealed tightly and heated to 80 °C for 4 hours. (Note: the precipitate typically dissolves between 60–65 °C, forming a clear yellow reaction mixture.) The reaction was quenched by the addition of 10 mL of 10% aqueous HCl. The resulting mixture was extracted with MTBE (3 × 40 mL). The combined organic layers were dried over anhydrous Na_2SO_4, and the solvents were removed under reduced pressure. The crude product was then purified as described in the Section 6.3.3.

7.3.2. IDPi-catalyzed variant:

In a pressure vial equipped with a magnetic stirring bar, flame-dried, evacuated, and filled with argon, IDPi-**5c** (2 mol%, 153 mg) was dissolved in $CHCl_3$ (6.8 mL). A mixture of isomers containing **6a** (1.0 equiv., 8.53 mmol, 2.0 g) was then added. The vial headspace was flushed with argon before sealing it tightly, and the reaction mixture was heated to 50 °C for 16 hours. The reaction was quenched by the addition of Et_3N (0.1 equiv., 120 μL). The solvents were removed under reduced pressure, and the crude product was purified as described in Section 6.3.3.

7.3.3. Purification procedure:

The product was purified by column chromatography on silica gel using 3% MTBE in pentane as the eluent, followed by distillation using a Kugelrohr apparatus (p = 0.8 mbar, T = 170 °C) to obtain a mixture of isomers. The isomeric mixture was then separated by preparative HPLC using the method for the separation of Isocyclemone isomers, as described in detail in Section 6.4.2.

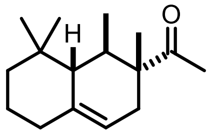

1d

1-((1*R*,2*R*,8a*S*)-1,2,8,8-tetramethyl-1,2,3,5,6,7,8,8a-octahydronaphthalen-2-yl)ethan-1-one IsoESuper plus – arborone (1d)

The product mixture was obtained in 60% yield (8.5 mmol, 1.2 g), with 1d comprising 14% of the mixture when using the achiral Lewis acid procedure. Upon catalysis with IDPi-**5c**, **1d** was obtained in 89% yield (7.6 mmol, 1.79 g), comprising 20% of the mixture. When using a 1:1 mixture of racemic **4a** and **6a**, **1d** was formed with 65:35 e.r., due to kinetic resolution catalyzed by IDPi-**5c**. When the **4a**:**6a** (1:1) mixture was enantioenriched (94:6 e.r.), the resulting **1d** was obtained with 94:6 e.r. In the spectra of the preparative HPLC-purified product, signals corresponding to a residual impurity are still visible. **^{1}HNMR** (501 MHz, $CDCl_3$): 5.43 (dq, *J* = 7.0, 1.7 Hz, 1H), 2.25 – 2.16 (m, 2H), 2.15 (s, 3H), 2.11 – 2.05 (m, 1H), 1.86 (dddd, *J* = 12.8, 8.3, 4.1, 1.8 Hz, 1H), 1.75 – 1.64 (m, 1H), 1.57 – 1.47 (m, 3H), 1.44 – 1.37 (m, 2H), 1.04 (s, 3H), 1.01 (d, *J* = 0.7 Hz, 3H), 0.88 (d, *J* = 6.6 Hz, 3H), 0.84 (s, 3H). **^{13}CNMR** (126 MHz, $CDCl_3$): 215.3, 141.0, 116.7, 53.7, 52.7, 43.4, 37.8, 36.3, 35.5, 33.7, 31.9, 25.8, 24.2, 20.5, 20.2, 16.0. **HRMS**: (GC-EI) (m/z) calculated for $C_{16}H_{26}O$ $[M]^{-}$: 234.197815, found: 234.197890. The enantiomeric excess of the product was determined by GC on a Chiral column: BGB-176/BGB-15 0,25/0,25df G/618, 30 m column length, temp.: 220/117 110 min iso /350, Gas: 0.70 bar H_2 gas; t_R^1 = 83.06 min., t_R^2 = 86.41 min., e.r. = 94:6; Preparative HPLC (achiral): t_R = 179.95 min at third cycle. The spectroscopic data in agreement with those reported.[15]

Additionally, from the isomer mixture the following compounds were isolated:

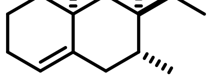

1e

1-((2*R*,3*R*,8a*R*)-2,3,8,8-tetramethyl-1,2,3,4,6,7,8,8a-octahydronaphthalen-2-yl)ethan-1-one – IsoAlpha (1e)

^{1}H NMR (600 MHz, $CDCl_3$): δ 5.39 – 5.34 (m, 1H), 2.13 (s, 3H), 2.07 (dd, *J* = 13.5, 4.2 Hz, 1H), 2.02 – 1.94 (m, 2H), 1.94 – 1.86 (m, 2H), 1.84 – 1.78 (m, 1H), 1.51 (dd, *J* = 12.3, 4.2 Hz, 1H), 1.36 – 1.29 (m, 2H), 1.26 (ddd, *J* = 12.8, 8.3, 5.5 Hz, 1H), 1.09 (s, 3H),

0.89 (s, 3H), 0.77 (s, 3H), 0.70 (d, J = 6.6 Hz, 3H) (ppm). **^{13}C NMR** (151 MHz, $CDCl_3$): δ 214.5, 138.3, 119.2, 52.6, 41.8, 39.7, 37.7, 37.0, 35.9, 31.2, 28.9, 25.4, 23.5, 22.6, 17.2, 13.4 (ppm). **HRMS**: (GC-EI) (m/z) calculated for $C_{16}H_{26}O$ $[M]^{+}$: 234.197815, found: 234.197890. Preparative HPLC (achiral): t_R = 210.25 min at tenth cycle.

NMR data supports the following structure

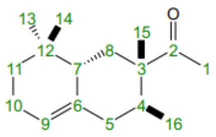

Chem3D model showing relevant NOE correlations

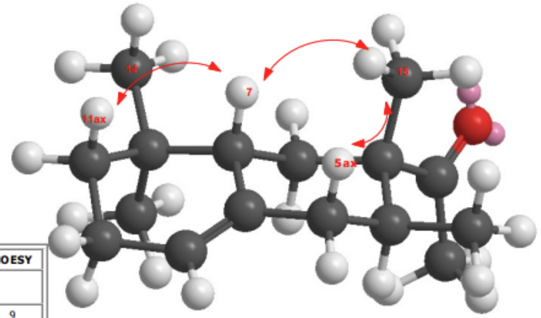

Remarks: ^{1}H NMR shifts of regions with complex multiplets and overlapping signals were extracted from cross peaks in the HSQC or the 1D selective NOESYs (see later pages)

Atom	J	δ (ppm)	HSQC	COSY	HMBC	NOESY
1 C		25.348	1			
H3		2.127	1		2, 3	8ax, 15, 16
2 C		214.457			1, 4, 8ax, 8eq, 15	
3 C		52.568			1, 4, 5eq, 8ax, 8eq, 15, 16	
4 C		37.026	4		5eq, 8eq, 15, 16	
H	6.6(16), 4.2(5eq)	1.984	4	5ax, 5eq, 16	2, 3, 5, 8, 15, 16	16
5 C		39.730	5ax, 5eq		4, 9, 16	
Hax	13.5(5eq)	1.893	5	4, 5eq		15, 16
Heq	13.5(5ax), 4.2(4)	2.072	5	4, 5ax	3, 4, 6, 7, 9, 16	9, 16
6 C		138.246			5eq, 8ax, 8eq, 10"	
7 C		41.839	7		5eq, 8ax, 8eq, 9, 11ax, 11eq, 13, 14	
H	13.2(8ax), 4.2(8eq)	1.811	7	8ax, 8eq		8eq, 11ax, 14, 15
8 C		37.740	8ax, 8eq		4, 15	
Hax	12.3(8eq), 13.2(7)	1.314	8	7, 8eq, 15	2, 3, 6, 7, 15	1, 13
Heq	12.3(8ax), 4.2(7)	1.506	8	7, 8ax	2, 3, 4, 6, 7, 12, 15	7, 13, 14, 15
9 C		119.226	9		5eq, 10", 11ax, 11eq	
H		5.368	9	10', 10"	5, 7, 10, 11	5eq, 10'

Atom	J	δ (ppm)	HSQC	COSY	HMBC	NOESY
10 C		22.559	10', 10"		9, 11ax, 11eq	
H'		1.904	10	9, 11ax, 11eq		9
H"		1.977	10	9, 11ax, 11eq	6, 9, 12	
11 C		35.905	11ax, 11eq		9, 13, 14	
Hax	12.8(11eq), 8.3(?), 5.5(?)	1.255	11	10', 10", 13	7, 9, 10, 12, 14	7, 13, 14
Heq	12.8(11ax)	1.330	11	10', 10"	7, 9, 10, 12, 14	13, 14
12 C		31.204			8eq, 10", 11ax, 11eq, 13, 14	
13 C		23.489	13		14	
H3		0.769	13	11ax, 14	7, 11, 12, 14	8ax, 8eq, 11ax, 11eq
14 C		28.906	14		11ax, 11eq, 13	
H3		0.895	14	13	7, 11, 12, 13	7, 8eq, 11ax, 11eq
15 C		13.441	15		4, 8ax, 8eq	
H3		1.093	15	8ax	2, 3, 4, 8	1, 5ax, 7, 8eq, 16
16 C		17.222	16		4, 5eq	
H3	6.6(4)	0.703	16	4	3, 4, 5	1, 4, 5ax, 5eq, 15

Table S4. Peak table for the product **1e**, COSY, HSQC, HMBC, NOESY signals for the assignment of relative stereochemistry. (Analysis was carried out using racemic sample).

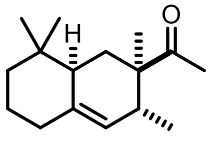

1f

1-((2*R*,3*R*,8a*R*)-2,3,8,8-tetramethyl-1,2,3,5,6,7,8,8a-octahydronaphthalen-2-yl)ethan-1-one – IsoGamma (1f)

^{1}H NMR (600 MHz, $CDCl_3$): δ 5.07 (app. q, *J* = 1.8 Hz, 1H), 2.65 (qtd, *J* = 7.3, 3.5, 1.7 Hz, 1H), 2.18 (ddt, *J* = 13.9, 4.0, 2.2 Hz, 1H), 2.15 (s, 3H), 1.97 – 1.88 (m, 1H), 1.81 (dddt, *J* = 11.3, 6.5, 3.4, 1.7 Hz, 1H), 1.57 – 1.41 (m, 5H), 1.34 (td, *J* = 13.0, 4.5 Hz, 1H), 0.95 (s, 3H), 0.90 (s, 3H), 0.75 (d, *J* = 7.3 Hz, 3H), 0.75 (s, 3H). **^{13}C NMR** (151 MHz, $CDCl_3$): δ 214.9, 136.6, 125.8, 51.3, 44.1, 42.5, 34.8, 34.7, 34.5, 34.4, 29.6, 25.9, 22.5, 20.7, 16.7, 13.9. **HRMS**: (GC-EI) (m/z) calculated for $C_{16}H_{26}O$ $[M]^{\cdot}$: 234.197815, found: 234.197800. Preparative HPLC (achiral): t_R = 216.21 min at tenth cycle.

NMR data supports the following structure

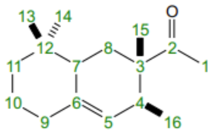

Chem3D model showing relevant NOE correlations

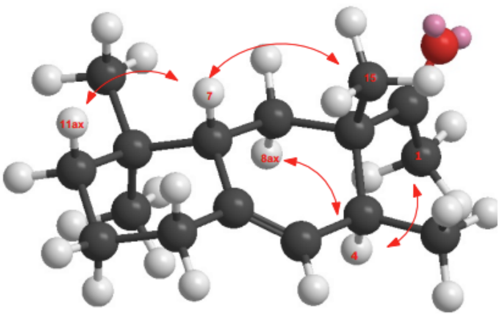

Remarks: ^{1}H NMR shifts of regions with complex multiplets and overlapping signals were extracted from cross peaks in the HSQC or the 1D selective NOESYs (see later pages)

Atom	J	δ (ppm)	HSQC	COSY	HMBC	NOESY
1 C		25.853	1			
H3		2.146	1		2, 3	4, 8ax, 8eq, 15, 16
2 C		214.985			1, 8ax, 8eq, 15	
3 C		51.250			1, 4, 5, 8ax, 8eq, 15, 16	
4 C		34.506	4		5, 8eq, 15, 16	
H	3.5(9ax), 3.5(7), 1.7(5), 7.3(16)	2.652	4	5, 7, 9ax, 16	3, 5, 8, 15, 16	1, 5, 8ax, 16
5 C		125.771	5		4, 7, 9eq, 16	
H	1.7(4)	5.071	5	4, 7, 9ax	3, 4, 11, 16	4, 9eq, 16
6 C		136.559			7, 8eq, 9eq	
7 C		44.074	7		8ax, 8eq, 9eq, 11ax, 13, 14	
H	11.3(8ax), 6.5(8eq), 3.5(4), 1.7(?)	1.811	7	4, 5, 8ax, 8eq	5, 6, 8, 9, 15	11ax, 13, 15
8 C		34.363	8ax, 8eq		4, 7, 15	
Hax	12.7(8eq), 11.3(7)	1.462	8	7, 15	2, 3, 7, 15	1, 4, 13, 14
Heq	12.7(8ax), 6.5(7)	1.532	8	7	2, 3, 4, 6, 7, 15, 16	1, 13, 15
9 C		34.803	9ax, 9eq		7	
Hax	13.9(9eq), 3.5(4)	1.926	9	4, 5, 9eq		
Heq	13.9(9ax)	2.179	9	9ax	5, 6, 7, 10, 11	5

Atom	J	δ (ppm)	HSQC	COSY	HMBC	NOESY
10 C		22.514	10		9eq, 11ax, 11eq	
H2		1.484	10		11	10
11 C		42.510	11ax, 11eq		5, 9eq, 10, 13, 14	
Hax	13.0(11eq), 13.0(10ax), 4.5(10eq)	1.341	11	11eq, 14	7, 10, 12, 13, 14	7, 13
Heq	13.0(11ax)	1.440	11	11ax	10, 13, 14	13, 14
12 C		34.728			11ax, 13, 14	
13 C		29.578	13		11ax, 11eq, 14	
H3		0.898	13		7, 11, 12, 14	7, 8ax, 8eq, 11ax, 11eq, 14
14 C		20.700	14		11ax, 11eq, 13	
H3		0.751	14	11ax	7, 11, 12, 13	8ax, 11eq, 13
15 C		13.914	15		4, 7, 8ax, 8eq	
H3		0.947	15	8ax	2, 3, 4, 8	1, 7, 8eq, 16
16 C		16.718	16		4, 5, 8eq	
H3	7.3(4)	0.751	16	4	3, 4, 5	1, 4, 5, 15

Table S5. Peak table for the product **1f**, COSY, HSQC, HMBC, NOESY signals for the assignment of relative stereochemistry. (Analysis was carried out using racemic sample).

7.4. Preparative HPLC Methods and achiral chromatographic data.

7.4.1. Method for isolation of 6a

The separation was performed using a Shimadzu HPLC system equipped with Agilent RX-Sil column (2x 250 mm × 9.5 mm i.d., 5 µm particle size). The eluent was a mixture of iso-hexane and MTBE (99.6:0.4, v/v) at a flow rate of 4.75 mL/min and a pressure of 6.2 MPa. The sample was applied to the column as a solution in iso-hexane (concentration < 64.2 mg/mL, injection volume: 1.6 mL). The column oven temperature was maintained at 298 K throughout the run. Detection was carried out using UV absorbance at λ = 210 nm with a 0.5 mm optical path length. To achieve complete separation of the isomers, circulation was necessary (Figures S2 and S3).

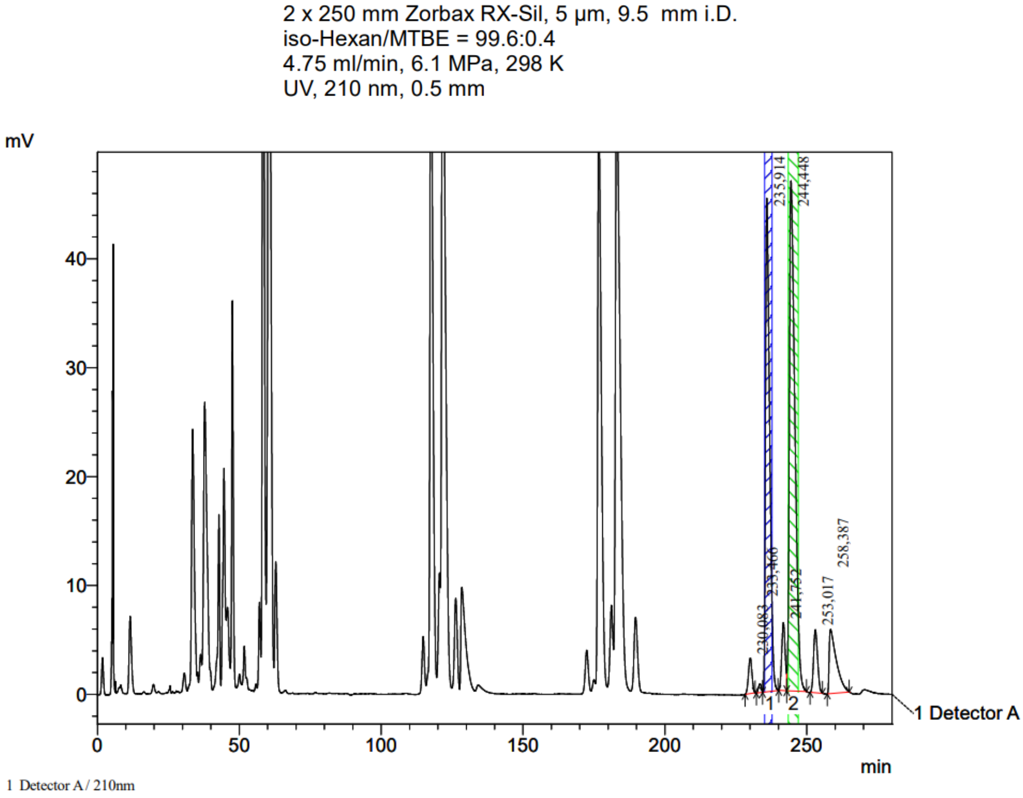

PeakTable

Detector A 210nm

Peak#	Ret. Time	Area %	k'	Name
1	230,08	2,10	0,00	
2	233,47	0,42	0,01	
3	235,91	34,39	0,03	
4	241,75	3,87	0,05	
5	244,45	47,22	0,06	
6	253,02	4,26	0,10	
7	258,39	7,73	0,12	
Total		100,00		

Figure S2. Separation of *rac*-**6a**. (102.8 mg, c = 64.2 mg/ml).

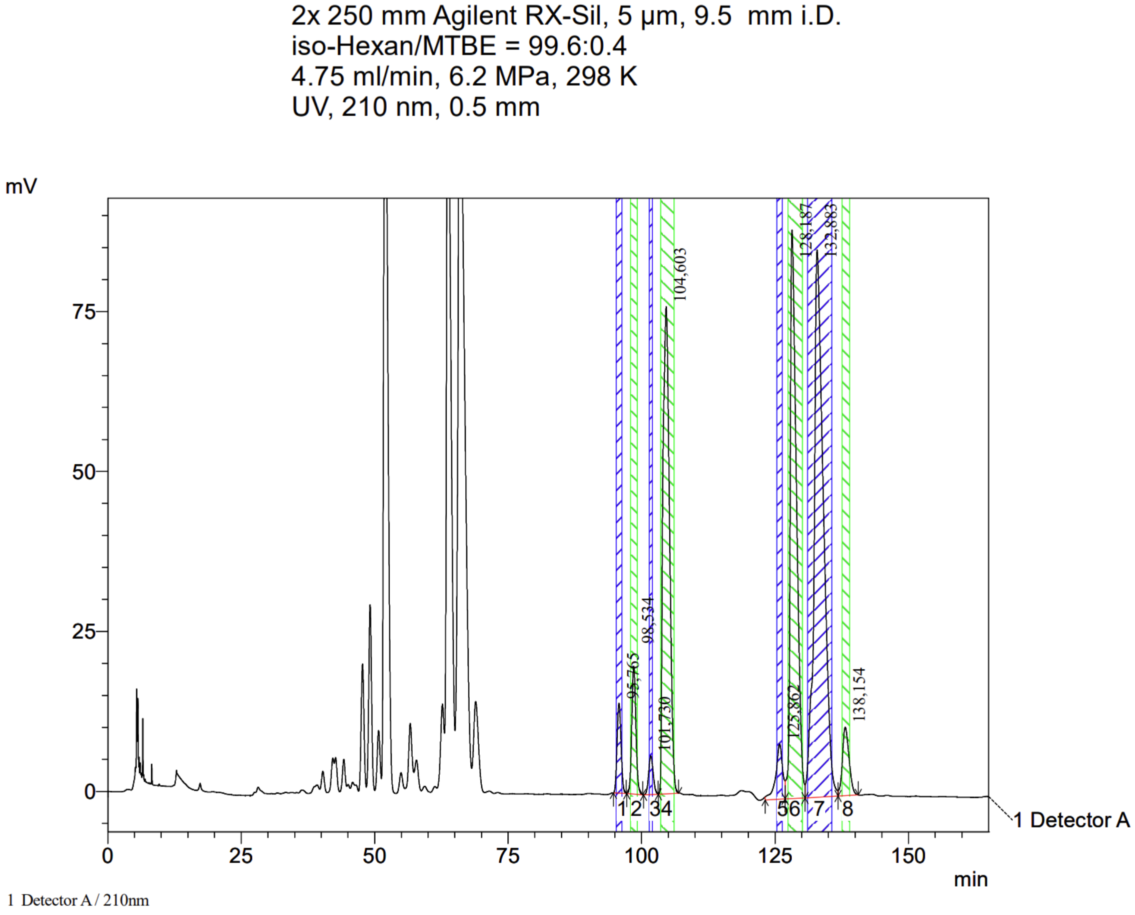

PeakTable

Detector A 210nm

Peak#	Ret. Time	Area %	k'	Name
1	95,77	2,59	0,00	
2	98,53	3,83	0,03	
3	101,73	1,28	0,06	
4	104,60	22,96	0,09	
5	125,86	2,75	0,31	
6	128,19	25,82	0,34	
7	132,88	37,46	0,39	
8	138,15	3,32	0,44	
Total		100,00		

Figure S3. Separation of enantiopure **6a**. (27.6 mg, c = 46 mg/ml).

7.4.2. Method for separation of Isocyclemone isomers 1c-f

The separation was performed using a Shimadzu HPLC system equipped with Agilent RX-Sil column (2x 250 mm × 9.5 mm i.d., 5 µm particle size). The eluent was a mixture of iso-hexane and MTBE (99.6:0.4, v/v), delivered at a flow rate of 4.75 mL/min and a pressure of 6.2 MPa. The sample mixture was applied to the column as a solution in iso-hexane. The column oven temperature was maintained at 298 K throughout the run, and

UV detection was used (λ = 210 nm, optical path length = 0.5 mm). To achieve separation of the isomers, circulation was necessary, as shown in Figures S4 and S5.

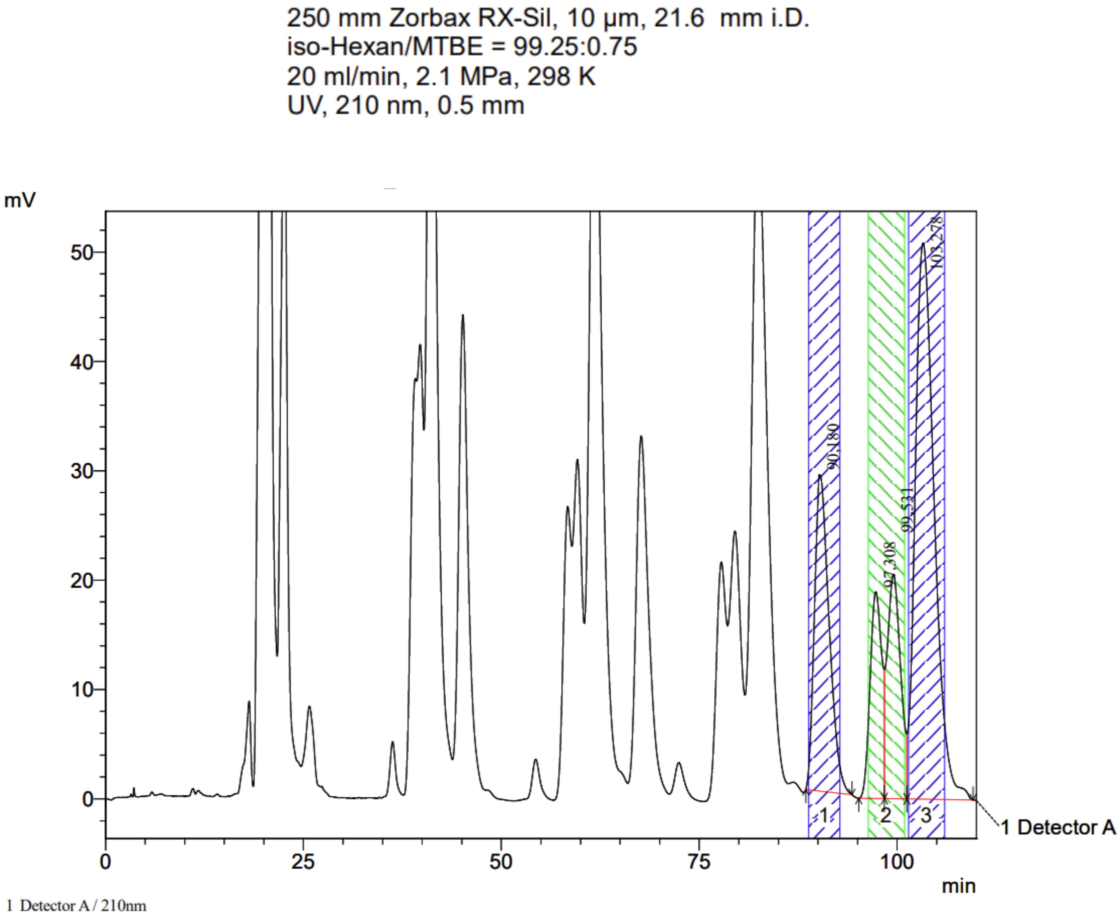

PeakTable

Detector A 210nm

Peak#	Ret. Time	Area %	k'	Name
1	90,18	22,41	0,00	
2	97,31	11,63	0,08	
3	99,53	14,56	0,10	
4	103,28	51,40	0,15	
Total		100,00		

Figure S4. Separation of *rac*-**1c-f**. (257.4 mg, c = 122.6 mg/ml)

2x 250 mm Agilent RX-Sil, 5 µm, 9.5 mm i.D.
iso-Hexan/MTBE = 99.6:0.4
4.75 ml/min, 6.2 MPa, 298 K
UV, 210 nm, 0.5 mm

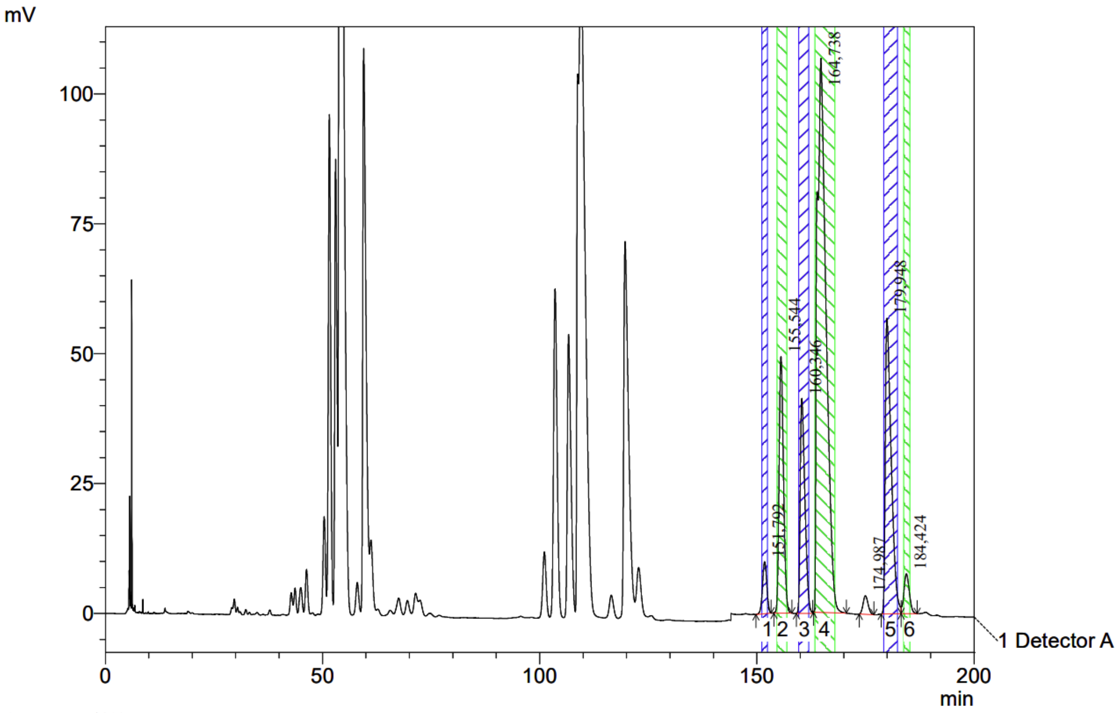

1 Detector A / 210nm

PeakTable

Detector A 210nm

Peak#	Ret. Time	Area %	k'	Name
1	151,79	2,40	0,00	
2	155,54	12,11	0,02	
3	160,35	10,80	0,06	
4	164,74	52,59	0,09	
5	174,99	1,00	0,15	
6	179,95	18,76	0,19	
7	184,42	2,33	0,21	
Total		100,00		

Figure S5. Separation of enantiopure **1c-f**. (95 mg, c = 95 mg/ml)

7.4.3. Chromatographic data for separated isomers (achiral separations)

100 mm Zorbax RX-Sil, 5 µm, 4.6 mm
iso-Hexan/MTBE = 99.5:05
1.0 ml/min, 7.6 MPa, RT
DAD, 210 nm

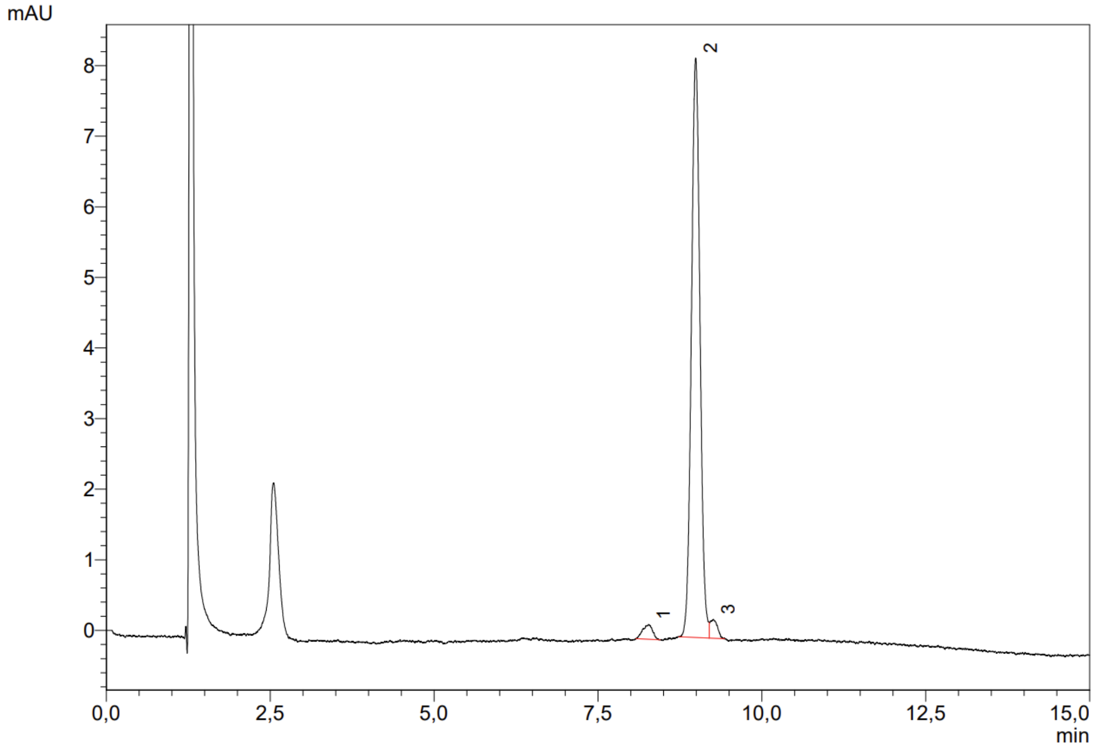

1 210nm,4nm

PDA Ch1 210nm

Peak #	Ret. Time	Area %	Name
1	8,28	2,77	
2	8,99	94,63	
3	9,26	2,60	
Total		100,00	

Figure S6. Isolated compound **6a**.

100 mm Zorbax RX-Sil, 1.8 µm, 4.6 mm
iso-Hexan/MTBE = 99.5:05
1.0 ml/min, 7.6 MPa, RT
DAD, 210 nm

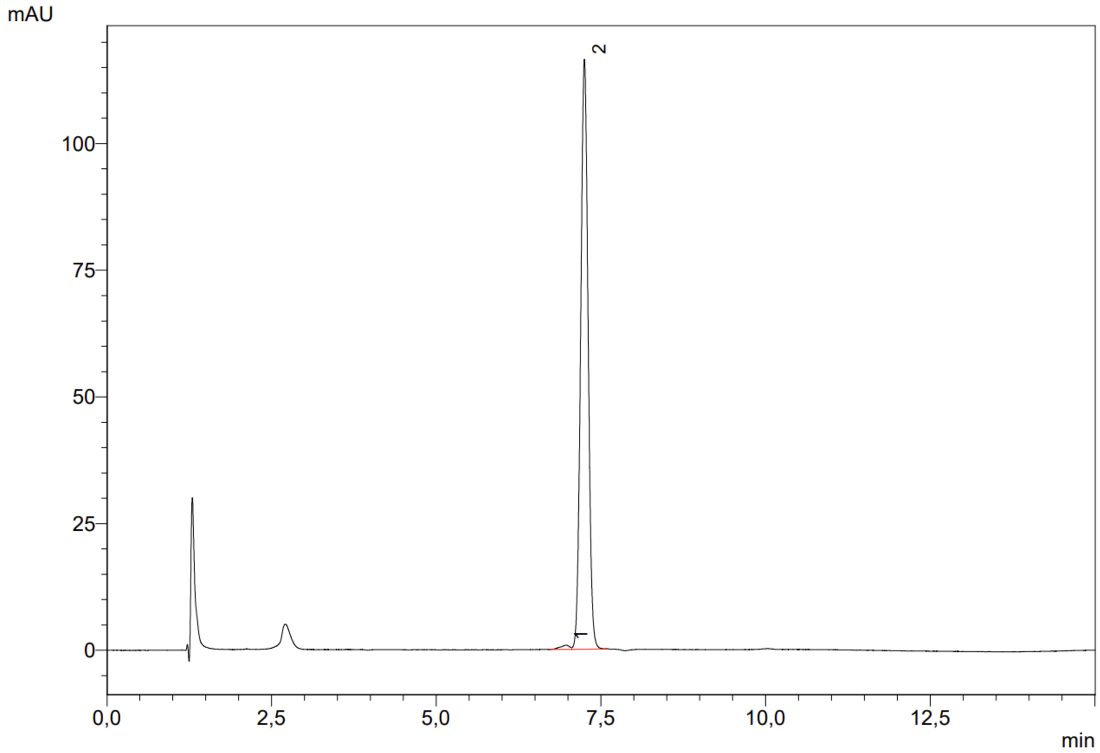

1 210nm,4nm

PDA Ch1 210nm

Peak #	Ret. Time	Area %	Name
1	6,97	0,90	
2	7,25	99,10	
Total		100,00	

Figure S7. Isolated compound **1c**.

100 mm Zorbax RX-Sil, 1.8 µm, 4.6 mm i.D.
iso-Hexan/MTBE = 99.6:0.4
1.0 ml/min, 8.6 MPa, 298 K
UV, 220 nm

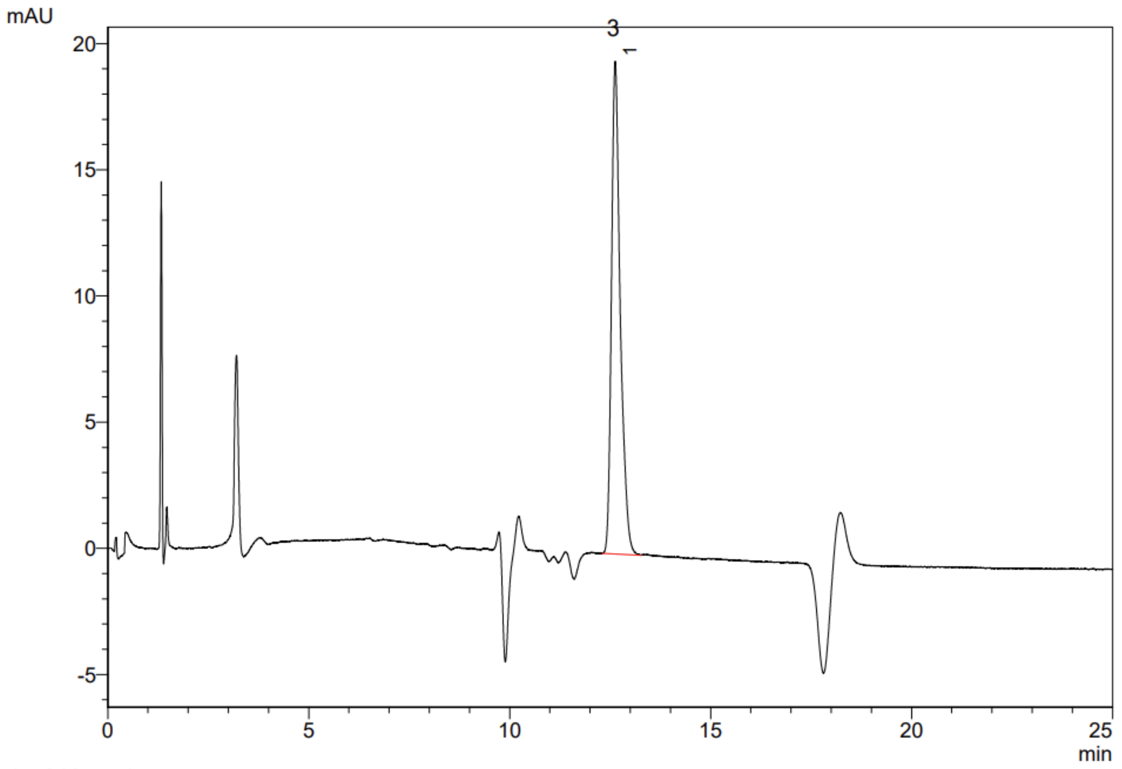

1 210nm,4nm

PDA Ch1 210nm

Peak #	Ret. Time	Area %	Name
1	12,63	100,00	
Total		100,00	

Figure S8. Isolated compound **1d**.

100 mm Zorbax RX-Sil, 1.8 µm, 4.6 mm
iso-Hexan/MTBE = 99.5:05
1.0 ml/min, 7.6 MPa, RT
DAD, 210 nm

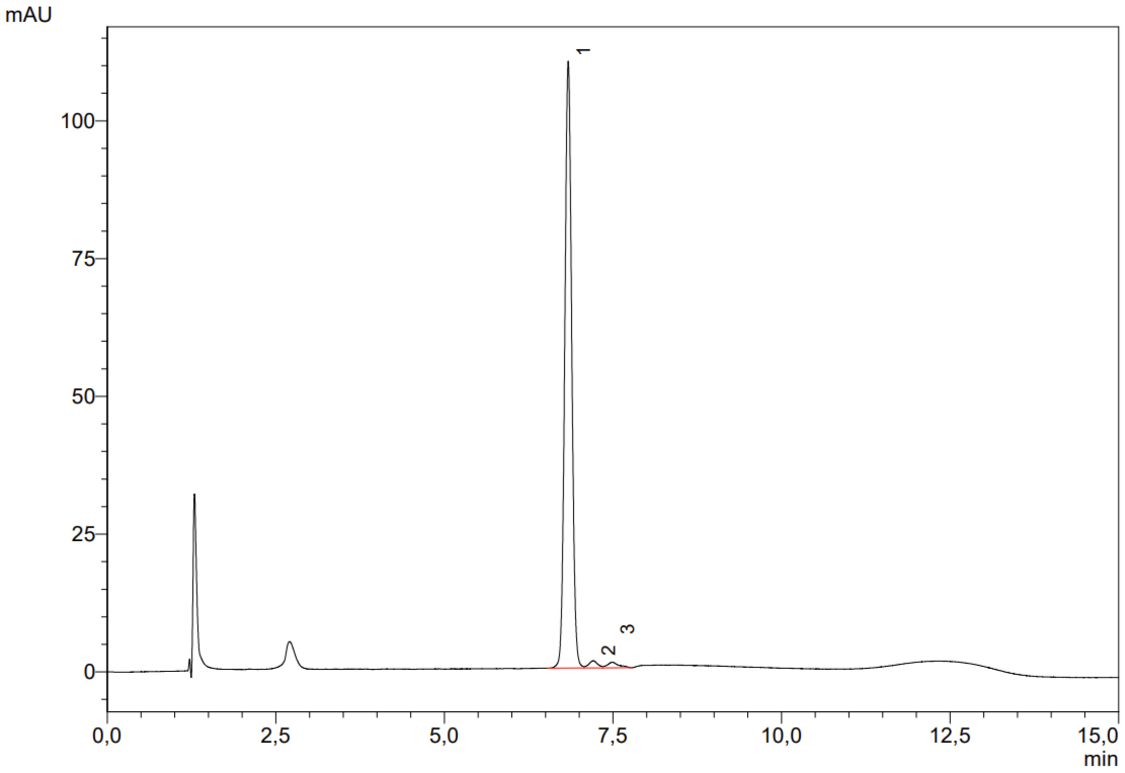

1 210nm,4nm

PDA Ch1 210nm

Peak #	Ret. Time	Area %	Name
1	6,84	97,07	
2	7,21	1,47	
3	7,49	1,46	
Total		100,00	

Figure S9. Isolated compound **1e**.

100 mm Zorbax RX-Sil, 1.8 µm, 4.6 mm
iso-Hexan/MTBE = 99.5:05
1.0 ml/min, 7.6 MPa, RT
DAD, 210 nm

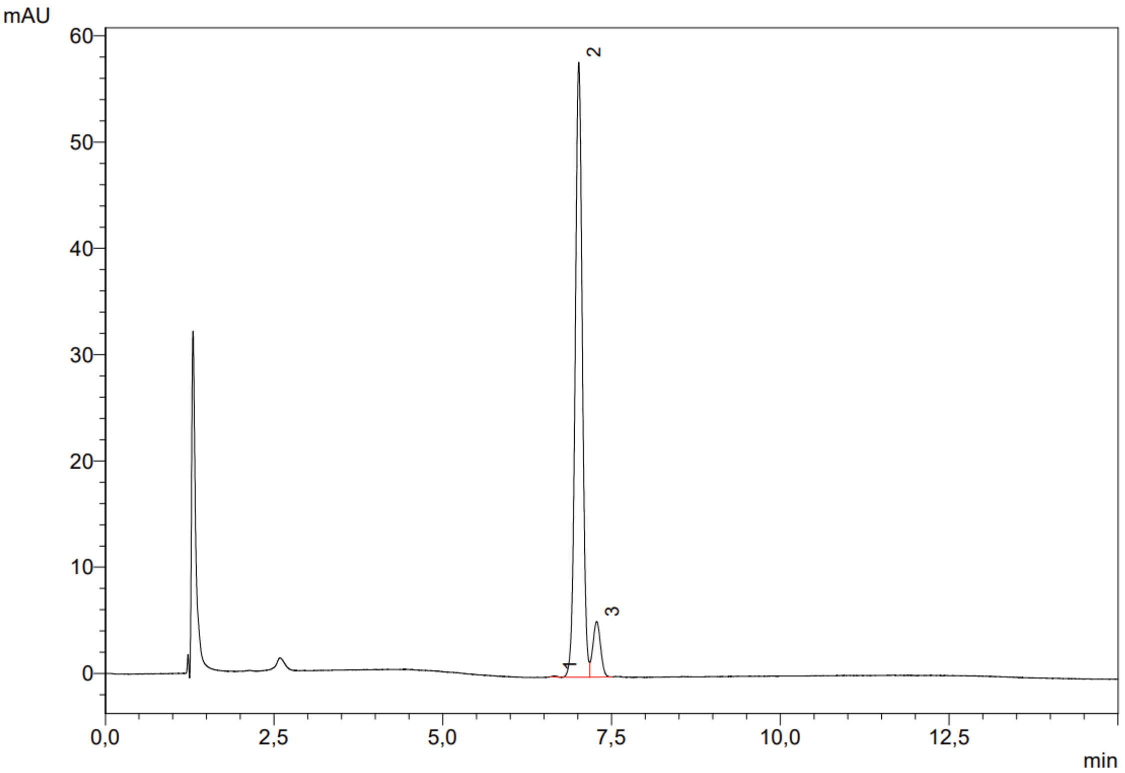

1 210nm,4nm

PDA Ch1 210nm

Peak #	Ret. Time	Area %	Name
1	6,65	0,10	
2	7,01	91,05	
3	7,28	8,85	
Total		100,00	

Figure S10. Isolated compound **1f**.

8. Synthesis of Damascones

8.1. Synthesis of α-Damascone Precursors

8.1.1. The isomerization of the Endocyclic Double Bond[16]

A pressure vial equipped with a magnetic stirring bar was flame-dried, evacuated, and filled with argon. The vial was then charged with $RhCl_3{\cdot}3H_2O$ (12.5 mg, 0.05 equiv., 5 mol%) dissolved in anhydrous ethanol (1 mL). Compound **4w** (200 mg, 1.2 mmol) was then added. The vial was sealed tightly, placed on a heating plate, and stirred at 80 °C for 2 hours. The crude reaction mixture was directly purified by column chromatography on silica gel using 2% Et_2O in pentane as the eluent, affording product **6w** as a colorless oil (184.2 mg, 1.1 mmol, 92% yield, 95:5 e.r.).

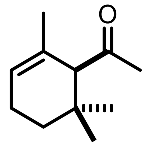

6w

(*S*)-1-(2,6,6-trimethylcyclohex-2-en-1-yl)ethan-1-one (6w)
^{1}H NMR (501 MHz, $CDCl_3$): δ 5.67 – 5.58 (m, 1H), 2.73 (s, 1H), 2.18 (s, 3H), 2.16 – 1.91 (m, 4H), 1.69 (ddd, J = 13.3, 10.5, 6.5 Hz, 1H), 1.61 – 1.58 (m, 3H), 1.18 (ddt, J = 13.5, 5.9, 2.0 Hz, 1H), 0.93 (d, J = 7.3 Hz, 6H). The impurities visible on ^{1}H NMR spectrum were not removed and did not have any impact on a final product formation. **^{13}C NMR** (126 MHz, $CDCl_3$): δ 212.4, 130.7, 123.9, 64.8, 32.6, 32.5, 31.3, 28.3, 28.3, 23.7, 23.0. The enantiomeric excess of the product was determined by GC on a Chiral column: BGB-176/BGB-30 0,25/0,25df, temp.: 220/80 1/min 220, 5 min iso/350, Gas: 0.50 bar He gas; t_R^1 = 24.54 min., t_R^2 = 25.01 min., e.r. = 95:5; The spectral data remained in agreement with the literature.[16]

8.1.2. Epimerisation of 4w to *trans*-4w[17]

A pressure vial equipped with a magnetic stirring bar was flame-dried, evacuated, and filled with argon. The vial was then charged with 4% (m/m) KOH solution in anhydrous ethanol (0.2 mL) and starting material **4w** (0.6 mmol, 100 mg). The vial headspace was flushed with argon, sealed tightly, and heated under reflux overnight. The crude reaction mixture was directly purified by column chromatography on silica gel using 3% Et_2O in pentane as the eluent to afford pure ***trans*-4w** (87% yield, 0.523 mmol, 87 mg). As the compound is volatile, the solvents were evaporated with great care. The spectroscopic data were in agreement with literature values.

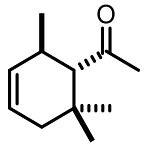

***trans*-4w**

1-((1*S*,2*R*)-2,6,6-trimethylcyclohex-3-en-1-yl)ethan-1-one (*trans*-4w)

^{1}H NMR (501 MHz, $CDCl_3$): δ 5.53 (ddt, *J* = 9.9, 4.9, 2.2 Hz, 1H), 5.45 (ddt, *J* = 9.9, 3.2, 1.7 Hz, 1H), 2.49 (dtt, *J* = 9.0, 4.4, 2.2 Hz, 1H), 2.30 (d, *J* = 10.7 Hz, 1H), 2.19 (s, 3H), 1.97 (ddt, *J* = 17.7, 4.7, 2.4 Hz, 1H), 1.70 (ddt, *J* = 17.5, 5.3, 1.7 Hz, 1H), 0.96 (d, *J* = 27.1 Hz, 6H), 0.89 (d, *J* = 6.9 Hz, 3H).; **^{13}C NMR** (126 MHz, $CDCl_3$): δ 213.5, 131.9, 124.3, 63.6, 41.9, 34.9, 33.0, 31.7, 29.9, 20.8, 20.0; The enantiomeric excess of the product was determined by GC on a Chiral column: Hydrodex-gamma DiMOM 0,25/0,25df, temp.: 220/70 min iso/350, Gas: 0.50 bar H_2 gas; t_R^1 = 32.58 min., t_R^2 = 33.93 min., e.r. = 95:5;

8.2. Synthesis of Damascones via Aldolization-crotonization Approach[18]

A flame-dried Schlenk tube (equipped with a magnetic stirring bar and septum) was filled with argon and charged with freshly distilled *N*-methylaniline (0.53 mmol, 1.1 equiv., 57.5 µL) and toluene (0.5 mL, dried over molecular sieves). The mixture was cooled in a salt–ice bath to below 0 °C. Then, a solution of MeMgBr in Et_2O (0.53 mmol, 1.1 equiv., 3 M, 177 µL) was added dropwise, and the reaction was stirred at room temperature for 10 minutes. The mixture was cooled again to below 0 °C, and a solution of the precursor (**4w**, ***trans*-4w**, or **6w**) in Et_2O (0.48 mmol, 0.48 M, 1 mL) was added via the septum, dropwise but rapidly. The reaction was stirred for 30 minutes, keeping the temperature below 20 °C. Subsequently, the reaction mixture was cooled once more to below 0 °C, and a solution of acetaldehyde in Et_2O (0.96 mmol, 2.0 equiv., 0.24 M, 4 mL) was added dropwise. The reaction was stirred in the ice bath for 1 hour, then allowed to warm to room temperature and stirred for an additional 30 minutes. The reaction mixture was quenched by pouring it into 20 mL of 2.5% aqueous HCl (m/m). The phases were separated, and the aqueous layer was extracted with Et_2O (3 × 10 mL). The combined organic layers were dried over anhydrous Na_2SO_4, filtered, and concentrated under reduced pressure. Note: Toluene was not removed due to the high volatility of the products. The crude product was redissolved in 2 mL of toluene in a round-bottom flask equipped with a magnetic stirring bar under an argon atmosphere.

Dehydration of the aldol product was carried out by adding *p*-toluenesulfonic acid monohydrate (0.048 mmol, 0.1 equiv., 8.3 mg) as a catalyst and sodium sulfate (0.48 mmol, 1.0 equiv., 68 mg) as a drying agent. The reaction mixture was heated to 85 °C and monitored by TLC (5% MTBE in pentane, *p*-anisaldehyde stain). Damascone products typically appeared as strong purple-blue or violet-red spots, confirming full conversion of the aldol intermediate into the desired damascone.

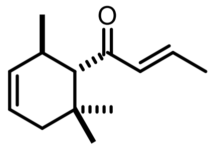

***trans*-1b**

(1*S*,2*R*)-*trans*-δ-Damascone (*trans*-1b)

Product was obtained from ***trans*-4w** as colorless volatile oil (67% yield, 95:5 e.r.). **^{1}H NMR** (600 MHz, $CDCl_3$): δ 6.82 (dq, *J* = 15.6, 6.9 Hz, 1H), 6.21 (dq, *J* = 15.6, 1.7 Hz, 1H), 5.54 (ddt, *J* = 9.8, 5.2, 2.2 Hz, 1H), 5.50 – 5.45 (m, 1H), 2.57 (m, 1H), 2.48 (d, *J* = 10.6 Hz, 1H), 2.03 – 1.96 (m, 1H), 1.89 (dd, *J* = 6.9, 1.7 Hz, 3H), 1.71 (ddt, *J* = 17.5, 5.3, 1.7 Hz, 1H), 0.94 (s, 3H), 0.90 (s, 3H), 0.84 (d, *J* = 6.9 Hz, 3H). **^{13}C NMR** (151 MHz, $CDCl_3$): δ 204.0, 141.8, 134.8, 132.2, 124.2, 60.2, 42.0, 33.4, 31.5, 30.1, 21.0, 20.1, 18.4. **HRMS**: (GC-ESI) (m/z) calculated for $C_{13}H_{20}O$ $[M]^+$: 192.150865, found: 192.150940; The enantiomeric excess of the product was determined by GC on a Chiral column: BGB-176/BGB-30 0,25/0,25df, temp.: 220/80 1/min 220, 5 min iso/350, Gas: 0.50 bar He gas; t_R^1 = 42.73 min., t_R^2 = 43.49 min., e.r. = 95:5; **$[\alpha]_D^{25}$** = - 61.818 (*c* = 0.35 M, CH_2Cl_2). Analogously the opposite enantiomer was synthesized to give pure **(*S*,*R*)-*trans*-δ-damascone** with 81% yield, >20:1 d.r. and 5:95 e.r.

NMR data supports the following structure with the shown relative stereochemistry

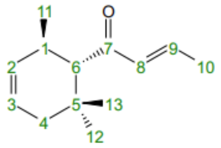

Remarks: Due to long-range (homo)-allylic couplings, many multiplets of the compound are rather complex and not all the *J*-values were determined or assignable.
The strong *J*-coupling between H8 and H9 (15.6 Hz) shows *trans*-configuration.
The large coupling between H1 and H6 (10.6 Hz) also shows the *trans* configuration at position 1 and 6. The following Chem3D model shows relevant NOEs that further support this:

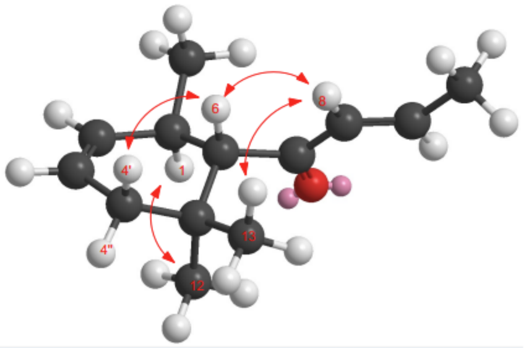

Atom	δ (ppm)	J	COSY	HSQC	HMBC	NOESY
1 C	31.510			1	2, 3, 6, 11	
H	2.572	10.60(6)	4', 6, 11	1		2, 11, 12
2 C	132.240			2	4", 11	
H	5.477	9.80(3)	2, 4'	2	1, 4, 6	1, 11
3 C	124.156			3	4"	
H	5.539	9.80(2), 5.20(4"), 2.20(t, n.a.)	4"	3	1, 4	4', 4"
4 C	42.044			4', 4"	2, 3, 6, 12, 13	
H'	1.989		1, 2, 4", 12	4		3, 6, 13
H"	1.711	5.20(3), 1.70(t, n.a.)	3, 4'	4	2, 3, 5, 6, 12	3, 12, 13
5 C	33.351				4", 6, 12, 13	
6 C	60.241			6	2, 4", 11, 12, 13	
H	2.477	10.60(1)	1	6	1, 4, 5, 7, 11, 12, 13	4', 8, 9, 11, 13
7 C	204.015				6, 8, 9	
8 C	134.814			8	10	
H	6.211	15.60(9), 1.70(10)	9, 10	8	7, 10	6, 13
9 C	141.821			9	10	
H	6.824	6.90(10), 15.60(8)	8, 10	9	7, 10	6
10 C	18.377			10	8, 9	
H3	1.888	6.90(9), 1.70(8)	8, 9	10	8, 9	
11 C	20.045			11	6	
H3	0.843		1	11	1, 2, 6	1, 2, 6
12 C	21.029			12	4", 6, 13	
H3	0.936		4'	12	4, 5, 6, 13	1, 4"
13 C	30.112			13	6, 12	
H3	0.905			13	4, 5, 6, 12	4', 4", 6, 8

Table S6. Peak table for the product ***trans*-1b**, COSY, HSQC, HMBC, NOESY signals for the assignment of relative stereochemistry. (Analysis was carried out using enantiopure sample)

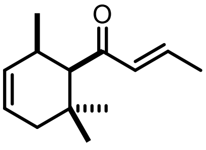

***cis*-1b**

(1*R*,2*R*)-*cis*-δ-Damascone (*cis*-1b)

Product was obtained from **4w** as colorless volatile oil 89% yield (82 mg, 95:5 e.r.). ^{1}**H NMR** (501 MHz, $CDCl_3$): δ 6.78 (dq, *J* = 15.5, 6.9 Hz, 1H), 6.19 (dq, *J* = 15.5, 1.7 Hz, 1H), 5.70 (ddt, *J* = 10.2, 5.2, 2.6 Hz, 1H), 5.49 – 5.42 (m, 1H), 2.74 (d, *J* = 6.4 Hz, 1H), 2.61 – 2.48 (m, 1H), 2.24 – 2.13 (m, 1H), 1.87 (dd, *J* = 6.9, 1.7 Hz, 3H), 1.74 – 1.63 (m, 1H), 0.99 (s, 3H), 0.93 (d, *J* = 7.6 Hz, 3H), 0.89 (s, 3H). ^{13}**C NMR** (126 MHz, $CDCl_3$): δ

203.3, 141.5, 134.6, 129.4, 125.9, 57.5, 36.2, 32.5, 30.4, 29.2, 29.0, 18.3, 17.8. **HRMS**: (GC-ESI) (m/z) calculated for $C_{13}H_{20}O$ $[M]^+$: 192.150865, found: 192.151180; The enantiomeric excess of the product was determined by GC on a Chiral column: BGB-176/BGB-30 0,25/0,25df, temp.: 220/80 1/min 220, 5 min iso/350, Gas: 0.50 bar He gas; t_R^1 = 44.94 min., t_R^2 = 45.86 min., e.r. = 95:5; $[\alpha]_D^{25}$ = -186.6 (*c* = 0.021 M, $CHCl_3$).

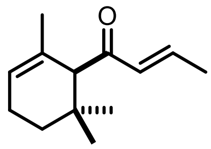

1a

(*S*)-α-Damascone (1a):

Product was obtained from **6w** as colorless volatile oil with 79% yield over all steps (73.3 mg, 95:5 e.r.). **^{1}H NMR** (501 MHz, $CDCl_3$) δ : 6.86 (dq, *J* = 15.5, 6.9 Hz, 1H), 6.29 (dq, *J* = 15.5, 1.7 Hz, 1H), 5.59 (td, *J* = 3.3, 1.7 Hz, 1H), 2.87 (s, 1H), 2.16 – 2.01 (m, 1H), 1.87 (dd, *J* = 6.8, 1.7 Hz, 3H), 1.68 (ddd, *J* = 13.3, 10.3, 6.4 Hz, 1H), 1.54 (q, J = 1.9 Hz, 3H), 1.15 (ddd, *J* = 13.3, 6.3, 2.6 Hz, 1H), 0.93 (s, 3H), 0.83 (s, 3H). **^{13}C NMR** (126 MHz, $CDCl_3$) δ 202.1, 142.1, 132.1, 130.4, 123.1, 61.2, 32.3, 31.2, 27.9, 27.7, 23.2, 22.6, 18.1. **HRMS**: (GC-ESI) (m/z) calculated for $C_{13}H_{20}O$ $[M]^+$: 192.150865, found: 192.150940; The enantiomeric excess of the product was determined by GC on a Chiral column: BGB-176/BGB-30 0,25/0,25df, temp.: 220/80 1/min 220, 5 min iso/350, Gas: 0.50 bar He gas; t_R^1 = 45.14 min., t_R^2 = 45.60 min., e.r. = 95:5; $[\alpha]_D^{25}$ = -245.85 (*c* = 0.0166 M, $CHCl_3$).

9. Synthesis of IDPi catalysts

9.1. Sulfonamide Synthesis

All sulfonamides except **7e** were obtained from commercial sources.

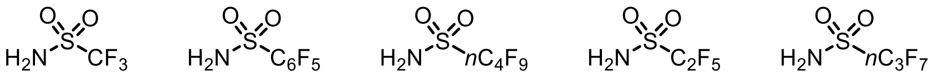

7a **7b** **7c** **7d** **7e**

The sulfonamide **7e** was synthesized as described below.

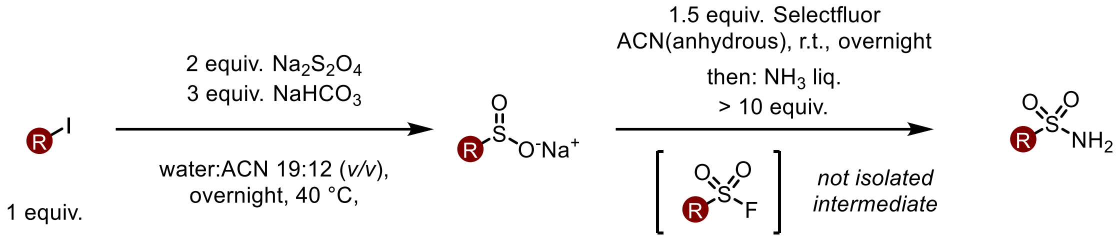

Step 1: Sulfinate salt synthesis:[19]

A Schlenk tube equipped with a magnetic stirring bar was evacuated and filled with argon. The reaction vessel was then charged with *n*-perfluoropropyl iodide (18.8 mmol, 4.1 g, 2 mL), water (19 mL), and acetonitrile (12 mL). The reaction mixture was cooled to −15 °C, and a solid mixture of sodium bicarbonate (3.0 equiv., 41 mmol, 3.49 g) and sodium dithionite (82% purity, 2.0 equiv., 27.7 mmol, 5.8 g) was added slowly. The Schlenk tube was then sealed carefully and protected due to potential pressure buildup. The reaction mixture was stirred at 40 °C overnight. After completion, the mixture was diluted with 50 mL of water and extracted with ethyl acetate (4 × 100 mL). The combined organic layers were washed with brine, dried over anhydrous Na_2SO_4, and the solvents were removed under reduced pressure. The product (sulfinate salt) was obtained as a yellowish solid in 98.3% yield (4.36 g, 17.02 mmol). The sulfinate salt was analyzed by ^{19}F NMR and compared with batches from previous projects.

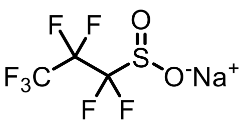

^{19}F NMR (471 MHz, CD_3CN): δ -82.11, -82.13, -82.15, -127.67, -132.82, -132.84, -132.86, -132.88.

Step 2: Sulfinate salt oxidative fluorination and ammonolysis of sulfonyl fluoride.

A Young tube equipped with a magnetic stirring bar was flame-dried thoroughly and filled with dry argon. The sulfinate salt (6.9 mmol, 1.77 g), previously dried for 4 days at room temperature under high vacuum (p = 0.01 mbar), was then added and dissolved in anhydrous acetonitrile (10 mL, spectroscopy grade, water content < 5 ppm). The resulting yellow solution was cooled to near 0 °C using an ice–water bath. Dry Selectfluor (2.0 equiv., 13.8 mmol, 4.89 g), also dried for 4 days at room temperature under high vacuum (p = 0.01 mbar), was added slowly to the reaction mixture. The tube was sealed tightly, and the reaction was stirred at room temperature (20–25 °C) for 16 hours. Upon completion, the reaction mixture turned milky white. The crude reaction mixture was analyzed by ^{19}F NMR (Figure S11), confirming complete conversion of the sulfinate salt into the corresponding sulfonyl fluoride. Due to the sensitivity of sulfonyl fluorides, the crude product was used directly in the next step.

Note: Strictly anhydrous conditions are essential for the success of this reaction.

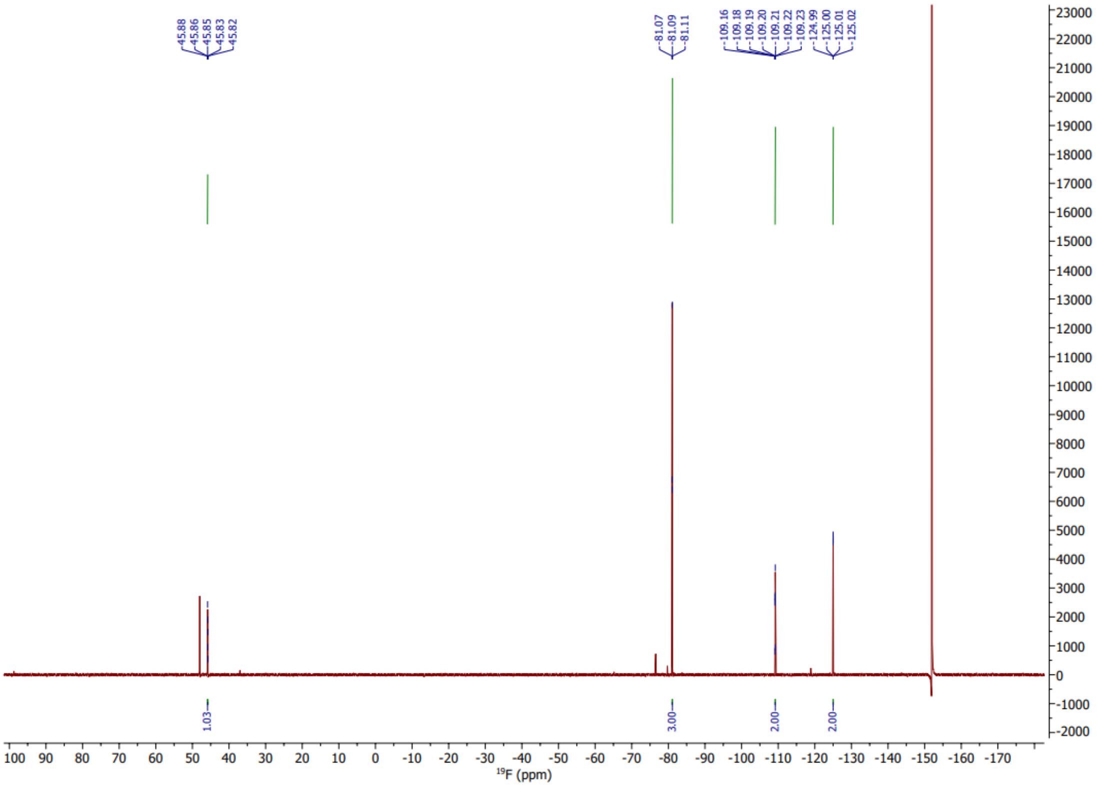

Figure S11. The ^{19}F NMR spectrum of the crude sulfonyl fluoride – precursor to **7e**.

In a flame-dried round-bottom flask (equipped with a magnetic stirring bar) and filled with argon, ammonia (10 mL) was condensed using a dry ice–acetone bath. While stirring, the crude mixture from the previous step was added. The cooling bath was then removed, and the vessel was opened to allow the ammonia to evaporate completely. After full evaporation, the residue was diluted with 10 mL of water and 20 mL of 6 M aqueous HCl (adjusting the final pH to below 1–2). The mixture was then extracted with DCM (5 × 50 mL). The combined organic layers were dried over anhydrous Na_2SO_4, filtered, and concentrated under reduced pressure to yield the sulfonamide as white to creamy crystals (52.5% yield, 3.26 mmol, 812.3 mg).

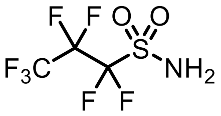

7e

^{1}H NMR (501 MHz, CD_3CN): δ 6.87 (br s, 2H). **^{19}F NMR** (565 MHz, DMSO): δ -80.38 (t, J = 9.2 Hz, 3F), -114.70 (dd, J = 9.4, 2.7 Hz, 2F), -124.46 (d, J = 2.4 Hz, 2F). **^{13}C**

NMR (151 MHz, DMSO): δ 117.0 (qt, J = 289, 35 Hz, 1C), 112.6 (tt, J = 278, 33Hz, 1C), 108.3 (tq, J = 257, 33 Hz, 1C). **^{13}C{^{19}F} NMR** (151 MHz, DMSO): δ 117.1, 112.6, 108.4. **HRMS**: (ESI-) (*m*/*z*) calculated for $C_3HO_2NF_7S$ $[M]^-$: 247.962176, found: 247.962270.

9.2. Phosphazine Synthesis

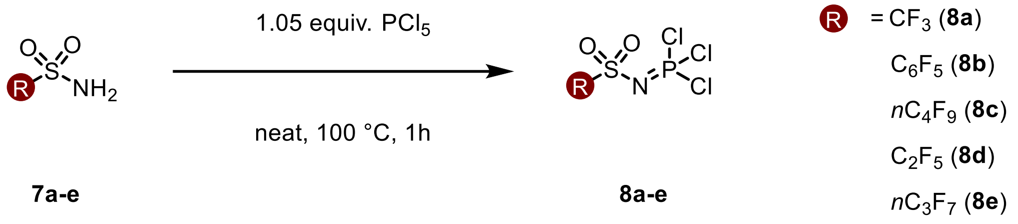

Phosphazine reagents were synthesized from sulfonamides **7a-e** according to the literature procedures.[20] Analytical data of freshly synthesized phosphazines **8a-d** were compared with batches synthesized in our laboratory for other projects. Herein only new phosphazine is described and characterized.

Compound **8e** was synthesized according to a literature method in the solid state. A Schlenk tube equipped with a magnetic stirring bar was flame-dried, evacuated, filled with argon, and connected via a distillation bridge to a two-neck flask containing anhydrous sodium hydroxide (18 mmol, 10 equiv.) and 5 Å molecular sieves (1 g). The Schlenk tube was then charged with PCl_5 (1.8 mmol, 375 mg, 1.05 equiv.) and the sulfonamide (1.7 mmol, 1.0 equiv., 423 mg) under a flow of argon. The system was sealed, and the Schlenk tube was heated to 100 °C and stirred for 2 hours. After the reaction, a vacuum pump was connected to the flask containing the base, and the system pressure was reduced to 40 mbar to remove residual PCl_5 by sublimation. Under a continuous flow of argon, the base-containing flask was then replaced with a flame-dried Schlenk tube filled with argon. The outlet of this new Schlenk tube was connected to the vacuum pump. The crude yellowish oily product was purified by distillation under reduced pressure (T = 150 °C, p = 1 mbar), with the receiving Schlenk tube cooled to −78 °C using a dry ice–acetone bath, yielding compound **8e** as a colorless oil (94% yield).

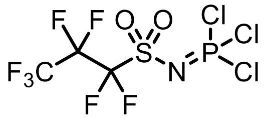

8e

^{19}F NMR: (471 MHz, $CDCl_3$) δ -80.65 (t, *J* = 9.2 Hz, 3F), -112.80 (q, *J* = 9.5 Hz, 2F), -124.36 (d, *J* = 3.5 Hz, 2F). **^{31}P NMR:** (203 MHz, $CDCl_3$) δ 15.29. The right assumption of the phosphazine structure was confirmed by successful use of it in catalyst's synthesis. As described in Section 8.4. the reaction with **8e** yielded the desired IDPi product which was fully characterized by NMR and HRMS.

9.3. General Procedure for BINOL synthesis 10a-f

BINOLS were synthesized according to the modified literature procedure.[21]

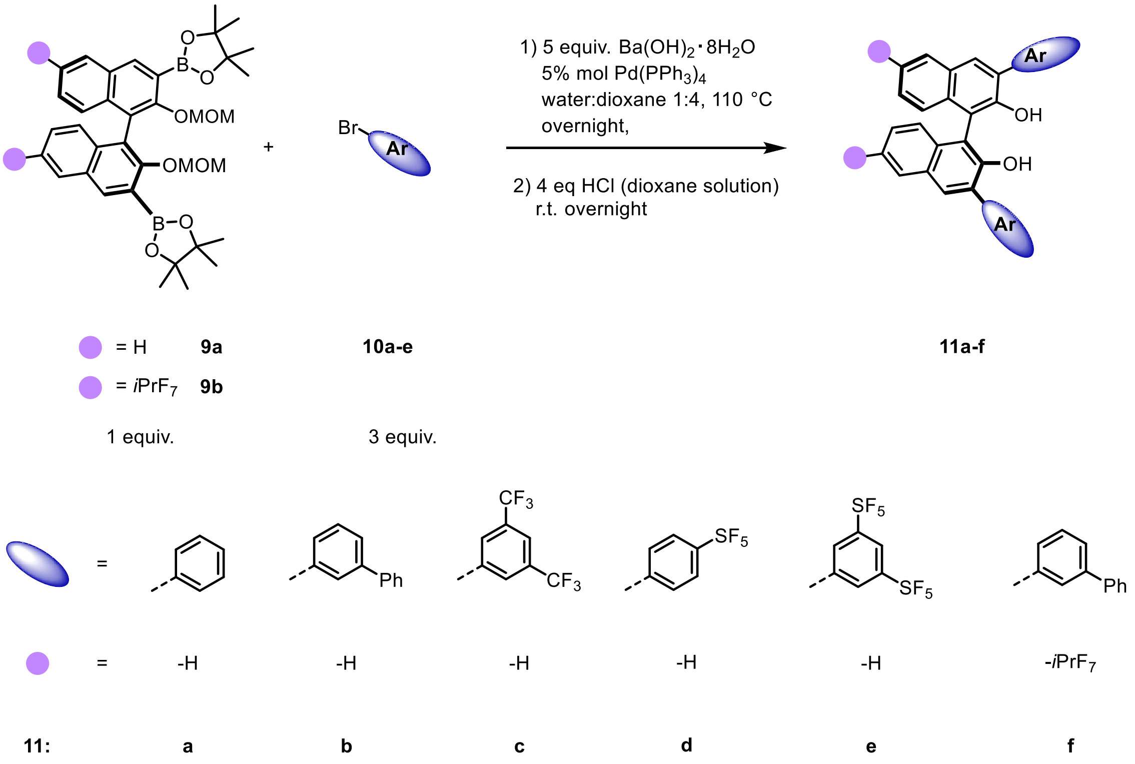

A Young tube (equipped with a magnetic stirring bar) was charged with **9a,b** (2.0 mmol, 1.0 equiv., 1.25 g), the corresponding bromide **10a–e** (3.0 equiv.), and barium hydroxide octahydrate (10.0 mmol, 5.0 equiv., 3.15 g). The solids were dissolved in 20 mL of a 4:1 dioxane:water (v/v) mixture and degassed using three freeze–pump–thaw cycles. $Pd(PPh_3)_4$ (0.01 mmol, 0.05 equiv., 115 mg) was then added under an argon atmosphere, followed by another freeze–pump–thaw cycle. The sealed Young tube was heated at 110 °C overnight. After completion of the reaction, the mixture was diluted with 10 mL of 10% aqueous HCl (m/m) and extracted with ethyl acetate (3 × 20 mL). The combined organic layers were dried over anhydrous Na_2SO_4, filtered, and concentrated under reduced pressure. The crude product was redissolved in 2 mL of 4 M HCl in dioxane and stirred overnight to effect MOM deprotection. After solvent removal under reduced pressure, the product was purified by column chromatography on silica gel using an 8:2 hexanes:ethyl acetate (v/v) mixture as the eluent. The (*S*)-BINOL derivatives were obtained from batches synthesized in parallel projects, following published procedures.[21,22] The products were isolated in the expected yields, and their spectral data matched those of previously synthesized batches. Compound (*S*)-BINOL 11f was fully characterized.

11f

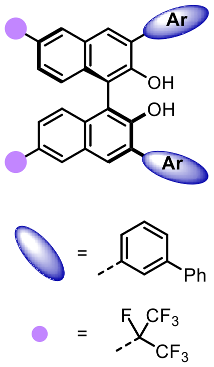

(S)-3,3'-di([1,1'-biphenyl]-3-yl)-6,6'-bis(perfluoropropan-2-yl)-[1,1'-binaphthalene]-2,2'-diol

(*S*)-11f was prepared according to the general procedure (from bromide **10b**) as a creamy-yellow crystals (92.75 % yield, 1.71 g, >95% purity) **^{1}H NMR:** (501 MHz, $CDCl_3$) δ 8.24 (d, *J* = 2.0 Hz, 2H), 8.19 (s, 2H), 7.94 (d, *J* = 1.9 Hz, 2H), 7.75 – 7.64 (m, 8H), 7.63 – 7.56 (m, 2H), 7.52 (d, *J* = 9.1 Hz, 2H), 7.49 – 7.42 (m, 4H), 7.41 – 7.33 (m, 4H), 5.57 (s, 2H). **^{19}F NMR:** (471 MHz, $CDCl_3$) δ -75.38 (m, 6F), -181.83 (hept, *J* = 7.3 Hz, 1F). **^{13}C NMR:** (126 MHz, $CDCl_3$) δ 151.9, 142.2, 140.9, 137.0, 134.0, 132.4, 132.3, 129.4, 129.0, 128.7, 128.6, 128.4, 127.8, 127.4, 127.4, 127.2, 127.1, 125.4, 123.5, 122.9, 122.7, 112.5. **HRMS**: (ESI-) (*m*/*z*) calculated for $C_{50}H_{27}O_2F_{14}$ $[M]^-$: 925.179304, found: 925.179640.

9.4. General procedure for the Synthesis of an IDPi Catalysts

The IDPi catalysts were synthesized according to the literature procedure.[21]

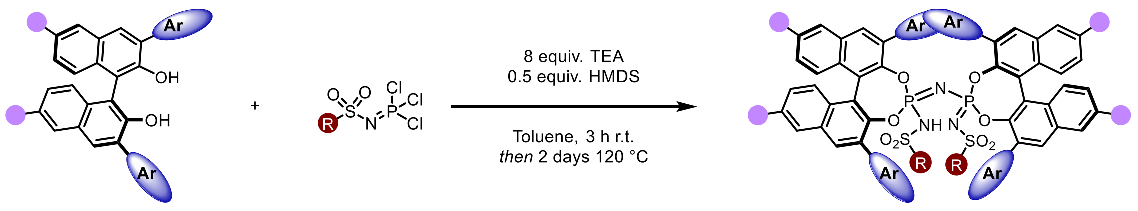

1 equiv. **11a-f** | 1 equiv. **9a-e** | **5a-m**

A Schlenk tube equipped with a magnetic stirring bar was evacuated, carefully flame-dried and filled with argon. The corresponding (*S*)-BINOL **11a–f** (1.02 equiv.), pre-dried overnight at room temperature under high vacuum (p = 10^{-2} mbar), was added and subsequently dissolved in dry, degassed toluene (c = 0.21 M). Dry triethylamine (9.0 equiv.) and the phosphazene reagent **8a–e** (1.0 equiv.) were then added. The reaction mixture was stirred at room temperature under argon for 3 hours. Next, dry and degassed hexamethyldisilazane (HMDS, 0.5 equiv.) was added, the Schlenk tube was sealed tightly, and the mixture was heated at 120 °C for 2 days. After cooling, the crude reaction mixture was directly purified by column chromatography on silica gel using a 95:5 toluene:ethyl acetate (v/v) mixture as the eluent to yield the catalyst salt. The catalyst salts were then acidified using 8 M aqueous HCl. For this, the catalyst salt was placed in a round-bottom flask and dissolved in DCM (10 mL per 300 mg of salt). Subsequently, 8 M HCl (10 mL per 300 mg of salt) was added to form a biphasic system, which was stirred vigorously at room temperature for 1 hour. After separation of the layers, the aqueous phase was extracted with DCM to recover any remaining catalyst. The combined organic layers were transferred to a separatory funnel, and a fresh portion of 8 M HCl (10 mL per 300 mg of catalyst) was added. The mixture was shaken vigorously, and the layers were separated again. The organic layer was collected and concentrated under reduced pressure. Finally, the catalyst was dried under vacuum for at least 1 hour.

The catalysts **5a,**[22] **b,**[22] **c,**[21] **d,**[22] **e,**[22] **f,**[22] **g,**[22] **i,**[22] **l,**[21] were prepared as in published methods. The batches used for this project were compared with those used previously in our group. The analytical data is presented for catalysts synthesized for this project for the first time.

5h

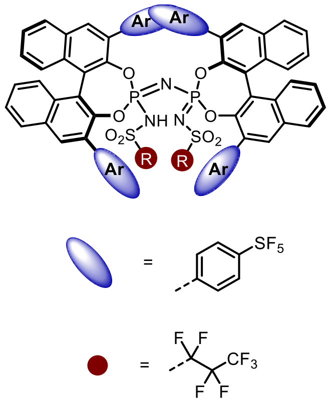

Catalyst **5h** was prepared from (*S*)-BINOL **11d** and phosphazine **8e** according to the general method. Product was obtained as white crystals (70% yield, 289 mg, >95% purity). **^{1}H NMR** (501 MHz, $CDCl_3$): δ 8.14 (d, *J* = 8.3 Hz, 2H), 8.10 (s, 2H), 8.04 (d, *J* = 8.3 Hz, 2H), 7.90 – 7.81 (m, 2H), 7.73 – 7.64 (m, 8H), 7.59 (ddd, *J* = 8.2, 6.6, 1.3 Hz, 2H), 7.44 – 7.37 (m, 6H), 7.36 – 7.30 (m, 4H), 6.63 (d, *J* = 8.4 Hz, 4H). **^{19}FNMR** (471 MHz, $CDCl_3$): δ 88.47 – 82.22 (m, 4F), 66.29 – 59.06 (m, 16F), -80.91 (t, *J* = 9.5 Hz, 6F), -112.65 – -112.83 (m, 4F), -124.38 – -124.86 (m, 4F). **^{31}PNMR** (203 MHz, $CDCl_3$): δ -10.58. **^{13}CNMR** (126 MHz, $CDCl_3$): δ 139.4, 139.0, 132.7, 132.6, 132.1, 132.0, 131.7, 131.1, 130.2, 129.8, 129.2, 129.0, 128.3, 127.6, 127.4, 127.2, 127.0, 126.9, 126.0, 125.2, 123.8, 122.1. In the spectra toluene and water are visible. **HRMS**: (ESI) (*m*/*z*) calculated for $C_{70}H_{36}O_8N_3F_{34}S_6P_2$ $[M]^+$: 1945.976475, found: 1945.977720.

5l

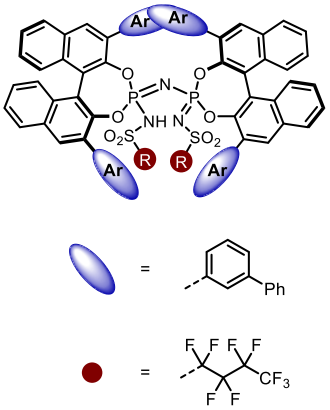

Catalyst **5l** was prepared from (*S*)-BINOL **11b** and phosphazine **8c** according to the general method as white crystals (64% yield, 232 mg, >95% purity). **^{1}H NMR** (501 MHz, $CDCl_3$): δ 8.13 (s, 2H), 7.99 (d, *J* = 8.3 Hz, 2H), 7.92 (d, *J* = 8.3 Hz, 2H), 7.84 – 7.73 (m, 4H), 7.66 – 7.59 (m, 5H), 7.58 – 7.50 (m, 5H), 7.46 (d, *J* = 7.6 Hz, 2H), 7.45 – 7.43 (m, 2H), 7.43 – 7.39 (m, 8H), 7.38 – 7.35 (m, 5H), 7.32 (t, *J* = 7.4 Hz, 6H), 7.29 – 7.27 (m,

3H), 7.24 – 7.15 (m, 8H), 7.15 – 7.07 (m, 5H), 7.02 (t, J = 7.8 Hz, 2H), 6.96 – 6.89 (m, 2H), 6.58 (d, J = 7.3 Hz, 2H), 5.39 (s, 2H). Some signals due to the overlaps may have integral different than number of protons expected. **^{19}F NMR** (471 MHz, $CDCl_3$): δ -80.72 (t, J = 10.1 Hz, 3F), -111.70 – -112.01 (m, 2F), -120.86 (p, J = 10.6 Hz, 2F), -125.94 (d, J = 13.6 Hz, 2F). **^{31}P NMR** (203 MHz, $CDCl_3$): δ -16.80. **^{13}C NMR** (126 MHz, $CDCl_3$): δ 143.9, 141.6, 140.5, 136.3, 133.5, 132.2, 132.1, 131.9, 131.8, 131.8, 131.3, 129.1, 128.8, 128.7, 128.4, 128.2, 128.1, 127.9, 127.6, 127.5, 127.4, 127.2, 127.0, 126.9, 126.6, 126.5, 123.6. **HRMS**: (API-) (m/z) calculated for $C_{96}H_{56}O_8N_3F_{18}S_2P_2$ $[M-H]^-$: 1846.27021, found: 1846.27051.

5m

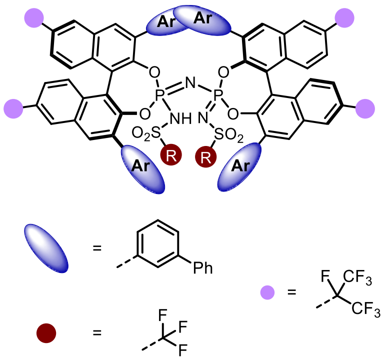

Catalyst **5m** was prepared from (S)-BINOL **11f** and phosphazine **8a** according to the general method and obtained as a creamy-yellow crystals (45% yield, 65 mg, >95% purity) **^{1}H NMR** (501 MHz, $CDCl_3$): δ 8.35 – 8.28 (m, 8H), 7.79 (s, 2H), 7.67 (d, J = 9.0 Hz, 2H), 7.63 – 7.58 (m, 5H), 7.57 – 7.53 (m, 2H), 7.53 – 7.47 (m, 2H), 7.48 – 7.44 (m, 2H), 7.43 – 7.39 (m, 6H), 7.37 – 7.30 (m, 2H), 7.24 – 7.17 (m, 12H), 7.16 – 7.07 (m, 8H), 7.06 – 7.00 (m, 2H), 6.96 (s, 2H), 6.36 (s, 2H). **^{19}F NMR** (471 MHz, $CDCl_3$): δ -74.85 – -75.60 (m), -78.22, -181.77 – -182.36 (m). Fluorine spectrum was not integrated due to the signal overlaps. **^{31}P NMR:** (203 MHz, $CDCl_3$): δ -16.34. **^{13}C NMR** (126 MHz, $CDCl_3$): δ 145.5, 142.7, 142.2, 141.1, 140.5, 135.4, 135.2, 135.0, 134.9, 133.2, 132.5, 132.3, 131.6, 131.4, 131.0, 129.3, 128.9, 128.6, 128.5, 128.3, 128.2, 128.0, 127.8, 127.7, 127.6, 127.6, 125.2, 124.0, 123.4, 123.1, 121.3. **HRMS**: (ESI) (m/z) calculated for $C_{102}H_{52}O_8N_3F_{34}S_2P_2$ $[M]^-$: 2218.213384, found: 2218.215650.

10. Upscaling Experiments

10.1. The Upscaling of Synthesis of 4a

A 50 mL Schlenk tube with a cooling jacket (equipped with a magnetic stirring bar) was dried overnight in a drying oven at 90 °C, then evacuated and filled with argon. At room temperature, IDPi-**5h** (0.36 mmol, 4 mol%, 0.04 equiv., 695 mg) and 5 Å molecular sieves (1.3 g, vacuum-dried at 120 °C for 4 days, p = 10^{-5} mbar) were added. The Schlenk tube was evacuated and refilled with argon once more. Anhydrous $CHCl_3$ (12 mL) was then added to suspend the molecular sieves in the catalyst solution. Cooling was applied, and once the temperature dropped below 0 °C, enone **2a** (8.9 mmol, 1.0 equiv., 1 mL) was added. The Schlenk tube was sealed with a septum and further cooled to −67 °C. Once the target temperature was reached, myrcene **3a** (4.0 equiv., 30 mmol, 6.1 mL) was added via syringe through the septum. The reaction mixture was stirred at −67 °C for 6 days. Upon completion, the catalyst was quenched by the addition of dry Et_3N (1.5 mmol, 210 µL), and stirring was continued for an additional 30 minutes at the reaction temperature. The mixture was then allowed to warm to room temperature, and the chloroform was removed under reduced pressure. The crude reaction mixture was purified by gradient flash column chromatography on silica gel. First, pure pentane was used to wash out unreacted diene, followed by a 97:3 (v/v) mixture of n-pentane:MTBE as the eluent to afford the desired product **4a** as a colorless liquid (1.95 g, 8.32 mmol, 93% yield, >20:1 r.r., 94:6 e.r.). Further the upscaling was performed on 13.4 mmol scale with amounts of reagents upscaled linearly to obtain identical results.

10.2. The Upscaling of Synthesis of 4w

A 5 mL flame-dried glass vial equipped with a magnetic stirring bar was charged with IDPi-**5c** (0.04 mmol, 2 mol%, 0.02 equiv.), 5 Å molecular sieves (400 mg), and methylcyclohexane (CyMe, 2.3 mL). To this solution, 1,1,1,3,3,3-hexafluoroisopropanol (HFIP, 1.31 mmol, 0.65 equiv., 138 µL; concentration in solvent: c = 0.57 M) was added. Mesityl oxide **2n** (2.0 mmol, 1.0 equiv., 230 µL) was then added, and the reaction vial was cooled to −80 °C. After 10 minutes, piperylene **3h** (4.0 mmol, 2.0 equiv., 400 µL) was added via syringe through the septum. The vial was then placed in a −40 °C temperature bath and stirred for 5 days. The reaction was quenched with Et_3N (approx. 40 µL), and stirring was continued for an additional 10 minutes. Upon warming, two phases were observed (CyMe and HFIP). The reaction mixture was then allowed to reach room temperature and was purified by flash column chromatography on silica gel using

1–3% Et_2O in hexane as the eluent. The desired product **4w** was obtained as a colorless oil with a strong odor reminiscent of rotten leaves and soil (229.4 mg, 1.38 mmol, 69% yield, 95:5 e.r., >20:1 r.r., >20:1 d.r.).

Note: During solvent removal, the rotary evaporator bath temperature was kept below 30 °C and pressure above 600 mbar to prevent product loss.

11. Catalyst recovery Experiments

11.1. The Recovery of IDPi-5h (for synthesis of 4a)

The recovery was performed for an upscaled reaction as described in Section 9.1. The catalyst was collected from the silica gel column used for purification of the reaction products by flushing with pure ethyl acetate. Elution of the catalyst was monitored by TLC (hexane:ethyl acetate 6:4). The catalyst-containing fractions were combined and dried overnight under vacuum (room temperature, $p < 10^{-2}$ mbar). Subsequent acidification was carried out using 8 M HCl, following the procedure outlined in General Procedure for the Synthesis of IDPi Catalysts (Section 8.4). The recovered catalyst was reused in the reaction as described in the Upscaling Experiments section (Section 9.1). The catalyst was successfully recovered and reused twice without significant loss in yield, regioselectivity, or stereoselectivity. The results are shown in Figure S12.

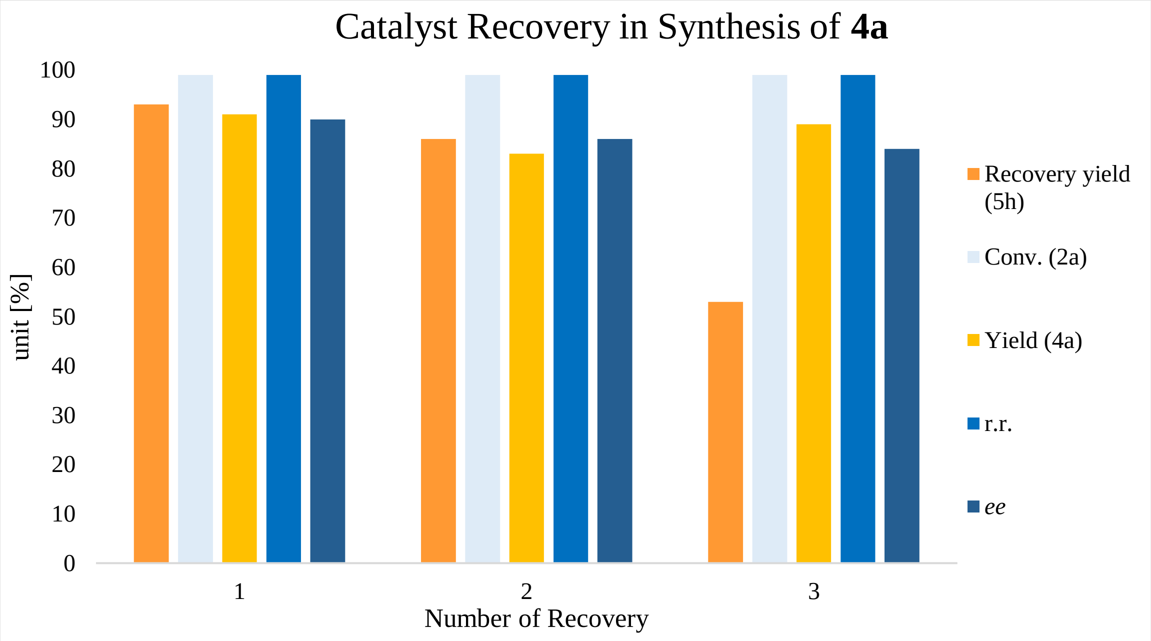

Figure S12. The recovery studies on Diels-Alder cycloaddition towards product **4a**.

Table S7. The recovery studies on Diels-Alder cycloaddition towards product **4a**.

Number of Recovery	Recovery yield (5h)[a]	Conv. (2n)[b]	Yield (4w)[c]	r.r.[d]	e.r.[d]
1	93%	99%	91%	>20:1	95:5
2	86%	99%	83%	>20:1	93:7
3	53%	99%	89%	>20:1	92:8

[a]Isolated yield of a catayst recovery is given in comparison to the mass of catalyst used initially. [b]Determined by ^{1}HNMR with 1 equiv. of 1,4-dioxane as an internal standard. [c]Yield of isolated compound after flash column chromatography. [d]Determined by GC or HPLC method as written together with analytical data for **4a**.

11.2. The Recovery of IDPi-5c (for synthesis of 4w)

The recovery was done as for **5h** and used for reaction as described in Section 9.2. The results are depicted on Figure S13.

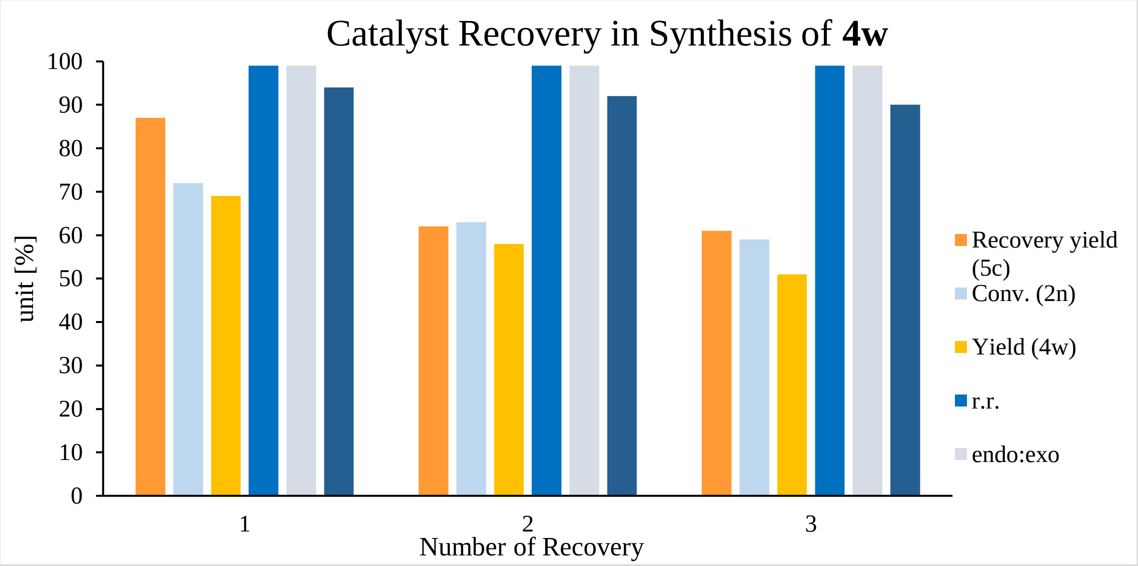

Figure S13. The recovery studies on Diels-Alder cycloaddition towards product **4w**.

Table S8. The recovery studies on Diels-Alder cycloaddition towards product **4w**.

**Number of Recovery**	**Recovery yield (5c)[a]**	**Conv. (2n)[b]**	**Yield (4w)[c]**	**r.r.[d]**	**endo:exo[g]**	**e.r.[d]**
1	87%	72%	69%	>20:1	>20:1	97:3
2	62%	63%	58%	>20:1	>20:1	96:4
3	61%	59%	51%	>20:1	>20:1	95:5

[a]Isolated yield of a catayst recovery is given in comparison to the mass of catalyst used initially. [b]Determined by ^{1}HNMR with 1 equiv. of 1,4-dioxane as an internal standard. [c]Yield of isolated compound after flash column chromatography. [d]Determined by GC method as written together with analytical data for **4w**.

12. Absolute Configuration determination by CD

12.1. Calculation of CD Spectra using DFT Methods

The CD-spectrum of **4n** (c = 1.0 x 10^{-4} M) was recorded in hexane (HPLC grade) at 20 °C and compared with the corresponding TD-DFT calculated CD spectra of the possible conformers. After a UV correction of the σ-value of 0.3 eV. The absolute configuration of the ketone **4n** was prepared by using (*S*, *S*)-**IDPi-5i** catalyst is *R*.

12.2. CD spectrum calculation

Density Functional Theory (DFT) calculations were performed on the Max-Planck-Institut für Kohlenforschung computer cluster using the ORCA program package (Version 5.0-Stable).[23] The geometry of the input structure was pre-optimized using the XTB2 method (the semi empirical GFN2-xTB method) by Grimme et. al.[24] and possible conformers were generated using CREST (Conformer-Rotamer Ensemble Sampling Tool) based on GFN methods by P.Pracht, S.Grimme et. al.[25] resulting in more than 1000 conformer structures, while for DFT calculations only 200 lowest energy conformers were taken. To minimize the number of conformers the RMSD was used and replicates (RMSD < 0.5) were removed before subsequent calculation steps. Structural optimizations and frequency calculations to identify all of the stationary points as minima (zero imaginary frequencies) and to obtain thermal and entropic correction were performed with the B3LYP functional[26] with D4 dispersion correction[27] along with RI approximation, utilizing the def2/J auxiliary basis set[28] and the def2-TZVP basis set on all atoms.[29] The libint2 library was used for the computation of 2-el integrals.[30] Tight SCF convergence and geometry optimization criterions were chosen.
The CD spectrum was computed by time-dependent density functional theory (TD-DFT, NROOTS = 25) at ωB97x/TZVPP level, solvent effects (hexane) were taken into account using the conductor-like polarized continuum model (CPCM).[31] The CD spectrum was created using Multiwfn[32] with a 0.3 eV half-width at ~1/3 height.

12.3. CD experiments for determination of the absolute configuration of Diels-Alder products 4n:

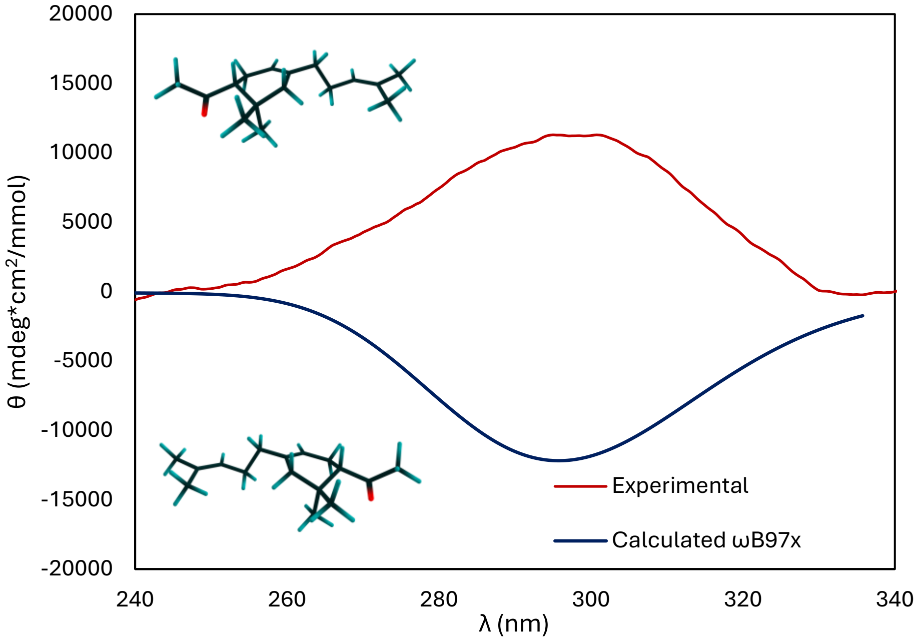

Figure S14. Comparison between the experimental CD-spectrum (red curve) and the calculated spectrum (blue curve) for the compound **4n**. Calculation details: Based on 116 conformers (Gfn-xTB method: CREST, Geom/Freq: B3LYP/*def2*-TZVP level of theory: TDDFT: ωB97x/*def2*-TZVP level of theory.

Calculated coordinates for *ent*-4n (representative conformer):

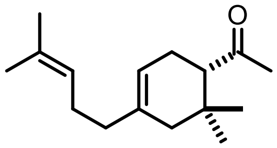

C	0.46794527270552	-1.79290123006661	0.58468317443882
C	-0.29028471227308	-0.92638807003270	-0.08461239551719
C	0.31331606306759	0.26546231079554	-0.78414527993597
C	1.72331024167156	0.64732554627817	-0.30118688420967
H	-0.34143959263276	1.13479721796057	-0.66393595893980
H	0.33402251705278	0.06413035173196	-1.86342628465899
C	2.55424537025034	-0.67256050529121	-0.26805554226203
C	1.63198726801303	1.30852431819629	1.08093534821545
C	2.33504447934560	1.64270284476468	-1.29273978200854

C	1.95557172961344	-1.68058791924476	0.72051454617768
C	4.02783700678848	-0.43967640208730	0.04025339381350
H	2.48689288960617	-1.09928389101902	-1.27446803054599
H	2.23008389385055	-1.39296612343642	1.74042236869925
H	2.41484417494636	-2.66192400838605	0.55969462123356
H	-0.00360239847056	-2.63961824450948	1.07586394319111
C	-1.78443128464576	-1.07065278670449	-0.17257173694687
C	-2.54048223983837	-0.10169691762583	0.76284402359544
H	-2.07400315008929	-2.09619075038903	0.07053405747300
H	-2.11614347491992	-0.88533326605084	-1.20040819203344
C	-4.02678319381272	-0.26456391481574	0.66777857109729
H	-2.24143490770604	0.92437771928329	0.54633263402966
H	-2.21766408916022	-0.30384086944246	1.78918118691598
C	-4.90721741642796	0.54911944386668	0.08065982831940
H	-4.41468158582099	-1.17738107138918	1.11525213467387
C	-4.55137477316077	1.83854782316778	-0.60545474739300
C	-6.37452001040790	0.21915722331567	0.06199393644995
H	-4.88327560800528	1.81871909477899	-1.64857145779752
H	-3.48405969798579	2.04957142188630	-0.59860831108486
H	-5.06629049561140	2.68143778439802	-0.13286779585819
H	-6.95937630076939	1.00811573218986	0.54671909374755
H	-6.58413627683654	-0.72178806134697	0.57122261381273
H	-6.74658648298611	0.14327882126489	-0.96524363769661
H	2.46472011303524	1.19356103188612	-2.28092083336398
H	3.30594323713506	2.00837304203778	-0.95120335825641
H	1.68233248560859	2.51127337627131	-1.40754579576680
H	2.61933429823692	1.52267387257916	1.48713500625627
H	1.10315186698674	0.67921993028246	1.79637883235879
H	1.08215806779137	2.24930136079420	0.99820324750702
C	5.00313941668794	-0.61440922672628	-1.1000428749946
H	6.01086065842668	-0.35935380819025	-0.77861454798821
H	4.71179351216251	0.00109005929190	-1.95385926195703
H	4.97881887292780	-1.65448537538453	-1.43974964395436
O	4.40709719255056	-0.14838252588246	1.15324707016378

13. Absolute Configuration determination by Mosher ester analysis[33]

13.1. Synthesis of Mosher ester of 4r

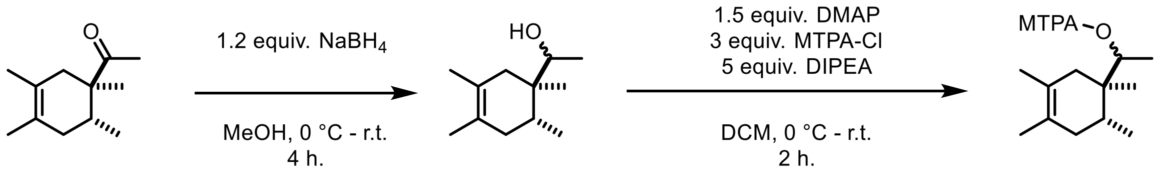

4r (1 equiv.) **12r** **(x)-13r**

Step 1: Reduction

In a dry round-bottom flask equipped with a magnetic stirring bar, ketone **4r** (1.0 equiv., 20 mg, 0.11 mmol) was dissolved in dry methanol (220 µL). The reaction mixture was cooled in an ice–water bath, and sodium borohydride (1.2 equiv., 0.13 mmol, 5 mg) was added slowly. The mixture was then stirred at room temperature for 4 hours (reaction progress monitored by TLC; 10% Et_2O in pentane, *p*-anisaldehyde stain; strong green spot corresponding to alcohol diastereoisomers). The reaction was quenched by adding 10 mL of cold saturated aqueous NH_4Cl, and the mixture was extracted with Et_2O (3 × 5 mL). The combined organic layers were dried over anhydrous Na_2SO_4, and the solvent was evaporated. The crude product was used directly in the next step without further purification.

Step 2: Ester Formation

The crude product from Step 1 was dissolved in dry DCM (5 mL) in the same flask, equipped again with a magnetic stirring bar. DMAP (1.5 equiv., 20 mg) and Mosher acid chloride (3.0 equiv., 63 µL) were added, and the reaction mixture was cooled to below 10 °C using an ice–water bath. DIPEA (5.0 equiv., 96 µL) was then added. The reaction was stirred until full conversion of the alcohol to the corresponding ester was observed (TLC monitoring; full conversion typically after 2 hours). The reaction was quenched by the addition of 10 mL of 1% aqueous HCl (v/v), and the layers were separated. The aqueous phase was extracted with DCM (3 × 5 mL), and the combined organic layers were dried over anhydrous Na_2SO_4. The solvent was removed under reduced pressure. The product was purified by column chromatography on silica gel using 5% Et_2O in pentane as the eluent (TLC: 5% Et_2O in pentane, p-anisaldehyde stain; strong blue spot corresponding to ester diastereoisomers). All Mosher esters were obtained as mixtures of diastereoisomers and were used as such for NMR-based assignment of absolute configuration.

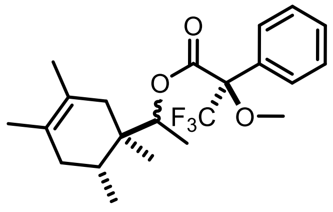

(*R*)-13r

Ester was synthesized as a mixture of diastereoisomers with 70% yield (0.078 mmol, 31 mg, 14:10 d.r.). **^{1}H NMR** (600 MHz, $CDCl_3$) δ 7.58 – 7.55 (m, 1H), 7.54 – 7.50 (m, 2H), 7.42 – 7.37 (m, 5H), 5.18 (q, *J* = 6.4 Hz, 1H), 5.10 (q, *J* = 6.4 Hz, 1H), 3.58 (q, *J* = 1.2 Hz, 2H), 3.50 (q, *J* = 1.2 Hz, 3H), 2.08 – 1.97 (m, 2H), 1.93 – 1.84 (m, 2H), 1.76 – 1.59 (m, 4H), 1.59 – 1.52 (m, 12H), 1.28 (d, *J* = 6.4 Hz, 2H), 1.23 (d, *J* = 6.4 Hz, 3H), 0.81 (d, *J* = 6.6 Hz, 2H), 0.77 (d, *J* = 6.6 Hz, 3H), 0.73 (d, *J* = 0.6 Hz, 3H), 0.69 (d, *J* = 0.6 Hz, 2H). **^{19}F NMR** (565 MHz, $CDCl_3$) δ -71.09, -71.12. **^{13}C NMR** (151 MHz, $CDCl_3$) δ 166.41, 166.31, 132.85, 132.39, 129.63, 129.60, 128.49, 128.44, 127.73 (q, *J* = 1.4 Hz), 127.41 (q, *J* = 1.4 Hz), 123.94, 123.90, 123.66 (q, *J* = 288.5 Hz), 123.62 (q, *J* = 288.6 Hz), 123.44, 123.35, 84.90 (q, *J* = 27.5 Hz), 84.42 (q, *J* = 27.7 Hz), 79.52, 77.86, 55.59 (q, *J* = 1.6 Hz), 55.38 (q, *J* = 1.4 Hz), 38.71, 38.52, 38.45, 38.33, 38.23, 37.55, 32.61, 31.96, 19.35, 19.30, 18.63, 18.62, 15.64, 15.54, 15.17, 15.15, 13.92, 13.90.

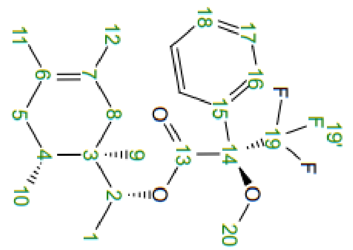

Atom	δ (ppm)	J	HSQC	COSY	HMBC	NOESY
1 C	13.900		1		2	
H3	1.225	6.40(2)	1	2	2, 3	2, 4, 8', 8", 9, 10, 16, 20
2 C	77.861		2		1, 9	
H	5.098	6.40(1)	2	1	1, 3, 4, 8, 9, 13	1, 4, 5', 8', 8", 9, 10
3 C	38.452				1, 2, 4, 5', 9, 10	
4 C	31.956		4		2, 5', 5", 8", 9, 10	
H	1.710	6.60(10)	4	5', 5", 10	3, 5, 9, 10	1, 2, 8', 10, 16, 20
5 C	38.327		5', 5"		4, 10	
H'	1.880		5	4, 5", 8', 11	3, 4, 6, 7, 10, 11	2, 5", 10, 11
H"	1.675		5	4, 5', 8', 11	4	5', 10
6 C	123.940				5', 11, 12	
7 C	123.349				5', 11, 12	
8 C	38.520		8', 8"		2, 9, 11, 12	
H'	2.031	0.60(9)	8	5', 5", 8", 9	9	1, 2, 4, 8", 20
H"	1.542		8	8'	4, 9, 12	1, 2, 8', 9
9 C	15.538		9		2, 4, 8', 8"	
H3	0.735	0.60(8')	9	8'	2, 3, 4, 8	1, 2, 8", 16, 20
10 C	15.172		10		4, 5'	
H3	0.772	6.60(4)	10	4	3, 4, 5	1, 2, 4, 5', 5"

Atom	δ (ppm)	J	HSQC	COSY	HMBC	NOESY
11 C	18.622		11		5'	
H3	1.565		11	5', 5"	6, 7, 8	5'
12 C	19.350		12		8"	
H3	1.558		12		6, 7, 8	
13 C	166.407				2	
14 C	84.896	27.50(19')			16, 20	
15 C	132.394				17	
16 C	127.732	1.40(19')	16		18	
H	7.516		16	17	14, 18	1, 4, 9, 20
17 C	128.487		17		17	
H	7.394		17	16	15, 17	
18 C	129.630		18		16	
H	7.393		18		16	
19 C	123.622	288.60(19')				
19' F	-71.093	1.20(20H), 1.40(20), 288.60(19), 1.40(16), 27.50(14)				
20 C	55.383	1.40(19')	20			
H3	3.503	1.20(19'F)	20		14	1, 4, 8', 9, 16

Table S9. Configuration assignment of *cis*-diastereoisomer (*R*)-**13r**.

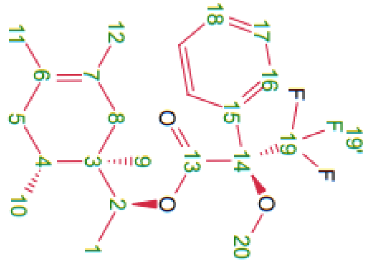

Atom	δ (ppm)	J	HSQC	COSY	HMBC	NOESY	Atom	δ (ppm)	J	HSQC	COSY	HMBC	NOESY
1 C	13.918		1		2		11 C	18.635		11		5'	
H3	1.281	6.40(2)	1	2	2, 3	2, 4, 8', 9, 10, 16, 20	H3	1.564		11			5'
2 C	79.522		2		1, 9		12 C	19.300		12		8"	
H	5.177	6.40(1)	2	1	1, 3, 8, 9, 13	1, 4, 5', 8', 9, 10	H3	1.551		12			
3 C	38.706				1, 2, 4, 5', 9, 10		13 C	166.310				2	
4 C	32.612		4		5', 5", 8", 9, 10		14 C	84.422	27.70(19')			16, 20	
H	1.619	6.60(10)	4	5', 5", 10	3, 5, 8, 9, 10	1, 2, 8', 10	15 C	132.855				17	
5 C	38.228		5', 5"		4, 10		16 C	127.411	1.40(19')	16		18	
H'	1.886		5	4, 5"	3, 4, 6, 7, 10, 11	2, 5", 10, 11	H	7.565		16	17	14, 18	1, 9, 20
H"	1.667		5	4, 5'	4	5', 10	17 C	128.441		17		17	
6 C	123.905				5'		H	7.394		17	16	15, 17	
7 C	123.440				5'		18 C	129.599		18		16	
8 C	37.554		8', 8"		2, 4, 9		H	7.398		18		16	
H'	2.009	0.60(9)	8	8", 9	9	1, 2, 4, 8"	19 C	123.661	288.50(19')				
H"	1.542		8	8'	4, 9, 12	8', 9, 20	19' F	-71.119	1.20(20H), 1.60(20), 288.50(19), 1.40(16), 27.70(14)				
9 C	15.639		9		2, 4, 8', 8"		20 C	55.587	1.60(19')	20			
H3	0.694	0.60(8')	9	8'	2, 3, 4, 8	1, 2, 8", 10, 16, 20	H3	3.583	1.20(19'F)	20		14	1, 8", 9, 16
10 C	15.154		10		4, 5'								
H3	0.808	6.60(4)	10	4	3, 4, 5	1, 2, 4, 5', 5", 9							

Table S10. Configuration assignment of *trans*-diastereoisomer (*R*)-**13r**.

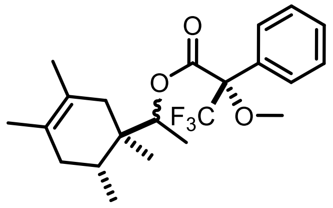

(*S*)-13r

Ester was synthesized as a mixture of diastereoisomers with 86% yield (0.095 mmol, 38 mg, 15:10 d.r.). **^{1}H NMR** (600 MHz, $CDCl_3$) δ 5.15 (q, *J* = 6.4 Hz, 1H), 5.11 (q, *J* = 6.4 Hz, 1H), 3.57 (q, *J* = 1.3 Hz, 3H), 3.52 (q, *J* = 1.1 Hz, 2H), 1.30 (d, *J* = 6.4 Hz, 3H), 1.21 (d, *J* = 6.4 Hz, 2H), 0.82 (d, *J* = 6.6 Hz, 2H), 0.76 (d, *J* = 0.5 Hz, 2H), 0.71 (d, *J* = 0.5 Hz, 3H), 0.71 (d, *J* = 6.5 Hz, 2H). **^{19}F NMR** (565 MHz, $CDCl_3$) δ -71.09, -71.16. **^{13}C NMR** (151 MHz, $CDCl_3$) δ 166.55, 166.15, 132.80, 132.37, 129.63, 129.57, 128.50, 128.41, 127.81 (q, *J* = 1.6 Hz), 127.36 (q, *J* = 1.4 Hz), 123.96, 123.88, 123.62 (q, *J* = 288.8 Hz), 123.47, 123.23, 84.94 (q, *J* = 27.7 Hz), 84.36 (q, *J* = 27.6 Hz), 79.72, 77.69, 55.56 (q, *J* = 1.6 Hz), 55.39 (q, *J* = 1.4 Hz), 38.59, 38.54, 38.48, 38.27, 38.22, 37.68, 32.65, 31.71, 19.35, 19.29, 18.63, 18.60, 16.00, 15.45, 15.16, 15.14, 14.14, 13.70.

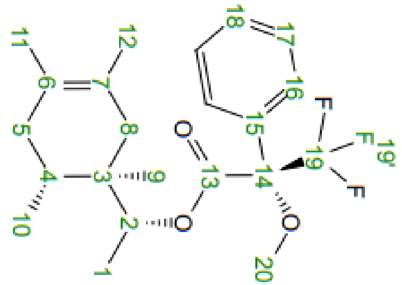

Atom	δ (ppm)	J	HSQC	COSY	HMBC	NOESY
1 C	14.144		1		2	
H3	1.302	6.40(2)	1	2	2, 3	2, 4, 8', 8", 9, 10, 16, 20
2 C	79.723		2		1, 9	
H	5.111	6.40(1)	2	1	1, 3, 4, 8, 9, 13	1, 4, 5', 8', 8", 9, 10
3 C	38.543				1, 2, 4, 5', 9, 10	
4 C	31.708		4		2, 5', 5", 8", 9, 10	
H	1.603	6.50(10)	4	5', 5", 10	3, 5, 9, 10	1, 2, 8', 10, 16, 20
5 C	38.273		5', 5"		4, 10	
H'	1.833		5	4, 5", 8', 11	3, 4, 6, 7, 10, 11	2, 5", 10, 11
H"	1.629		5	4, 5', 8', 11	4	5', 10
6 C	123.963				5', 11, 12	
7 C	123.232				5', 11, 12	
8 C	38.476		8', 8"		2, 9, 11, 12	
H'	2.026	0.50(9), 17.20(8")	8	5', 5", 8", 9	9	1, 2, 4, 8", 20
H"	1.546	17.20(8')	8	8'	4, 9, 12	1, 2, 8', 9
9 C	15.453		9		2, 4, 8', 8"	
H3	0.713	0.50(8')	9	8'	2, 3, 4, 8	1, 2, 8", 16, 20
10 C	15.136		10		4, 5'	
H3	0.709	6.50(4)	10	4	3, 4, 5	1, 2, 4, 5', 5"

Atom	δ (ppm)	J	HSQC	COSY	HMBC	NOESY
11 C	18.596		11		5'	
H3	1.552		11	5', 5"	6, 7, 8	5'
12 C	19.348		12		8"	
H3	1.556		12		6, 7, 8	
13 C	166.147				2	
14 C	84.359	27.60(19')			16, 20	
15 C	132.802				17	
16 C	127.357	1.40(19')	16		18	
H	7.552		16		14, 18	1, 4, 9, 20
17 C	128.415		17		17	
H	7.383		17		15, 17	
18 C	129.575		18		16	
H	7.387		18		16	
19 C	123.623	288.80(19')				
19' F	-71.093	1.40(16), 288.80(19), 1.60(20), 27.60(14), 1.30(20H)				
20 C	55.564	1.60(19')	20			
H3	3.568	1.30(19'F)	20		14	1, 4, 8', 9, 16

Table S11. Configuration assignment of *cis*-diastereoisomer of (*S*)-**13r**.

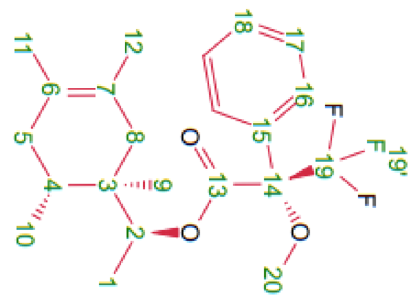

Atom	δ (ppm)	J	HSQC	COSY	HMBC	NOESY
1 C	13.696		1		2	
H3	1.208	6.40(2)	1	2	2, 3	2, 4, 8', 9, 10, 16, 20
2 C	79.723		2		1, 9	
H	5.152	6.40(1)	2	1	1, 3, 8, 9, 13	1, 4, 5', 8', 9, 10, 16
3 C	38.594				1, 2, 4, 5', 9, 10	
4 C	32.652		4		5', 5", 8", 9, 10	
H	1.653	6.60(10)	4	5', 5", 10	3, 5, 8, 9, 10	1, 2, 8', 10
5 C	38.225		5', 5"		4, 10	
H'	1.903		5	4, 5"	3, 4, 6, 7, 10, 11	2, 5", 10, 11
H"	1.688		5	4, 5'	4	5', 10
6 C	123.879				5', 8'	
7 C	123.469				5', 8'	
8 C	37.682		8', 8"		2, 4, 9	
H'	1.998	0.50(9)	8	8", 9	6, 7, 9	1, 2, 4, 8"
H"	1.549		8	8'	4, 9, 12	8', 9, 20
9 C	16.002		9		2, 4, 8', 8"	
H3	0.760	0.50(8')	9	8'	2, 3, 4, 8	1, 2, 8", 16, 20
10 C	15.160		10		4, 5'	
H3	0.820	6.60(4)	10	4	3, 4, 5	1, 2, 4, 5', 5"

Atom	δ (ppm)	J	HSQC	COSY	HMBC	NOESY
11 C	18.626		11		5'	
H3	1.568		11			5'
12 C	19.292		12		8"	
H3	1.556		12			
13 C	166.551				2	
14 C	84.940	27.70(19')			16, 20	
15 C	132.366				17	
16 C	127.810	1.60(19')	16		18	
H	7.536		16		14, 18	1, 2, 9, 20
17 C	128.502		17		17	
H	7.406		17	17	15, 17	
18 C	129.634		18		16	
H	7.403		18		16	
19 C	123.623	288.80(19')				
19' F	-71.161	1.20(20H), 1.60(16), 288.80(19), 1.40(20), 27.70(14)				
20 C	55.386	1.40(19')	20			
H3	3.519	1.20(19'F)	20		14	1, 8", 9, 16

Table S12. Configuration assignment of *trans*-diastereoisomer of (*S*)-**13r**.

13.2. Analysis of Mosher esters

The relative stereochemistry was established based on a combination of NOE data, $^3J_{CH}$ couplings, and conformational analysis (with atom numbering as shown in Figure S15). The absolute configuration was determined using Mosher ester analysis. The results are presented in Table S9 (major diastereomer, *R*-ester), Table S10 (minor diastereomer, *R*-ester), Table S11 (major diastereomer, *S*-ester), and Table S12 (minor diastereomer, *S*-ester). A careful analysis of both diastereomers supports the assignment of the absolute configuration at positions 3 and 4 as 3*R*, 4*R* (see Figure S15).

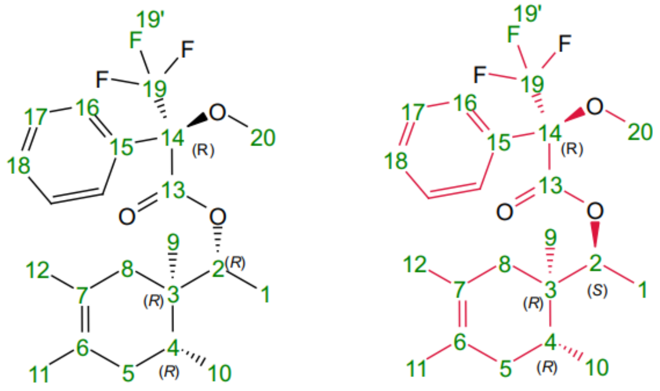

Figure S15. Numbering of atoms in analyzed Mosher ester.

Observations that lead to the determination of the relative stereochemistry:

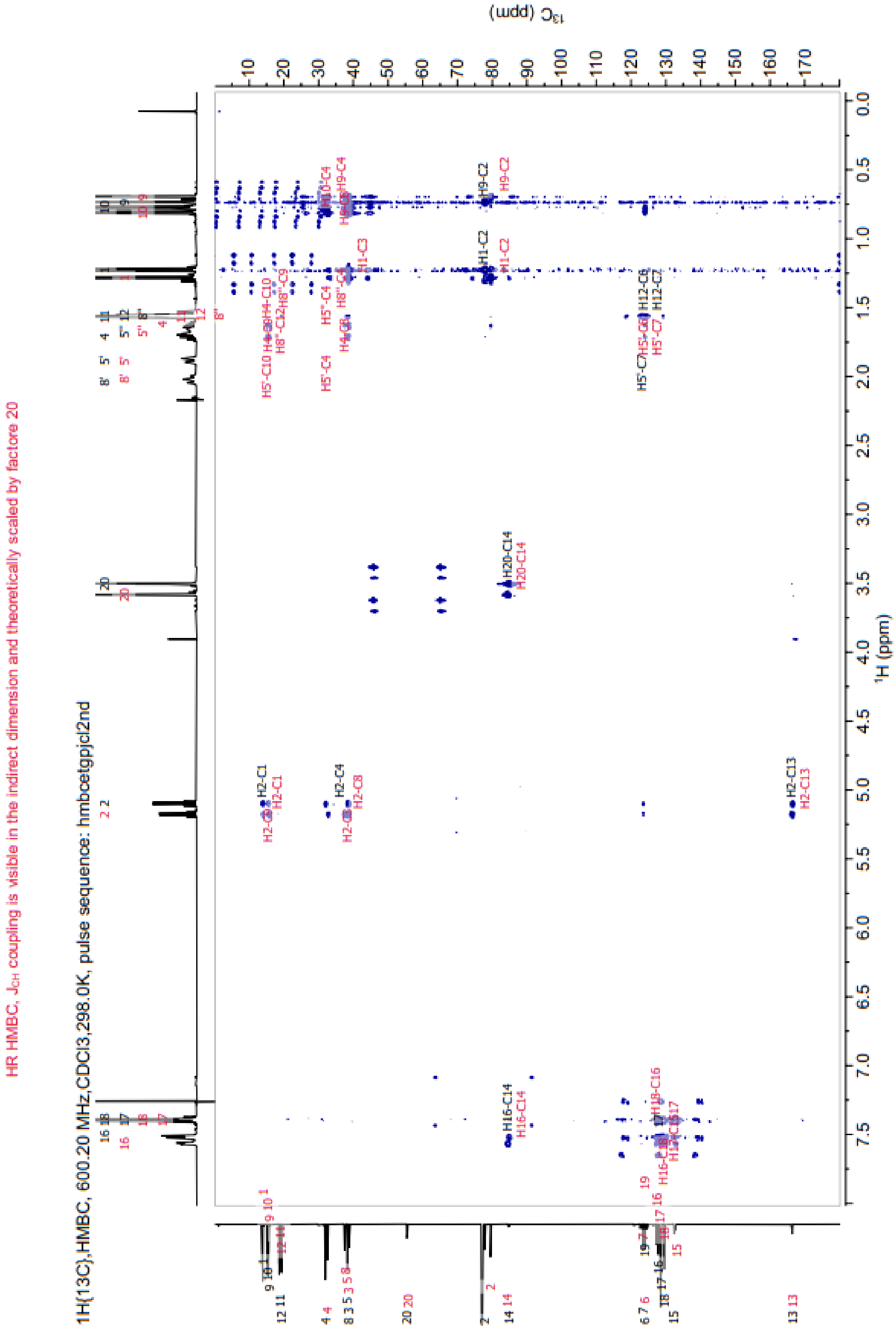

Figure S16. ^{1}H, ^{13}C-HR-HMBC of the ***(R)*-13r**.

The ^{1}H, ^{13}C HR-HMBC spectrum (Figure S16) enabled comparison of the $^{n}J_{CH}$ coupling constants between H2 and carbons C3, C4, C8, and C9. The largest observed coupling was $^{3}J_{H2–C8} \approx 4$ Hz. This indicates that in the structure of the dominant conformer H2 proton is in *trans* position relatively to C8. Comparison of the NOE cross-peaks for the two diastereomers revealed that the major diastereomer shows NOE cross-relaxation between H1 and both H8″ and H8′, whereas the minor diastereomer exhibits a cross-peak only between H1 and H8′. These observations are in good agreement with the Newman projections shown in Figure S17.

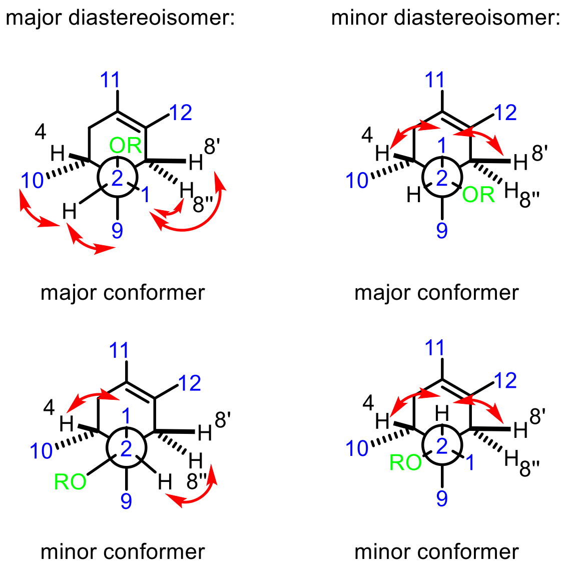

major diastereoisomer:

J_{H2C3} = ~2.5 Hz J_{H2C9} = ~2.5 Hz

J_{H2C4} = ~2.0 Hz J_{H4C9} = ~5 Hz (*trans*)

J_{H2C8} = ~4.0 Hz (*trans* dominating)

minor diastereoisomer:

J_{H2C3} = ~2.2 Hz J_{H2C9} = ~3.5 Hz

J_{H2C4} = ~1.5 Hz J_{H4C9} = ~5 Hz (*trans*)

J_{H2C8} = ~4.3 Hz (*trans* dominating)

NOE effects

NOE_H4H1 < NOE_H4H8'/H8"

NOE_H4H1 < NOE_H4H1

NOE_H8'H1 < NOE_H8'H1

NOE_H8"H1 > NOE_H8'H1'

Figure S17. Newman projections of the selected conformers of **(*R*)-13r**.

Additionally, H2 shows NOE cross-peaks to both H8′ and H8″, which likely arise from a less populated conformer within the major diastereomer. In contrast, H2 in the minor diastereomer displays only a weak NOE to H8′ and none to H8″. The $^{3}J_{H2–C9}$ coupling constant in the minor diastereomer is significantly larger than in the major one, whereas the opposite trend is observed for $^{3}J_{H2–C4}$. The relatively large $^{3}J_{H4–C9}$ coupling (~5 Hz, indicative of a trans relationship) is consistent with the methyl groups at positions 9 and 10 being cis to each other, with the H4 proton predominantly adopting an axial orientation within the conformer ensemble. In the minor diastereomer, a strong NOE cross-peak is observed between H9 and H10, which do not significantly overlap in the spectrum, further supporting this assignment. Both diastereomers also show NOE cross-peaks from H2 to H5′, likely due to contributions from a conformer in which Me-10 is axial and Me-9 occupies the equatorial position. All relevant NOE interactions and J_{CH} couplings are

depicted in Figure S17. Taken together, the NMR data support the relative stereochemistry as presented in Figure S15.

Observations that lead to the determination of the absolute stereochemistry:

The analysis suggests that the major diastereomer is (*R*) configured on C2 as described in Table S13.

Table S13. Analysis of the Mosher esters of major diastereoisomer of **13r**.

**Major diastereoisomer**		(*S*)-Ester		(*R*)-Ester		
Atom		δ (ppm)		δ (ppm)		ΔδSR (ppm)
1 C		14.144		13.9		
H3		1.302		1.225		0.08
2 C		77.688		77.861		
H		5.111		5.098		0.01
3 C		38.543		38.452		
4 C		31.708		31.956		
H		1.603		1.71		-0.11
5 C		38.273		38.327		
H'		1.833		1.88		-0.05
H"		1.629		1.675		-0.05
6 C		123.963		123.94		
7 C		123.232		123.349		
8 C		38.476		38.52		
H'		2.026		2.031		-0.01
H"		1.549		1.542		0.01
9 C		15.453		15.538		
H3		0.713		0.735		-0.02
10 C		15.136		15.172		
H3		0.709		0.772		-0.06
11 C		18.596		18.622		
H3		1.552		1.565		-0.01
12 C		19.348		19.35		
H3		1.556		1.558		0.00

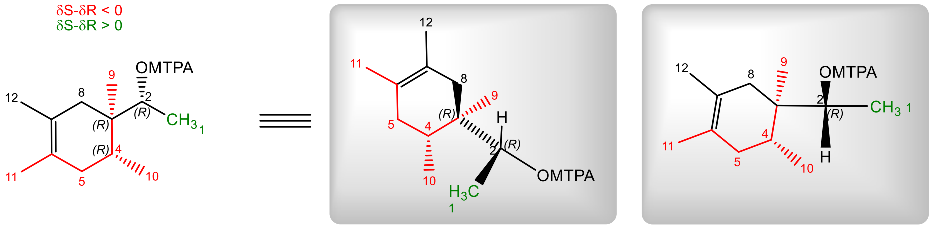

The analysis suggests that the minor diastereomer is (*S*) configured on C2 as described in Table S14.

Table S14. Analysis of the Mosher esters of major diastereoisomer of **13r**.

Minor diastereoisomer		(*S*)-Ester	(*R*)-Ester	
Atom		δ (ppm)	δ (ppm)	ΔδSR (ppm)
1 C		13.696	13.918	
H3		1.208	1.281	-0.07
2 C		79.723	79.522	
H		5.152	5.177	-0.02
3 C		38.594	38.706	
4 C		32.652	32.612	
H		1.653	1.619	0.03
5 C		38.225	38.228	
H'		1.903	1.886	0.02
H"		1.688	1.667	0.02
6 C		123.879	123.905	
7 C		123.469	123.44	
8 C		37.682	37.554	
H'		1.998	2.009	-0.01
H"		1.549	1.542	0.01
9 C		16.002	15.639	
H3		0.76	0.694	0.07
10 C		15.16	15.154	
H3		0.82	0.808	0.01
11 C		18.626	18.635	
H3		1.568	1.564	0.00
12 C		19.292	19.3	
H3		1.556	1.551	0.01

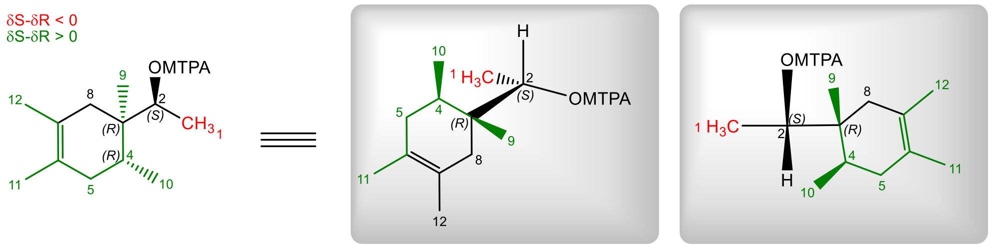

The data collected above when analyzed as depicted in Figure S18 support *R* configuration on C4.

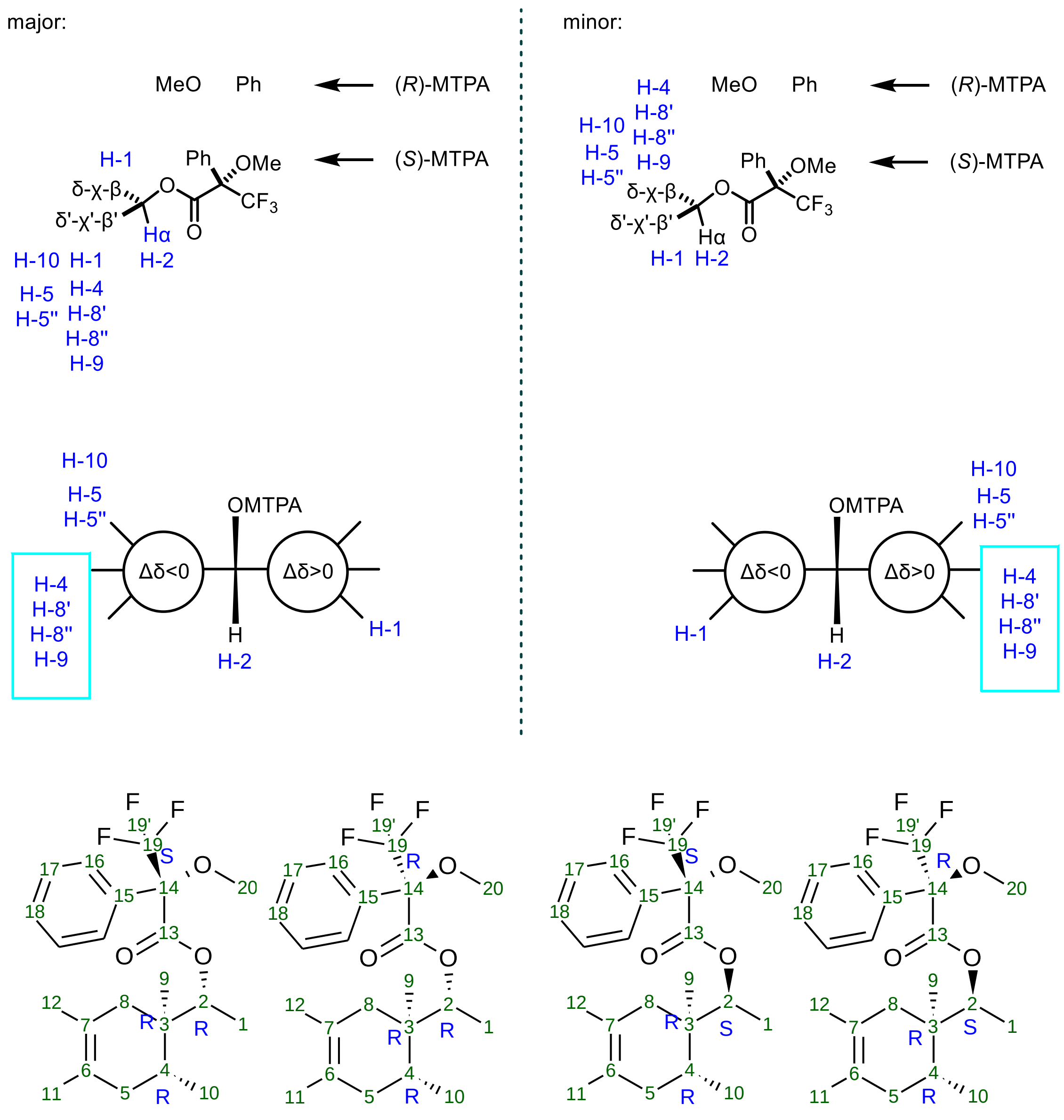

Figure S18. The Analysis of absolute stereochemistry.

14. Absolute Configuration determination by single crystal X-ray diffraction (SC-XRD)

14.1. Syntheses of osmate esters for structural analyses

Osmate esters were synthesized according to the literature method.[34]

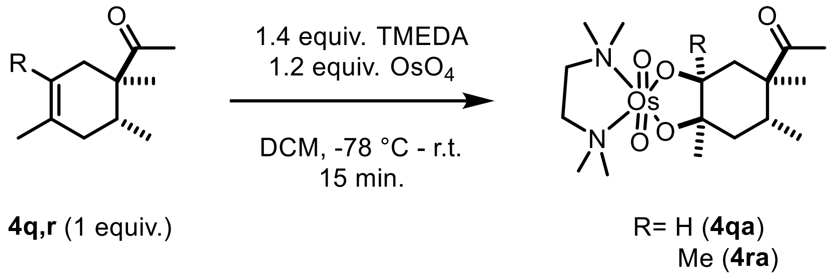

In a flame-dried vial equipped with a magnetic stirring bar, ketone **4q** or **4r** (1.0 equiv.) was dissolved in DCM (c = 1 M), followed by the addition of TMEDA (1.4 equiv.). The vial was sealed with a cap, protected with parafilm, and cooled in a dry ice bath. The mixture was stirred in the dry ice bath for 20 minutes, after which a stock solution of OsO_4 (1.2 equiv., c = 1.4 M) was added via syringe through the cap. The reaction mixture initially turned red, then brown. Stirring was continued in the dry ice bath for an additional 15 minutes, after which the reaction was allowed to slowly warm to room temperature. Once TLC analysis (DCM:EtOH 8:2, PMA stain) indicated full consumption of the starting material and formation of the product, the crude reaction mixture was directly subjected to silica gel column chromatography and eluted with a DCM:EtOH (8:2, v/v) mixture to afford the pure osmate ester. For X-ray analysis, the osmate esters were crystallized using the vapor diffusion method with THF and pentane as co-solvents. Yellow-brown crystals were obtained.

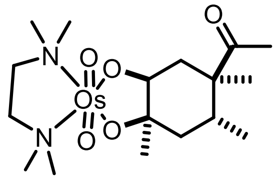

4qa

Product was obtained from 7.8 mg (0.05 mmol) of ketone as brown-yellow crystals with 76.6% yield (0.036 mmol, 19.3 mg) and crystalized with pentane from 200 µL of THF. **^{1}H NMR** (501 MHz, $CDCl_3$): δ 3.65 (dd, *J* = 10.5, 6.0 Hz, 1H), 3.11 – 3.02 (m, 4H), 2.83 (d, *J* = 2.2 Hz, 6H), 2.78 (d, *J* = 5.2 Hz, 6H), 2.44 (dd, *J* = 12.9, 10.5 Hz, 1H), 2.37 – 2.28 (m, 1H), 2.23 (dd, *J* = 14.7, 4.1 Hz, 1H), 2.12 (s, 3H), 2.00 (dd, *J* = 14.8, 12.3 Hz, 1H),

1.41 – 1.33 (m, 1H), 1.30 (s, 3H), 1.05 (s, 3H), 0.71 (d, *J* = 6.8 Hz, 3H). **^{13}C NMR** (126 MHz, $CDCl_3$): δ 214.3, 87.1, 64.4, 64.0, 52.6, 51.7, 51.5, 51.3, 51.1, 41.9, 35.7, 30.4, 26.5, 25.0, 16.6, 13.0. **HRMS**: (ESIpos) (m/z) calculated for $C_{17}H_{34}N_2O_5Os$ $[M+Na]^+$: 561.19747, found: 561.19741.

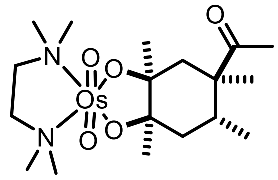

4ra

Product was obtained from 28.1 mg (0.17 mmol) of ketone as brown-yellow crystals with 60% yield (0.102 mmol, 56 mg) and crystalized with pentane from 200 μL of THF. **^{1}H NMR** (501 MHz, $CDCl_3$): δ 3.14 – 2.95 (m, 4H), 2.84 – 2.71 (m, 14H), 2.46 (ddp, *J* = 11.0, 7.0, 3.5 Hz, 1H), 2.14 (s, 3H), 2.09 (dd, *J* = 14.5, 3.9 Hz, 1H), 1.77 (dd, *J* = 14.4, 11.3 Hz, 1H), 1.50 (s, 3H), 1.16 (s, 3H), 1.05 (s, 3H), 0.73 (d, *J* = 7.0 Hz, 3H). **^{13}C NMR** (126 MHz, $CDCl_3$): δ 214.1, 88.4, 87.3, 63.8, 51.9, 51.0, 51.0, 50.9, 50.8, 50.8, 41.8, 41.1, 29.3, 25.7, 24.9, 23.4, 16.5, 15.9. **HRMS**: (ESIpos) (m/z) calculated for $C_{18}H_{36}N_2O_5Os$ $[M+Na]^+$: 575.21312, found: 575.21320.

14.2. Structural analyses of the single crystals of osmate esters

14.2.1. Analysis of 4qa

Table S15. Crystal data and structure refinement. CCDC: 2472884

Identification code	16240	
Empirical formula	C_{17} H_{34} N_2 O_5 Os	
Color	yellow	
Formula weight	536.66 g·mol^{-1}	
Temperature	100(2) K	
Wavelength	0.71073 Å	
Crystal system	Monoclinic	
Space group	$P2_1$, (no. 4)	
Unit cell dimensions	a = 7.6404(2) Å	α= 90°.
	b = 11.0961(3) Å	β= 97.6687(15)°.

	c = 12.0511(3) Å	γ = 90°.
Volume	1012.54(5) $Å^3$	
Z	2	
Density (calculated)	1.760 $Mg \cdot m^{-3}$	
Absorption coefficient	6.324 mm^{-1}	
F(000)	532 e	
Crystal size	0.112 x 0.061 x 0.031 mm^3	
θ range for data collection	2.505 to 32.630°.	
Index ranges	$-11 \le h \le 11$, $-16 \le k \le 16$, $-18 \le l \le 18$	
Reflections collected	155580	
Independent reflections	7427 [R_{int} = 0.0593]	
Reflections with I>2σ(I)	7186	
Completeness to θ = 25.242°	99.9 %	
Absorption correction	Gaussian	
Max. and min. transmission	0.86543 and 0.64829	
Refinement method	Full-matrix least-squares on F^2	
Data / restraints / parameters	7427 / 1 / 234	
Goodness-of-fit on F^2	1.107	
Final R indices [I>2σ(I)]	R_1 = 0.0154	wR^2 = 0.0357
R indices (all data)	R_1 = 0.0166	wR^2 = 0.0362
Absolute structure parameter	-0.013(3)	
Extinction coefficient	n/a	
Largest diff. peak and hole	1.672 and -0.694 $e \cdot Å^{-3}$	

Table S16. Bond lengths [Å] and angles [°].

Os(1)-O(1)	1.964(2)	Os(1)-O(2)
1.9452(19)	Os(1)-O(3)	1.742(2)
Os(1)-O(4)	1.746(2)	Os(1)-N(1)
2.217(3)	Os(1)-N(2)	2.239(2)
O(1)-C(1)	1.441(4)	O(2)-C(2)
1.442(5)	O(5)-C(10)	1.229(5)
N(1)-C(12)	1.501(4)	N(1)-C(15)
1.489(4)	N(1)-C(16)	1.485(4)
N(2)-C(14)	1.487(4)	N(2)-C(17)
1.491(5)	N(2)-C(18)	1.488(4)
C(1)-H(1)	1.0000	C(1)-C(2)
1.523(4)	C(1)-C(6)	1.526(4)
C(2)-C(3)	1.531(4)	C(2)-C(7)
1.526(4)	C(3)-H(3A)	0.9900
C(3)-H(3B)	0.9900	C(3)-C(4)
1.539(4)	C(4)-H(4)	1.0000
C(4)-C(5)	1.572(5)	C(4)-C(8)
1.518(4)	C(5)-C(6)	1.541(4)
C(5)-C(9)	1.527(6)	C(5)-C(10)
1.543(8)	C(6)-H(6A)	0.9900
C(6)-H(6B)	0.9900	C(7)-H(7A)
0.9800	C(7)-H(7B)	0.9800
C(7)-H(7C)	0.9800	C(8)-H(8A)
0.9800	C(8)-H(8B)	0.9800
C(8)-H(8C)	0.9800	C(9)-H(9A)
0.9800	C(9)-H(9B)	0.9800
C(9)-H(9C)	0.9800	C(10)-C(11)
1.493(11)	C(11)-H(11A)	0.9800
C(11)-H(11B)	0.9800	C(11)-H(11C)
0.9800	C(12)-H(12A)	0.9900
C(12)-H(12B)	0.9900	C(12)-C(14)
1.519(5)	C(14)-H(14A)	0.9900
C(14)-H(14B)	0.9900	C(15)-H(15A)
0.9800	C(15)-H(15B)	0.9800

C(15)-H(15C)	0.9800	C(16)-H(16A)
0.9800	C(16)-H(16B)	0.9800
C(16)-H(16C)	0.9800	C(17)-H(17A)
0.9800	C(17)-H(17B)	0.9800
C(17)-H(17C)	0.9800	C(18)-H(18A)
0.9800	C(18)-H(18B)	0.9800
C(18)-H(18C)	0.9800	

O(1)-Os(1)-N(1)	178.70(10)	O(1)-Os(1)-N(2)
96.99(11)	O(2)-Os(1)-O(1)	84.32(13)
O(2)-Os(1)-N(1)	96.76(12)	O(2)-Os(1)-N(2)
178.67(14)	O(3)-Os(1)-O(1)	96.96(12)
O(3)-Os(1)-O(2)	96.87(12)	O(3)-Os(1)-O(4)
161.23(11)	O(3)-Os(1)-N(1)	82.21(10)
O(3)-Os(1)-N(2)	83.18(11)	O(4)-Os(1)-O(1)
96.68(10)	O(4)-Os(1)-O(2)	97.25(11)
O(4)-Os(1)-N(1)	83.90(10)	O(4)-Os(1)-N(2)
82.42(10)	N(1)-Os(1)-N(2)	81.93(10)
C(1)-O(1)-Os(1)	110.59(18)	C(2)-O(2)-Os(1)
112.4(2)	C(12)-N(1)-Os(1)	104.79(17)
C(15)-N(1)-Os(1)	113.59(19)	C(15)-N(1)-C(12)
110.1(2)	C(16)-N(1)-Os(1)	111.06(19)
C(16)-N(1)-C(12)	109.2(2)	C(16)-N(1)-C(15)
108.0(2)	C(14)-N(2)-Os(1)	105.85(19)
C(14)-N(2)-C(17)	109.6(2)	C(14)-N(2)-C(18)
111.0(3)	C(17)-N(2)-Os(1)	111.4(2)
C(18)-N(2)-Os(1)	111.89(18)	C(18)-N(2)-C(17)
107.0(3)	O(1)-C(1)-H(1)	108.8
O(1)-C(1)-C(2)	108.5(2)	O(1)-C(1)-C(6)
108.1(2)	C(2)-C(1)-H(1)	108.8
C(2)-C(1)-C(6)	113.7(2)	C(6)-C(1)-H(1)
108.8	O(2)-C(2)-C(1)	107.3(2)
O(2)-C(2)-C(3)	107.2(3)	O(2)-C(2)-C(7)
108.6(2)	C(1)-C(2)-C(3)	113.1(2)
C(1)-C(2)-C(7)	110.8(3)	C(7)-C(2)-C(3)
109.8(3)	C(2)-C(3)-H(3A)	108.4

C(2)-C(3)-H(3B)	108.4	C(2)-C(3)-C(4)
115.3(3)	H(3A)-C(3)-H(3B)	107.5
C(4)-C(3)-H(3A)	108.4	C(4)-C(3)-H(3B)
108.4	C(3)-C(4)-H(4)	108.1
C(3)-C(4)-C(5)	109.6(3)	C(5)-C(4)-H(4)
108.1	C(8)-C(4)-C(3)	109.3(3)
C(8)-C(4)-H(4)	108.1	C(8)-C(4)-C(5)
113.5(3)	C(6)-C(5)-C(4)	107.6(2)
C(6)-C(5)-C(10)	109.3(3)	C(9)-C(5)-C(4)
113.0(3)	C(9)-C(5)-C(6)	110.8(3)
C(9)-C(5)-C(10)	110.0(4)	C(10)-C(5)-C(4)
106.0(4)	C(1)-C(6)-C(5)	114.6(3)
C(1)-C(6)-H(6A)	108.6	C(1)-C(6)-H(6B)
108.6	C(5)-C(6)-H(6A)	108.6
C(5)-C(6)-H(6B)	108.6	H(6A)-C(6)-H(6B)
107.6	C(2)-C(7)-H(7A)	109.5
C(2)-C(7)-H(7B)	109.5	C(2)-C(7)-H(7C)
109.5	H(7A)-C(7)-H(7B)	109.5
H(7A)-C(7)-H(7C)	109.5	H(7B)-C(7)-H(7C)
109.5	C(4)-C(8)-H(8A)	109.5
C(4)-C(8)-H(8B)	109.5	C(4)-C(8)-H(8C)
109.5	H(8A)-C(8)-H(8B)	109.5
H(8A)-C(8)-H(8C)	109.5	H(8B)-C(8)-H(8C)
109.5	C(5)-C(9)-H(9A)	109.5
C(5)-C(9)-H(9B)	109.5	C(5)-C(9)-H(9C)
109.5	H(9A)-C(9)-H(9B)	109.5
H(9A)-C(9)-H(9C)	109.5	H(9B)-C(9)-H(9C)
109.5	O(5)-C(10)-C(5)	119.5(7)
O(5)-C(10)-C(11)	120.5(6)	C(11)-C(10)-C(5)
120.0(4)	C(10)-C(11)-H(11A)	109.5
C(10)-C(11)-H(11B)	109.5	C(10)-C(11)-H(11C)
109.5	H(11A)-C(11)-H(11B)	109.5
H(11A)-C(11)-H(11C)	109.5	H(11B)-C(11)-H(11C)
109.5	N(1)-C(12)-H(12A)	109.6
N(1)-C(12)-H(12B)	109.6	N(1)-C(12)-C(14)
110.4(2)	H(12A)-C(12)-H(12B)	108.1

C(14)-C(12)-H(12A)	109.6	C(14)-C(12)-H(12B)
109.6	N(2)-C(14)-C(12)	110.7(2)
N(2)-C(14)-H(14A)	109.5	N(2)-C(14)-H(14B)
109.5	C(12)-C(14)-H(14A)	109.5
C(12)-C(14)-H(14B)	109.5	H(14A)-C(14)-H(14B)
108.1	N(1)-C(15)-H(15A)	109.5
N(1)-C(15)-H(15B)	109.5	N(1)-C(15)-H(15C)
109.5	H(15A)-C(15)-H(15B)	109.5
H(15A)-C(15)-H(15C)	109.5	H(15B)-C(15)-H(15C)
109.5	N(1)-C(16)-H(16A)	109.5
N(1)-C(16)-H(16B)	109.5	N(1)-C(16)-H(16C)
109.5	H(16A)-C(16)-H(16B)	109.5
H(16A)-C(16)-H(16C)	109.5	H(16B)-C(16)-H(16C)
109.5	N(2)-C(17)-H(17A)	109.5
N(2)-C(17)-H(17B)	109.5	N(2)-C(17)-H(17C)
109.5	H(17A)-C(17)-H(17B)	109.5
H(17A)-C(17)-H(17C)	109.5	H(17B)-C(17)-H(17C)
109.5	N(2)-C(18)-H(18A)	109.5
N(2)-C(18)-H(18B)	109.5	N(2)-C(18)-H(18C)
109.5	H(18A)-C(18)-H(18B)	109.5
H(18A)-C(18)-H(18C)	109.5	H(18B)-C(18)-H(18C)
109.5		

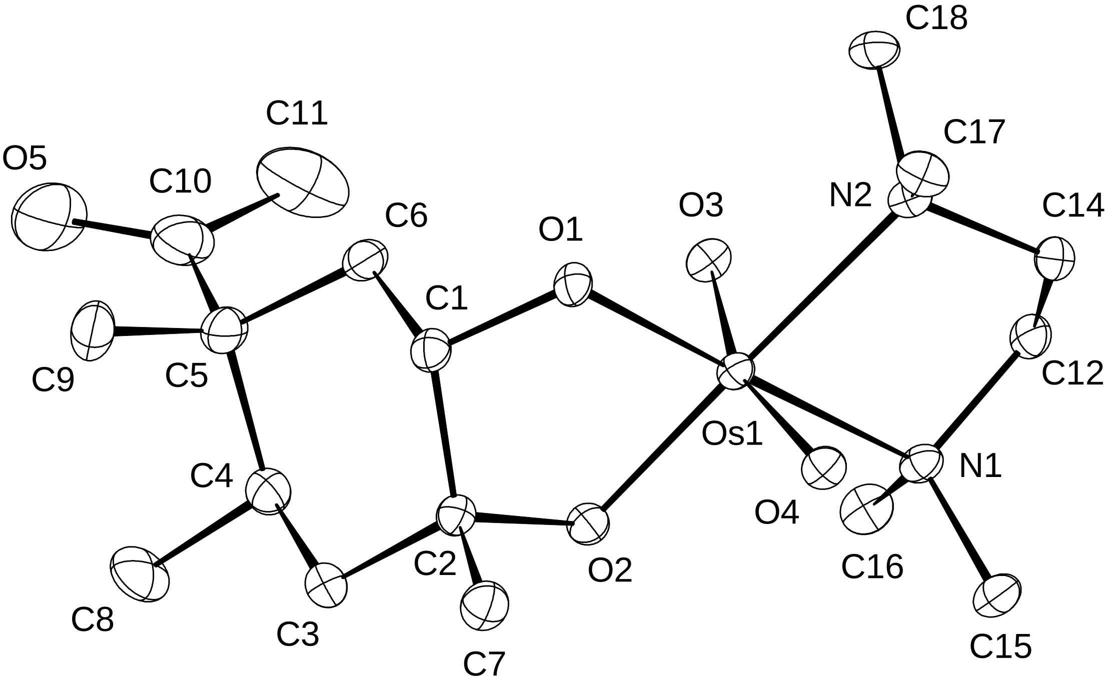

Figure S18. Structure of **4qa** (hydrogens removed for clarity)

14.2.2. Analysis of 4ra

Table S17. Crystal data and structure refinement. CCDC: 2472883

Identification code	16239	
Empirical formula	$C_{18} H_{36} N_2 O_5 Os$	
Color	yellow	
Formula weight	550.69 g·mol^{-1}	
Temperature	100(2) K	
Wavelength	0.71073 Å	
Crystal system	Monoclinic	
Space group	$P2_1$, (no. 4)	
Unit cell dimensions	a = 8.5320(7) Å	α= 90°.
	b = 12.0721(10) Å	β= 103.863(3)°.
	c = 10.6708(9) Å	γ = 90°.
Volume	1067.07(15) Å^3	
Z	2	
Density (calculated)	1.714 Mg·m^{-3}	
Absorption coefficient	6.004 mm^{-1}	
F(000)	548 e	
Crystal size	0.151 x 0.142 x 0.02 mm^3	
θ range for data collection	1.966 to 31.943°.	
Index ranges	$-12 \le h \le 12$, $-17 \le k \le 17$, $-15 \le l \le 15$	
Reflections collected	36553	
Independent reflections	7319 [R_{int} = 0.0354]	
Reflections with $I>2\sigma(I)$	6862	
Completeness to θ = 25.242°	100.0 %	
Absorption correction	Gaussian	
Max. and min. transmission	0.89087 and 0.57702	
Refinement method	Full-matrix least-squares on F^2	
Data / restraints / parameters	7319 / 73 / 303	
Goodness-of-fit on F^2	1.004	
Final R indices [$I>2\sigma(I)$]	R_1 = 0.0183	wR^2 = 0.0331
R indices (all data)	R_1 = 0.0211	wR^2 = 0.0337
Absolute structure parameter	0.011(4)	
Extinction coefficient	n/a	
Largest diff. peak and hole	1.022 and -1.142 e·Å^{-3}	

Table S18. Bond lengths [Å] and angles [°].

Os(1)-O(1)	1.941(2)	Os(1)-O(2)
1.945(2)	Os(1)-O(3)	1.741(2)
Os(1)-O(4)	1.746(2)	Os(1)-N(1)
2.232(3)	Os(1)-N(2)	2.231(3)
O(1)-C(1)	1.456(3)	O(2)-C(6)
1.446(4)	O(5)-C(9)	1.215(4)
N(1)-C(13A)	1.473(6)	N(1)-C(15A)
1.529(6)	N(1)-C(16A)	1.444(7)
N(1)-C(13B)	1.592(11)	N(1)-C(15B)
1.435(12)	N(1)-C(16B)	1.526(13)
N(2)-C(14A)	1.464(6)	N(2)-C(17A)
1.444(6)	N(2)-C(18A)	1.536(7)
N(2)-C(14B)	1.506(11)	N(2)-C(17B)
1.612(15)	N(2)-C(18B)	1.426(13)
C(1)-C(2)	1.542(4)	C(1)-C(6)
1.539(4)	C(1)-C(7)	1.525(4)
C(2)-H(2A)	0.9900	C(2)-H(2B)
0.9900	C(2)-C(3)	1.539(4)
C(3)-C(4)	1.550(4)	C(3)-C(8)
1.540(4)	C(3)-C(9)	1.532(4)
C(4)-H(4)	1.0000	C(4)-C(5)
1.519(4)	C(4)-C(11)	1.529(4)
C(5)-H(5A)	0.9900	C(5)-H(5B)
0.9900	C(5)-C(6)	1.524(5)
C(6)-C(12)	1.527(5)	C(7)-H(7A)
0.9800	C(7)-H(7B)	0.9800
C(7)-H(7C)	0.9800	C(8)-H(8A)
0.9800	C(8)-H(8B)	0.9800
C(8)-H(8C)	0.9800	C(9)-C(10)
1.509(4)	C(10)-H(10A)	0.9800
C(10)-H(10B)	0.9800	C(10)-H(10C)
0.9800	C(11)-H(11A)	0.9800
C(11)-H(11B)	0.9800	C(11)-H(11C)
0.9800	C(12)-H(12A)	0.9800

C(12)-H(12B)	0.9800	C(12)-H(12C)
0.9800	C(13A)-H(13A)	0.9900
C(13A)-H(13B)	0.9900	C(13A)-C(14A)
1.540(9)	C(14A)-H(14A)	0.9900
C(14A)-H(14B)	0.9900	C(15A)-H(15A)
0.9800	C(15A)-H(15B)	0.9800
C(15A)-H(15C)	0.9800	C(16A)-H(16A)
0.9800	C(16A)-H(16B)	0.9800
C(16A)-H(16C)	0.9800	C(17A)-H(17A)
0.9800	C(17A)-H(17B)	0.9800
C(17A)-H(17C)	0.9800	C(18A)-H(18A)
0.9800	C(18A)-H(18B)	0.9800
C(18A)-H(18C)	0.9800	C(13B)-H(13C)
0.9900	C(13B)-H(13D)	0.9900
C(13B)-C(14B)	1.471(16)	C(14B)-H(14C)
0.9900	C(14B)-H(14D)	0.9900
C(15B)-H(15D)	0.9800	C(15B)-H(15E)
0.9800	C(15B)-H(15F)	0.9800
C(16B)-H(16D)	0.9800	C(16B)-H(16E)
0.9800	C(16B)-H(16F)	0.9800
C(17B)-H(17D)	0.9800	C(17B)-H(17E)
0.9800	C(17B)-H(17F)	0.9800
C(18B)-H(18D)	0.9800	C(18B)-H(18E)
0.9800	C(18B)-H(18F)	0.9800

O(1)-Os(1)-O(2)	84.15(8)	O(1)-Os(1)-N(1)
96.80(9)	O(1)-Os(1)-N(2)	178.48(9)
O(2)-Os(1)-N(1)	179.04(9)	O(2)-Os(1)-N(2)
97.05(10)	O(3)-Os(1)-O(1)	97.97(10)
O(3)-Os(1)-O(2)	96.89(10)	O(3)-Os(1)-O(4)
160.28(11)	O(3)-Os(1)-N(1)	82.84(12)
O(3)-Os(1)-N(2)	82.84(11)	O(4)-Os(1)-O(1)
96.66(10)	O(4)-Os(1)-O(2)	97.69(10)
O(4)-Os(1)-N(1)	82.36(11)	O(4)-Os(1)-N(2)
82.26(11)	N(2)-Os(1)-N(1)	82.01(10)
C(1)-O(1)-Os(1)	112.30(16)	C(6)-O(2)-Os(1)

112.25(18)	C(13A)-N(1)-Os(1)	104.9(3)
C(13A)-N(1)-C(15A)	109.1(4)	C(15A)-N(1)-Os(1)
108.7(3)	C(16A)-N(1)-Os(1)	113.1(3)
C(16A)-N(1)-C(13A)	113.8(4)	C(16A)-N(1)-C(15A)
107.0(4)	C(13B)-N(1)-Os(1)	105.4(4)
C(15B)-N(1)-Os(1)	117.9(5)	C(15B)-N(1)-C(13B)
108.4(7)	C(15B)-N(1)-C(16B)	107.1(7)
C(16B)-N(1)-Os(1)	114.3(5)	C(16B)-N(1)-C(13B)
102.6(7)	C(14A)-N(2)-Os(1)	105.0(3)
C(14A)-N(2)-C(18A)	109.3(4)	C(17A)-N(2)-Os(1)
112.6(3)	C(17A)-N(2)-C(14A)	111.4(4)
C(17A)-N(2)-C(18A)	107.4(4)	C(18A)-N(2)-Os(1)
111.0(3)	C(14B)-N(2)-Os(1)	106.2(4)
C(14B)-N(2)-C(17B)	104.9(7)	C(17B)-N(2)-Os(1)
109.4(6)	C(18B)-N(2)-Os(1)	112.9(6)
C(18B)-N(2)-C(14B)	115.5(7)	C(18B)-N(2)-C(17B)
107.5(8)	O(1)-C(1)-C(2)	107.0(2)
O(1)-C(1)-C(6)	106.9(2)	O(1)-C(1)-C(7)
105.1(2)	C(6)-C(1)-C(2)	111.0(2)
C(7)-C(1)-C(2)	112.3(3)	C(7)-C(1)-C(6)
114.0(3)	C(1)-C(2)-H(2A)	107.8
C(1)-C(2)-H(2B)	107.8	H(2A)-C(2)-H(2B)
107.1	C(3)-C(2)-C(1)	118.1(2)
C(3)-C(2)-H(2A)	107.8	C(3)-C(2)-H(2B)
107.8	C(2)-C(3)-C(4)	109.3(2)
C(2)-C(3)-C(8)	112.5(2)	C(8)-C(3)-C(4)
111.7(2)	C(9)-C(3)-C(2)	107.8(2)
C(9)-C(3)-C(4)	107.4(2)	C(9)-C(3)-C(8)
107.9(2)	C(3)-C(4)-H(4)	107.7
C(5)-C(4)-C(3)	110.3(2)	C(5)-C(4)-H(4)
107.7	C(5)-C(4)-C(11)	110.5(3)
C(11)-C(4)-C(3)	112.7(3)	C(11)-C(4)-H(4)
107.7	C(4)-C(5)-H(5A)	108.5
C(4)-C(5)-H(5B)	108.5	C(4)-C(5)-C(6)
114.9(2)	H(5A)-C(5)-H(5B)	107.5
C(6)-C(5)-H(5A)	108.5	C(6)-C(5)-H(5B)

108.5	O(2)-C(6)-C(1)	106.6(2)
O(2)-C(6)-C(5)	107.0(2)	O(2)-C(6)-C(12)
108.5(3)	C(5)-C(6)-C(1)	112.0(3)
C(5)-C(6)-C(12)	109.6(3)	C(12)-C(6)-C(1)
112.9(3)	C(1)-C(7)-H(7A)	109.5
C(1)-C(7)-H(7B)	109.5	C(1)-C(7)-H(7C)
109.5	H(7A)-C(7)-H(7B)	109.5
H(7A)-C(7)-H(7C)	109.5	H(7B)-C(7)-H(7C)
109.5	C(3)-C(8)-H(8A)	109.5
C(3)-C(8)-H(8B)	109.5	C(3)-C(8)-H(8C)
109.5	H(8A)-C(8)-H(8B)	109.5
H(8A)-C(8)-H(8C)	109.5	H(8B)-C(8)-H(8C)
109.5	O(5)-C(9)-C(3)	120.9(3)
O(5)-C(9)-C(10)	119.3(3)	C(10)-C(9)-C(3)
119.8(3)	C(9)-C(10)-H(10A)	109.5
C(9)-C(10)-H(10B)	109.5	C(9)-C(10)-H(10C)
109.5	H(10A)-C(10)-H(10B)	109.5
H(10A)-C(10)-H(10C)	109.5	H(10B)-C(10)-H(10C)
109.5	C(4)-C(11)-H(11A)	109.5
C(4)-C(11)-H(11B)	109.5	C(4)-C(11)-H(11C)
109.5	H(11A)-C(11)-H(11B)	109.5
H(11A)-C(11)-H(11C)	109.5	H(11B)-C(11)-H(11C)
109.5	C(6)-C(12)-H(12A)	109.5
C(6)-C(12)-H(12B)	109.5	C(6)-C(12)-H(12C)
109.5	H(12A)-C(12)-H(12B)	109.5
H(12A)-C(12)-H(12C)	109.5	H(12B)-C(12)-H(12C)
109.5	N(1)-C(13A)-H(13A)	109.8
N(1)-C(13A)-H(13B)	109.8	N(1)-C(13A)-C(14A)
109.5(5)	H(13A)-C(13A)-H(13B)	108.2
C(14A)-C(13A)-H(13A)	109.8	C(14A)-C(13A)-H(13B)
109.8	N(2)-C(14A)-C(13A)	112.1(5)
N(2)-C(14A)-H(14A)	109.2	N(2)-C(14A)-H(14B)
109.2	C(13A)-C(14A)-H(14A)	109.2
C(13A)-C(14A)-H(14B)	109.2	H(14A)-C(14A)-H(14B)
107.9	N(1)-C(15A)-H(15A)	109.5
N(1)-C(15A)-H(15B)	109.5	N(1)-C(15A)-H(15C)

109.5	H(15A)-C(15A)-H(15B)	109.5
H(15A)-C(15A)-H(15C)	109.5	H(15B)-C(15A)-H(15C)
109.5	N(1)-C(16A)-H(16A)	109.5
N(1)-C(16A)-H(16B)	109.5	N(1)-C(16A)-H(16C)
109.5	H(16A)-C(16A)-H(16B)	109.5
H(16A)-C(16A)-H(16C)	109.5	H(16B)-C(16A)-H(16C)
109.5	N(2)-C(17A)-H(17A)	109.5
N(2)-C(17A)-H(17B)	109.5	N(2)-C(17A)-H(17C)
109.5	H(17A)-C(17A)-H(17B)	109.5
H(17A)-C(17A)-H(17C)	109.5	H(17B)-C(17A)-H(17C)
109.5	N(2)-C(18A)-H(18A)	109.5
N(2)-C(18A)-H(18B)	109.5	N(2)-C(18A)-H(18C)
109.5	H(18A)-C(18A)-H(18B)	109.5
H(18A)-C(18A)-H(18C)	109.5	H(18B)-C(18A)-H(18C)
109.5	N(1)-C(13B)-H(13C)	110.1
N(1)-C(13B)-H(13D)	110.1	H(13C)-C(13B)-H(13D)
108.5	C(14B)-C(13B)-N(1)	107.8(8)
C(14B)-C(13B)-H(13C)	110.1	C(14B)-C(13B)-H(13D)
110.1	N(2)-C(14B)-H(14C)	108.6
N(2)-C(14B)-H(14D)	108.6	C(13B)-C(14B)-N(2)
114.7(8)	C(13B)-C(14B)-H(14C)	108.6
C(13B)-C(14B)-H(14D)	108.6	H(14C)-C(14B)-H(14D)
107.6	N(1)-C(15B)-H(15D)	109.5
N(1)-C(15B)-H(15E)	109.5	N(1)-C(15B)-H(15F)
109.5	H(15D)-C(15B)-H(15E)	109.5
H(15D)-C(15B)-H(15F)	109.5	H(15E)-C(15B)-H(15F)
109.5	N(1)-C(16B)-H(16D)	109.5
N(1)-C(16B)-H(16E)	109.5	N(1)-C(16B)-H(16F)
109.5	H(16D)-C(16B)-H(16E)	109.5
H(16D)-C(16B)-H(16F)	109.5	H(16E)-C(16B)-H(16F)
109.5	N(2)-C(17B)-H(17D)	109.5
N(2)-C(17B)-H(17E)	109.5	N(2)-C(17B)-H(17F)
109.5	H(17D)-C(17B)-H(17E)	109.5
H(17D)-C(17B)-H(17F)	109.5	H(17E)-C(17B)-H(17F)
109.5	N(2)-C(18B)-H(18D)	109.5
N(2)-C(18B)-H(18E)	109.5	N(2)-C(18B)-H(18F)

109.5	H(18D)-C(18B)-H(18E)	109.5
H(18D)-C(18B)-H(18F)	109.5	H(18E)-C(18B)-H(18F)
109.5		

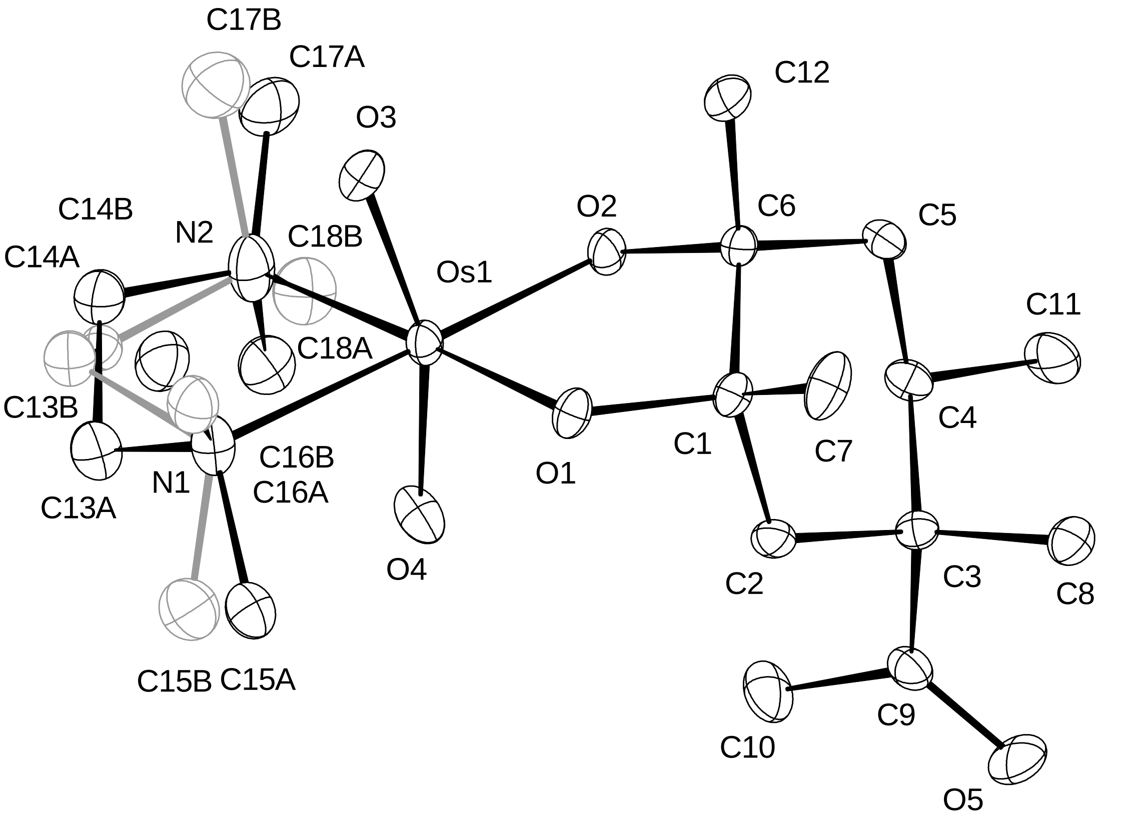

Figure S19. Structure of **4ra** The H atoms have been omitted for clarity, and the minor disorder is shown in grey.

15. Intramolecular Competition Kinetic Isotope Effect (KIE) Experiments

15.1. Introduction to Experimental Mechanistic Investigation of Diels-Alder Cycloaddition

The studies were performed using **4r** as a model compound, which was obtained with high enantioselectivity from the cycloaddition of symmetrical diene **3d** and model enone **2a**.

15.2. NMR assignment data of compound 4r in CD_2Cl_2

Table S19. The assignment of ^{1}H and ^{13}C NMR chemical shifts to the structure of **4r**.

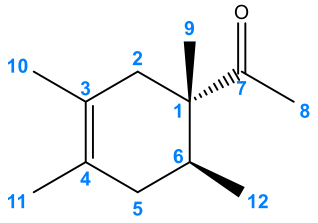

4r

Atom	δ (ppm)	T_1(s)	HSQC	COSY	HMBC
**1 C**	51.33	33.6			5', 5", 6, 8, 9, 12
**2 C**	41.22	3.3	2', 2"		6, 9, 10
**H'**	1.634		2	2", 10	3, 4, 7, 9, 10
**H''**	2.338		2	2', 10	3, 4, 9
**3 C**	123.27	16.1			2', 2", 5', 5", 10, 11
**4 C**	124.43	16.3			2', 2", 5', 5", 6, 10, 11
**5 C**	37.92	3.2	5', 5"		6, 11, 12
**H'**	1.634		5	6, 11	1, 3, 4, 12
**H''**	1.953		5	6, 11	1, 3, 4, 12
**6 C**	33.45	5.7	6		9, 12
**H**	2.109		6	5', 5", 12	1, 2, 4, 5, 9, 12
**7 C**	213.80	21.0			2', 8, 9
**8 C**	25.40	10.8	8		
**H3**	2.088		8		1, 7
**9 C**	15.86	3.3	9		2', 2", 6
**H3**	0.935		9		1, 2, 6, 7
**10 C**	19.14	10.6	10		2'
**H3**	1.596		10	2', 2"	2, 3, 4
**11 C**	18.85	10.1	11		
**H3**	1.578		11	5', 5"	3, 4, 5
**12 C**	16.37	3.6	12		5', 5", 6
**H3**	0.772		12	6	1, 5, 6

^{13}C relaxation times were determined using an inversion-recovery NMR pulse sequence.

15.3. NMR data acquisition and processing methods

NMR data acquisition

Quantitative ^{13}C NMR spectra were acquired on Bruker AvanceNeo 600 MHz NMR spectrometer at 298K equipped with a cryogenically-cooled BBO probe using an inverse-gated decoupling ^{13}C NMR sequence after a pi/6 pulse (^{13}C pulse length 3.33 µs; bruker pulse sequence: zgig30) with a pulse offset at 100 ppm and an acquisition time of 2.2437828 s. The pulse is be considered quantitative (99.6% uniform excitation) ± 100 ppm around the offset. The raw FID contained 178078 complex data points with a dwell time of 12.6 µs (spectral with: 262.9132 ppm) and for a spectrum 64-128 FIDs were averaged. A relaxation delay time (d1; time between scans) of 120 s was used (~7.4 times of T_1(C-4); longest T_1 of carbons of interest).

NMR data processing

After acquisition the FIDs were zero filled to 512k data points and Fourier transformed with an EM window function (lb = 0.3 Hz) in Bruker Topspin 4.0.6. In order to remove the baseline curvature from the cryoprobe data, the resulting spectra were phased and baseline corrected with the Bruker Deep-learning based phase and baseline correction command *apbk*. (S. Bruderer, F. Paruzzo, C. Bolliger; Deep learning-based phase and baseline correction of 1D 1H NMR Spectra; Bruker Application notes, link: https://www.bruker.com/content/dam/bruker/int/en/resources/bbio/magnetic-resonance/application-notes/T186209_Bruker%20Whitepaper%20Deep%20Learning%20in%20NMR.pdf).

The resulting spectra were visually checked and the processed spectra was then imported into Mestrelab MNOVA. The imported spectra of each sample were stacked and the signals of interest (C-2, C-3, C-4, C-5, C-10, C-11) were integrated ±0.05 ppm around the peak maxima.

15.4. Sample Preparation

15.4.1. The Synthetic procedures:

Thermal Diels-Alder Cycloaddition:

A flame-dried pressure vessel equipped with a magnetic stirring bar was charged under an argon atmosphere with **2a** (5 mmol, 1.0 equiv., 0.545 mL) and **3d** (25 mmol, 5.0 equiv., 2.82 mL). The vessel was sealed thoroughly to prevent the loss of volatile starting materials and heated overnight at 145 °C. The crude reaction mixture was directly purified by silica gel column chromatography, initially eluting with pentane to remove unreacted starting materials and oligomers, followed by 3% Et_2O in pentane to elute the product. Product-containing fractions were concentrated under reduced pressure, and the crude material was further purified by distillation (T = 140 °C, p = 5 mbar) to yield pure **4r-1** as a colorless oil (189 mg, 21% yield). Further upscaling of this protocol resulted in poor yields; therefore, the reaction was performed in parallel batches to obtain a larger quantity of the compound.

The aluminum chloride catalyzed Diels-Alder cycloaddition:

The compound **4r-2** was synthesized according to the Alumina Chloride Catalyzed Diels-Alder Reaction (See section 4.1) from **2a** (5 mmol, 1 equiv., 0.545 mL) and **3d** (12.5 mmol, 2.5 equiv., 1.41 mL) as a colorless oil (577 mg, 64% yield).

The IDPi-5c catalyzed Diels-Alder cycloaddition:

The compound **4r-3** was synthesized according to the procedure for the upscaling of synthesis of **4a** (See section 5) with **IDPi-5c** as a catalyst, using **2a** (1 mmol, 1 equiv., 109 µL) and **3d** (4 mmol, 4 equiv., 452 µL). The purified product was obtained as a colorless oil (148 mg, 82% yield, 52:48 e.r.).

Diels-Alder IDPi-5h catalyzed cycloaddition:

The compound **4r-4** was synthesized according to the procedure for the upscaling of synthesis of **4a** (See section 5) using **2a** (1 mmol, 1 equiv., 109 µL) and **3d** (4 mmol, 4 equiv., 452 µL). The purified product was obtained as a colorless oil (160 mg, 89% yield, 95:5 e.r.).

15.4.2. The preparation of NMR samples:

The given mass of compound **4r** (at least 80 mg) was dissolved in 0.3 mL of DCM-d_2 and placed into Wilmad 15'' 5 mm Thin Wall glass NMR tube under atmosphere of air. Then, tube was closed and the lower part was put into dry-ice ethanol bath and cooled down to decrease the pressure inside before the tube was flame-sealed for NMR measurements.

Sample 1: Prepared from compound **4r-1** (228 mg).

Sample 2: Prepared from compound **4r-2** (180 mg)

Sample 3: Prepared from compound **4r-3** (119 mg)

Sample 4: Prepared from compound **4r-4** (84 mg)

15.5. NMR Integrals and KIE Results

15.5.1. Overview of obtained KIE data

Scheme S1. General equation of reaction leading to the **4r**.

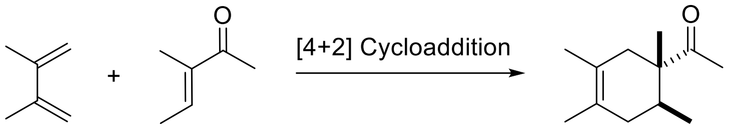

Table S20. The Results of the KIE experiments: KIE values with error value.

catalyst	Relative ^{13}C KIE					
	C2	C3	C4	C5	C10	C11
$AlCl_3$	1.000 ± 0.000	1.000 ± 0.000	1.002 ± 0.001	1.024 ± 0.002	1.000 ± 0.000	0.999 ± 0.002
Thermal, 145 °C	1.000 ± 0.000	1.000 ± 0.000	1.003 ± 0.000	1.019 ± 0.001	1.000 ± 0.000	0.996 ± 0.001
5c	1.000 ± 0.000	1.000 ± 0.000	1.003 ± 0.001	1.030 ± 0.002	1.000 ± 0.000	1.001 ± 0.002
5h	1.000 ± 0.000	1.000 ± 0.000	1.002 ± 0.003	1.031 ± 0.002	1.000 ± 0.000	0.999 ± 0.004

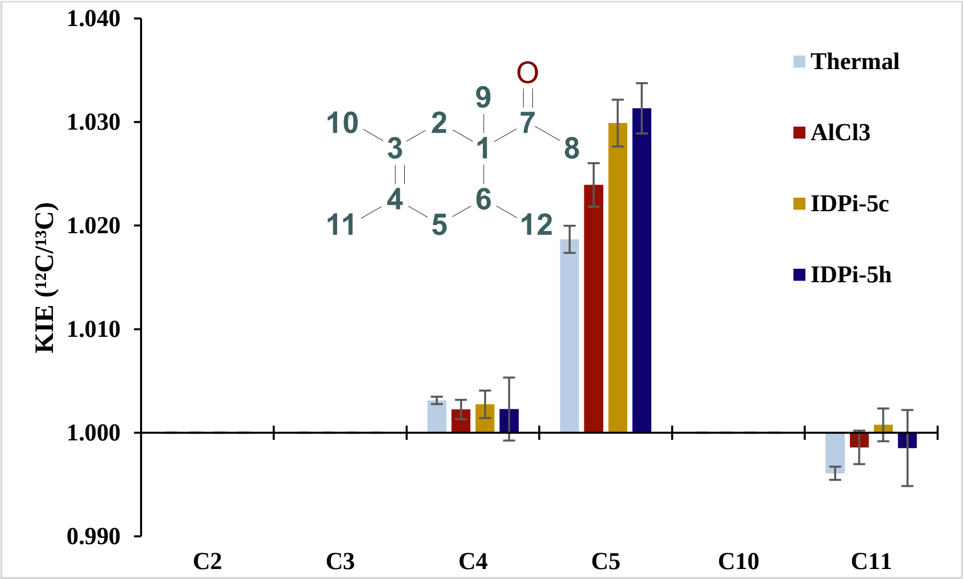

Figure S20. Intramolecular competitive ^{13}C kinetic isotope effect under standard reaction conditions. Values are given for different reaction conditions (see SI for further details). Error bars in the graph in indicate the standard derivation of six NMR measurements of the same sample.

15.5.2. Sample 1 – Thermal Reaction (4r-1)

Table S21. Overview of obtained NMR integrals for sample 1 from 6 individual measurements and data derived from those measurements.

Integrals (a.u. × 10^{-12})						
**#**	**C2**	**C3**	**C4**	**C5**	**C10**	**C11**
1	5.1029	4.9604	4.9439	5.0165	5.2372	5.2601
2	5.1124	4.9621	4.9481	5.0260	5.2456	5.2621
3	5.1164	4.9531	4.9405	5.0150	5.2341	5.2583
4	5.1107	4.9695	4.9534	5.0192	5.2367	5.2601
5	5.1185	4.9665	4.9503	5.0206	5.2382	5.2575
6	5.1179	4.9719	4.9546	5.0192	5.2462	5.2633
**relative Integrals**						
**#**	**C2**	**C3**	**C4**	**C5**	**C10**	**C11**
1	1.0000	1.0000	0.9967	0.9831	1.0000	1.0044
2	1.0000	1.0000	0.9972	0.9831	1.0000	1.0032
3	1.0000	1.0000	0.9975	0.9802	1.0000	1.0046
4	1.0000	1.0000	0.9968	0.9821	1.0000	1.0045
5	1.0000	1.0000	0.9967	0.9809	1.0000	1.0037
6	1.0000	1.0000	0.9965	0.9807	1.0000	1.0033
av	1.000 ± 0.000	1.000 ± 0.000	0.997 ± 0.000	0.982 ± 0.001	1.000 ± 0.000	1.004 ± 0.001
**Relative ^{13}C KIE**						
**#**	**C2**	**C3**	**C4**	**C5**	**C10**	**C11**
1	1.000	1.000	1.003	1.017	1.000	0.996
2	1.000	1.000	1.003	1.017	1.000	0.997
3	1.000	1.000	1.003	1.020	1.000	0.995
4	1.000	1.000	1.003	1.018	1.000	0.996
5	1.000	1.000	1.003	1.020	1.000	0.996
6	1.000	1.000	1.003	1.020	1.000	0.997
av	**1.000 ± 0.000**	**1.000 ± 0.000**	**1.003 ± 0.000**	**1.019 ± 0.001**	**1.000 ± 0.000**	**0.996 ± 0.001**

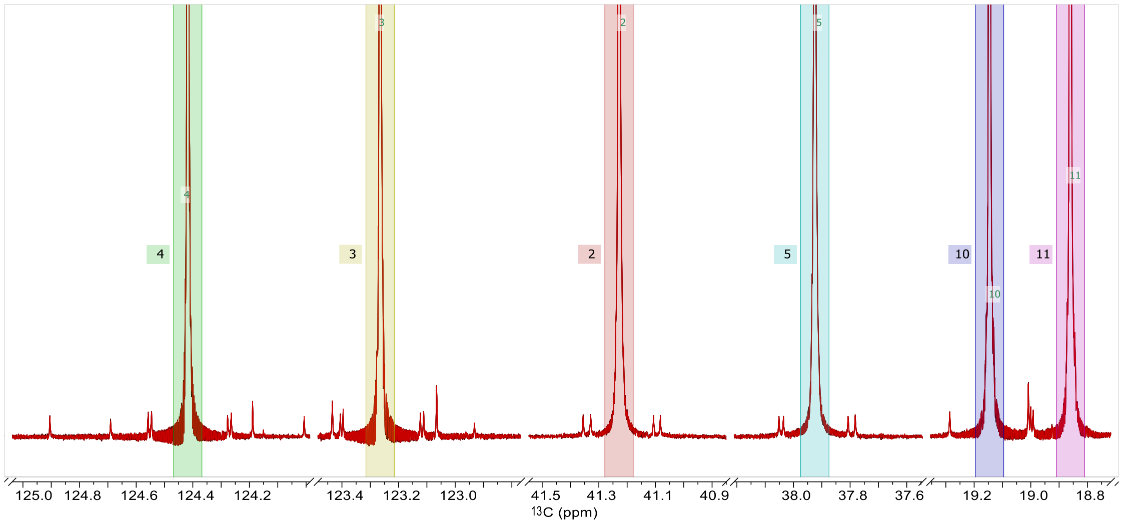

Figure S21: Stacked quantitative ^{13}C NMR spectra of sample 2 showing integration regions of signals of interest.

15.5.3. Sample 2 – $AlCl_3$-catalysed cycloaddition (4r-2)

Table S22. Overview of obtained NMR integrals for sample 2 from 6 individual measurements and data derived from those measurements.

Integrals (a.u. × 10^{-12})						
**#**	**C2**	**C3**	**C4**	**C5**	**C10**	**C11**
1	4.1881	4.0702	4.0616	4.1013	4.2767	4.2761
2	4.1998	4.0776	4.0703	4.1003	4.2851	4.2919
3	4.2059	4.0845	4.0769	4.1053	4.2890	4.2852
4	4.2057	4.0835	4.0744	4.0965	4.2915	4.3033
5	4.2127	4.0884	4.0825	4.1091	4.2894	4.2979
6	4.1995	4.0907	4.0742	4.1102	4.2836	4.2975
**relative Integrals**						
**#**	**C2**	**C3**	**C4**	**C5**	**C10**	**C11**
1	1.0000	1.0000	0.9979	0.9793	1.0000	0.9998
2	1.0000	1.0000	0.9982	0.9763	1.0000	1.0016
3	1.0000	1.0000	0.9982	0.9761	1.0000	0.9991
4	1.0000	1.0000	0.9978	0.9740	1.0000	1.0027
5	1.0000	1.0000	0.9986	0.9754	1.0000	1.0020
6	1.0000	1.0000	0.9960	0.9787	1.0000	1.0032
av	1.000 ± 0.000	1.000 ± 0.000	0.998 ± 0.001	0.977 ± 0.002	1.000 ± 0.000	1.001 ± 0.002
**Relative ^{13}C KIE**						
**#**	**C2**	**C3**	**C4**	**C5**	**C10**	**C11**
1	1.000	1.000	1.002	1.021	1.000	1.000
2	1.000	1.000	1.002	1.024	1.000	0.998
3	1.000	1.000	1.002	1.025	1.000	1.001
4	1.000	1.000	1.002	1.027	1.000	0.997
5	1.000	1.000	1.001	1.025	1.000	0.998
6	1.000	1.000	1.004	1.022	1.000	0.997
av	**1.000 ± 0.000**	**1.000 ± 0.000**	**1.002 ± 0.001**	**1.024 ± 0.002**	**1.000 ± 0.000**	**0.999 ± 0.002**

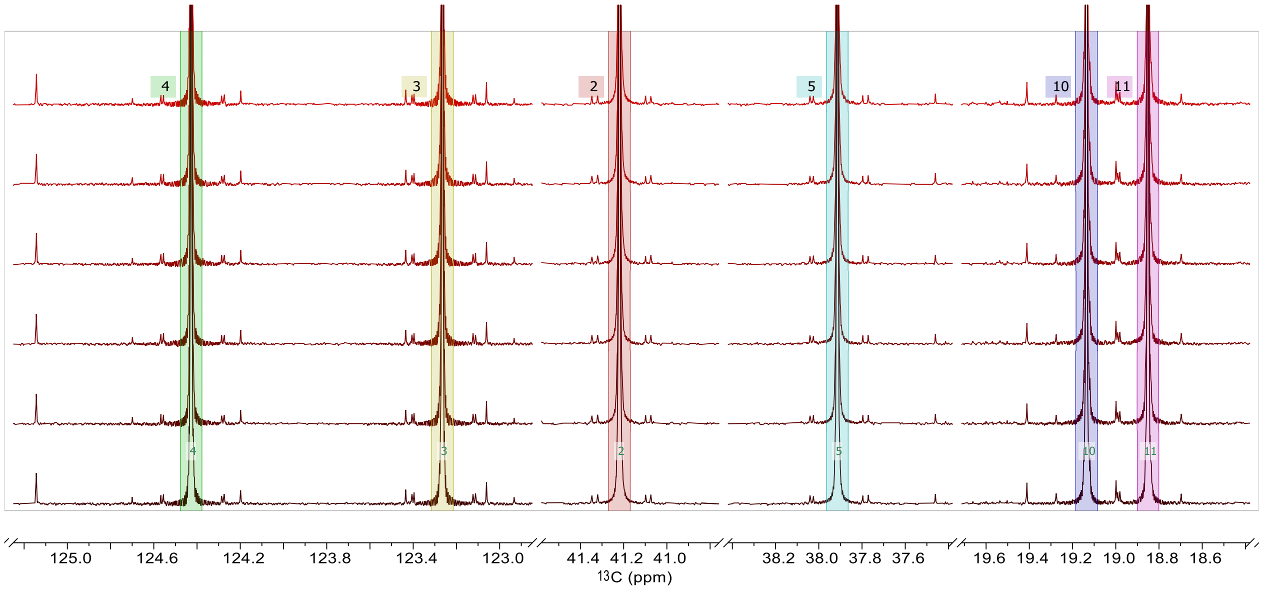

Figure S22. Stacked quantitative ^{13}C NMR spectra of sample 1 showing integration regions of signals of interest

15.5.4. Sample 3 – IDPi-5c catalyzed cycloaddition (4r-3)

Table S23. Overview of obtained NMR integrals for sample 3 from 6 individual measurements and data derived from those measurements.

Integrals (a.u. × 10^{-12})						
**#**	**C2**	**C3**	**C4**	**C5**	**C10**	**C11**
1	3.2672	3.1786	3.1725	3.1768	3.3389	3.3460
2	3.2789	3.1927	3.1821	3.1817	3.3557	3.3504
3	3.2858	3.2023	3.1903	3.1906	3.3549	3.3534
4	3.1149	2.9618	2.9485	3.0338	3.2135	3.2070
5	3.1215	2.9600	2.9568	3.0205	3.2198	3.2132
6	3.1284	2.9598	2.9544	3.0363	3.2130	3.2111
**relative Integrals**						
**#**	**C2**	**C3**	**C4**	**C5**	**C10**	**C11**
1	1.0000	1.0000	0.9981	0.9723	1.0000	1.0021
2	1.0000	1.0000	0.9967	0.9704	1.0000	0.9984
3	1.0000	1.0000	0.9962	0.9710	1.0000	0.9996
4	1.0000	1.0000	0.9955	0.9740	1.0000	0.9980
5	1.0000	1.0000	0.9989	0.9676	1.0000	0.9979
6	1.0000	1.0000	0.9982	0.9705	1.0000	0.9994
av	1.000 ± 0.000	1.000 ± 0.000	0.997 ± 0.001	0.971 ± 0.002	1.000 ± 0.000	0.999 ± 0.002
**Relative ^{13}C KIE**						
**#**	**C2**	**C3**	**C4**	**C5**	**C10**	**C11**
1	1.000	1.000	1.002	1.028	1.000	0.998
2	1.000	1.000	1.003	1.031	1.000	1.002
3	1.000	1.000	1.004	1.030	1.000	1.000
4	1.000	1.000	1.005	1.027	1.000	1.002
5	1.000	1.000	1.001	1.033	1.000	1.002
6	1.000	1.000	1.002	1.030	1.000	1.001
av	**1.000 ± 0.000**	**1.000 ± 0.000**	**1.003 ± 0.001**	**1.030 ± 0.002**	**1.000 ± 0.000**	**1.001 ± 0.002**

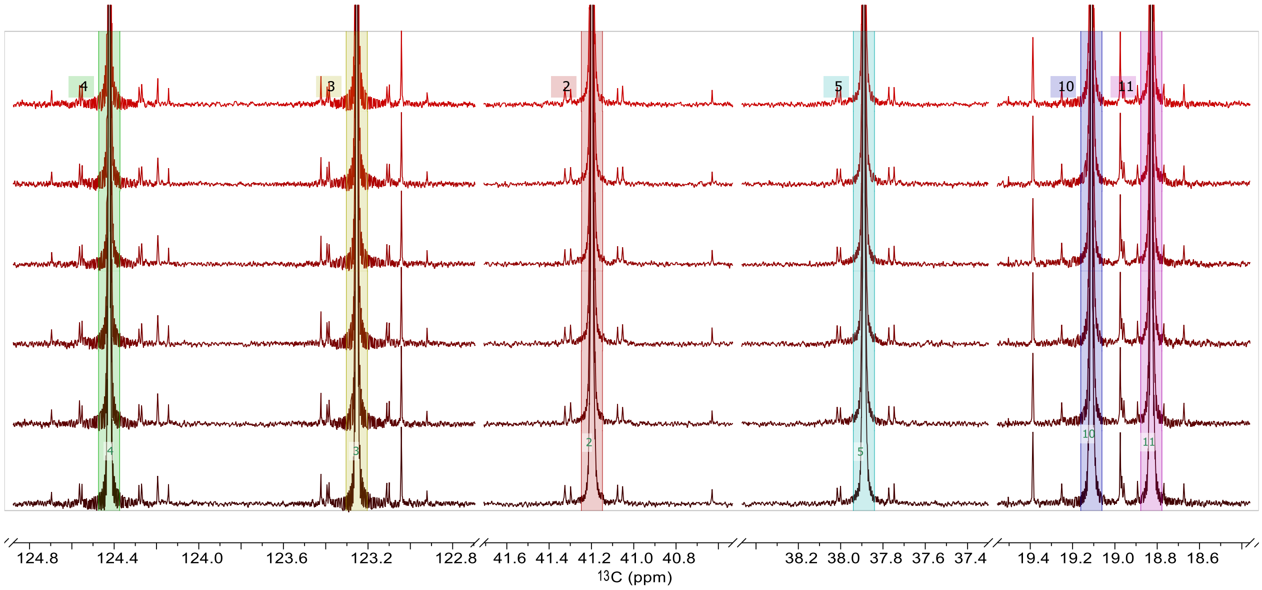

Figure S23. Stacked quantitative ^{13}C NMR spectra of sample 3 showing integration regions of signals of interest.

15.5.5. Sample 4 – IDPi-5h catalyzed cycloaddition (4r-4)

Table S24. Overview of obtained NMR integrals for sample 4 from 6 individual measurements and data derived from those measurements.

Integrals (a.u. × 10^{-12})						
**#**	**C2**	**C3**	**C4**	**C5**	**C10**	**C11**
1	1.7437	1.6901	1.6873	1.6939	1.7973	1.7941
2	1.7513	1.6914	1.6901	1.6964	1.7978	1.7949
3	1.7648	1.7073	1.6961	1.7044	1.7980	1.8007
4	1.7572	1.6968	1.6875	1.7085	1.7994	1.8134
5	1.7584	1.7010	1.7006	1.7049	1.8038	1.8102
6	1.7570	1.6964	1.6981	1.7043	1.8079	1.8069
**relative Integrals**						
**#**	**C2**	**C3**	**C4**	**C5**	**C10**	**C11**
1	1.0000	1.0000	0.9983	0.9715	1.0000	0.9982
2	1.0000	1.0000	0.9992	0.9687	1.0000	0.9984
3	1.0000	1.0000	0.9935	0.9658	1.0000	1.0015
4	1.0000	1.0000	0.9946	0.9723	1.0000	1.0078
5	1.0000	1.0000	0.9998	0.9696	1.0000	1.0035
6	1.0000	1.0000	1.0010	0.9700	1.0000	0.9994
av	1.000 ± 0.000	1.000 ± 0.000	0.998 ± 0.003	0.970 ± 0.002	1.000 ± 0.000	1.001 ± 0.004
**Relative ^{13}C KIE**						
**#**	**C2**	**C3**	**C4**	**C5**	**C10**	**C11**
1	1.000	1.000	1.002	1.029	1.000	1.002
2	1.000	1.000	1.001	1.032	1.000	1.002
3	1.000	1.000	1.007	1.035	1.000	0.998
4	1.000	1.000	1.005	1.029	1.000	0.992
5	1.000	1.000	1.000	1.031	1.000	0.996
6	1.000	1.000	0.999	1.031	1.000	1.001
av	**1.000 ± 0.000**	**1.000 ± 0.000**	**1.002 ± 0.003**	**1.031 ± 0.002**	**1.000 ± 0.000**	**0.999 ± 0.004**

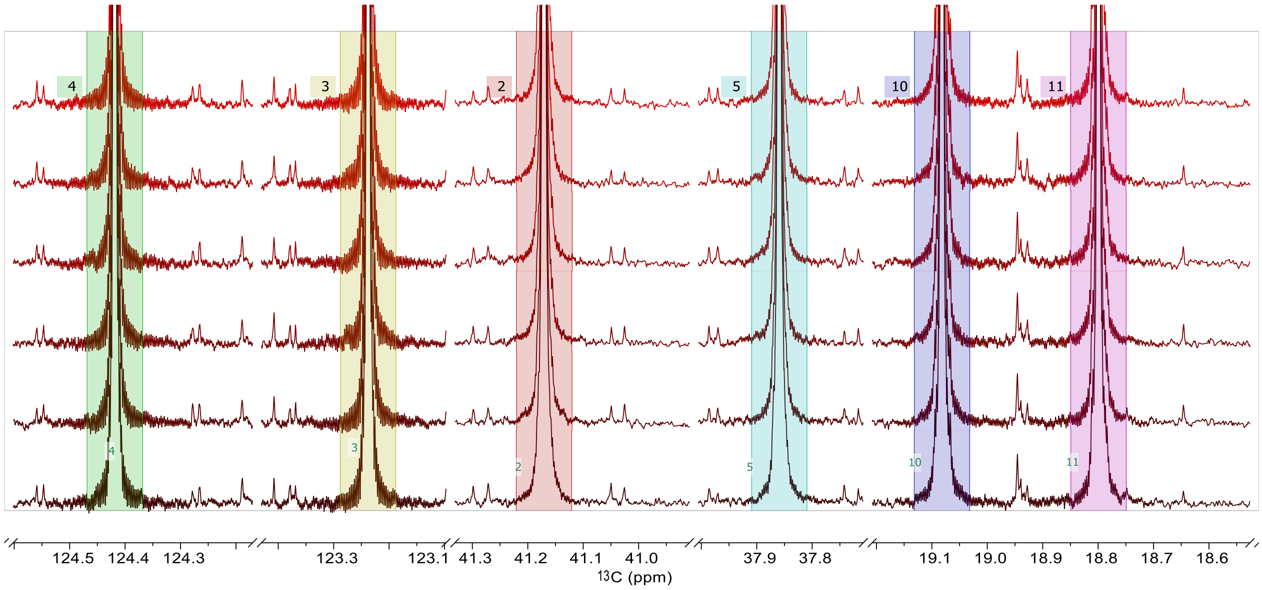

Figure S24. Stacked quantitative ^{13}C NMR spectra of sample 4 showing integration regions of signals of interest.

16. Computational studies

16.1. Method

Possible TS conformations were explored by the artificial force induced reaction (AFIR) method[35] implemented in the global route reaction mapping (GRRM) program.[36] An extensive conformational search has been performed on possible catalyst substrate orientations at GFN2-xTB level of theory[24] implemented in Orca 4.2.1[23], using SC-AFIR with constraint. Molecular geometries were optimized at r^2SCAN-3c[37] implemented in Orca 5.0.3 program.[38] In the case of transition state optimization, they were combined with GRRM program. Thermal free energy corrections have been performed at the same level of theory using Orca 5.0.3 program, and the temperature was set at 213.15K. Transition state structures were verified by the presence of a single imaginary vibrational frequency and the corresponding intrinsic reaction coordinates. Solvation effect has been accounted by using SMD ($CHCl_3$) solvation model as implemented in Orca 5.0.3 program. All single point energy is calculated at SMD($CHCl_3$)-ωB97M-V/def2-TZVPP level of theory.[29,39] RI approximation was used with RIJCOSX with def2/J as an auxiliary basis set implemented in Orca 5.0.3. IGMH analysis was performed using Multiwfn.[32,40] The wavefunction file was generated at ωB97XD/def2-TZVPP level of theory using Gaussian 16.[41] The visualizations of the molecular geometries were generated using the ChimeraX[42] version 1.9 followed by rendering with Blender version 4.0. Local Energy Decomposition (LED) analysis was performed at the DLPNO-CCSD(T)/def2-TZVP level with RIJCOSX and def2-TZVP/C, using the normal PNO setting.[42a] Conversion of enantiomeric ratio and ΔΔG was performed based on the Boltzmann distribution as follows: $\Delta\Delta G = RT\ln(\mathrm{pdt}(R)/\mathrm{pdt}(S))$.[43] IGMH analysis[40] was conducted with Multiwfn 3.8(dev).[32,44]

KIE values were obtained using PyQuiver program.[45]

16.2. Results and Discussion

The DFT calculation is performed using diene **3a**, ketone **2**, and catalyst **5h**. The calculated energy diagram and the proposed reaction mechanism are shown in Figure S25A. Intermediates **I** and **II** correspond to the reactants and products, respectively, connected by the IRC pathway from each transition state. The calculated results at SMD($CHCl_3$)-ωB97M-V/def2-TZVPP//r^2SCAN-3c level of theory suggest that the C-C bond forming **TS** is the enantio-determining step, regardless of the observed stereoselectivity. For all $\mathbf{TS_{maj}}$, $\mathbf{TS_{min}}$, and $\mathbf{TS_{reg}}$, a few negative vibrational modes were

observed; however, each transition state was confirmed by a single distinct imaginary frequency corresponding to the reaction coordinate, as well as by IRC analysis. The additional low-magnitude negative frequencies (all > -50 cm^{-1}) are likely numerical artifacts and do not correspond to meaningful reaction coordinates. The calculated energy difference between **TS_{maj}** and **TS_{min}** agreed well with the experimental one (1.0 kcal/mol (calc) vs. 1.0 kcal/mol (exp)). Furthermore, the energy difference between **TS_{maj}** and **TS_{reg}** also agrees well with experimental data (1.7 kcal/mol calculated vs. >1.2 kcal/mol experimental, Figure S25B).

Initially, our study focused on the s-trans ketone due to its intrinsic preference for the s-trans conformation (4.4 kcal/mol lower in energy). Here, we have additionally considered the possibility of s-cis conformations. Namely, conformer sampling of the s-cis isomer based on TSmaj and geometry optimizations with fixed forming bond lengths were performed. At the r^2SCAN-3c level of theory, none of these structures were lower in energy than TSmaj. Although they are not true transition states, the small energy difference (0.5 kcal/mol) suggests that an s-cis pathway cannot be completely excluded (Figure S25C); however, the overall results indicate that the s-trans pathway is more likely to be favored.

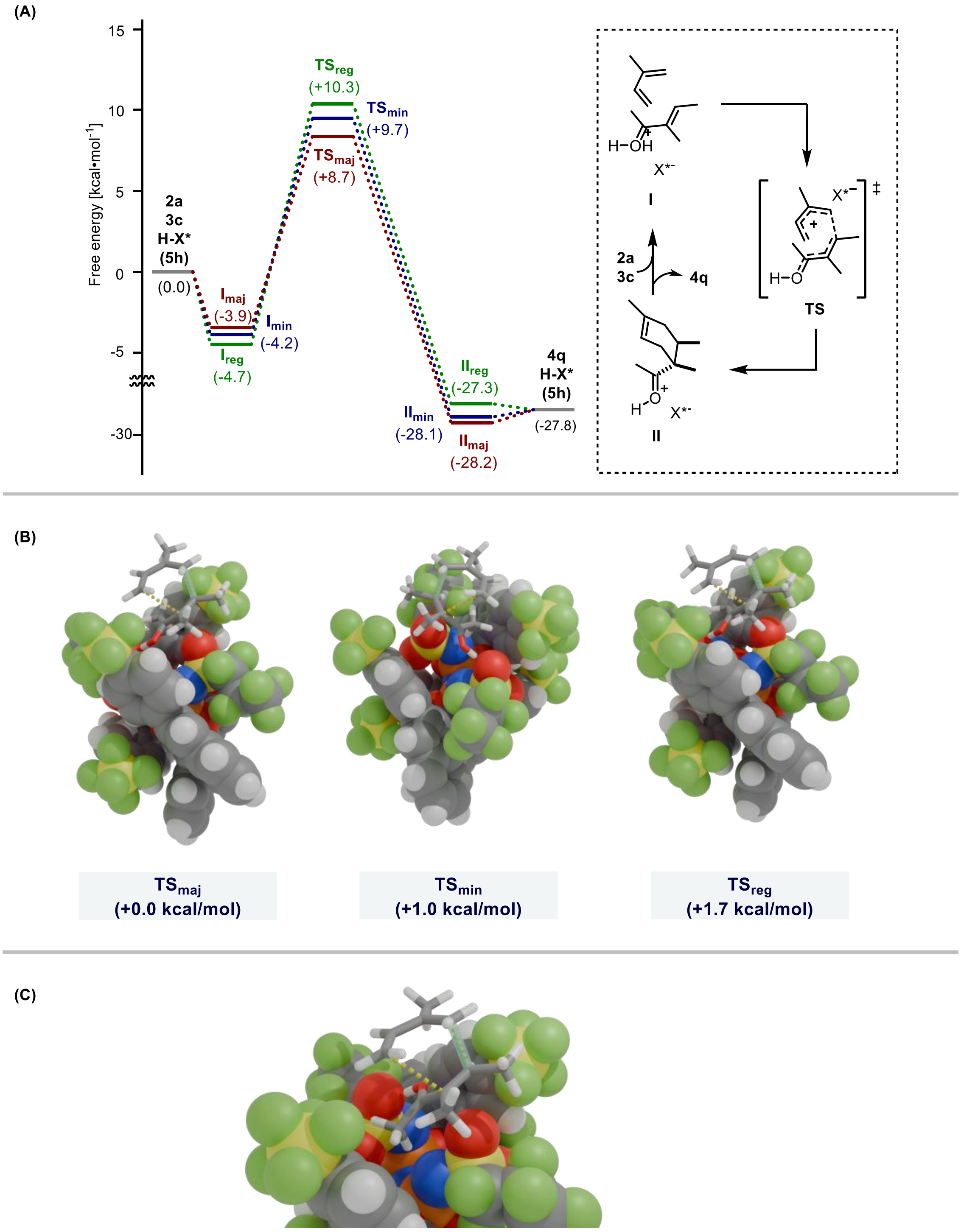

Figure S25. The calculated energy diagram of the proposed catalytic cycle.

IGMH analysis was conducted with Multiwfn 3.8(dev) using the default parameters with isovalue of 0.004 a.u.. **TS$_{maj}$** was partitioned into fragments comprising the chiral counteranion and the protonated aldehyde–diene complex.

To gain deeper insight into the origin of enantioselectivity, a distortion–interaction analysis was performed following the Houk–Bickelhaupt protocol (Table S25). Gas phase single point energies of the optimized **TS$_{maj}$** and **TS$_{min}$**, which lead to the major and minor enantiomers, respectively, were calculated at ωB97M-V/def2-TZVPP level of theory. These transition states were then decomposed into catalyst and substrate fragments. The results suggest that distortion of the catalyst is the primary factor controlling enantioselectivity. In contrast, comparison of **TS$_{maj}$** and **TS$_{reg}$** indicates that high regioselectivity arises from the substrate component, as **TS$_{reg}$** features a less stabilized cationic intermediate.

Table S25. Summary of the distortion-interaction analysis of **TS$_{maj}$** and **TS$_{min}$** relative to **I$_{maj}$**.

TS	ωB97M-V/def2-TZVPP<br>ΔE (kcal/mol)	Relative energy<br>ΔΔE (kcal/mol)
**TS$_{maj}$**	+12.32	+2.18
**TS$_{min}$**	+14.49	
Substrate fragment		
subst- **TS$_{maj}$**	+3.13	+0.37
subst- **TS$_{min}$**	+3.50	
Catalyst fragment		
cat- **TS$_{maj}$**	-0.51	+1.03
cat- **TS$_{min}$**	+0.52	
Total distortion		+1.39
Total interaction		+0.78

Table S26. Summary of the distortion-interaction analysis of **TS$_{maj}$** and **TS$_{reg}$** relative to **I$_{maj}$.**

TS	ωB97M-V/def2-TZVPP (in hartree)	Relative energy ΔΔE (kcal/mol)
**TS$_{maj}$**	+12.32	+1.20
**TS$_{reg}$**	+13.52	
Substrate fragment		
subst- **TS$_{maj}$**	+3.13	+2.22
subst- **TS$_{reg}$**	+5.35	
Catalyst fragment		
cat- **TS$_{maj}$**	-0.51	+0.83
cat- **TS$_{reg}$**	+0.32	
Total distortion		+3.05
Total interaction		-1.85

Additionally, a D3BJ "knock-out" study was performed at the B3LYP-D4 and B3LYP levels of theory (Table S27). The results indicate that dispersion correction is essential to reproduce the experimental outcomes, further supporting the importance of the noncovalent interactions discussed in the main text.

Table S27. Summary of the dispersion correction "knock-out" study between SMD(Chloroform)-B3LYP-D4/def2-TZVPP and SMD(Chloroform)-B3LYP/def2-TZVPP level of theory.

	B3LYP-D4 ΔΔG (kcal/mol)	B3LYP ΔΔG (kcal/mol)
**TS$_{maj}$**	0.00	0.00
**TS$_{min}$**	+1.89	-2.39
**TS$_{reg}$**	+1.94	+2.10

Furthermore, With the optimized **TS_{maj}**, **TS_{min}**, and **TS_{reg}**, Local Energy Decomposition analysis (LED, normalPNO) was performed following the procedure described by Bistoni *et al.* (Table S28, S29). In this analysis, the transition states were separated into the substrate cation and the chiral counteranion. The results indicate that **TS_{maj}** is more stable than **TS_{min}** due to interactions between the two fragments, while the energy difference between **TS_{maj}** and **TS_{reg}** mainly arises from geometry-preparation energy. These findings are consistent with those in Tables S25 and S26, although it should be noted that the definitions of geometry-preparation energy in LED and distortion energy in the Houk–Bickelhaupt model differ, which can lead to differences in how the energy components are distributed.

Table S28: Summary of the decomposition analysis between **TS_{maj}** and **TS_{min}** based on the DLPNO-CCSD(T)/def2-TZVP in gas phase using LED.

	**TS_{maj}** (in hartree)	**TS_{min}** (in hartree)	ΔΔE (in kcal/mol)
Total Energy	-10413.23749	-10413.23304	+2.79 ($\Delta E^{\ddagger}$)
Dispersion (strong pairs)	-0.009403497	-0.011886830	-1.56
Dispersion (weak pairs)	-0.028723448	-0.025593951	+1.96
Sum of dispersive correlation terms	-0.038126945	-0.037480781	+0.41 ($\Delta E_{disp}^{\ddagger}$)
Non dispersion (strong pairs)	-25.284534243	-25.277571915	+4.37
Non dispersion (weak pairs)	-0.301455724	-0.300309070	+0.72
Sum of non dispersive correlation terms	-25.58598997	-25.57788099	+5.09 ($\Delta E_{no\text{-}disp}^{\ddagger}$)
Sum of Interaction energies	-25.62411691	-25.61536177	+5.49 ($\Delta E_{int}^{\ddagger}$) (predominant factor)
Geometry preparation energy	-10387.61337	-10387.61768	-2.70 ($\Delta E_{geo\text{-}prep}^{\ddagger}$)

Table S29: Summary of the decomposition analysis between $\mathbf{TS_{maj}}$ and $\mathbf{TS_{reg}}$ based on the DLPNO-CCSD(T)/def2-TZVP in gas phase using LED.

	$\mathbf{TS_{maj}}$ (in hartree)	$\mathbf{TS_{reg}}$ (in hartree)	ΔΔE (in kcal/mol)
Total Energy	-10413.23749	-10413.23583	+1.04 ($\Delta E^{\ddagger}$)
Dispersion (strong pairs)	-0.009403497	-0.010516764	-0.70
Dispersion (weak pairs)	-0.028723448	-0.029131486	-0.25
Sum of dispersive correlation terms	-0.038126945	-0.03964825	-0.95 ($\Delta E_{disp}^{\ddagger}$)
Non dispersion (strong pairs)	-25.284534243	-25.28744484	-1.82
Non dispersion (weak pairs)	-0.301455724	-0.300174996	0.80
Sum of non dispersive correlation terms	-25.58598997	-25.58761984	-1.02 ($\Delta E_{no\text{-}disp}^{\ddagger}$)
Sum of Interaction energies	-25.62411691	-25.62726809	-1.98 ($\Delta E_{int}^{\ddagger}$)
Geometry preparation energy	-10387.61337	-10387.60856	+3.02 ($\Delta E_{geo\text{-}prep}^{\ddagger}$) (predominant factor)

For the KIE study, the methyl group of **2a** in the $\mathbf{TS_{maj}}$ was replaced with a hydrogen atom, and the corresponding transition state was re-optimized. Using this transition state and the ground state structure connected via the IRC pathway, a frequency calculation was performed at 213.15 K using the ORCA 5.0.3 program. KIE values were determined using the PyQuiver program with C10 as the reference atom (Table S30), and the

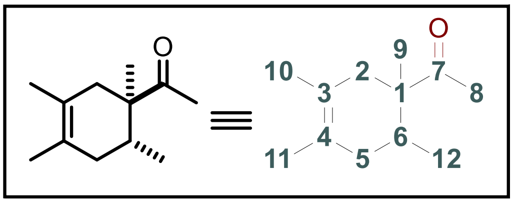

uncorrected KIE values are presented in the main text after change of reference to C2. For achiral transition states KIE was calculated manually (Tables S31,32). For achiral examples transition states were optimized using the same computational methods as used for the example catalyzed by **5h**.

Table S30. Summary of calculated KIE values for reaction catalysed by **IDPi-5h**.[45]

Isotopologue	Uncorrected KIE	Wigner KIE	Inverted parabola KIE
C2	1.0067	1.0071	1.0072
C3	1.0030	1.0030	1.0030
C4	1.0033	1.0035	1.0035
C5	1.0310	1.0355	1.0365
C11	1.0012	1.0012	1.0012
KIEs referenced to isotopologue C10, whose absolute KIEs are:			
C10	0.9987	0.9987	0.9987

Table S31. Summary of calculated KIE values for reaction under thermal conditions (393 K).

Isotopologue	Uncorrected KIE
C4	1.0042
C5	1.0166
C11	1.0021
KIEs referenced to isotopologue C2, whose absolute KIE is:	
C2	0.9927

Table S32. Summary of calculated KIE values for reaction catalyzed by $AlCl_3$ (113 K).

Isotopologue	Uncorrected KIE
C4	1.0026
C5	1.0215
C11	0.9992
KIEs referenced to isotopologue C2, whose absolute KIE is:	
C2	1.1809

Table S33. Energy table of the optimized structures. Energies are given in Hartree. Computed single point energies (E), Gibbs free energy corrections (Gcorr), Gibbs free energies (G), and the most negative imaginary frequencies for transition states are provided.

Structures	E (solv) in Hartree	Gcorr in Hartree	G (solv) in Hartree	Imaginary frequency
**2a**	-195.2773728	0.09376408	-195.1836087	-
**3c**	-309.8576797	0.12319064	-309.734489	-
**5h**	-9923.386136	0.93102766	-9922.455108	-
**I_{maj}**	-10428.56223	1.18277626	-10427.37945	-
**I_{min}**	-10428.56192	1.18197792	-10427.37994	-
**I_{reg}**	-10428.56251	1.18186687	-10427.38064	-
**TS_{maj}**	-10428.54517	1.18577345	-10427.3594	-291.81643777
**TS_{min}**	-10428.54395	1.18619729	-10427.35775	-293.20155122
**TS_{reg}**	-10428.54249	1.18573302	-10427.35676	-312.73505329
**II_{maj}**	-10428.61146	1.19330953	-10427.41815	-
**II_{min}**	-10428.60971	1.1916861	-10427.41803	-
**II_{reg}**	-10428.61043	1.19365052	-10427.41678	-
**4q**	-505.2040438	0.24171157	-504.9623322	-

Cartesian coordinates of the optimized structures

2a

C 0.946801 1.908735 -0.724271

H 1.116815 0.852425 -0.904472

H 1.326214 2.622294 -1.449120

C 0.284392 2.332541 0.363481

C -0.241486 1.341621 1.368782

H 0.585874 0.853328 1.893691

H -0.882453 1.801932 2.121287

H -0.816577 0.558455 0.867947

C 0.078608 3.762612 0.560769

H 0.501317 4.395758 -0.219600

C -0.558371 4.361224 1.572081

H	-0.637478	5.441574	1.595136
H	-1.009286	3.805122	2.385799

3c

C	-5.411253	-2.038565	-5.434012
H	-4.805293	-2.939845	-5.363276
C	-4.810051	-0.842031	-5.310968
C	-3.341226	-0.760028	-5.057034
O	-2.798120	0.324423	-4.908890
C	-2.514236	-2.031185	-4.979359
H	-2.847348	-2.670893	-4.155156
H	-2.590759	-2.613083	-5.903672
H	-1.473448	-1.750187	-4.815053
C	-5.549479	0.467320	-5.395990
H	-4.835484	1.291251	-5.433526
H	-6.186443	0.510400	-6.284647
H	-6.189063	0.618495	-4.518596
C	-6.867104	-2.269873	-5.656866
H	-7.030829	-2.808631	-6.599406
H	-7.276273	-2.910147	-4.864935
H	-7.444280	-1.344029	-5.682307

5h

O	2.190624	2.716050	-1.829536
O	0.229155	2.379843	-0.240478
O	2.066248	-1.651545	1.011300
O	3.250880	-1.574587	-1.222659
S	-0.858042	-1.716625	-0.765552
S	0.758404	0.891855	-4.154327
O	-1.088239	-1.671521	0.685227
O	-1.564194	-0.702726	-1.594438
O	2.167878	1.246402	-4.276971
O	0.194938	-0.383430	-4.563756
N	0.675887	-1.865945	-1.213580
N	0.233742	1.190102	-2.567130
P	1.882845	-1.115478	-0.504983
N	1.932798	0.479892	-0.462758
P	1.199133	1.599513	-1.255571
C	4.891930	2.122747	-1.085827
C	5.565159	1.211940	-0.266739
C	6.357325	0.210229	-0.809012
C	6.469415	0.129912	-2.188762
C	5.796361	1.002225	-3.033940
C	4.999603	1.993163	-2.473618
H	5.454034	1.267772	0.812336
H	6.869945	-0.488401	-0.159000

H	5.892100	0.929998	-4.110667	C	1.044767	0.074436	3.161395
H	4.474291	2.678716	-3.129796	C	0.029383	0.961191	3.500300
C	-2.563990	2.592707	-0.912082	C	0.298285	2.323529	3.486982
C	-3.491756	2.347748	-1.930951	C	1.544740	2.821607	3.135990
C	-4.450526	1.353381	-1.799551	C	2.548837	1.920791	2.813789
C	-4.476298	0.607257	-0.630661	H	0.834748	-0.989263	3.167034
C	-3.559912	0.808179	0.389338	H	-0.946828	0.580592	3.775044
C	-2.600701	1.799049	0.237325	H	1.741212	3.886426	3.106530
H	-3.457703	2.932549	-2.845225	H	3.520505	2.304870	2.517318
H	-5.159145	1.168696	-2.597716	C	-0.259404	3.632233	-0.664688
H	-3.577259	0.204320	1.288360	C	2.789948	3.511843	-0.834271
H	-1.889550	1.965463	1.036074	C	3.313421	-1.434357	1.597751
C	2.919267	-3.357349	-3.431724	C	3.612076	-2.918334	-1.037357
C	1.941279	-4.092877	-4.106205	C	-1.621638	3.723842	-1.035011
C	1.522845	-3.727997	-5.379191	C	3.411574	-3.811803	-2.115262
C	2.108647	-2.623254	-5.978116	C	4.120721	3.218207	-0.461964
C	3.074273	-1.864467	-5.333695	C	3.442722	-0.370112	2.521853
C	3.464325	-2.230559	-4.054250	C	0.574169	8.189415	-2.034676
H	1.478015	-4.945892	-3.620365	C	1.043669	7.026774	-1.472989
H	0.749903	-4.296455	-5.882411	C	0.176699	5.929642	-1.255027
H	3.519339	-0.999548	-5.810050	C	-1.189202	6.045839	-1.658374
H	4.221802	-1.645440	-3.548292	C	-1.643093	7.264097	-2.218853
C	2.321296	0.540933	2.832770	C	-0.781180	8.315490	-2.402667

H	1.255492	9.017870	-2.203741	C	8.184475	-1.393392	2.844498
H	2.091011	6.938798	-1.204097	H	8.920896	-2.932776	1.512440
C	0.624091	4.689804	-0.709359	H	6.720097	-3.464907	0.577464
C	-2.055765	4.943589	-1.511286	C	4.357597	-2.253606	1.219428
H	-2.685600	7.344356	-2.515235	C	4.684504	-0.171988	3.088501
H	-3.101933	5.062149	-1.780917	H	7.185477	0.101748	4.006139
C	3.728563	6.612184	2.997606	H	4.813001	0.620044	3.822693
C	4.482138	5.699311	2.302923	C	4.882594	-7.421965	0.792021
C	3.928478	4.981901	1.214602	C	4.465114	-6.972775	-0.435250
C	2.578453	5.246430	0.821577	C	4.196138	-5.599593	-0.651808
C	1.824132	6.183861	1.567648	C	4.395853	-4.673217	0.418749
C	2.386610	6.845862	2.632058	C	4.802066	-5.172743	1.679218
H	5.698397	3.799650	0.847808	C	5.039007	-6.514006	1.859384
H	5.512163	5.501369	2.588130	H	3.614411	-5.846952	-2.714026
C	4.669265	3.981684	0.547196	H	4.322199	-7.667213	-1.258999
C	2.020062	4.496258	-0.255771	H	4.916517	-4.485328	2.510795
H	0.786209	6.358751	1.306704	H	5.343268	-6.879706	2.835554
H	1.787420	7.547561	3.204134	C	3.725382	-5.136566	-1.898727
C	8.043981	-2.398270	1.864933	C	4.130303	-3.290318	0.186198
C	6.810174	-2.700171	1.340798	H	-1.136703	9.242238	-2.842560
C	5.652068	-2.005049	1.768008	H	4.158744	7.147731	3.838385
C	5.801915	-0.956715	2.730084	H	9.165678	-1.171510	3.252989
C	7.085527	-0.687706	3.265522	H	5.079566	-8.478150	0.948745

H	-0.537729	0.530627	-2.301289	F	6.778062	-2.352994	-2.086449
S	-5.758430	-0.685310	-0.434141	F	8.526082	-2.306664	-3.536266
F	-6.093936	-0.205631	1.098710	C	-1.988947	-3.434548	-2.833037
F	-4.676158	-1.768012	0.143462	C	-1.539196	-3.420822	-1.343421
F	-5.516517	-1.250523	-1.953561	C	-2.270611	-4.861271	-3.408273
F	-6.935624	0.312180	-0.998361	C	-0.294316	2.177269	-5.130244
F	-6.904303	-1.828804	-0.259034	C	0.220853	3.641165	-5.048476
S	1.589873	-2.147379	-7.668670	C	-0.767366	4.714895	-5.609672
F	0.001827	-2.225453	-7.277970	F	-0.278257	1.764727	-6.403555
F	1.545189	-3.711044	-8.165604	F	-1.548924	2.096419	-4.646784
F	3.147320	-2.035256	-8.178082	F	1.369657	3.732032	-5.747038
F	1.603600	-0.545887	-7.305387	F	0.457823	3.956341	-3.754501
F	1.135284	-1.728469	-9.176970	F	-1.877374	4.779911	-4.860867
S	-1.018110	3.511317	3.959712	F	-0.165411	5.909901	-5.583402
F	-2.207516	2.567996	3.334149	F	-1.108460	4.423859	-6.870455
F	-0.891708	4.300014	2.514121	F	-3.127381	-2.723673	-2.949804
F	0.058010	4.556286	4.622167	F	-1.023616	-2.879764	-3.596472
F	-1.230335	2.801535	5.418817	F	-2.587558	-3.698817	-0.555302
F	-2.181843	4.575088	4.363226	F	-0.581735	-4.348964	-1.145510
S	7.561334	-1.158353	-2.906390	F	-3.106540	-5.539263	-2.612159
F	8.642193	-1.026425	-1.680532	F	-2.826339	-4.740575	-4.620353
F	8.404332	-0.050349	-3.769763	F	-1.132201	-5.562989	-3.535995
F	6.568653	-1.395166	-4.192091				

I$_{maj}$

O	2.376094	2.361115	-2.069542
O	0.599776	1.883314	-0.337451
O	2.375295	-2.234818	0.297122
O	3.062918	-2.158601	-2.131755
S	-0.750092	-2.387854	-0.605524
S	0.645874	0.919161	-4.318008
O	-0.631792	-1.712085	0.700134
O	-1.978737	-2.168184	-1.387749
O	2.022624	0.467484	-4.573213
O	-0.450218	0.228816	-5.054971
N	0.532935	-2.301071	-1.553823
N	0.225653	1.107562	-2.797792
P	1.916890	-1.612667	-1.128669
N	2.096329	-0.045232	-1.082830
P	1.326248	1.212820	-1.616934
C	5.079725	1.472818	-1.887881
C	5.876666	0.466578	-1.333184
C	6.419191	-0.536393	-2.122120
C	6.145590	-0.529327	-3.480939
C	5.362039	0.452703	-4.069198
C	4.833424	1.453628	-3.264548
H	6.049261	0.440963	-0.261453
H	7.025663	-1.312203	-1.671829
H	5.153743	0.448960	-5.132148
H	4.217757	2.217877	-3.723315
C	-2.248895	2.452730	-0.484955
C	-2.164874	1.267784	0.256420
C	-3.245116	0.396917	0.342096
C	-4.419232	0.721592	-0.318975
C	-4.524416	1.854988	-1.113780
C	-3.430079	2.700499	-1.203301
H	-1.264203	1.015244	0.799723
H	-3.150449	-0.510259	0.926128
H	-5.435516	2.081741	-1.654646
H	-3.497807	3.572370	-1.846033
C	2.124948	-3.967120	-4.197147
C	2.323240	-2.760115	-4.875778
C	1.589435	-2.451430	-6.015273
C	0.652617	-3.363410	-6.476432
C	0.402718	-4.555010	-5.811668
C	1.133521	-4.839906	-4.668497
H	3.064786	-2.050126	-4.534676
H	1.763679	-1.511080	-6.523797
H	-0.347521	-5.250900	-6.167251
H	0.918972	-5.754688	-4.124434

C	3.025238	-0.243836	2.235891	C	-0.128098	8.216770	-0.916863
C	1.758862	-0.695563	2.623897	H	1.970378	8.703723	-1.122799
C	0.825272	0.177693	3.166626	H	2.745332	6.396010	-0.834965
C	1.173549	1.510979	3.326755	C	1.166509	4.204738	-0.409506
C	2.421775	1.992435	2.963469	C	-1.568576	4.820699	-0.519601
C	3.337202	1.108281	2.412407	H	-2.112300	7.432853	-0.745101
H	1.480818	-1.734103	2.496415	H	-2.623138	5.078919	-0.491547
H	-0.152783	-0.191014	3.451542	C	5.043822	5.444217	2.848718
H	2.682285	3.036499	3.085071	C	5.577659	4.587432	1.919462
H	4.298719	1.490450	2.083022	C	4.774858	4.044514	0.886067
C	0.196214	3.218049	-0.421531	C	3.397942	4.422608	0.797928
C	3.174985	2.926393	-1.080472	C	2.875945	5.296299	1.783228
C	3.708897	-2.104056	0.662527	C	3.678630	5.790966	2.782766
C	3.405676	-3.509984	-2.033098	H	6.357245	2.840823	0.047698
C	-1.195238	3.491248	-0.479850	H	6.624955	4.300131	1.969110
C	2.957452	-4.390569	-3.050425	C	5.305501	3.107146	-0.026581
C	4.528194	2.517994	-1.000984	C	2.596269	3.854443	-0.240891
C	4.040977	-1.142402	1.648457	H	1.825171	5.558942	1.758969
C	1.247915	7.910168	-0.956621	H	3.252355	6.446961	3.535772
C	1.684423	6.617645	-0.794936	C	8.358824	-3.293739	0.072885
C	0.762544	5.562599	-0.591241	C	7.042231	-3.491897	-0.266558
C	-0.631476	5.866767	-0.608041	C	6.008151	-2.783416	0.393561
C	-1.047829	7.213130	-0.750431	C	6.368377	-1.835521	1.402369

C	7.734303	-1.671679	1.741145	H	5.150652	-9.136848	-0.478201
C	8.710680	-2.384950	1.092403	H	-1.101097	-1.122837	-4.670323
H	9.136045	-3.835308	-0.457829	S	-5.880855	-0.360127	-0.124982
H	6.788399	-4.180868	-1.063957	F	-5.642152	-0.540289	1.478027
C	4.629266	-2.930717	0.050911	F	-5.002348	-1.717177	-0.415234
C	5.367931	-1.049387	2.013941	F	-6.251599	-0.263057	-1.744343
H	7.995483	-0.956807	2.517481	F	-6.874245	0.912479	0.155724
H	5.657023	-0.345917	2.791196	F	-7.188742	-1.326164	0.021591
C	4.944524	-8.075134	-0.574242	S	-0.278634	-2.991203	-8.007816
C	4.328248	-7.589814	-1.699366	F	-0.618207	-1.462609	-7.518920
C	4.041074	-6.208955	-1.829234	F	-1.704517	-3.509485	-7.353667
C	4.443059	-5.309634	-0.795944	F	-0.023320	-4.496479	-8.609371
C	5.048488	-5.847454	0.365541	F	1.057389	-2.451282	-8.780596
C	5.289684	-7.195699	0.472623	F	-1.119335	-2.665478	-9.369699
H	3.057267	-6.411192	-3.728634	S	-0.047285	2.680660	4.032786
H	4.031955	-8.260951	-2.501366	F	0.180932	3.755597	2.803898
H	5.310482	-5.185405	1.183440	F	1.099036	3.454927	4.916510
H	5.744887	-7.589115	1.376695	F	-0.364398	1.693741	5.301482
C	3.329050	-5.715802	-2.939333	F	-1.289870	2.004943	3.197969
C	4.168383	-3.916859	-0.953836	F	-1.130056	3.727665	4.654380
H	-0.456139	9.244899	-1.036281	S	6.836874	-1.871084	-4.518927
H	5.665912	5.847284	3.642131	F	6.259756	-3.032729	-3.502413
H	9.755233	-2.243491	1.352891	F	8.260492	-1.867580	-3.701492

F	7.453715	-0.808950	-5.603768	C	-4.796489	-2.873733	-3.274424
F	5.466900	-1.987635	-5.416542	H	-5.155795	-1.885457	-3.020463
F	7.450629	-3.071608	-5.433605	C	-3.555329	-2.992364	-3.805336
C	-0.925077	-4.265458	-0.230418	C	-2.773519	-1.798351	-3.993299
C	0.233096	-4.948863	0.544553	O	-1.622178	-1.985334	-4.533749
C	-0.055862	-6.411021	1.018932	C	-3.155859	-0.421112	-3.600235
C	0.581523	2.666132	-5.119445	H	-2.599964	-0.176041	-2.685699
C	-0.543617	3.589526	-4.569482	H	-2.846711	0.282010	-4.376790
C	-0.891085	4.808013	-5.484269	H	-4.218251	-0.310141	-3.402548
F	1.772736	3.258331	-4.914553	C	-2.907573	-4.291390	-4.191849
F	0.409597	2.483500	-6.444857	H	-3.462253	-5.146236	-3.805688
F	-0.153199	4.083908	-3.377180	H	-2.833855	-4.380271	-5.281026
F	-1.686445	2.878314	-4.406841	H	-1.889225	-4.332217	-3.798260
F	0.221655	5.472301	-5.822800	C	-5.740322	-3.965869	-2.949575
F	-1.523326	4.414582	-6.595036	H	-6.750256	-3.699691	-3.281210
F	-1.700524	5.638795	-4.807950	H	-5.467063	-4.935906	-3.365353
F	-2.065883	-4.394552	0.484343	H	-5.800625	-4.056441	-1.855163
F	-1.081957	-4.898528	-1.415469	C	-4.256866	-1.343916	-6.946002
F	0.528356	-4.228661	1.650368	H	-3.458078	-1.311203	-7.679191
F	1.318496	-5.006142	-0.258231	H	-4.498781	-2.317418	-6.526868
F	-0.441355	-7.178109	-0.011876	C	-6.774854	-1.176085	-5.265959
F	1.069660	-6.925431	1.536253	H	-6.676480	-2.139295	-5.758103
F	-1.006783	-6.438162	1.959373	H	-7.558708	-1.073967	-4.520843

C	-5.990639	-0.138778	-5.591222
C	-4.902304	-0.233668	-6.577092
H	-4.581186	0.715280	-7.009720
C	-6.194794	1.224038	-4.983202
H	-6.483567	1.944114	-5.759182
H	-6.974874	1.210791	-4.218085
H	-5.267912	1.602778	-4.534294

I$_{min}$

O	2.284373	3.078640	-1.805862
O	0.163706	2.684104	-0.483302
O	1.562489	-1.541803	0.429435
O	2.771623	-1.413658	-1.787774
S	-1.267352	-1.613336	-1.082445
S	0.955574	1.941953	-4.402218
O	-1.413201	-0.931253	0.216174
O	-2.336596	-1.478863	-2.084006
O	2.406780	1.728359	-4.484402
O	0.134115	1.163217	-5.378319
N	0.164916	-1.444599	-1.778091
N	0.286055	1.876051	-2.969775
P	1.454109	-0.850868	-1.031277
N	1.696193	0.701268	-0.891896
P	1.112264	1.983013	-1.586242
C	4.847418	2.155974	-1.003946
C	5.453820	1.120208	-0.286676
C	6.137068	0.100506	-0.933972
C	6.205462	0.126783	-2.318698
C	5.619162	1.139345	-3.065247
C	4.939558	2.150828	-2.399822
H	5.362891	1.084422	0.794726
H	6.589873	-0.699629	-0.361078
H	5.680768	1.152920	-4.146769
H	4.475190	2.936693	-2.983365
C	-2.585376	3.250657	-1.170892
C	-3.609506	3.400353	-2.117379
C	-4.687568	2.528622	-2.159879
C	-4.727402	1.480206	-1.250501
C	-3.709924	1.266534	-0.335933
C	-2.642866	2.154498	-0.302453
H	-3.565395	4.209694	-2.839040
H	-5.480790	2.676944	-2.883013
H	-3.727336	0.428837	0.349718
H	-1.865775	1.987259	0.430835
C	2.420346	-2.991809	-4.184243

C	1.622049	-3.755910	-5.048490	C	-1.539992	4.292306	-1.061041
C	1.313374	-3.317517	-6.327454	C	2.829665	-3.563404	-2.882196
C	1.796990	-2.084301	-6.743298	C	4.129976	3.220441	-0.271165
C	2.555843	-1.281259	-5.906787	C	2.981510	-0.590953	2.119460
C	2.861664	-1.739684	-4.631239	C	1.028499	8.671171	-1.141910
H	1.225812	-4.709966	-4.715460	C	1.392236	7.384866	-0.824543
H	0.702266	-3.930353	-6.979431	C	0.434796	6.342393	-0.823160
H	2.913737	-0.309516	-6.223820	C	-0.909329	6.650140	-1.194907
H	3.471935	-1.111459	-3.996544	C	-1.254644	7.989757	-1.496753
C	1.919203	0.360056	2.508919	C	-0.307700	8.981935	-1.468453
C	0.591770	-0.041419	2.689982	H	1.779199	9.455813	-1.147708
C	-0.383354	0.872290	3.068045	H	2.424934	7.156713	-0.583122
C	-0.018522	2.198122	3.256170	C	0.764198	4.990172	-0.506244
C	1.286238	2.633293	3.079037	C	-1.864841	5.617552	-1.271941
C	2.247235	1.705268	2.704620	H	-2.283105	8.213745	-1.767996
H	0.303482	-1.074069	2.533278	H	-2.892880	5.883910	-1.501113
H	-1.405465	0.541545	3.206968	C	3.765495	6.152536	3.589372
H	1.558667	3.672820	3.214496	C	4.492689	5.284315	2.814222
H	3.263104	2.046006	2.528280	C	3.955955	4.758256	1.613495
C	-0.197528	4.016113	-0.695519	C	2.652004	5.171460	1.192328
C	2.843322	3.656260	-0.671873	C	1.923633	6.057198	2.023378
C	2.797282	-1.514857	1.062318	C	2.466187	6.530979	3.193449
C	3.021675	-2.783328	-1.712245	H	5.664659	3.511470	1.182249

H	5.488657	4.973066	3.119197	H	3.005100	-5.545019	-3.659140
C	4.668297	3.801779	0.856812	H	3.487347	-7.529134	-2.353828
C	2.109216	4.615663	-0.007186	H	4.019932	-4.754270	1.733015
H	0.916448	6.341577	1.741884	H	4.266093	-7.192481	1.854267
H	1.882044	7.193596	3.824791	C	3.082255	-4.916874	-2.776112
C	7.391921	-2.983139	1.373055	C	3.474772	-3.299681	-0.512305
C	6.158942	-3.098436	0.777693	H	-0.580434	10.004631	-1.710586
C	5.056146	-2.338335	1.238257	H	4.182349	6.539254	4.514354
C	5.263184	-1.426572	2.322100	H	8.565601	-2.034188	2.926285
C	6.542268	-1.349356	2.925762	H	4.036684	-8.590199	-0.190270
C	7.586386	-2.110605	2.463458	H	-1.326975	0.820592	-5.265644
H	8.226953	-3.562836	0.991359	S	-6.179229	0.367633	-1.231445
H	6.030103	-3.760458	-0.070426	F	-6.282181	0.397808	0.398846
C	3.767781	-2.390047	0.621786	F	-5.244635	-0.976424	-1.153022
C	4.210713	-0.584147	2.742960	F	-6.198704	0.250087	-2.880423
H	6.684857	-0.661873	3.755840	F	-7.230562	1.624223	-1.325161
H	4.381714	0.094075	3.575818	F	-7.479193	-0.624748	-1.231319
C	3.917583	-7.513051	-0.257250	S	1.430442	-1.507897	-8.442730
C	3.616775	-6.925024	-1.459524	F	-0.183139	-1.842988	-8.270965
C	3.446811	-5.522564	-1.557482	F	1.659545	-3.010140	-9.068861
C	3.629937	-4.713711	-0.394781	F	2.996348	-1.142631	-8.735226
C	3.918559	-5.353490	0.834433	F	1.154786	0.023487	-7.949571
C	4.055427	-6.718925	0.899977	F	1.088956	-1.013007	-9.957484

S	-1.292802	3.418086	3.752969	F	-2.206360	5.748592	-6.270620
F	-2.448594	2.706508	2.830547	F	-1.164668	6.160688	-4.409440
F	-0.911782	4.412582	2.495055	F	-2.663979	-4.436557	-2.307054
F	-0.242237	4.234306	4.714323	F	-0.537699	-4.095821	-2.761708
F	-1.761919	2.510376	5.034057	F	-2.420912	-3.636304	0.175737
F	-2.420187	4.511839	4.187699	F	-0.229960	-3.764869	0.135068
S	7.095804	-1.222113	-3.183315	F	-1.801611	-6.342267	-0.370024
F	8.261245	-1.289177	-2.030307	F	-1.404549	-6.729648	-2.478472
F	8.004036	-0.164463	-4.044410	F	0.214878	-6.108022	-1.170196
F	5.997377	-1.267140	-4.403338	C	-4.369625	-2.127301	-4.427643
F	6.239912	-2.380104	-2.382736	H	-3.595249	-2.771901	-4.025316
F	7.882888	-2.427430	-3.945801	C	-3.988766	-0.966278	-5.017557
C	-1.410961	-4.461790	-1.792593	C	-2.587611	-0.638667	-5.036234
C	-1.327800	-3.469847	-0.596766	O	-2.329572	0.598588	-5.276169
C	-1.086071	-5.947749	-1.430243	C	-1.457126	-1.580335	-4.903732
C	0.717504	3.761441	-4.986789	H	-0.700536	-1.190122	-4.215958
C	-0.716513	4.075857	-5.501845	H	-1.760575	-2.572492	-4.585421
C	-1.042708	5.600403	-5.618841	H	-0.998506	-1.660520	-5.898822
F	1.007721	4.577266	-3.950931	C	-4.921053	0.069892	-5.581398
F	1.601344	3.978247	-5.975879	H	-4.868735	0.994200	-4.999088
F	-1.626209	3.538184	-4.651927	H	-4.632160	0.320541	-6.607422
F	-0.878244	3.530774	-6.724668	H	-5.954329	-0.274086	-5.582179
F	-0.081104	6.234875	-6.301577	C	-5.747837	-2.598252	-4.180467

H	-5.838006	-3.643887	-4.496714
H	-5.928787	-2.588970	-3.096816
H	-6.522814	-2.000089	-4.658925
C	-3.348533	-2.762128	-8.018407
H	-2.979953	-2.099271	-8.794897
H	-4.302077	-2.515541	-7.558200
C	-4.324604	-5.125443	-6.375456
H	-5.132291	-4.701710	-6.965086
H	-4.589640	-5.845339	-5.606413
C	-3.045455	-4.810146	-6.615721
C	-2.648919	-3.835445	-7.644015
H	-1.676657	-4.005179	-8.108741
C	-1.913347	-5.469184	-5.874490
H	-2.275485	-6.170528	-5.119432
H	-1.270419	-6.018202	-6.575077
H	-1.277542	-4.724760	-5.380073

I$_{reg}$

O	2.299549	2.333284	-2.136925
O	0.576458	1.841336	-0.354654
O	2.449753	-2.223942	0.295545
O	3.077233	-2.170803	-2.149471
S	-0.685179	-2.495910	-0.518880
S	0.542444	0.845537	-4.329019
O	-0.557315	-1.801175	0.775845
O	-1.945107	-2.338061	-1.265391
O	1.921213	0.423163	-4.619463
O	-0.557352	0.125186	-5.032319
N	0.563129	-2.360858	-1.507240
N	0.156986	1.033087	-2.799366
P	1.941786	-1.636514	-1.128425
N	2.088729	-0.065251	-1.115653
P	1.282701	1.171167	-1.645965
C	5.021027	1.492140	-2.019017
C	5.843536	0.500356	-1.476040
C	6.383930	-0.498633	-2.271499
C	6.083173	-0.501843	-3.624537
C	5.272460	0.465212	-4.200837
C	4.745342	1.461802	-3.389789
H	6.038864	0.482953	-0.408000
H	7.010721	-1.263070	-1.829759
H	5.042437	0.453394	-5.259216
H	4.108090	2.214197	-3.838760
C	-2.284748	2.362150	-0.404421
C	-2.151103	1.179589	0.334333

C	-3.212284	0.290456	0.464833	C	3.395080	1.163075	2.348885
C	-4.418356	0.594280	-0.147444	H	1.596406	-1.713554	2.505714
C	-4.575340	1.726388	-0.935398	H	-0.043038	-0.191996	3.485377
C	-3.499911	2.589564	-1.070904	H	2.719232	3.085363	3.018091
H	-1.225187	0.942353	0.840906	H	4.340765	1.559801	1.992096
H	-3.077855	-0.615309	1.043545	C	0.148572	3.168153	-0.435310
H	-5.511852	1.938621	-1.437017	C	3.114091	2.921033	-1.174239
H	-3.610098	3.459574	-1.710119	C	3.788964	-2.061681	0.625473
C	2.124457	-4.020957	-4.162226	C	3.450084	-3.513119	-2.040542
C	2.298837	-2.824406	-4.865294	C	-1.248720	3.417762	-0.448709
C	1.537438	-2.540421	-5.992568	C	2.990382	-4.417260	-3.031160
C	0.596165	-3.466376	-6.415251	C	4.475399	2.535843	-1.126708
C	0.366424	-4.646069	-5.722405	C	4.124795	-1.081316	1.591604
C	1.126598	-4.907195	-4.592655	C	1.104114	7.870871	-1.049033
H	3.044601	-2.105011	-4.554300	C	1.567283	6.587799	-0.886433
H	1.694199	-1.609122	-6.522824	C	0.670035	5.520013	-0.643317
H	-0.388447	-5.351696	-6.047680	C	-0.728683	5.800936	-0.619610
H	0.931829	-5.813394	-4.027201	C	-1.172064	7.138494	-0.763617
C	3.105319	-0.196568	2.193552	C	-0.275056	8.155171	-0.970181
C	1.857529	-0.668704	2.616421	H	1.807562	8.674312	-1.246375
C	0.920643	0.192251	3.173161	H	2.630095	6.383317	-0.956382
C	1.246941	1.533547	3.311810	C	1.102090	4.171049	-0.460585
C	2.476252	2.035242	2.913307	C	-1.644726	4.740828	-0.488723

H	-2.239428	7.340436	-0.727132
H	-2.701414	4.983351	-0.425653
C	5.044927	5.513779	2.675360
C	5.567692	4.654564	1.742064
C	4.746617	4.086325	0.737066
C	3.361624	4.440621	0.682018
C	2.851884	5.317251	1.671114
C	3.672852	5.837214	2.642548
H	6.325747	2.898268	-0.129905
H	6.620620	4.385148	1.766586
C	5.268278	3.147208	-0.179058
C	2.541989	3.847422	-0.328557
H	1.796436	5.561572	1.672883
H	3.256056	6.495040	3.399256
C	8.447143	-3.162225	-0.064048
C	7.127179	-3.390803	-0.369346
C	6.094950	-2.696806	0.309025
C	6.459677	-1.729946	1.297871
C	7.829713	-1.535252	1.602213
C	8.804558	-2.236023	0.937764
H	9.222463	-3.693273	-0.608021
H	6.867972	-4.092703	-1.153856
C	4.711771	-2.877484	0.002526
C	5.458008	-0.957013	1.924156
H	8.094841	-0.806739	2.364393
H	5.750878	-0.238843	2.686376
C	5.106661	-8.025879	-0.551241
C	4.450713	-7.569935	-1.666244
C	4.137370	-6.196355	-1.811223
C	4.553658	-5.273073	-0.804805
C	5.200611	-5.781033	0.347580
C	5.467057	-7.123209	0.470285
H	3.102380	-6.447331	-3.678229
H	4.142719	-8.259538	-2.447891
H	5.474244	-5.100869	1.146558
H	5.954056	-7.493714	1.367453
C	3.385624	-5.734332	-2.908852
C	4.248271	-3.888173	-0.976078
H	-0.623917	9.176266	-1.090904
H	5.681428	5.936628	3.446739
H	9.851929	-2.071008	1.171703
H	5.332636	-9.082397	-0.443289
H	-1.188585	-1.220660	-4.566840
S	-5.848872	-0.519786	0.091165
F	-5.543795	-0.711320	1.681520
F	-4.954544	-1.854729	-0.248041

F	-6.282628	-0.413725	-1.510710	C	0.194194	-6.472980	1.131815
F	-6.855801	0.729139	0.424161	C	0.415471	2.585144	-5.140808
F	-7.130062	-1.514120	0.277457	C	-0.704754	3.494154	-4.556287
S	-0.363734	-3.130929	-7.936742	C	-1.127917	4.680601	-5.480811
F	-0.696792	-1.591408	-7.474562	F	1.602087	3.200992	-4.986728
F	-1.774921	-3.637514	-7.244500	F	0.196639	2.387382	-6.457379
F	-0.115020	-4.648017	-8.510287	F	-0.269301	4.024805	-3.395877
F	0.956371	-2.604509	-8.745953	F	-1.821329	2.761263	-4.320284
F	-1.230403	-2.836331	-9.288688	F	-0.049846	5.359637	-5.893699
S	0.021286	2.686930	4.036141	F	-1.808341	4.243918	-6.546560
F	0.210436	3.761548	2.800260	F	-1.920222	5.510547	-4.783930
F	1.171818	3.481292	4.896142	F	-1.900478	-4.551691	0.598198
F	-0.258110	1.700040	5.313594	F	-0.912025	-5.022887	-1.308099
F	-1.225170	1.989251	3.225585	F	0.697039	-4.262811	1.737820
F	-1.066046	3.719439	4.674058	F	1.497438	-5.025308	-0.171902
S	6.774920	-1.837318	-4.670542	F	-0.183458	-7.261152	0.114099
F	6.225132	-3.003696	-3.644184	F	1.348087	-6.942283	1.629490
F	8.209921	-1.816730	-3.873561	F	-0.735840	-6.528375	2.091587
F	7.364524	-0.769505	-5.765072	C	-4.873464	-2.954483	-3.129567
F	5.394287	-1.970095	-5.549222	H	-5.226226	-1.961252	-2.884132
F	7.389092	-3.032129	-5.592199	C	-3.634316	-3.081893	-3.660792
C	-0.773121	-4.376927	-0.127709	C	-2.847531	-1.889808	-3.852078
C	0.419174	-5.004566	0.641742	O	-1.705706	-2.080053	-4.411794

C	-3.205590	-0.527102	-3.393356
H	-2.799230	-0.420699	-2.378779
H	-2.732780	0.226267	-4.024445
H	-4.279962	-0.361173	-3.350961
C	-2.991062	-4.381598	-4.051098
H	-3.564186	-5.236477	-3.693073
H	-2.891915	-4.453499	-5.139428
H	-1.982678	-4.439065	-3.632914
C	-5.826648	-4.037411	-2.798452
H	-5.543047	-5.018568	-3.179560
H	-5.918108	-4.097124	-1.704390
H	-6.826811	-3.781387	-3.166434
C	-4.226663	-1.428541	-6.687088
H	-3.340052	-1.389459	-7.310684
H	-4.606429	-2.413764	-6.432790
C	-6.898883	-1.310916	-5.264579
H	-6.810318	-2.238337	-5.824575
H	-7.748333	-1.213583	-4.595560
C	-6.022620	-0.311765	-5.407859
C	-4.820199	-0.306586	-6.256028
H	-6.208243	0.612813	-4.857463
C	-4.262573	1.052756	-6.581372
H	-4.984097	1.639139	-7.163529
H	-4.063240	1.621065	-5.663666
H	-3.332046	0.981080	-7.149056

II$_{maj}$

O	2.509545	2.470835	-1.987820
O	0.722696	2.044132	-0.255536
O	2.273638	-2.181424	0.261278
O	3.056023	-2.055723	-2.137013
S	-0.823892	-2.154141	-0.788395
S	0.696783	1.039513	-4.222287
O	-0.742964	-1.504553	0.532375
O	-2.005775	-1.876621	-1.625085
O	2.067022	0.567497	-4.475528
O	-0.413652	0.326525	-4.917384
N	0.506000	-2.102168	-1.671416
N	0.299525	1.304611	-2.710727
P	1.897753	-1.491516	-1.157963
N	2.142967	0.063206	-1.037732
P	1.416051	1.358825	-1.544073
C	5.171531	1.441404	-1.795844
C	5.923376	0.400322	-1.242054
C	6.444205	-0.610930	-2.034725
C	6.193599	-0.577606	-3.397627

C	5.445967	0.433458	-3.983808
C	4.938009	1.442149	-3.174951
H	6.079041	0.357464	-0.168297
H	7.018329	-1.411539	-1.585271
H	5.253676	0.448475	-5.049808
H	4.352320	2.230404	-3.632514
C	-2.094459	2.730859	-0.486278
C	-2.091498	1.541117	0.250147
C	-3.204322	0.708986	0.269877
C	-4.326339	1.075502	-0.457476
C	-4.347057	2.214607	-1.250953
C	-3.222932	3.024911	-1.267689
H	-1.229174	1.254198	0.836680
H	-3.175714	-0.203807	0.852049
H	-5.215993	2.472531	-1.844489
H	-3.221204	3.901451	-1.907712
C	2.120389	-3.766270	-4.296831
C	2.391444	-2.542856	-4.917531
C	1.727291	-2.164964	-6.077919
C	0.787918	-3.025725	-6.623054
C	0.468319	-4.234598	-6.021015
C	1.126864	-4.586321	-4.852535
H	3.134897	-1.871735	-4.510344

H	1.956549	-1.211281	-6.536327
H	-0.278507	-4.894758	-6.445235
H	0.853083	-5.512738	-4.357422
C	2.949298	-0.236646	2.246589
C	1.657257	-0.637549	2.604656
C	0.755641	0.266882	3.150883
C	1.160586	1.580105	3.342183
C	2.431868	2.013038	2.999020
C	3.315067	1.098944	2.444771
H	1.336747	-1.661113	2.455468
H	-0.240614	-0.063498	3.419599
H	2.736250	3.042447	3.141401
H	4.296149	1.444618	2.133116
C	0.373879	3.395865	-0.338632
C	3.331831	2.981115	-0.986852
C	3.600260	-2.108106	0.669666
C	3.333166	-3.424926	-2.070461
C	-1.002622	3.726410	-0.425224
C	2.883341	-4.256653	-3.128590
C	4.661831	2.503111	-0.903328
C	3.941606	-1.171345	1.675883
C	1.626910	8.048453	-0.786551
C	2.007211	6.736286	-0.641556

C	1.039367	5.716696	-0.472510
C	-0.340829	6.078102	-0.508271
C	-0.698845	7.442739	-0.634184
C	0.264033	8.410471	-0.765562
H	2.384104	8.814497	-0.924958
H	3.058763	6.471901	-0.666812
C	1.383795	4.340757	-0.305335
C	-1.321433	5.069976	-0.456479
H	-1.753308	7.706270	-0.643644
H	-2.366012	5.367767	-0.454151
C	5.294134	5.342032	2.994864
C	5.793282	4.477127	2.053964
C	4.971752	3.988418	1.008137
C	3.614886	4.432563	0.919346
C	3.127055	5.311073	1.917850
C	3.946150	5.751213	2.929493
H	6.497925	2.721130	0.160195
H	6.825949	4.141070	2.103712
C	5.461187	3.040252	0.083971
C	2.794377	3.922415	-0.133969
H	2.089368	5.622101	1.892349
H	3.546394	6.412645	3.692227
C	8.216598	-3.469143	0.192181
C	6.903970	-3.609200	-0.189166
C	5.879278	-2.870454	0.452120
C	6.246391	-1.952918	1.486208
C	7.607033	-1.848256	1.867058
C	8.573493	-2.590180	1.235830
H	8.987277	-4.033727	-0.323898
H	6.647411	-4.276597	-1.003894
C	4.506314	-2.957236	0.067811
C	5.260369	-1.135267	2.079327
H	7.872745	-1.155548	2.661713
H	5.554655	-0.452719	2.873108
C	4.620219	-8.092343	-0.691838
C	4.063956	-7.550092	-1.822235
C	3.842133	-6.154795	-1.922316
C	4.247652	-5.302713	-0.851274
C	4.790288	-5.897542	0.313326
C	4.968865	-7.257539	0.389907
H	2.921522	-6.260846	-3.859862
H	3.765521	-8.185603	-2.651998
H	5.053775	-5.269626	1.157379
H	5.376560	-7.694950	1.296421
C	3.192745	-5.599805	-3.041454
C	4.038749	-3.896028	-0.978279

H	-0.019544	9.453198	-0.871563	S	6.873706	-1.919325	-4.443512
H	5.929948	5.703246	3.797491	F	6.258415	-3.082752	-3.451837
H	9.614552	-2.494498	1.529095	F	8.284015	-1.954469	-3.604385
H	4.776266	-9.164399	-0.619144	F	7.527965	-0.855078	-5.504237
H	-1.097709	-1.025606	-4.580775	F	5.516219	-1.996745	-5.363445
S	-5.830151	0.038710	-0.358838	F	7.478332	-3.119342	-5.364861
F	-5.721364	-0.109761	1.263016	C	-1.075805	-4.031395	-0.456715
F	-4.969235	-1.345316	-0.547579	C	0.020848	-4.770282	0.356080
F	-6.065575	0.108616	-2.000348	C	-0.347761	-6.225853	0.795721
F	-6.807526	1.342501	-0.181660	C	0.644364	2.757464	-5.081791
F	-7.171433	-0.890266	-0.294663	C	-0.451607	3.719473	-4.540052
S	-0.045790	-2.552933	-8.183755	C	-0.778226	4.927681	-5.476129
F	-0.373525	-1.043006	-7.633863	F	1.850273	3.330964	-4.911842
F	-1.523862	-3.058490	-7.636179	F	0.446657	2.538426	-6.397739
F	0.200059	-4.035963	-8.841836	F	-0.039758	4.228476	-3.360809
F	1.346404	-2.016586	-8.852074	F	-1.609388	3.038576	-4.355023
F	-0.795965	-2.136944	-9.571161	F	0.346106	5.563401	-5.830667
S	-0.009469	2.787619	4.069955	F	-1.422406	4.527045	-6.577083
F	0.270737	3.877521	2.865320	F	-1.568240	5.786488	-4.811777
F	1.166910	3.491214	4.972895	F	-2.250734	-4.135837	0.206593
F	-0.373152	1.787928	5.316175	F	-1.200433	-4.637126	-1.660557
F	-1.278473	2.185925	3.218978	F	0.294853	-4.076998	1.484108
F	-1.047048	3.867338	4.711969	F	1.136500	-4.861600	-0.400008

F	-0.718015	-6.962307	-0.262455
F	0.732277	-6.792425	1.353691
F	-1.339110	-6.226522	1.693641
C	-4.965517	-2.860590	-3.485027
H	-5.034742	-1.891588	-2.986613
C	-3.850186	-2.769927	-4.574894
C	-2.895698	-1.655892	-4.325953
O	-1.673048	-1.869155	-4.608554
C	-3.350477	-0.321336	-3.893842
H	-3.369013	-0.334709	-2.794973
H	-2.659041	0.463476	-4.204742
H	-4.372329	-0.136310	-4.237267
C	-3.124579	-4.096602	-4.800375
H	-3.862632	-4.884332	-4.978961
H	-2.473097	-4.034132	-5.672960
H	-2.515534	-4.374546	-3.937389
C	-4.686628	-3.899256	-2.401384
H	-5.461551	-3.826467	-1.632190
H	-4.700964	-4.920352	-2.795561
H	-3.725987	-3.720280	-1.916088
C	-4.472649	-2.309319	-5.983031
H	-3.715331	-1.790286	-6.582118
H	-4.651525	-3.259061	-6.505846
C	-6.347648	-3.084940	-4.133372
H	-6.342177	-4.028673	-4.702480
H	-7.094564	-3.199383	-3.339782
C	-6.687006	-1.923155	-5.024574
C	-5.740380	-1.528712	-5.883471
H	-5.883551	-0.666058	-6.530687
C	-8.001963	-1.238095	-4.845820
H	-8.829414	-1.940194	-5.009511
H	-8.103044	-0.868052	-3.816961
H	-8.119653	-0.396454	-5.533997

II_{min}

O	2.230380	3.072386	-1.825350
O	0.158504	2.713272	-0.417310
O	1.515561	-1.525448	0.414904
O	2.799903	-1.385798	-1.761068
S	-1.253525	-1.694800	-1.229629
S	0.744787	1.972349	-4.364549
O	-1.485323	-1.008218	0.052660
O	-2.272540	-1.601490	-2.288298
O	2.181271	1.705499	-4.521634
O	-0.158293	1.232992	-5.300469

N	0.204442	-1.483999	-1.861124	H	-3.780160	0.518346	0.430387
N	0.146059	1.923534	-2.903912	H	-1.877680	2.034627	0.508124
P	1.440473	-0.852049	-1.058112	C	2.550854	-3.012471	-4.164862
N	1.636742	0.704141	-0.908558	C	1.868663	-3.852165	-5.060319
P	1.043640	1.999738	-1.563293	C	1.582035	-3.454610	-6.356865
C	4.794733	2.057671	-1.080352	C	1.956270	-2.178960	-6.756719
C	5.403920	1.025663	-0.359939	C	2.590151	-1.300713	-5.891654
C	6.082038	0.001025	-1.003337	C	2.886111	-1.724116	-4.601649
C	6.137663	0.012657	-2.388167	H	1.537773	-4.833268	-4.735650
C	5.532115	1.010707	-3.139532	H	1.061166	-4.125082	-7.030455
C	4.858907	2.030163	-2.477997	H	2.855244	-0.295602	-6.196431
H	5.327070	1.003187	0.722990	H	3.401626	-1.035486	-3.946783
H	6.544443	-0.789986	-0.425892	C	1.804405	0.443127	2.470928
H	5.582074	1.011006	-4.222055	C	0.471021	0.051708	2.630952
H	4.386099	2.808558	-3.065128	C	-0.498055	0.969833	3.017000
C	-2.597860	3.342985	-1.054720	C	-0.122355	2.289209	3.230861
C	-3.631641	3.531350	-1.983279	C	1.185107	2.716322	3.057322
C	-4.725345	2.680750	-2.030119	C	2.139257	1.785658	2.674476
C	-4.776505	1.618936	-1.137579	H	0.175548	-0.977143	2.461876
C	-3.750881	1.367378	-0.241006	H	-1.523027	0.645625	3.152221
C	-2.663628	2.230737	-0.208264	H	1.466825	3.751325	3.206849
H	-3.581095	4.352533	-2.690759	H	3.159053	2.119910	2.508327
H	-5.518719	2.851007	-2.747819	C	-0.184292	4.053898	-0.619658

C	2.841725	3.624338	-0.704527	C	2.071008	6.040081	2.026714
C	2.733306	-1.459853	1.079563	C	2.667562	6.504136	3.174192
C	3.074332	-2.751164	-1.671715	H	5.710870	3.395200	1.064489
C	-1.529387	4.360296	-0.948327	H	5.648125	4.875025	2.994220
C	2.947169	-3.545689	-2.842193	C	4.714024	3.714784	0.769976
C	4.123825	3.146903	-0.339923	C	2.155171	4.598951	-0.011020
C	2.876689	-0.512234	2.121910	H	1.060270	6.346367	1.781986
C	1.129956	8.683410	-1.091805	H	2.122995	7.182225	3.824302
C	1.475634	7.387797	-0.792819	C	7.336159	-2.866769	1.562714
C	0.495459	6.367082	-0.762532	C	6.127596	-2.998690	0.922357
C	-0.853217	6.706239	-1.086413	C	5.000117	-2.247401	1.336826
C	-1.178988	8.054813	-1.369990	C	5.159907	-1.321596	2.415761
C	-0.209626	9.025534	-1.370000	C	6.414620	-1.226822	3.066735
H	1.897320	9.451270	-1.120289	C	7.481467	-1.983819	2.652630
H	2.510782	7.135692	-0.588476	H	8.190145	-3.441886	1.217668
C	0.803819	5.006629	-0.461773	H	6.037970	-3.668478	0.075068
C	-1.831635	5.694174	-1.139759	C	3.732558	-2.323368	0.680967
H	-2.210909	8.302621	-1.604808	C	4.085843	-0.483114	2.784631
H	-2.859997	5.983012	-1.337965	H	6.519815	-0.529504	3.894161
C	3.972347	6.097762	3.522525	H	4.222485	0.210302	3.611133
C	4.649321	5.209453	2.725213	C	4.064487	-7.442188	-0.148177
C	4.056014	4.692979	1.546916	C	3.802391	-6.870121	-1.366966
C	2.749209	5.137632	1.170853	C	3.587558	-5.474782	-1.480061

C	3.686647	-4.655981	-0.315439	F	3.045196	-0.962290	-8.662145
C	3.936805	-5.279504	0.930350	F	0.982079	-0.189285	-7.931282
C	4.117110	-6.639103	1.010156	F	1.206847	-1.147690	-9.971262
H	3.241519	-5.524141	-3.594775	S	-1.377230	3.511298	3.771346
H	3.736312	-7.481820	-2.263032	F	-2.548928	2.841039	2.840202
H	3.972718	-4.673039	1.829115	F	-0.990301	4.536239	2.539739
H	4.296269	-7.101503	1.976273	F	-0.307763	4.285159	4.747066
C	3.253666	-4.887290	-2.715177	F	-1.847006	2.573502	5.030318
C	3.495700	-3.248644	-0.452051	F	-2.485757	4.607029	4.245724
H	-0.467543	10.055290	-1.598069	S	7.049215	-1.330949	-3.238685
H	4.432567	6.478522	4.429244	F	8.237919	-1.335107	-2.107402
H	8.441284	-1.895884	3.152546	F	7.909177	-0.268868	-4.142717
H	4.217119	-8.514263	-0.069061	F	5.929562	-1.437231	-4.434367
H	-1.540999	0.674843	-5.085372	F	6.245334	-2.493223	-2.391463
S	-6.254466	0.540438	-1.123783	F	7.856850	-2.531421	-3.986983
F	-6.385594	0.618409	0.505928	C	-1.408129	-4.540064	-1.939317
F	-5.353458	-0.819809	-0.986472	C	-1.248330	-3.555747	-0.742956
F	-6.238381	0.389235	-2.767939	C	-0.953747	-6.005446	-1.640869
F	-7.272747	1.820889	-1.265200	C	0.537696	3.798795	-4.939214
F	-7.574726	-0.417036	-1.124623	C	-0.896043	4.137009	-5.442206
S	1.568593	-1.628448	-8.458099	C	-1.204421	5.667537	-5.526609
F	0.037894	-2.260048	-8.379763	F	0.850188	4.603588	-3.901913
F	2.101918	-3.037717	-9.106052	F	1.417116	4.005963	-5.933811

F	-1.808983	3.592834	-4.600508
F	-1.066600	3.619779	-6.675633
F	-0.234884	6.304077	-6.196464
F	-2.366004	5.843687	-6.174294
F	-1.318736	6.203688	-4.305412
F	-2.709401	-4.589650	-2.300759
F	-0.675316	-4.114937	-2.999387
F	-2.270490	-3.749376	0.112741
F	-0.090146	-3.831557	-0.102043
F	-1.515566	-6.453469	-0.511759
F	-1.342647	-6.795301	-2.658406
F	0.377002	-6.087987	-1.534739
C	-3.959267	-2.900901	-4.895926
H	-3.017939	-3.193809	-4.419271
C	-3.654441	-1.699472	-5.858398
C	-2.412304	-1.010102	-5.418344
O	-2.470118	0.247890	-5.229130
C	-1.127055	-1.715282	-5.285700
H	-0.875765	-1.766850	-4.217421
H	-1.176950	-2.725226	-5.691435
H	-0.331671	-1.135458	-5.761755
C	-4.823987	-0.722527	-5.990002
H	-5.037493	-0.207027	-5.052266
H	-4.609704	0.031731	-6.753349
H	-5.720661	-1.269148	-6.296416
C	-4.938193	-2.545341	-3.782290
H	-5.006535	-3.384150	-3.083274
H	-4.603115	-1.679949	-3.210923
H	-5.943153	-2.346416	-4.167697
C	-3.277749	-2.217682	-7.320266
H	-2.578241	-1.522984	-7.801638
H	-4.216419	-2.126571	-7.883924
C	-4.431893	-4.131036	-5.696012
H	-5.360800	-3.883810	-6.234622
H	-4.681888	-4.932342	-4.990903
C	-3.360718	-4.573664	-6.652546
C	-2.780320	-3.624995	-7.393978
H	-1.968894	-3.855803	-8.080178
C	-2.959943	-6.013280	-6.669184
H	-3.817124	-6.652643	-6.915575
H	-2.164729	-6.205570	-7.394974
H	-2.612397	-6.327876	-5.675662

II$_{reg}$

O	2.407353	2.417806	-2.071100

O	0.650921	1.959459	-0.314446	H	4.239174	2.242998	-3.738566
O	2.339247	-2.207167	0.237470	C	-2.189339	2.553879	-0.461733
O	3.055550	-2.095268	-2.182695	C	-2.121587	1.388279	0.309891
S	-0.778523	-2.322487	-0.721709	C	-3.211703	0.532246	0.412167
S	0.627055	0.964669	-4.284332	C	-4.376032	0.848068	-0.270836
O	-0.700332	-1.630910	0.577515	C	-4.459551	1.956072	-1.103859
O	-1.996701	-2.136228	-1.533487	C	-3.358407	2.792023	-1.201522
O	2.001216	0.510907	-4.547886	H	-1.226260	1.142655	0.864621
O	-0.476863	0.246612	-4.987920	H	-3.134023	-0.356940	1.025560
N	0.520140	-2.217939	-1.645327	H	-5.360945	2.171045	-1.665245
N	0.225024	1.195161	-2.767772	H	-3.408973	3.646608	-1.868722
P	1.905585	-1.554163	-1.181773	C	2.125171	-3.858559	-4.297343
N	2.105293	0.008804	-1.095758	C	2.347669	-2.637910	-4.942763
P	1.347162	1.283527	-1.605852	C	1.643481	-2.296165	-6.090942
C	5.102355	1.488086	-1.907823	C	0.713492	-3.190217	-6.597899
C	5.890680	0.472667	-1.357461	C	0.441958	-4.397277	-5.969664
C	6.424409	-0.532023	-2.150132	C	1.140433	-4.713253	-4.814251
C	6.150975	-0.517401	-3.508943	H	3.084369	-1.941284	-4.566511
C	5.371491	0.470988	-4.092248	H	1.835742	-1.344596	-6.570645
C	4.850703	1.473048	-3.283618	H	-0.297863	-5.083523	-6.363701
H	6.064669	0.442664	-0.286093	H	0.905133	-5.639144	-4.298670
H	7.025248	-1.314036	-1.702935	C	3.006520	-0.223945	2.187742
H	5.162913	0.472640	-5.155220	C	1.732721	-0.653924	2.576348

C	0.820627	0.232147	3.135403	H	2.840306	6.450554	-0.822086
C	1.196329	1.556662	3.308013	C	1.239339	4.274943	-0.399808
C	2.448986	2.018218	2.934725	C	-1.490230	4.916337	-0.516447
C	3.342960	1.121772	2.368950	H	-2.007883	7.532780	-0.754032
H	1.434416	-1.686092	2.441926	H	-2.543026	5.182888	-0.495879
H	-0.160980	-0.120969	3.428138	C	5.152530	5.445974	2.838988
H	2.730333	3.056113	3.062659	C	5.669059	4.585611	1.903247
H	4.309039	1.488578	2.035417	C	4.852504	4.058014	0.872641
C	0.259170	3.298898	-0.403156	C	3.480986	4.456877	0.794153
C	3.223305	2.970239	-1.087734	C	2.976311	5.332907	1.786339
C	3.672820	-2.092291	0.612392	C	3.791762	5.811770	2.783212
C	3.378830	-3.453535	-2.105601	H	6.410605	2.832427	0.020823
C	-1.128674	3.584300	-0.464807	H	6.712469	4.283461	1.945676
C	2.930570	-4.311675	-3.142583	C	5.362766	3.115429	-0.046216
C	4.570233	2.540242	-1.017137	C	2.665088	3.905165	-0.241718
C	4.010451	-1.137415	1.602725	H	1.928910	5.609867	1.768767
C	1.357509	7.978350	-0.952958	H	3.379388	6.470099	3.541892
C	1.781326	6.682137	-0.786948	C	8.313322	-3.328939	0.041176
C	0.848820	5.636214	-0.584468	C	6.996834	-3.509155	-0.308593
C	-0.542619	5.953142	-0.606609	C	5.966634	-2.793694	0.350080
C	-0.945552	7.302899	-0.754884	C	6.331162	-1.857691	1.368273
C	-0.015783	8.297507	-0.920005	C	7.696589	-1.711996	1.717239
H	2.087935	8.764968	-1.117298	C	8.668928	-2.431811	1.069711

H	9.087701	-3.875977	-0.488086	S	-5.854566	-0.206613	-0.052222
H	6.740674	-4.189655	-1.112457	F	-5.692200	-0.234261	1.572711
C	4.588289	-2.922394	-0.002031	F	-4.974660	-1.586634	-0.163963
C	5.336308	-1.062481	1.976788	F	-6.136378	-0.257011	-1.684321
H	7.960727	-1.005920	2.500579	F	-6.850490	1.090413	0.064641
H	5.629010	-0.365666	2.758636	F	-7.175299	-1.148925	0.121067
C	4.840789	-8.060987	-0.701993	S	-0.172887	-2.767599	-8.143740
C	4.242265	-7.550350	-1.825500	F	-0.523919	-1.255746	-7.613313
C	3.975642	-6.163796	-1.937198	F	-1.622003	-3.300084	-7.546829
C	4.380166	-5.286034	-0.886654	F	0.093397	-4.255746	-8.781593
C	4.967096	-5.849286	0.272150	F	1.186829	-2.209352	-8.859256
C	5.188440	-7.202233	0.361140	F	-0.970933	-2.396903	-9.517345
H	3.011880	-6.323037	-3.850336	S	0.011584	2.740919	4.050912
H	3.944231	-8.205261	-2.640167	F	0.240921	3.827529	2.832433
H	5.230398	-5.203103	1.102310	F	1.188428	3.479603	4.924542
H	5.629635	-7.615518	1.263275	F	-0.302689	1.742757	5.311378
C	3.282739	-5.643591	-3.047006	F	-1.258734	2.101721	3.229834
C	4.124781	-3.887932	-1.025366	F	-1.038746	3.800413	4.705366
H	-0.333869	9.328312	-1.043254	S	6.841083	-1.854817	-4.553728
H	5.784907	5.837085	3.630241	F	6.258299	-3.021001	-3.545826
H	9.713300	-2.304410	1.338055	F	8.262442	-1.859795	-3.732711
H	5.031023	-9.126824	-0.619946	F	7.463254	-0.787343	-5.630205
H	-1.114557	-1.090044	-4.597479	F	5.473341	-1.962241	-5.455633

F	7.454201	-3.051081	-5.474164	C	-3.800242	-2.921012	-4.301567
C	-0.923446	-4.200827	-0.328463	C	-2.864770	-1.767866	-4.165953
C	0.222863	-4.861403	0.482981	O	-1.667888	-1.951999	-4.555467
C	-0.074755	-6.313712	0.984471	C	-3.307927	-0.430405	-3.724443
C	0.549633	2.690406	-5.130605	H	-3.120717	-0.381795	-2.641819
C	-0.550844	3.636742	-4.571273	H	-2.719841	0.360778	-4.193745
C	-0.900575	4.847620	-5.495434	H	-4.381019	-0.301419	-3.881202
F	1.751239	3.274590	-4.969184	C	-3.036468	-4.226793	-4.531900
F	0.341057	2.477562	-6.445583	H	-3.748394	-5.050538	-4.628400
F	-0.130480	4.141420	-3.393644	H	-2.452700	-4.170350	-5.451782
F	-1.699033	2.941891	-4.377435	H	-2.354458	-4.443201	-3.706904
F	0.213550	5.496340	-5.858692	C	-4.615506	-4.151574	-2.160735
F	-1.553318	4.447285	-6.591502	H	-4.817925	-5.117874	-2.634050
F	-1.690961	5.693955	-4.816371	H	-3.603392	-4.159995	-1.756736
F	-2.078859	-4.345135	0.360440	H	-5.304663	-4.043935	-1.317485
F	-1.037235	-4.848115	-1.511645	C	-4.526392	-2.557676	-5.678235
F	0.495588	-4.117244	1.578505	H	-3.784416	-2.210392	-6.407460
F	1.322000	-4.934306	-0.298552	H	-4.878685	-3.530898	-6.048583
F	-0.463585	-7.097805	-0.031638	C	-6.292878	-2.980711	-3.628516
F	1.048009	-6.823526	1.512426	H	-6.499376	-3.945798	-4.121130
F	-1.025739	-6.316901	1.924796	H	-6.957158	-2.928258	-2.759866
C	-4.832652	-2.983747	-3.121753	C	-6.554009	-1.847901	-4.571259
H	-4.714741	-2.073776	-2.532478	C	-5.686311	-1.615603	-5.560080

H	-7.422145	-1.208827	-4.424498
C	-5.798083	-0.501617	-6.553158
H	-5.846041	-0.891730	-7.577799
H	-6.689832	0.105029	-6.373860
H	-4.916585	0.153204	-6.513083

4q

C	-5.062682	-3.338920	-3.395874
H	-4.747099	-3.410029	-2.348235
C	-3.898170	-2.687318	-4.180988
C	-3.566016	-1.325509	-3.531833
O	-3.675839	-1.172799	-2.331183
C	-3.031619	-0.207796	-4.400219
H	-3.818472	0.133345	-5.082587
H	-2.715006	0.618548	-3.762367
H	-2.191985	-0.544216	-5.017824
C	-2.594898	-3.499362	-4.056674
H	-2.690943	-4.465270	-4.561240
H	-1.754312	-2.971412	-4.521985
H	-2.348908	-3.673704	-3.004306
C	-5.417711	-4.741736	-3.896828
H	-6.179607	-5.181930	-3.244820
H	-5.832448	-4.714575	-4.910642
H	-4.557851	-5.418089	-3.892867
C	-4.300617	-2.548740	-5.654687
H	-3.552363	-1.966989	-6.207362
H	-4.280170	-3.546113	-6.121843
C	-6.296185	-2.424650	-3.462462
H	-7.183141	-2.983701	-3.129604
H	-6.191874	-1.592467	-2.751866
C	-6.564200	-1.864472	-4.835859
C	-5.659407	-1.932524	-5.817862
H	-5.908862	-1.527085	-6.798406
C	-7.905107	-1.219336	-5.027107
H	-8.712859	-1.945536	-4.868916
H	-8.057771	-0.417304	-4.292986
H	-8.013807	-0.794691	-6.028994

TS$_{maj}$, Imaginary frequency = -291.81643777, -34.04241426

O	2.471430760594	2.431885568069	-2.041623149185

O	0.667921483675	1.974044726226	-0.335631912949
O	2.279551778233	-2.193007513047	0.255081884024
O	3.015074625951	-2.115086440506	-2.159249900962
S	-0.829761702149	-2.264297828834	-0.703874418254
S	0.716808438139	1.092978648145	-4.341007344453
O	-0.713446288939	-1.628012323088	0.622075357280
O	-2.044085751357	-2.003872410527	-1.493749593235
O	2.076357529642	0.580195403545	-4.580564020590
O	-0.396270061094	0.476434382004	-5.108393909737
N	0.469694409467	-2.182751490247	-1.628955263484
N	0.287167240201	1.271190047532	-2.819678603459
P	1.865861989793	-1.543074934390	-1.173410309175
N	2.100896327211	0.015629329092	-1.112524858388
P	1.377639555435	1.308729521049	-1.628529470999
C	5.144267800914	1.461321327395	-1.833605965720
C	5.915565961365	0.435778885508	-1.277612909694
C	6.435483487084	-0.580165930937	-2.065340745007
C	6.164723888567	-0.566441316331	-3.424749504236
C	5.406977829998	0.434684862733	-4.014429642654
C	4.901726375454	1.448652283148	-3.211146919595
H	6.083620252407	0.405243834854	-0.205372761843
H	7.021385902573	-1.371143566019	-1.614054477312
H	5.199893864995	0.435783891236	-5.077650134704

H	4.305853056197	2.226962947529	-3.671897052896
C	-2.160159940442	2.602711145502	-0.480719784421
C	-2.100150837620	1.417006054691	0.261219591694
C	-3.194688485977	0.564387008464	0.341772516305
C	-4.358143846866	0.907556561486	-0.328156120615
C	-4.439224514403	2.040949873094	-1.125998791364
C	-3.331821796717	2.870226766454	-1.206520384172
H	-1.206528636093	1.149158272403	0.808384786430
H	-3.118356491549	-0.343460428456	0.927305925654
H	-5.342830867084	2.281920532813	-1.672909975632
H	-3.379894553646	3.742492696824	-1.850479241181
C	2.089949702164	-3.878332377718	-4.274846444885
C	2.296347548509	-2.649494140744	-4.911685837163
C	1.597084206059	-2.313565289873	-6.065149746407
C	0.691319643235	-3.223164905908	-6.587543128960
C	0.435515073944	-4.438321897837	-5.969096557489
C	1.125080894628	-4.746425675312	-4.806934287478
H	3.015920013426	-1.940226252475	-4.525891524211
H	1.775640133994	-1.354234231376	-6.535370495701
H	-0.285906938407	-5.136598011558	-6.376246237206
H	0.898214098528	-5.677767115249	-4.297691452184
C	2.946280509607	-0.230899177038	2.218256942117
C	1.664642074707	-0.652955255922	2.589383312308

C	0.750966093811	0.238185492443	3.137248753187
C	1.133542415340	1.559677866778	3.317305711832
C	2.395307601162	2.012607105413	2.964814839853
C	3.290781615305	1.111259649232	2.409151414788
H	1.359999492780	-1.682126925909	2.446624604528
H	-0.237715031901	-0.108465381256	3.413288910499
H	2.682449519458	3.047930767721	3.099581566692
H	4.263846023407	1.472223698562	2.089763642846
C	0.296347281831	3.317827699087	-0.400877386855
C	3.271020290782	2.960930816251	-1.035362152482
C	3.608764736134	-2.100043125071	0.645044532255
C	3.323898596220	-3.473655813178	-2.070052069401
C	-1.088045660535	3.621793663331	-0.465897231284
C	2.885797463236	-4.330674443641	-3.112518277147
C	4.612488758228	2.515991029589	-0.945545341308
C	3.947975636865	-1.151720105730	1.641386551158
C	1.455309573970	7.990777455982	-0.870488993889
C	1.861781024759	6.687342719624	-0.717818268403
C	0.915203167965	5.650443521813	-0.535670177593
C	-0.471934499902	5.984834989853	-0.564656279469
C	-0.857028544394	7.341343191023	-0.697229563876
C	0.085939482768	8.326574926978	-0.842799912325
H	2.196620966281	8.770087638008	-1.020363344562

H	2.917963217313	6.442781409552	-0.749033312832
C	1.288313702318	4.281945187835	-0.367525348997
C	-1.433049260277	4.958756231284	-0.496744294761
H	-1.916458031576	7.584379255303	-0.701431010166
H	-2.482269094802	5.238869769346	-0.476187545819
C	5.165530831570	5.392598347582	2.936560878135
C	5.686715166349	4.532524222056	2.003182696345
C	4.880063623007	4.018596964679	0.957941980382
C	3.513469573346	4.430863326203	0.861729336115
C	3.003544467790	5.305643081541	1.852527388820
C	3.809183320675	5.771278763904	2.863604046042
H	6.439372168250	2.783183486789	0.122871804049
H	6.726412977198	4.219770374807	2.058550658878
C	5.395473685488	3.076727763606	0.041204477976
C	2.707818381114	3.894819778680	-0.190978367164
H	1.959257792168	5.592824757774	1.822503866751
H	3.391939699319	6.429025240127	3.620149714621
C	8.236839671482	-3.411510334006	0.141526926270
C	6.922491237589	-3.572312931200	-0.225459745688
C	5.894295965780	-2.838863136821	0.416274584275
C	6.259931139782	-1.905779437177	1.436911643896
C	7.622643201237	-1.779687541037	1.803518239064
C	8.592657188419	-2.516320104154	1.171536631981

H	9.009485404249	-3.972249881075	-0.375817078068
H	6.667382383921	-4.251630012891	-1.030455678921
C	4.518573402902	-2.947047593301	0.045455513781
C	5.269465187999	-1.095236520493	2.032358581898
H	7.886828270312	-1.074893808562	2.588026636826
H	5.562192960916	-0.402973270182	2.818295862609
C	4.739470291353	-8.087925851826	-0.640402381191
C	4.164750394249	-7.574300400918	-1.774893820767
C	3.906262099944	-6.186502947438	-1.891228200711
C	4.295453724623	-5.310577431608	-0.833587054427
C	4.857659493045	-5.876805475623	0.336056321676
C	5.070594994759	-7.230807059248	0.429368453218
H	2.977295451091	-6.342095337293	-3.820757858897
H	3.879103738821	-8.227684958874	-2.595257331941
H	5.108527515739	-5.231709359253	1.170902683821
H	5.492348905475	-7.646084082061	1.339866094497
C	3.234971861978	-5.663363260456	-3.012642431839
C	4.053522706857	-3.910704885766	-0.979135053822
H	-0.218597408120	9.362718943069	-0.955110015721
H	5.790396100209	5.773228320649	3.738834031305
H	9.635173373476	-2.403910771228	1.453516531139
H	4.923221667105	-9.154657457806	-0.554977894680
H	-1.093227164671	-0.964158858727	-4.791358662757

S	-5.839407394617	-0.146916074703	-0.136218411985
F	-5.641990802854	-0.285268195402	1.476152830612
F	-4.981292769235	-1.525904202844	-0.366698044607
F	-6.171163510397	-0.090860161272	-1.767862970633
F	-6.821635947518	1.146996570288	0.084664984018
F	-7.168397483114	-1.087731891260	0.004860092785
S	-0.189903674731	-2.816139024444	-8.139249208600
F	-0.575063802538	-1.309143812088	-7.615621030131
F	-1.632432438787	-3.379306388442	-7.554736798794
F	0.110485277365	-4.297840179692	-8.778357454910
F	1.162720147122	-2.230452959118	-8.845380670740
F	-0.987161279253	-2.460709178011	-9.519815766265
S	-0.055400959524	2.751913089923	4.039884568553
F	0.197097911602	3.834800944729	2.822921241427
F	1.112068979711	3.486926157113	4.929532818130
F	-0.393708386478	1.758442609839	5.298490955757
F	-1.317553380350	2.117850518039	3.202228286994
F	-1.109946415449	3.818625179527	4.677043347361
S	6.823851585822	-1.924508592986	-4.462448137578
F	6.228644398372	-3.072577613495	-3.441401134046
F	8.250959931139	-1.947804372078	-3.650772816112
F	7.457793740641	-0.877657041844	-5.552584073918
F	5.448059213162	-2.015629349653	-5.353424623249

F	7.410033335191	-3.139250398486	-5.377201601312
C	-1.039788640242	-4.147758661481	-0.382639532959
C	0.074457394487	-4.867529350573	0.423488601100
C	-0.259817974966	-6.333040966423	0.856372415570
C	0.762189053264	2.861285036026	-5.096438485408
C	-0.323565907054	3.831468502579	-4.548089846511
C	-0.588132548335	5.084480653182	-5.443744638086
F	1.978465992882	3.387028725997	-4.856736291552
F	0.607625239579	2.719926267392	-6.430122103341
F	0.069204660818	4.282609202157	-3.339115479250
F	-1.504826136360	3.179561786059	-4.420452197075
F	0.564911308488	5.692941472320	-5.752053947472
F	-1.222572103406	4.748308454145	-6.572210748056
F	-1.361769136976	5.946785823049	-4.764652885092
F	-2.213640097083	-4.283847198070	0.279714335886
F	-1.154427360528	-4.751670538666	-1.588539550437
F	0.339985373561	-4.176029660005	1.554511488277
F	1.188740093269	-4.929862895576	-0.338036130304
F	-0.624337338503	-7.071232713918	-0.203171231602
F	0.835587138771	-6.881707421244	1.401864857296
F	-1.244128386330	-6.360254312642	1.762201920985
C	-4.766074606634	-2.735570320317	-3.307161312689
H	-5.012409759963	-1.784460178033	-2.855952242894

C	-3.575230796926	-2.809686742291	-4.041214558619
C	-2.802735839038	-1.648933758687	-4.236729106996
O	-1.595165737448	-1.823217089747	-4.705627639592
C	-3.204823928156	-0.276115827574	-3.818708170679
H	-2.804549556925	-0.097953165905	-2.812405217233
H	-2.770559264971	0.468601767597	-4.489276170103
H	-4.286952662484	-0.153604159506	-3.789434192607
C	-3.033434112533	-4.141512607900	-4.468059777663
H	-3.820103916897	-4.760926835553	-4.917029977798
H	-2.222538963329	-4.029591294492	-5.185808177012
H	-2.645614769774	-4.692963610256	-3.603474382109
C	-5.236027353572	-3.926098800233	-2.519451054736
H	-6.193899950930	-3.731090050172	-2.033028424905
H	-5.314412534527	-4.836350221927	-3.120039924001
H	-4.493575286541	-4.109409845982	-1.730744108583
C	-4.294987971070	-1.800394511099	-6.635600966354
H	-3.489157000028	-1.388799665469	-7.235097900443
H	-4.281756193790	-2.874816739469	-6.498428162587
C	-6.345160031923	-2.704162461593	-4.752979964831
H	-5.884969483459	-3.529660966560	-5.286372602046
H	-7.159597472839	-2.975796195974	-4.088471917592
C	-6.328905113339	-1.430951957622	-5.286210411094
C	-5.310002079547	-1.017776543997	-6.200722466505

H	-5.292552418661	0.039701998770	-6.463533940579
C	-7.285047944874	-0.385146420531	-4.790729948154
H	-7.949928588514	-0.074281517006	-5.606818887377
H	-7.902082835680	-0.751719791584	-3.967945886342
H	-6.757567453929	0.514795666089	-4.453875904936

TS$_{min}$, Imaginary frequency = -293.20155122, -15.98016653, -7.50590247

O	2.271448978492	3.036134816212	-1.885838549045
O	0.142434189455	2.642401654033	-0.577456809315
O	1.519138556341	-1.539118020362	0.420603491725
O	2.760180340121	-1.459463422252	-1.781221480359
S	-1.267380680396	-1.778325323727	-1.099001063886
S	0.969299770854	1.956425738631	-4.503477697108
O	-1.441084308728	-1.149157650549	0.221647431947
O	-2.351887589593	-1.669592246252	-2.087239149635
O	2.426784334378	1.767531545756	-4.560939076665
O	0.175856741498	1.207039917922	-5.513959992045
N	0.145939224407	-1.510335747000	-1.803315615369
N	0.286987968570	1.838431567449	-3.074915983591
P	1.424841481267	-0.889279287414	-1.061270361908
N	1.663246165092	0.664294713757	-0.971838026422
P	1.092911717976	1.943724265764	-1.682592682928
C	4.822795509875	2.126112694116	-1.039231771198

C	5.424293515664	1.107852944625	-0.293178082188
C	6.116606795272	0.075699543592	-0.910526660967
C	6.199400803024	0.071748163240	-2.294764979957
C	5.616982710343	1.065390988152	-3.069492792572
C	4.927408293062	2.089480619177	-2.433858972514
H	5.322009268802	1.095839347402	0.787750264248
H	6.565365383274	-0.710361896645	-0.315264941343
H	5.689606971361	1.055349582650	-4.150447761295
H	4.464769812144	2.858935048498	-3.040067734561
C	-2.628036809301	3.180159104595	-1.144172939961
C	-3.704428182299	3.288081718976	-2.036382228428
C	-4.794479906443	2.432826902394	-1.958414267812
C	-4.791800110151	1.443090301740	-0.984487384836
C	-3.714891075925	1.257413126750	-0.133274922469
C	-2.637796164309	2.127927504777	-0.220714182392
H	-3.695141651085	4.056023424297	-2.803420833684
H	-5.630416229093	2.550418514053	-2.637770502323
H	-3.700337388651	0.460732687225	0.599802779179
H	-1.816422678442	1.992499459379	0.469357542996
C	2.508126676867	-3.059200094299	-4.171602604405
C	1.787285795147	-3.851240862745	-5.078948742542
C	1.550620059557	-3.430830878946	-6.379448672428
C	2.016813522392	-2.182459382942	-6.770426453663

C	2.684274735565	-1.346428451289	-5.889624984965
C	2.928191208452	-1.791770610110	-4.596863153758
H	1.400194581289	-4.815331201608	-4.763698081171
H	1.012552162481	-4.069546370315	-7.070400080722
H	3.020476935957	-0.359945773682	-6.185191259728
H	3.477150637064	-1.140095958336	-3.931166346930
C	1.853242651431	0.398347162948	2.471138471782
C	0.526643275127	-0.002248301055	2.660869414516
C	-0.446374681166	0.914297452990	3.038558793554
C	-0.081730566440	2.242603862501	3.212501086480
C	1.220436943978	2.677654792945	3.016982674362
C	2.180001693497	1.746442678946	2.646742695602
H	0.238278174145	-1.036596564405	2.515329100758
H	-1.466310642327	0.582999691905	3.192243027206
H	1.493112153754	3.718875200358	3.137764365108
H	3.194627163519	2.086221025689	2.461509007507
C	-0.231781320792	3.969149929994	-0.785670195186
C	2.813296794280	3.630217346508	-0.754601656988
C	2.746605622730	-1.502884069584	1.065526976098
C	3.020639252956	-2.822874898309	-1.682295188130
C	-1.585454342604	4.229674193328	-1.118949337194
C	2.872104101326	-3.616733811494	-2.849714645542
C	4.097699386838	3.204874629038	-0.334971217347

C	2.919028995012	-0.558622084611	2.106569353474
C	0.942611313515	8.628172017765	-1.301647387222
C	1.322204569943	7.349388599246	-0.972340837843
C	0.374671754818	6.298544812631	-0.945146658049
C	-0.977654635256	6.589033241438	-1.301219581226
C	-1.339084488912	7.921512388523	-1.614760121461
C	-0.400788058804	8.922414412918	-1.613254989848
H	1.685981604799	9.419347553688	-1.328337500837
H	2.360086076613	7.132986451249	-0.742043500773
C	0.722574834407	4.953632863861	-0.616614404667
C	-1.927000648831	5.547974007925	-1.343902503279
H	-2.373261682226	8.133053525822	-1.874027194000
H	-2.962896512149	5.803042058298	-1.549651809837
C	3.696287161225	6.202478927088	3.470926343700
C	4.432236415902	5.324782461833	2.714923420060
C	3.905599483328	4.775871400445	1.519970369959
C	2.602561905994	5.175503363095	1.083188842367
C	1.864561965406	6.070693597367	1.895632334989
C	2.397424316820	6.566958110773	3.060911487532
H	5.620941499471	3.526369146350	1.124156818189
H	5.427630307249	5.023179049640	3.031314482238
C	4.626350768817	3.808097368464	0.785991172900
C	2.070549320404	4.597862164204	-0.111407675151

H	0.857201764430	6.344262775739	1.604315745130
H	1.805517014027	7.236462018705	3.677602064484
C	7.335789579609	-2.969147270265	1.469685961959
C	6.110794181421	-3.095719508972	0.860226268916
C	5.002089789739	-2.326150836921	1.290132704916
C	5.196335230428	-1.392238769039	2.357240062810
C	6.467067680690	-1.303548063337	2.976669066362
C	7.516651614544	-2.074836683813	2.544600638617
H	8.175313351802	-3.556762273775	1.110590893549
H	5.994057614865	-3.774946040974	0.024185068369
C	3.721583792826	-2.389201236282	0.657207977707
C	4.139665489537	-0.540167577849	2.746153177800
H	6.598979819071	-0.598812426695	3.793948852557
H	4.300181884059	0.154256024483	3.567678930883
C	3.928104053630	-7.523611106778	-0.148383922347
C	3.655436587058	-6.952635915487	-1.365560797160
C	3.473622958010	-5.553339878309	-1.484872926120
C	3.615165870407	-4.729798897944	-0.326863247808
C	3.876538583474	-5.351807323715	0.917276918787
C	4.025442088248	-6.714897265758	1.002890643601
H	3.101286516587	-5.605206004828	-3.598209553353
H	3.557694191428	-7.568369054013	-2.256023053621
H	3.946568614261	-4.740319559681	1.810653833935

H	4.214466644209	-7.175236738748	1.968121420392
C	3.139877413616	-4.965452204692	-2.721068369399
C	3.450223147857	-3.319092736208	-0.465725690087
H	-0.686353693000	9.939290609593	-1.864957897150
H	4.105510303954	6.606884768358	4.391744296869
H	8.489529753009	-1.989828004397	3.019147247886
H	4.056132097798	-8.598626948343	-0.065367210748
H	-1.374729978120	0.785076629003	-5.492062182493
S	-6.270221497359	0.384626736508	-0.775179773164
F	-6.261797106085	0.565149776832	0.849502048532
F	-5.366506511272	-0.977416999837	-0.629187508795
F	-6.400565729104	0.119694526161	-2.402264881787
F	-7.289436915583	1.662810359784	-0.916083533771
F	-7.590899446742	-0.558475480440	-0.600304414959
S	1.753420218031	-1.622734292083	-8.493120705819
F	0.167422099497	-2.110152102772	-8.474467776435
F	2.177763299621	-3.088688928362	-9.100786325272
F	3.294785608422	-1.106074604528	-8.641308811205
F	1.285217939071	-0.132498529499	-8.014971013471
F	1.502183269015	-1.140367445213	-10.028988312383
S	-1.349547697218	3.465673777214	3.720084419034
F	-2.525394721270	2.734654958855	2.840131864173
F	-1.002734260840	4.439826782923	2.436427174423

F	-0.279972223618	4.301709188877	4.642318887098
F	-1.782283045726	2.576794851233	5.027171835697
F	-2.470222415552	4.562434903298	4.164337718969
S	7.106001494761	-1.291065320688	-3.119830277031
F	8.257699188381	-1.329267472888	-1.951565282659
F	8.020772209998	-0.247838849487	-3.991771846136
F	6.024283641441	-1.367663641707	-4.352703354318
F	6.245070368096	-2.436036115928	-2.305736967091
F	7.908648005818	-2.509034870765	-3.846498073557
C	-1.461816892229	-4.592121506881	-1.903593505334
C	-1.195657802380	-3.658807911741	-0.687686117233
C	-1.030157724641	-6.079941640956	-1.699390210600
C	0.711396739407	3.788270214860	-5.035067883746
C	-0.725887524946	4.099050847494	-5.545355432220
C	-1.058615036132	5.622591633958	-5.658068847651
F	0.990707669775	4.586581224199	-3.981932819167
F	1.592925535895	4.039947047313	-6.019645143908
F	-1.632382628649	3.554013247879	-4.697573073016
F	-0.888240576835	3.562004144848	-6.772454878422
F	-0.107301190677	6.261585022957	-6.351581640276
F	-2.229873658883	5.766628754211	-6.298274746501
F	-1.170162145233	6.184325559620	-4.448722550776
F	-2.786934680725	-4.597977049465	-2.179971488552

F	-0.790669671539	-4.139937576588	-2.993388152891
F	-2.132227436540	-3.901012948765	0.250699213695
F	0.020299901361	-3.952110563441	-0.173245268153
F	-1.538648143899	-6.568836216190	-0.562761347707
F	-1.498126034101	-6.810867079033	-2.729819080755
F	0.301012309485	-6.200577034167	-1.675772259720
C	-3.969697083730	-2.530666587044	-4.501116380042
H	-3.084318330913	-3.075231711273	-4.196849986325
C	-3.757206759095	-1.265778778603	-5.073744098779
C	-2.446932550834	-0.792418001541	-5.255327663554
O	-2.332160616990	0.490959374332	-5.506190395605
C	-1.194446248237	-1.572818785812	-5.086540939059
H	-0.741718347541	-1.337447288661	-4.115135551368
H	-1.361210025013	-2.646748497267	-5.138769143067
H	-0.480805192720	-1.287511610034	-5.863557452781
C	-4.896424874833	-0.316898272084	-5.319201579664
H	-5.087263349436	0.292780901534	-4.428882447921
H	-4.671292198243	0.368417823826	-6.138939644694
H	-5.821495037000	-0.853622679399	-5.549733644158
C	-5.158243976097	-2.778403647569	-3.616662566039
H	-5.238257132892	-3.830511330679	-3.336571106632
H	-4.995794993557	-2.203236663517	-2.698203865459
H	-6.103912786346	-2.448723192219	-4.053308243756

C	-3.115991392147	-1.948874274903	-7.835382210175
H	-2.493934485867	-1.257187694946	-8.395296027182
H	-4.121804332494	-1.619405943017	-7.602502207706
C	-4.538230652385	-3.826717567140	-6.092642040649
H	-5.203473195576	-3.084979583671	-6.522755463563
H	-5.022163954124	-4.591910042107	-5.494882917947
C	-3.334298732592	-4.125324855653	-6.693532047833
C	-2.666591986455	-3.190397263360	-7.546459356602
H	-1.669996964129	-3.466586100857	-7.883803140695
C	-2.579673541862	-5.364783260039	-6.309671031251
H	-3.134976941516	-5.981329746965	-5.599531844596
H	-2.366394266018	-5.968400781625	-7.200423192168
H	-1.608810007869	-5.111566823424	-5.863009448863

TS_{reg}, Imaginary frequency = -312.73505329, -22.84453636

O	2.369561281030	2.389368074583	-2.105536535232
O	0.591927355441	1.912268239466	-0.374962192505
O	2.352576890231	-2.202579534958	0.258746261863
O	2.998684048435	-2.140216352461	-2.182902225873
S	-0.775689743345	-2.400309352176	-0.565942737018
S	0.633399658039	1.001850971027	-4.373599994639
O	-0.638821692091	-1.730547070630	0.740807671799
O	-2.033155445300	-2.216933469299	-1.309774265065

O	2.002930801303	0.523104958720	-4.624401956711
O	-0.471160018820	0.354090162298	-5.129205053101
N	0.474556986022	-2.269731734961	-1.551263405801
N	0.209855119351	1.168866784983	-2.849074995153
P	1.871027068333	-1.586675962669	-1.162413640944
N	2.063086563031	-0.021416334558	-1.136159152121
P	1.307230450863	1.247282662317	-1.664965875619
C	5.070141789881	1.503543888194	-1.937230823182
C	5.870560279448	0.495096424861	-1.391688048925
C	6.400998217026	-0.509739812261	-2.186829805777
C	6.112299915212	-0.501462377786	-3.542619586842
C	5.328469085483	0.485239829583	-4.122479494794
C	4.812306767197	1.487619914458	-3.311859302568
H	6.053805596019	0.467862238480	-0.321821695808
H	7.008434826377	-1.288702872829	-1.743298443334
H	5.108547902260	0.483128900953	-5.183046727007
H	4.195328184970	2.254351752105	-3.764307014052
C	-2.248484655895	2.477292920275	-0.482558266264
C	-2.159487563791	1.318729544819	0.298811882250
C	-3.241073311464	0.454871016229	0.426905557046
C	-4.418943696288	0.757631010383	-0.238370399324
C	-4.524779273947	1.856341767652	-1.080520819070
C	-3.431118496151	2.698447299499	-1.205865157272

H	-1.254325708298	1.085371776727	0.843495500473
H	-3.145469990013	-0.428305731091	1.046700692673
H	-5.438120773857	2.062342398363	-1.625608836610
H	-3.499550670734	3.546648217274	-1.879540012388
C	2.078369927162	-3.938203179563	-4.266704734365
C	2.261541218314	-2.715494927853	-4.922118138992
C	1.541116826768	-2.402387644150	-6.068899927209
C	0.636665723268	-3.328102673983	-6.565044642981
C	0.404493901988	-4.538111560669	-5.927758237722
C	1.116203815828	-4.824240420548	-4.773123879781
H	2.980352387412	-1.994195550668	-4.557334661705
H	1.702284437235	-1.447933749746	-6.555087617983
H	-0.315605160868	-5.249283798418	-6.314333084387
H	0.910932463149	-5.753227393744	-4.250411311926
C	3.040273565768	-0.221729311723	2.192732295499
C	1.777666980018	-0.668460452446	2.598445233998
C	0.857460367365	0.207484099014	3.159454795322
C	1.214863716127	1.538554935089	3.318668260047
C	2.459826611464	2.015079635632	2.937863361918
C	3.362203164980	1.127974160745	2.370088059977
H	1.491579495434	-1.704725113355	2.470524130512
H	-0.117728326659	-0.157634919328	3.458446362712
H	2.727513522307	3.057470203721	3.058681854456

H	4.320772296422	1.506190385291	2.027915232693
C	0.191971741958	3.247168403630	-0.451118444523
C	3.168667518203	2.952658542178	-1.117707468666
C	3.691550503131	-2.080928144885	0.603931572763
C	3.348176714029	-3.488792720606	-2.092053313078
C	-1.199139506493	3.519577716000	-0.505146927844
C	2.902863591364	-4.365586980930	-3.114897111766
C	4.523058253542	2.546028484671	-1.044126438752
C	4.042659977086	-1.123911941921	1.588138622384
C	1.241662956295	7.935636299886	-1.009478368616
C	1.678607282165	6.644819909296	-0.835480354074
C	0.757128318148	5.590256252852	-0.627907601175
C	-0.637444157571	5.893128754934	-0.652486046724
C	-1.054116893446	7.237830484351	-0.807710215005
C	-0.134706689842	8.241100311840	-0.978696489478
H	1.964176971886	8.728460188976	-1.178948424952
H	2.739844767722	6.423806106156	-0.869698804329
C	1.162597553337	4.233219073549	-0.441691556370
C	-1.574977557064	4.847425569662	-0.557399328081
H	-2.118815828483	7.456554486453	-0.808899597466
H	-2.630070164476	5.104448538286	-0.532040701436
C	5.050029046889	5.470009494202	2.805602454365
C	5.580903746369	4.612923622495	1.874828761217

C	4.775311700469	4.070825174529	0.843238285103
C	3.398198726257	4.449582080091	0.759067181224
C	2.879638803767	5.324315001346	1.745138683550
C	3.685125259227	5.818383203984	2.742738298616
H	6.355868446968	2.869062524166	-0.001849078689
H	6.628124595999	4.324865500456	1.921858992763
C	5.303564924338	3.134416617643	-0.071987790104
C	2.592819915504	3.881602740128	-0.276871775218
H	1.829095239215	5.588041284946	1.722993928983
H	3.261309700896	6.474948380064	3.496634886730
C	8.325976007618	-3.302439347801	-0.042270876888
C	7.004062073364	-3.491004712426	-0.366249634325
C	5.982558581161	-2.776093949506	0.306346876081
C	6.361716352166	-1.832330345359	1.312089226345
C	7.732647973465	-1.677889933958	1.634478527204
C	8.696462552614	-2.397008078985	0.973513773994
H	9.092854084753	-3.848896645922	-0.582938262521
H	6.736881227529	-4.177304712657	-1.161392918688
C	4.598203822803	-2.913414242702	-0.020134019755
C	5.374392641043	-1.040572541692	1.937455670017
H	8.007747053784	-0.965463743724	2.408246676173
H	5.677785473200	-0.341199610903	2.712982417645
C	4.898163103000	-8.056176007748	-0.653383588470

C	4.280351443798	-7.567636404180	-1.776298251755
C	3.993186106563	-6.186356394287	-1.901947696642
C	4.398002454516	-5.289889335665	-0.867624680391
C	5.005822784266	-5.830700289698	0.291127012044
C	5.245862250770	-7.179426476797	0.394902422093
H	3.015267405903	-6.382564008664	-3.803807550745
H	3.982111857644	-8.236641659812	-2.579388236864
H	5.269515445159	-5.170627148850	1.110108425305
H	5.701951739633	-7.575293663022	1.297462598689
C	3.280496029919	-5.689898164265	-3.010058985264
C	4.121644677741	-3.896838772776	-1.020539106099
H	-0.463310635819	9.267804444962	-1.108453244644
H	5.674397784172	5.872241189921	3.597654725279
H	9.745016898818	-2.262728592520	1.221433967228
H	5.103502090433	-9.118303532934	-0.560153759910
H	-1.115316881150	-1.087722869350	-4.754157550919
S	-5.890153870788	-0.297275972078	0.024721659842
F	-5.688869855307	-0.322492277652	1.642593213374
F	-5.016770313297	-1.680544055620	-0.109428387395
F	-6.218873690150	-0.355169563112	-1.607649740925
F	-6.885289034491	0.997917778645	0.158510947648
F	-7.207622713556	-1.239600589675	0.229905590742
S	-0.274295999660	-2.948412937546	-8.107026219893

F	-0.664697487018	-1.438870536013	-7.594988132538
F	-1.702555917328	-3.516109826921	-7.492257541750
F	0.029073459406	-4.435470799749	-8.731931937542
F	1.061365123379	-2.359532582497	-8.842265394601
F	-1.096509636204	-2.617652042535	-9.478513387625
S	0.010122054649	2.712212708015	4.045738660497
F	0.218705306543	3.785315857265	2.812007325342
F	1.173781935877	3.484240433722	4.908247738337
F	-0.287103909558	1.726990279878	5.320805508481
F	-1.249459454030	2.039373538911	3.234031781645
F	-1.058887159330	3.762413218933	4.685998512157
S	6.779905709348	-1.848969358115	-4.588777673834
F	6.201775523338	-3.006211870565	-3.567948079952
F	8.211875344985	-1.860540286114	-3.785543756320
F	7.396582345638	-0.791826487721	-5.678811389408
F	5.400318600505	-1.951507168070	-5.472467482447
F	7.373269274992	-3.054612876476	-5.510742555952
C	-0.889746705254	-4.286568501976	-0.204343650862
C	0.280419011549	-4.945223086275	0.572674201839
C	0.025100186103	-6.416653508871	1.038461540128
C	0.625221264016	2.766579826354	-5.140325706139
C	-0.480202160310	3.710969582195	-4.587416384861
C	-0.787035461979	4.951728108399	-5.486676248706

F	1.829489111178	3.325728396797	-4.916929420180
F	0.460828728418	2.611484411824	-6.471131244511
F	-0.087717644012	4.178229952039	-3.384788358804
F	-1.642830036010	3.028626032181	-4.444917191636
F	0.345919535549	5.589569205349	-5.809195321833
F	-1.423072336005	4.592606411628	-6.607104817546
F	-1.577137920816	5.795923375851	-4.803832246804
F	-2.032569462965	-4.460056297538	0.501599259578
F	-1.020933593750	-4.914987344648	-1.396209164886
F	0.555051667889	-4.224508949858	1.683358998429
F	1.369016772024	-4.972426207927	-0.226688601340
F	-0.349844109879	-7.184918757340	0.004389779732
F	1.163835201901	-6.910941568256	1.546323167279
F	-0.920107142009	-6.470971162149	1.983656887330
C	-4.629414669668	-2.935685481693	-3.005614144736
H	-4.820760762855	-2.000731442880	-2.499198913456
C	-3.505122721373	-2.983972878603	-3.838150157236
C	-2.766756753056	-1.804055614739	-4.076650235734
O	-1.592191052209	-1.957295042058	-4.627399933214
C	-3.160833152074	-0.449261224218	-3.601973143801
H	-2.730378222167	-0.300165240848	-2.603657075354
H	-2.755455057537	0.321357292676	-4.261206511822
H	-4.243143159634	-0.337813963722	-3.528407334744

C	-2.969384047259	-4.293233133667	-4.332301022034
H	-3.780894446966	-4.971195210515	-4.619874411203
H	-2.306048301418	-4.153334249908	-5.184523857471
H	-2.395648343200	-4.788644087971	-3.539118796142
C	-5.042637929395	-4.147823893096	-2.219293245093
H	-5.153431426585	-5.045743249094	-2.832932002771
H	-4.255606977219	-4.343311207748	-1.478490076321
H	-5.971139780994	-3.969628670770	-1.672882214579
C	-4.362544652003	-1.961459678704	-6.309213618567
H	-3.562059451273	-1.601648642903	-6.948855498262
H	-4.417729535297	-3.034461593797	-6.178153714533
C	-6.298664362272	-2.808483960382	-4.305794079411
H	-5.942059979758	-3.669947553427	-4.863082785459
H	-7.067706125538	-3.020716589915	-3.569583135502
C	-6.267604698094	-1.549288452860	-4.866983057832
C	-5.326290970058	-1.116941951721	-5.844279784535
H	-6.896362582732	-0.781813419494	-4.415710179196
C	-5.330512945323	0.338158967408	-6.237588080474
H	-6.184009312872	0.548932350293	-6.892534359717
H	-5.424186701188	0.991952771849	-5.363627320275
H	-4.419659959242	0.608781271862	-6.776855283446

TS (for KIE study), Imaginary frequency = -289.99539023, -33.52898863

O	2.462634224852	2.427414648968	-2.048225023637
O	0.663427383081	1.966281704091	-0.338566587531
O	2.290134131101	-2.194396218962	0.260002502344
O	3.019007998705	-2.119006664367	-2.156193993441
S	-0.821799386808	-2.274684957913	-0.688957506605
S	0.710230700376	1.075691076369	-4.342171021598
O	-0.699535743292	-1.645180190709	0.639796353873
O	-2.039323295917	-2.008877314793	-1.471503313704
O	2.075043728517	0.578715472424	-4.584747031677
O	-0.397269775060	0.443441942724	-5.104533245436
N	0.474401969821	-2.188856439314	-1.618826101972
N	0.282811269925	1.254072339086	-2.820249100726
P	1.871059297623	-1.547609740031	-1.168366139462
N	2.103984624695	0.011936524549	-1.111582427263
P	1.374533914470	1.300397595936	-1.630584634913
C	5.138043904155	1.462615846264	-1.843664472723
C	5.911307256226	0.438584071537	-1.287629112534
C	6.432050221229	-0.577048942766	-2.075177237849
C	6.159922140040	-0.564677658860	-3.434313980860
C	5.400089098601	0.434853792899	-4.023990600170
C	4.894172640386	1.448671261593	-3.220920600491
H	6.080285985160	0.408941920991	-0.215492406161
H	7.019601083148	-1.366807432728	-1.623919877283

H	5.191755371573	0.434722063673	-5.086961563591
H	4.296527986056	2.225717467003	-3.681552827265
C	-2.167066907223	2.588286230360	-0.473936545503
C	-2.100987795246	1.401005857197	0.265091686942
C	-3.193930162816	0.546728927181	0.350665995206
C	-4.362324548239	0.889826712192	-0.310665469907
C	-4.449641589554	2.024700766729	-1.105767405002
C	-3.343682419912	2.855365519653	-1.192018952723
H	-1.203883121561	1.133395492291	0.806705172777
H	-3.112635299891	-0.362237750608	0.933833453247
H	-5.357745860141	2.266695484658	-1.644780886680
H	-3.397328699628	3.729267837611	-1.833326189239
C	2.090719115035	-3.889190155118	-4.263813661759
C	2.291428786012	-2.661964661842	-4.905554022404
C	1.585748050838	-2.331481740621	-6.056720655678
C	0.678979935862	-3.244695121894	-6.571142819339
C	0.428086651636	-4.457900387861	-5.946920789183
C	1.124560235245	-4.760797111730	-4.787587041777
H	3.011710795169	-1.950049891211	-4.525838166772
H	1.760054944528	-1.373562268776	-6.531393981816
H	-0.294791923752	-5.158509016727	-6.347366859321
H	0.901969114417	-5.690554365552	-4.273583558405
C	2.955793057806	-0.228369788793	2.219525493896

C	1.675241019619	-0.652831000259	2.591711077856
C	0.759964657616	0.236913864405	3.139171748801
C	1.139963806789	1.559277449024	3.318141079704
C	2.400753787222	2.014516818504	2.965033873774
C	3.297769369383	1.114585937219	2.409562631263
H	1.372341966410	-1.682614575159	2.449645652577
H	-0.228025522806	-0.111495139694	3.415504602564
H	2.685906842594	3.050505830554	3.099027022076
H	4.269918965935	1.477310209556	2.089403752783
C	0.288512907904	3.308958039503	-0.403496762299
C	3.263095379759	2.959305453949	-1.044045710753
C	3.619910098481	-2.097366149282	0.646897359032
C	3.332032688260	-3.476414779798	-2.064965961540
C	-1.096962467870	3.609656330359	-0.462980441853
C	2.893179107528	-4.336702088900	-3.104272156946
C	4.605573776585	2.517142046371	-0.955891117857
C	3.958703368860	-1.147258051123	1.641720598785
C	1.434508786518	7.984943170139	-0.876038937590
C	1.844615471270	6.682358927599	-0.725986003645
C	0.901187585576	5.643166706622	-0.540501604959
C	-0.486819695081	5.974298628539	-0.563398054401
C	-0.875604461766	7.330028232007	-0.693407918239
C	0.064470937396	8.317539252500	-0.842237466575

H	2.173374321078	8.766085993087	-1.028403716631
H	2.901220582769	6.440221322813	-0.761620490020
C	1.278224149523	4.275649654011	-0.373449539349
C	-1.445129078720	4.945892891718	-0.491522896316
H	-1.935597250698	7.570665499767	-0.692726700408
H	-2.494825517873	5.223738011290	-0.465807654734
C	5.159149913920	5.398815511033	2.922335306637
C	5.680444648112	4.538780854827	1.988983846947
C	4.873055868972	4.022182684801	0.945636093760
C	3.505438417489	4.431494538739	0.851313496163
C	2.995488785353	5.306272248898	1.842089023276
C	3.801929552079	5.774674812292	2.851258976033
H	6.433474124936	2.788835294882	0.109552246803
H	6.720844063286	4.228127704148	2.042902128485
C	5.388908732347	3.080344902183	0.029125188868
C	2.699168277918	3.892558248287	-0.199525883001
H	1.950538411842	5.591154907986	1.813768040448
H	3.384631464251	6.432357257222	3.607835478746
C	8.250350165785	-3.397226237432	0.135052050853
C	6.935680943636	-3.561676953685	-0.229144491472
C	5.906915282989	-2.830454764904	0.414240985188
C	6.272216417591	-1.895549975047	1.433311756974
C	7.635331993454	-1.765799680580	1.797150874310

C	8.605928964462	-2.500467956531	1.163787314546
H	9.023389045594	-3.956326485588	-0.383475178348
H	6.680607756626	-4.242142520372	-1.033199126491
C	4.530724591695	-2.942703550629	0.046462945524
C	5.280822284623	-1.087124389803	2.030090462904
H	7.899310327188	-1.059782781122	2.580633671733
H	5.573317125759	-0.393568587593	2.814967862181
C	4.764964674421	-8.084098483993	-0.631198317476
C	4.185812696492	-7.574088853275	-1.765063713906
C	3.923009252744	-6.187250526296	-1.883094456889
C	4.312376559140	-5.308327083790	-0.827994953592
C	4.879218787905	-5.870950652915	0.341161524245
C	5.096327292556	-7.224172065766	0.436233831126
H	2.988041861773	-6.349026629277	-3.809315805910
H	3.899794141133	-8.229700470293	-2.583512016129
H	5.130276422532	-5.223760165500	1.174323536112
H	5.521520898759	-7.636669186824	1.346401690897
C	3.246703262139	-5.668125600282	-3.003338599814
C	4.065959046767	-3.909377740352	-0.975334993692
H	-0.242916822537	9.353084318942	-0.952309258620
H	5.784625457290	5.781529090086	3.723138820059
H	9.648713528715	-2.385284696045	1.443655230183
H	4.951933903637	-9.150161077769	-0.544388836054

H	-1.088773972175	-1.000084380251	-4.756792595628
S	-5.841806041329	-0.165633168831	-0.109012646814
F	-5.626902067979	-0.312901937138	1.500309976556
F	-4.986353131078	-1.542717824591	-0.357105239687
F	-6.191805566982	-0.099535316390	-1.736476470283
F	-6.820612768362	1.127969051658	0.130099012198
F	-7.169343043755	-1.106321537532	0.041278768925
S	-0.210689864936	-2.846416010573	-8.120095255885
F	-0.606023335695	-1.341104433817	-7.596379492537
F	-1.645973402343	-3.420159104541	-7.530464868435
F	0.099583740878	-4.325888849873	-8.759159649107
F	1.134467112370	-2.250033491918	-8.831812070636
F	-1.015656586772	-2.497919891128	-9.498297449346
S	-0.051344677195	2.749515612228	4.040009100644
F	0.201249451716	3.833625504376	2.824136512747
F	1.114202408039	3.485337853051	4.931532473562
F	-0.390062489216	1.754942357698	5.297513342322
F	-1.311616149395	2.114441986413	3.200412228890
F	-1.107889336428	3.814657630431	4.676577333654
S	6.819985937620	-1.922624477682	-4.471539014380
F	6.228506966143	-3.070724309695	-3.448403094210
F	8.248383810210	-1.942403256885	-3.662005340021
F	7.450376723116	-0.875742669777	-5.563759006971

F	5.443033728382	-2.017178663483	-5.360385677535
F	7.407047252875	-3.137220217632	-5.385947255032
C	-1.030338945988	-4.159812466874	-0.378722594039
C	0.087528119578	-4.882387887805	0.419865135424
C	-0.242345816686	-6.351007151976	0.845134161650
C	0.731271037408	2.842689841813	-5.100450148510
C	-0.367646383460	3.798397503840	-4.552980817920
C	-0.649720230836	5.046951699049	-5.449540482354
F	1.940109626332	3.385877220739	-4.862304467984
F	0.577540115848	2.697602319332	-6.434065622256
F	0.018223086442	4.255560021387	-3.344235913090
F	-1.540434641287	3.130527653018	-4.425615379707
F	0.494222154266	5.671250001966	-5.759216458738
F	-1.280344765975	4.701009639659	-6.577478982449
F	-1.435157866368	5.898907060200	-4.770814339532
F	-2.201620097886	-4.300477769424	0.286585478531
F	-1.148199513977	-4.756157186637	-1.587921019716
F	0.355230883466	-4.196849303941	1.554008800128
F	1.199864708363	-4.938162061925	-0.345179385794
F	-0.606701876440	-7.084251621561	-0.217990896223
F	0.855501395112	-6.899894754136	1.385669297115
F	-1.224959787071	-6.385995038048	1.752481065002
C	-4.801268325813	-2.723523665877	-3.311440662275

H	-5.039657740538	-1.773179903136	-2.854959729661
C	-3.595558075172	-2.815541465259	-4.015062166322
C	-2.805722758477	-1.664907341167	-4.206016873607
O	-1.595450724843	-1.854366117310	-4.663986177615
C	-3.191020092680	-0.288946997651	-3.783982495792
H	-2.780866145582	-0.115583974576	-2.780942943865
H	-2.755165757419	0.452869847081	-4.456887674207
H	-4.271654527725	-0.156963217411	-3.743458240260
C	-3.068577763636	-4.154093602186	-4.439558302395
H	-3.854045823753	-4.754557041926	-4.915512761060
H	-2.236499697944	-4.051777878554	-5.134197951244
H	-2.715566327821	-4.719435779455	-3.569030884519
C	-5.332142405675	-3.914155263730	-2.563967421658
H	-6.297703176041	-3.696667241795	-2.102649105867
H	-5.424473133090	-4.807505079066	-3.187862644347
H	-4.622585600618	-4.144479670849	-1.757665448132
C	-4.240873923725	-1.766249245121	-6.599118523839
H	-3.393595422568	-1.416804180711	-7.180847468112
H	-4.298520613718	-2.835919944625	-6.449641606155
C	-6.331562607626	-2.612381322465	-4.817930257215
H	-5.875846836517	-3.441585684879	-5.347766597981
H	-7.171941806707	-2.877127425754	-4.183808204326
C	-6.277436007516	-1.328340130369	-5.323749631595

C	-5.222596442471	-0.908452634325	-6.214008699155
C	-7.241851611825	-0.299440365166	-4.808359957914
H	-7.833209456412	0.117817140573	-5.632983815190
H	-7.929591187709	-0.727973137225	-4.077205819396
H	-6.727998649299	0.546737633450	-4.337088514502
C	-5.140957177877	0.541585108116	-6.618180938474
H	-6.042278733527	0.849144209833	-7.160238882090
H	-5.048867477176	1.204635892831	-5.750052398970
H	-4.280340809953	0.714952827119	-7.267253747371

Reactant (for KIE study)

O	2.411729	2.373461	-2.079239
O	0.640201	1.896152	-0.342355
O	2.375420	-2.223245	0.290852
O	3.081844	-2.158914	-2.131877
S	-0.746059	-2.356254	-0.639594
S	0.670493	0.943914	-4.327132
O	-0.626289	-1.696150	0.674264
O	-1.966741	-2.112278	-1.426477
O	2.045642	0.490487	-4.587109
O	-0.428505	0.258597	-5.063247
N	0.546053	-2.277038	-1.576161
N	0.254940	1.130064	-2.804732
P	1.932072	-1.600742	-1.140194
N	2.126553	-0.034789	-1.099074
P	1.359515	1.227502	-1.626911
C	5.110419	1.471554	-1.891649
C	5.900274	0.463048	-1.330883
C	6.437891	-0.547018	-2.114094
C	6.166674	-0.544431	-3.473440
C	5.389606	0.439292	-4.067486
C	4.865563	1.447121	-3.268444
H	6.070690	0.441431	-0.258717
H	7.038597	-1.324650	-1.659166
H	5.183042	0.431815	-5.130755

H	4.254238	2.212257	-3.731590
C	-2.212241	2.464816	-0.447273
C	-2.106770	1.269252	0.274256
C	-3.182947	0.395326	0.378842
C	-4.377305	0.728191	-0.239506
C	-4.507643	1.875566	-1.010687
C	-3.416381	2.722241	-1.123383
H	-1.191560	1.009283	0.788512
H	-3.068325	-0.520912	0.944582
H	-5.437199	2.114385	-1.513727
H	-3.506233	3.603351	-1.750486
C	2.118625	-3.968535	-4.179191
C	2.332414	-2.771228	-4.870003
C	1.596005	-2.461212	-6.007240
C	0.640390	-3.361713	-6.452295
C	0.371664	-4.540462	-5.771878
C	1.107117	-4.827475	-4.632264
H	3.088727	-2.071089	-4.540891
H	1.783128	-1.529714	-6.527344
H	-0.394546	-5.225545	-6.114110
H	0.880589	-5.732146	-4.076280
C	3.024557	-0.227274	2.224386
C	1.756016	-0.673474	2.611962

C	0.825554	0.204289	3.152918
C	1.178781	1.536615	3.310969
C	2.428106	2.013043	2.945062
C	3.340402	1.124567	2.395790
H	1.474402	-1.711403	2.486489
H	-0.153614	-0.160374	3.439424
H	2.692227	3.056480	3.063972
H	4.303055	1.502753	2.064927
C	0.234748	3.230376	-0.420349
C	3.212050	2.936738	-1.090385
C	3.707804	-2.098012	0.663387
C	3.410771	-3.512908	-2.024333
C	-1.157898	3.503323	-0.460539
C	2.952423	-4.395088	-3.035038
C	4.563648	2.523414	-1.009745
C	4.039409	-1.132183	1.645235
C	1.282734	7.922089	-0.962748
C	1.720467	6.629425	-0.805548
C	0.800228	5.574834	-0.592803
C	-0.593736	5.879158	-0.594553
C	-1.011060	7.225731	-0.732282
C	-0.092743	8.229033	-0.908498
H	2.003626	8.715494	-1.136303

H	2.780841	6.407173	-0.856207
C	1.205337	4.216996	-0.415057
C	-1.530049	4.833502	-0.493871
H	-2.075294	7.446028	-0.715061
H	-2.583433	5.093653	-0.449211
C	5.091531	5.459149	2.830971
C	5.622082	4.599080	1.902928
C	4.816334	4.054628	0.872622
C	3.439979	4.435221	0.785611
C	2.921149	5.311396	1.770486
C	3.726593	5.807209	2.767246
H	6.394406	2.843498	0.036737
H	6.668944	4.310007	1.951319
C	5.343477	3.112960	-0.037618
C	2.635833	3.866835	-0.251414
H	1.870570	5.575014	1.748367
H	3.302683	6.465407	3.519666
C	8.355418	-3.309627	0.101240
C	7.039456	-3.504859	-0.242307
C	6.005554	-2.788321	0.409265
C	6.365152	-1.835716	1.413841
C	7.730299	-1.675110	1.757248
C	8.706558	-2.395930	1.116641
H	9.132750	-3.857261	-0.423049
H	6.785912	-4.197777	-1.036419
C	4.627874	-2.931996	0.061045
C	5.365030	-1.042209	2.016363
H	7.991160	-0.956479	2.530222
H	5.653258	-0.335350	2.790869
C	4.909945	-8.082907	-0.540849
C	4.296258	-7.598480	-1.667796
C	4.020536	-6.216009	-1.804937
C	4.430870	-5.314571	-0.776476
C	5.033218	-5.851288	0.387107
C	5.263670	-7.200866	0.500936
H	3.028954	-6.420816	-3.700679
H	3.993472	-8.271409	-2.465855
H	5.301633	-5.187091	1.201193
H	5.717106	-7.593211	1.406365
C	3.310605	-5.723216	-2.916732
C	4.166843	-3.920599	-0.941061
H	-0.421715	9.257239	-1.024676
H	5.715900	5.863447	3.621964
H	9.750584	-2.256865	1.380525
H	5.107146	-9.145807	-0.439322
H	-1.109872	-1.083291	-4.669193

S	-5.830897	-0.360346	-0.017757
F	-5.520963	-0.598687	1.564825
F	-4.971891	-1.705972	-0.397765
F	-6.272964	-0.204588	-1.614439
F	-6.806042	0.904375	0.351808
F	-7.135113	-1.326852	0.152686
S	-0.288039	-2.993608	-7.985891
F	-0.601795	-1.453164	-7.512374
F	-1.718293	-3.483565	-7.322863
F	-0.055008	-4.508511	-8.571790
F	1.054471	-2.481319	-8.767113
F	-1.126191	-2.669264	-9.348624
S	-0.035479	2.711467	4.019889
F	0.195337	3.786155	2.791155
F	1.116267	3.480125	4.901584
F	-0.354154	1.725460	5.288920
F	-1.282524	2.041778	3.187525
F	-1.112167	3.763139	4.644267
S	6.853020	-1.894201	-4.504359
F	6.265388	-3.048609	-3.485760
F	8.273962	-1.896603	-3.682286
F	7.480010	-0.839612	-5.590829
F	5.485386	-2.005465	-5.406310

F	7.462359	-3.101667	-5.412830
C	-0.942118	-4.234772	-0.284208
C	0.197547	-4.931490	0.506800
C	-0.108818	-6.395764	0.963304
C	0.603841	2.693683	-5.120992
C	-0.522571	3.613589	-4.566378
C	-0.878128	4.829692	-5.481197
F	1.794130	3.287506	-4.916022
F	0.430001	2.516102	-6.447256
F	-0.129149	4.109701	-3.375954
F	-1.663278	2.899674	-4.398616
F	0.231176	5.494313	-5.829969
F	-1.518623	4.432483	-6.586313
F	-1.683448	5.661317	-4.801253
F	-2.096696	-4.362163	0.408495
F	-1.081157	-4.857663	-1.476885
F	0.477173	-4.222417	1.623603
F	1.297278	-4.988732	-0.276301
F	-0.479155	-7.152001	-0.081124
F	1.003592	-6.921309	1.497362
F	-1.077630	-6.425190	1.885248
C	-4.905512	-2.672700	-3.341452
H	-5.251970	-1.675666	-3.106439

C	-3.648936	-2.834951	-3.821217
C	-2.814152	-1.680737	-4.005070
O	-1.665096	-1.921301	-4.532878
C	-3.126694	-0.287949	-3.601230
H	-2.482052	-0.042648	-2.747139
H	-2.872682	0.398023	-4.412967
H	-4.161409	-0.147887	-3.302995
C	-3.059566	-4.170812	-4.174491
H	-3.642880	-4.985446	-3.743591
H	-3.018526	-4.302802	-5.261896
H	-2.034523	-4.242720	-3.804331
C	-5.861425	-3.771593	-3.049280
H	-6.874771	-3.387357	-2.920669
H	-5.862632	-4.542325	-3.825468
H	-5.572133	-4.259187	-2.106726
C	-4.139627	-1.292693	-6.829025
H	-3.234317	-1.059348	-7.380237
H	-4.307682	-2.335412	-6.579982
C	-6.893769	-1.815537	-5.878588
H	-6.547249	-2.575600	-6.571392
H	-7.801017	-2.027060	-5.320196
C	-6.260862	-0.641026	-5.744958
C	-5.009463	-0.334352	-6.479973
C	-6.830230	0.435797	-4.857318
H	-7.170662	1.292221	-5.451766
H	-7.682015	0.062219	-4.284288
H	-6.085582	0.822817	-4.152155
C	-4.725977	1.110332	-6.798722
H	-5.538518	1.552805	-7.387342
H	-4.632028	1.714110	-5.887225
H	-3.797143	1.207748	-7.365261

Transition state for the thermal synthesis of *rac*-**4r**. Imaginary frequency: -442.90

C	-1.36414301810991	0.61955208917797	-0.85198851866758
C	-1.81858909645916	-0.35293098835880	0.11294076798328
C	-1.30887230845492	-1.65260964911026	0.09503159440903
C	-2.72677456204169	0.07934948299537	1.22941883281656
H	-1.74503798192695	-2.37720254142104	0.79131861667004
H	-0.97469891584019	-2.08897446333721	-0.85031349047514
C	-0.40531024560635	0.29866625450157	-1.77441323454932
C	-1.82299756915805	2.04687264178384	-0.70924746457287
H	0.00151106027295	1.06783831593637	-2.43910821200217
H	-0.09758243506747	-0.72660031479267	-1.97722087390767
H	-2.27211879618440	0.89292695684187	1.81710624134515
H	-2.94538140524446	-0.75699697205789	1.90997841755357
H	-3.69003744064098	0.45204267699481	0.83694236790379
H	-2.92327542142911	2.12363002225121	-0.72124631964956
H	-1.42925991165086	2.67519111710479	-1.52202182735583
H	-1.47282568768517	2.45976731026339	0.25275059473809
C	1.37441758574673	-0.66683858199326	0.19224119612376
C	0.60527270383065	-1.63407356279478	0.86719484550928
C	1.19113814407990	0.71014803562012	0.64867697626717
C	2.40647720405846	-1.05566182762363	-0.82898610819957
H	3.30440241101470	-1.49347453418083	-0.34953774621047
H	2.74067423841046	-0.20117217517962	-1.43465243461927
H	2.02895119309465	-1.82318090434969	-1.52738472765535
C	0.95534102477617	-3.09777917748743	0.77142574137429
H	0.22723357309966	-1.28855737680987	1.83612711278837
H	1.94448322520573	-3.27875117594959	1.23015918372379
H	1.01514521460846	-3.45131029305724	-0.27099565005737
H	0.22484155529737	-3.72823625191559	1.30129232104825
C	2.11592905882173	1.80168790260596	0.13658052716855

H	2.02287196801818	1.93250533817815	-0.95411031760334
H	3.17168995189302	1.55925974735283	0.34760609926118
H	1.85522166631465	2.74604048013793	0.63426246582675
O	0.29551565015620	0.98761886067326	1.45611843321458

Reactant for the thermal synthesis of *rac*-**4r**.

C	-1.71674916499055	0.72964188607739	-0.32143080946048
C	-2.11386325183653	-0.06228210165406	0.87001091630898
C	-1.33327703271464	-0.12239200833609	1.97057340006053
C	-3.40147176253230	-0.84360902955783	0.79315579152695
H	-0.38266492882666	0.41192590181295	2.04911882132190
H	-1.64553830246190	-0.71515493421543	2.83764469703113
C	-0.84076535984374	1.75210833478123	-0.23997220187612
C	-2.29427675221485	0.32050060819001	-1.65220762680177
H	-0.44551146063547	2.10184107492849	0.71669726653755
H	-0.54660762222256	2.30614642726289	-1.13744901650497
H	-3.36903688780464	-1.60684626110207	-0.00437972261021
H	-4.25903598594187	-0.18618038376543	0.56466999953113
H	-3.60822152792402	-1.35659011617257	1.74404660151022
H	-1.88827655741763	0.93650691946421	-2.46843160346371
H	-3.39343900967952	0.42116038339101	-1.66646029569452
H	-2.07496840656127	-0.73900908341267	-1.87694408681390
C	1.37457156396134	-0.27010162632702	-0.29427863842024
C	0.97047522283480	-1.31810839130342	0.46625034144820
C	2.03050550486958	0.86245564503811	0.43174423212049
C	1.32530351750322	-0.25015208145889	-1.79946111088773
H	1.04031202326353	0.73715014560750	-2.19336713819034
H	0.60351958771042	-0.97957459179144	-2.19418961033654
H	2.31043019836071	-0.49947041139324	-2.23508268633443
C	0.34514980806199	-2.59143422414515	0.01089948931244
H	1.16666050129375	-1.23076318844727	1.54107192136481

H	0.04810443394124	-2.58429501849823	-1.04829426561561
H	-0.54689164034245	-2.81648366344246	0.62037922222250
H	1.04182759574197	-3.43783383309828	0.15967616802782
C	2.80874874420454	1.86907205679085	-0.39341378931432
H	3.34406968971915	2.55035900239988	0.28164386030709
H	2.12055231776323	2.45626186906356	-1.02495972795014
H	3.51928674356305	1.37133450185420	-1.07385036135454
O	1.95388382665808	0.98926036595933	1.64622884639785

Transition state for the $AlCl_3$-catalyzed synthesis of *rac*-**4r**. Imaginary frequency: -243.91

C	-2.55888595329312	-0.70377126925101	0.27530217188491
C	-2.60425894978253	-0.10817159310781	-1.04454779348665
C	-2.91318188868232	1.21911380817348	-1.23052654422261
C	-2.18321608293835	-0.93139295375016	-2.22055083355853
H	-3.00686538156887	1.59164857883201	-2.24305775652284
H	-3.44098981598084	1.78691795809411	-0.47580186768260
C	-2.78658619959966	0.03283482531309	1.39103786292984
C	-2.15408752054942	-2.14415893669643	0.39372591723043
H	-2.67343669207255	-0.41482660504773	2.37160515026600
H	-3.17026630814116	1.04176299587560	1.36591376708690
H	-2.27642465597745	-0.37092343424333	-3.15046776652661
H	-2.79558255074411	-1.83487405256625	-2.29983753068506
H	-1.14594764059180	-1.26587143369944	-2.11826378766809
H	-2.07409678864342	-2.44640510647702	1.43689009796140
H	-1.19141245101622	-2.33650416842589	-0.08630670180575
H	-2.88915181682625	-2.79077004364746	-0.09602432607686
C	-0.91385405540281	2.18269399920798	0.60631070612102
C	-1.06180088629992	2.31652644185227	-0.77588926756578
C	-0.14174795890201	1.08795218939967	1.08212323562759
C	-1.51711940767937	3.21183617471553	1.51716419560668

H	-0.97330273880946	4.16102982394505	1.45925058472545
H	-1.53182393958004	2.90276802184568	2.55932329780843
H	-2.55099594179100	3.42932102082895	1.22812114298588
C	-1.46108789548825	3.61861463763219	-1.40180666376505
H	-0.45101060453807	1.66206850325110	-1.38570138321158
H	-1.60503996279986	3.51948696834973	-2.47727990286223
H	-0.65351816426486	4.34342940997857	-1.24266039174746
H	-2.36534307852990	4.04681726658087	-0.96523883017549
C	0.28549946145536	0.93318623090314	2.50484275321215
H	1.32485124041663	1.27665955851071	2.57760356034744
H	0.28730868323495	-0.12347912336409	2.77589461333845
H	-0.31660517190461	1.49817782981556	3.21047636286071
O	0.31791394567716	0.24654089861303	0.22615754863441
Al	1.69929067090493	-0.92616176252816	0.17380009221160
Cl	1.35314518381151	-2.44693098962741	1.63713626979054
Cl	3.43023155849295	0.24525136979537	0.58653483108361
Cl	1.59995508430490	-1.69229882658158	-1.81419067855021

Reagent for the $AlCl_3$-catalyzed synthesis of *rac*-**4r**.

C	-2.62367101695168	-0.89283721145110	0.23038333997309
C	-2.86632180260158	-0.14101753949490	-1.02245408625617
C	-3.82132661713959	0.79464936614629	-1.10451623150422
C	-2.03101290952426	-0.51189400207531	-2.21626912275383
H	-3.99287948708784	1.33547489475667	-2.02901822391794
H	-4.47616479322228	1.01056529639745	-0.26877912707771
C	-2.70003784648575	-0.29911202245314	1.42858108726283
C	-2.26905293671775	-2.34574351312037	0.09471907616757
H	-2.54170912646839	-0.86308931627888	2.34095163400435
H	-2.92901140005370	0.75696025298978	1.51873444401017
H	-2.19999569777859	0.18167924559656	-3.04159392588016

H	-2.27941862424470	-1.51849280970853	-2.56654971736210
H	-0.96525743374164	-0.53119100858624	-1.97267021110842
H	-2.17268980680472	-2.81798248098468	1.07127360449865
H	-1.31907133750839	-2.47349476027586	-0.43313310865322
H	-3.03169441525475	-2.87607853318604	-0.48513987926941
C	-0.63382977922407	2.38104694689253	0.53553448427487
C	-0.77017416557062	2.48324889924444	-0.80601989683571
C	0.21509205417679	1.31702536508482	1.05282127892112
C	-1.28392798992801	3.33149235882808	1.49967832208309
H	-0.57823178119095	4.09066589172062	1.85312675192225
H	-1.67221224260554	2.81328955794387	2.37824526214726
H	-2.11425690782535	3.85495758137262	1.02755539495250
C	-1.54844713164194	3.53183565846838	-1.51498998969673
H	-0.24023192065801	1.75588317196873	-1.41166703690649
H	-1.58486890242426	3.34406623220851	-2.58700074051489
H	-1.09772619151587	4.51887950429331	-1.35728287137939
H	-2.57244632468275	3.58827505889334	-1.13646776758067
C	0.56178357061890	1.27450632876812	2.49930518770592
H	1.49839629890897	0.74187574923784	2.65775560975417
H	-0.22797273792405	0.71346540410032	3.01456806812366
H	0.60708801216699	2.27126854048050	2.93588572645724
O	0.65564193350414	0.43684494346638	0.27007022742695
Al	1.74649079284185	-1.04658461703834	0.43848163217960
Cl	0.89729518140064	-2.19775768881992	2.01777017751289
Cl	3.65317895181736	-0.23333725871495	0.90754491260195
Cl	1.59922985724172	-1.96124527417109	-1.46837214968299

17. References:

1. Austin, W. F., Zhang, Y., Danheiser, R. L., Reactions of (Trialkylsilyl)vinylketenes with Lithium Ynolates: A New Benzannulation Strategy *Org. Lett.*, **2005**, *7*, 3905–3908.
2. Danheiser R. L., Carini D. J., Fink D. M., Basak A.; Scope and stereochemical course of the (trimethylsilyl)cyclopentene annulation *Tetrahedron*, **1983**, *39*, 935–947.
3. Hong, P., Mise, T., Yamazaki, H., Synthesis of α,β-unsaturated ethyl ketones by cross-hydrocarbonylation of acetylenes and ethylene with carbon monoxide and hydrogen, *J. of Organomet. Chem.* **1987**, *334*, 129–140.
4. Powell, R. W., Buteler, M. P., Lenka, S., Crotti, M., Santangelo, S., Burg, M. J., Bruner, S., Brenna, E., Roitberga, A. E., Stewart, J. D., Investigating Saccharomyces cerevisiae alkene reductase OYE 3 by substrate profiling, X-ray crystallography and computational methods ***Catal. Sci. Technol.***, **2018**, ***8***, 5003–5016.
5. Peters B. B. C., Jongcharoenkamol J., Krajangsri S., Andersson P. G.; Highly Enantioselective Iridium-Catalyzed Hydrogenation of Conjugated Trisubstituted Enones, *Org. Lett*. **2021**, *23*, 242–246.
6. Zulfiqar, F., Malik, A., Facile Approach to Versatile Chiral Intermediates, *Naturforsch* **2001**, *56*, 1227–1234.
7. Steffen S., Schäfer A., Hiersemann M., Substituted *cis*-Hydrindan-4-ones by Sequential Cycloadditions, *Synthesis*, **2015**, *47*, 3489–3504.
8. Cadierno V., García-Garrido S. E., Gimeno J., Isomerization of Propargylic Alcohols into α,β-Unsaturated Carbonyl Compounds Catalyzed by the Sixteen-ElectronAllyl-Ruthenium(II) Complex [Ru(η^3-2-C_3H_4Me)(CO)(dppf)][SbF_6], *Adv. Synth. Catal.* **2006**, *348*, 101–110.
9. Zaidlewicz M., Transformation of Olefins into Homoallylic Alcohols, β,γ- and α,β-Unsaturated Ketones via Allylic Diethylboranes, *Synthesis* **1988**; *9*, 701–703; Fleming I., Perry D.A. The synthesis of αβ-unsaturated ketones from β-silylenones and β-silylynones, *Tetrahedron*, **1981**, *37*, 4027–4034.
10. Huijun, D., Yang, B., Pennington, L.D., Comer, E., Ford, M., Aversa, R., Weiss, M.M., Zhu, X., Beuming, T., KYMERA THERAPEUTICS - WO2023/220425, **2023**, A1.
11. Franov L. J., Hart J. D., Pullella G. A., Sumby C. J., George J. H., Bioinspired Total Synthesis of Erectones A and B, and the Revised Structure of Hyperelodione D, *Angew. Chem.Int. Ed.* **2022**, *61*, e202200420.
12. Dang, H.T., Nguyen, V. T., Nguyen, V. D., Arman, H.D., Larionov, O.V., *Org. Biomol. Chem.*, **2018**, *16*, 3605–3609.
13. Matsushita H., Negishi E., Selective carbon-carbon bond formation via transition-metal catalysis. Part 18. Palladium-catalyzed stereo- and regiospecific coupling of allylic derivatives with alkenyl- and arylmetals. A highly selective synthesis and 1,4-dienes, *J. Am. Chem. Soc.* **1981**, *103*, 2882–2884.
14. Muratore, A.; Duñach, E.; Clinet, J.-C.; Plessis, C. New Norbornyl Derivatives as Woody Fragrant Materials, *Chem. Biodiversity* **2008**, *5*, 1099–1114.
15. Hong S., Corey E. J., Enantioselective Syntheses of Georgyone, Arborone, and Structural Relatives. Relevance to the Molecular-Level Understanding of Olfaction, *J. Am. Chem. Soc.* **2006**, *128*, 1346–1352.
16. Watanabe S., Ujihara H., Yamamoto T., Hagiwara T., Production process of cyclohexenyl ketones, US6822121B2, **2001**.
17. Fernández Mateos A., Martín de la Nava E.M., Rubio González R., The Nazarov cyclization of β-carbonyl-β′-furyl-divinyl ketones and related compounds as induced by perchloric acid *Tetrahedron* **2001**, *57*, 1049–1057.

18. Ayyar, K. S.; Cookson, R. C.; Kagi, D. A.; Synthesis of 6-Damascone [trans-I -(2,6,6-Trimethylcyclohex-3-enyl) but-2-en-I -one] and p-Damascenone [trans-I -(2,6,6-Trimethylcyclohexa-l,3- dienyl)but-2-en-l –one, *Perkin Trans. 1* **1975**, *17*, 1727–1736.
19. Höfler, D., Kaupmees, K., Leito, I., List, B., A Chiral Sulfoxide-Based C–H Acid *Synlett*, **2021**, *32*, 45–47.
20. Lee, S., Kaib, P. S. J., List, B., *N*-Triflylphosphorimidoyl Trichloride: A Versatile Reagent for the Synthesis of Strong Chiral Brønsted Acids, *Synlett*, **2017**, *28*, 1478–1480.
21. Brunen, S., Mitschke, B., Leutzsch, M., List, B., Asymmetric Catalytic Friedel–Crafts Reactions of Unactivated Arenes, *J. Am. Chem. Soc.* **2023**, 145, 15708–15713.
22. Ghosh S., Erchinger J. E., Maji R., List B., Catalytic Asymmetric Spirocyclizing Diels–Alder Reactions of Enones: Stereoselective Total and Formal Syntheses of α-Chamigrene, β-Chamigrene, Laurencenone C, Colletoic Acid and Omphalic Acid, *J. Am. Chem. Soc.* **2022**, *144*, 6703–6708.
23. Neese, F. The ORCA program system. *WIREs Comput. Mol. Sci.* **2012**, *2*, 73–78.
24. Bannwarth, C., Ehlert, S., Grimme, S., GFN2-xTB-An Accurate and Broadly Parametrized Self-Consistent Tight-Binding Quantum Chemical Method with Multipole Electrostatics and Density-Dependent Dispersion Contributions. *J. Chem. Theory Comput.* **2019**, *15*, 1652-1671.
25. Pracht, P., Bohle, F., Grimme, S., Automated exploration of the low-energy chemical space with fast quantum chemical methods. *Phys. Chem. Chem. Phys.* **2020**, *22*, 7169–7192.
26. Becke, A. D., A new mixing of Hartree–Fock and local density-functional theories. *J. Chem. Phys.* **1993**, *98*, 1372–1377.
27. Caldeweyher, E. Bannwarth, C., Grimme, S., Extension of the D3 dispersion coefficient model, *J. Chem. Phys.* **2017**, *147*, 034112.
28. Weigend, F. Accurate Coulomb-fitting basis sets for H to Rn. *Phys. Chem. Chem. Phys.* **2006**, *8*, 1057–1065.
29. Weigend, F., Ahlrichs, R., Balanced basis sets of split valence, triple zeta valence and quadruple zeta valence quality for H to Rn: Design and assessment of accuracy, *Phys. Chem. Chem. Phys.* **2005**, *7*, 3297–3305.
30. Valeev, E. F. Libint: A library for the evaluation of molecular integrals of many-body operators over Gaussian functions, *http://libint.valeyev.net/*
31. Barone, V., Cossi, M., Quantum calculation of molecular energies and energy gradients in solution by a conductor solvent model, *J. Phys. Chem. A* **1998**, *102*, 1995–2001.
32. Lu, T.; Chen, F., Multiwfn: A multifunctional wavefunction analyzer. *J. Comput. Chem.* **2012**, *33*, 580–592. Xtb, Version 6.3; University Bonn: 2020; please refer to xtb@thch.uni-bonn.de. T. Lu, *molclus program*, *http://www.keinsci.com/research/molclus.html*.
33. Dale, J. A.; Dull, D. L.; Mosher, H. S., α-Methoxy-α-trifluoromethylphenylacetic acid, a versatile reagent for the determination of enantiomeric composition of alcohols and amines. *J. Org. Chem.* **1969**, *34*, 2543–2549.; Hoye, T. R.; Jeffrey, C. S.; Shao, F. Mosher Ester Analysis for the Determination of Absolute Configuration of Stereogenic (Chiral) Carbinol Carbons. *Nat. Protoc.* **2007**, *2*, 2451–2458.; Breitmaier, E. 200 and More NMR Experiments: A Practical Course; Wiley-VCH: Weinheim, **2004**.
34. Burns, A. S., Dooley, C., Carlson, P. R., Ziller, J. W., Rychnovsky, S. D., Relative and Absolute Structure Assignments of Alkenes Using Crystalline Osmate Derivatives for X-ray Analysis *Org. Lett.* **2019**, *21*, 10125–10129.
35. Maeda, S., Harabuchi, Y., Takagi, M., Taketsugu, T., Morokuma, K., Artificial Force Induced Reaction (AFIR) Method for Exploring Quantum Chemical Potential Energy Surfaces *Chem. Rec.* **2016**, *16*, 2232–2248.

36. Maeda, S., Ohno, K., Morokuma, K., Systematic exploration of the mechanism of chemical reactions: the global reaction route mapping (GRRM) strategy using the ADDF and AFIR methods *Phys. Chem. Chem. Phys.* **2013**, *15*, 3683–3701.
37. Grimme, S., Hansen, A., Ehlert, S., Mewes, J.-M., r^2SCAN-3c: A “Swiss army knife” composite electronic-structure method *J. Chem. Phys.* **2021** *154*, 064103.
38. Neese, F., Software update: The ORCA program system—Version 5.0 *Comput Mol Sci.* **2022**, *12*, e1606.
39. Mardirossian, N., Head-Gordon, M., ωB97M-V: A combinatorially optimized, range-separated hybrid, meta-GGA density functional with VV10 nonlocal correlation *J. Chem. Phys.* **2016**, *144*, 214110.
40. Lu, T., Chen, Q., Independent gradient model based on Hirshfeld partition:A new method for visual study of interactions inchemical systems *J Comput Chem.* **2022**;*43*:539–555.
41. Frisch, M. J.; Trucks, G. W.; Schlegel, H. B.; Scuseria, G. E.; Robb, M. A.; Cheeseman, J. R.; Scalmani, G.; Barone, V.; Petersson, G. A.; Nakatsuji, H.; Li, X.; Caricato, M.; Marenich, A. V.; Bloino, J.; Janesko, B. G.; Gomperts, R.; Mennucci, B.; Hratchian, H. P.; Ortiz, J. V.; Izmaylov, A. F.; Sonnenberg, J. L.; Williams-Young, D.; Ding, F.; Lipparini, F.; Egidi, F.; Goings, J.; Peng, B.; Petrone, A.; Henderson, T.; Ranasinghe, D.; Zakrzewski, V. G.; Gao, J.; Rega, N.; Zheng, G.; Liang, W.; Hada, M.; Ehara, M.; Toyota, K.; Fukuda, R.; Hasegawa, J.; Ishida, M.; Nakajima, T.; Honda, Y.; Kitao, O.; Nakai, H.; Vreven, T.; Throssell, K.; Montgomery, J. A., Jr.; Peralta, J. E.; Ogliaro, F.; Bearpark, M. J.; Heyd, J. J.; Brothers, E. N.; Kudin, K. N.; Staroverov, V. N.; Keith, T. A.; Kobayashi, R.; Normand, J.; Raghavachari, K.; Rendell, A. P.; Burant, J. C.; Iyengar, S. S.; Tomasi, J.; Cossi, M.; Millam, J. M.; Klene, M.; Adamo, C.; Cammi, R.; Ochterski, J. W.; Martin, R. L.; Morokuma, K.; Farkas, O.; Foresman, J. B.; Fox, D. J. Gaussian 16, Revision A.03; Gaussian, Inc.: Wallingford, CT, **2016**.
42. Meng, E. C., Goddard, T. D., Pettersen, E. F., Couch, G. S., Pearson, Z. J., Morris, J. H., Ferrin, T. E. UCSF ChimeraX: Tools for structure building and analysis *Protein Science* **2023**; *32*, e4792.
43. Peng, Q., Duarte, F., Paton, R. S. Computing organic stereoselectivity – from concepts to quantitative calculations and predictions *Chem. Soc. Rev.*, **2016**, *45*, 6093–6107.
44. Lu, T., A comprehensive electron wavefunction analysis toolbox for chemists, Multiwfn *J. Chem. Phys.* **2024**, *161*, 082503.
45. Anderson, T.L.; Kwan, E.E. PyQuiver 2020, www.github.com/ekwan/PyQuiver

18. NMR spectra

18.1. Enones:

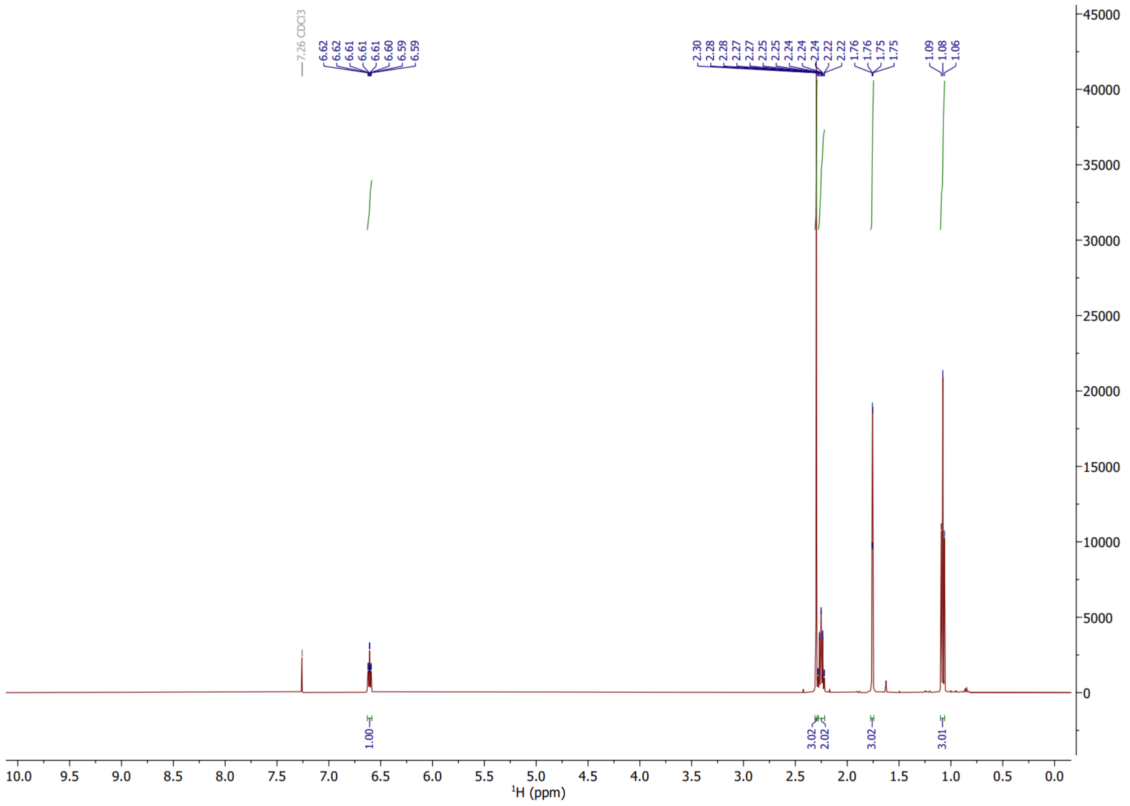

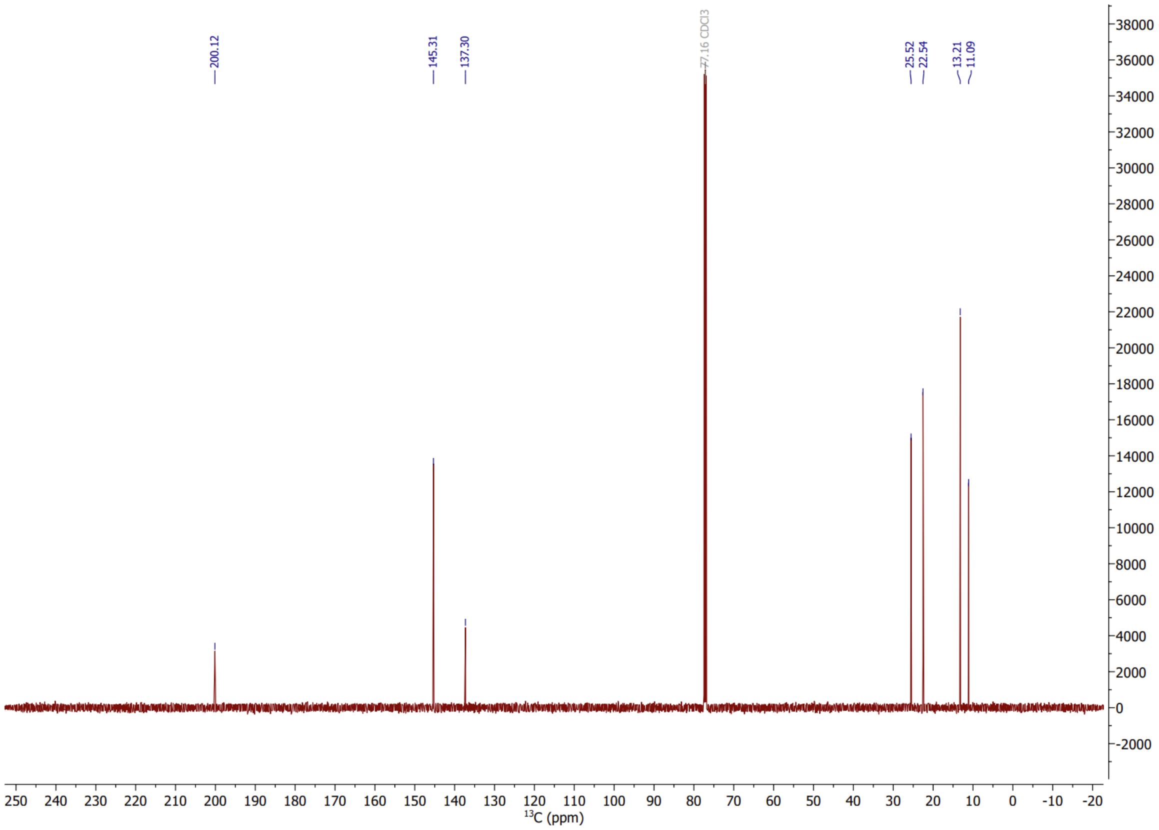

Figure S26. ^{1}H NMR and ^{13}C NMR spectra of compound **2b**.

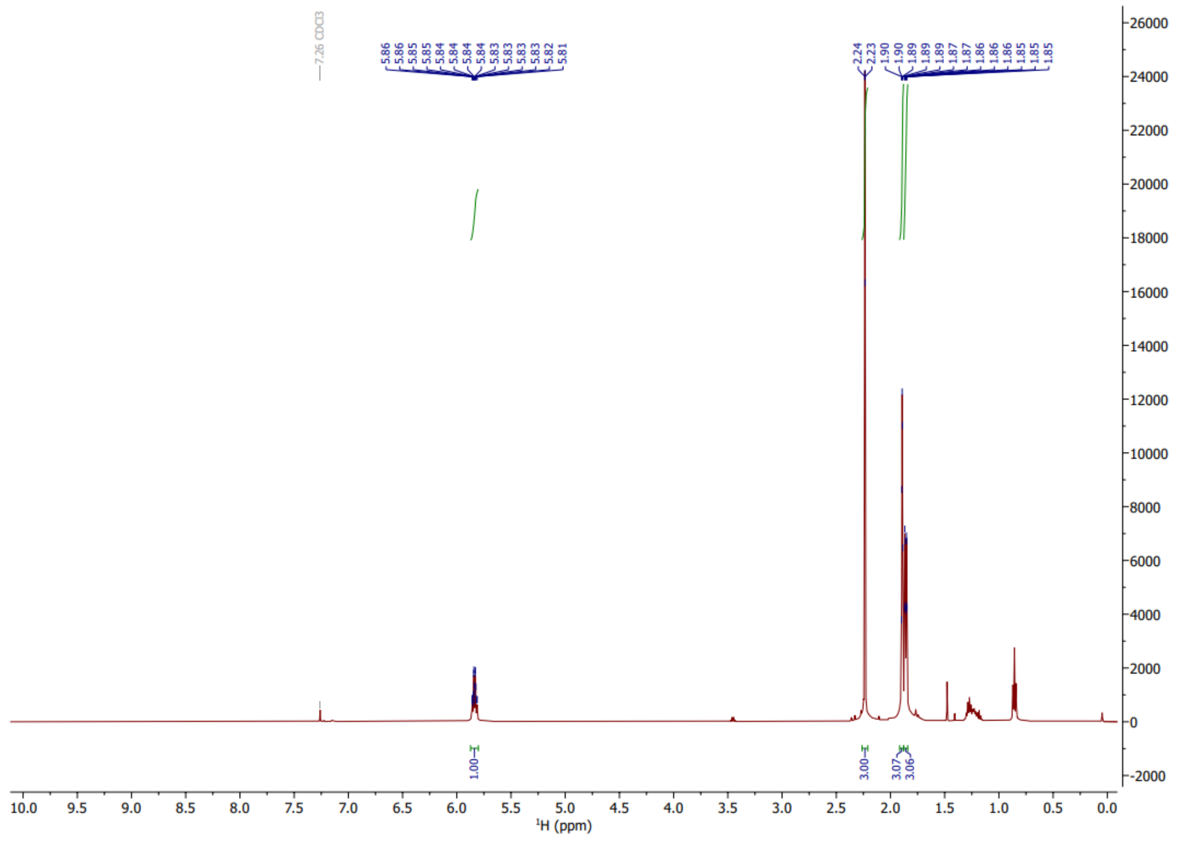

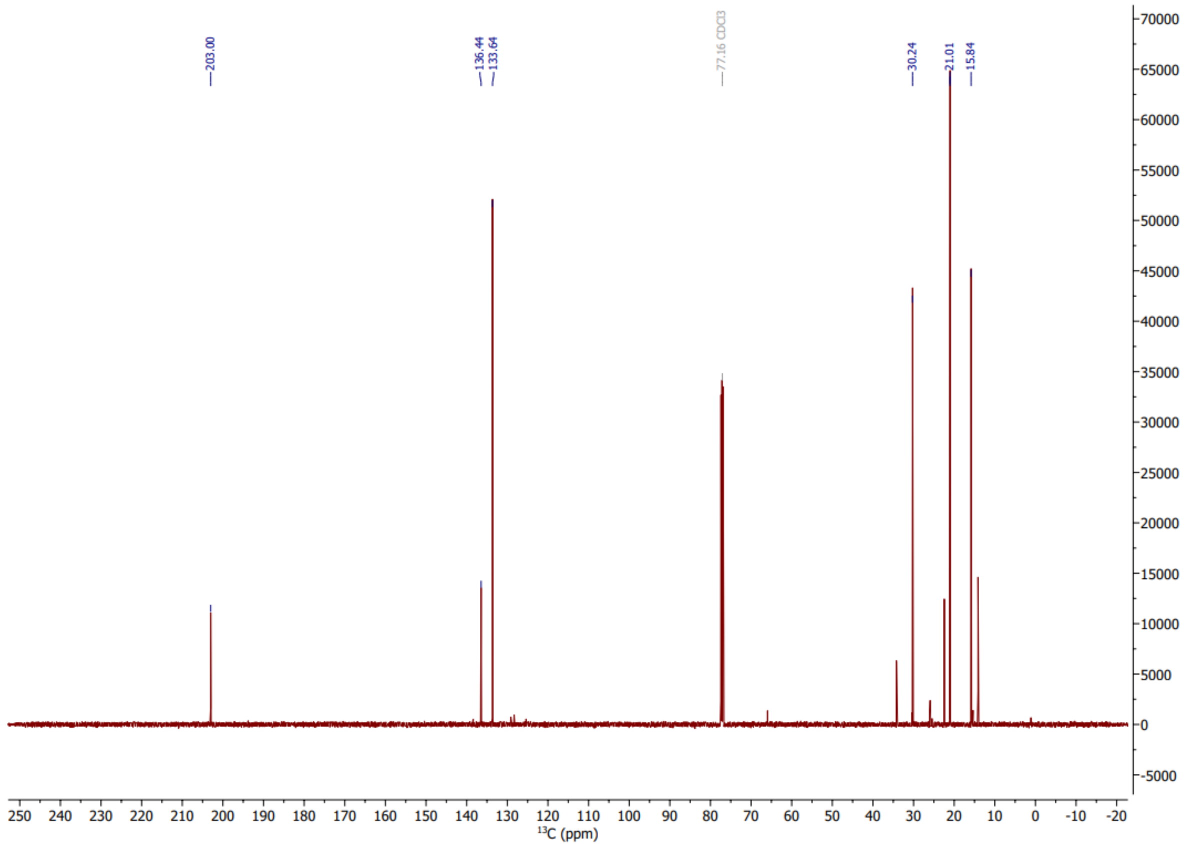

Figure S27. ^{1}H NMR and ^{13}C NMR spectra of compound **(*Z*)-2a**.

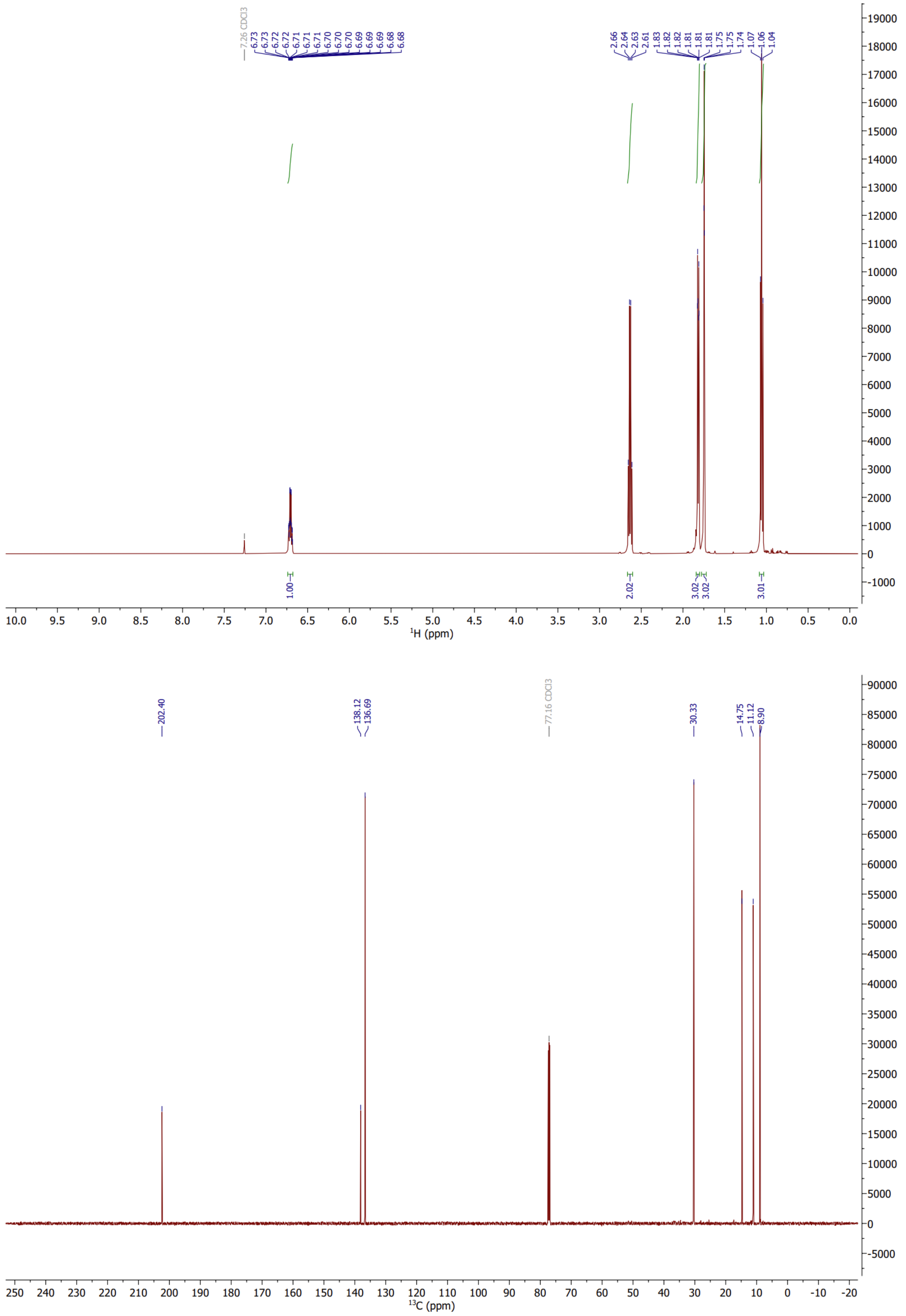

Figure S28. ^{1}H NMR and ^{13}C NMR spectra of compound **2q'**.

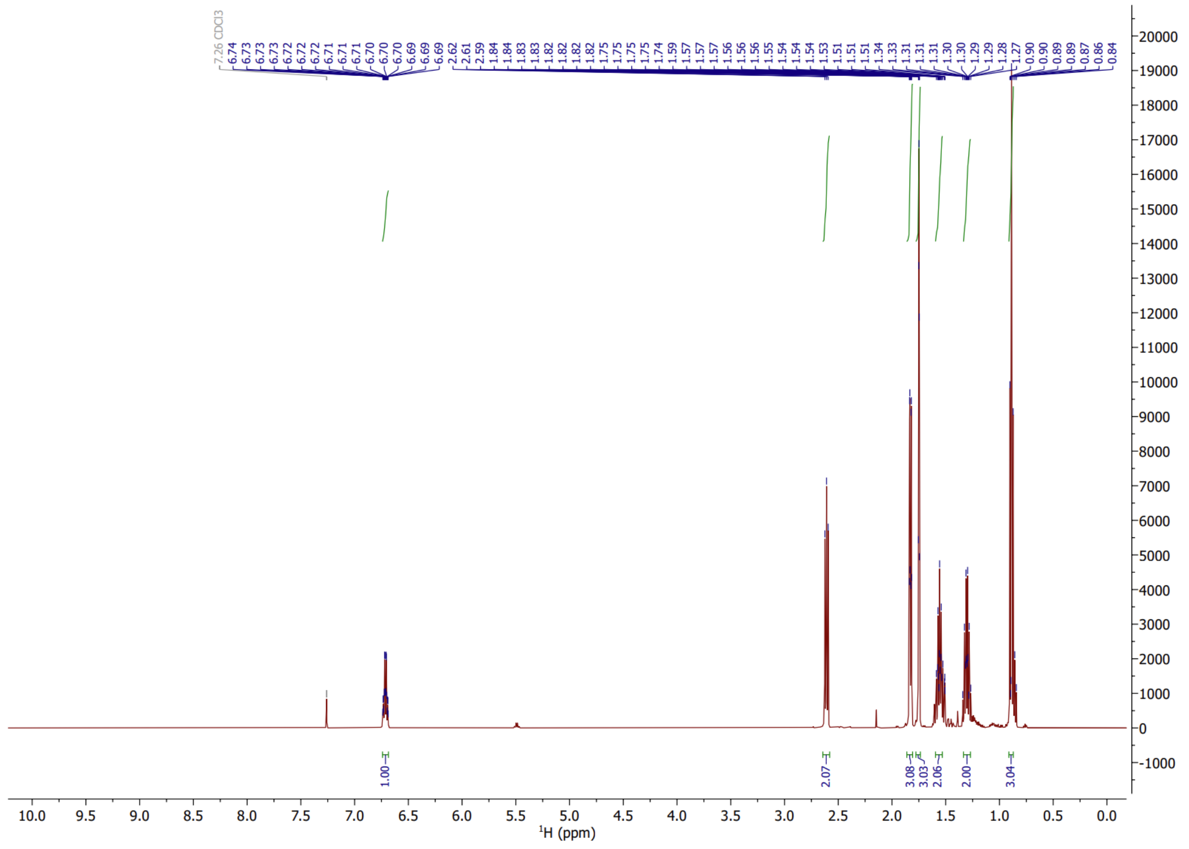

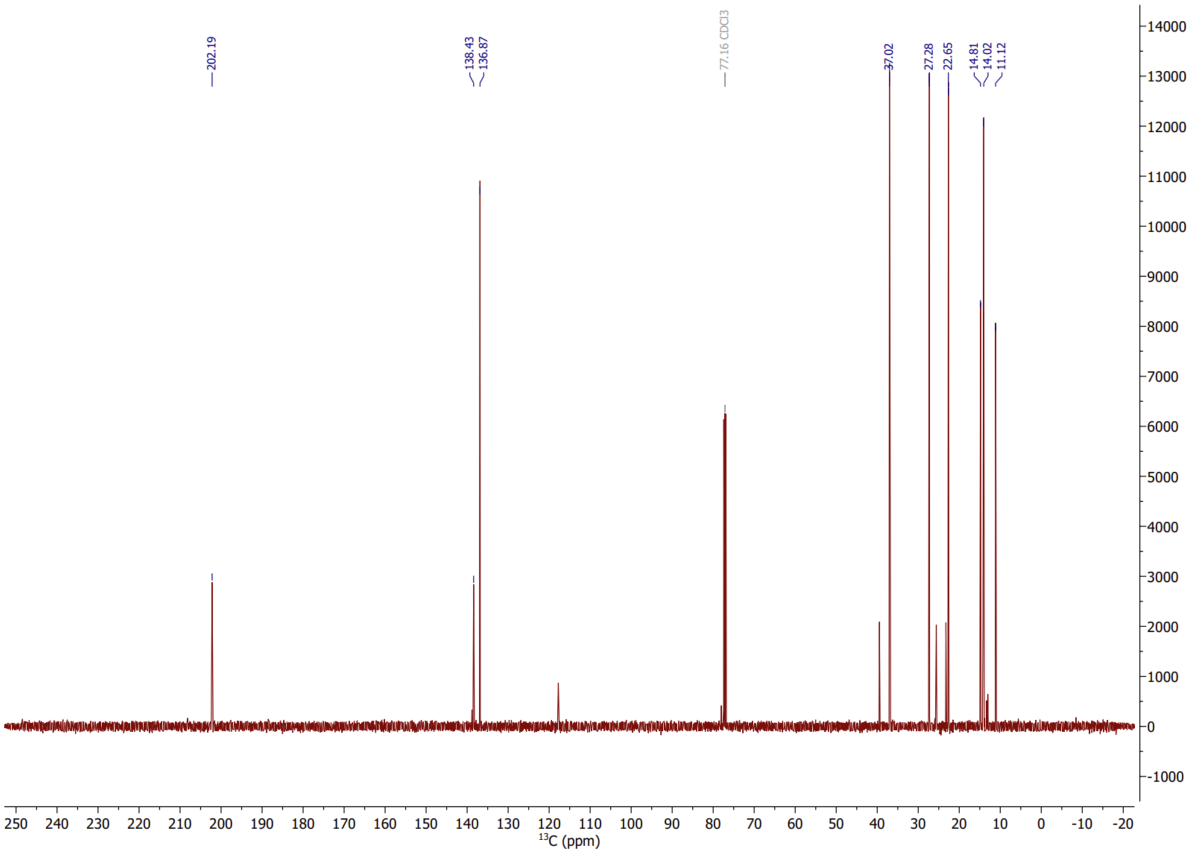

Figure S29. ^{1}H NMR and ^{13}C NMR spectra of compound **2q’’**.

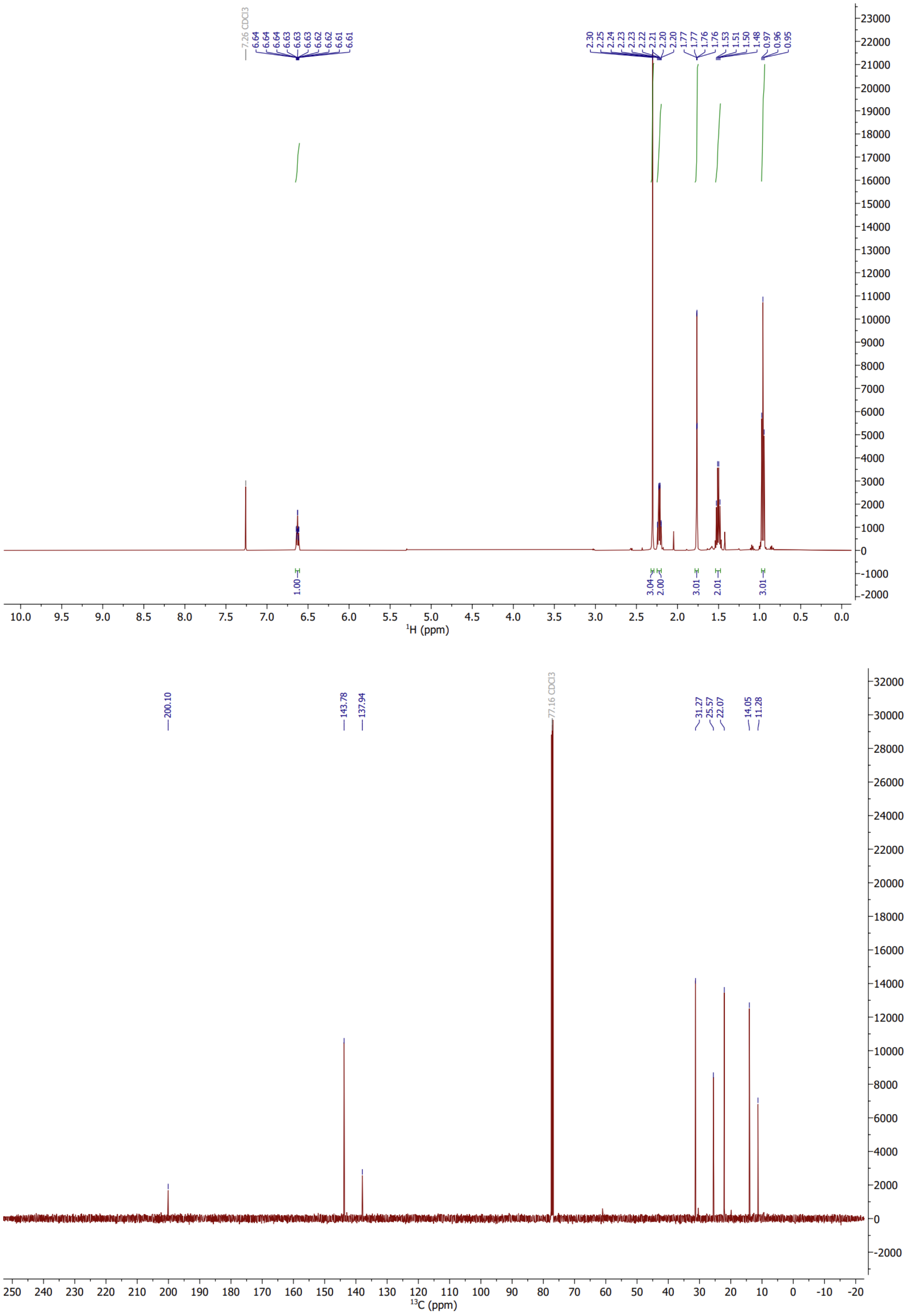

Figure S30. ^{1}H NMR and ^{13}C NMR spectra of compound **2c**.

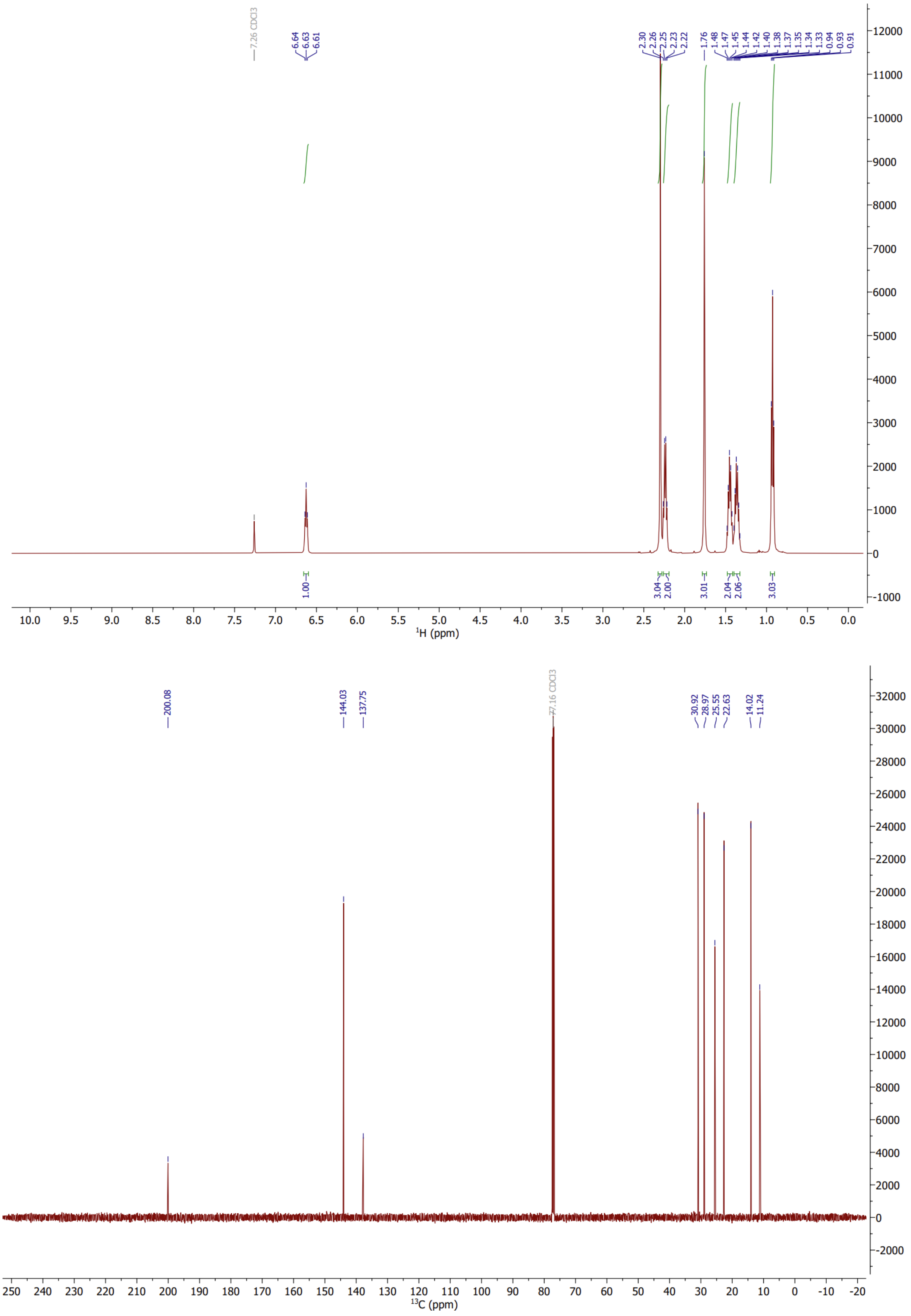

Figure S31. ^{1}H NMR and ^{13}C NMR spectra of compound **2d**.

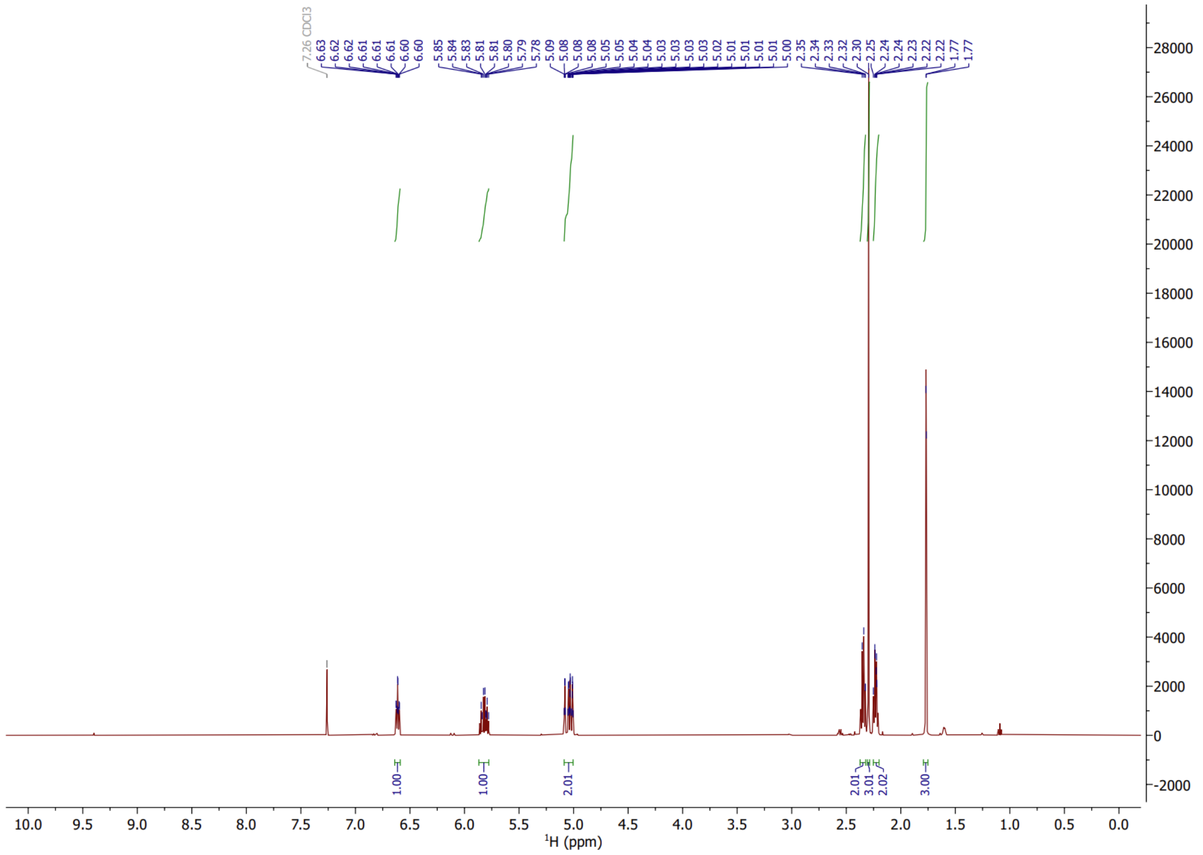

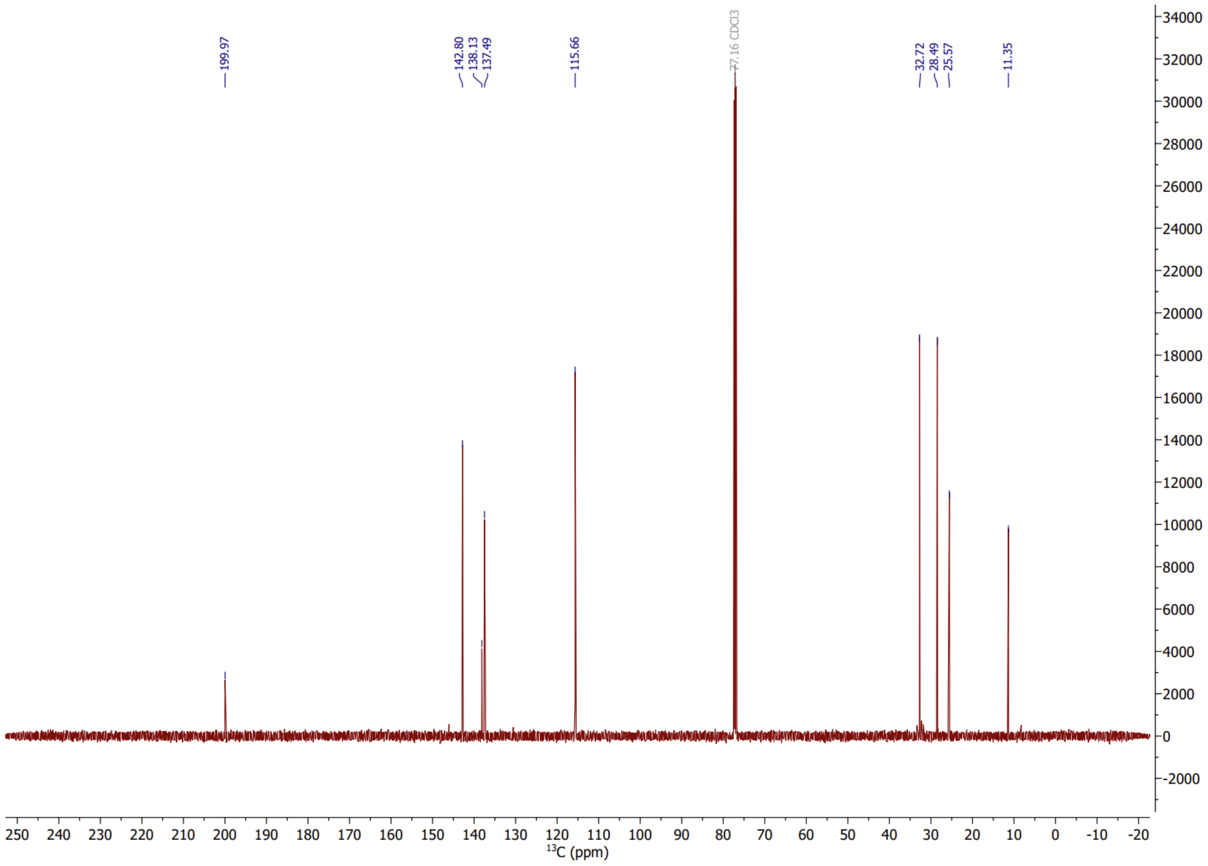

Figure S32. ^{1}H NMR and ^{13}C NMR spectra of compound **2e**.

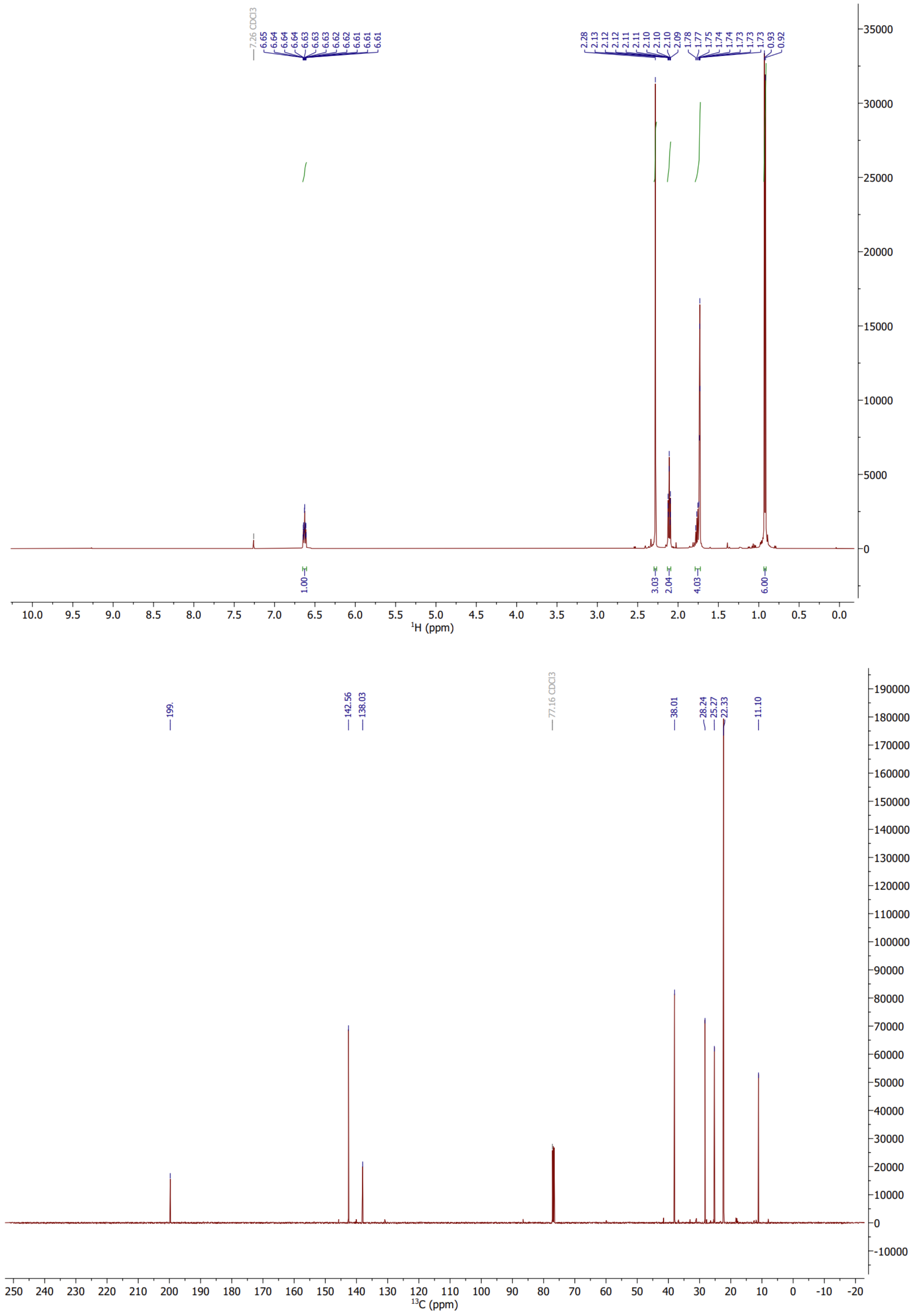

Figure S33. ^{1}H NMR and ^{13}C NMR spectra of compound **2f**.

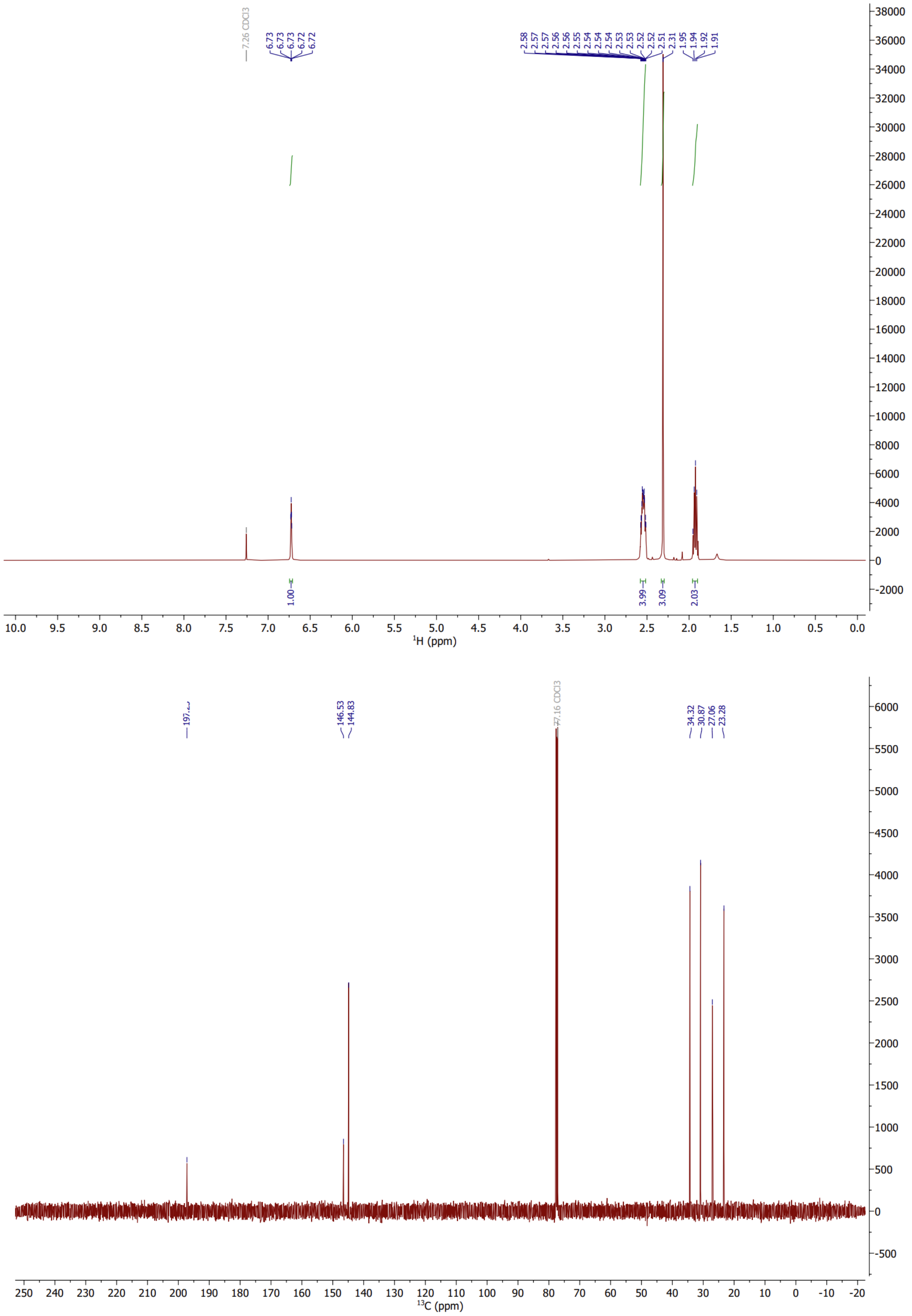

Figure S34. ^{1}H NMR and ^{13}C NMR spectra of compound **2g**.

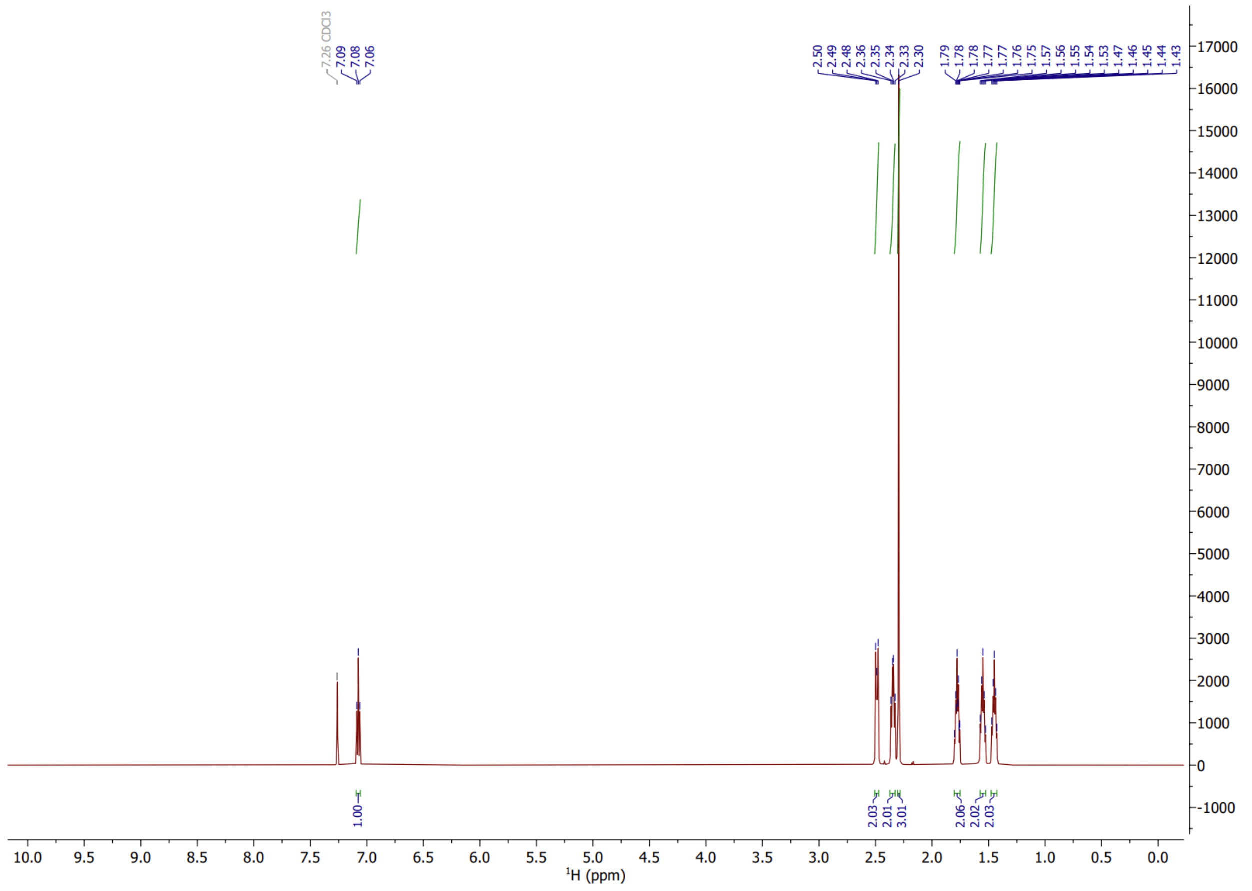

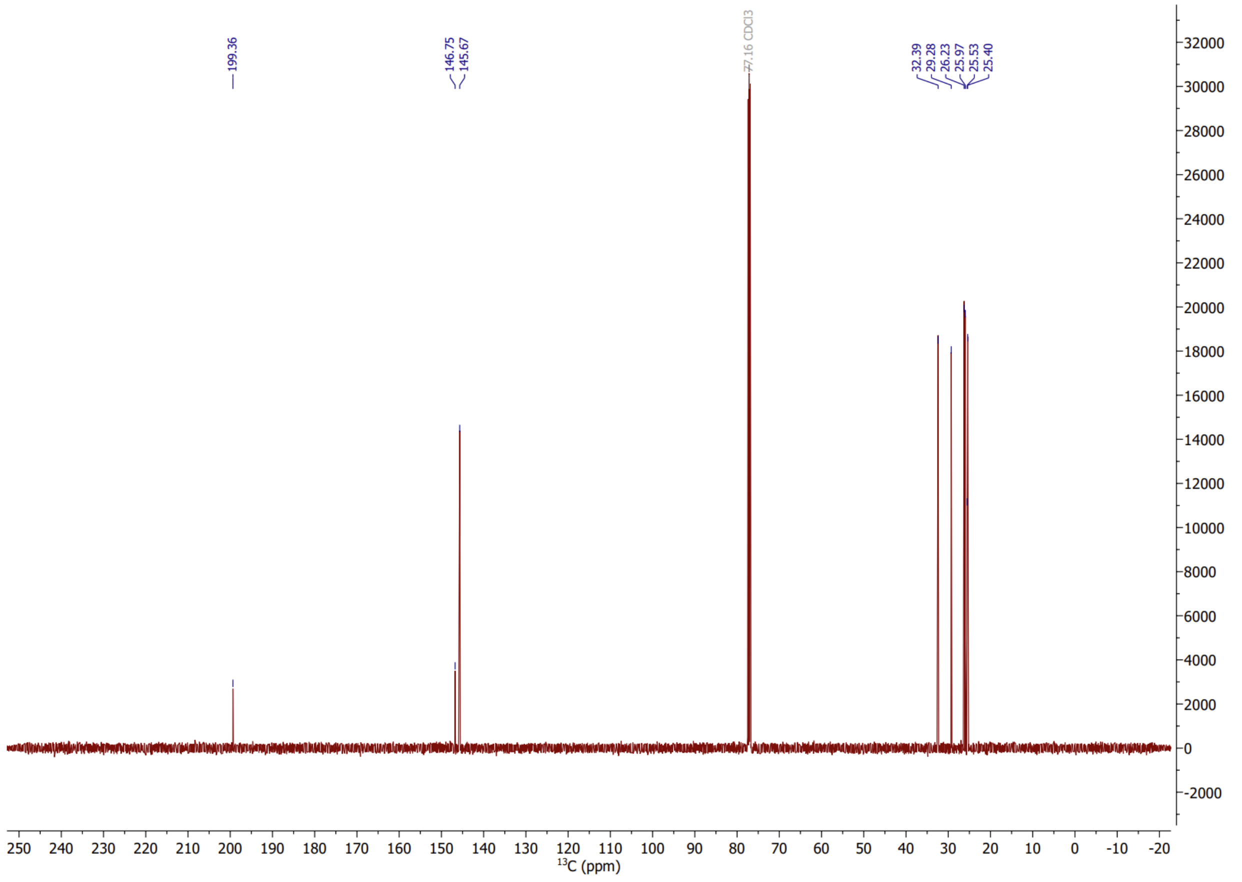

Figure S35. ^{1}H NMR and ^{13}C NMR spectra of compound **2i**.

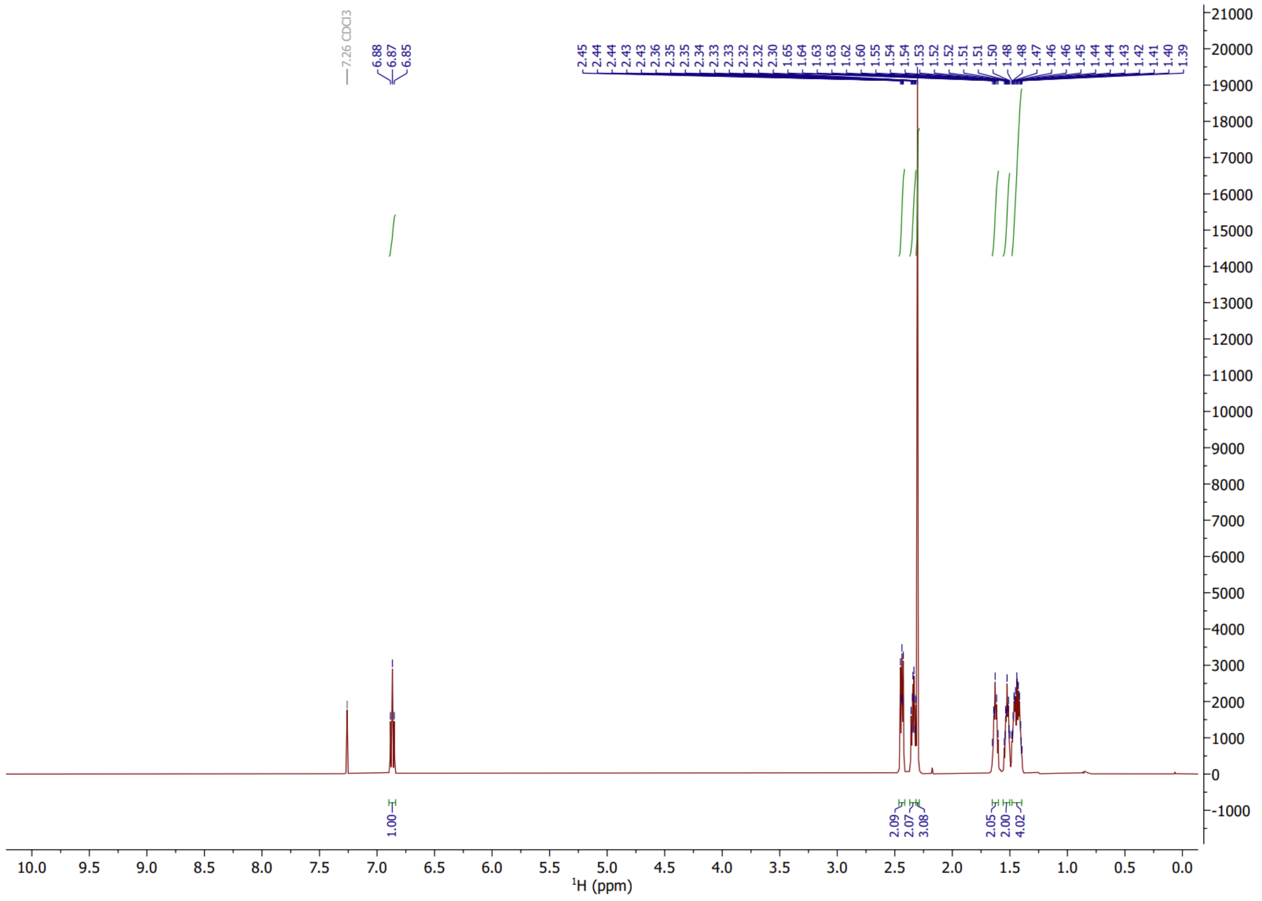

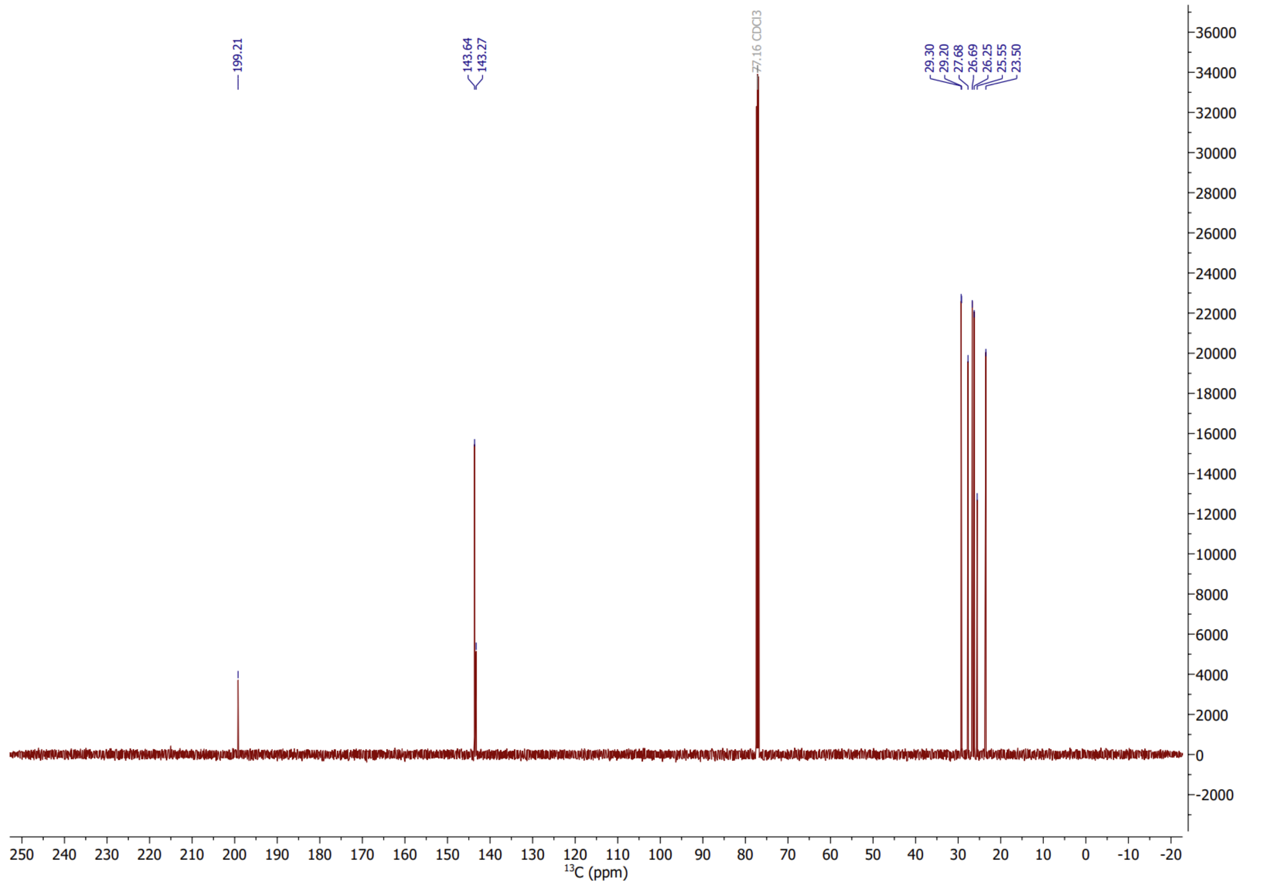

Figure S36. ^{1}H NMR and ^{13}C NMR spectra of compound **2j**.

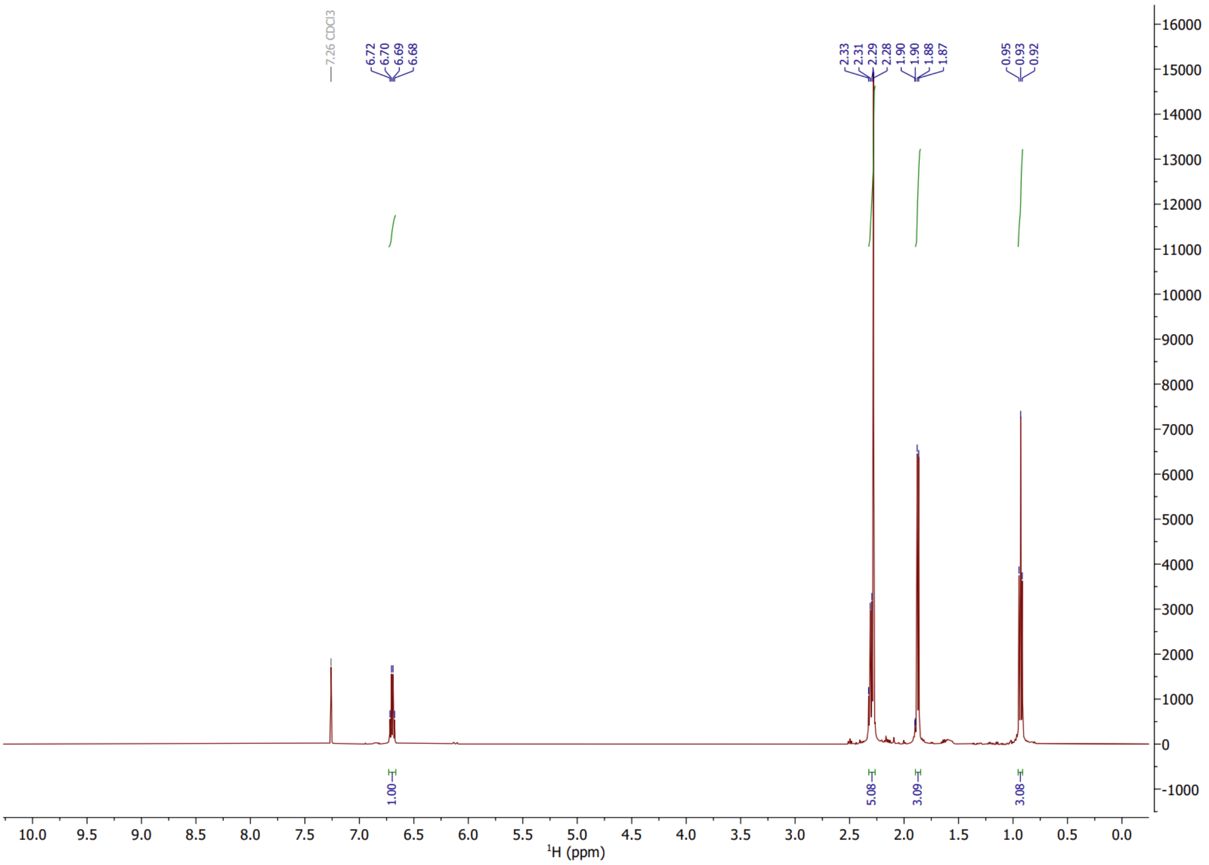

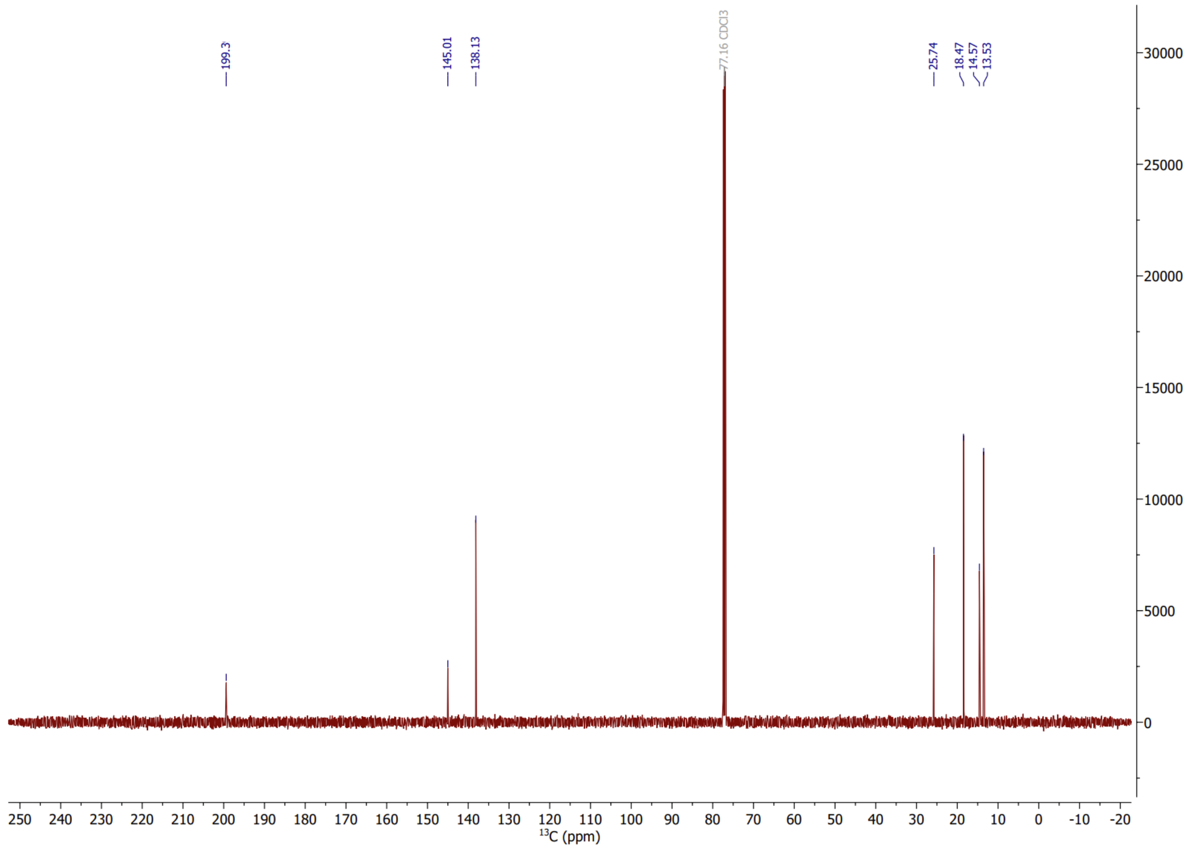

Figure S37. ^{1}H NMR and ^{13}C NMR spectra of compound **2l**.

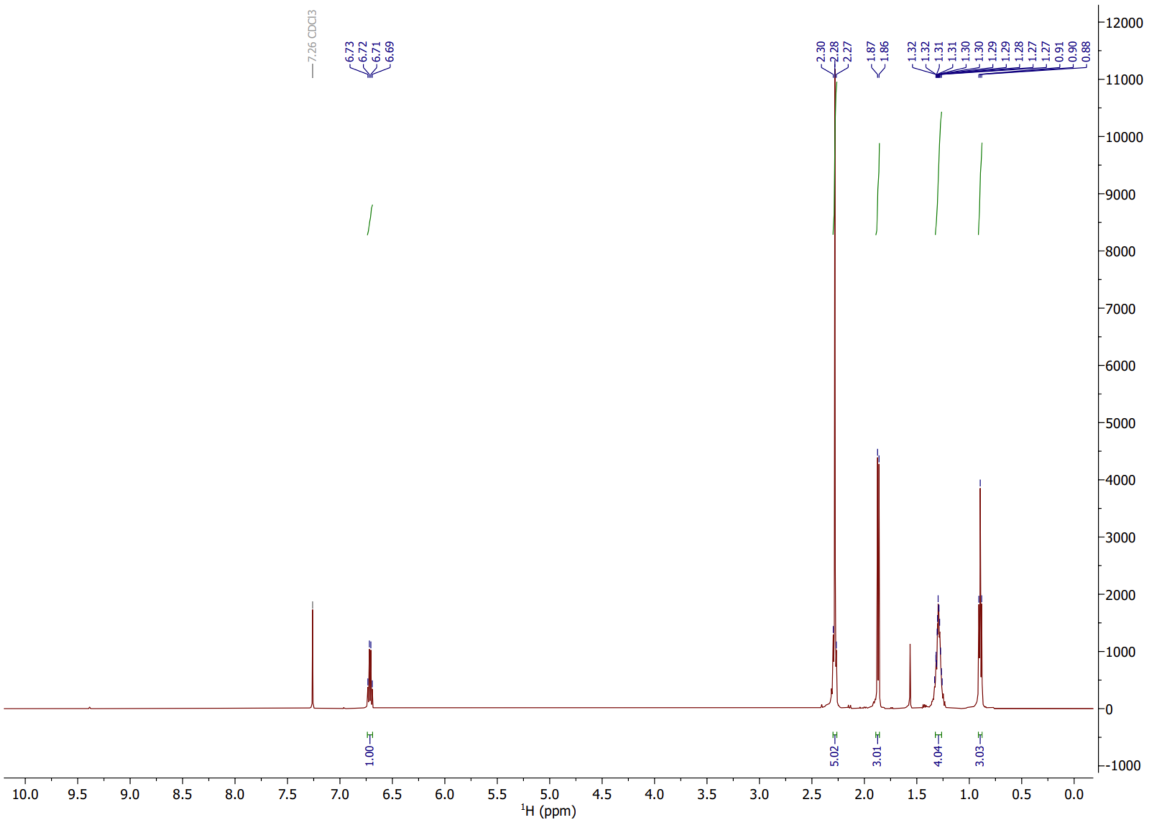

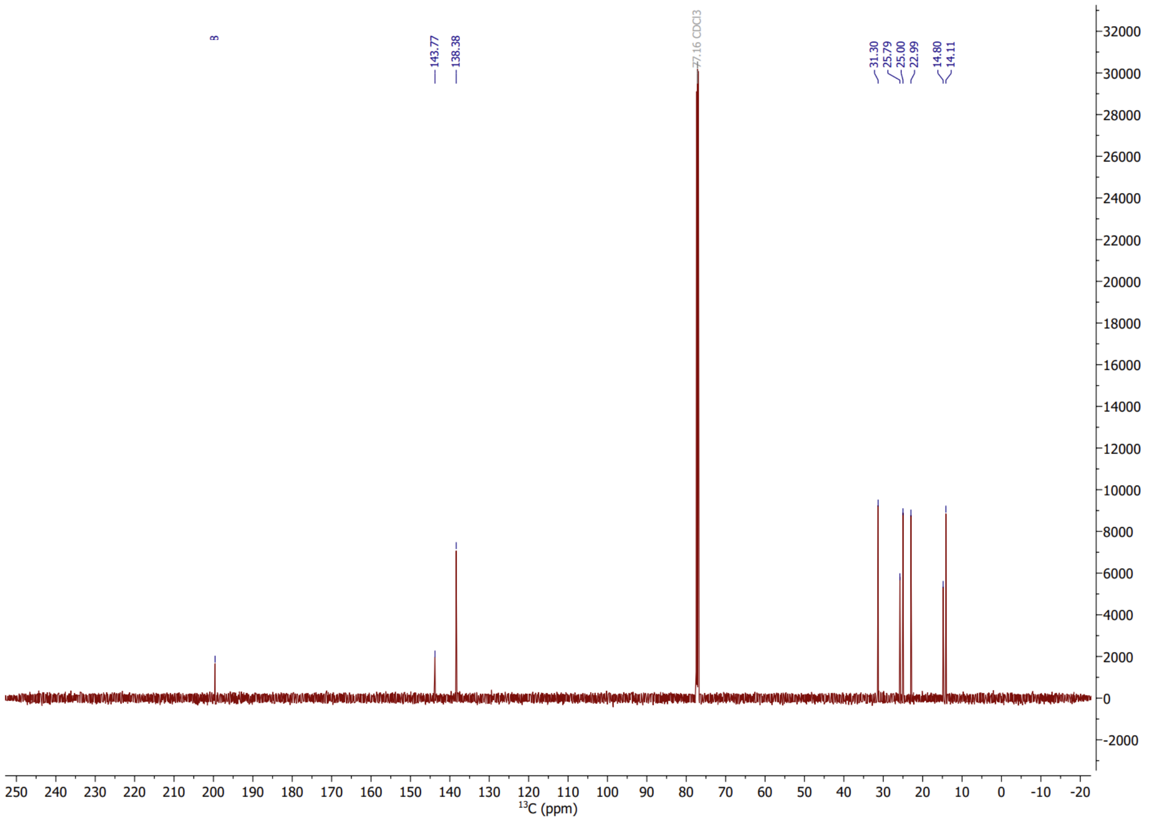

Figure S38. ^{1}H NMR and ^{13}C NMR spectra of compound **2m**.

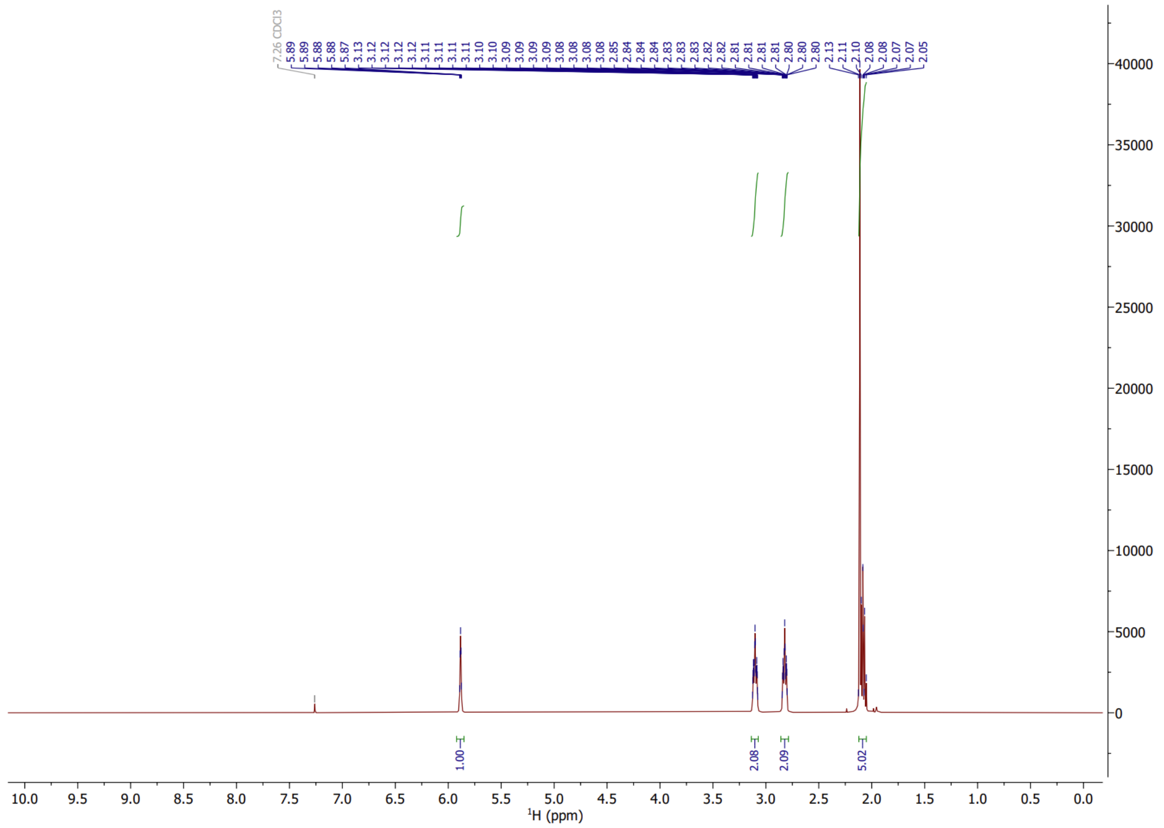

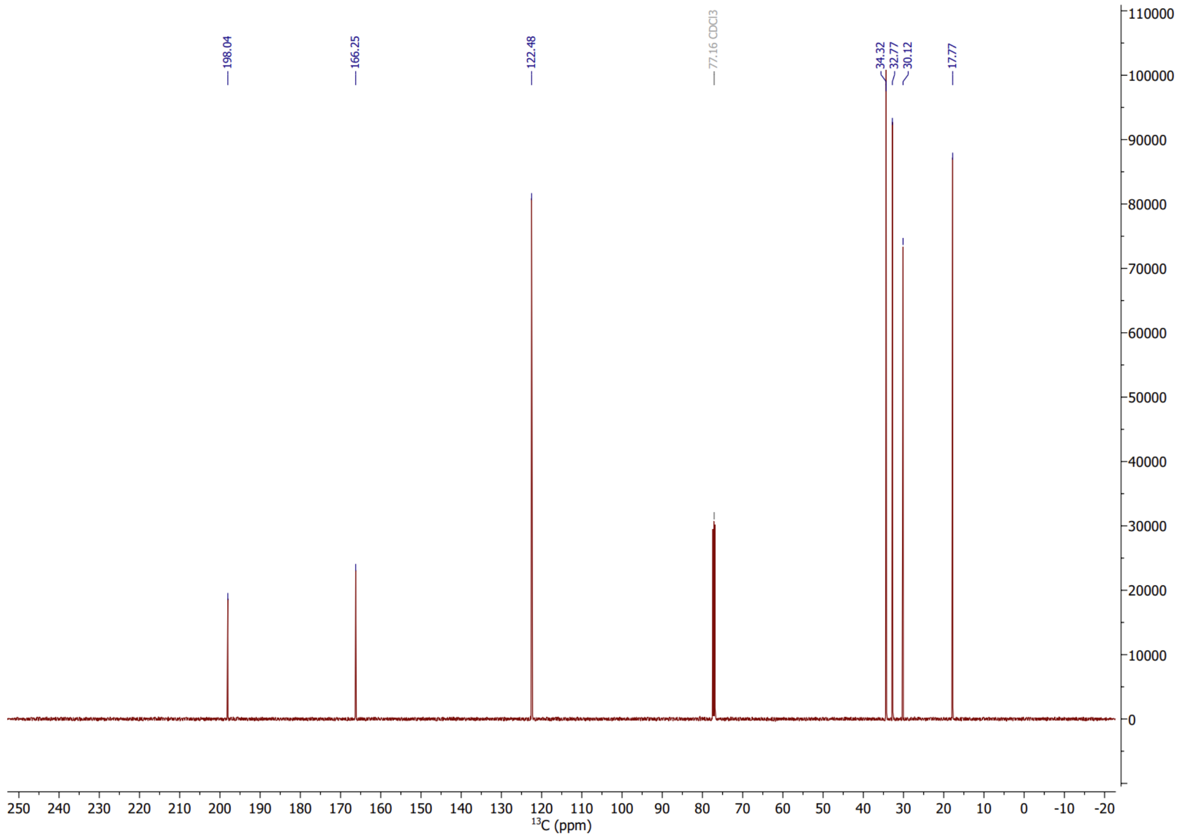

Figure S39. ^{1}H NMR and ^{13}C NMR spectra of compound **2o**.

18.2. (*E*,*E*)-α-farnesene

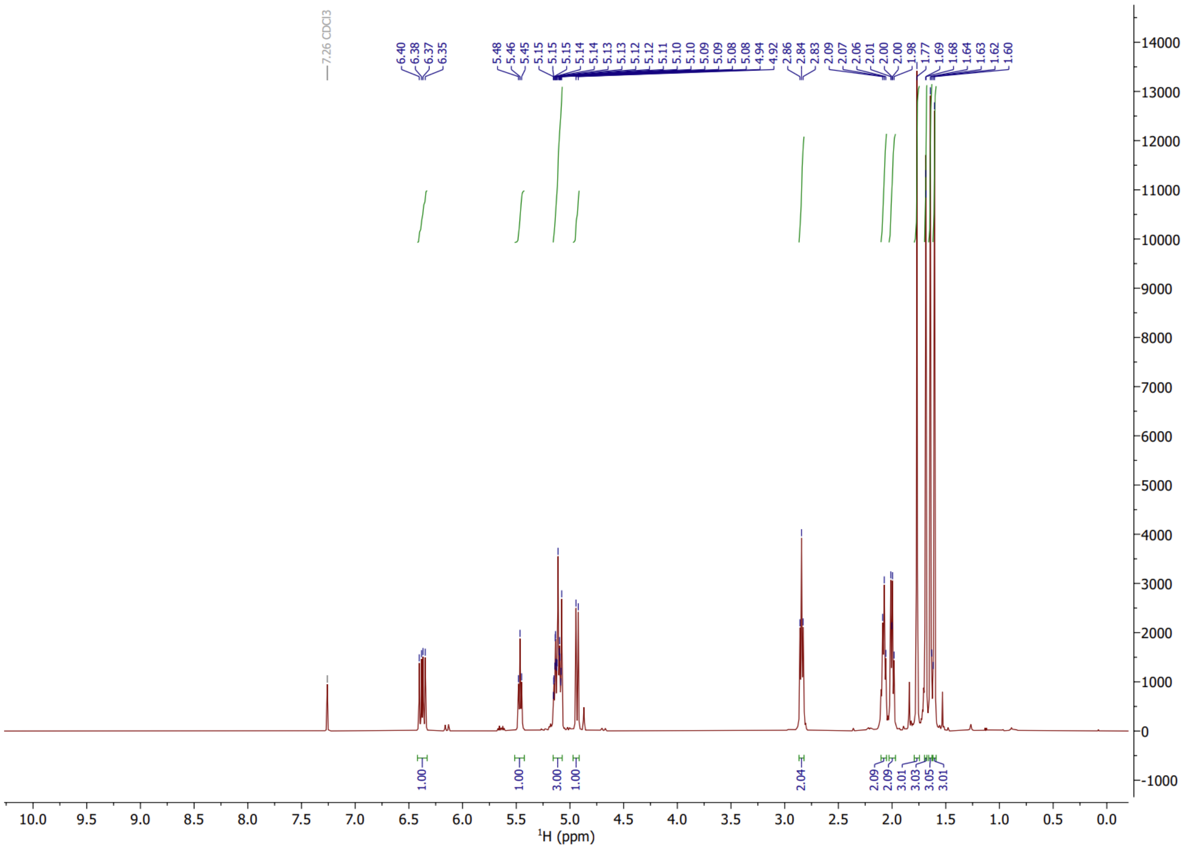

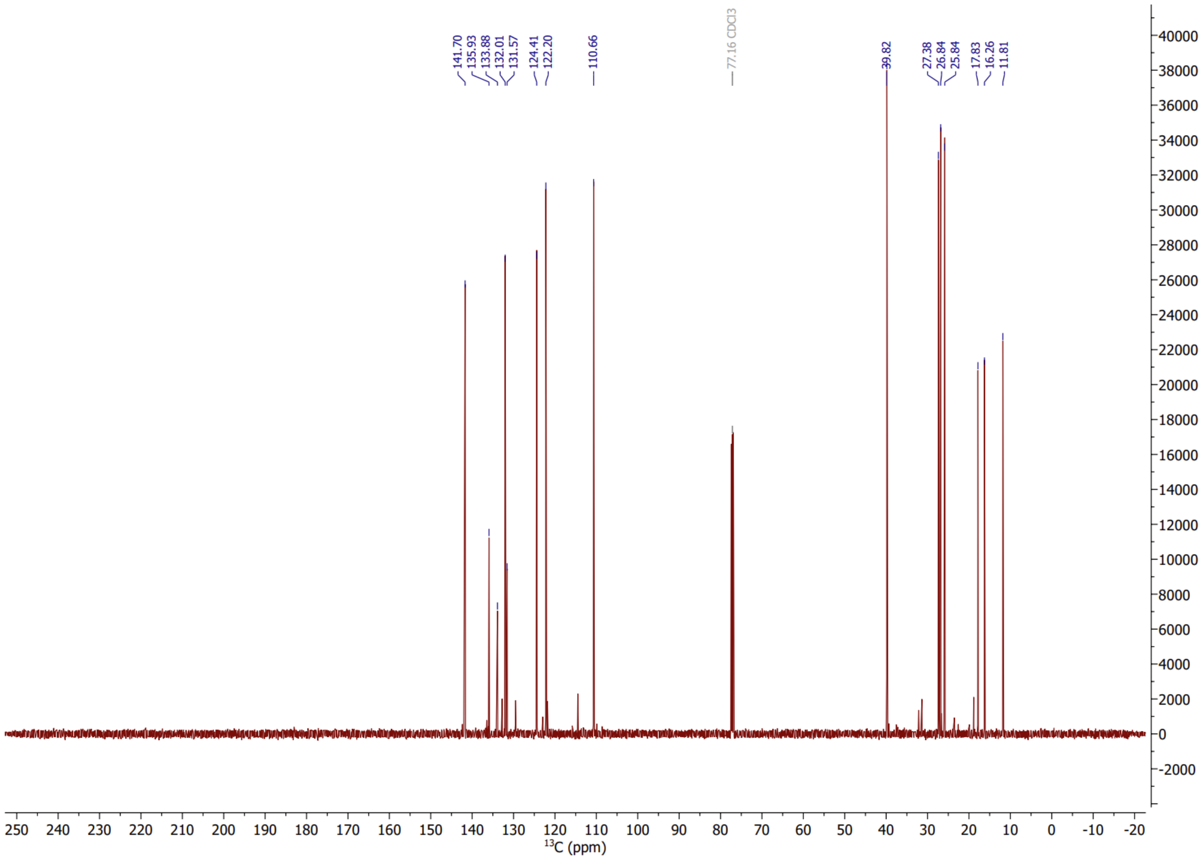

Figure S40. ^{1}H NMR and ^{13}C NMR spectra of compound **3f**

18.3. Diels-Alder Cycloaddition Products (4a-w)

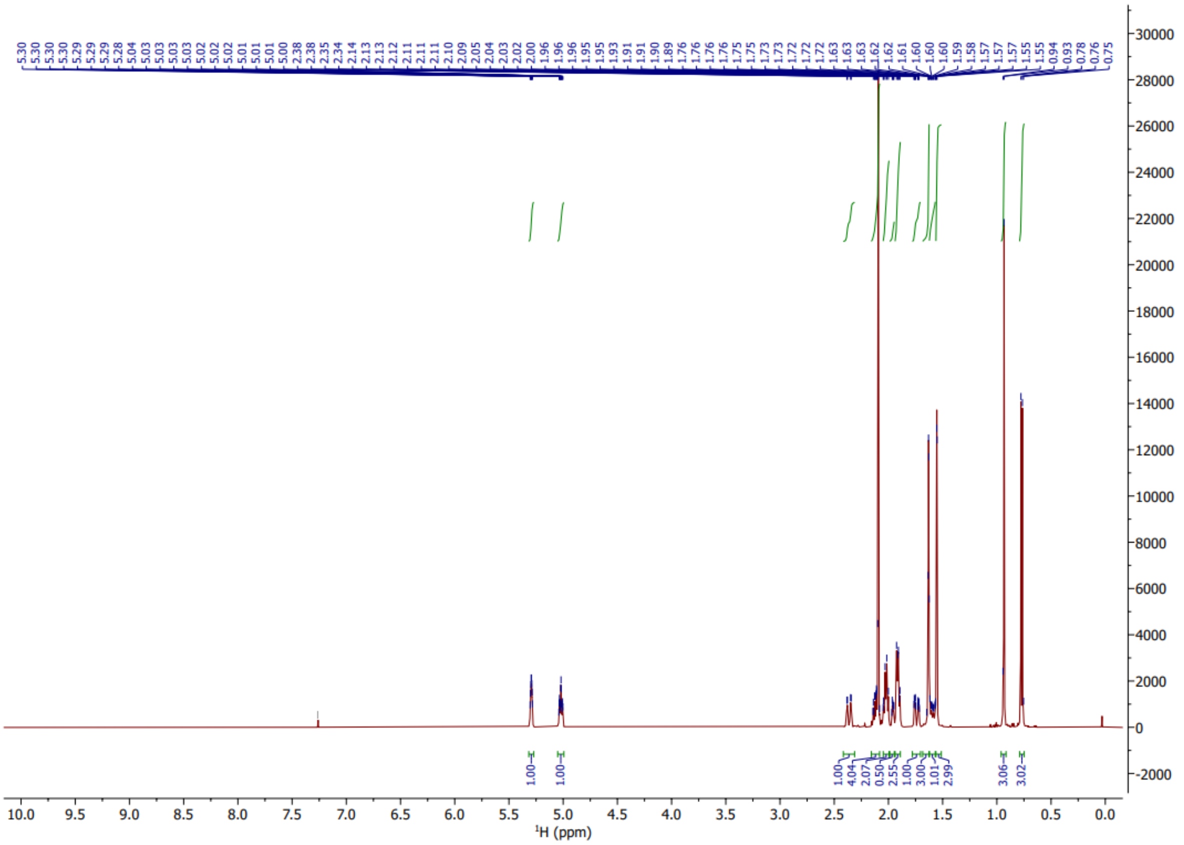

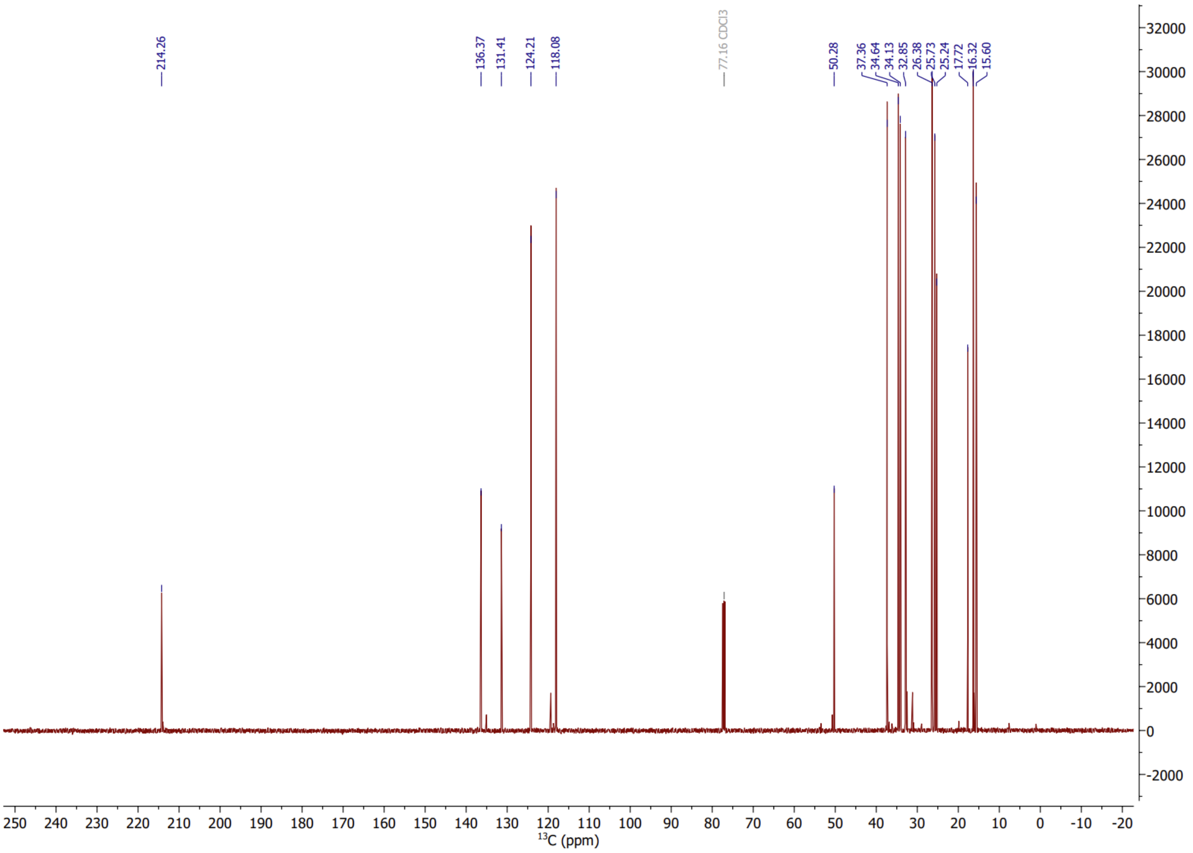

Figure S41. ^{1}H NMR and ^{13}C NMR spectra of compound **4a**.

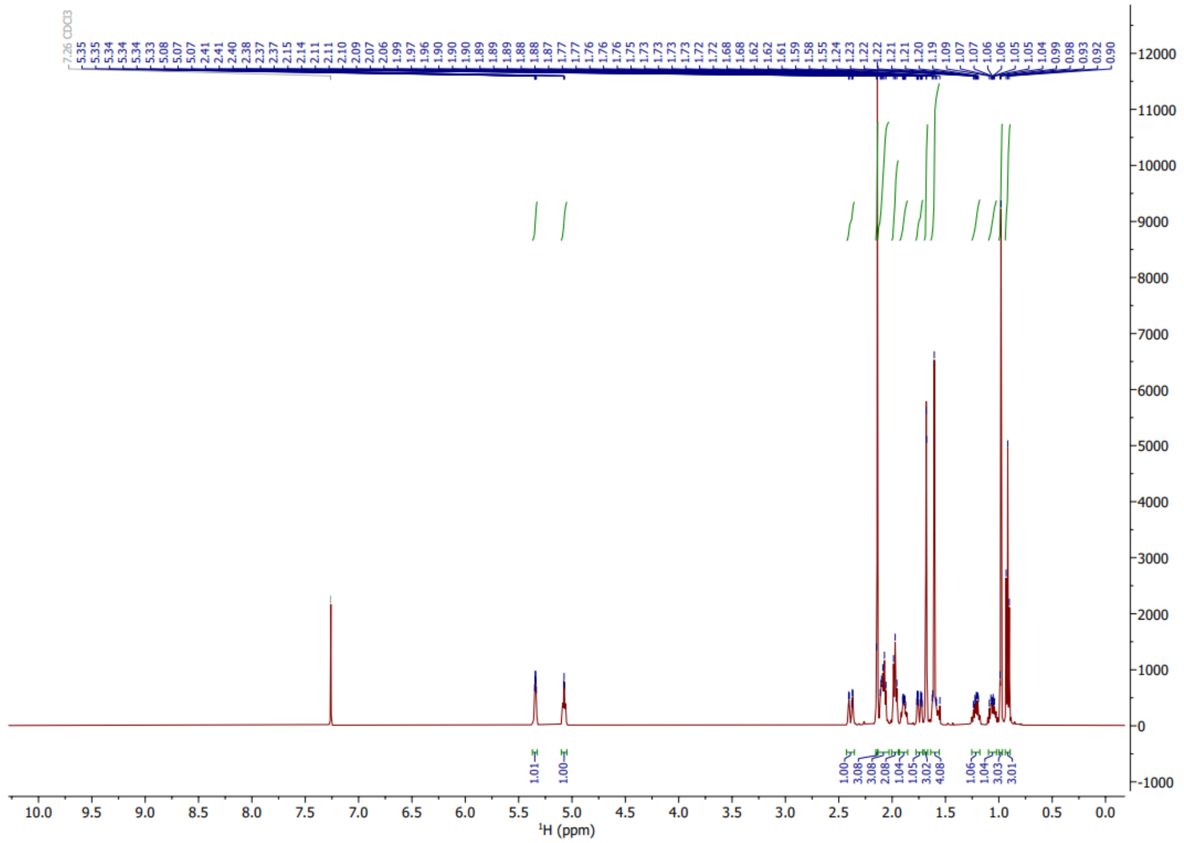

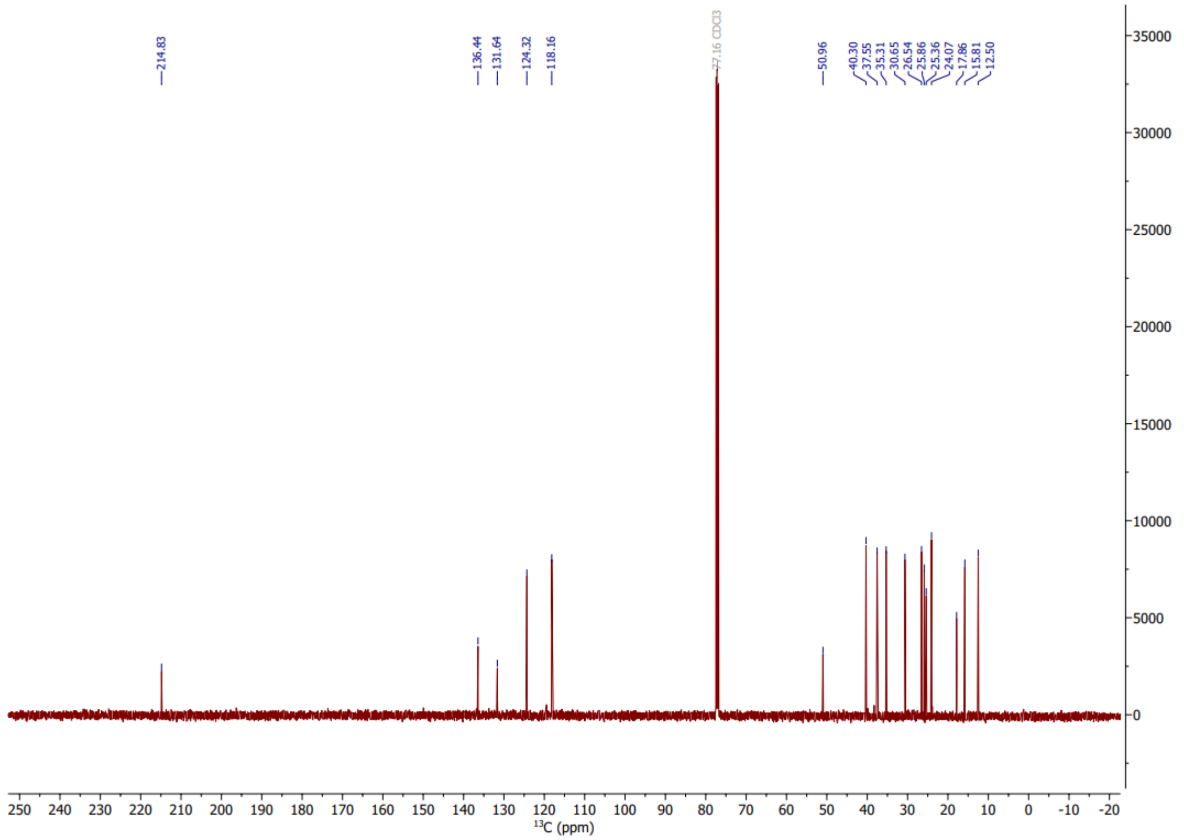

Figure S42. ^{1}H NMR and ^{13}C NMR spectra of compound **4b**.

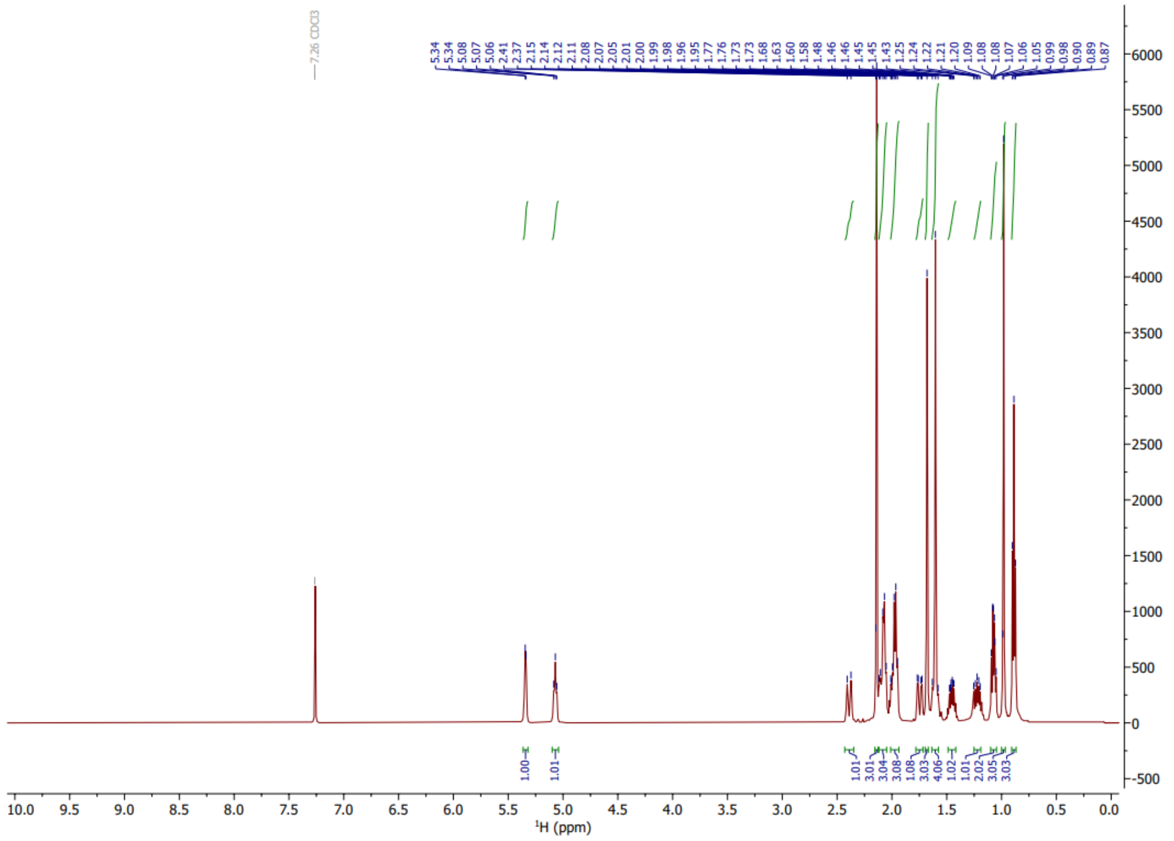

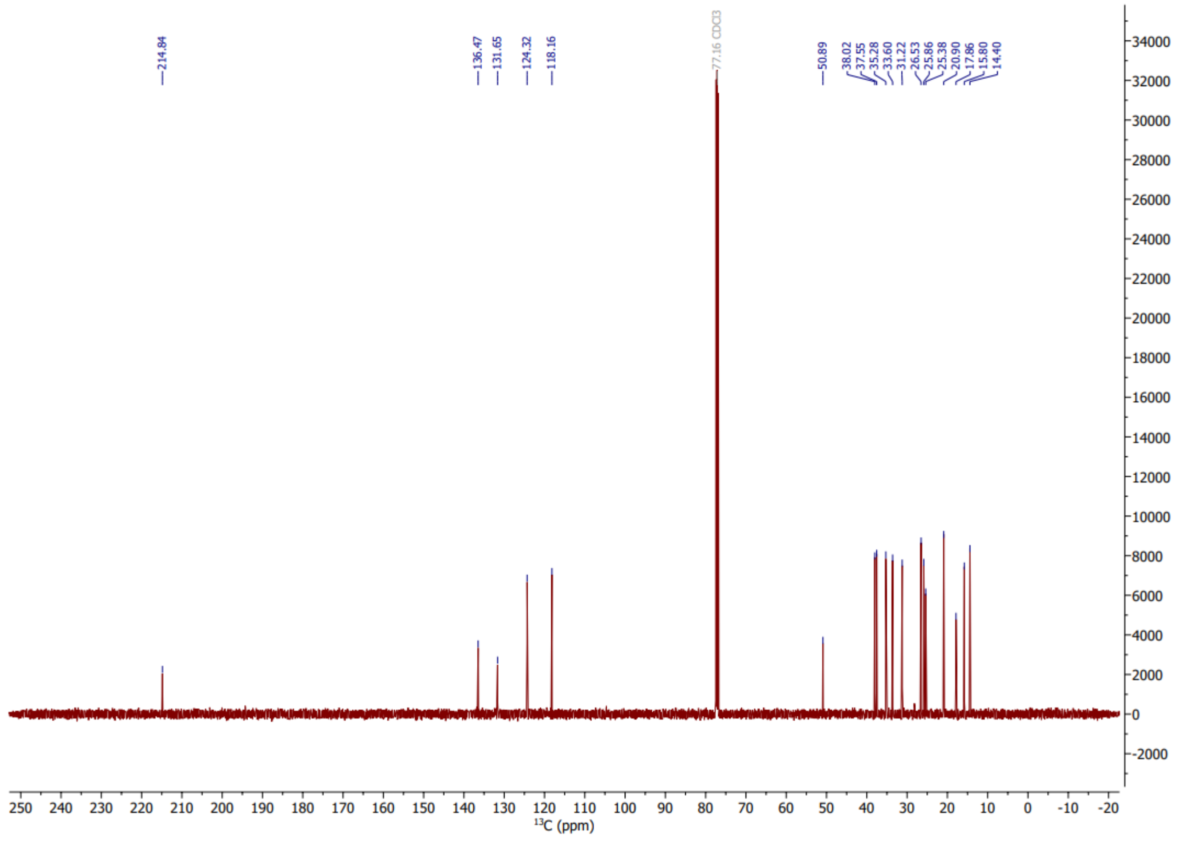

Figure S43. ^{1}H NMR and ^{13}C NMR spectra of compound **4c**.

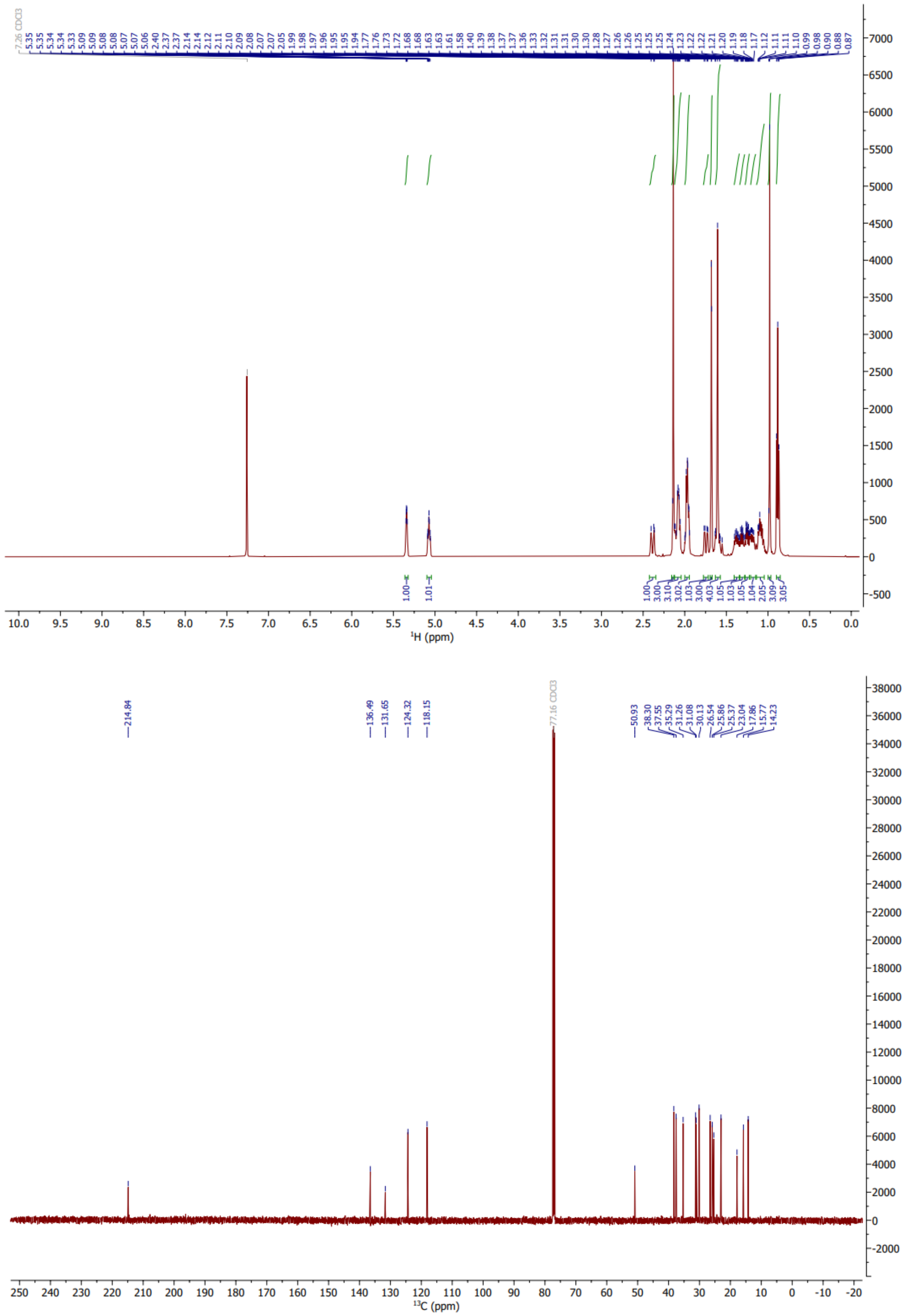

Figure S44. ^{1}H NMR and ^{13}C NMR spectra of compound **4d**.

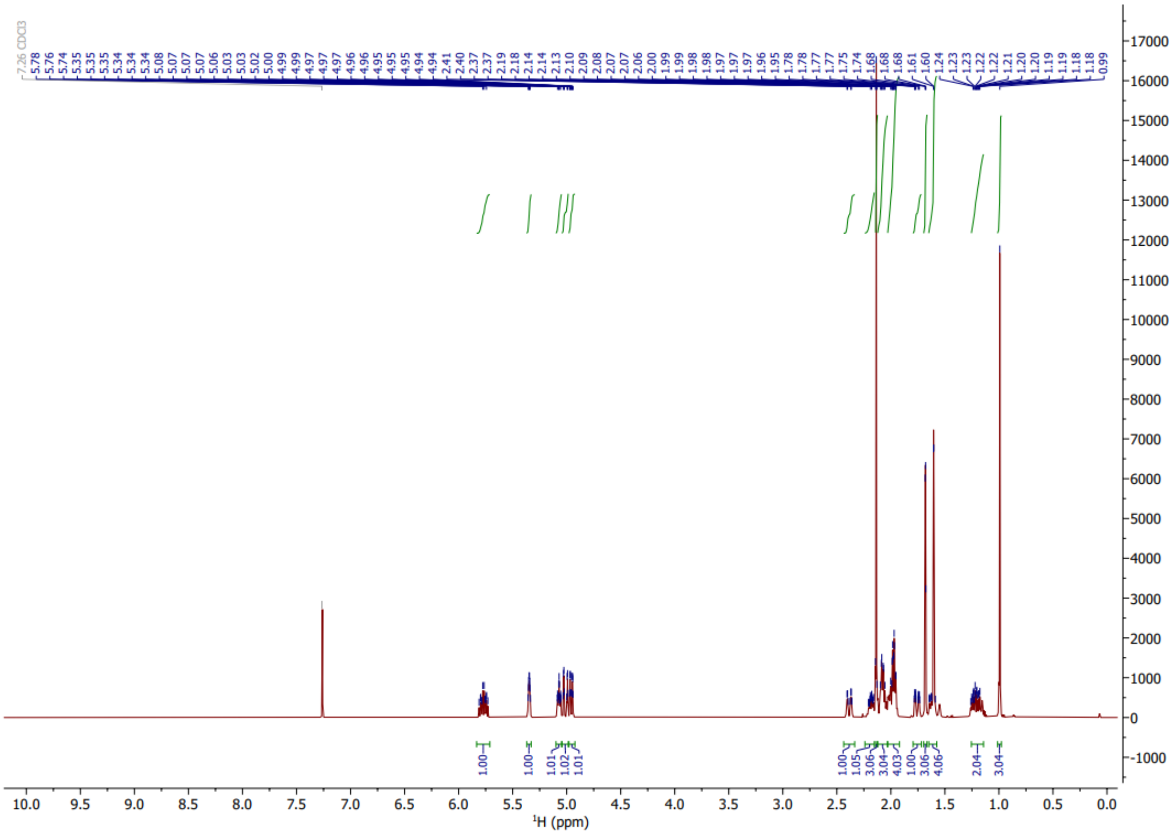

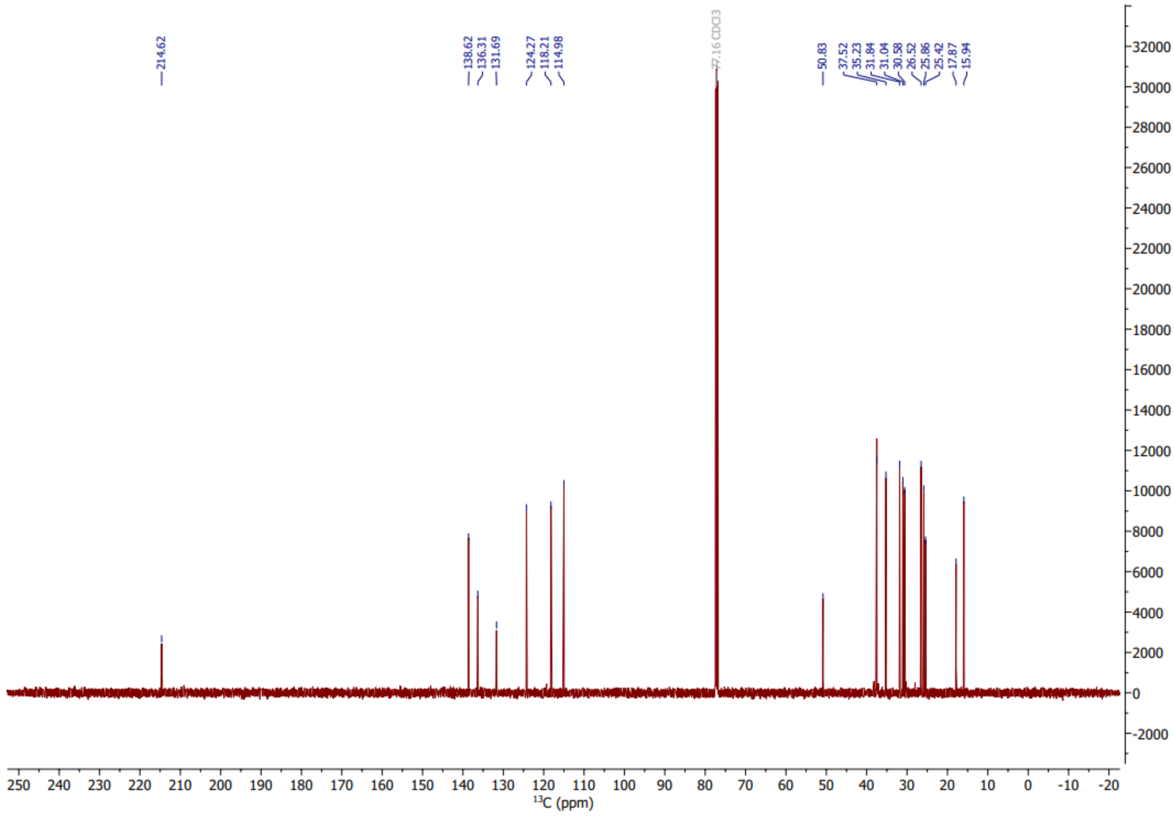

Figure S45. ^{1}H NMR and ^{13}C NMR spectra of compound **4e**.

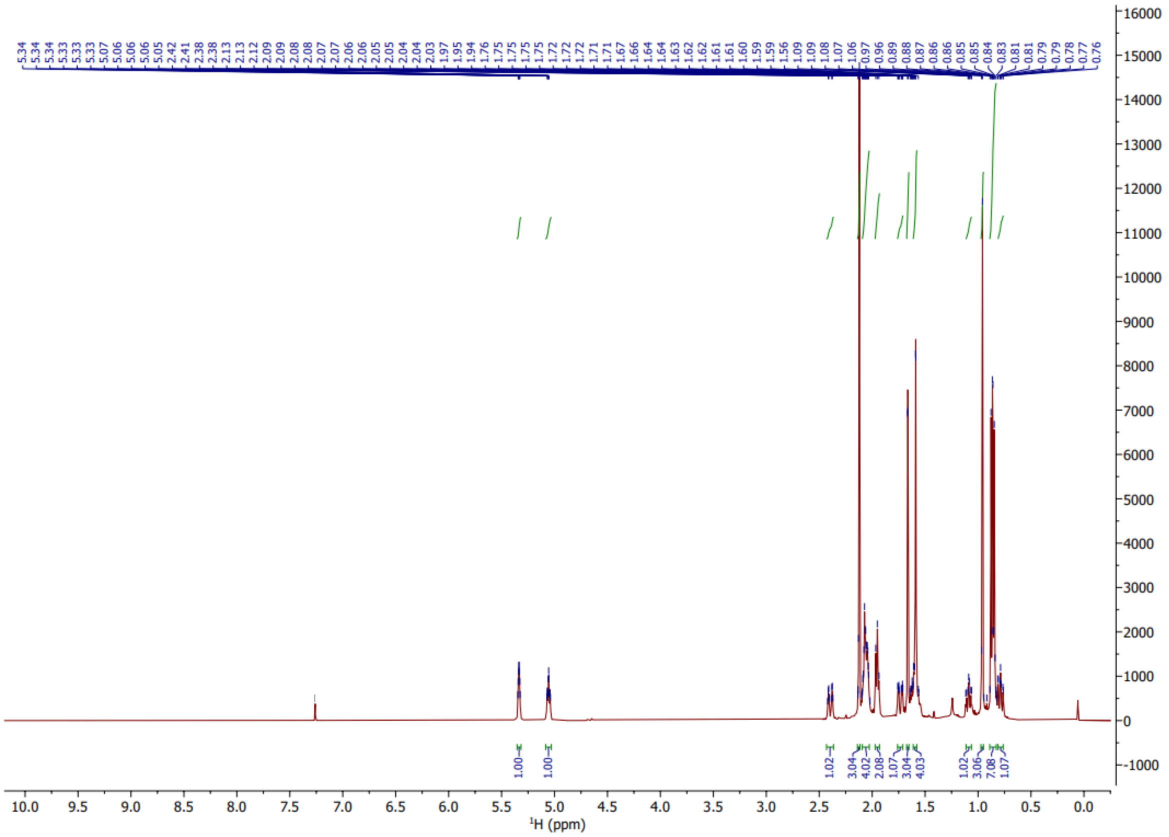

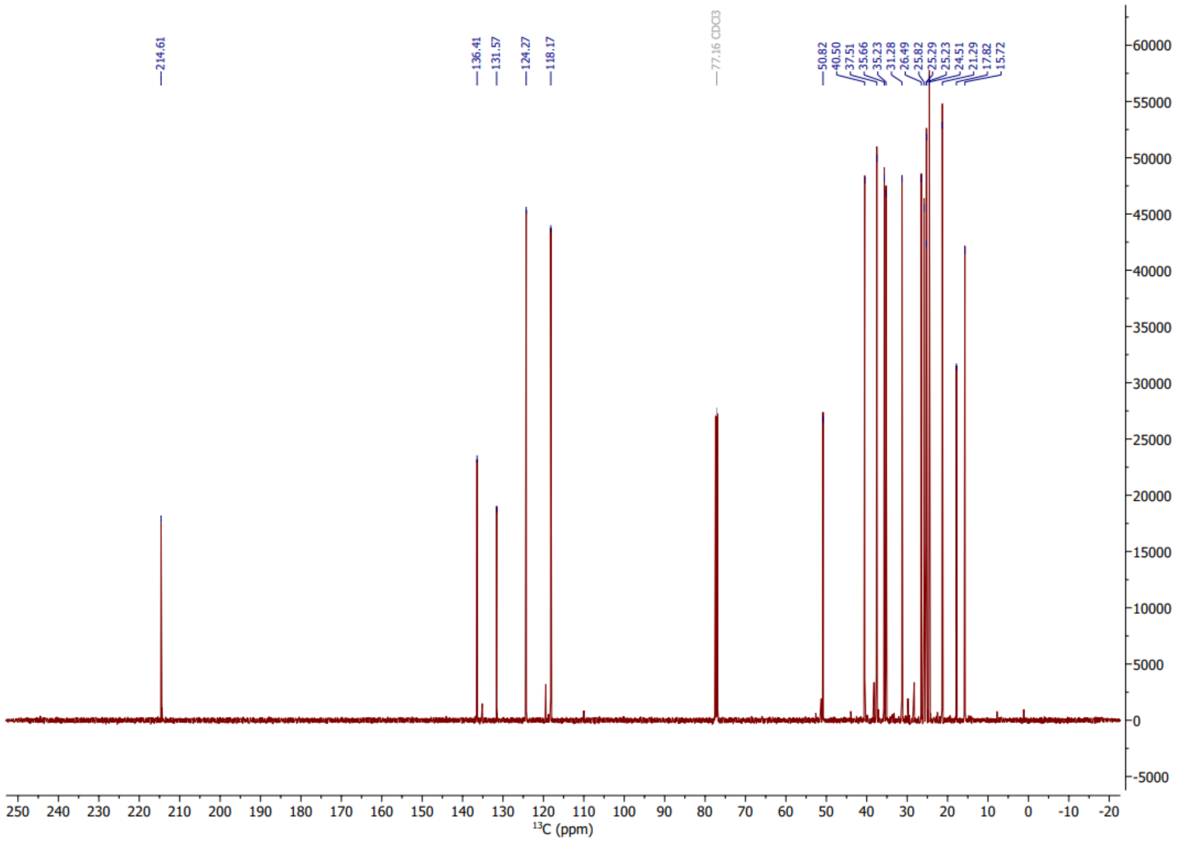

Figure S46. ^{1}H NMR and ^{13}C NMR spectra of compound **4f**.

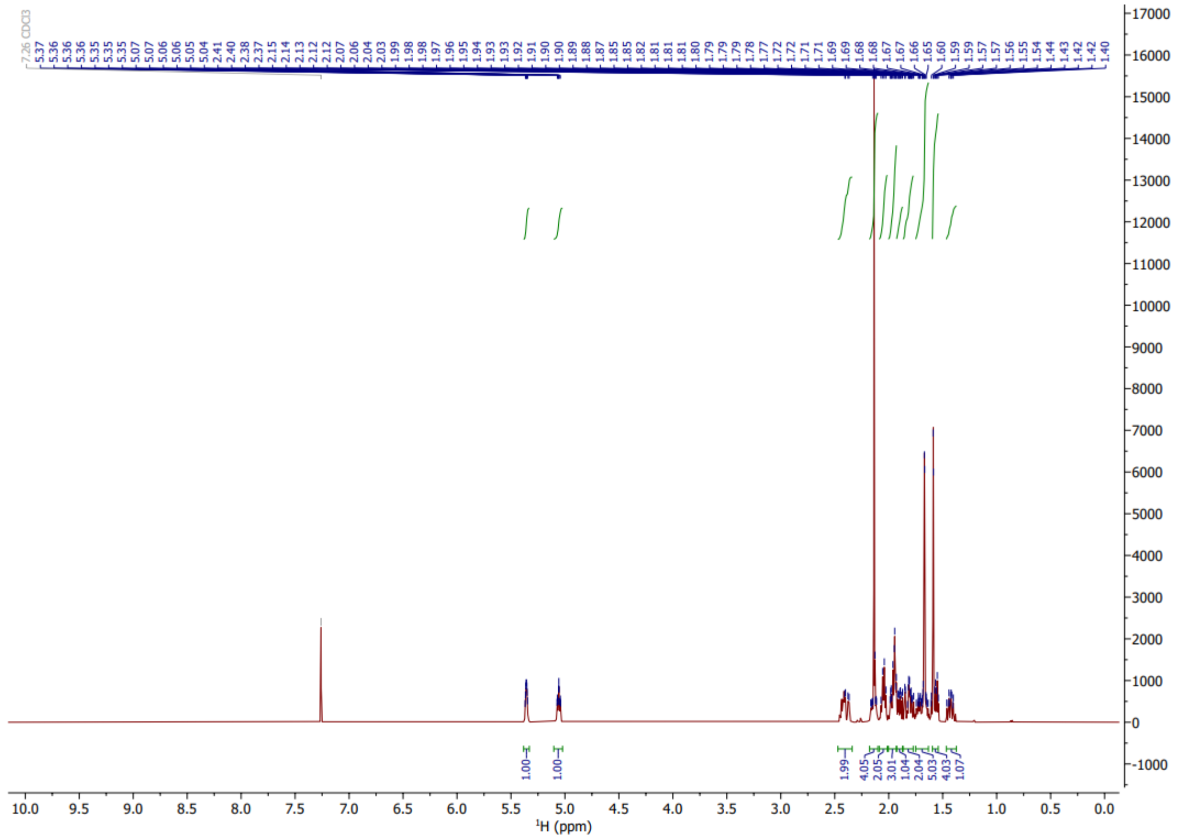

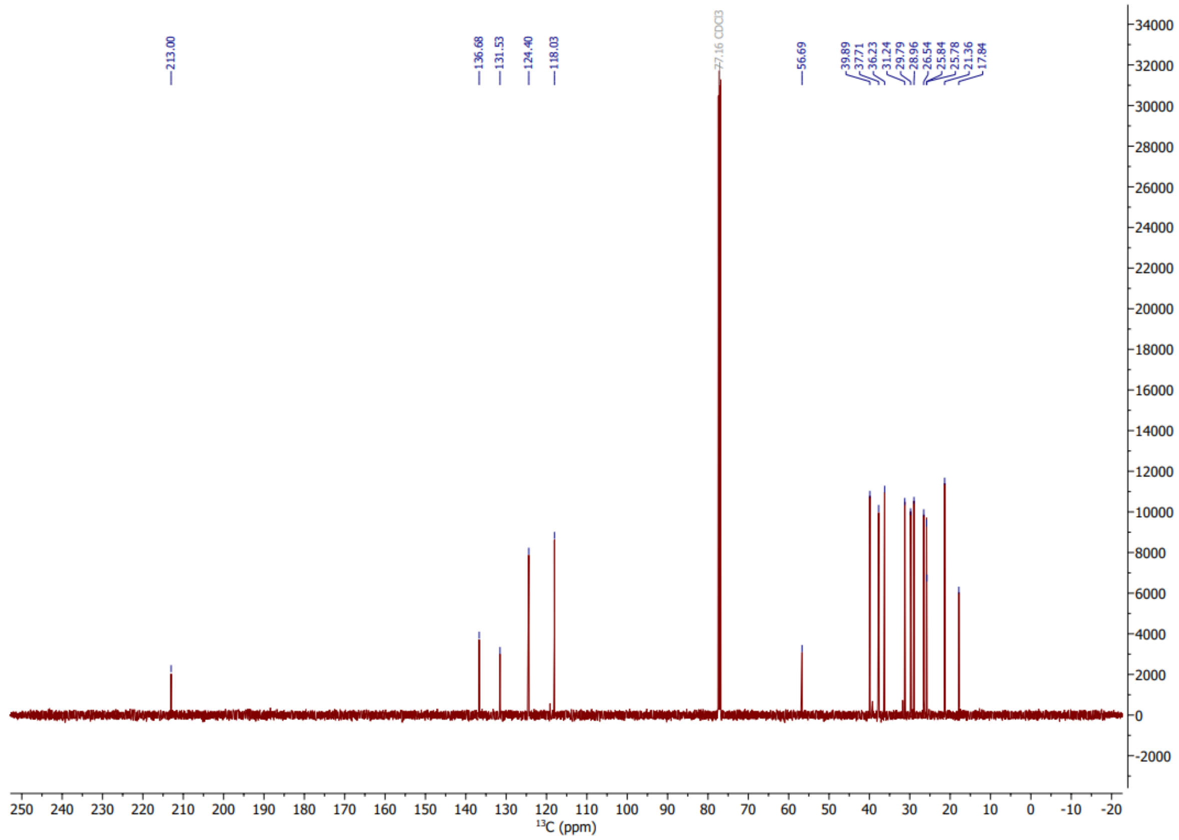

Figure S47. ^{1}H NMR and ^{13}C NMR spectra of compound **4g**.

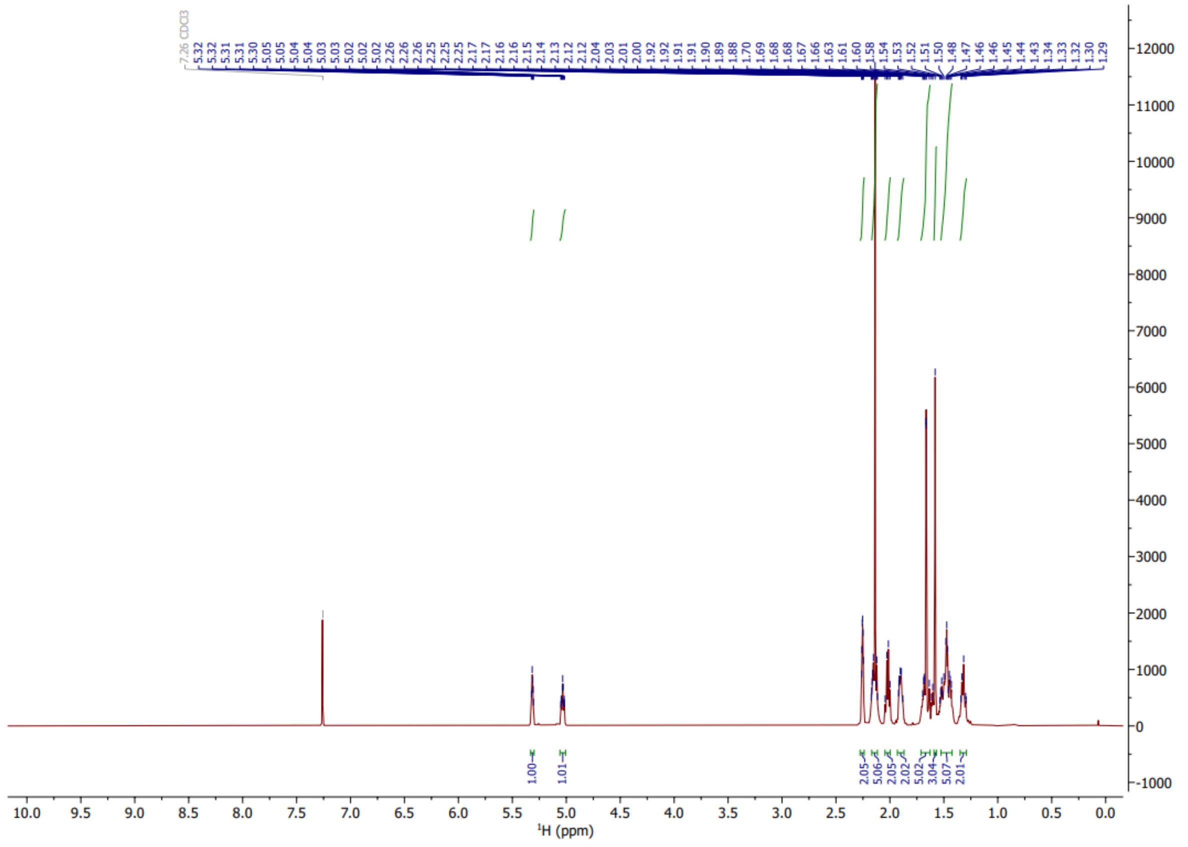

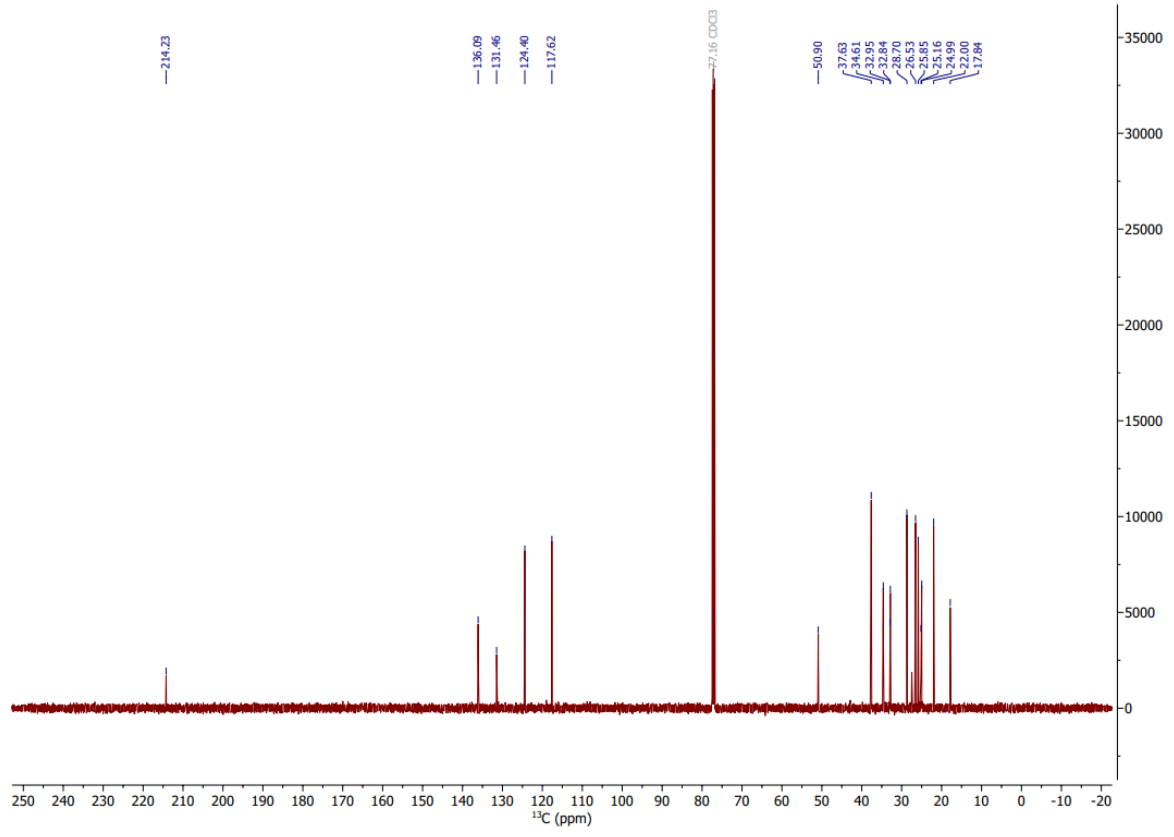

Figure S48. ^{1}H NMR and ^{13}C NMR spectra of compound **4h**.

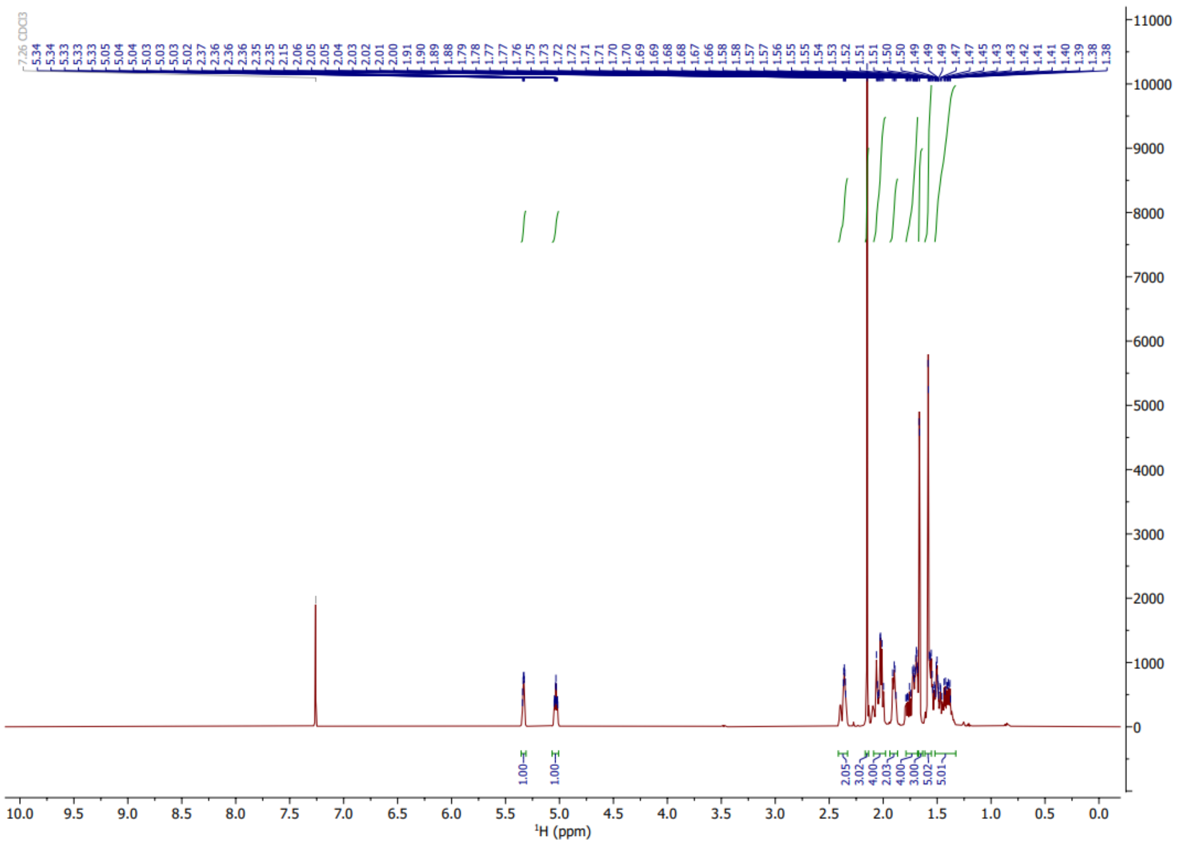

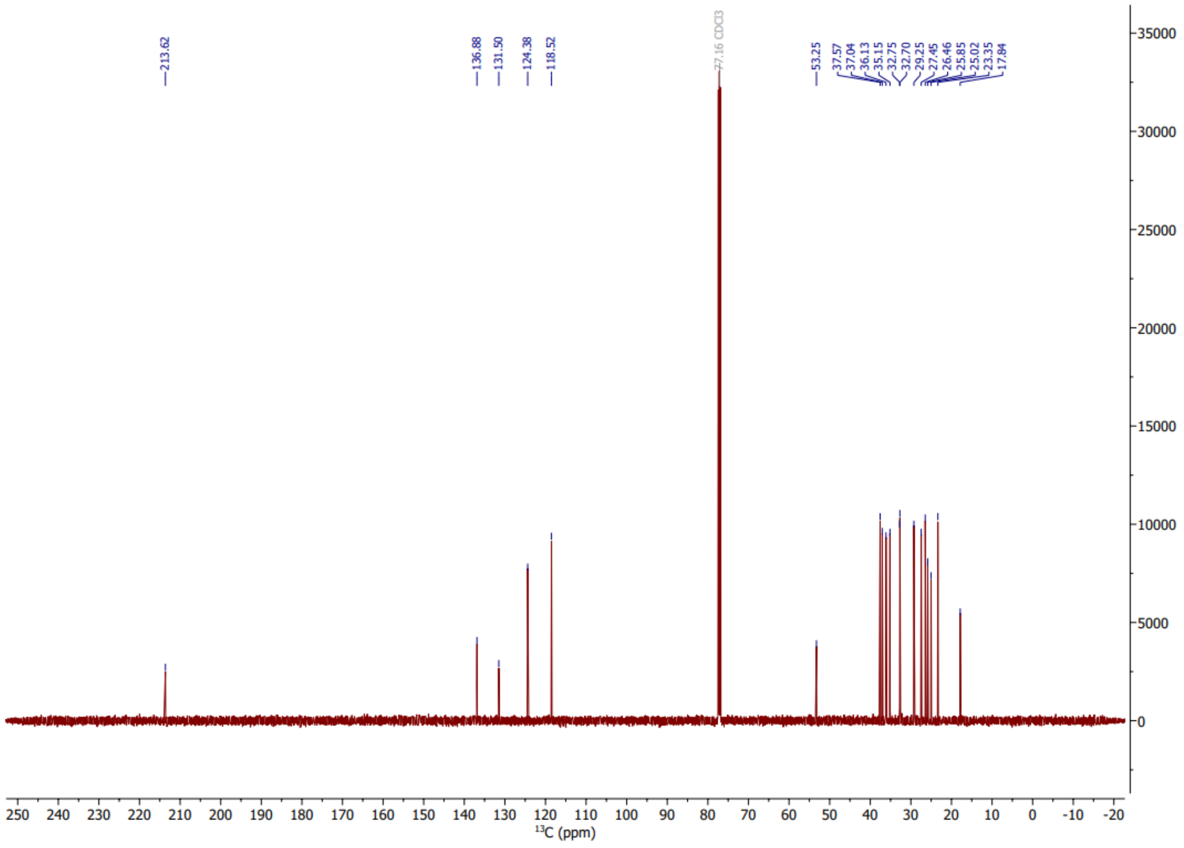

Figure S49. ^{1}H NMR and ^{13}C NMR spectra of compound **4i**.

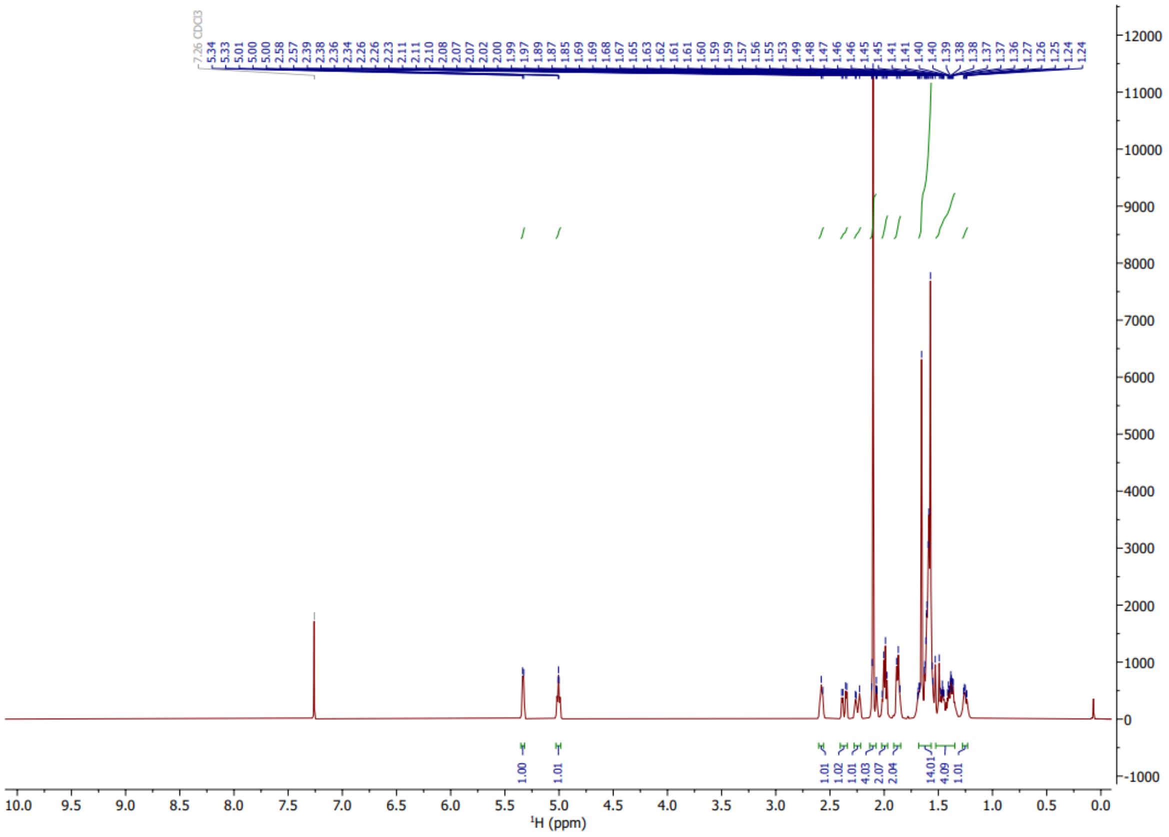

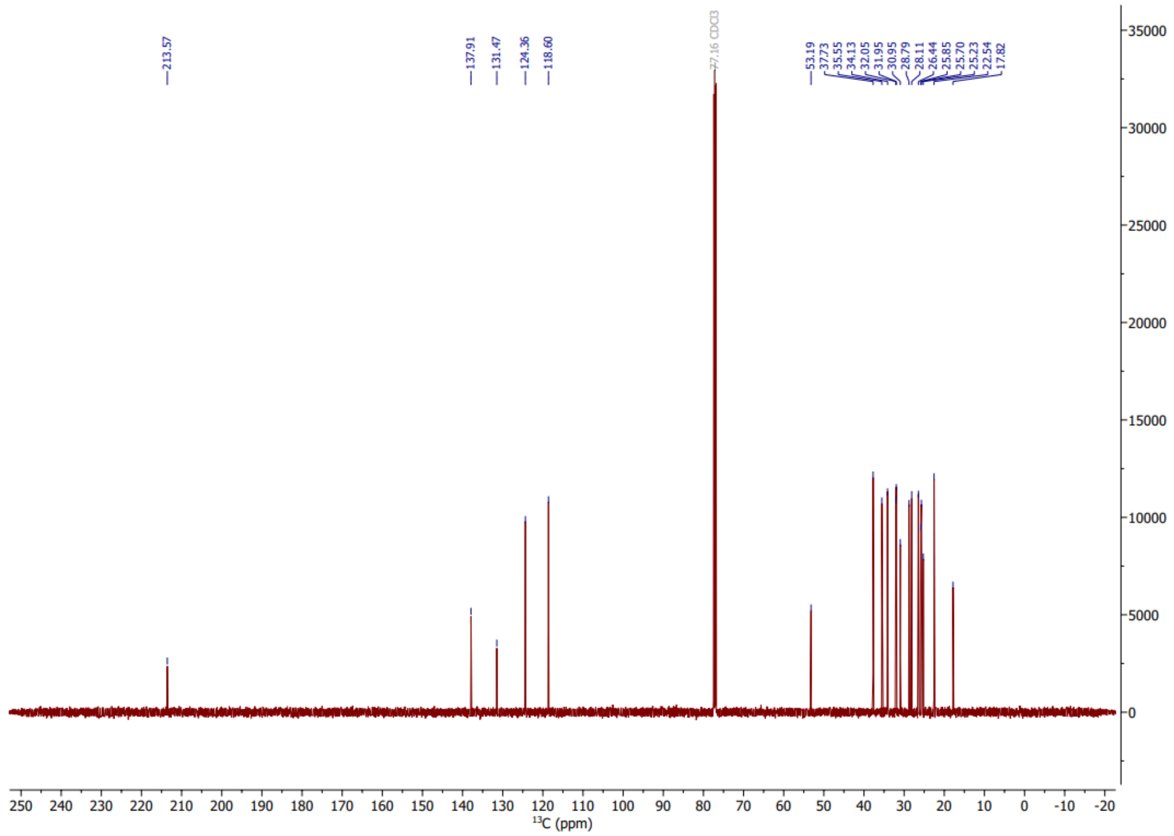

Figure S50. ^{1}H NMR and ^{13}C NMR spectra of compound **4j**.

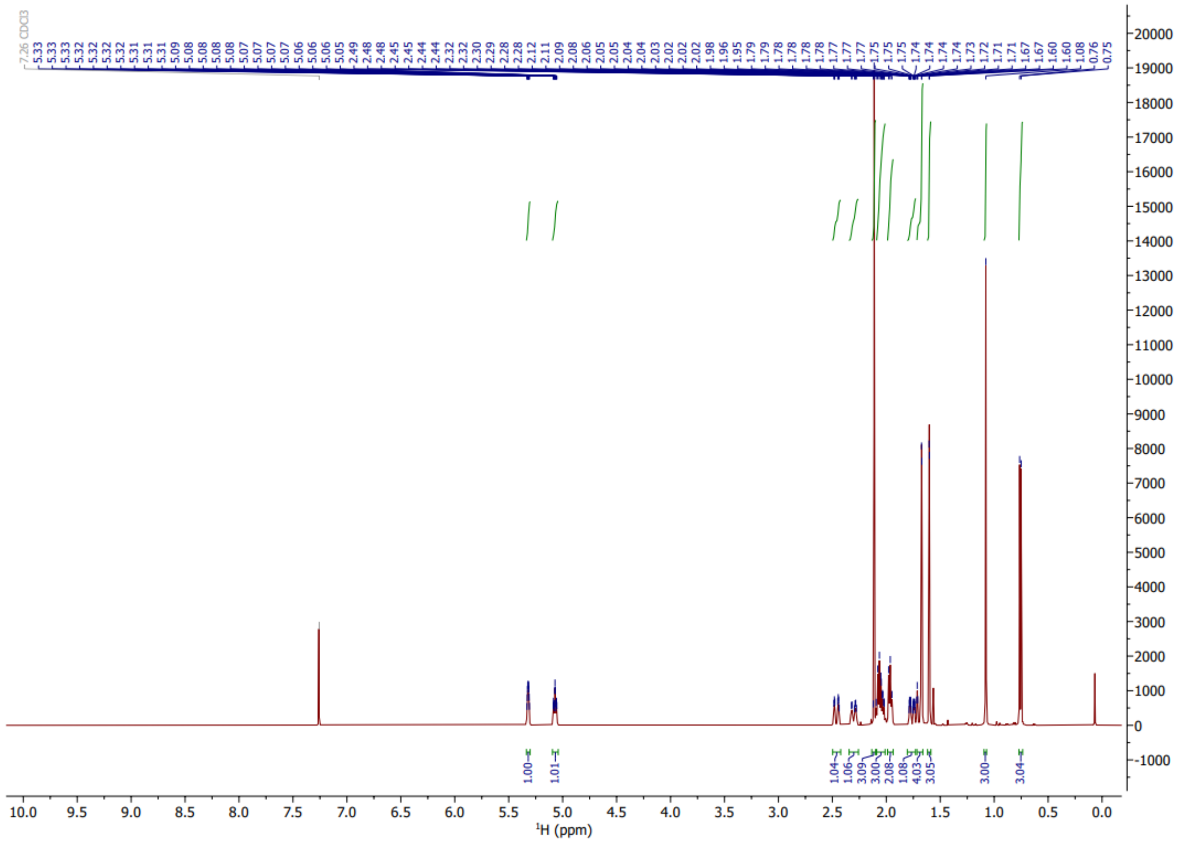

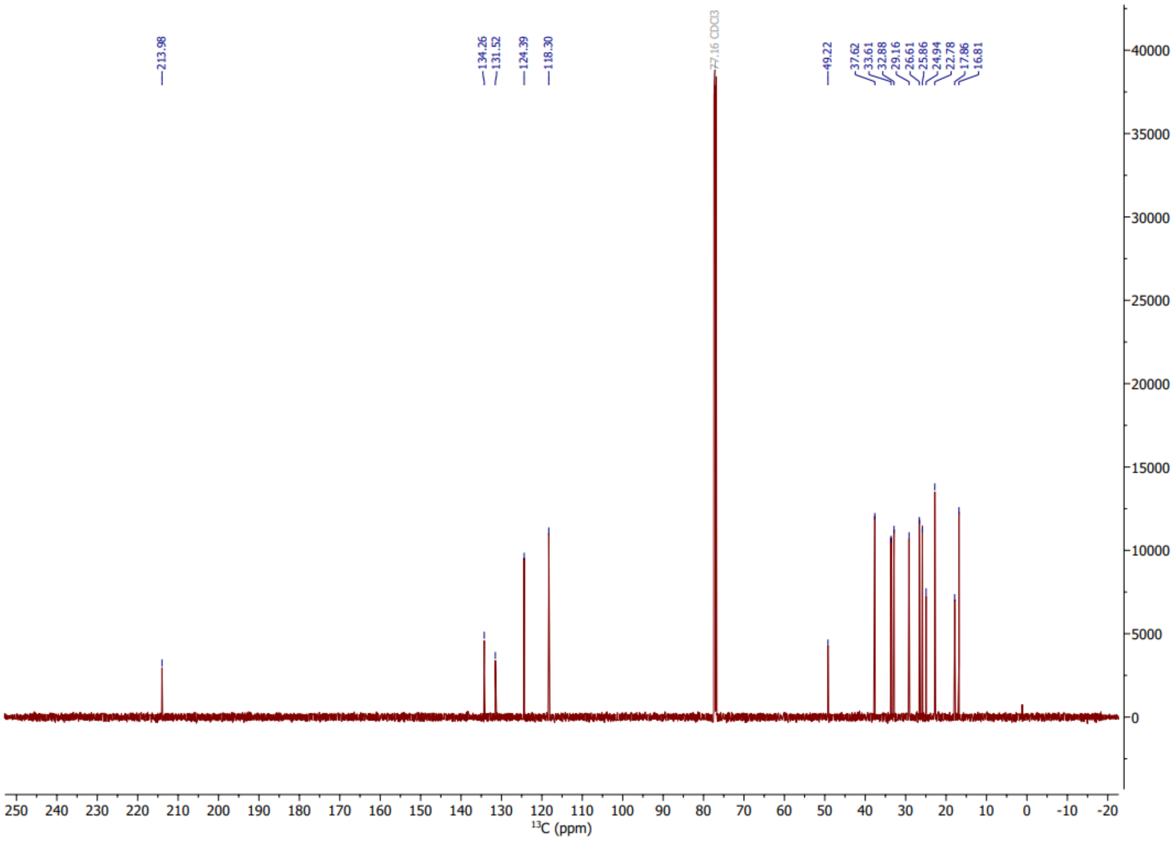

Figure S51. ^{1}H NMR and ^{13}C NMR spectra of compound **4k**.

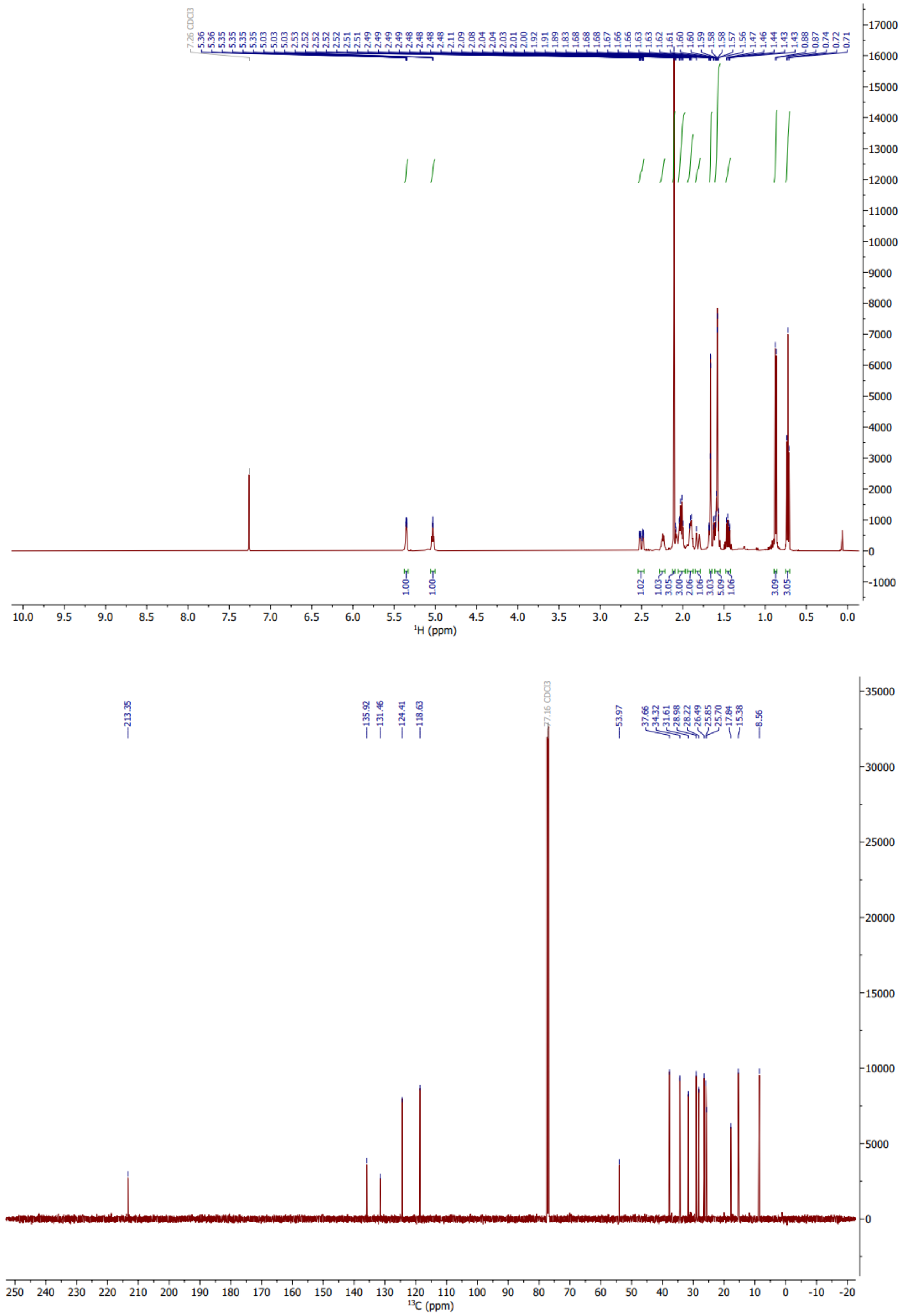

Figure S52. ^{1}H NMR and ^{13}C NMR spectra of compound **4l**.

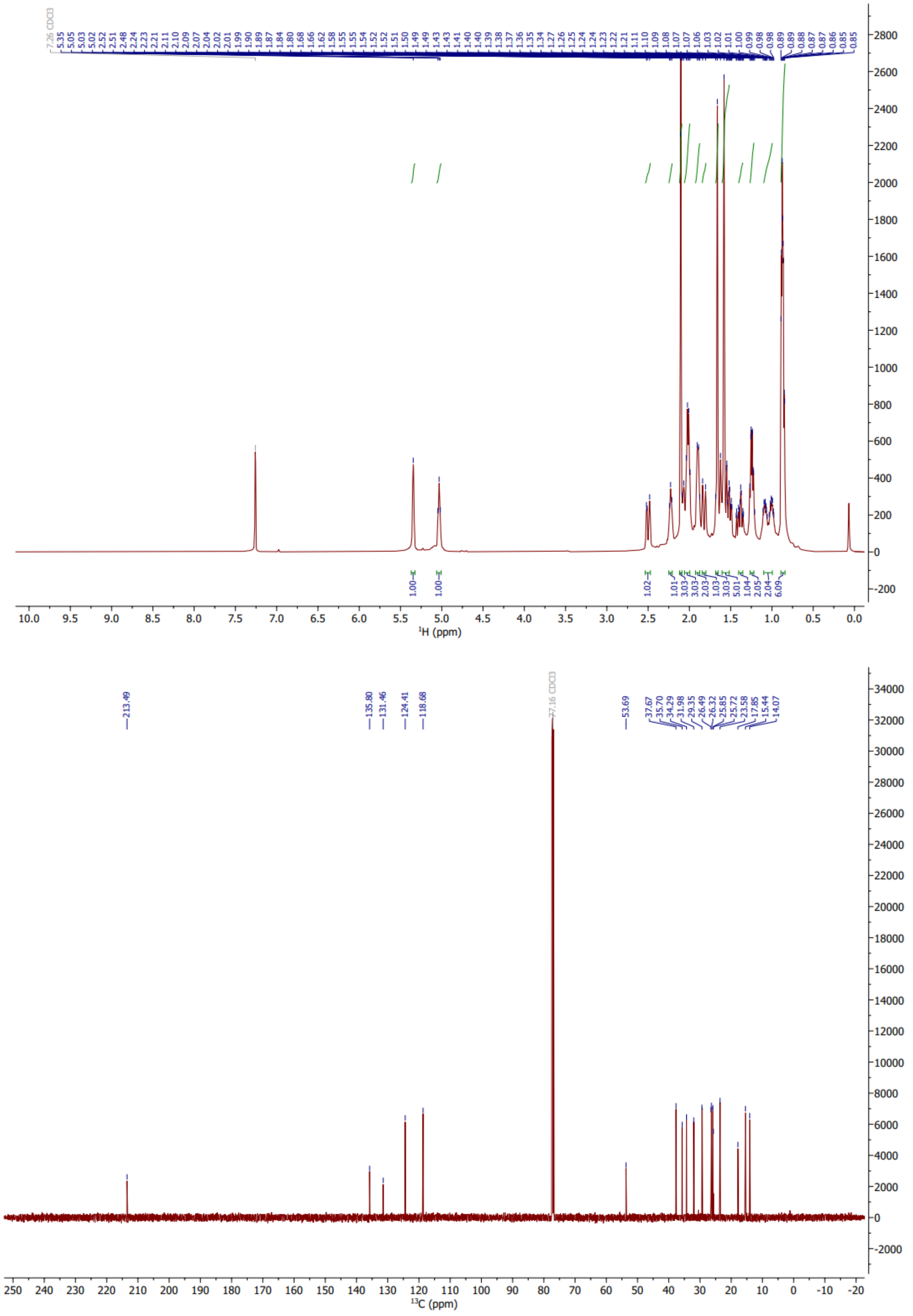

Figure S53. ^{1}H NMR and ^{13}C NMR spectra of compound **4m**.

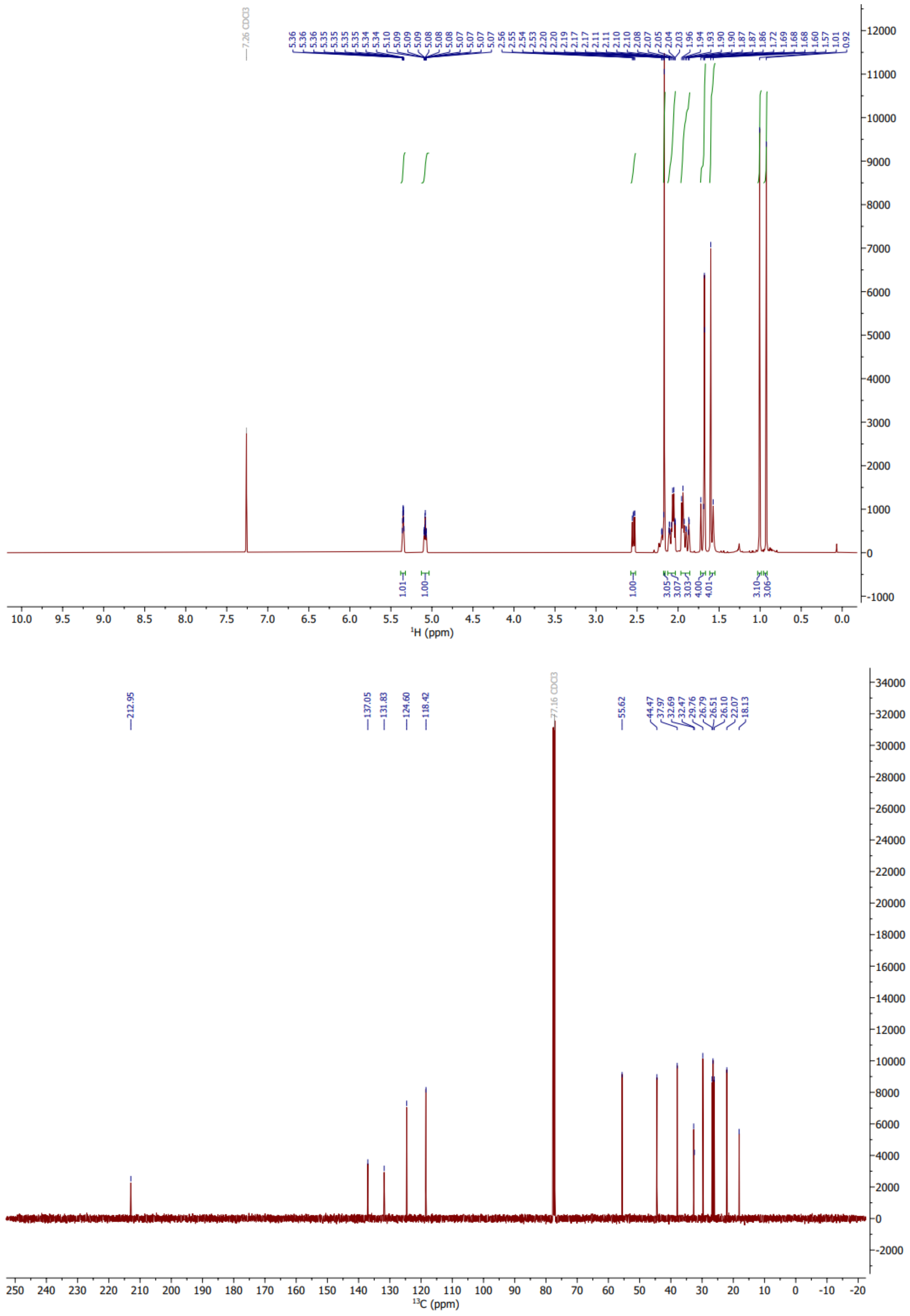

Figure S54. ^{1}H NMR and ^{13}C NMR spectra of compound **4n**.

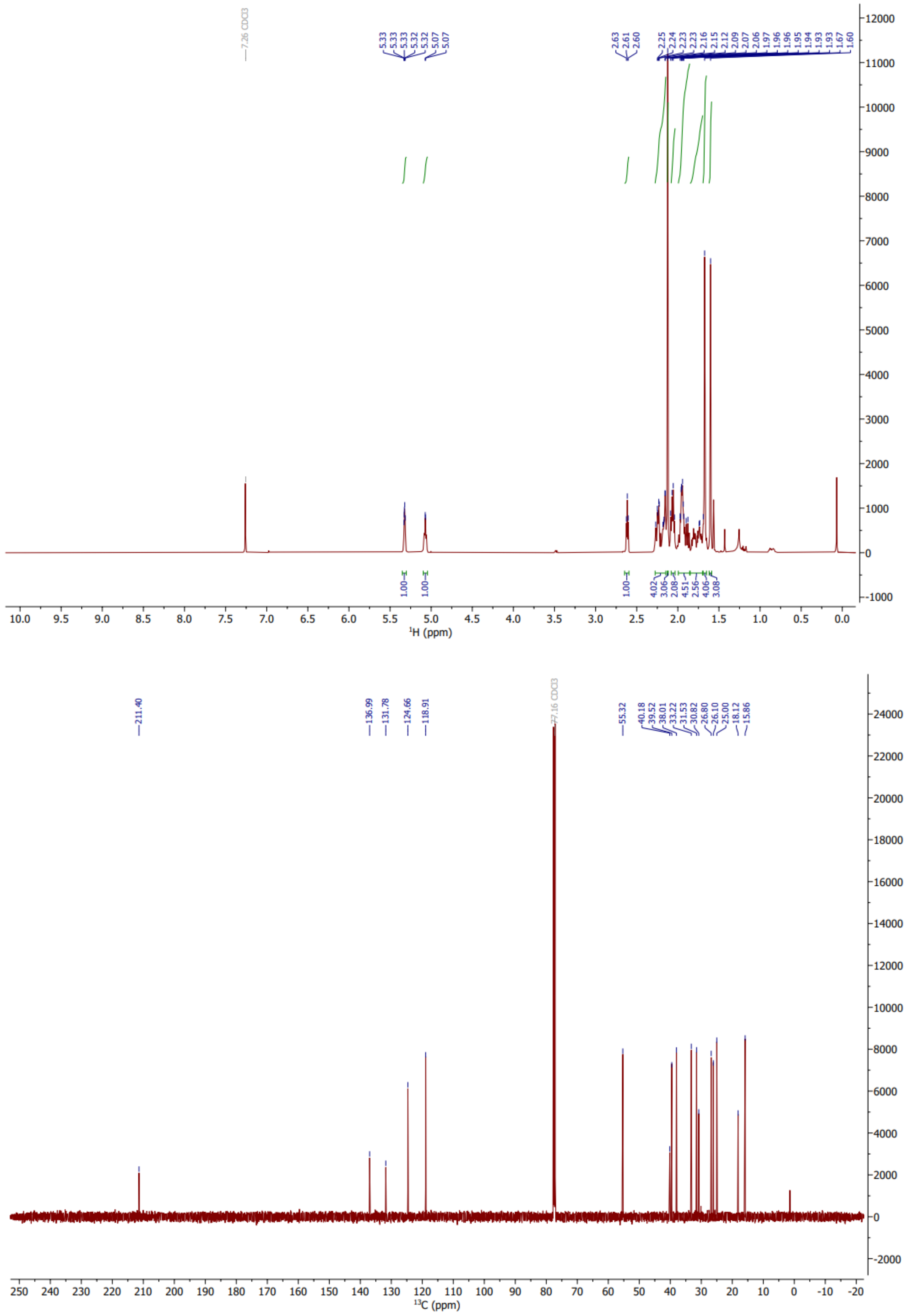

Figure S55. ^{1}H NMR and ^{13}C NMR spectra of compound **4o**.

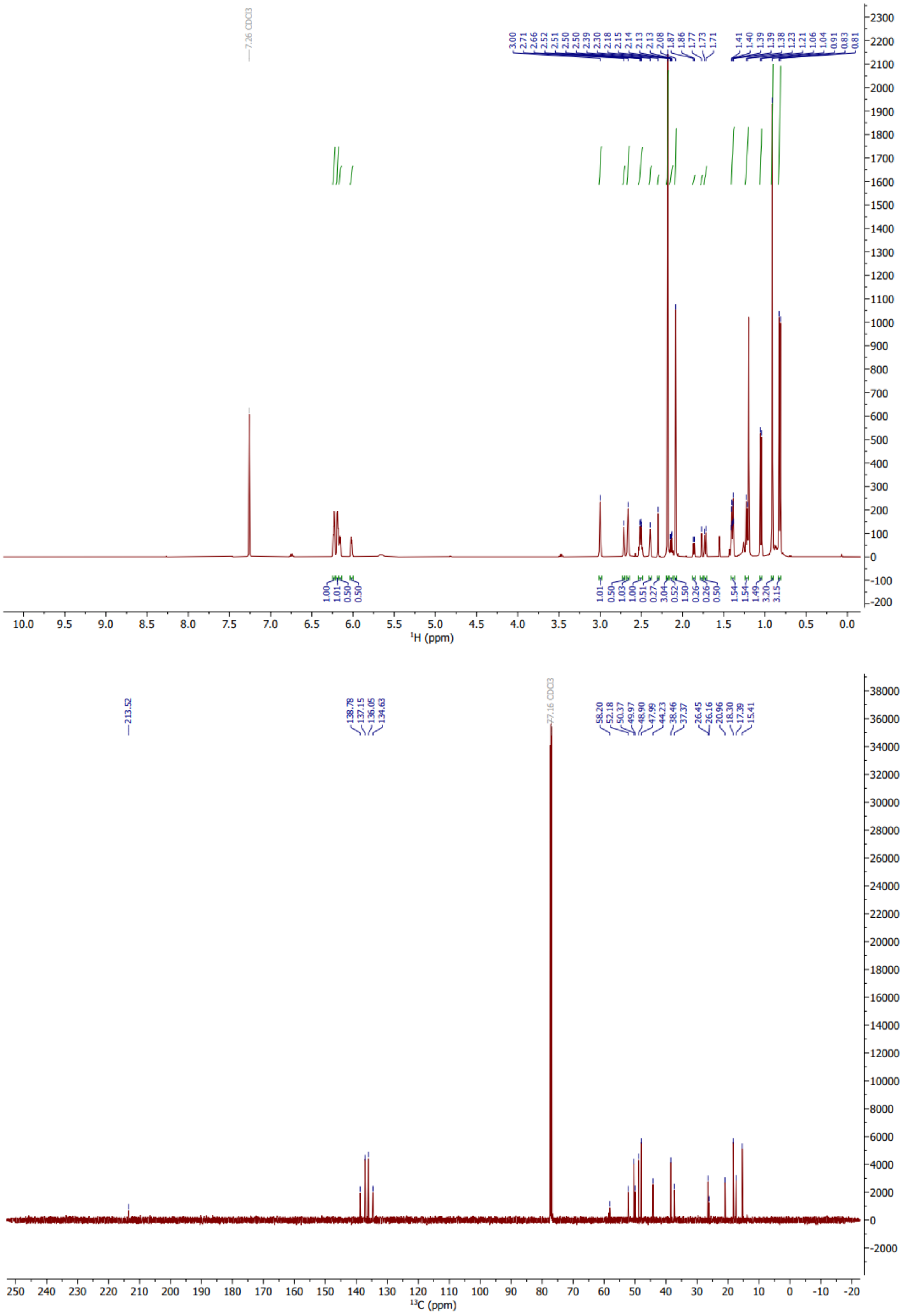

Figure S56. ^{1}H NMR and ^{13}C NMR spectra of compound **4p**.

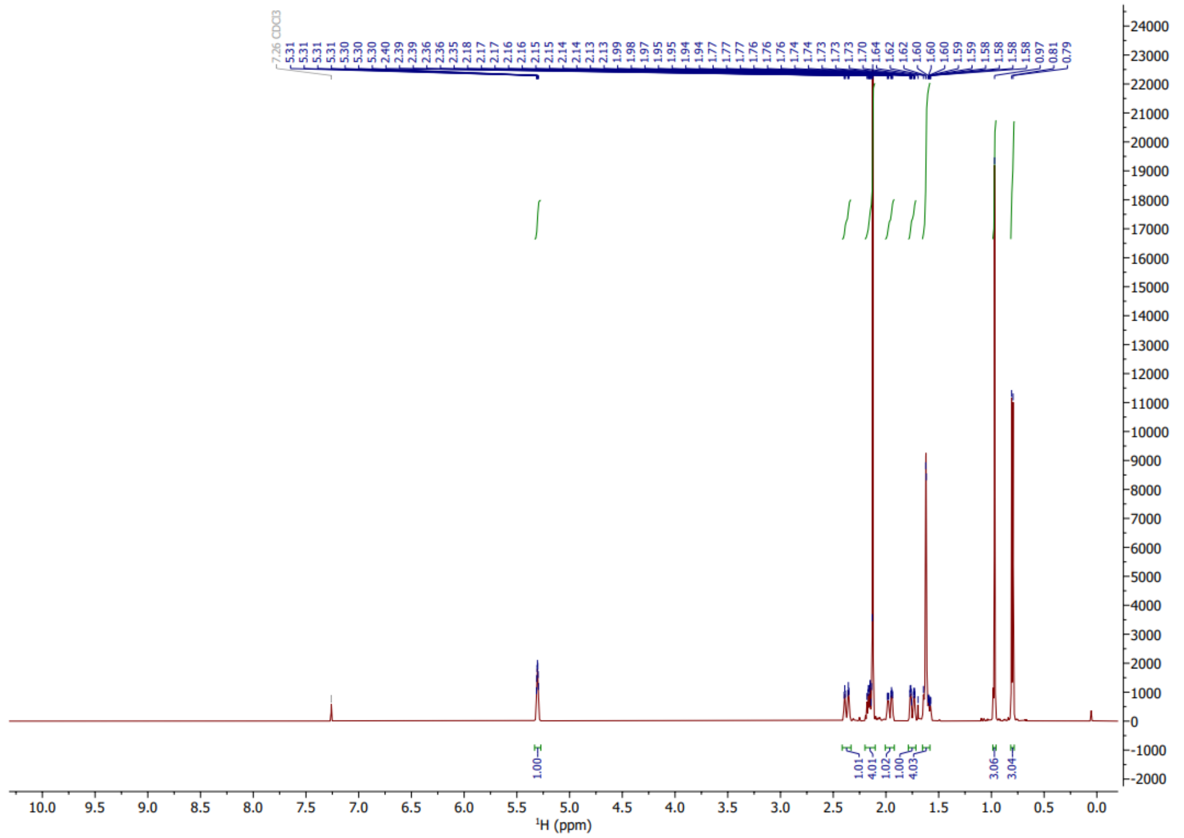

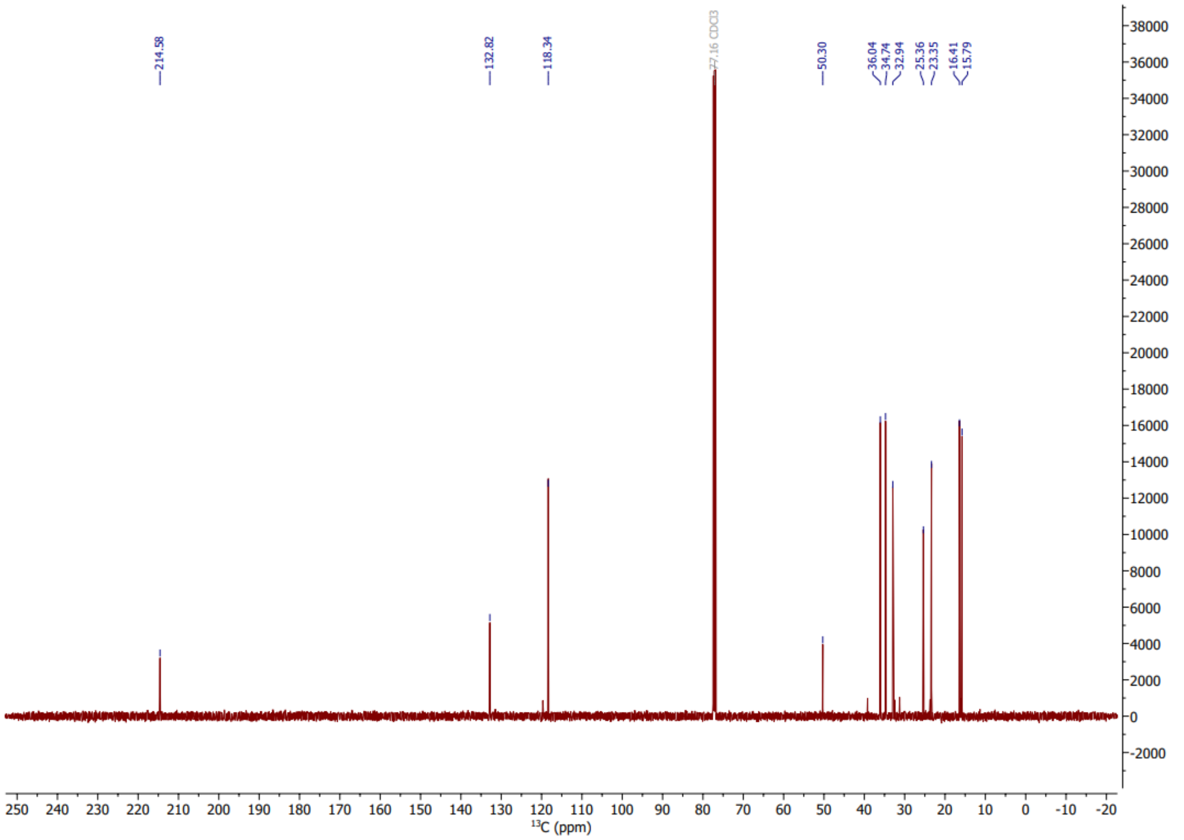

Figure S57. ^{1}H NMR and ^{13}C NMR spectra of compound **4q**.

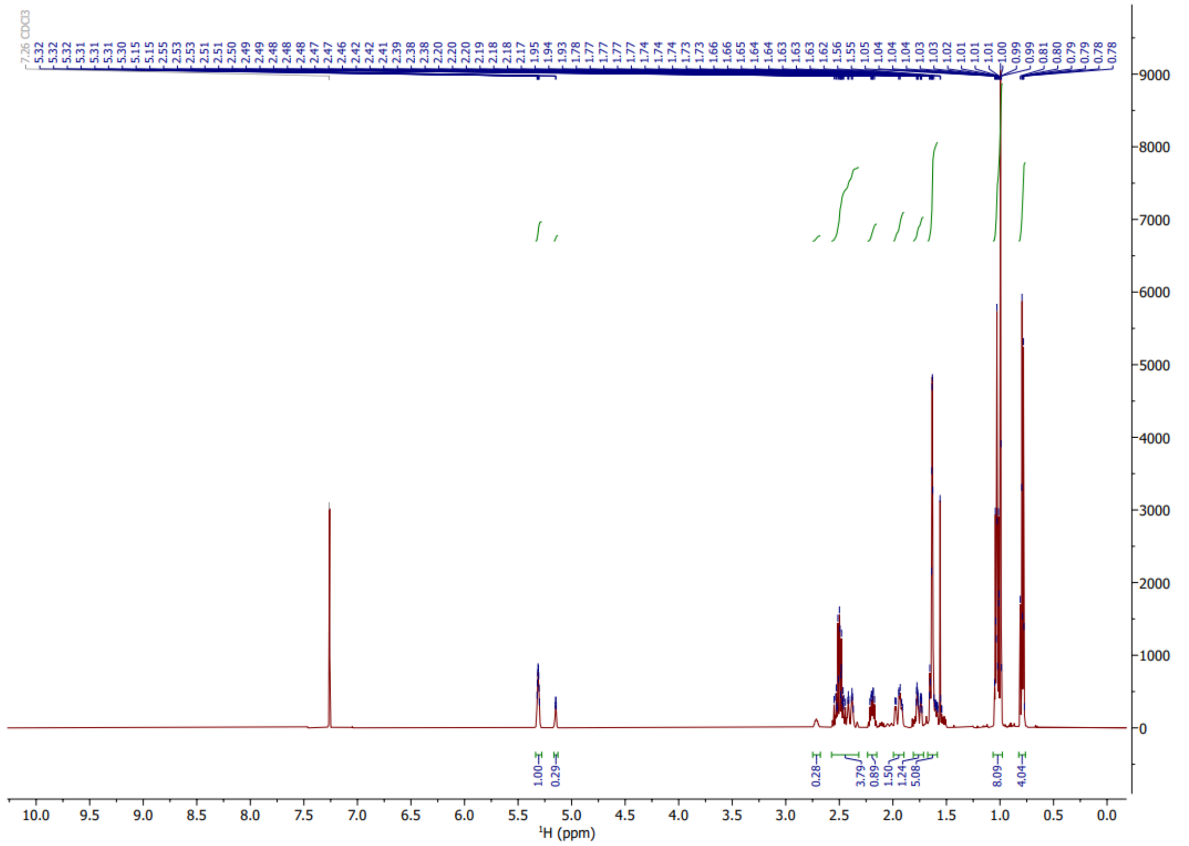

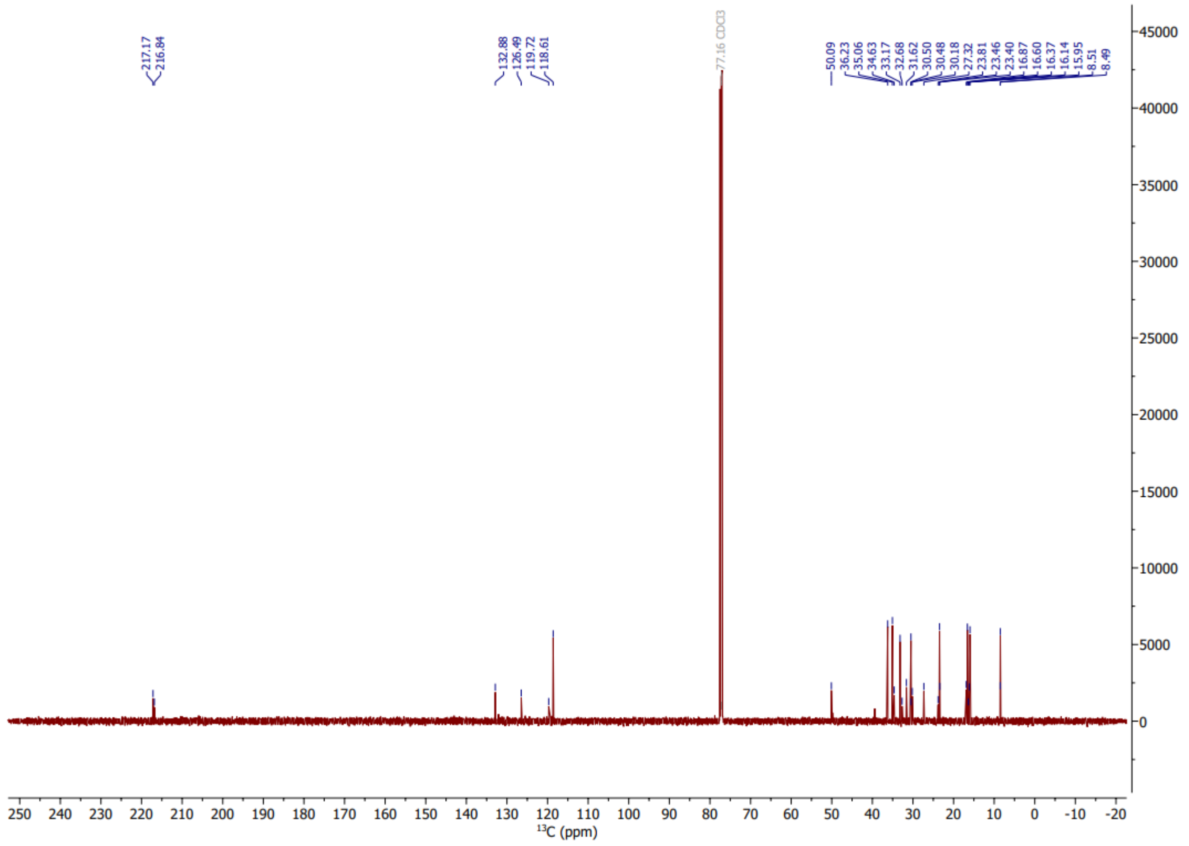

Figure S58. ^{1}H NMR and ^{13}C NMR spectra of compound **4q’**.

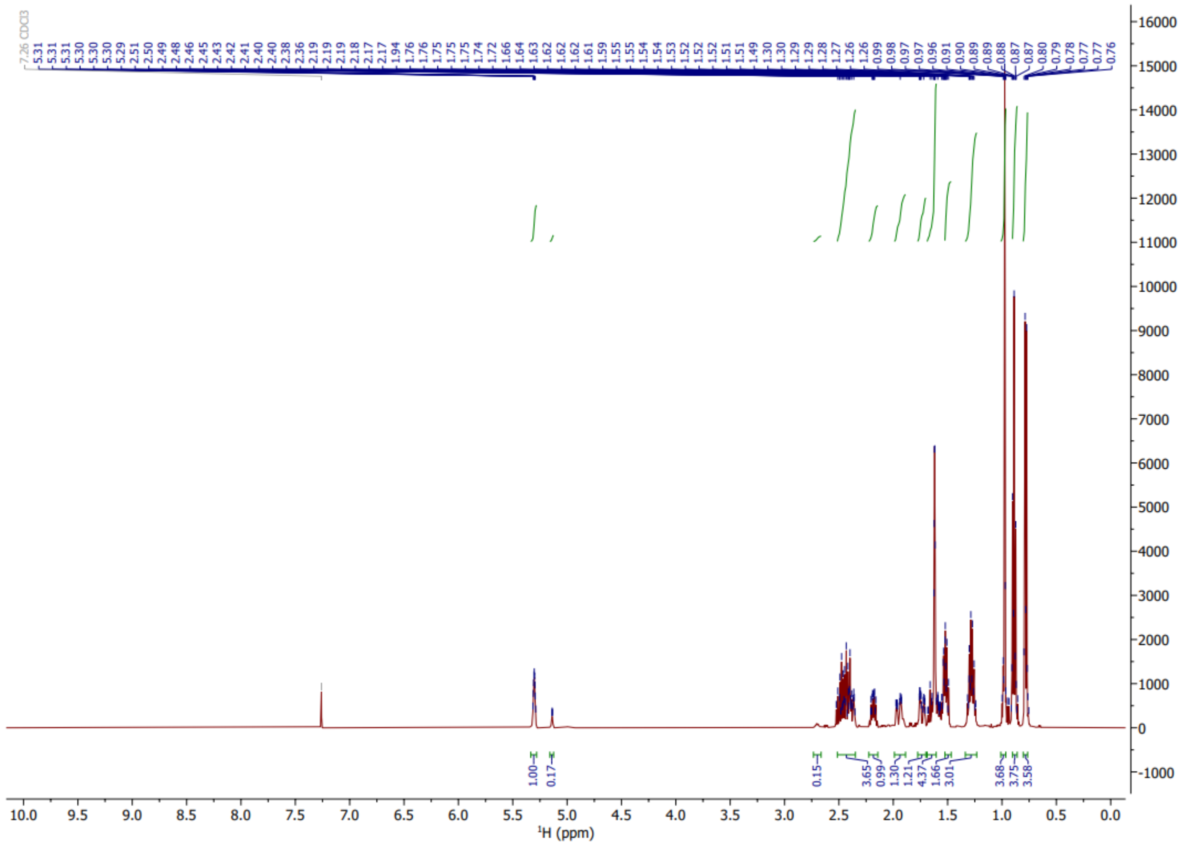

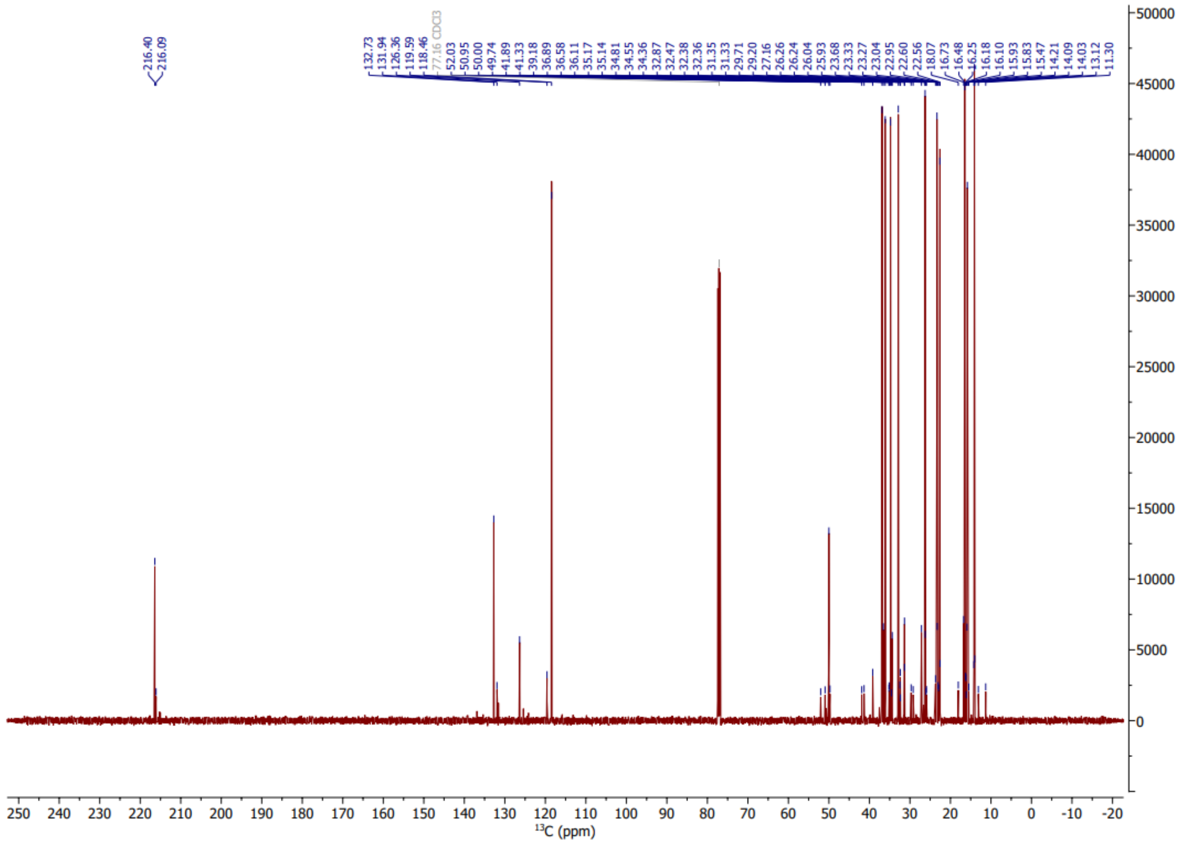

Figure S59. ^{1}H NMR and ^{13}C NMR spectra of compound **4q''**.

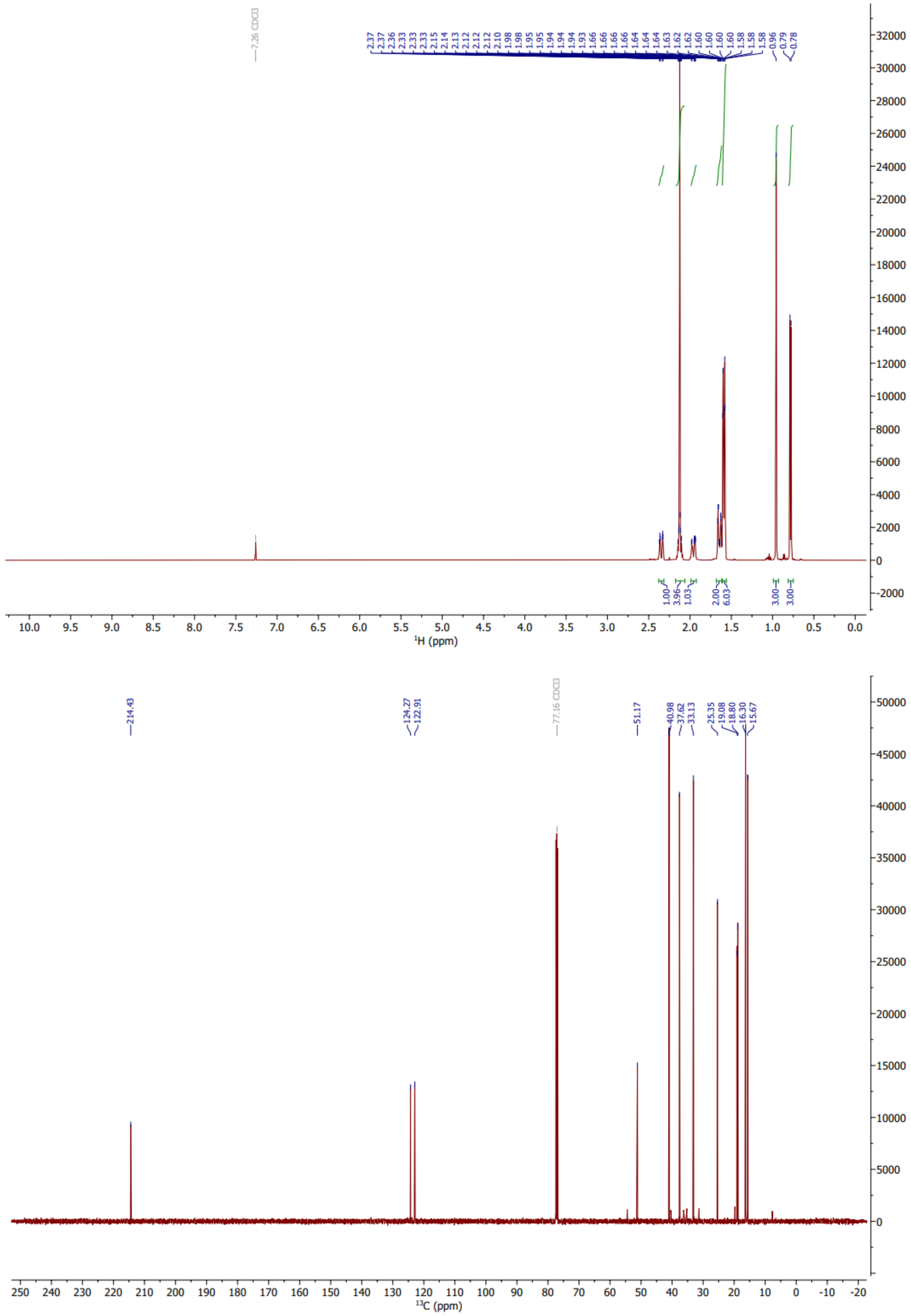

Figure S60. ^{1}H NMR and ^{13}C NMR spectra of compound **4r**.

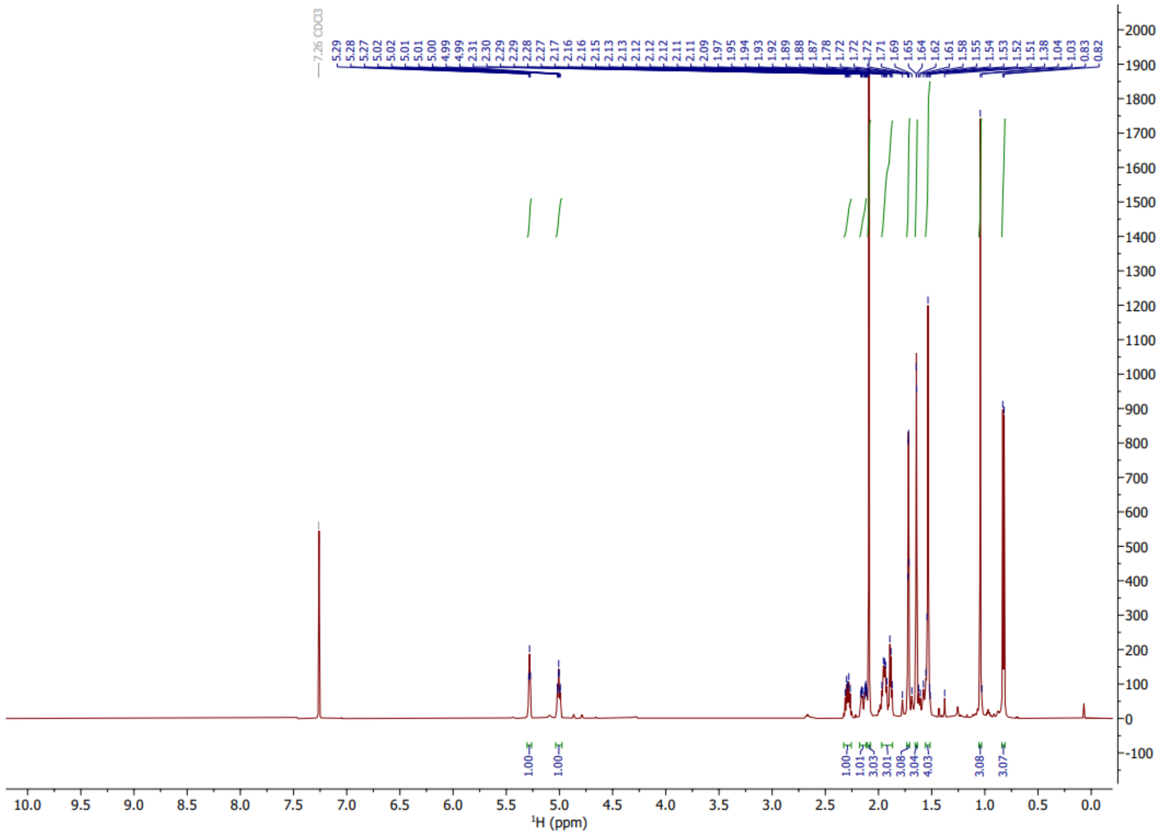

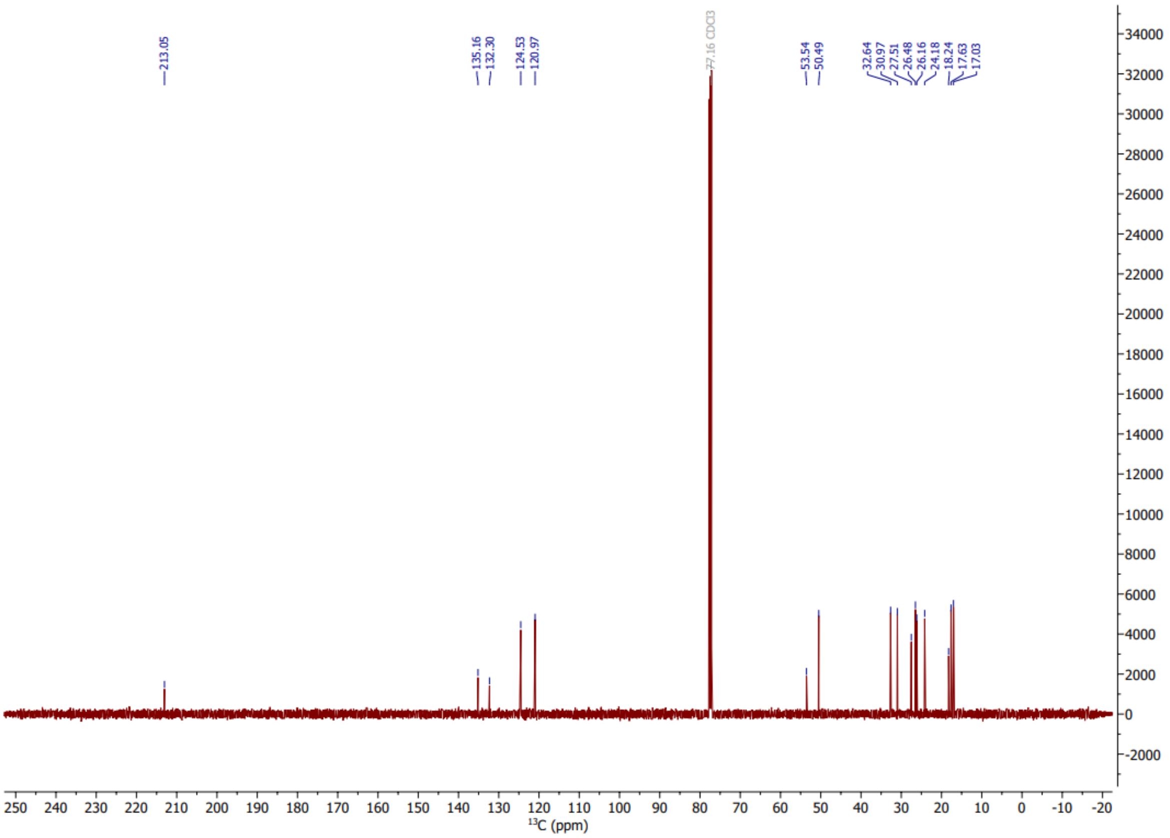

Figure S61. ^{1}H NMR and ^{13}C NMR spectra of compound **4s**.

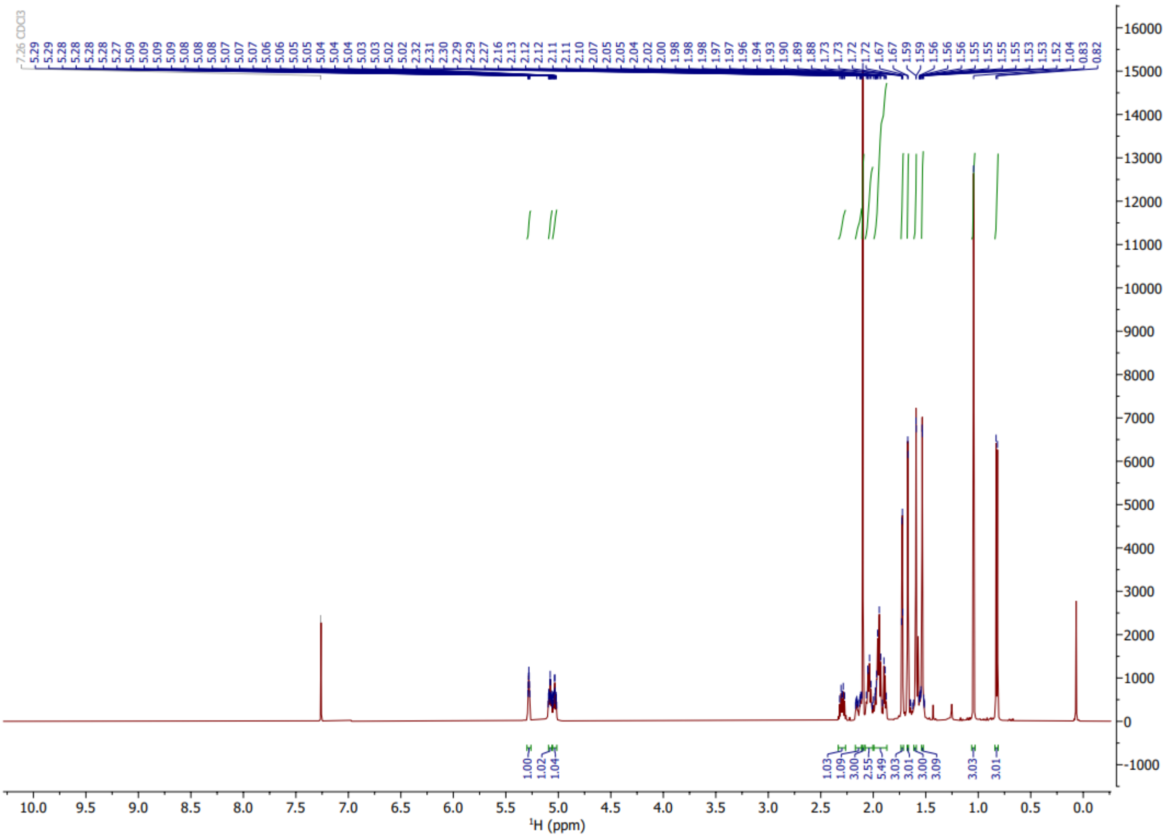

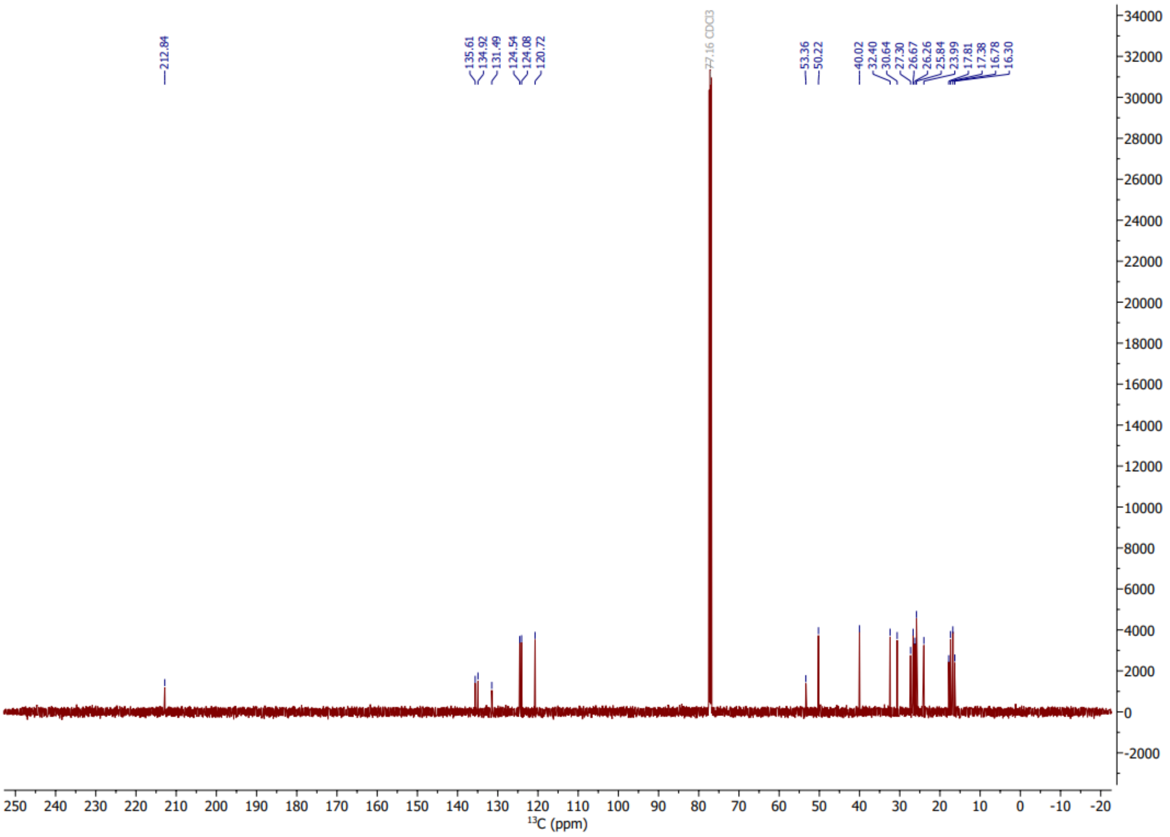

Figure S62. ^{1}H NMR and ^{13}C NMR spectra of compound **4t**.

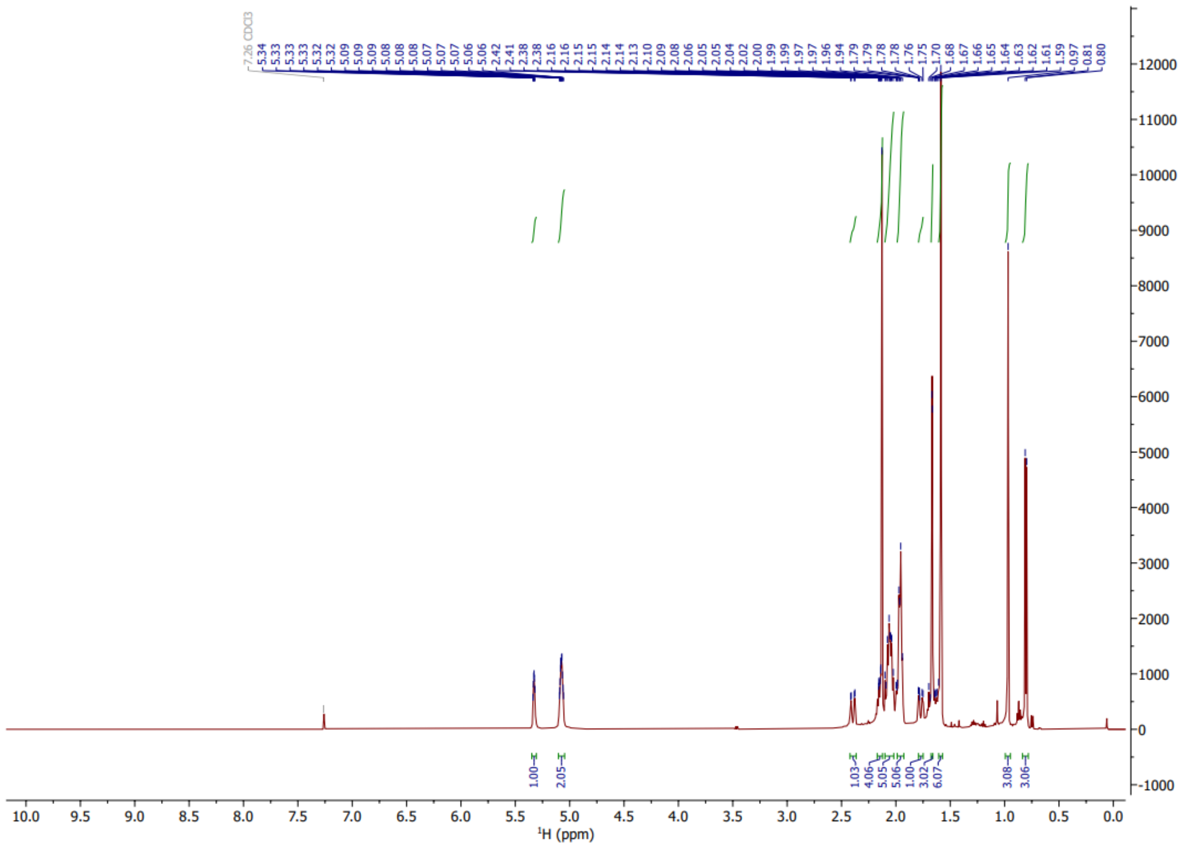

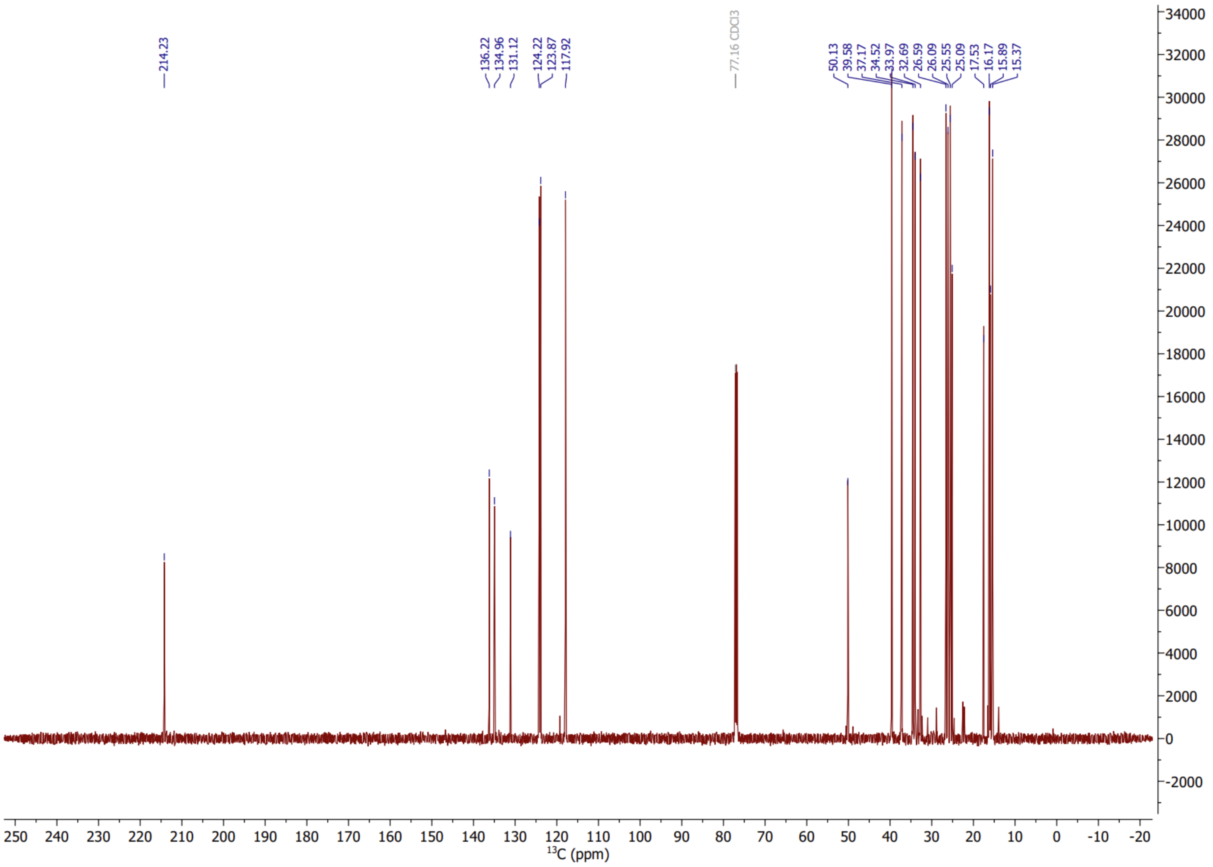

Figure S63. ^{1}H NMR and ^{13}C NMR spectra of compound **4u**.

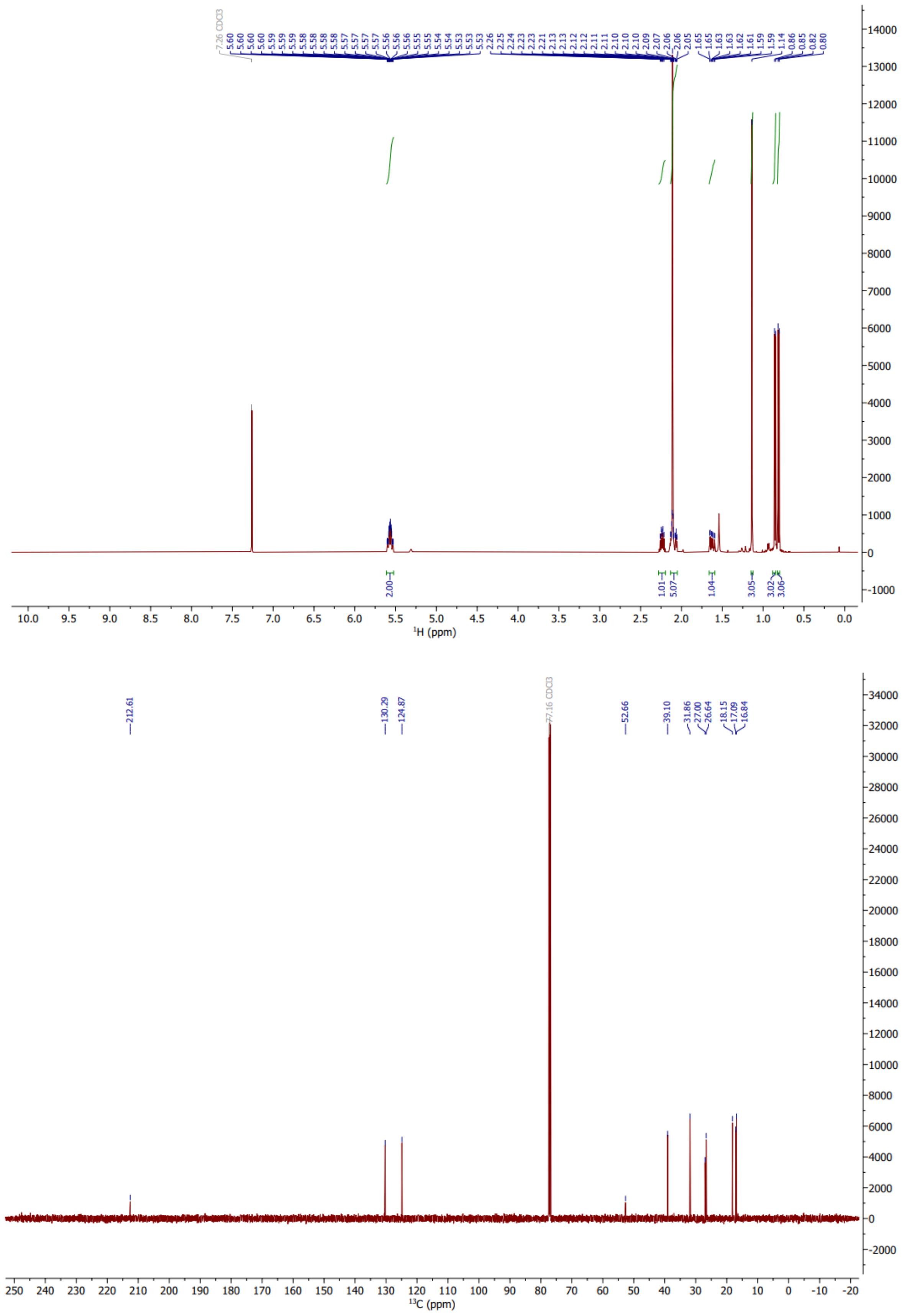

Figure S64. ^{1}H NMR and ^{13}C NMR spectra of compound **4v**.

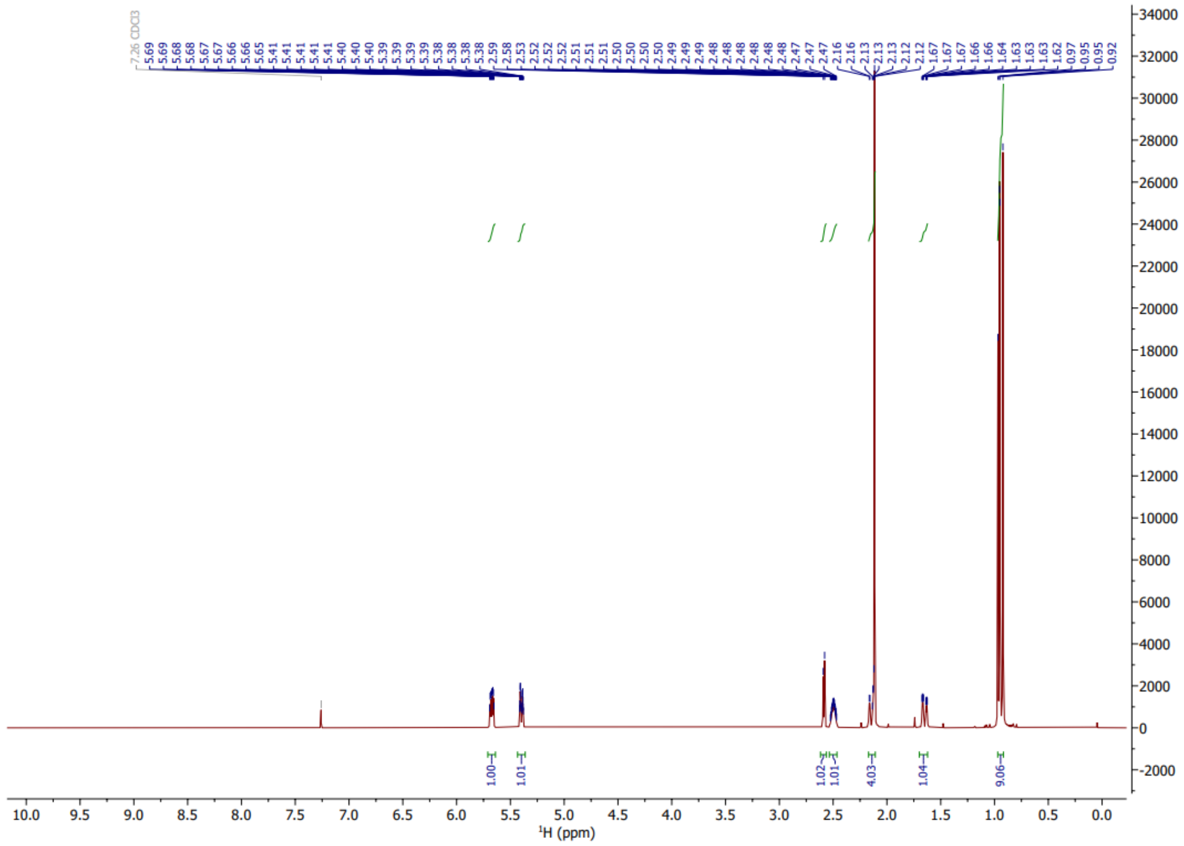

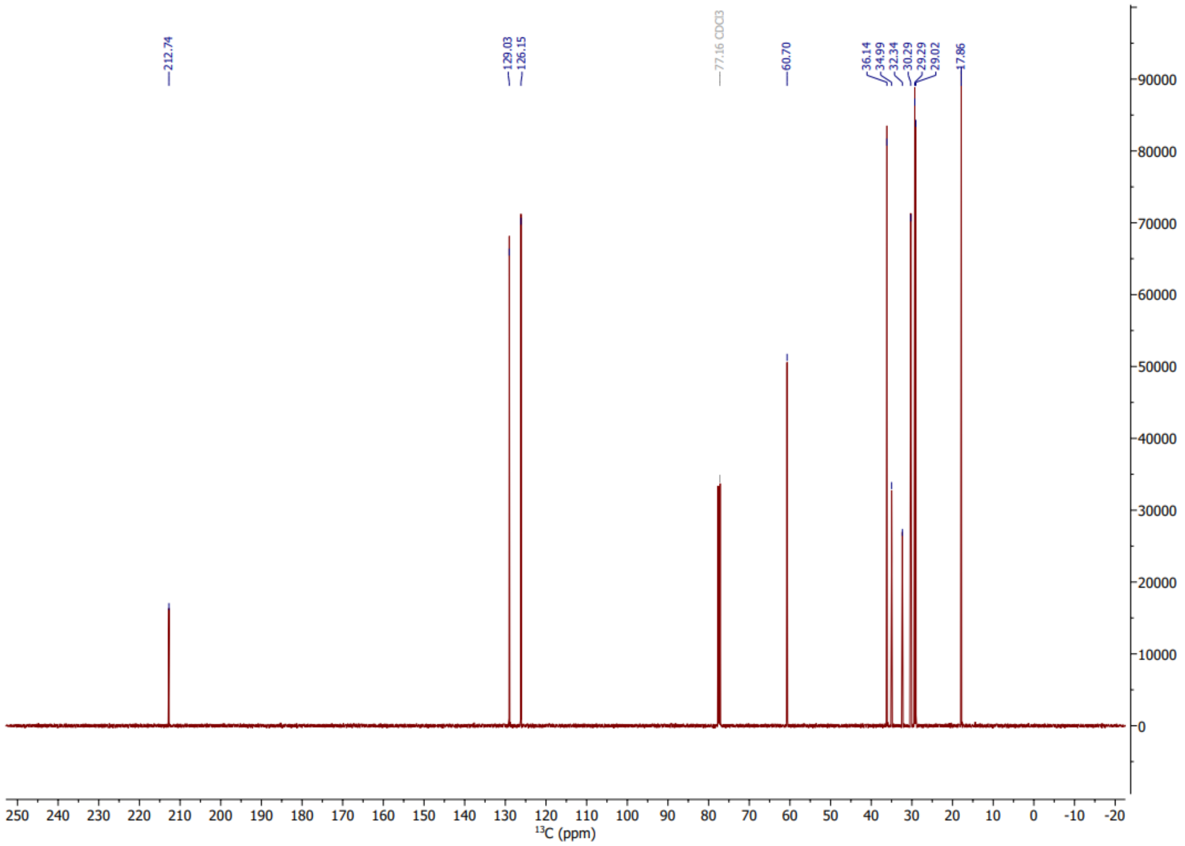

Figure S65. ^{1}H NMR and ^{13}C NMR spectra of compound **4w**.

18.4. Derivation products

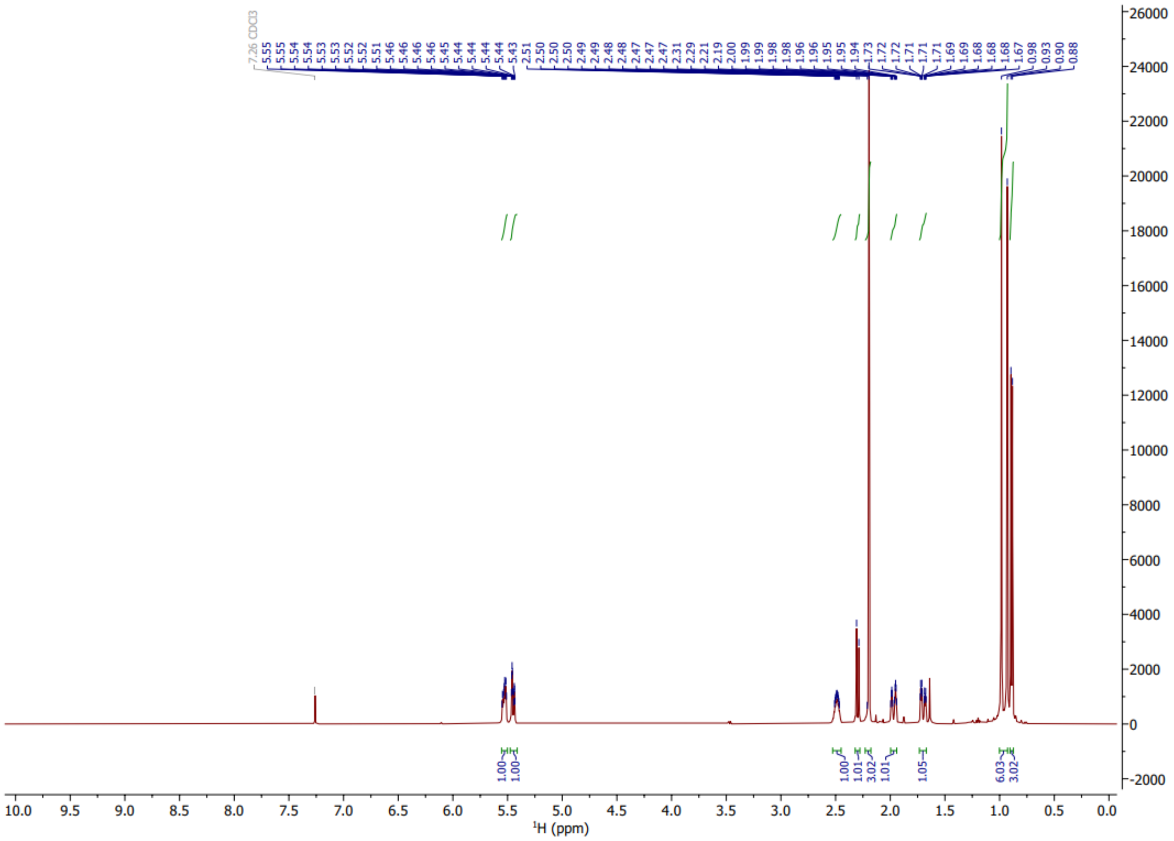

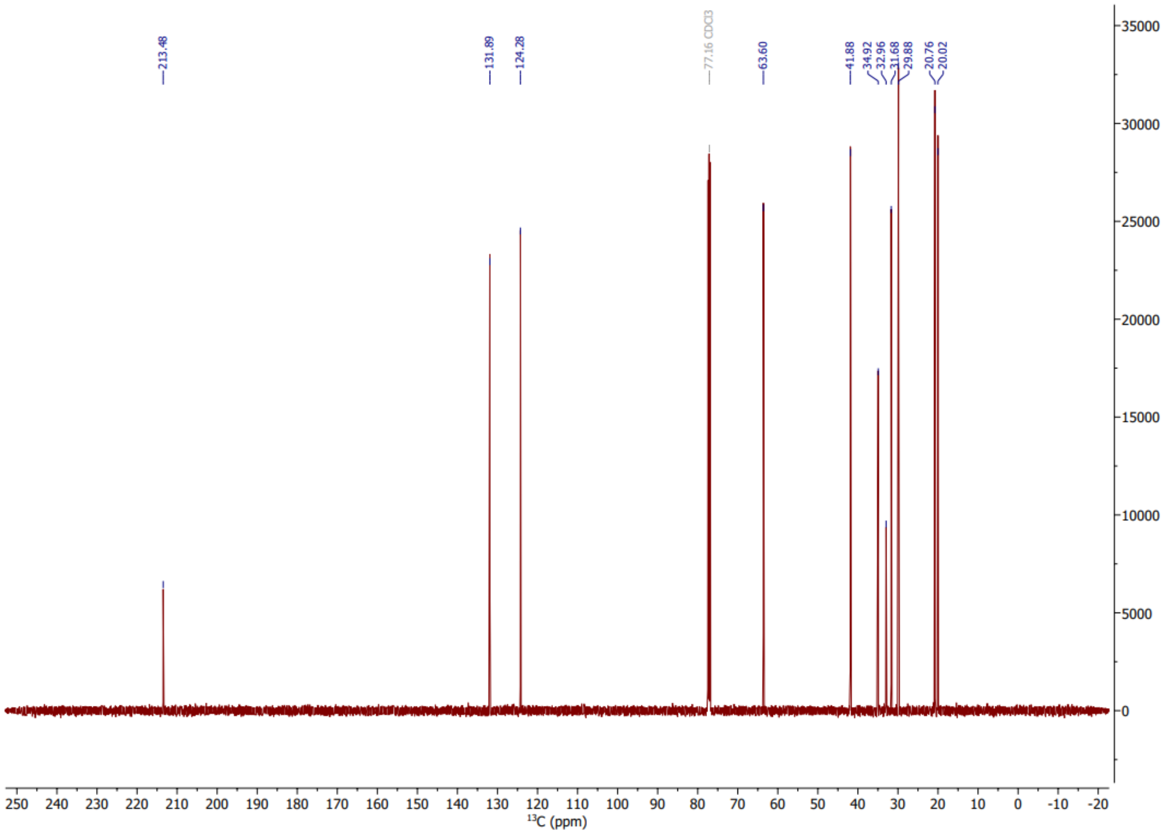

Figure S65. ^{1}H NMR and ^{13}C NMR spectra of compound ***trans*-4w**.

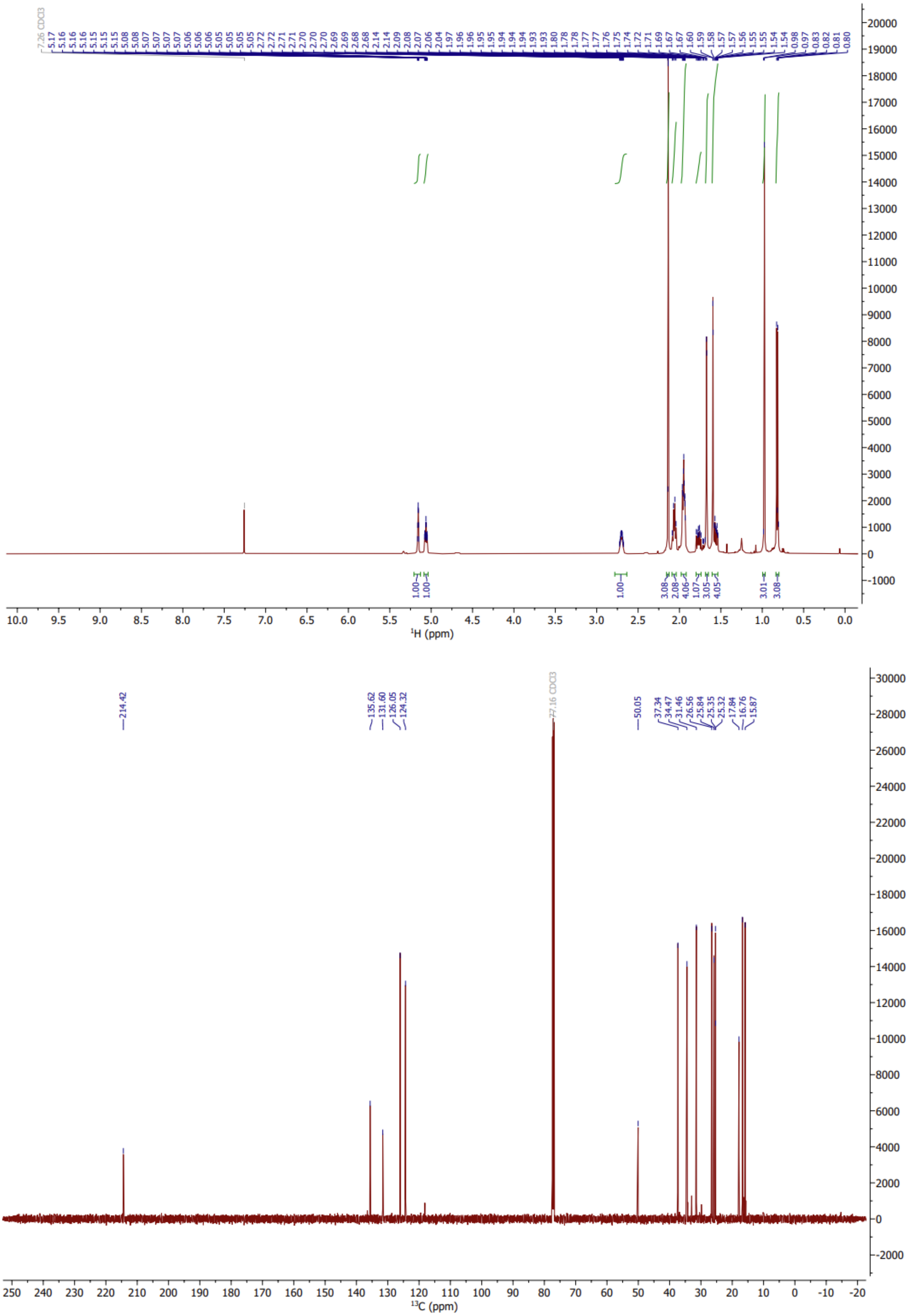

Figure S66. ^{1}H NMR and ^{13}C NMR spectra of compound **6a**.

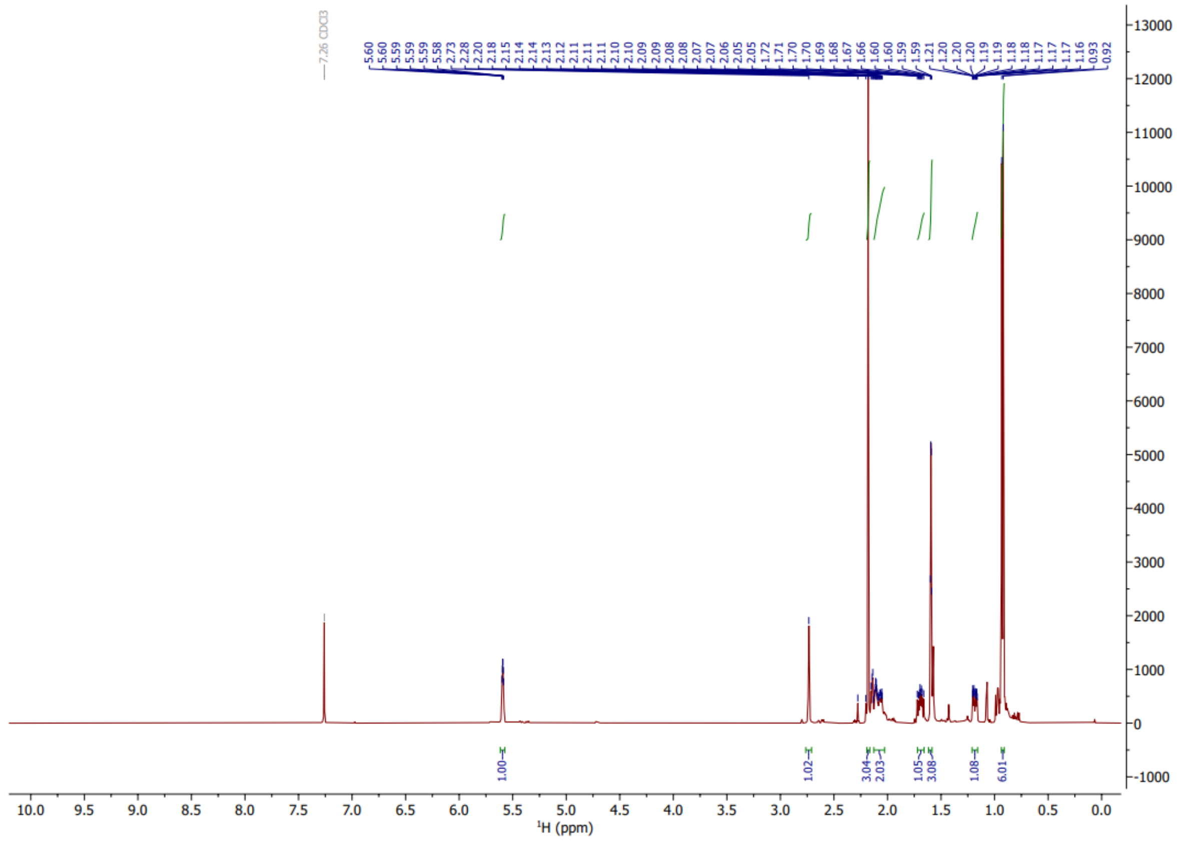

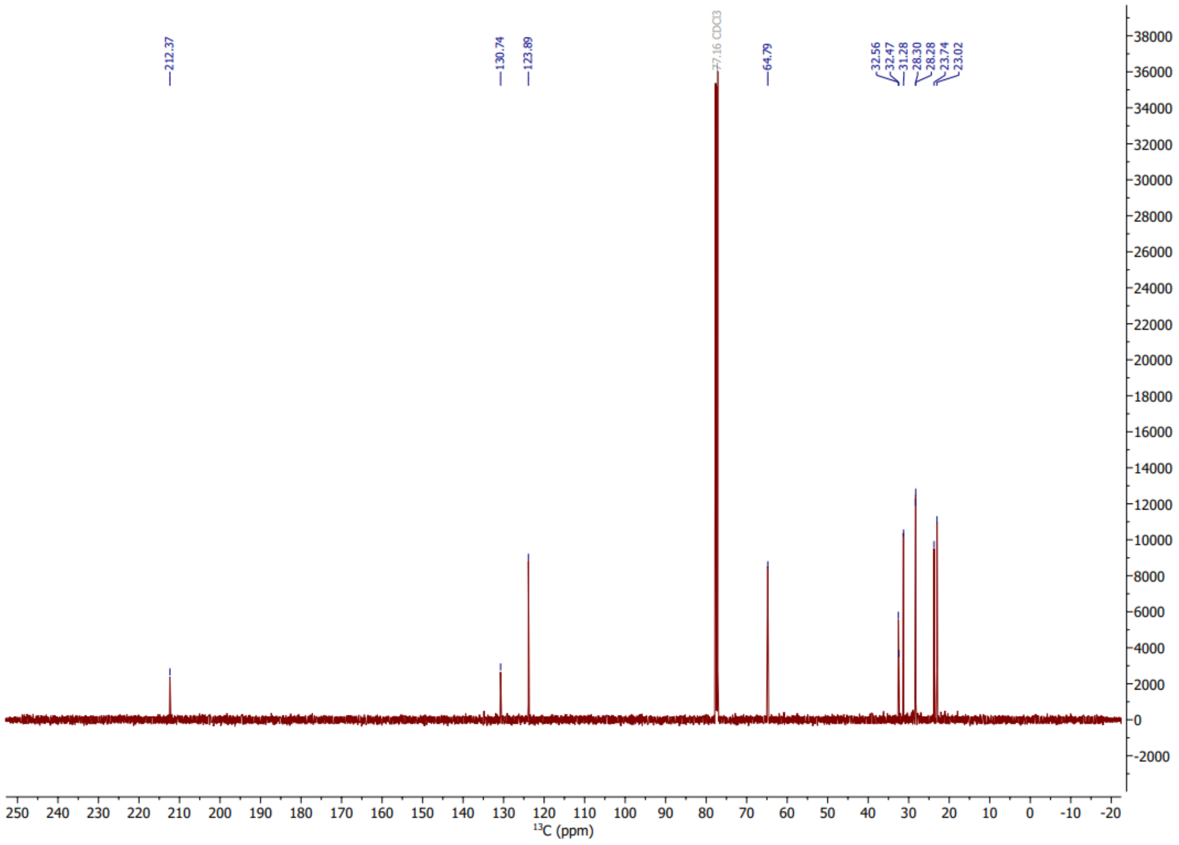

Figure S67. ^{1}H NMR and ^{13}C NMR spectra of compound **6w**.

18.5. Synthetic Targets

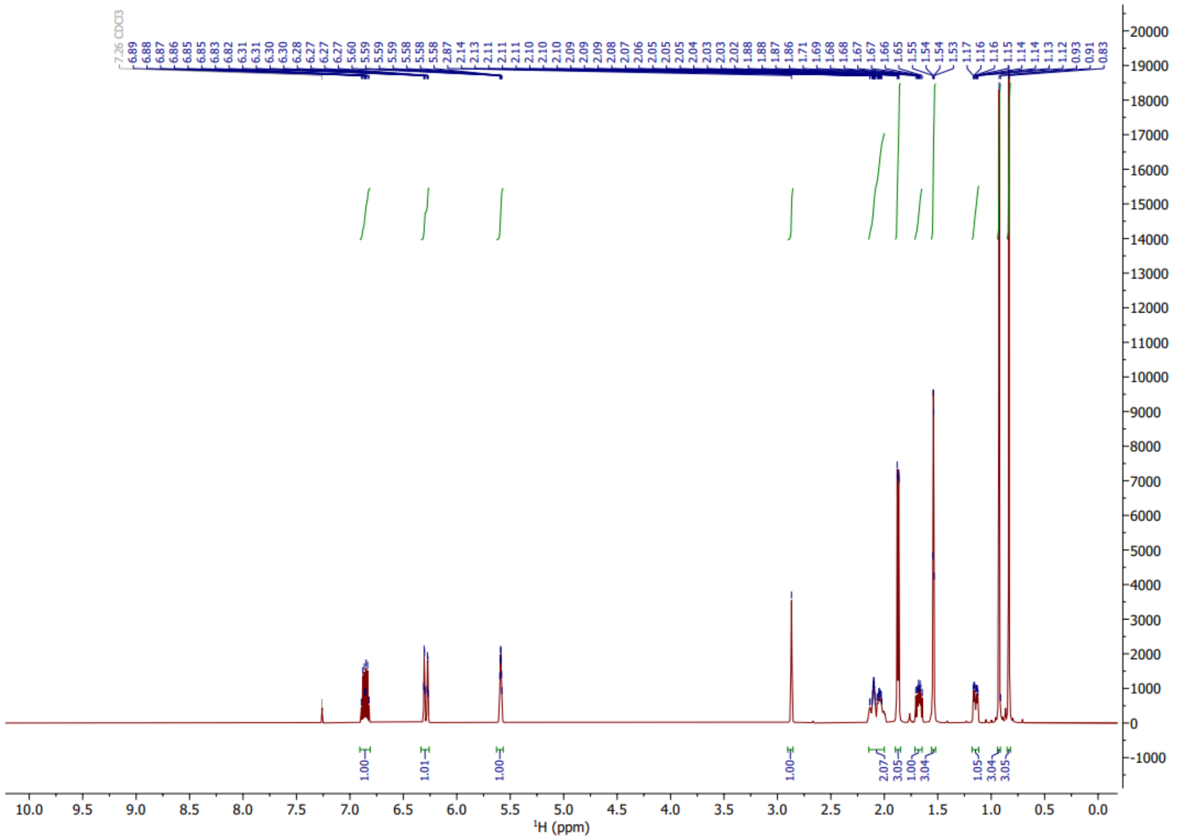

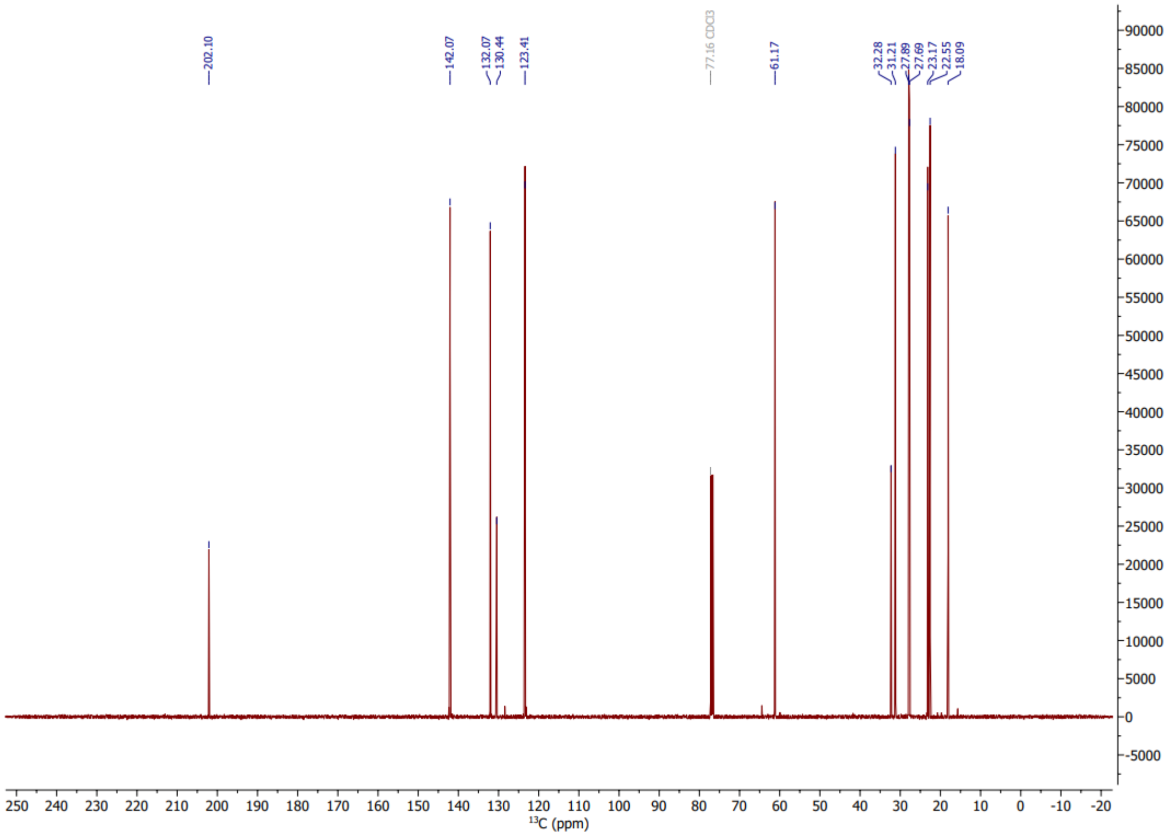

Figure S68. ^{1}H NMR and ^{13}C NMR spectra of compound **1a**.

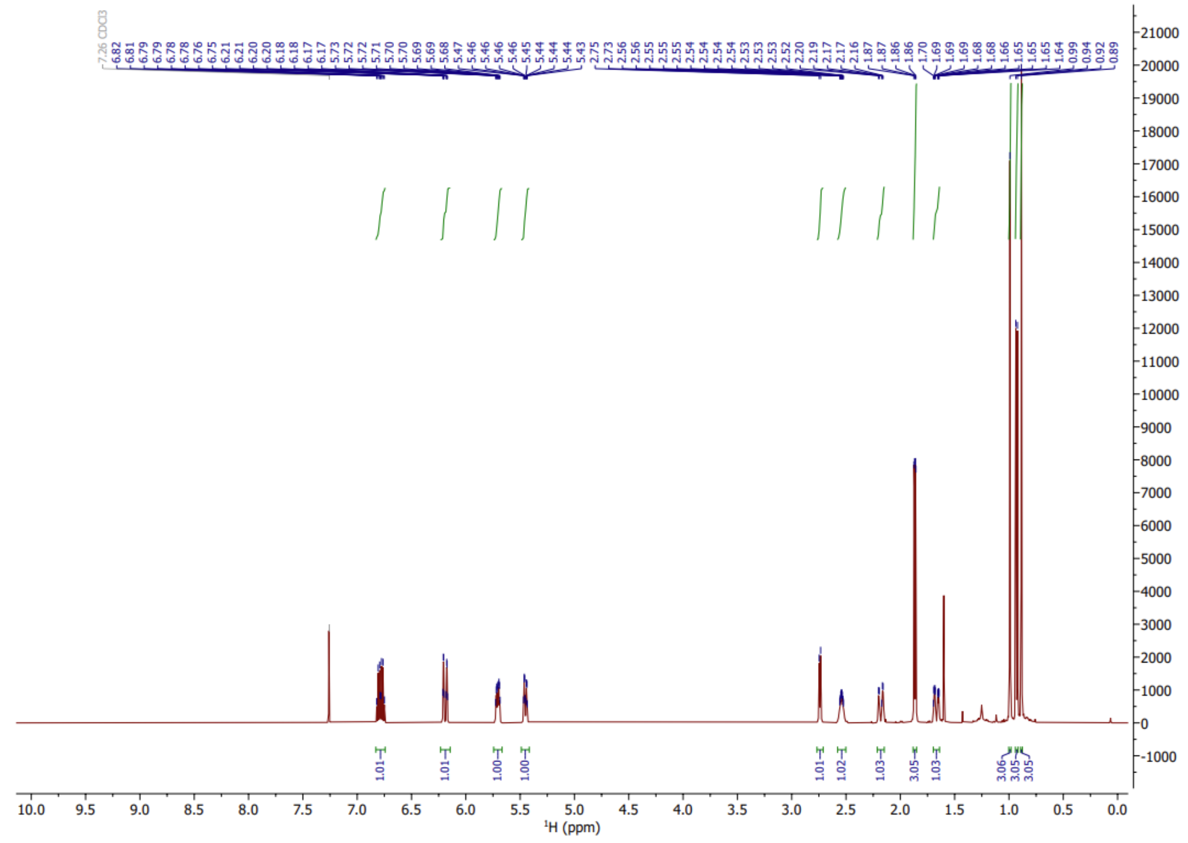

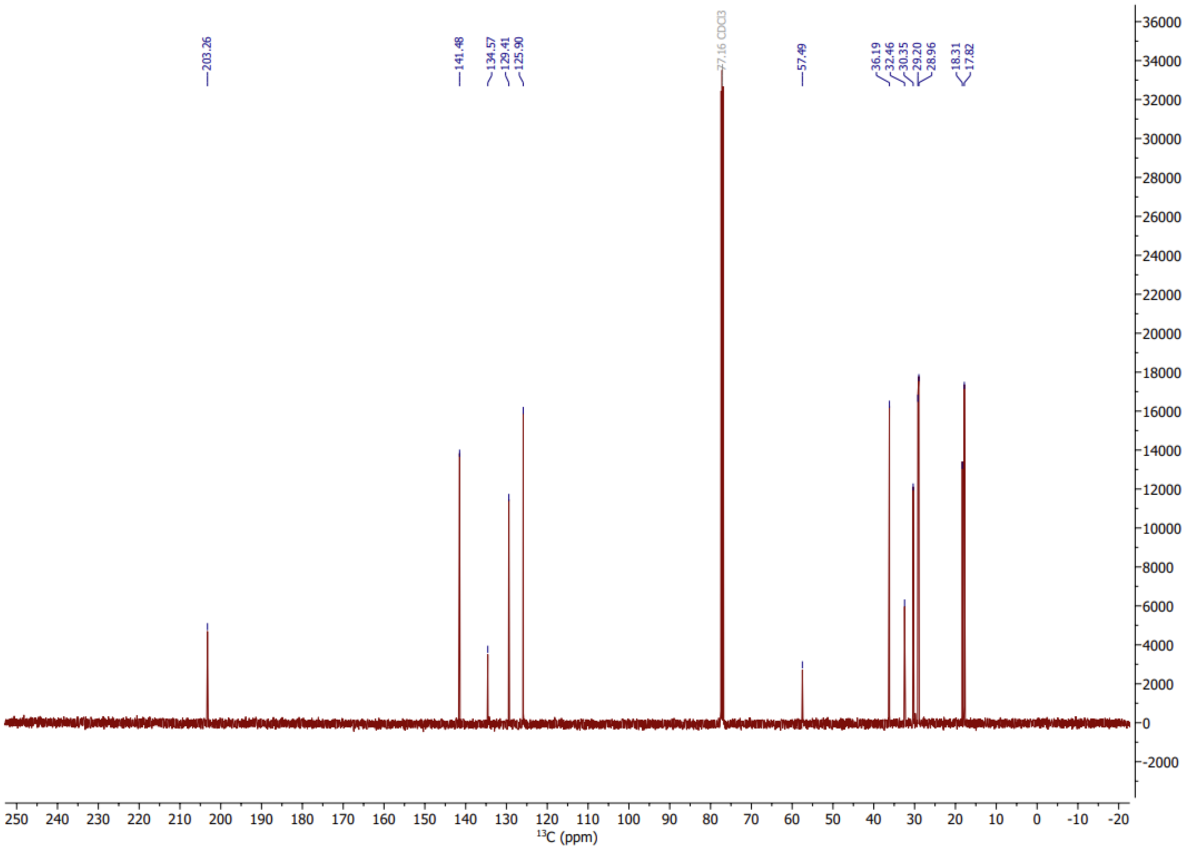

Figure S69. ^{1}H NMR and ^{13}C NMR spectra of compound ***cis*-1b**.

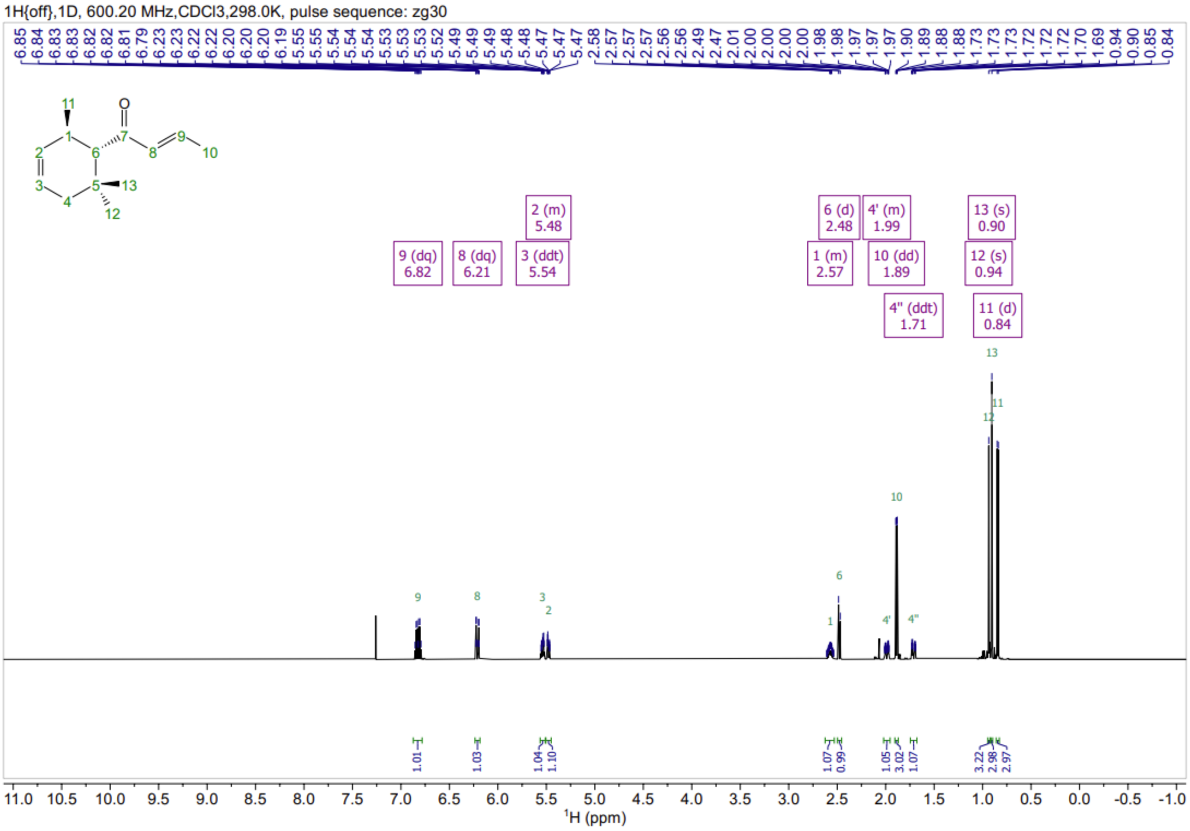

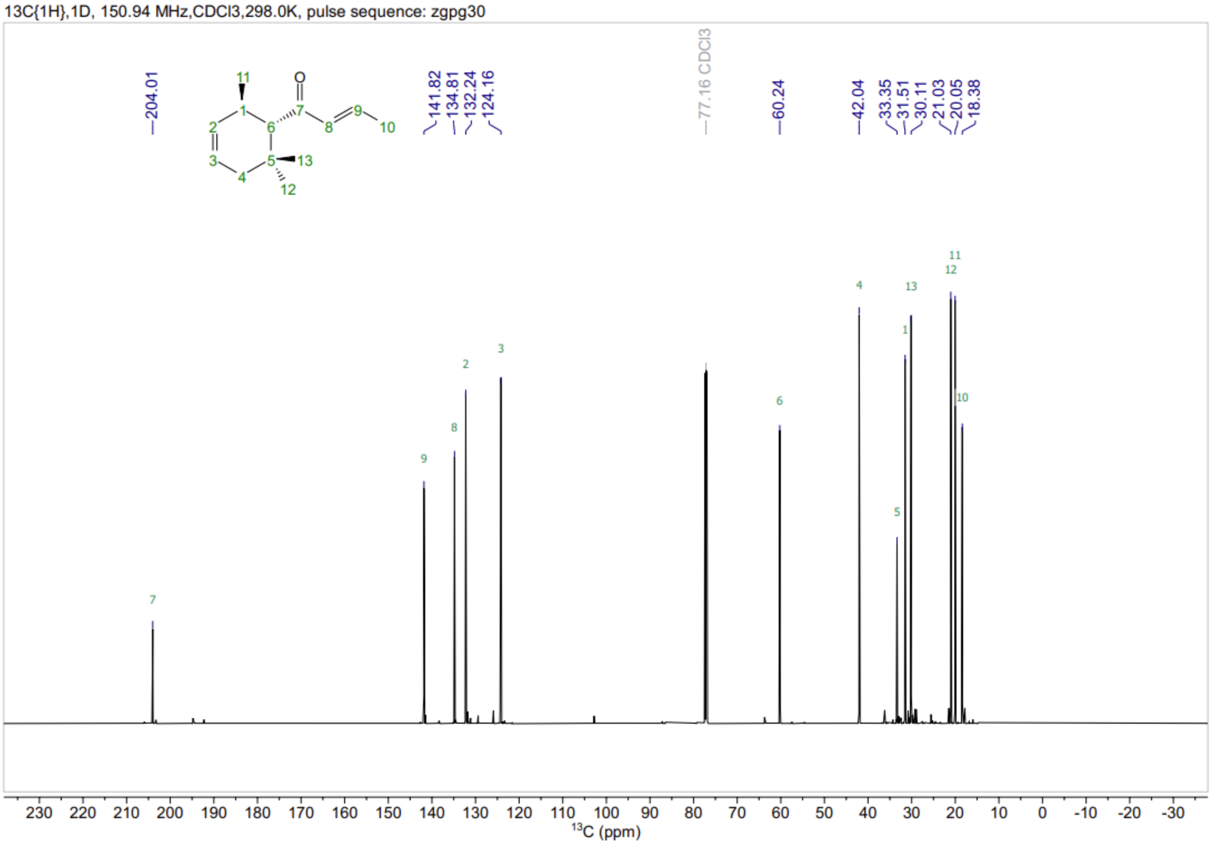

Figure S70. ^{1}H NMR and ^{13}C NMR spectra of compound ***trans*-1b**.

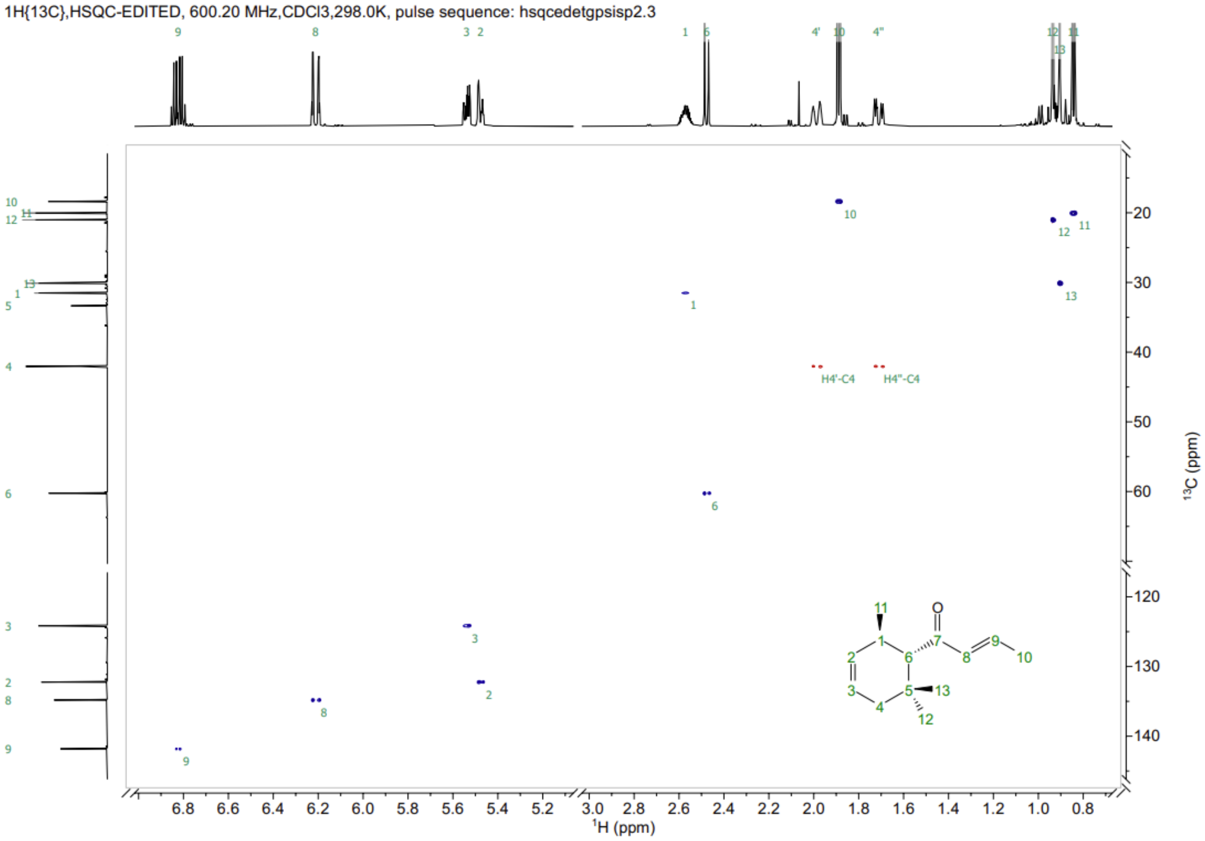

Figure S71. HSQC spectrum of compound ***trans*-1b**.

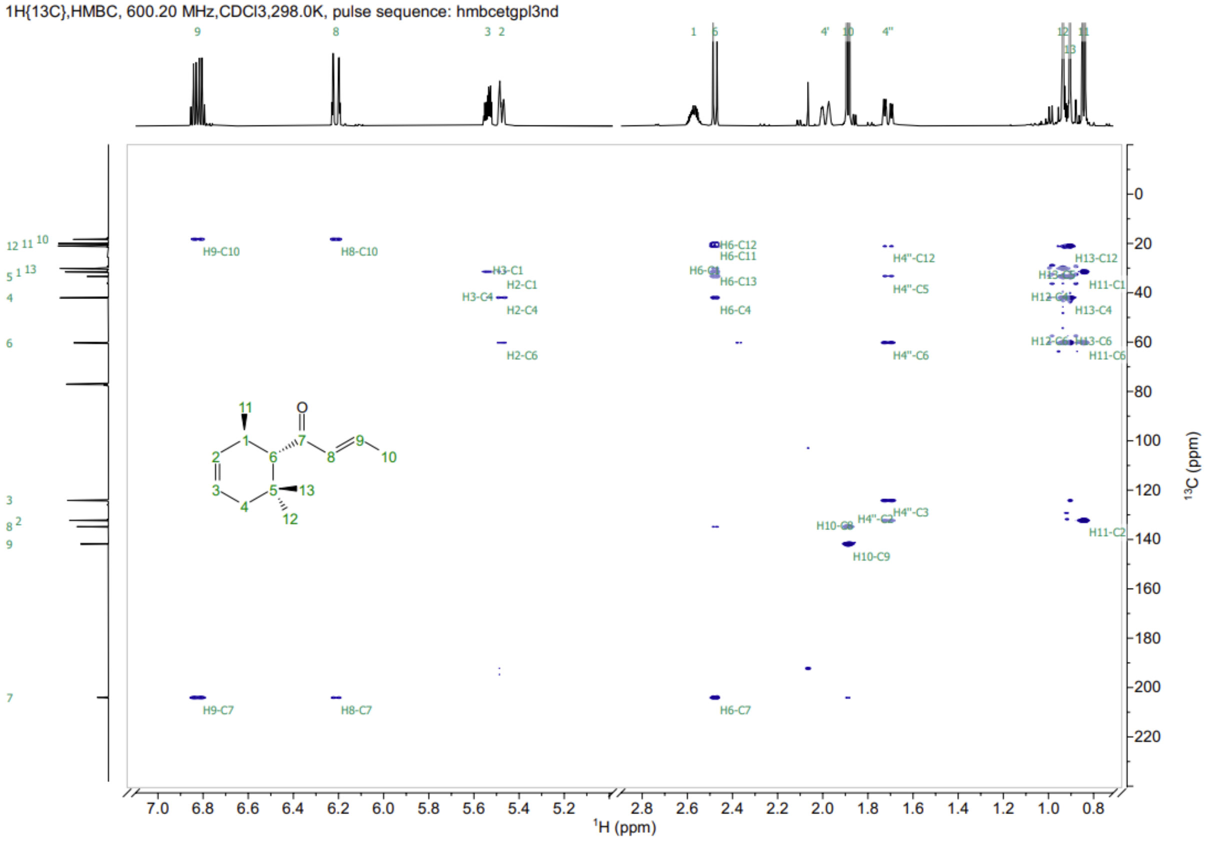

Figure S72. HMBC spectrum of compound ***trans*-1b**.

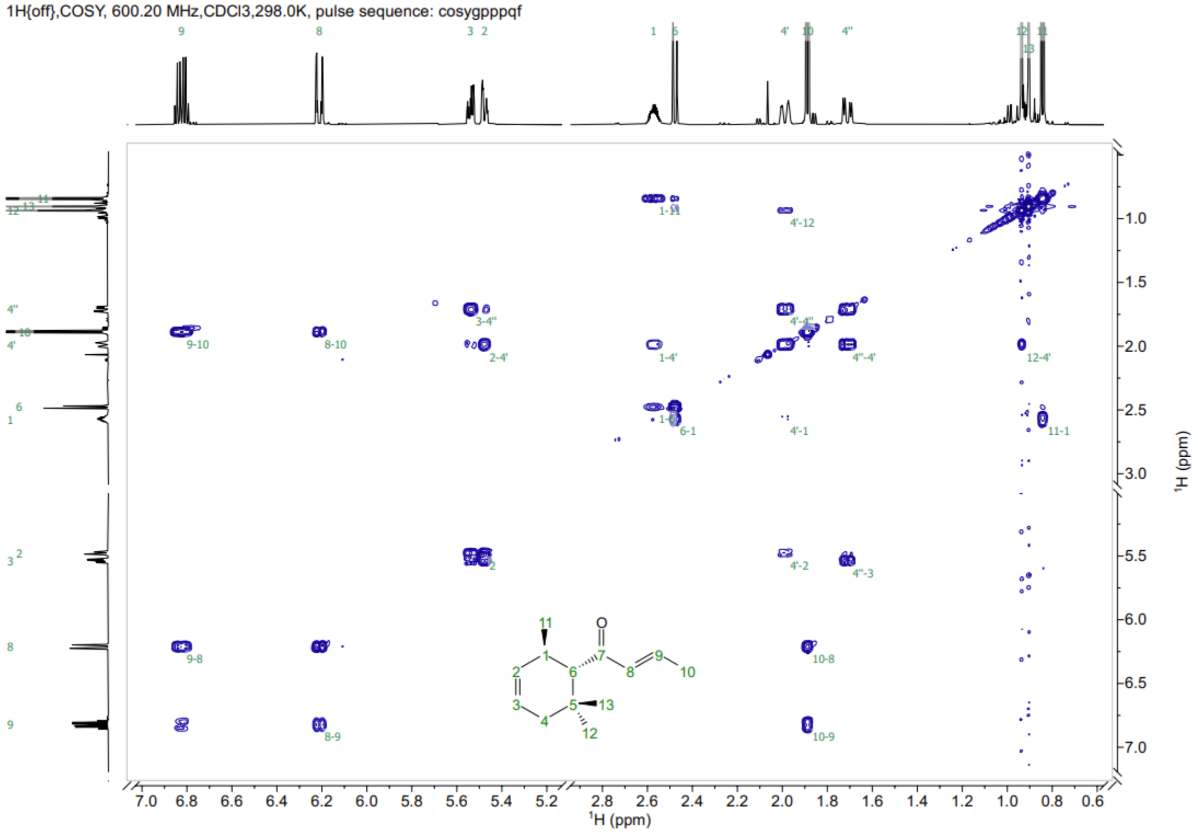

Figure S73. COSY spectrum of compound ***trans*-1b**.

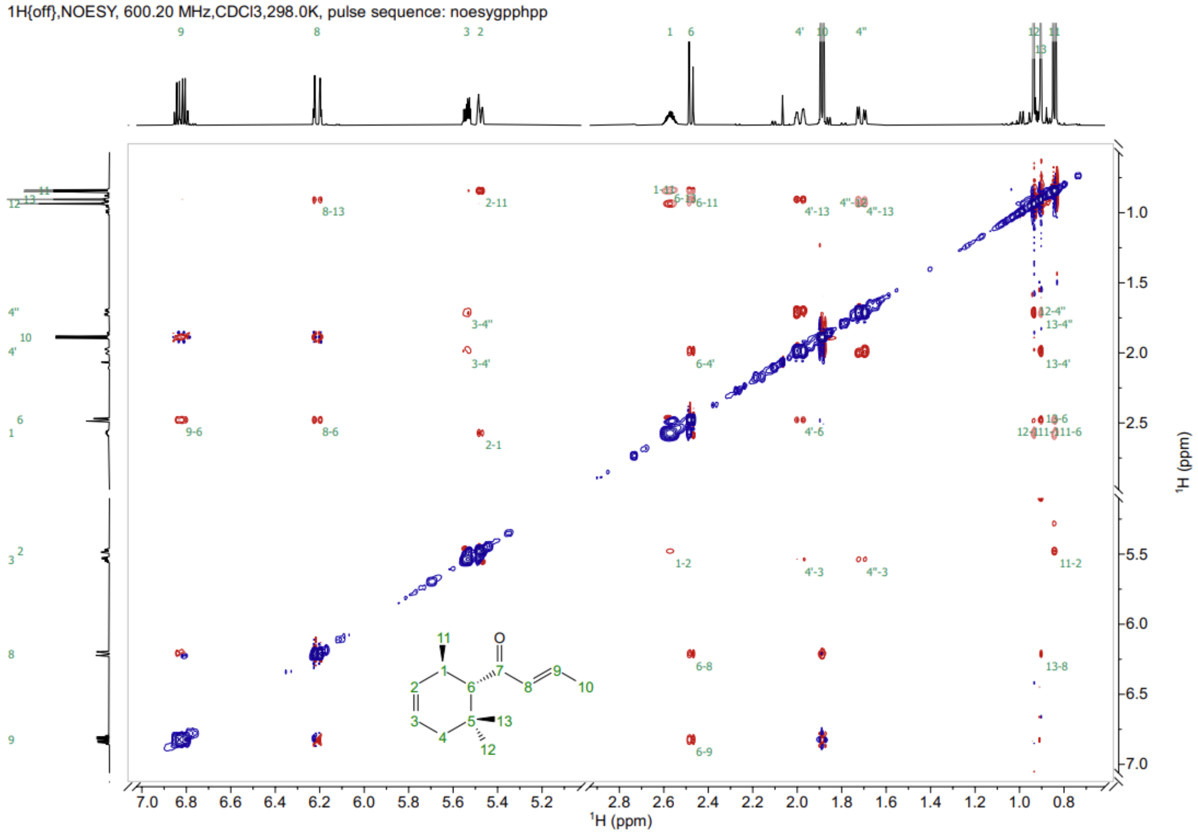

Figure S74. NOESY spectra of compound ***trans*-1b**.

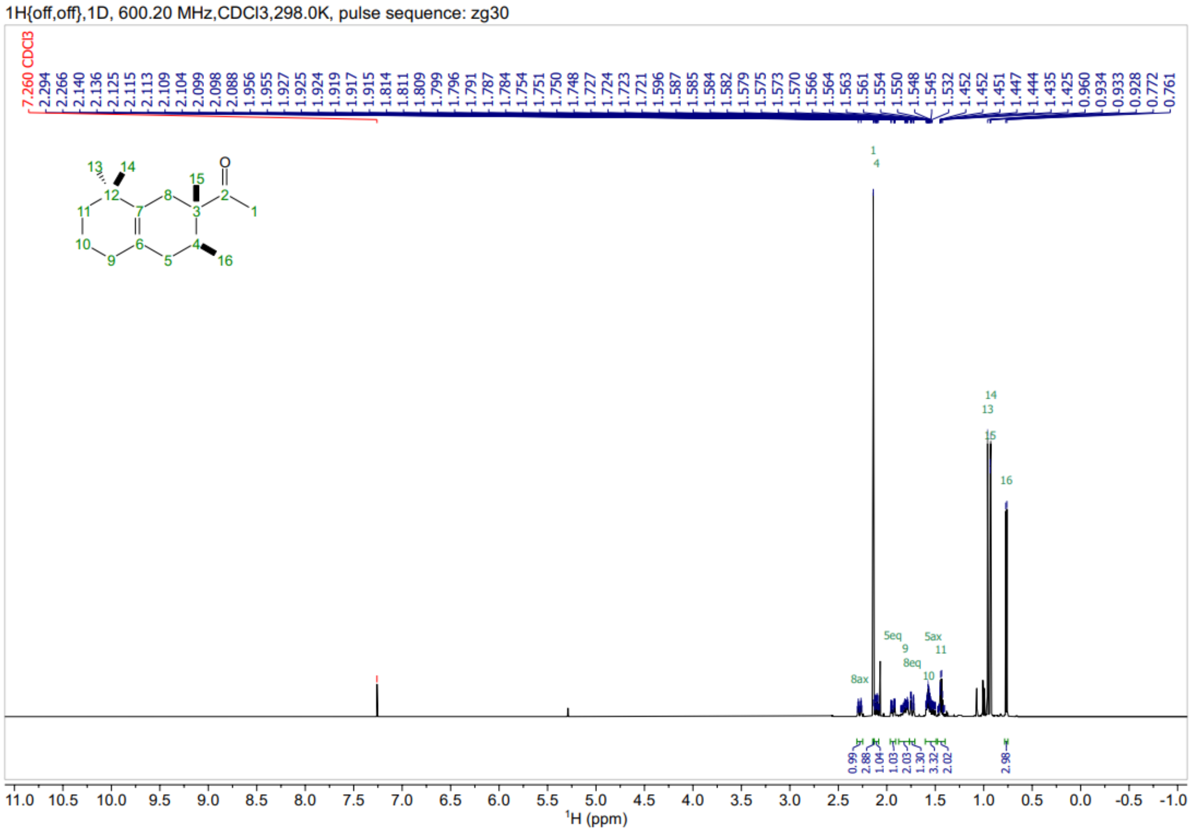

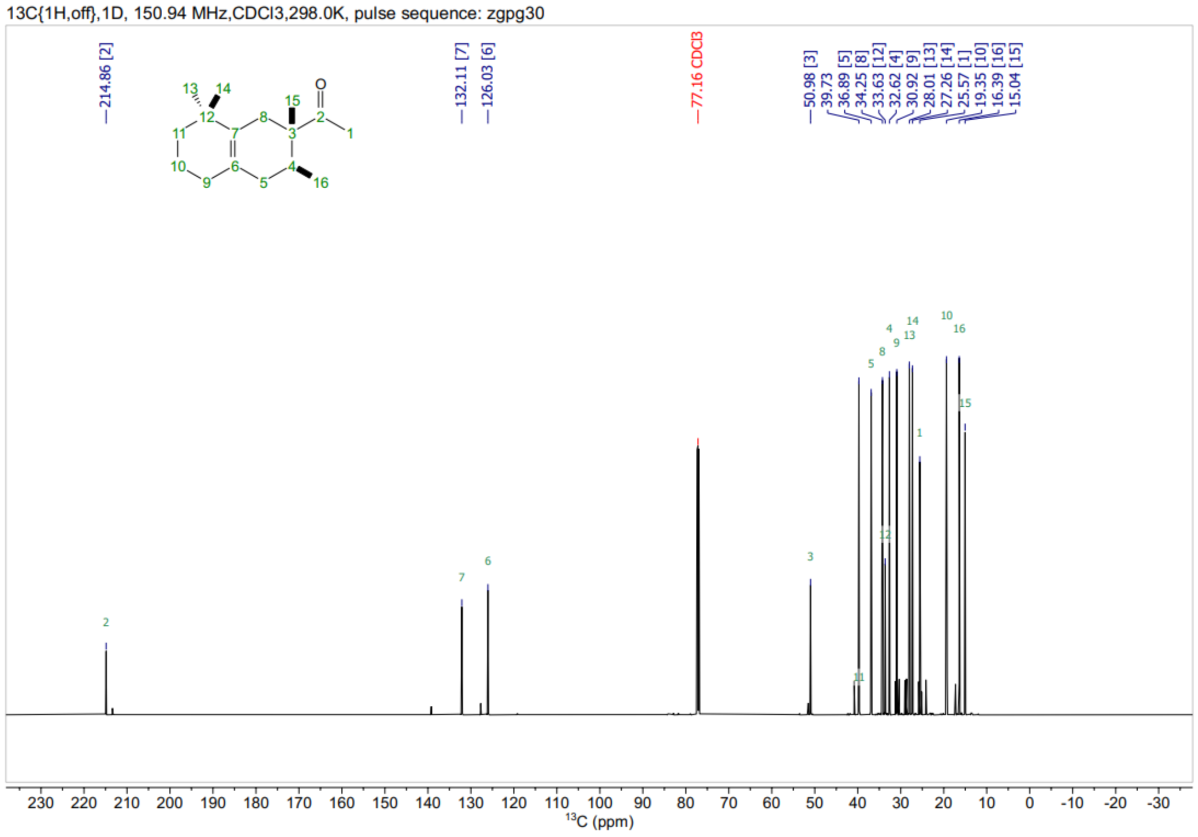

Figure S75. ^{1}H NMR and ^{13}C NMR spectra of compound **1c**.

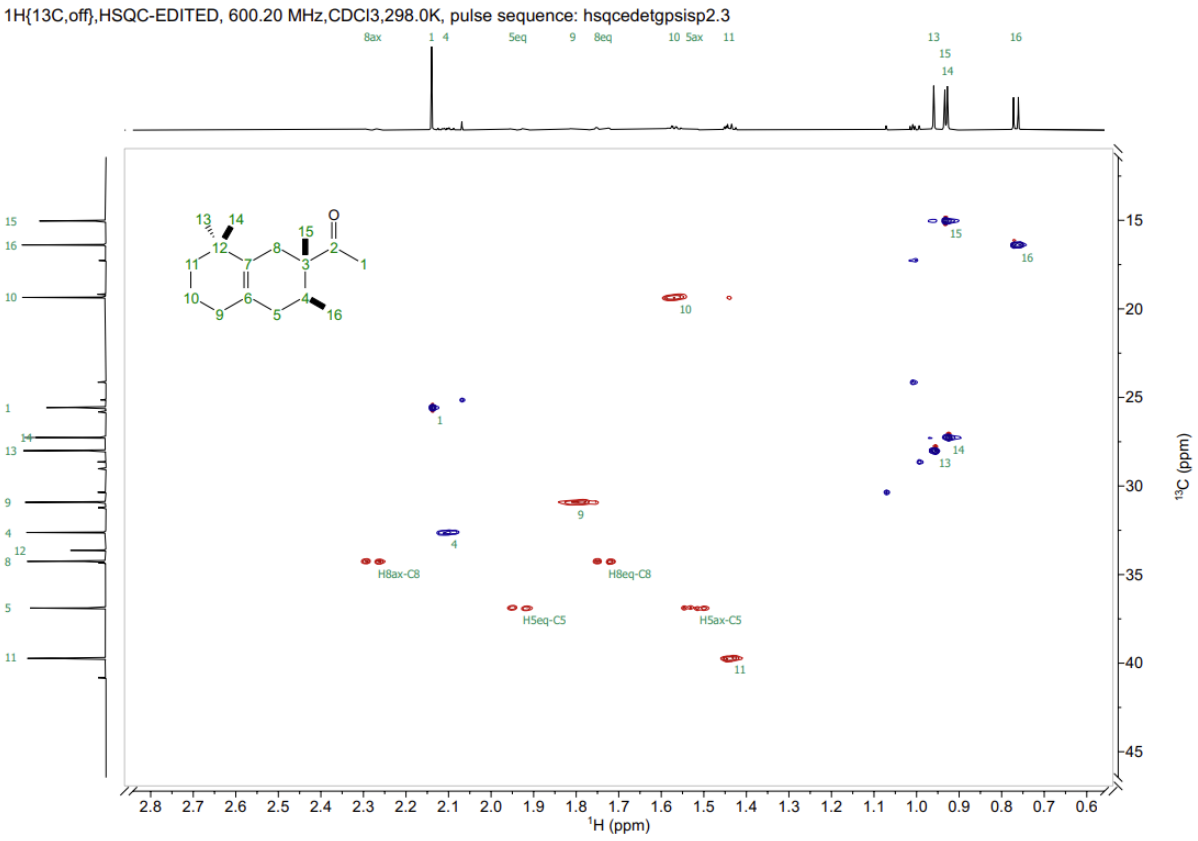

Figure S76. HSQC spectrum of compound **1c**.

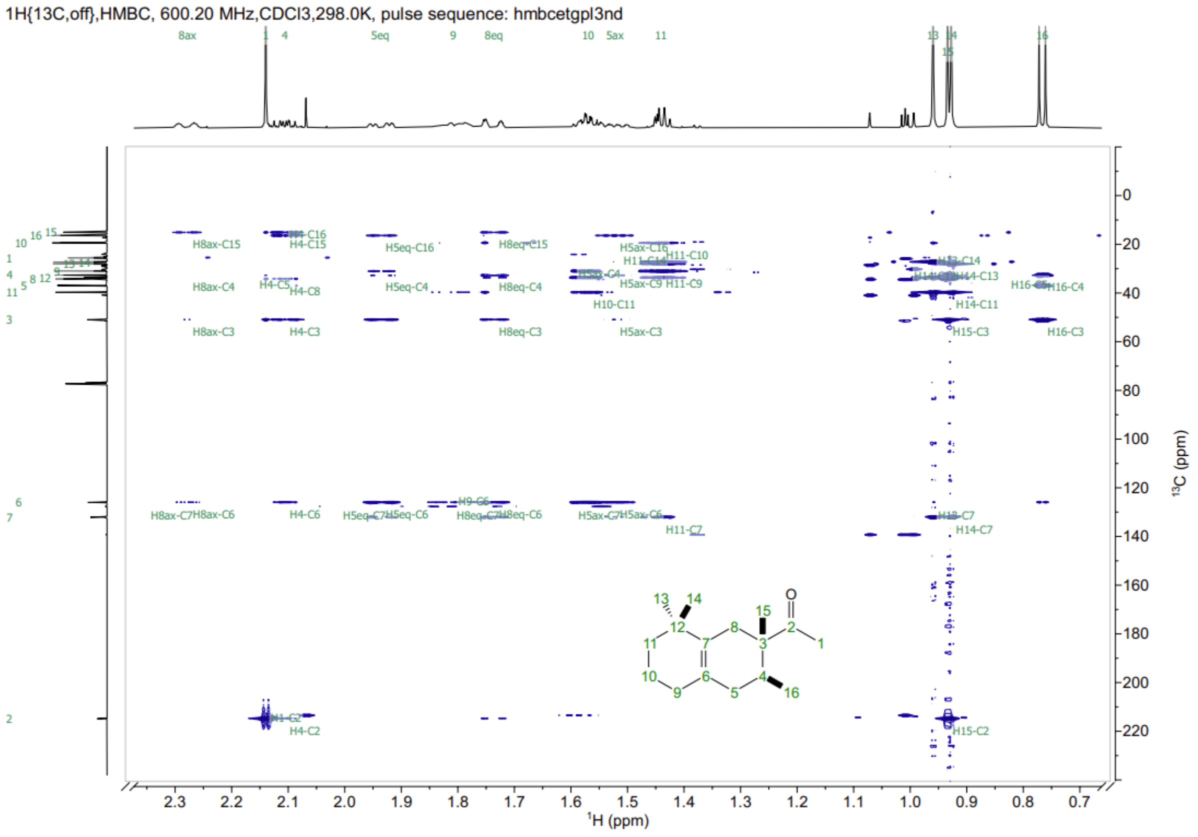

Figure S77. HMBC spectrum of compound **1c**.

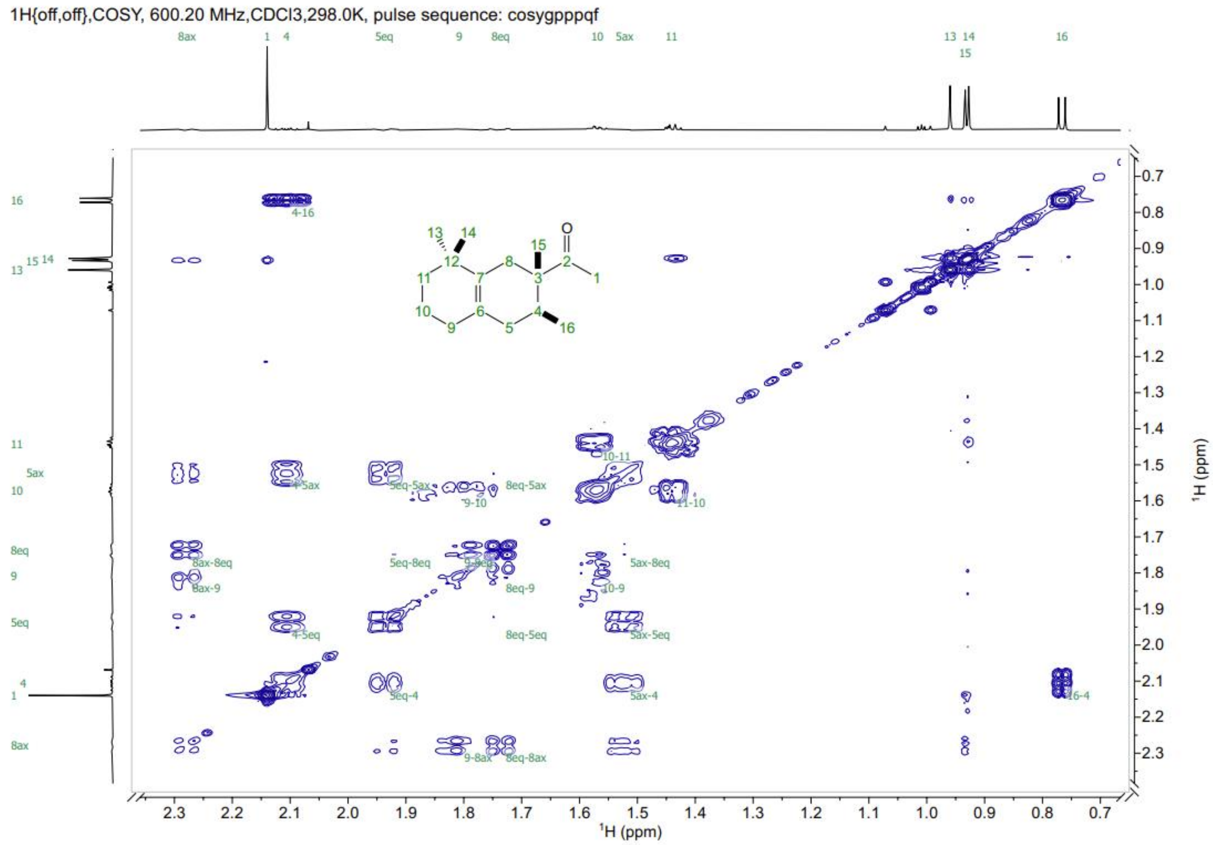

Figure S78. COSY spectrum of compound **1c**.

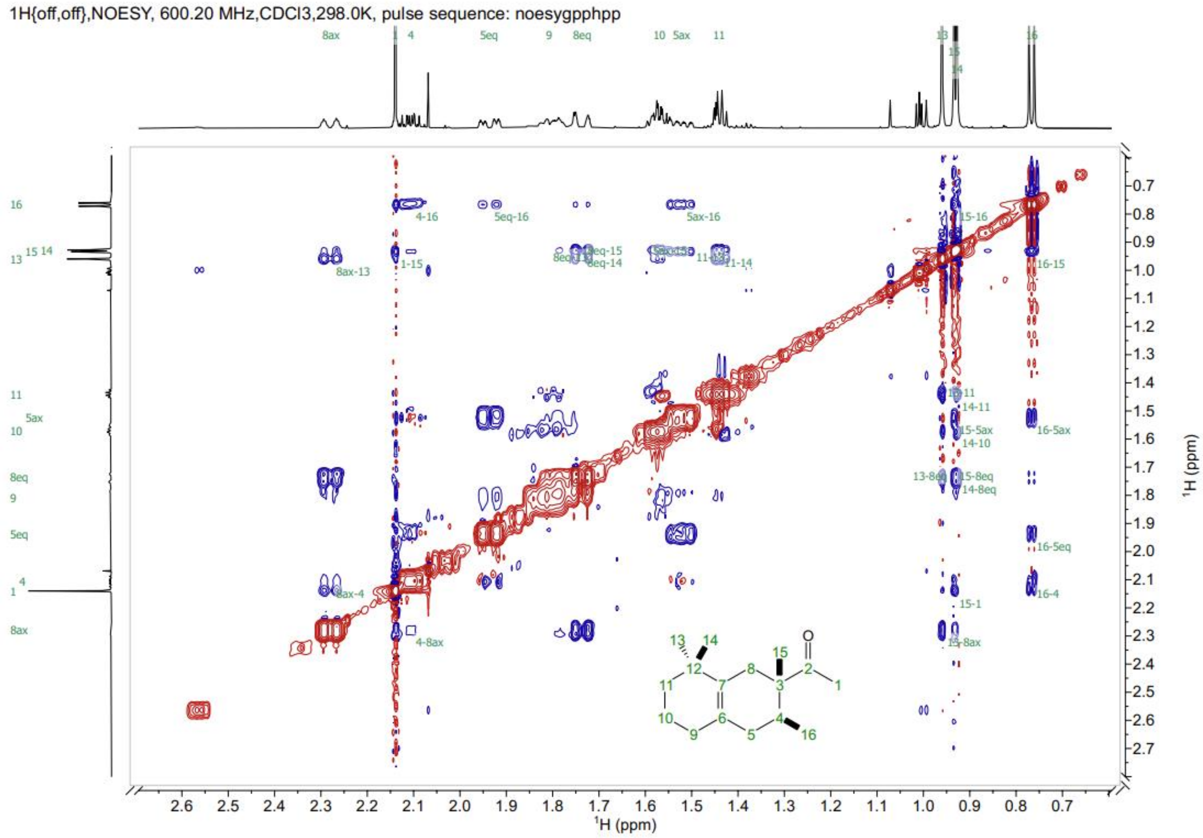

Figure S79. NOESY spectra of compound **1c**.

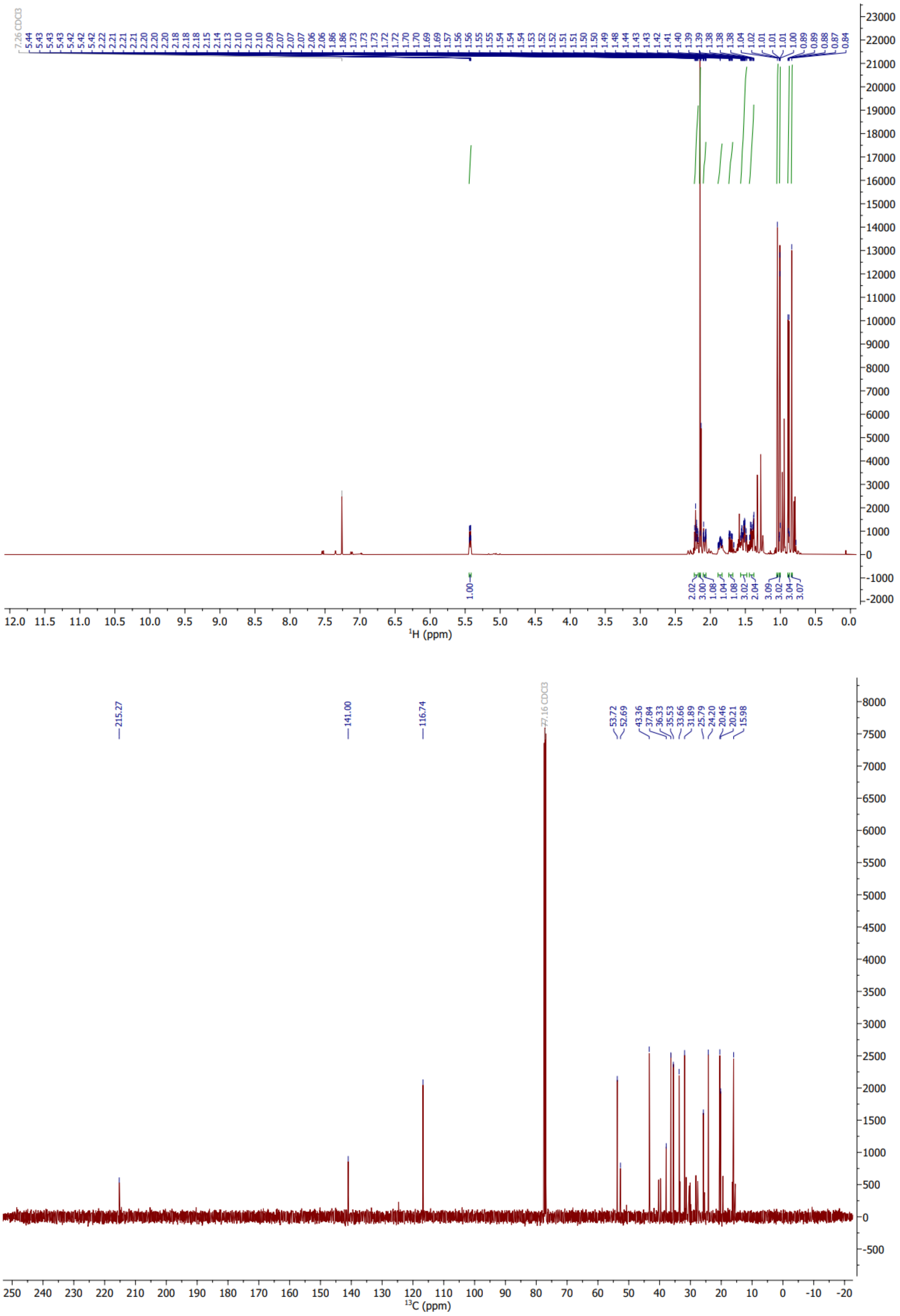

Figure S80. ^{1}H NMR and ^{13}C NMR spectra of compound **1d**.

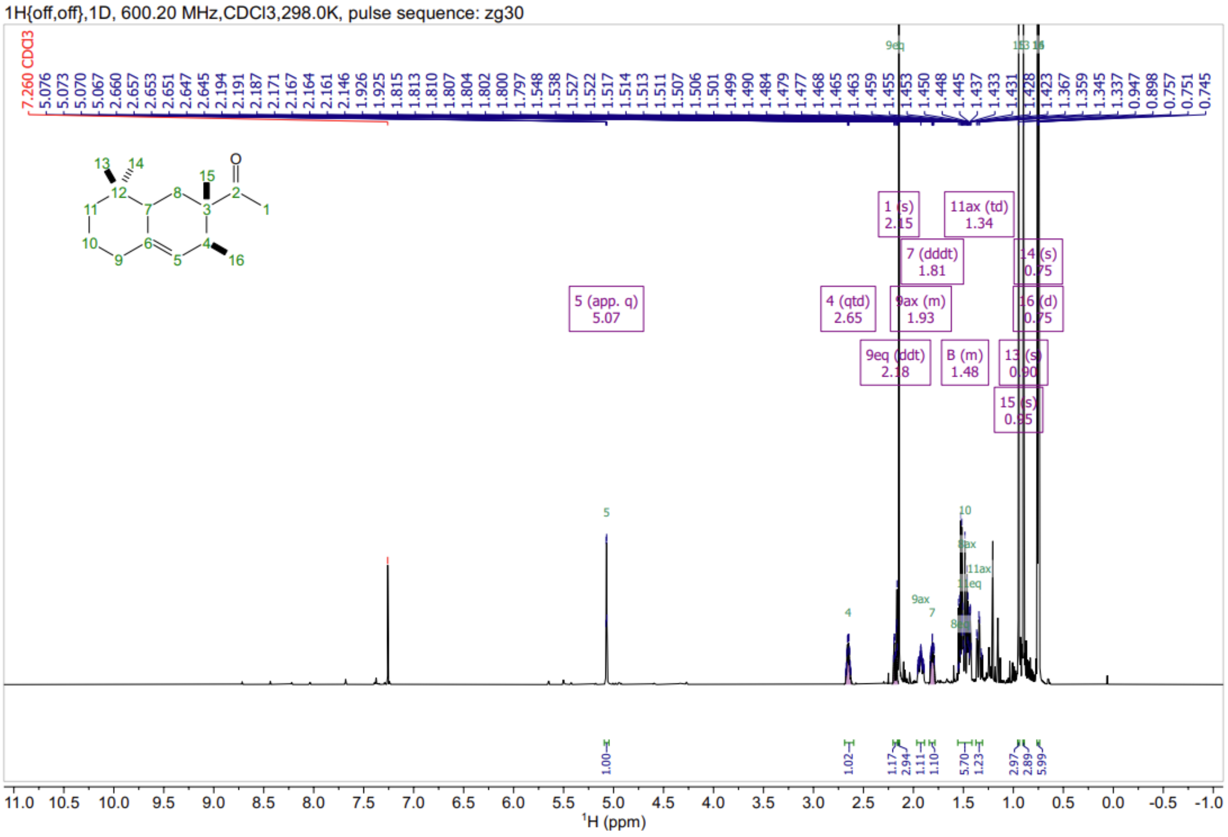

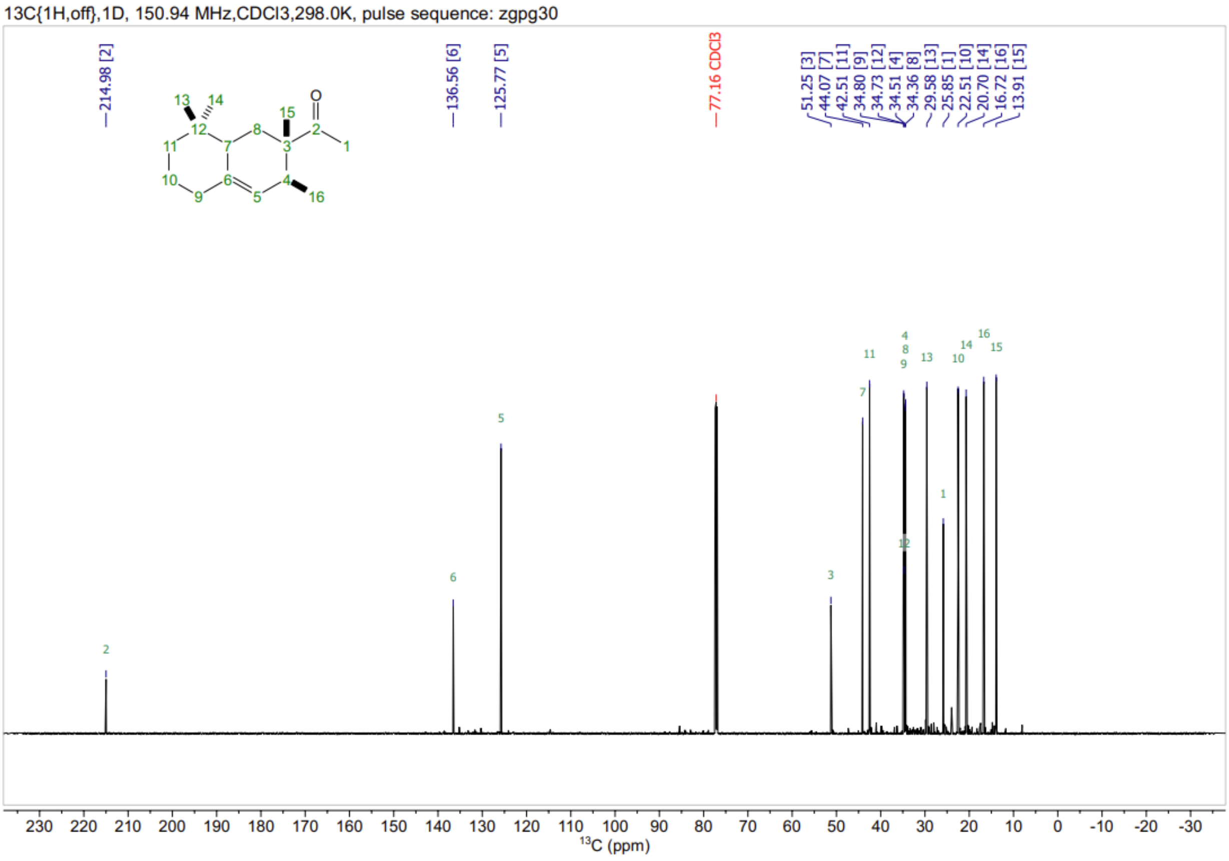

Figure S81. ^{1}H NMR and ^{13}C NMR spectra of compound **1e**.

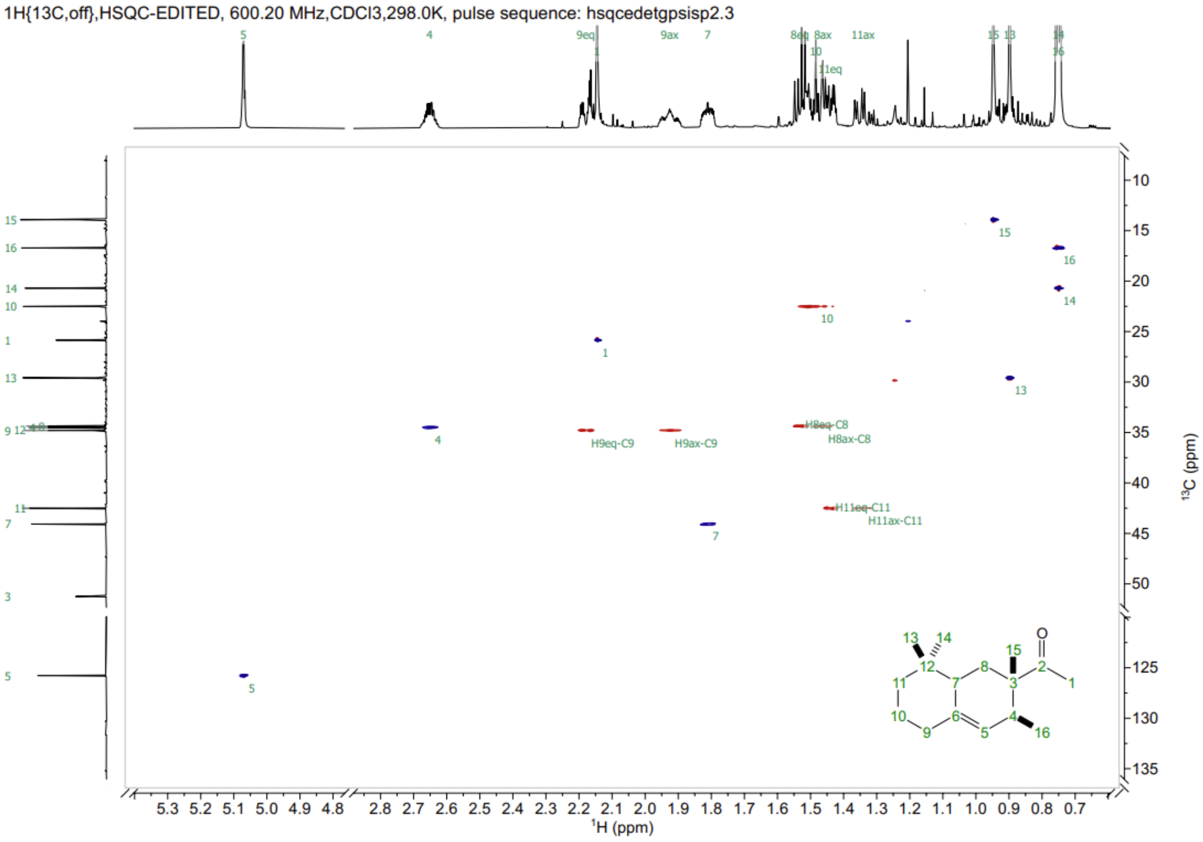

Figure S82. HSQC spectrum of compound **1e**.

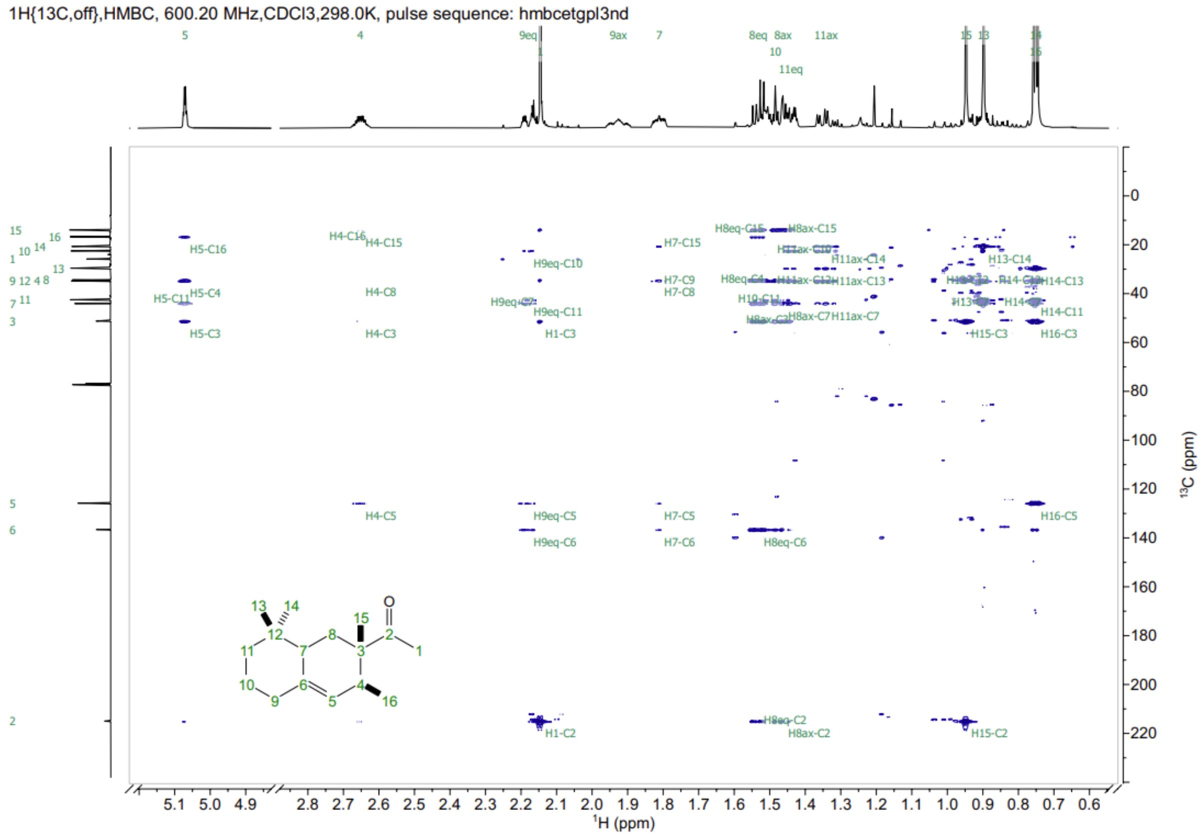

Figure S83. HMBC spectrum of compound **1e**.

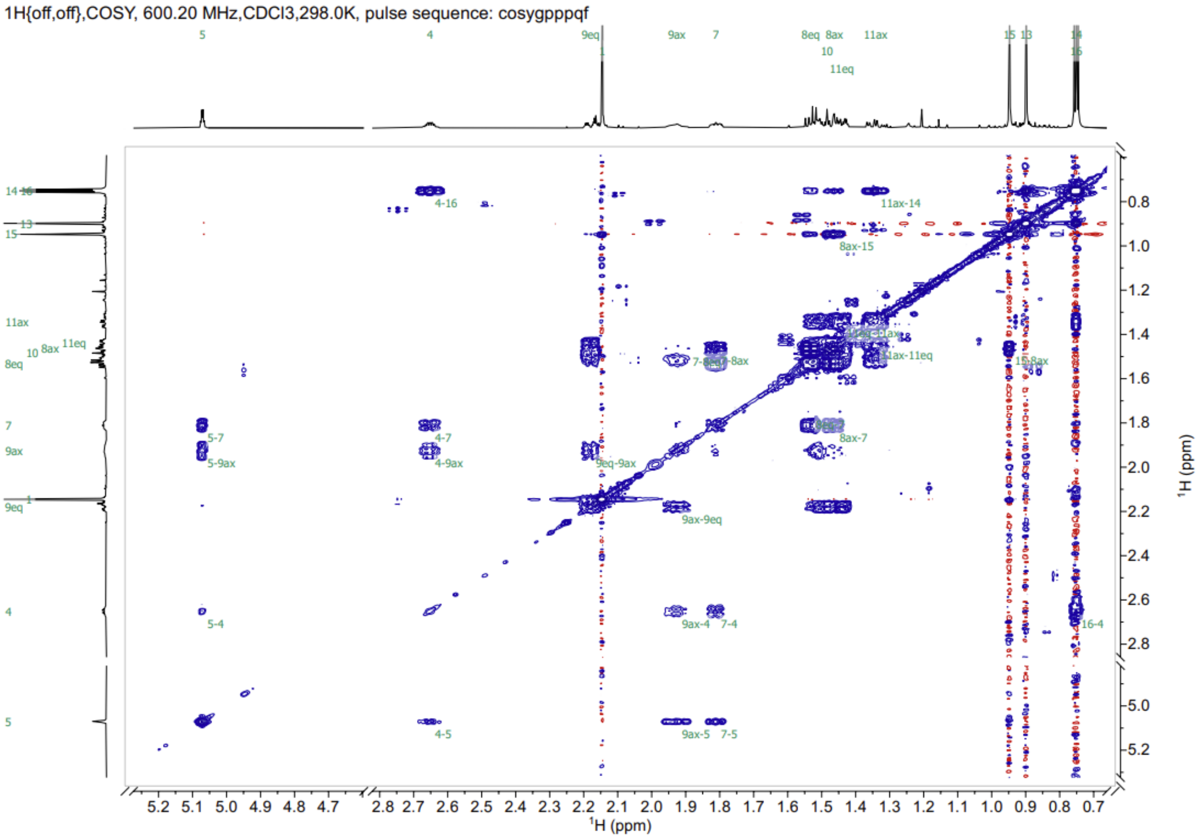

Figure S84. COSY spectrum of compound **1e**.

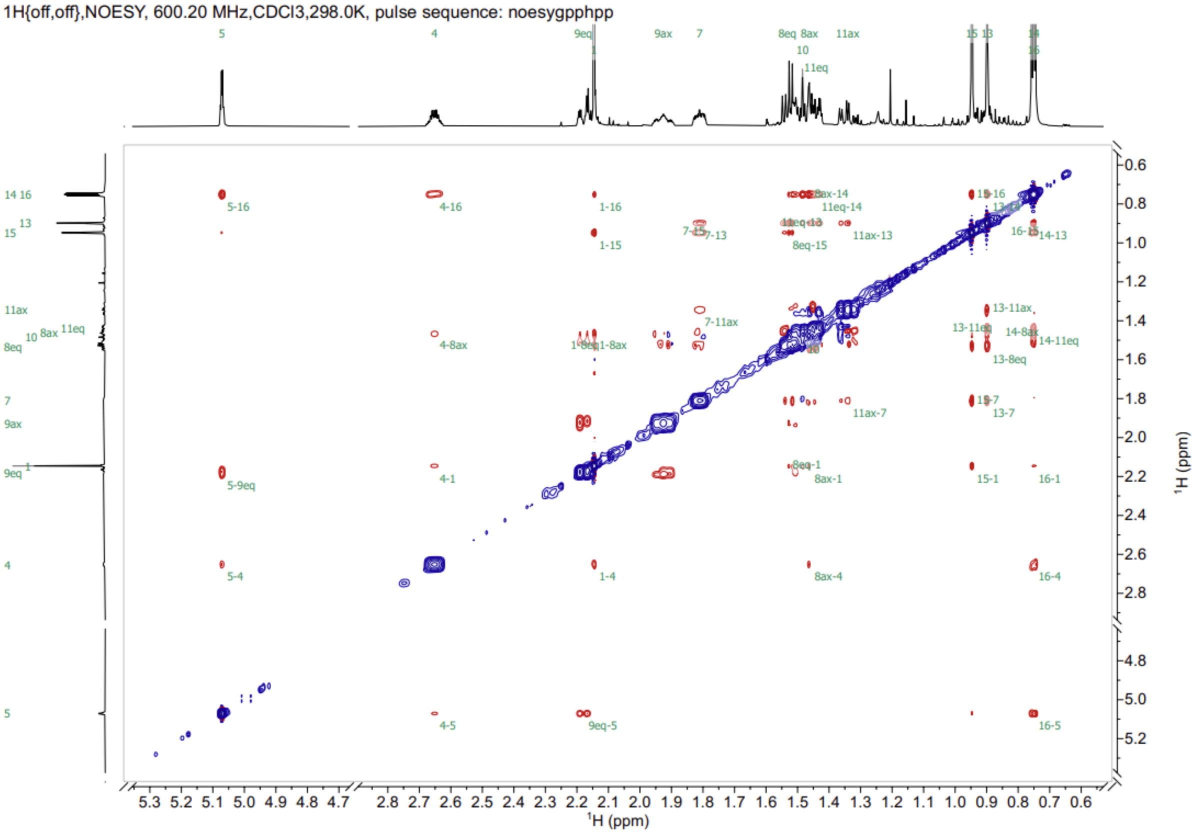

Figure S85. NOESY spectra of compound **1e**.

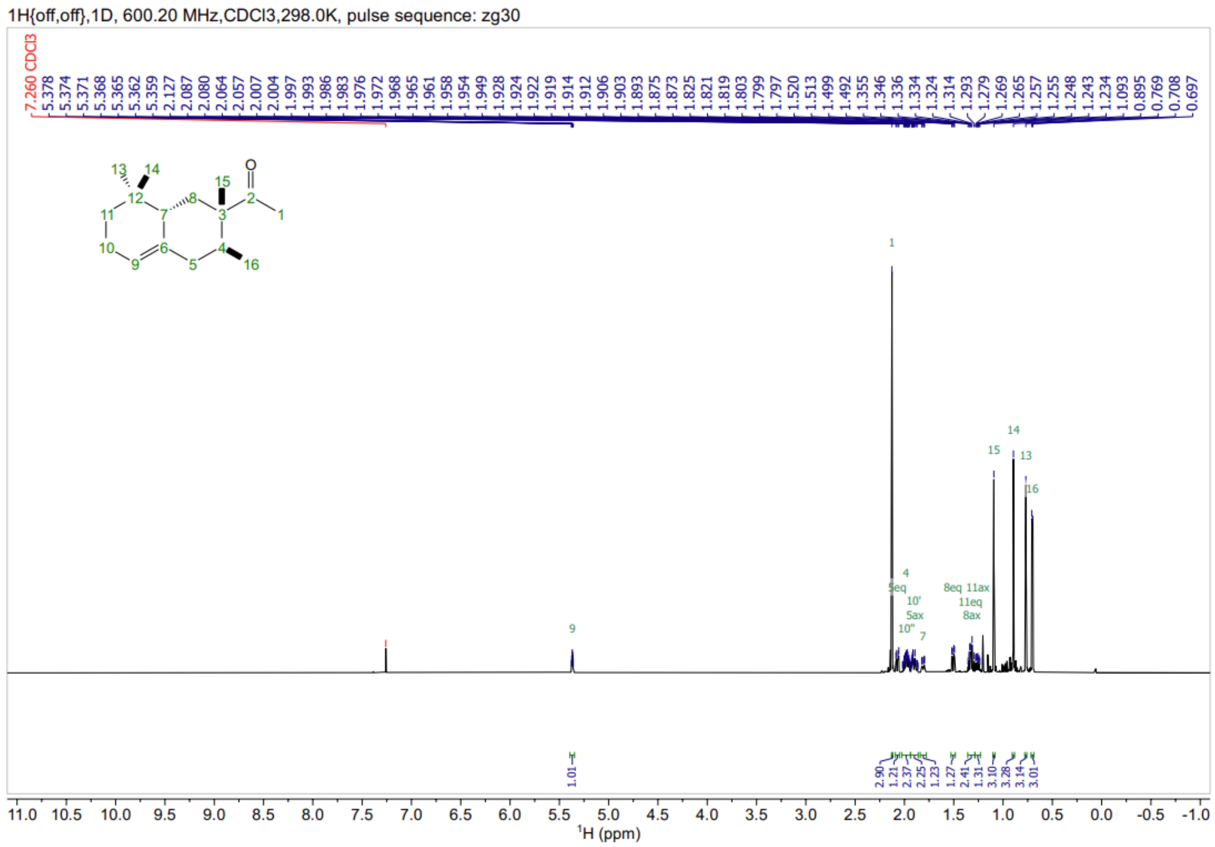

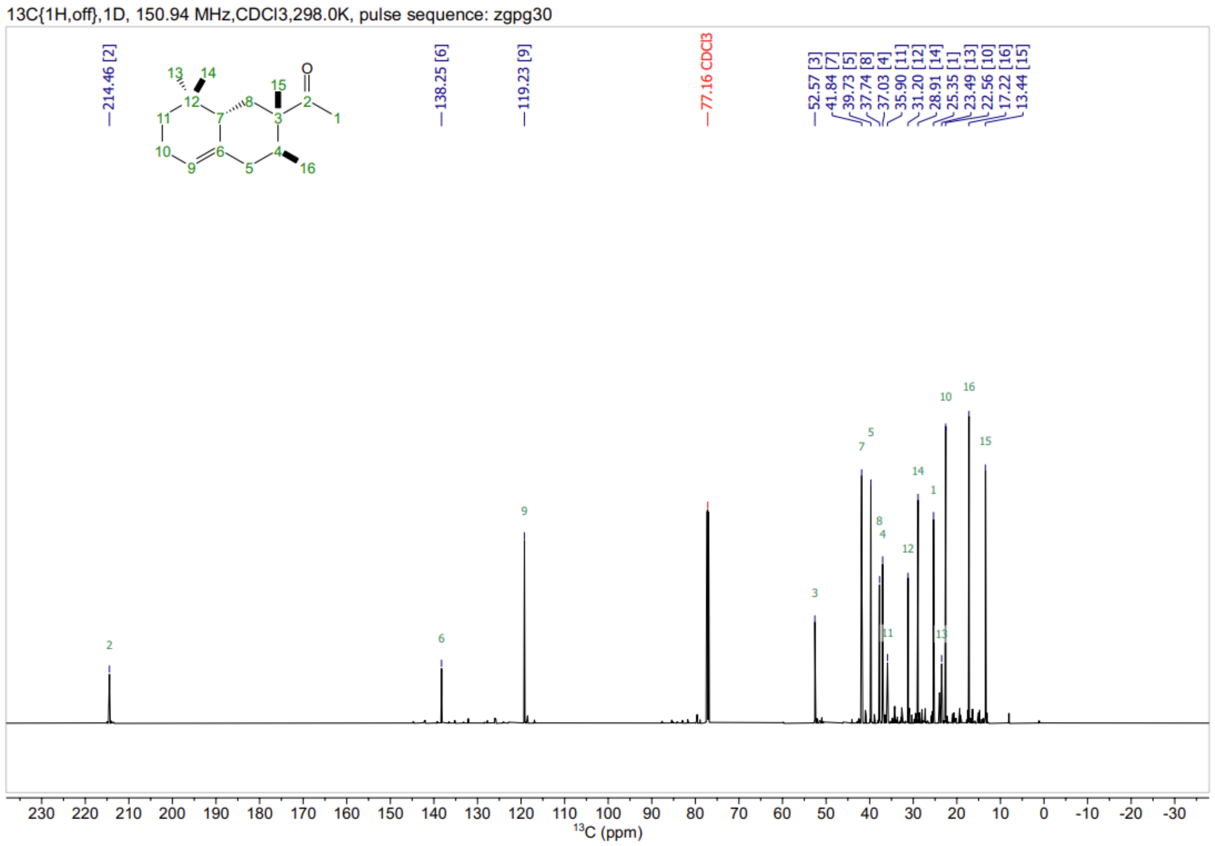

Figure S86. ^{1}H NMR and ^{13}C NMR spectra of compound **1f**.

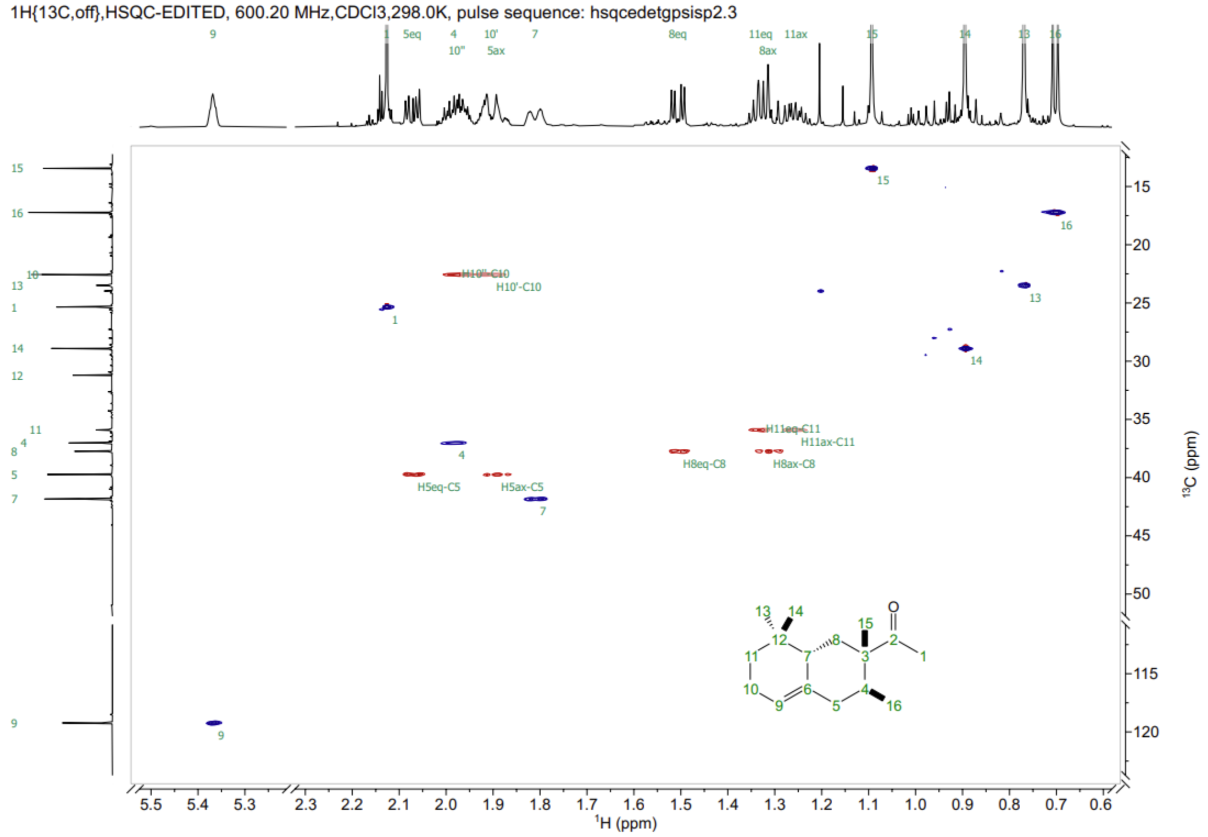

Figure S87. HSQC spectrum of compound **1f**.

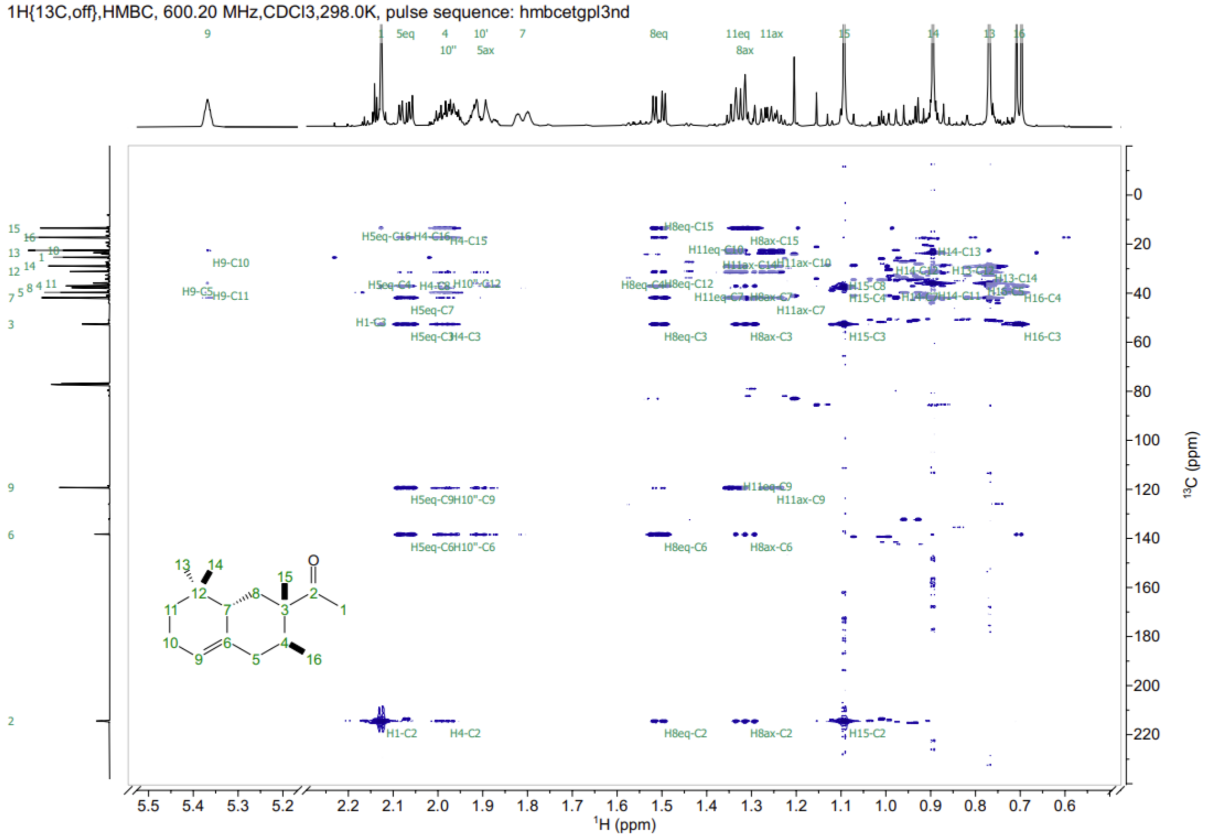

Figure S88. HMBC spectrum of compound **1f**.

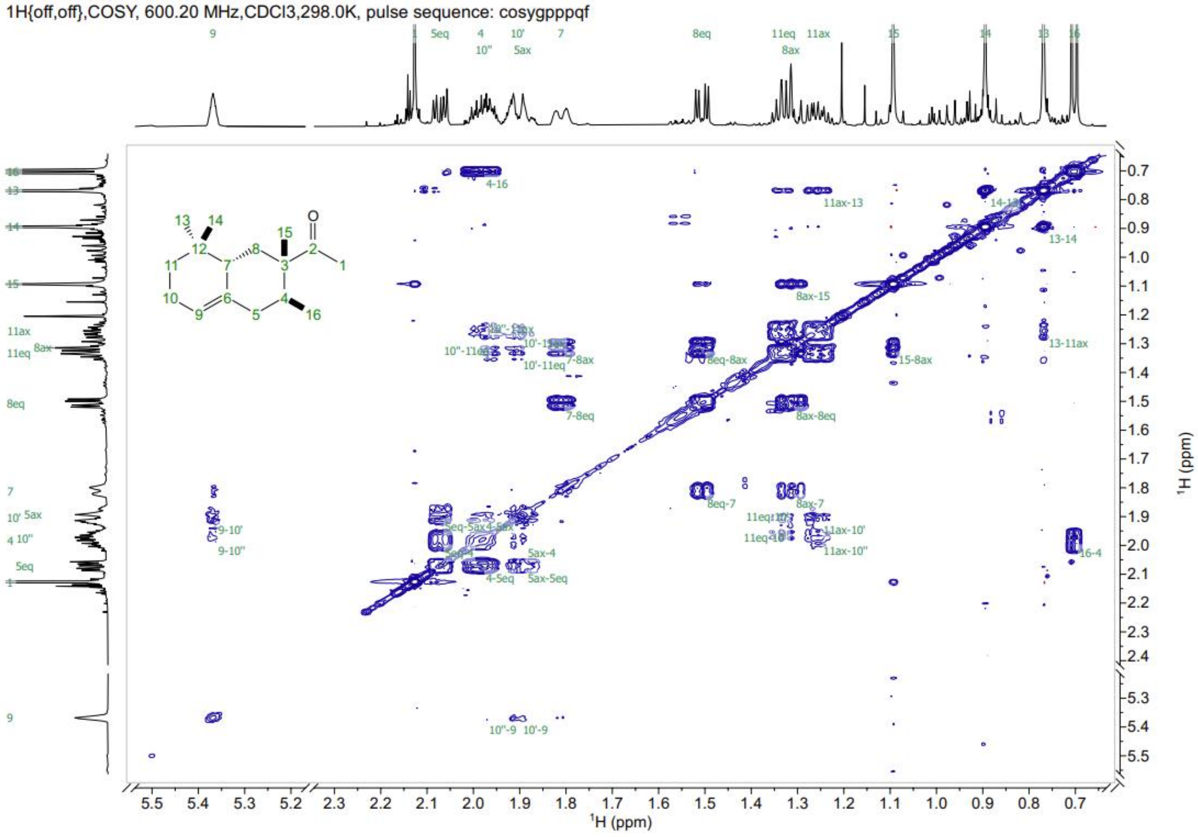

Figure S89. COSY spectrum of compound **1f**.

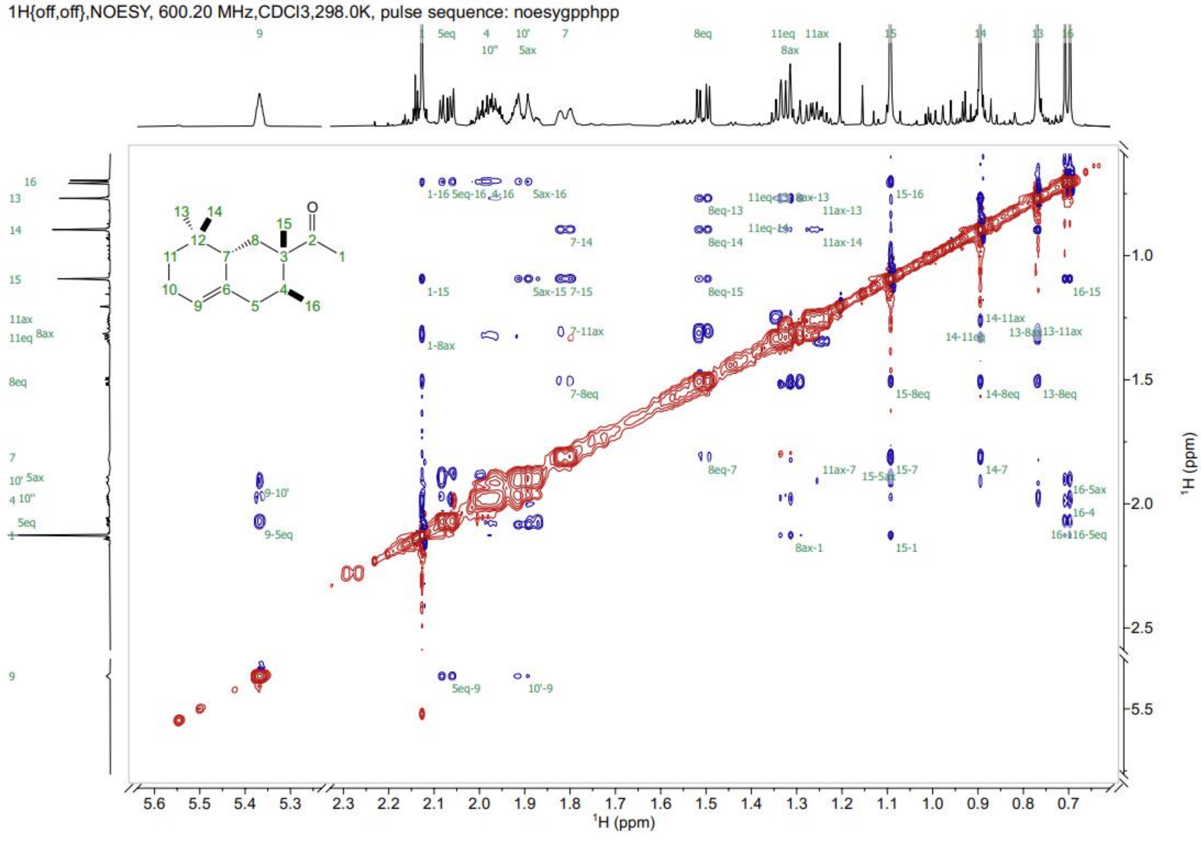

Figure S90. NOESY spectra of compound **1f**.

18.5.1. Sulfonamides

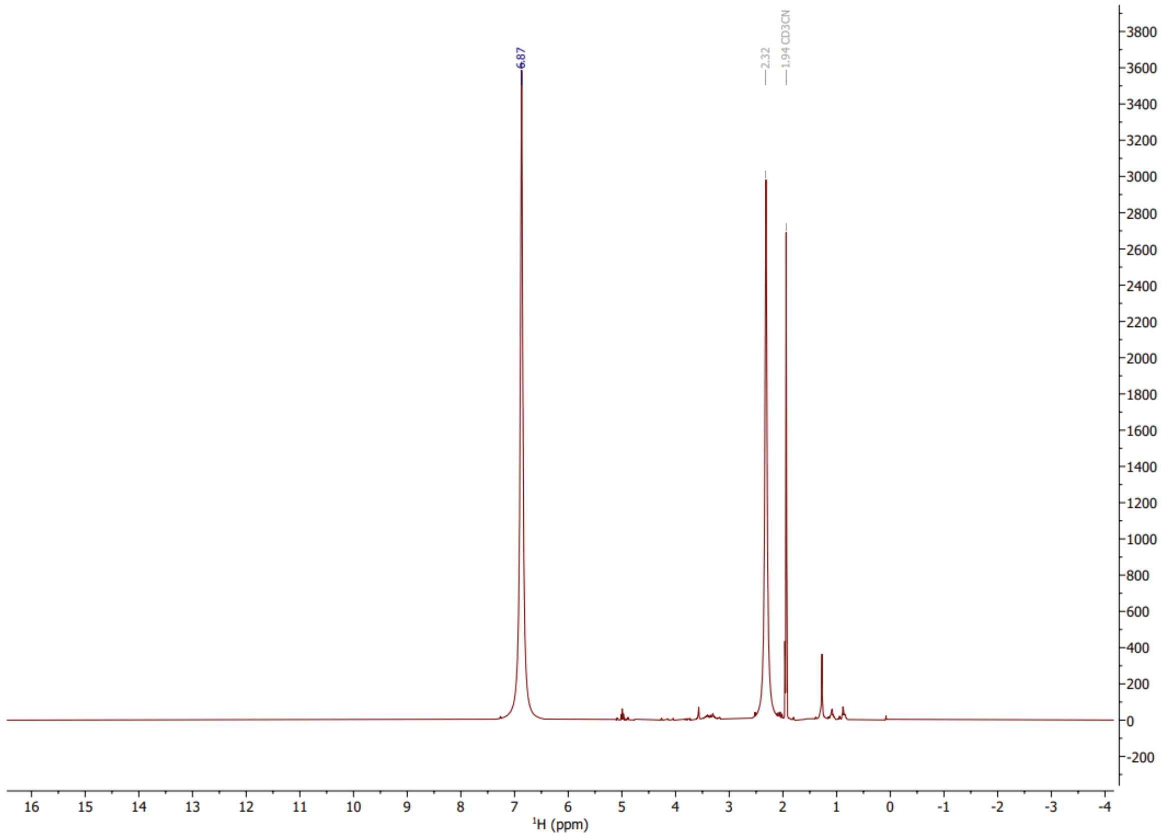

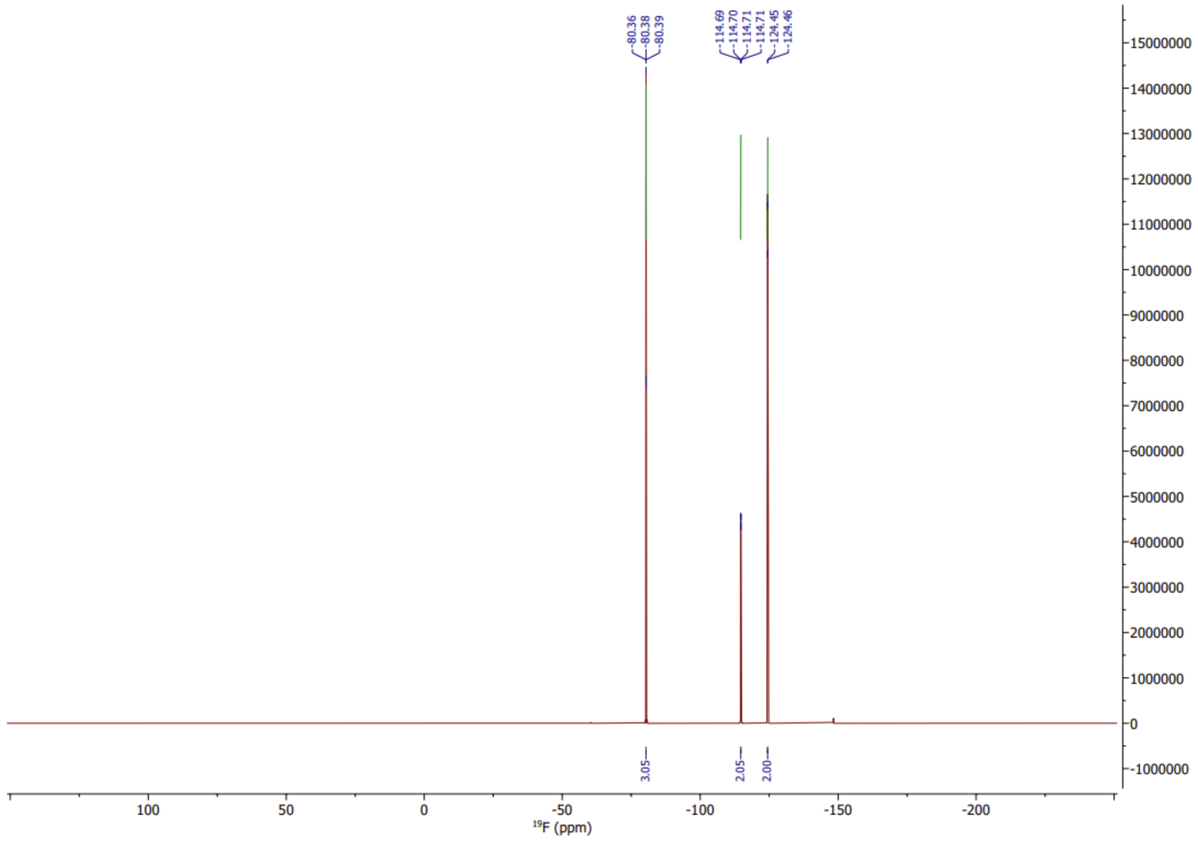

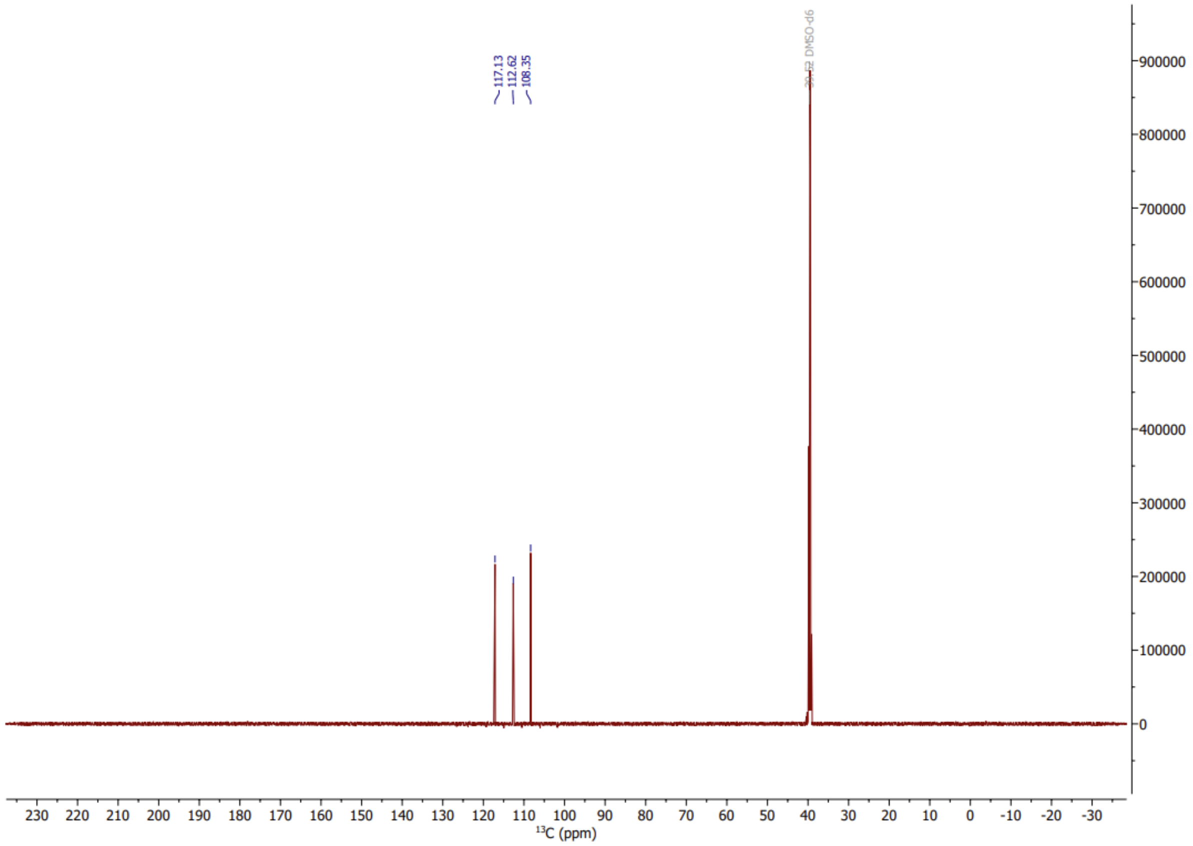

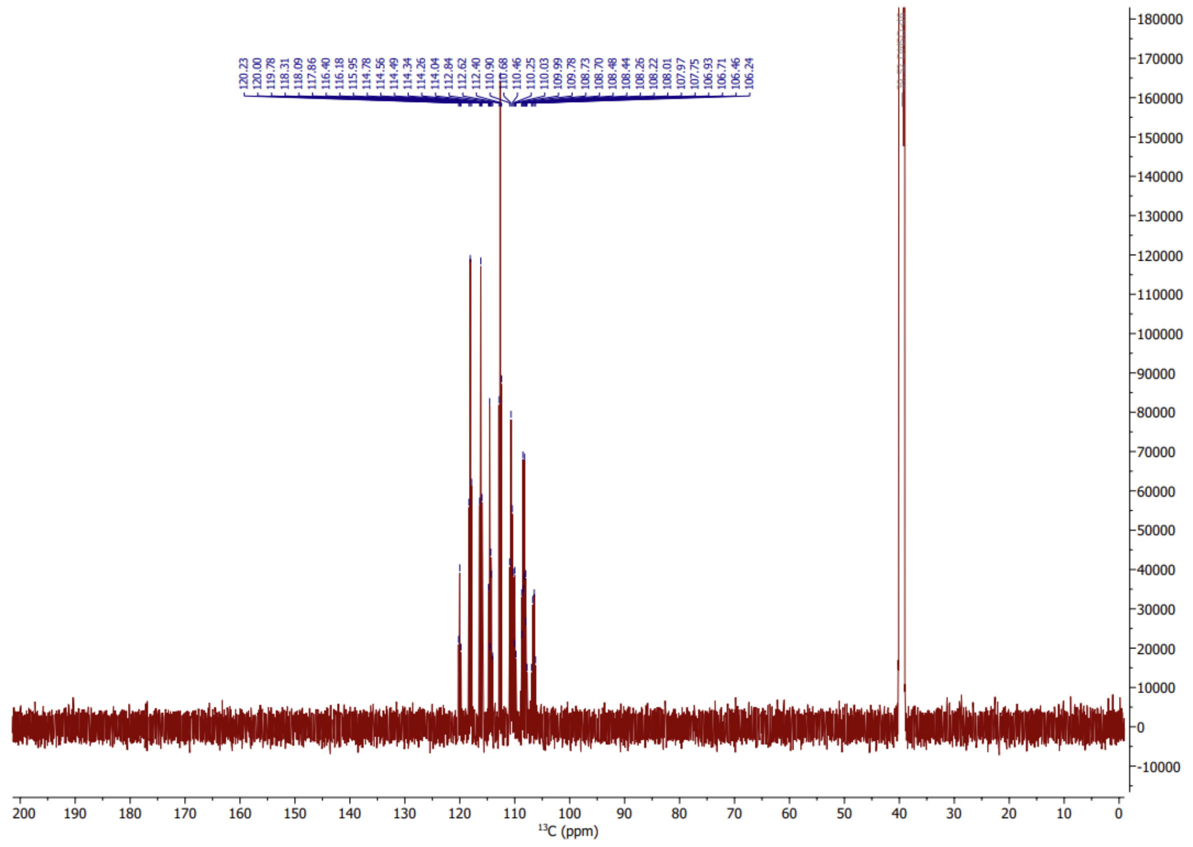

Figure S91. ^{1}H NMR, ^{19}F NMR, ^{13}C{^{19}F} NMR and ^{13}C NMR spectra of compound **7e**.

18.5.2. Phosphazine 8e

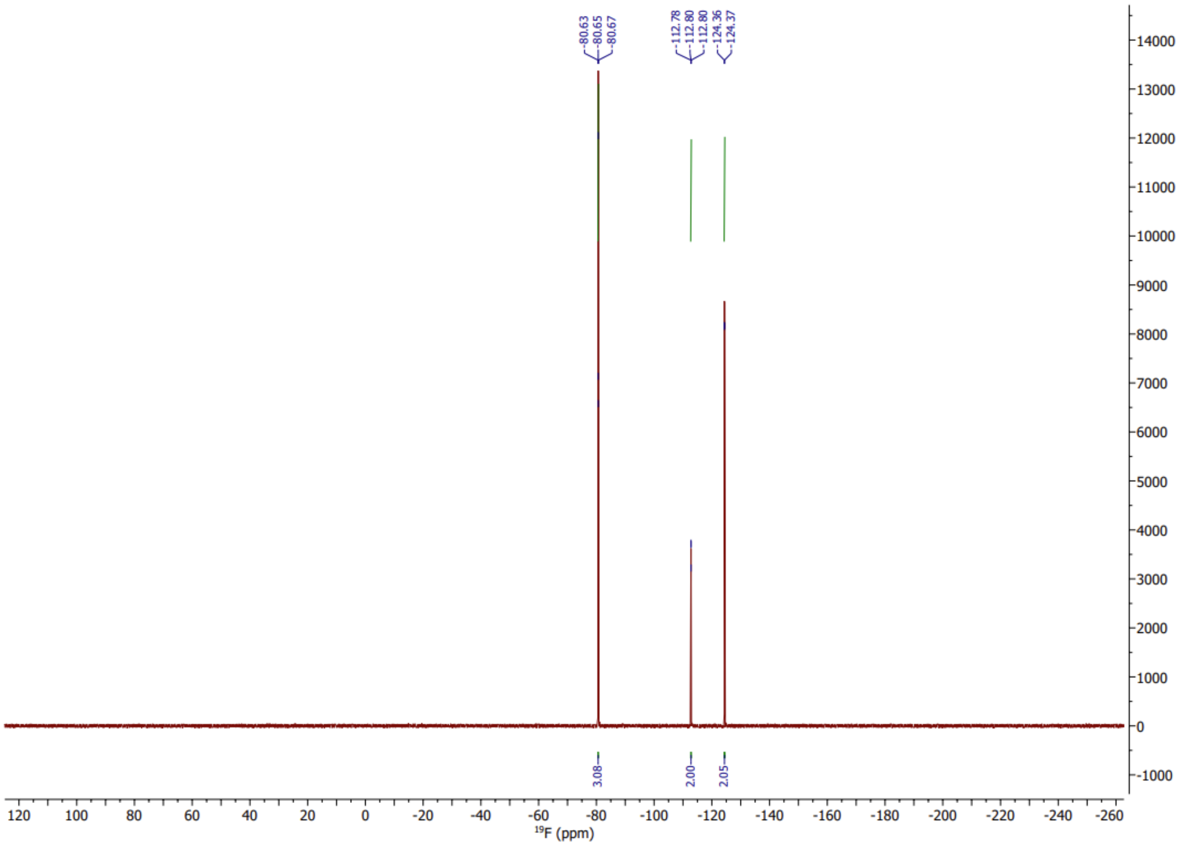

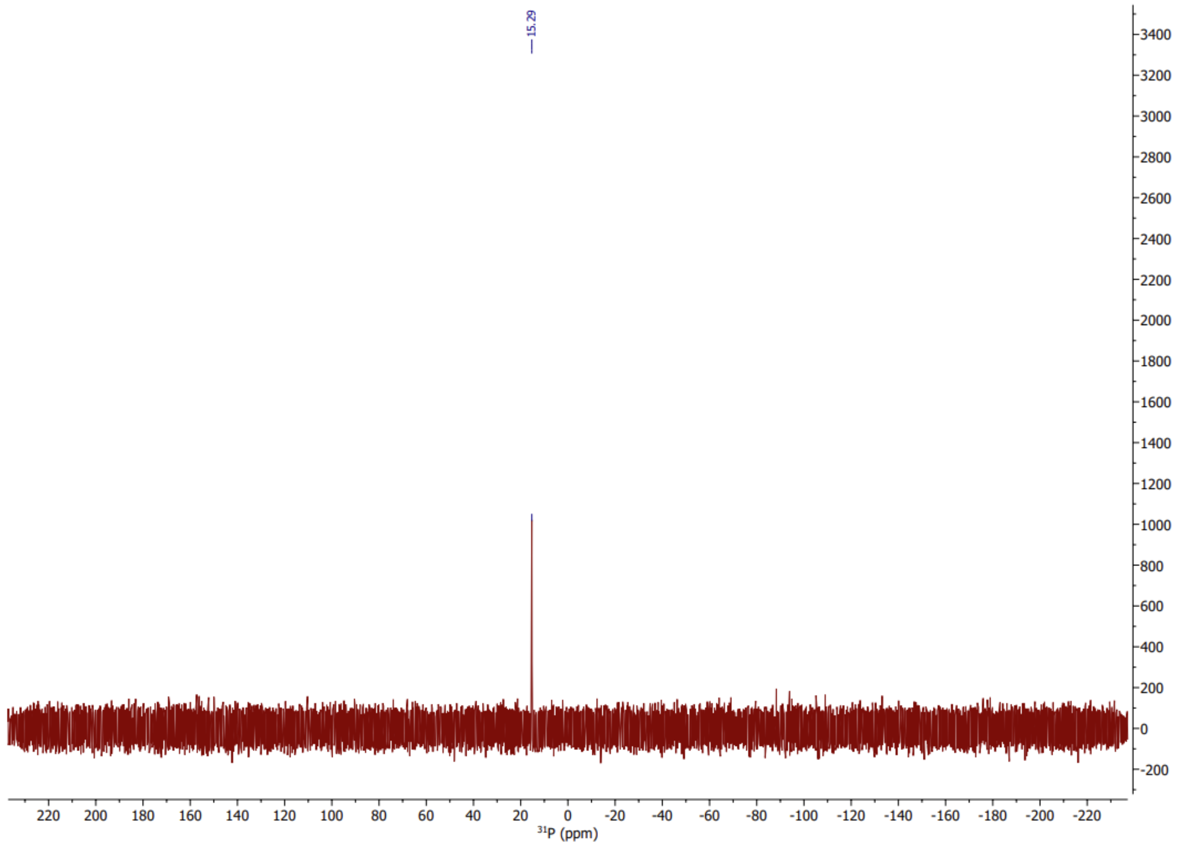

Figure S92. ^{19}F NMR, ^{31}P NMR spectra of compound **8e**.

18.5.3. (*S*)-BINOL

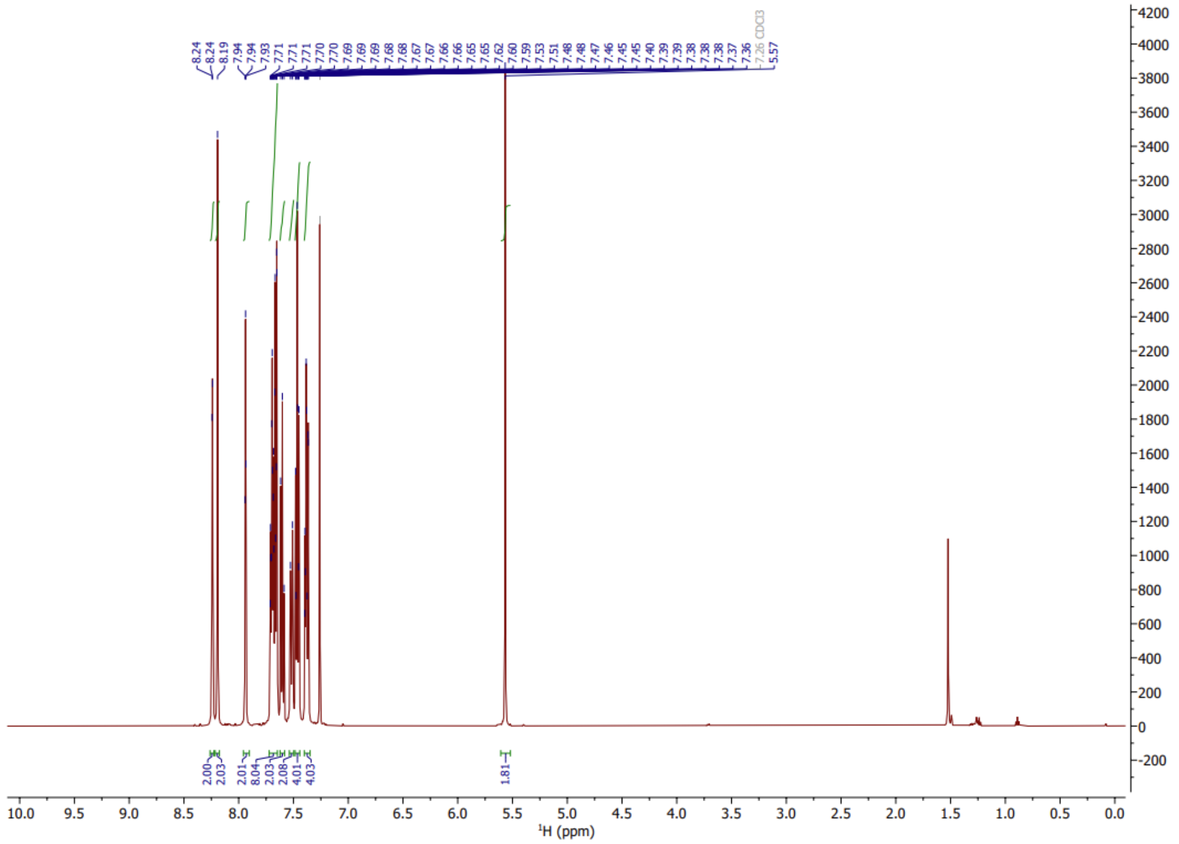
8.24
8.24
8.19
7.94
7.94
7.93
7.71
7.71
7.71
7.71
7.70
7.70
7.69
7.69
7.68
7.68
7.67
7.67
7.66
7.66
7.65
7.65
7.62
7.60
7.59
7.53
7.51
7.48
7.48
7.47
7.46
7.45
7.45
7.40
7.39
7.39
7.38
7.38
7.38
7.37
7.36
7.26 CDCl3
5.57
2.00
2.03
2.01
8.04
2.03
2.08
4.01
4.03
1.81
¹H (ppm)

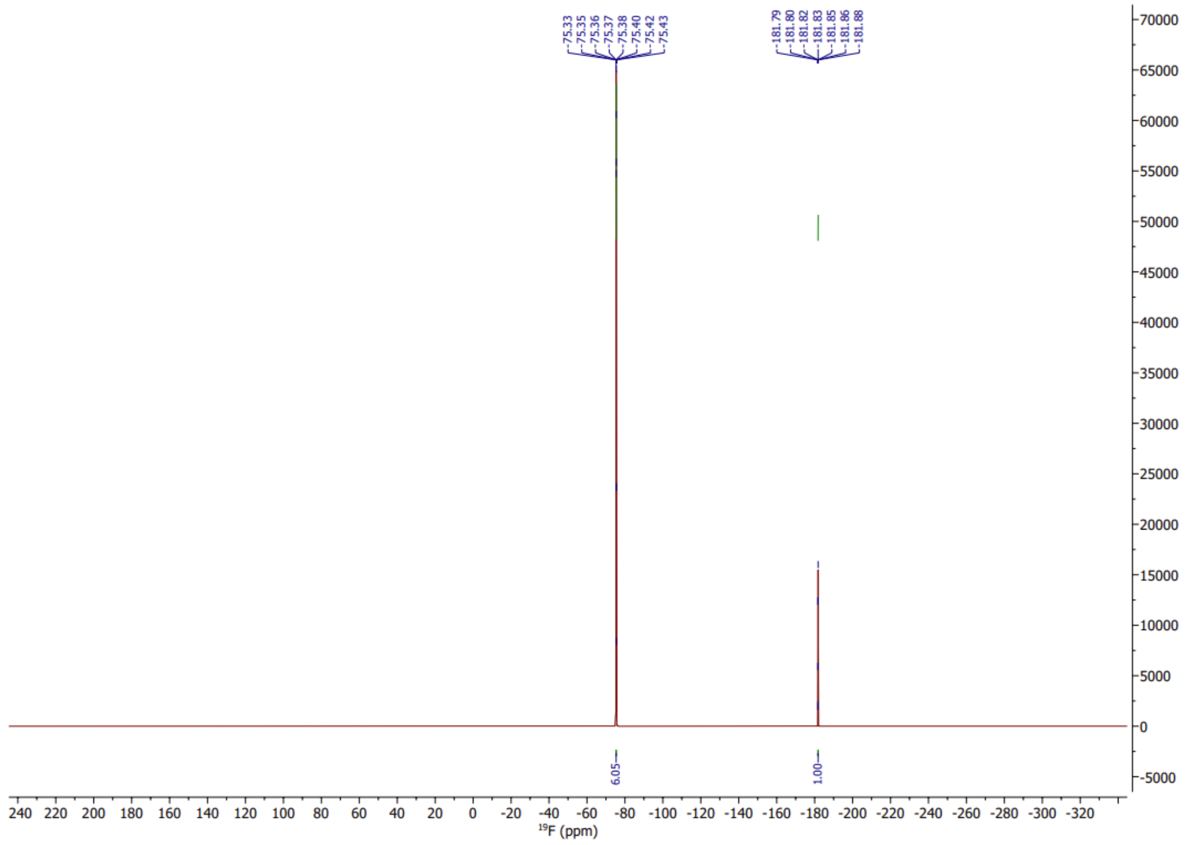
-75.33
-75.35
-75.36
-75.37
-75.38
-75.40
-75.42
-75.43
-181.79
-181.80
-181.82
-181.83
-181.85
-181.86
-181.88
6.05
1.00
¹⁹F (ppm)

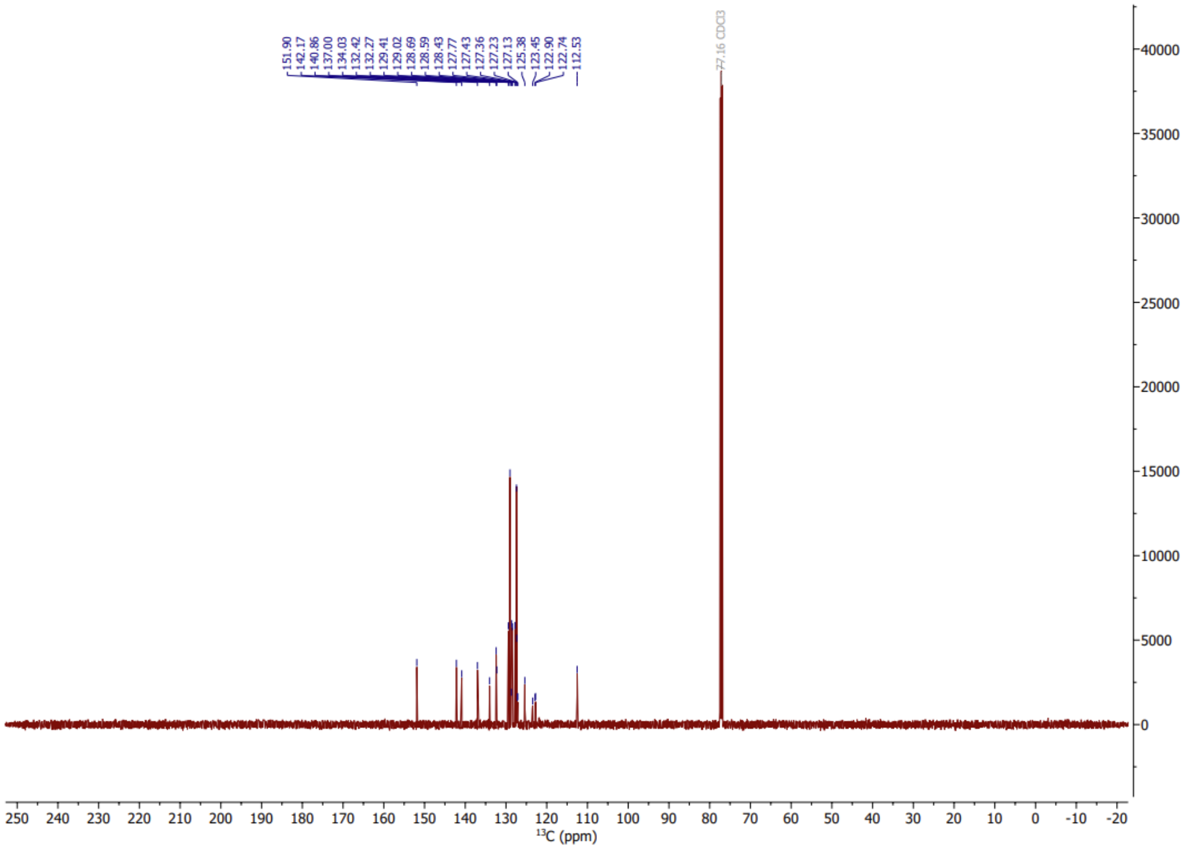

Figure S93. ^{1}H NMR, ^{19}F NMR, ^{13}C NMR spectra of compound **11f**.

18.6. IDPi Catalysts

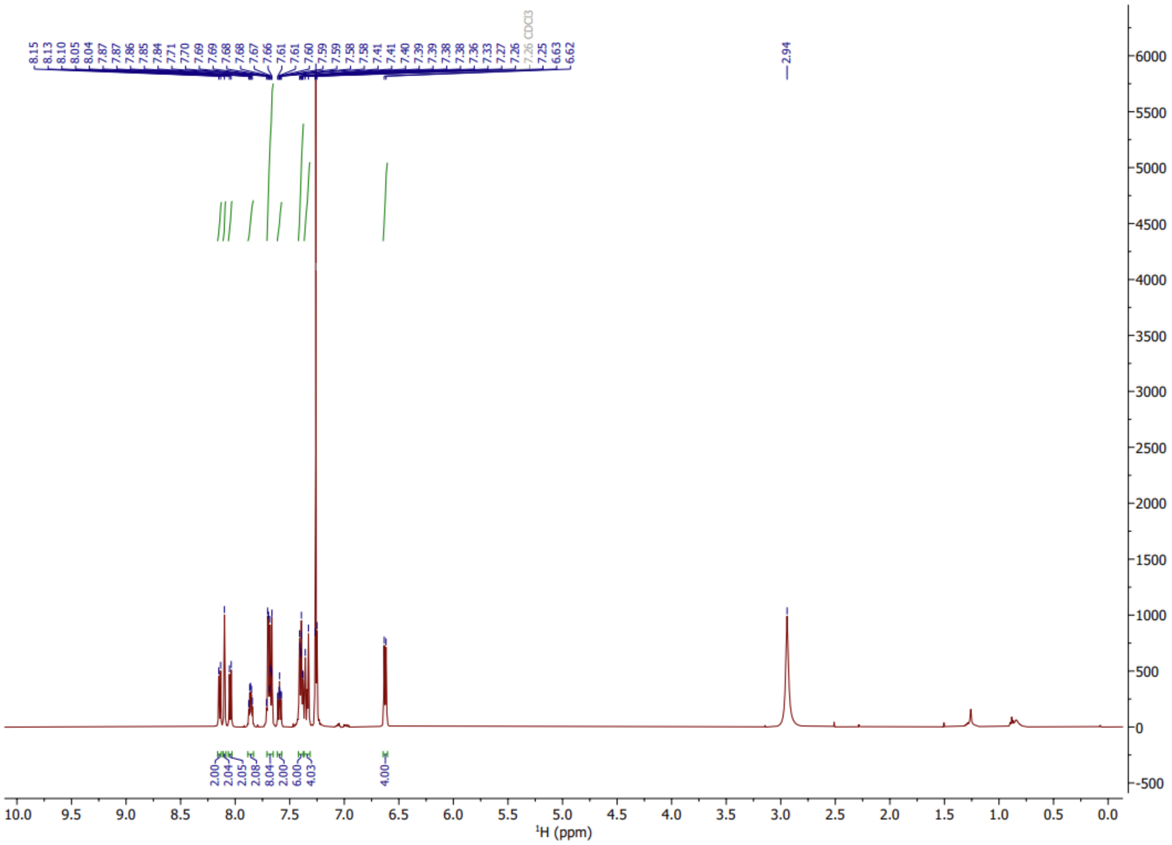

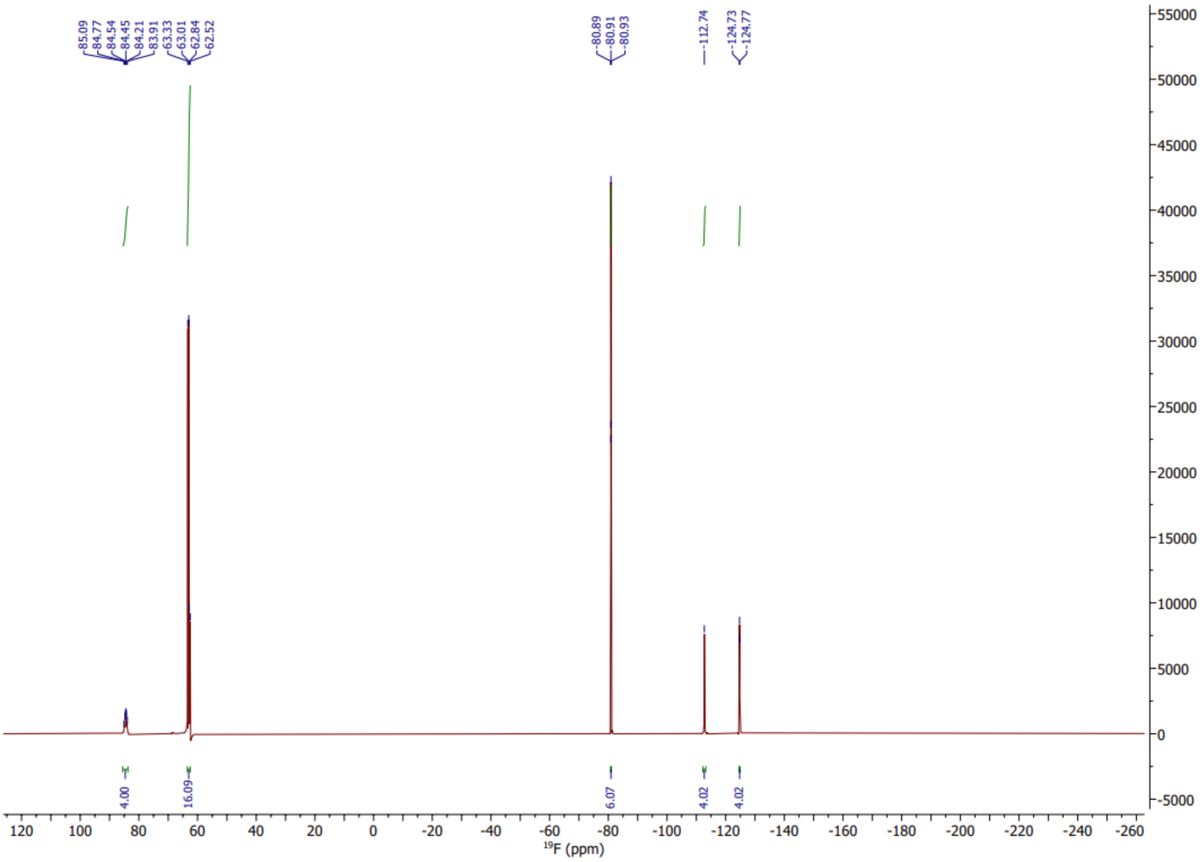

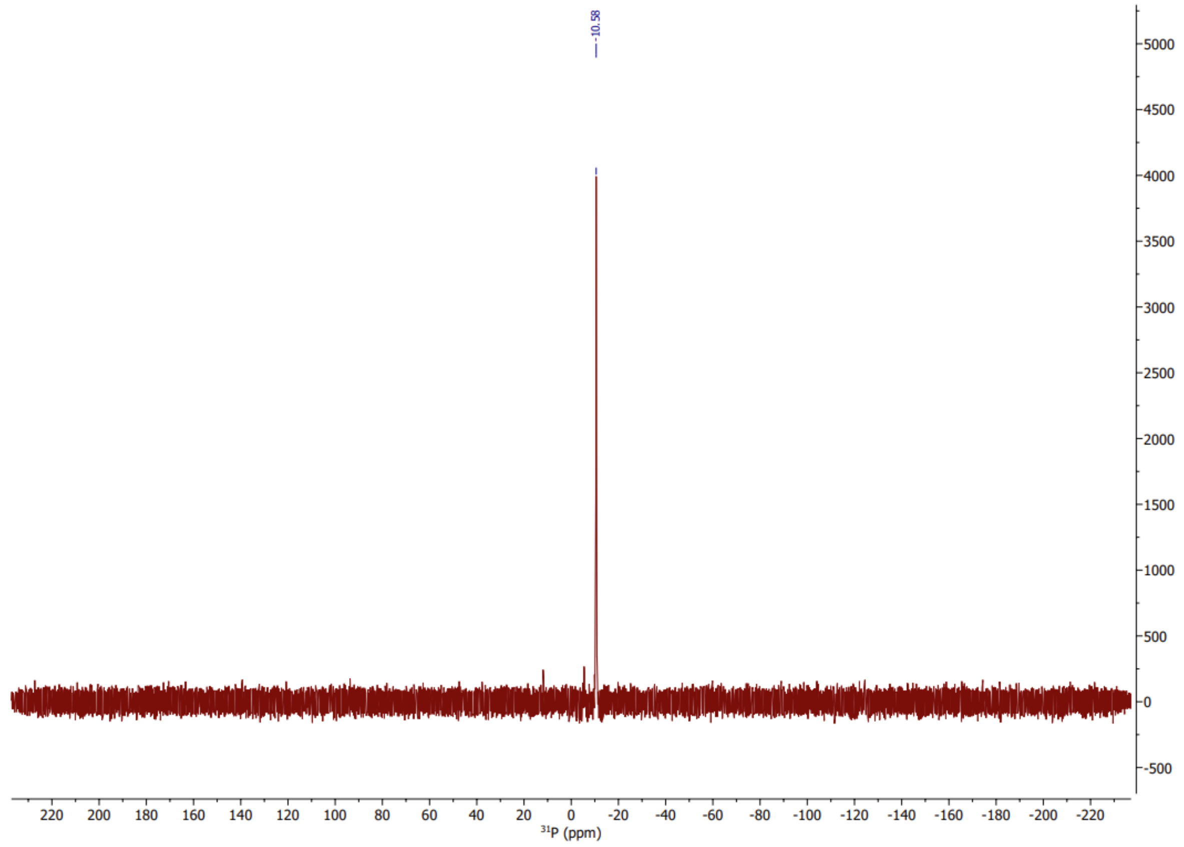

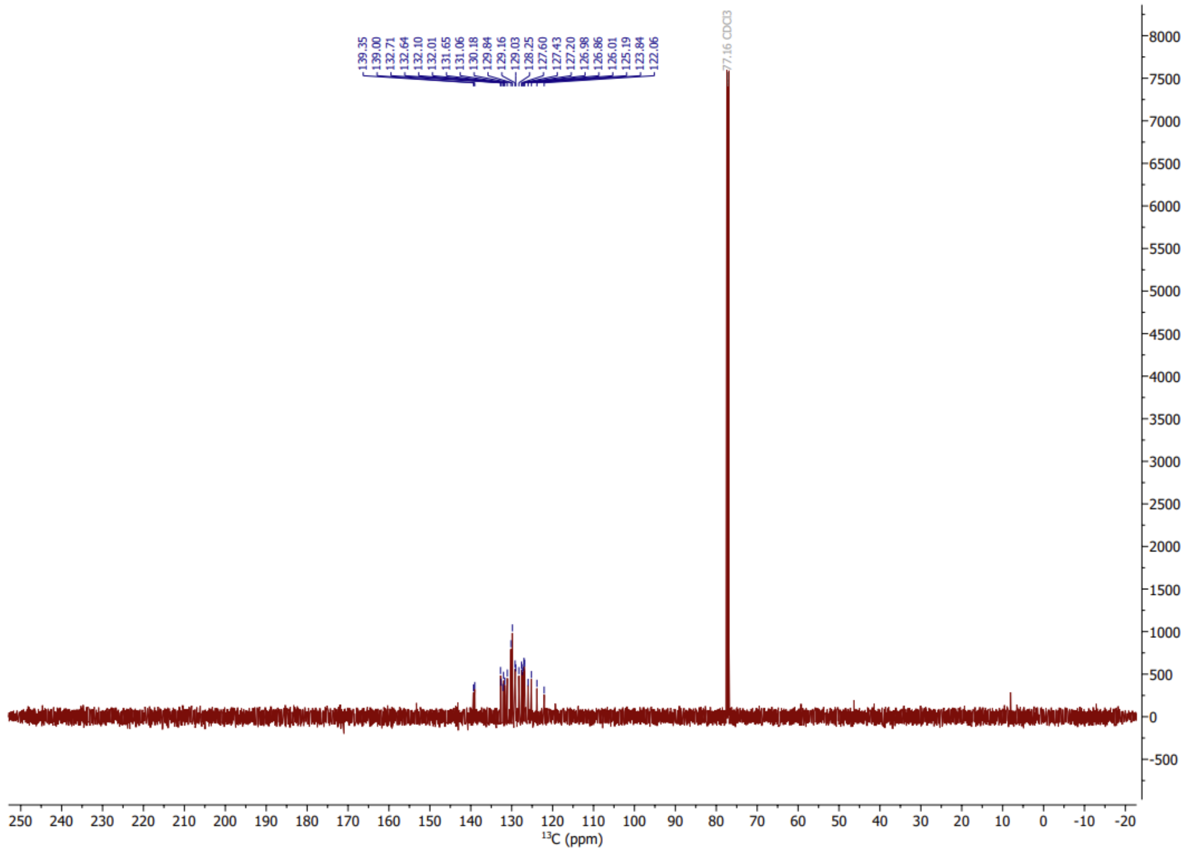

Figure S94. ^{1}H NMR, ^{19}F NMR, ^{31}P NMR and ^{13}C NMR spectra of compound **5h**.

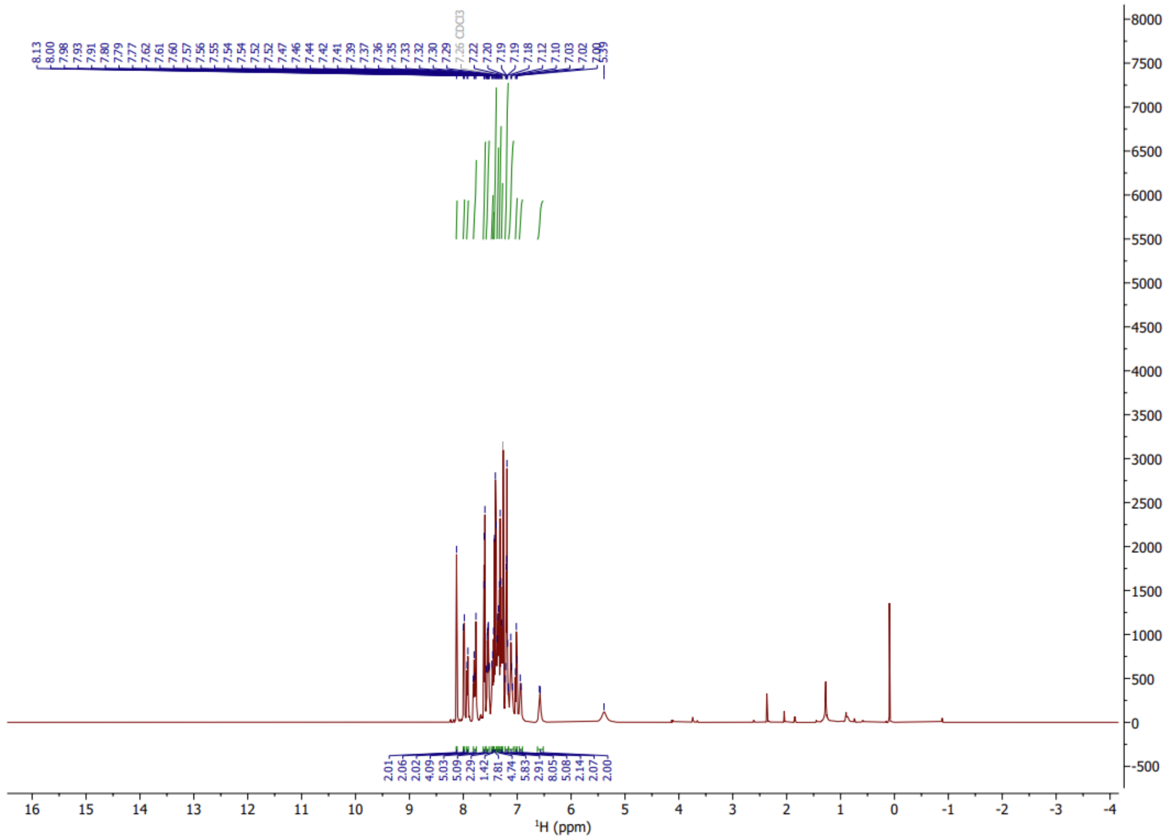

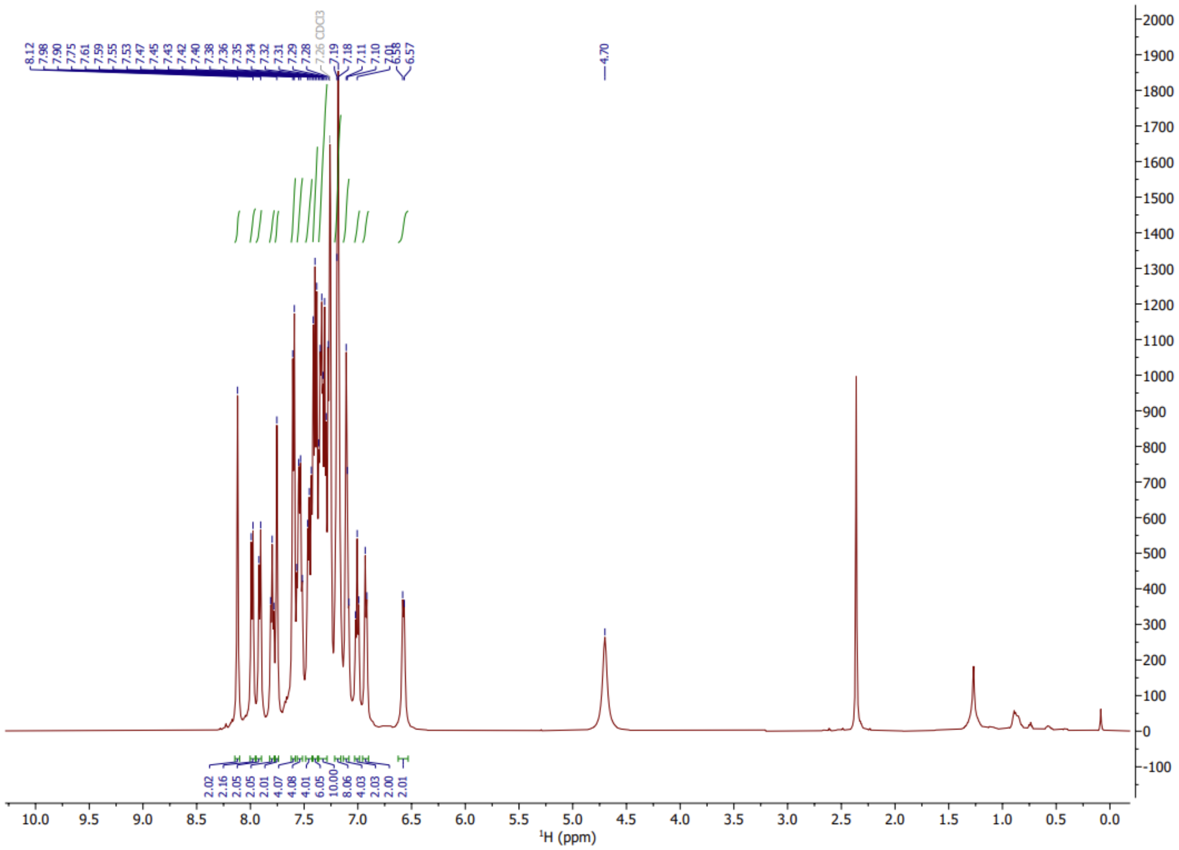

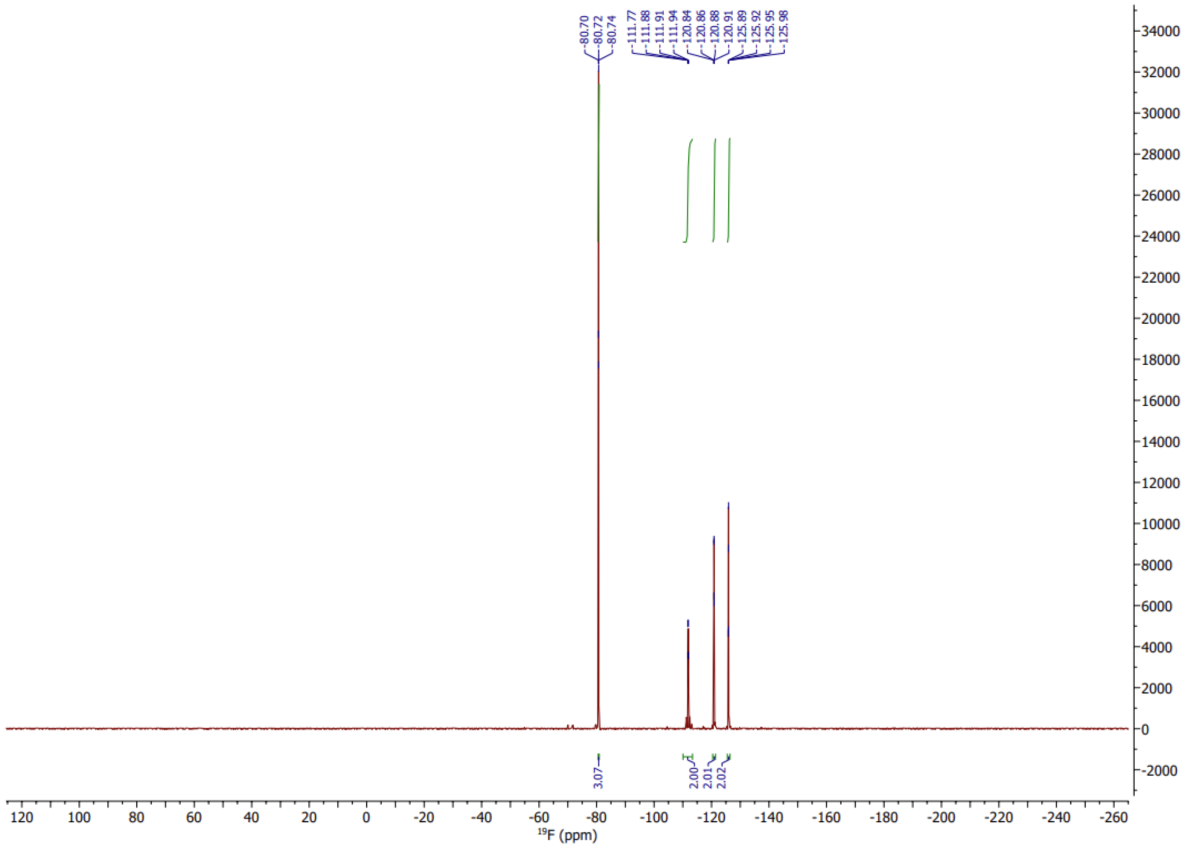
-80.70
-80.72
-80.74
-111.77
-111.88
-111.91
-111.94
-120.84
-120.86
-120.88
-120.91
-125.89
-125.92
-125.95
-125.98
3.07
2.00
2.01
2.02
¹⁹F (ppm)

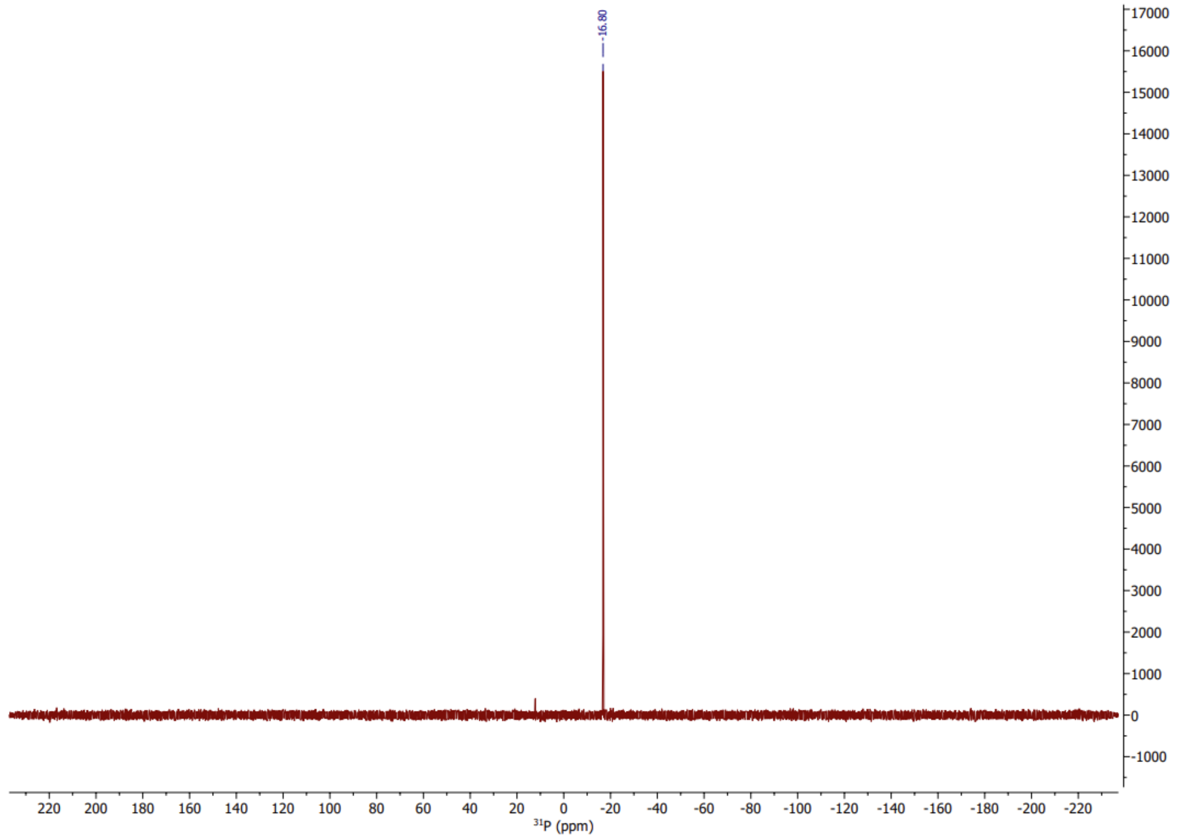
-16.80
31P (ppm)

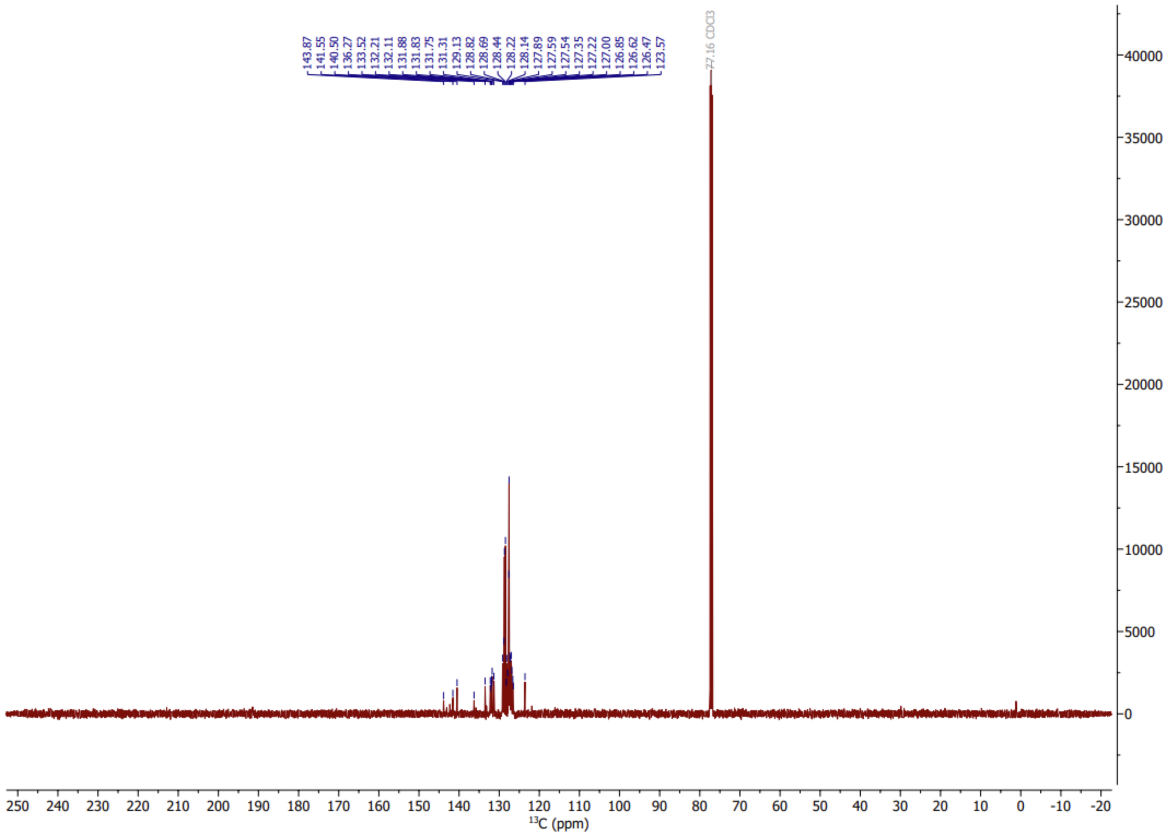

Figure S95. ^{1}H NMR, ^{1}H NMR (enhanced), ^{19}F NMR, ^{31}P NMR and ^{13}C NMR spectra of compound **5l**.

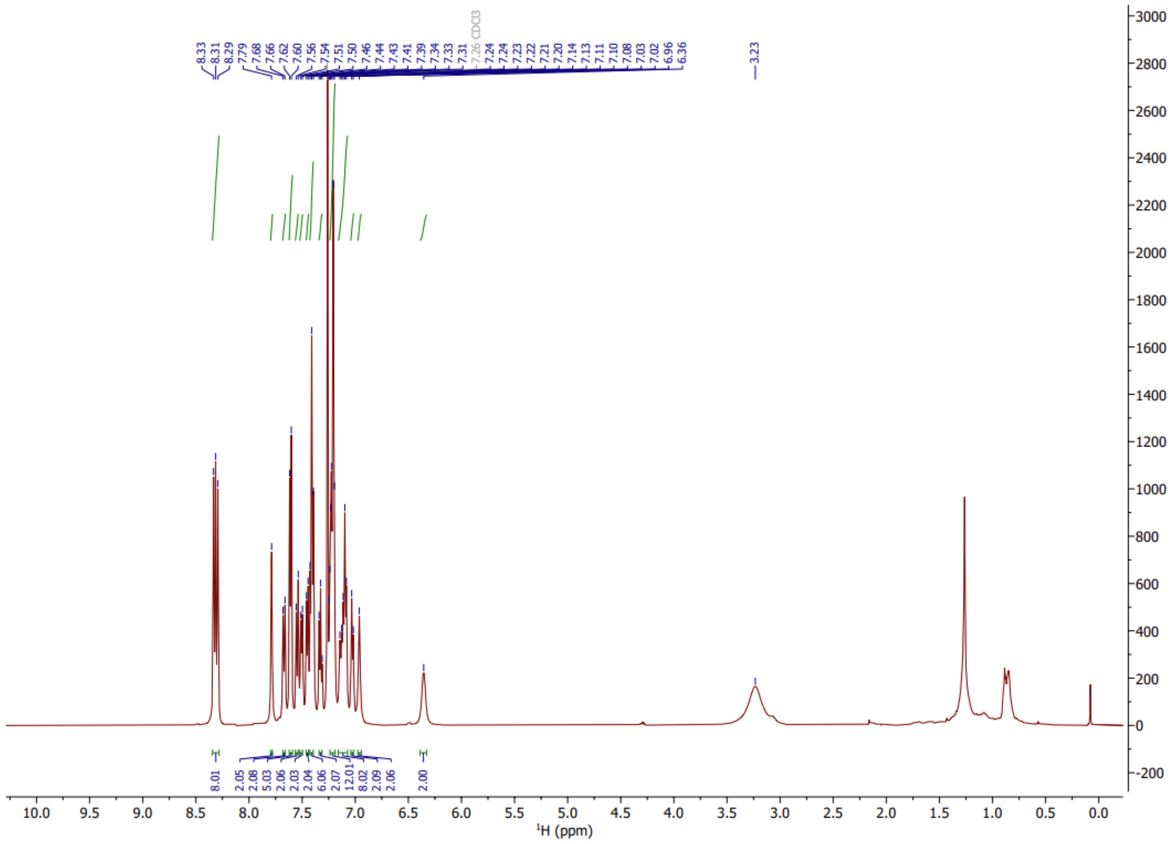

CDCl3
¹H (ppm)

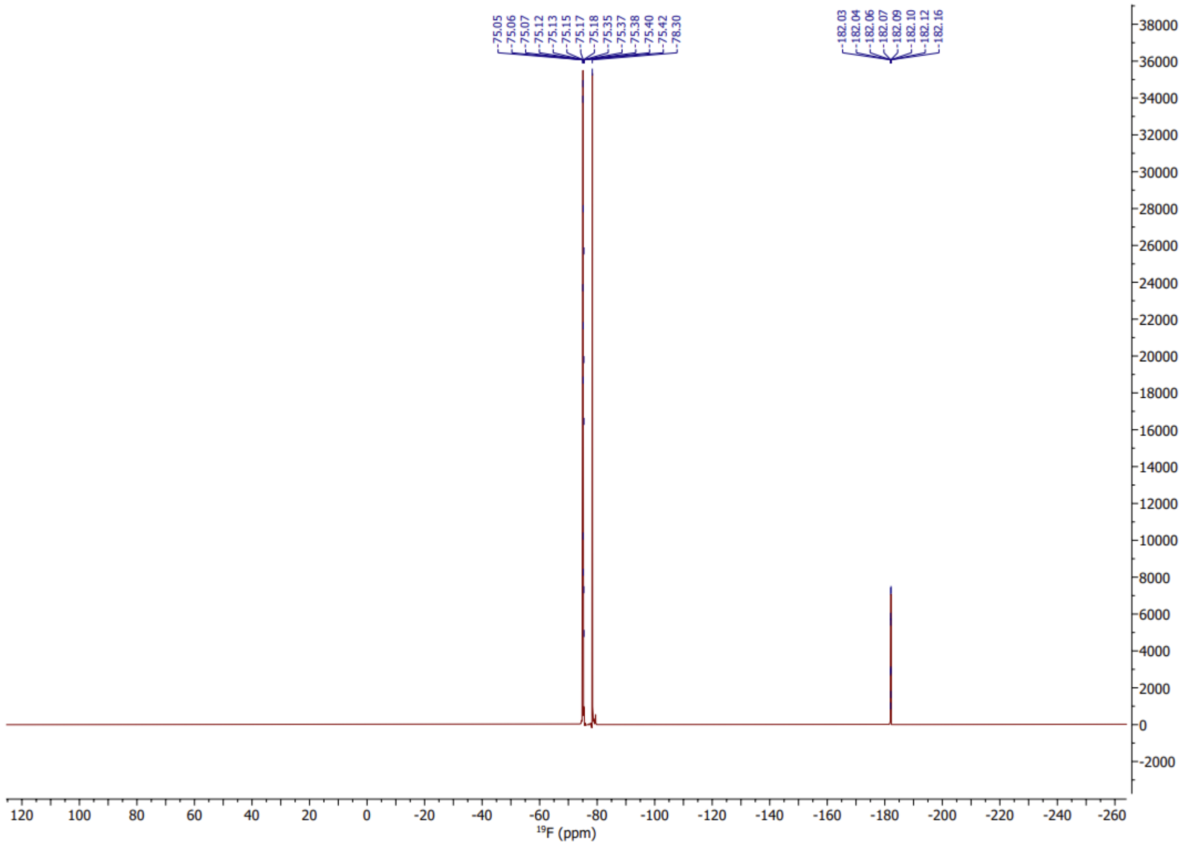

¹⁹F (ppm)

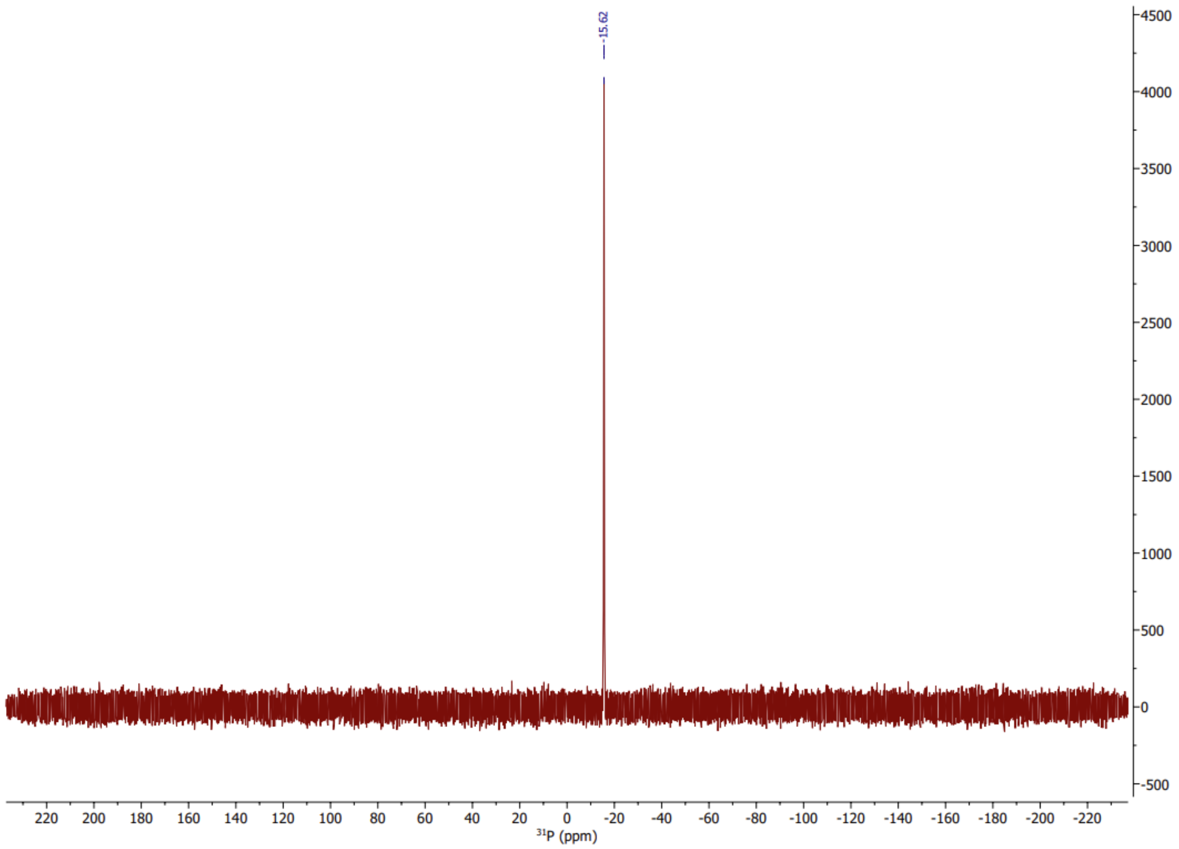

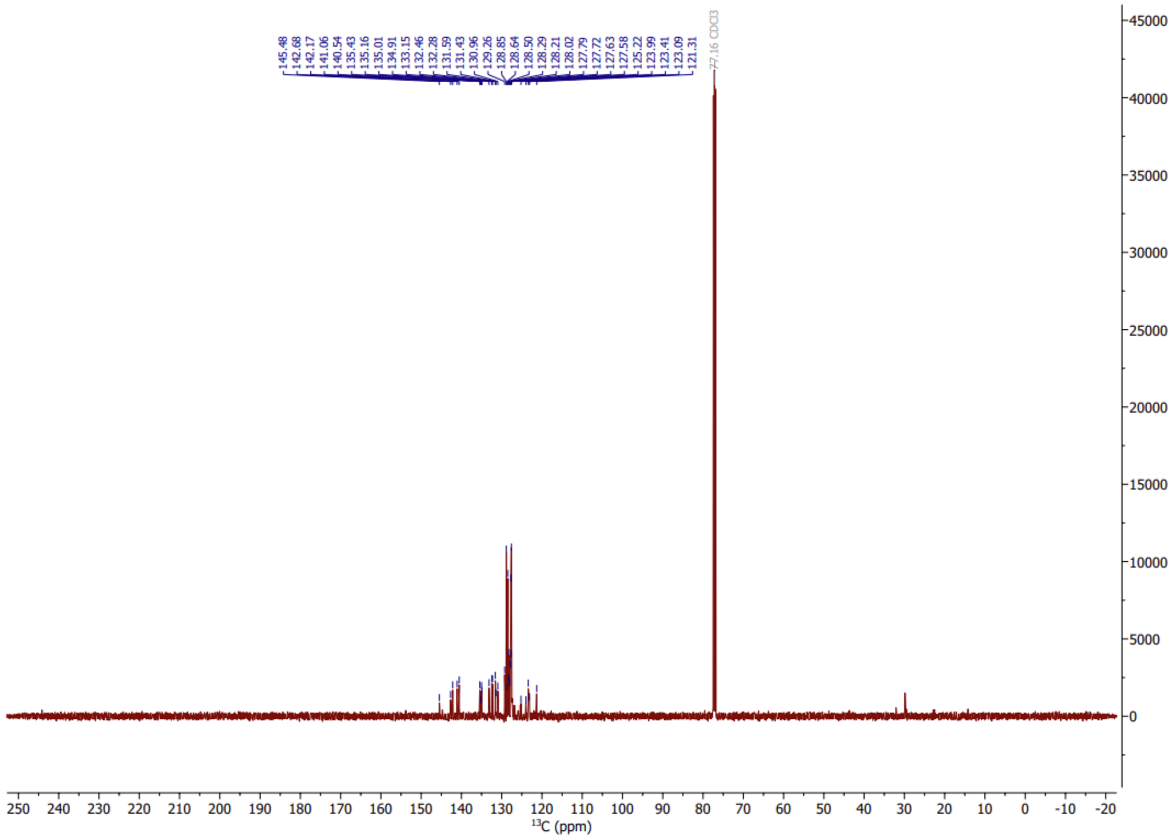

Figure S96. ^{1}H NMR, ^{19}F NMR, ^{31}P NMR and ^{13}C NMR spectra of compound **5m**.

18.7. Mosher esters

1H{off},1D, 600.20 MHz,CDCl3,298.0K, pulse sequence: zg30

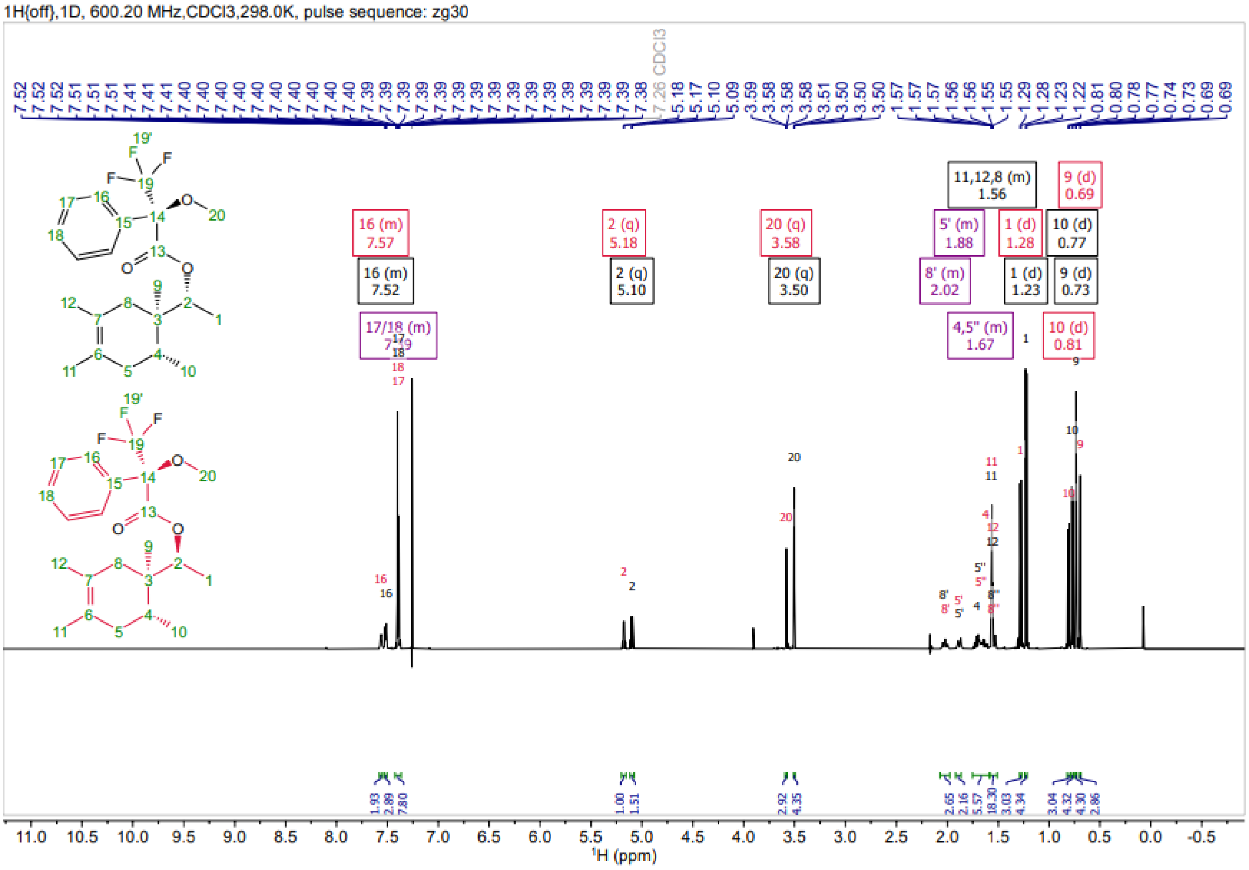

19F{off},1D, 564.72 MHz,CDCl3,298.0K, pulse sequence: zg30

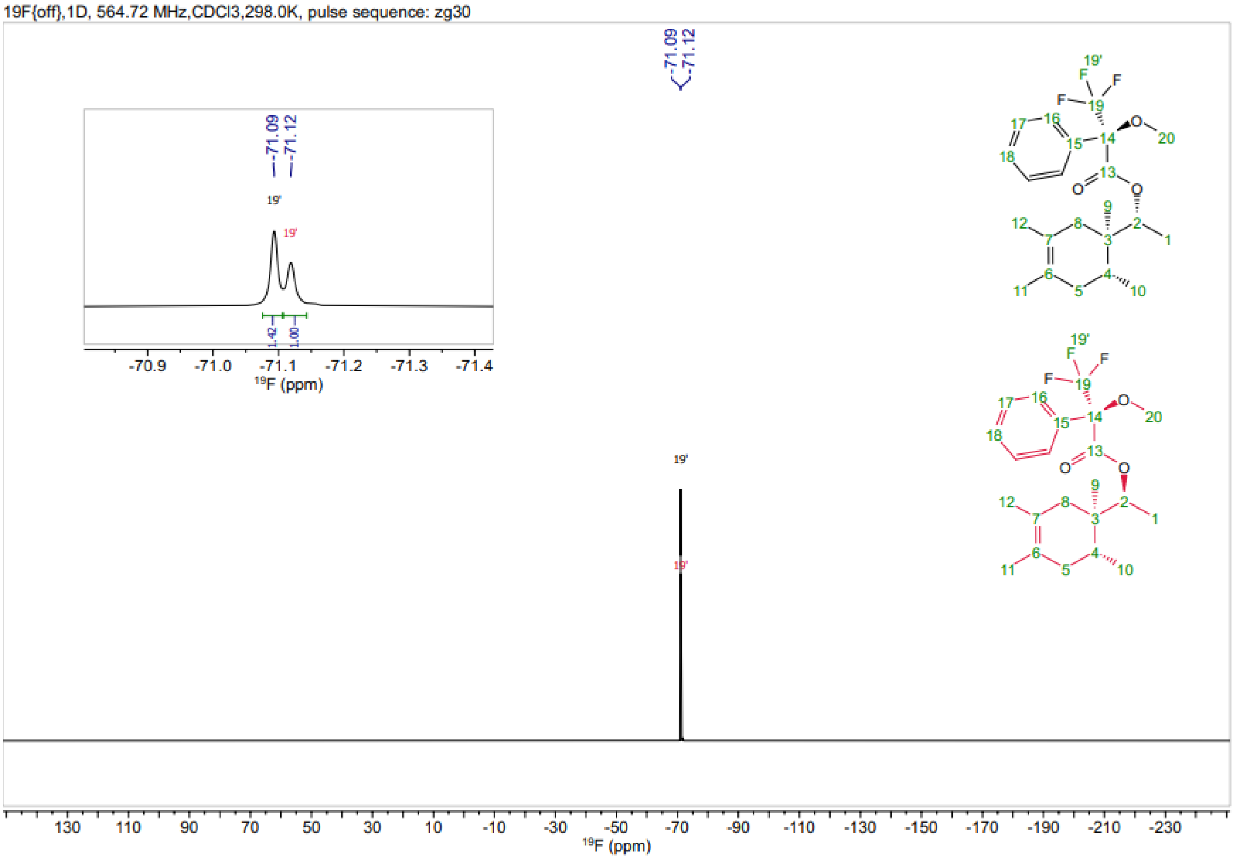

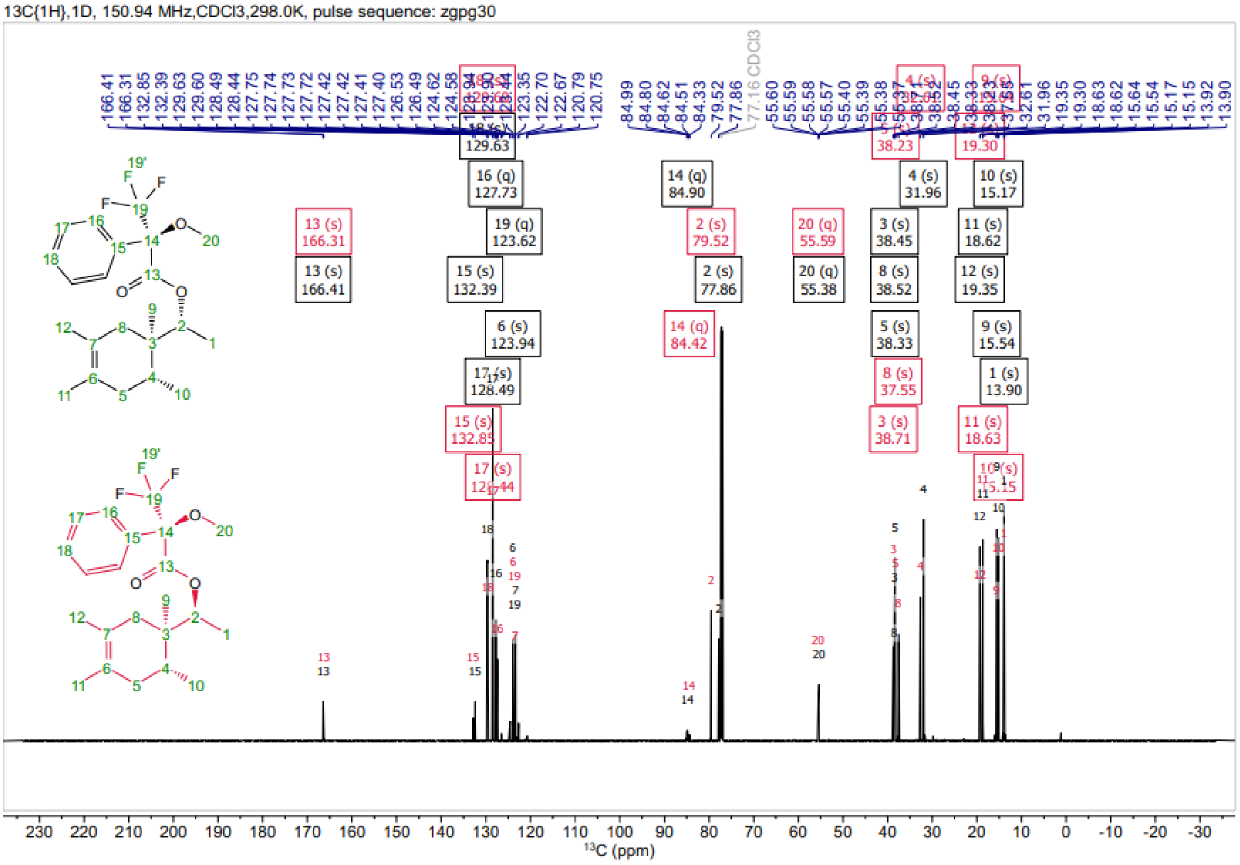

Figure S96. ^{1}H NMR, ^{19}F NMR and ^{13}C NMR spectra of compound **(*R*)-13r**.

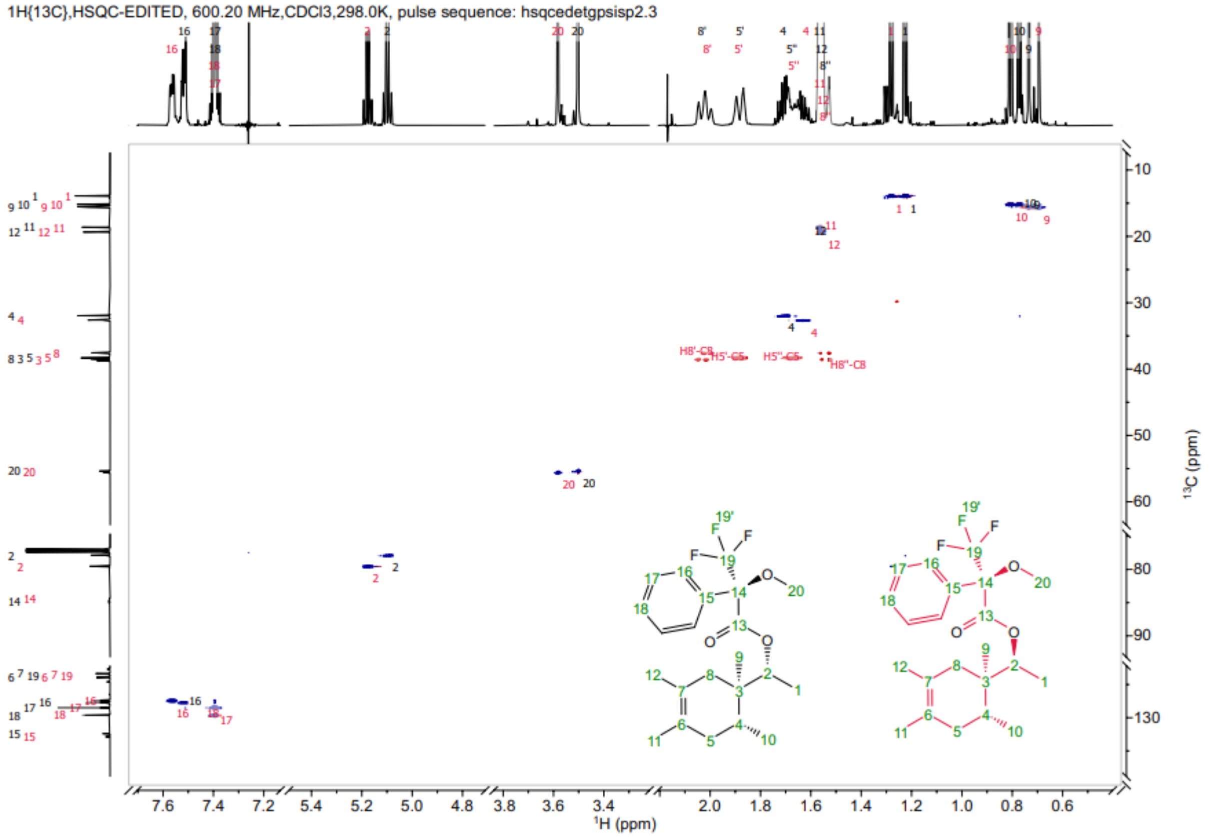

Figure S97. ^{1}H NMR – ^{13}C NMR HSQC spectrum of compound **(*R*)-13r**.

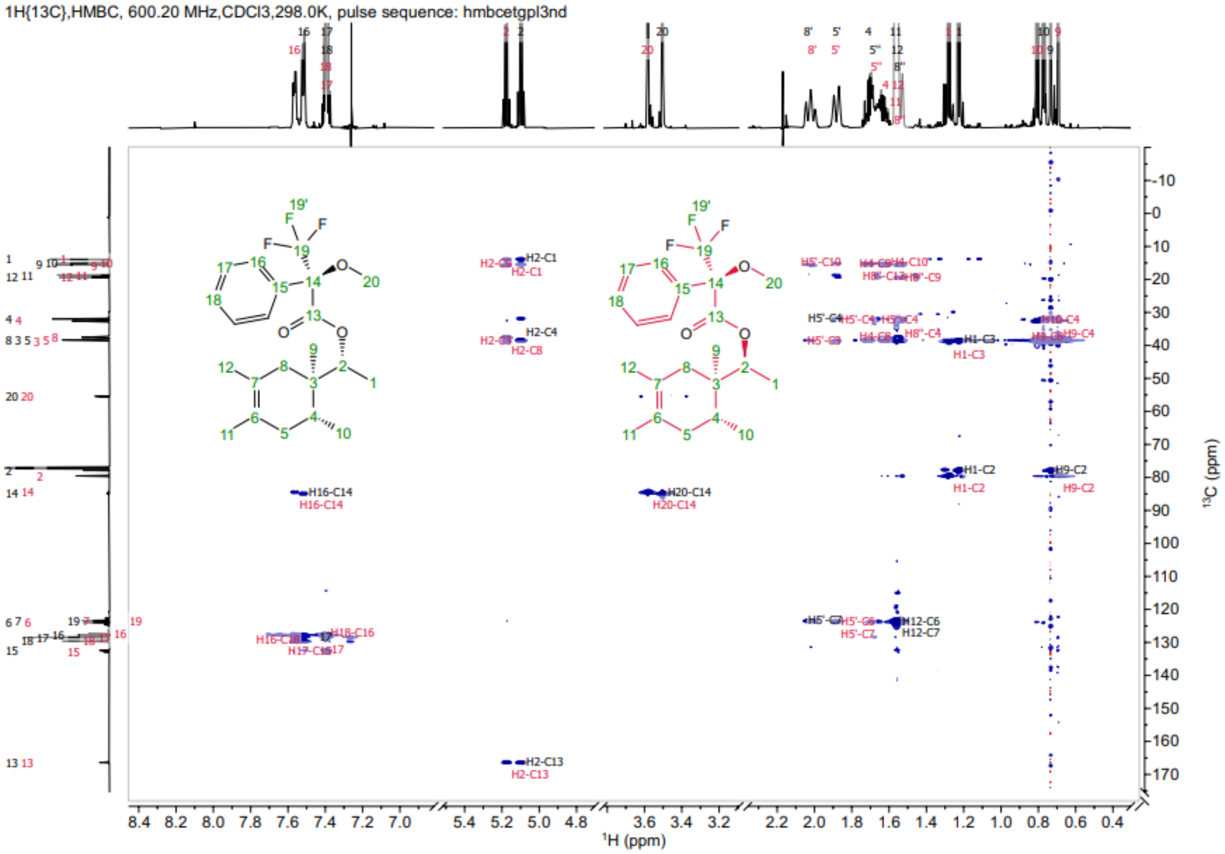

Figure S98. ^{1}HNMR – ^{13}CNMR HMBC spectrum of compound **(*R*)-13r**.

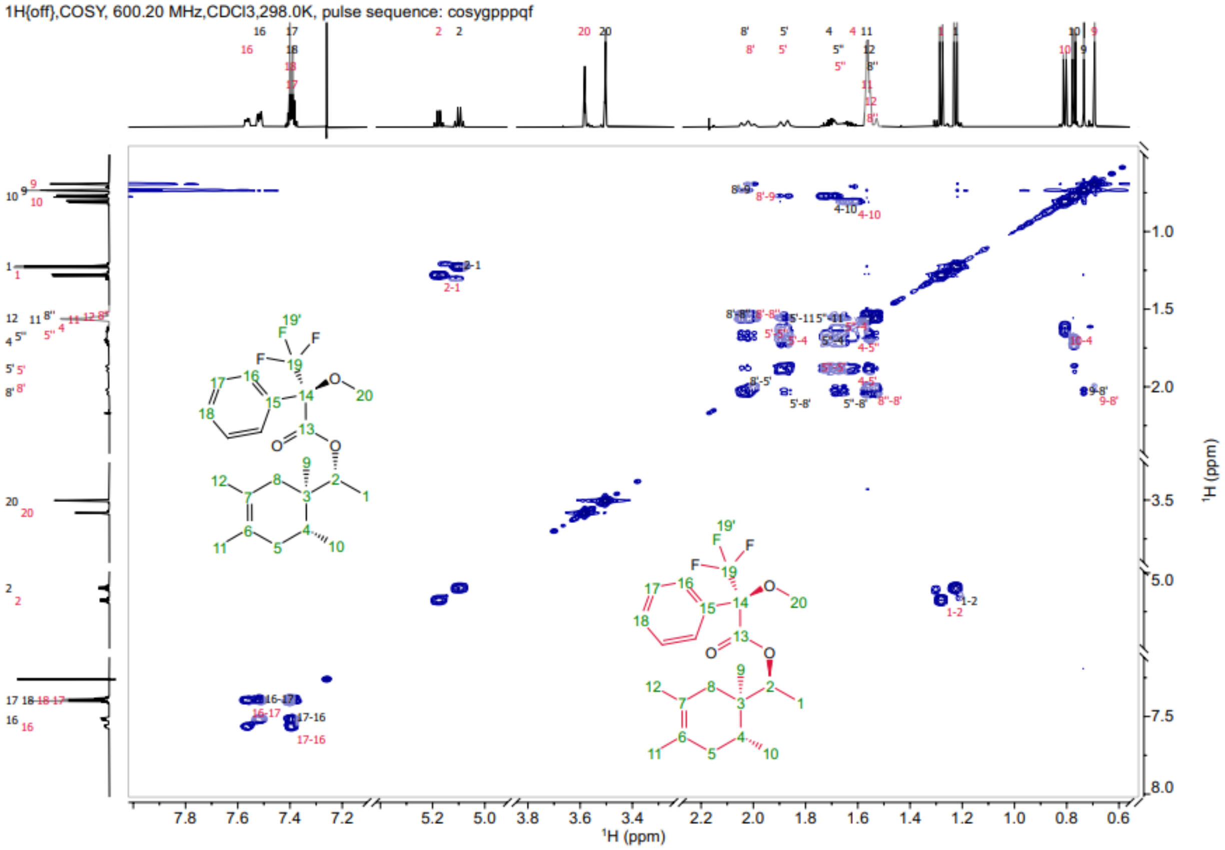

Figure S99. ^{1}H NMR COSY spectrum of compound **(*R*)-13r**.

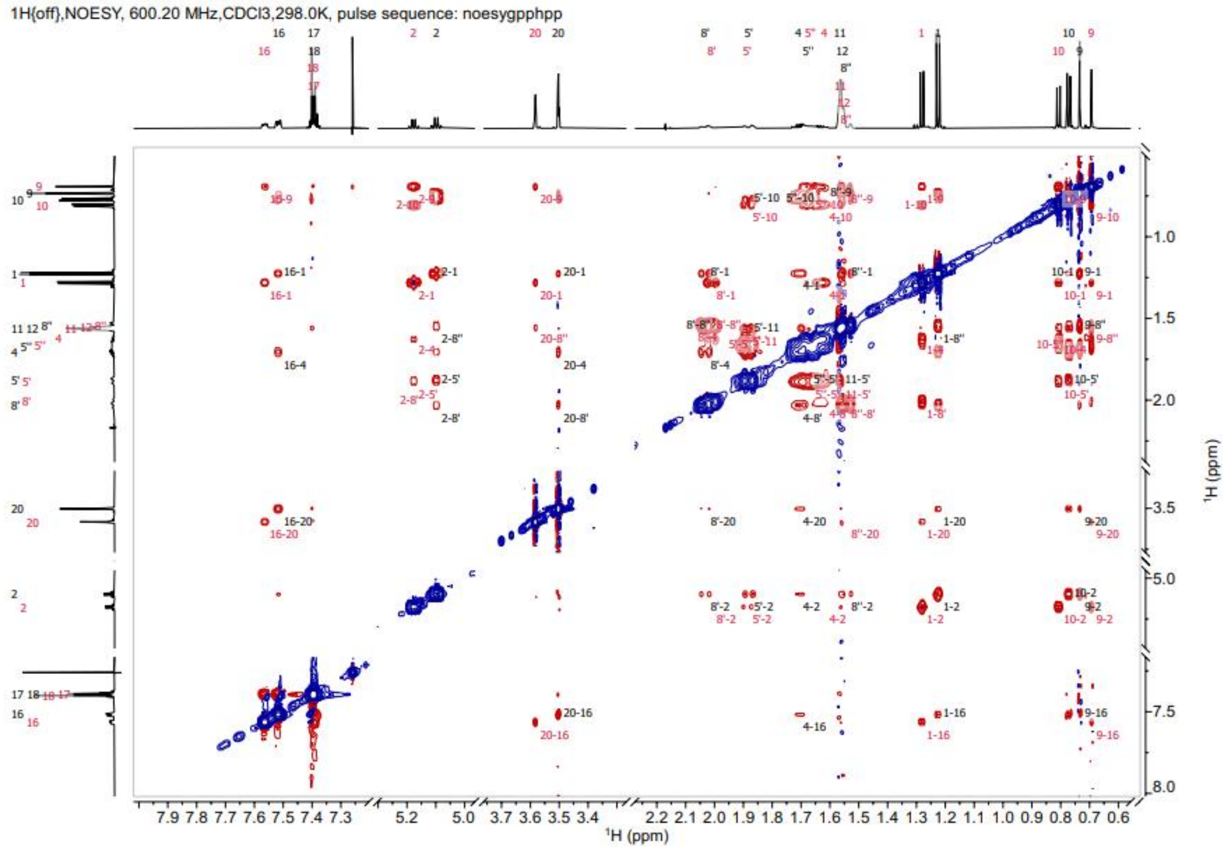

Figure S100. NOESY spectrum of compound **(*R*)-13r**.

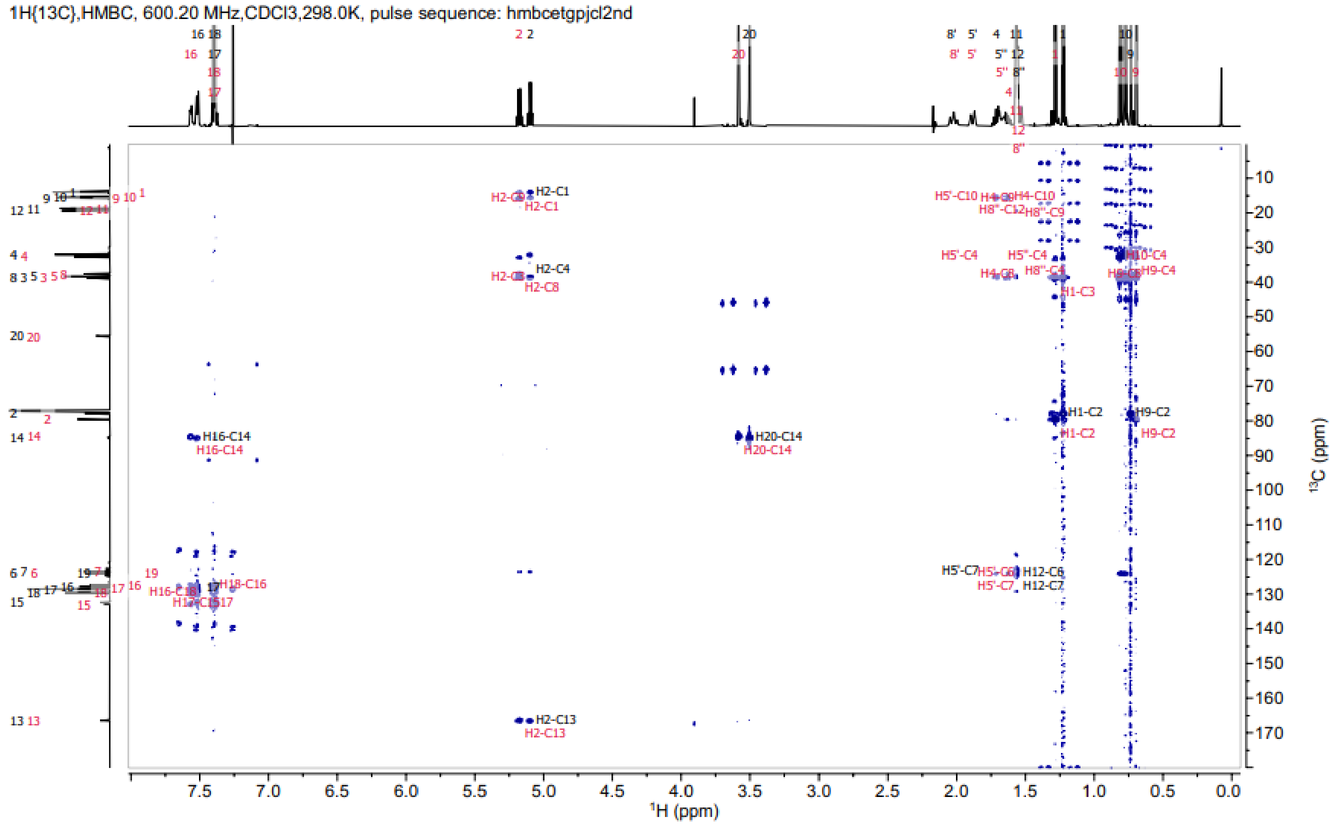

Figure S101. HR–HMBC spectrum of compound **(*R*)-13r**. J_{CH} coupling is visible in the indirect dimension and theoretically scaled by 20.

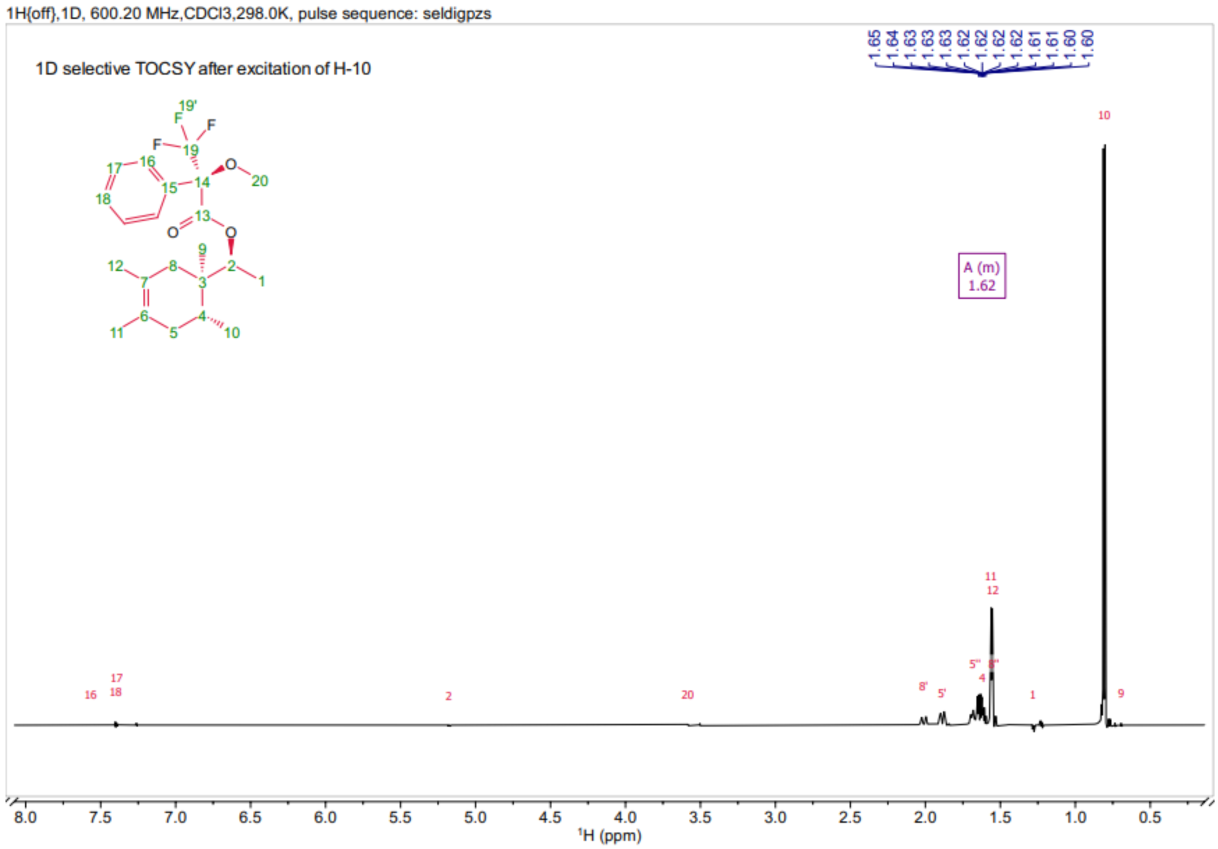

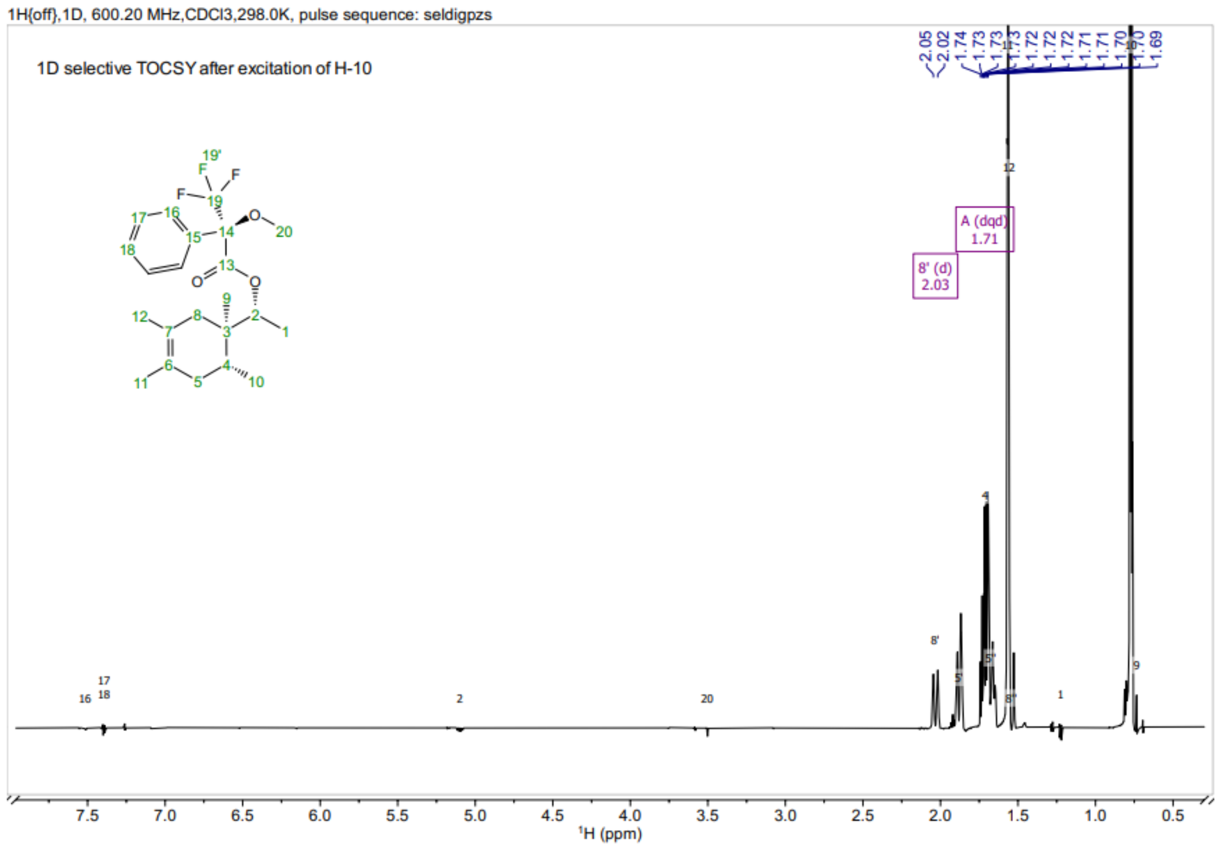

Figure S102. 1D selective TOCSY spectrum after excitation of H-10.

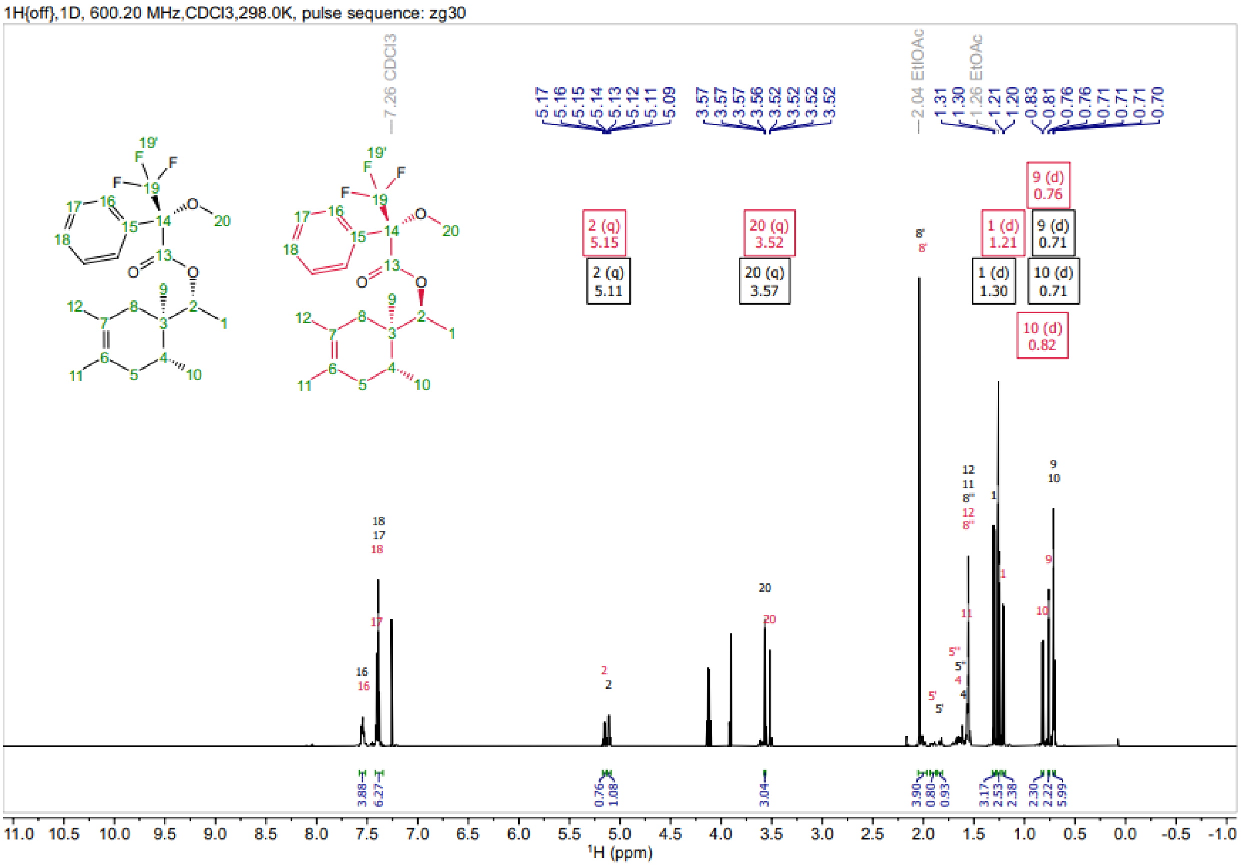
1H{off},1D, 600.20 MHz,CDCl3,298.0K, pulse sequence: zg30
7.26 CDCl3
2.04 EtOAc
1.26 EtOAc
2 (q) 5.15
2 (q) 5.11
20 (q) 3.52
20 (q) 3.57
1 (d) 1.21
1 (d) 1.30
9 (d) 0.76
9 (d) 0.71
10 (d) 0.71
10 (d) 0.82
^{1}H (ppm)

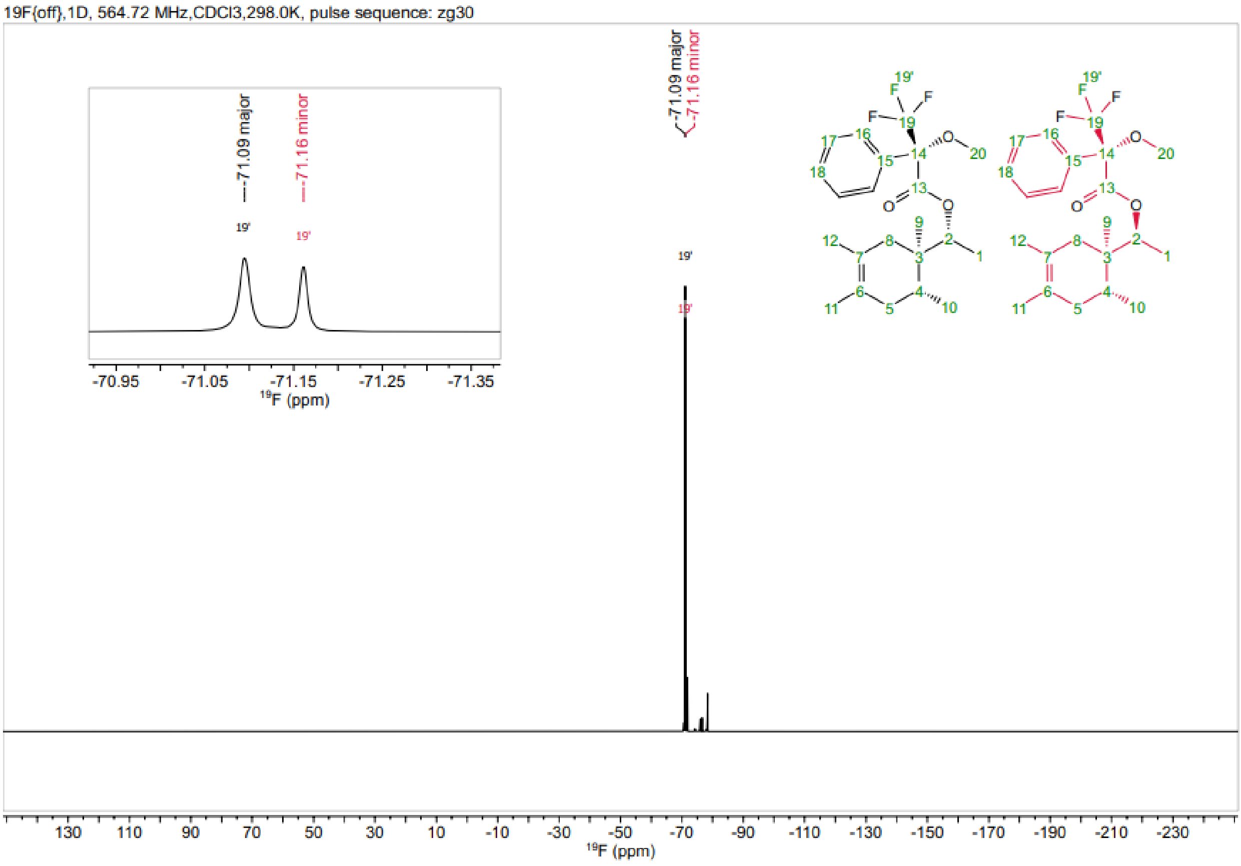
19F{off},1D, 564.72 MHz,CDCl3,298.0K, pulse sequence: zg30
-71.09 major
-71.16 minor
^{19}F (ppm)

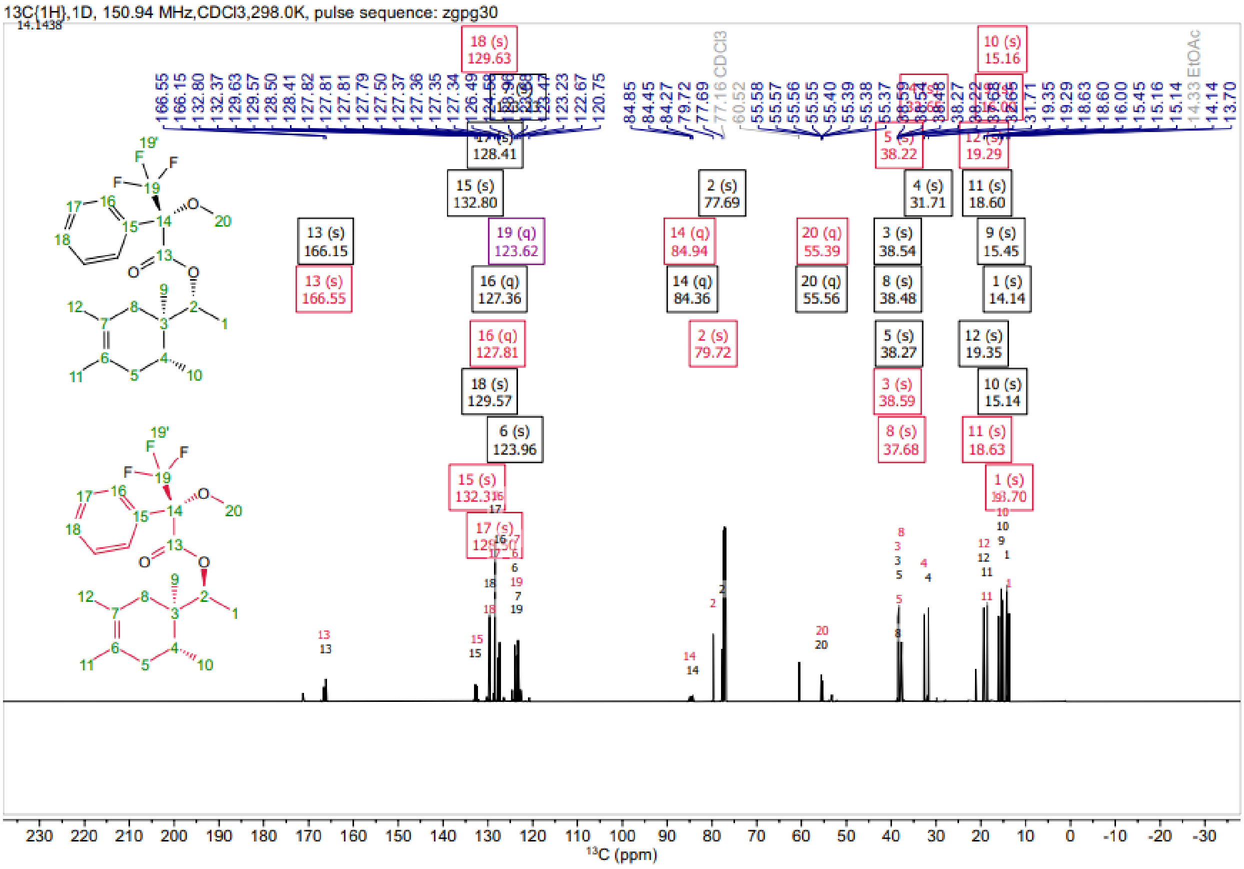

Figure S103. ^{1}H NMR, ^{19}F NMR and ^{13}C NMR spectra of compound **(*S*)-13r**.

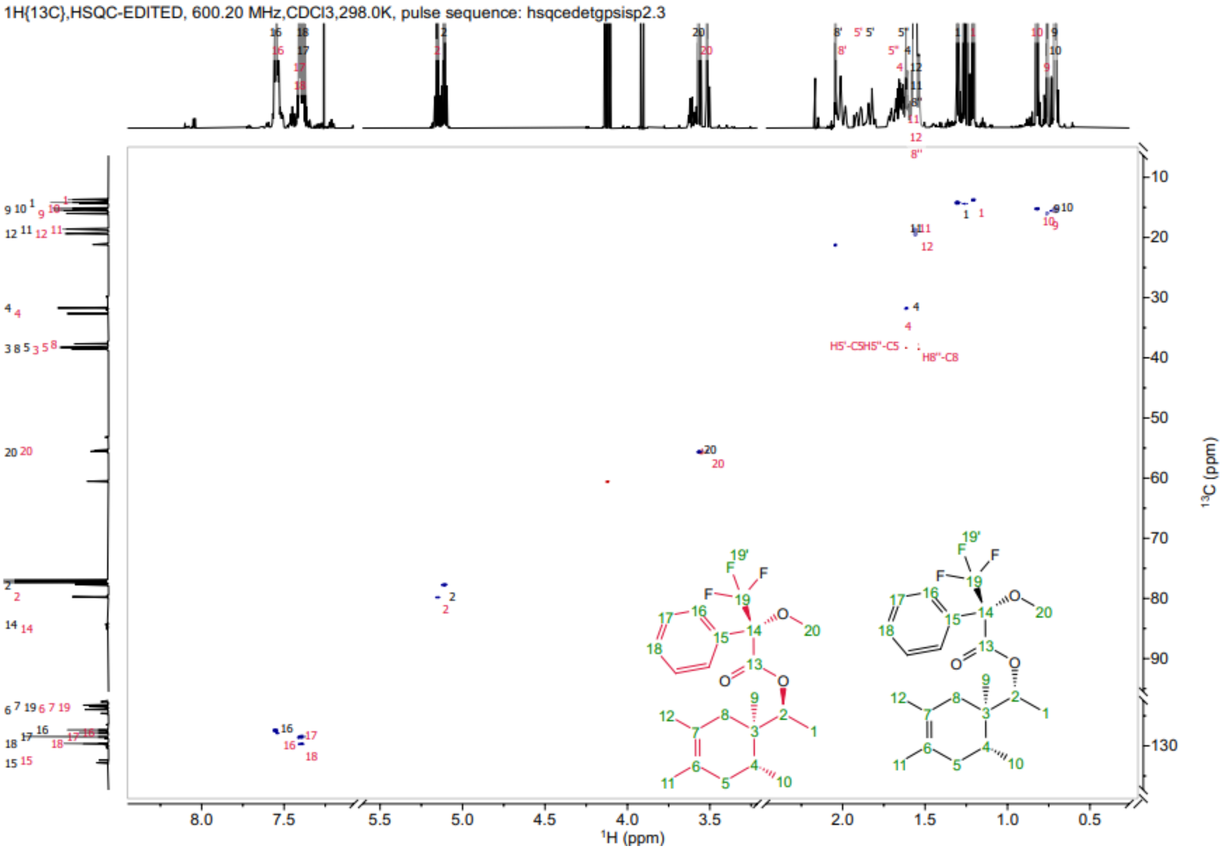

Figure S104. ^{1}H NMR – ^{13}C NMR HSQC spectrum of compound **(*S*)-13r**.

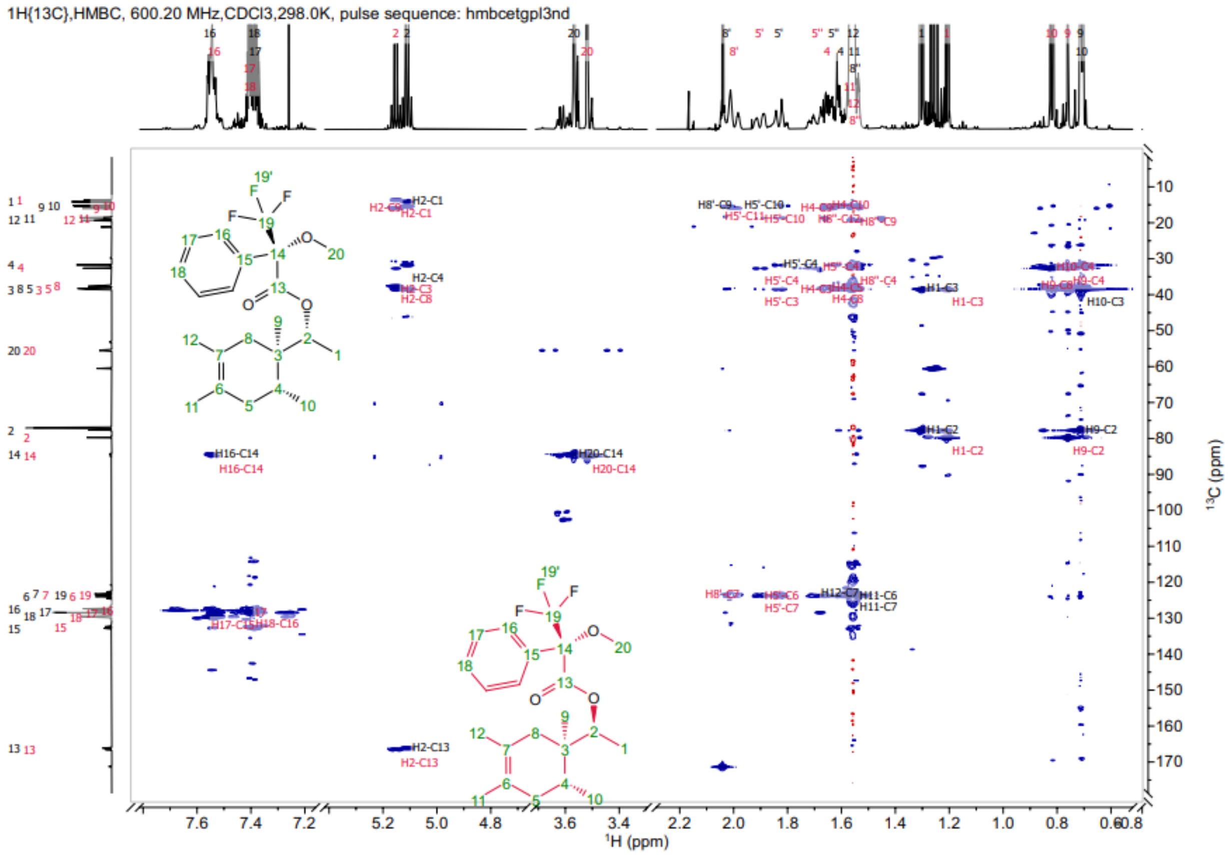

Figure S105. ^{1}H NMR – ^{13}C NMR HMBC spectrum of compound **(*S*)-13r**.

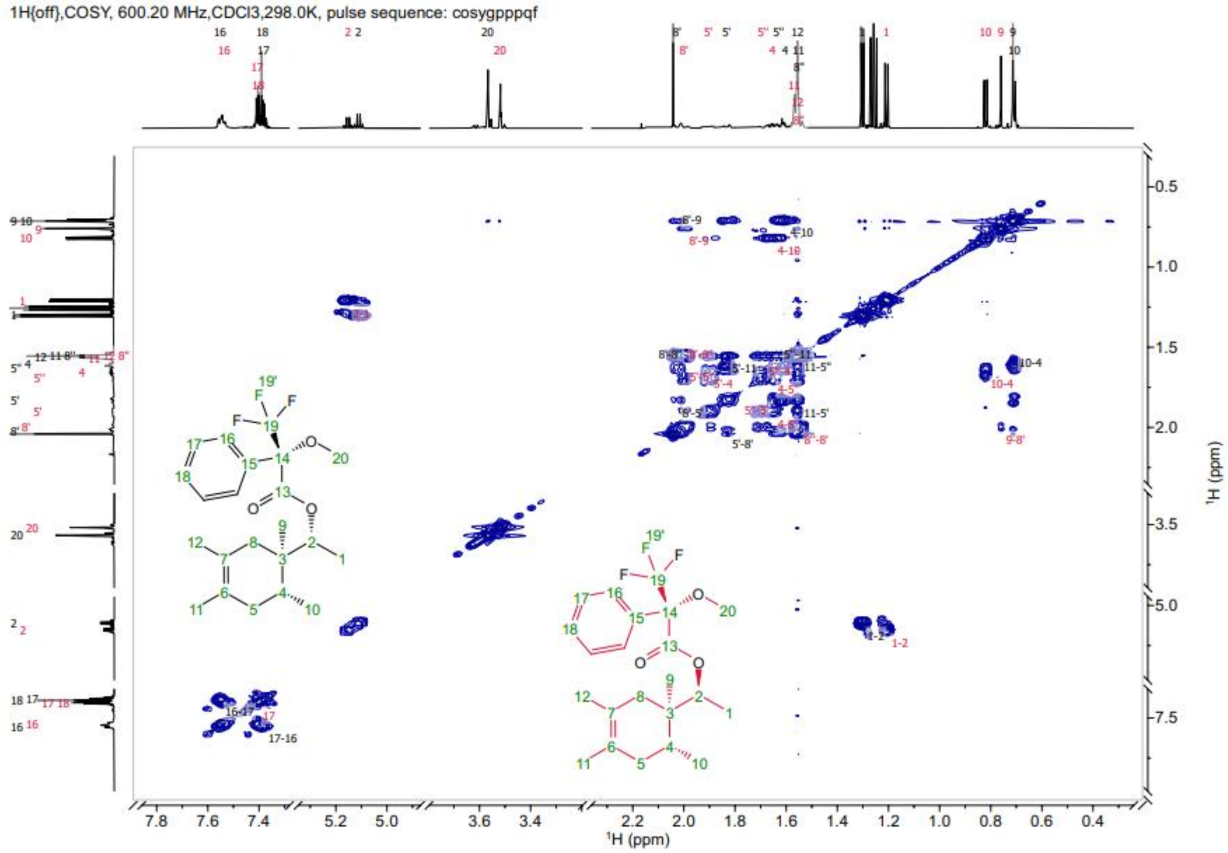

Figure S106. ^{1}H NMR COSY spectrum of compound **(*S*)-13r**.

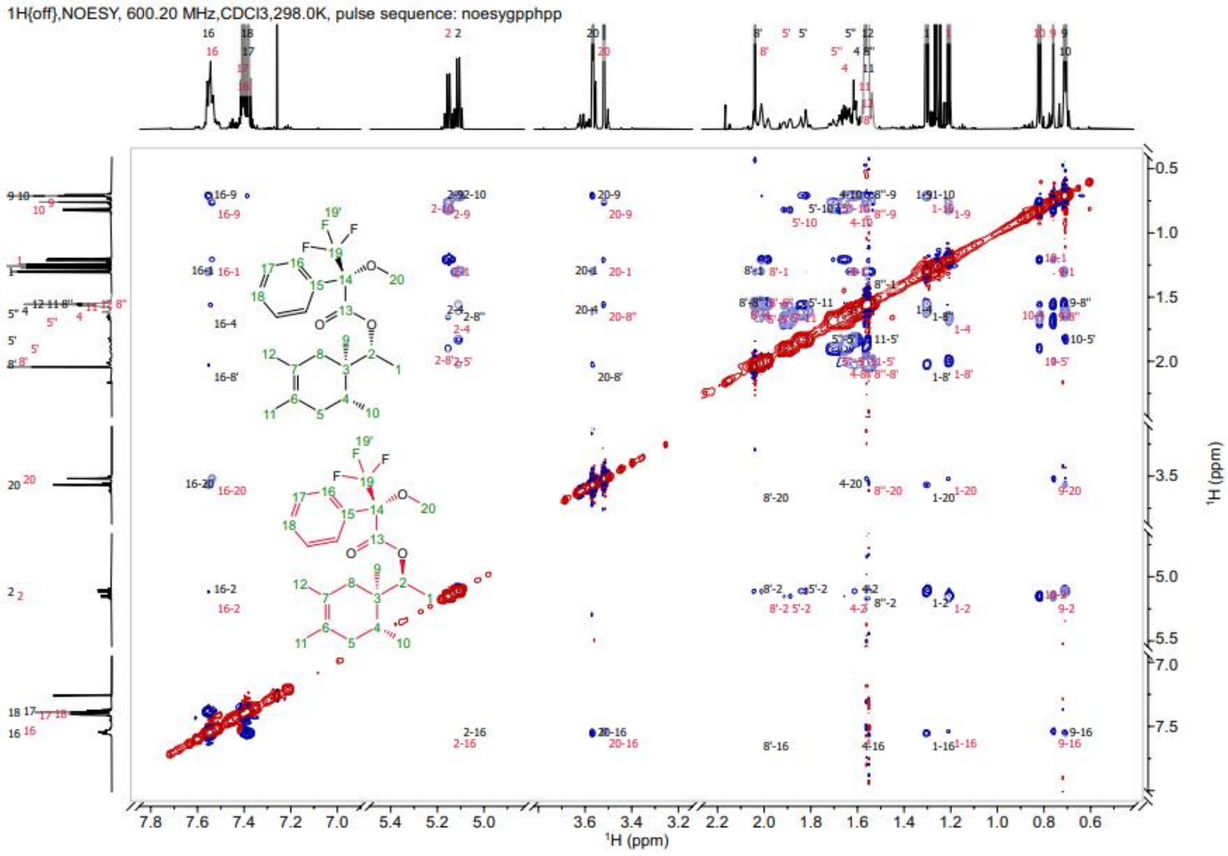

Figure S107. NOESY spectrum of compound **(*S*)-13r**.

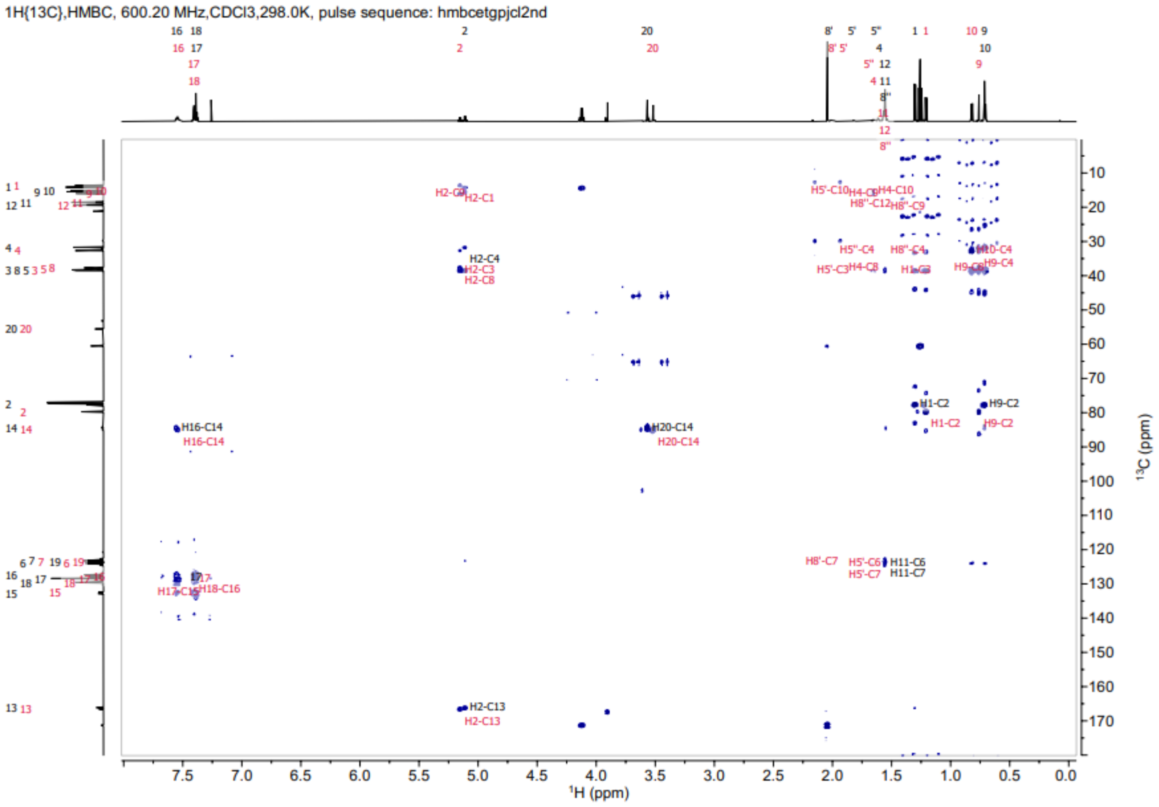

Figure S108. HR-HMBC spectrum of compound **(*S*)-13r**. J_{CH} coupling is visible in the indirect dimension and theoretically scaled by 20.

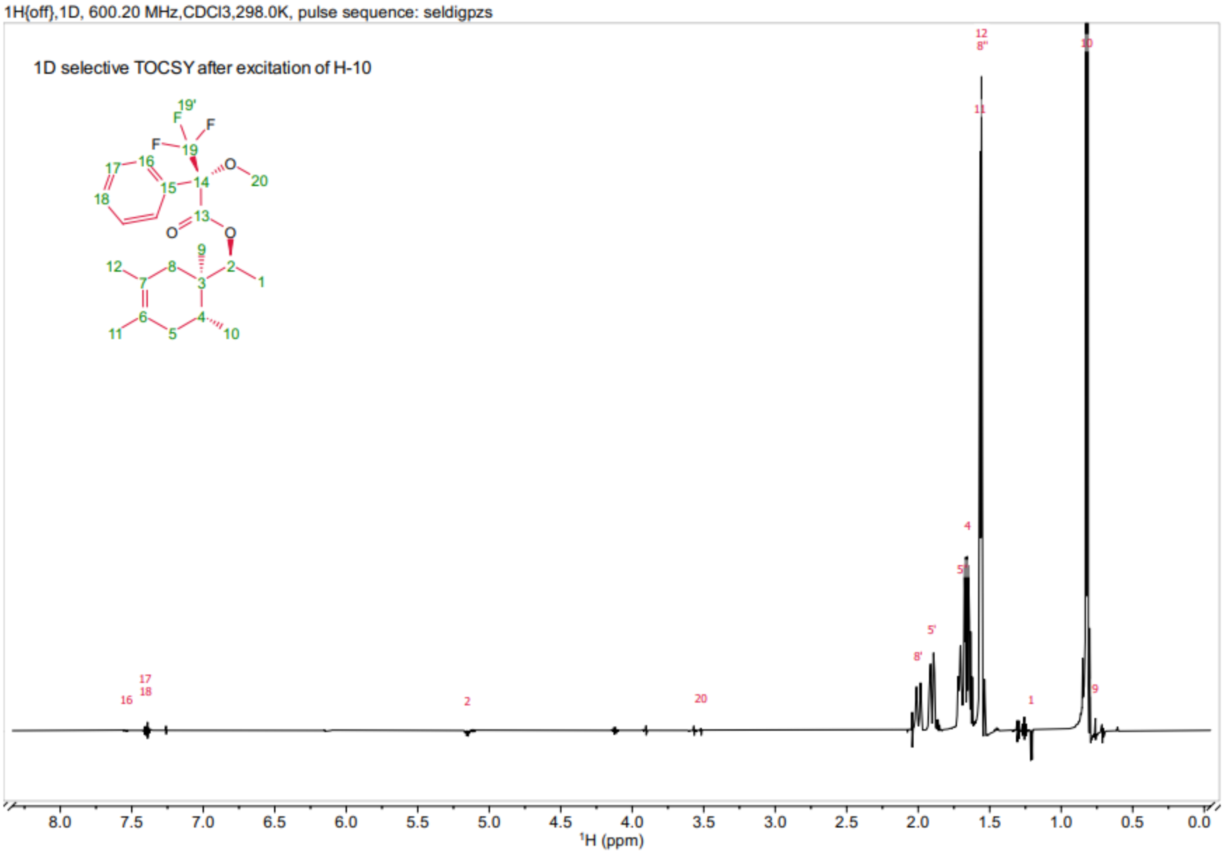

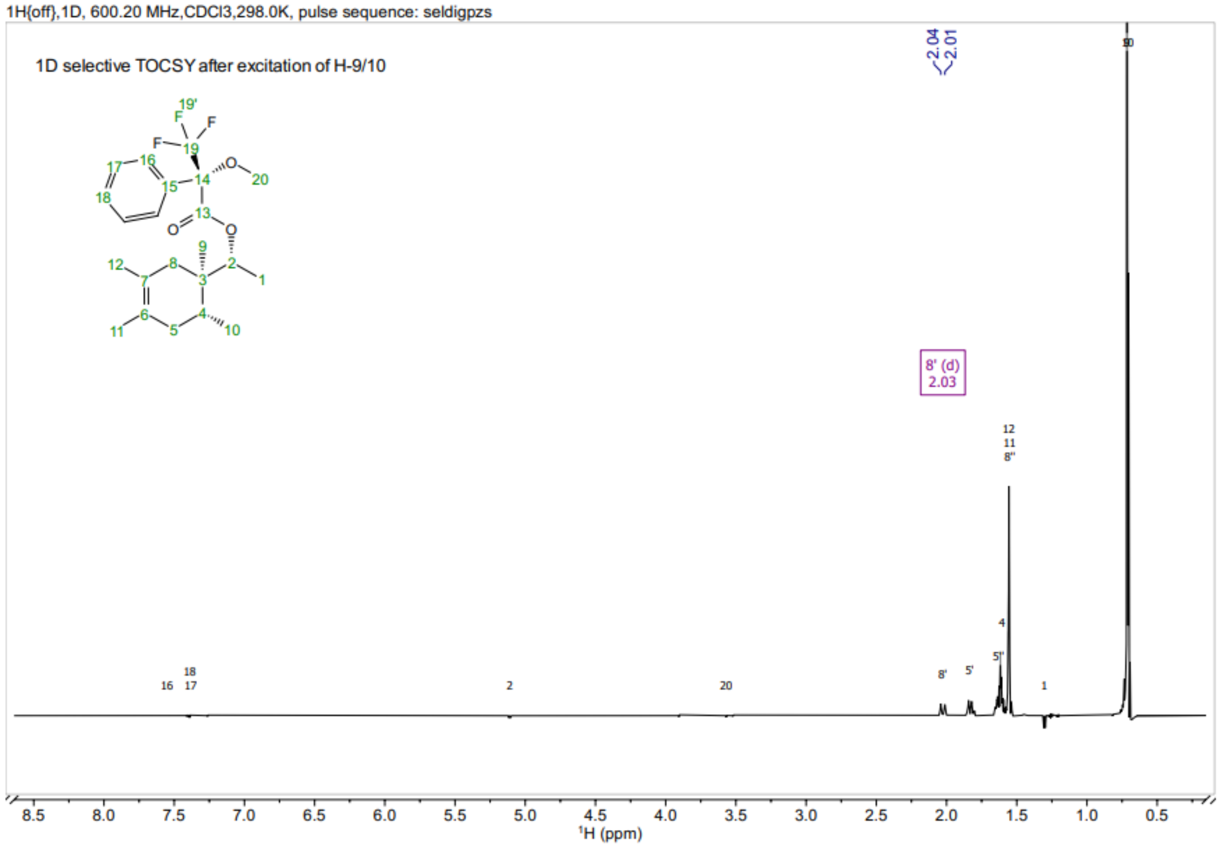

Figure S109. 1D selective TOCSY spectrum after excitation of H-10.

18.8. Osmate esters

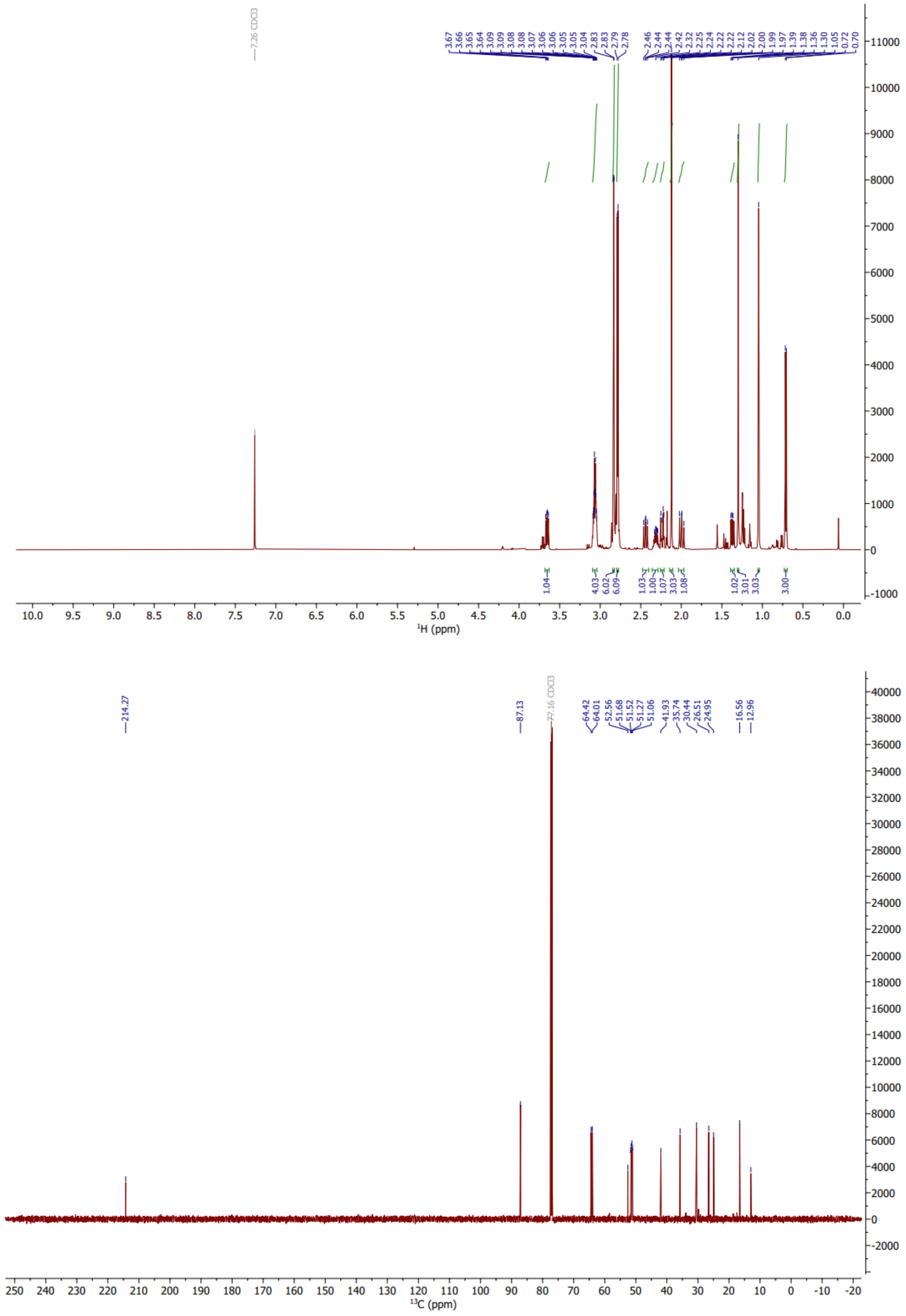

Figure S110. ^{1}H NMR and ^{13}C NMR spectra of compound **4qa**.

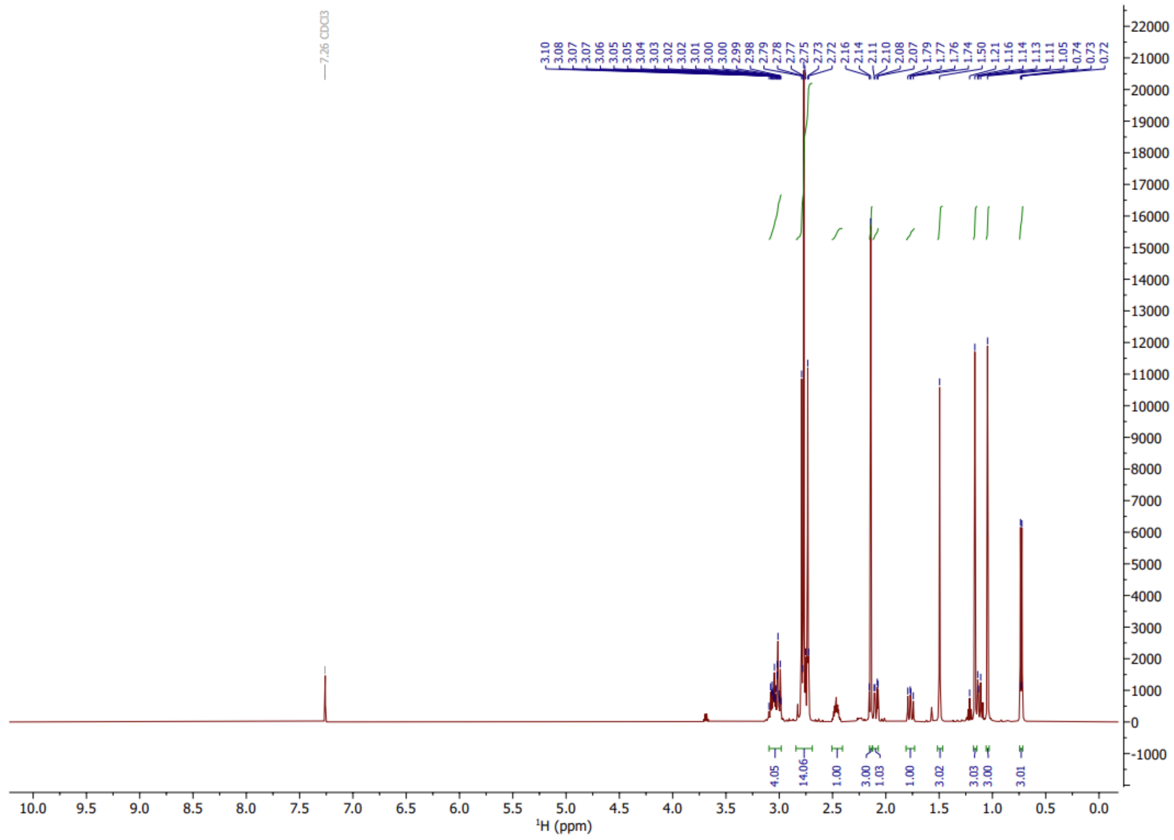

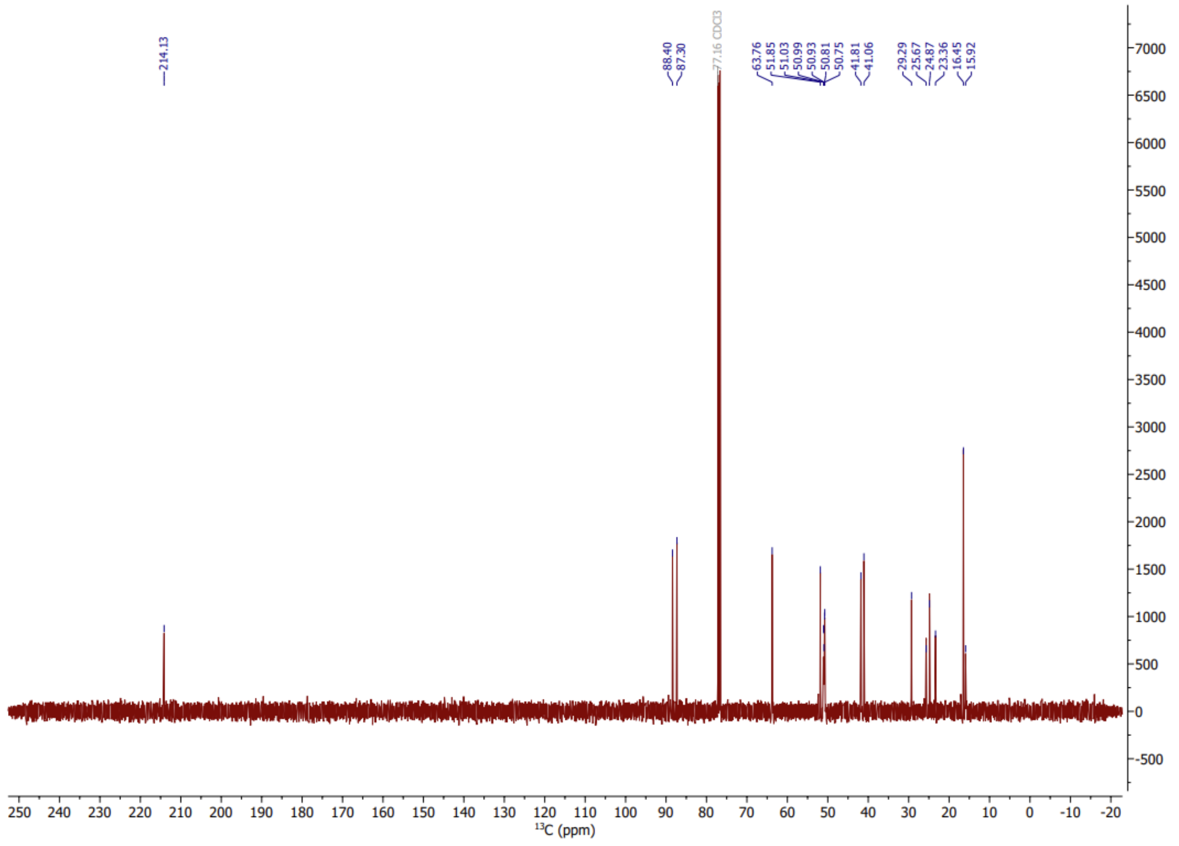

Figure S111. ^{1}H NMR and ^{13}C NMR spectra of compound **4ra**.

19. HPLC and GC traces

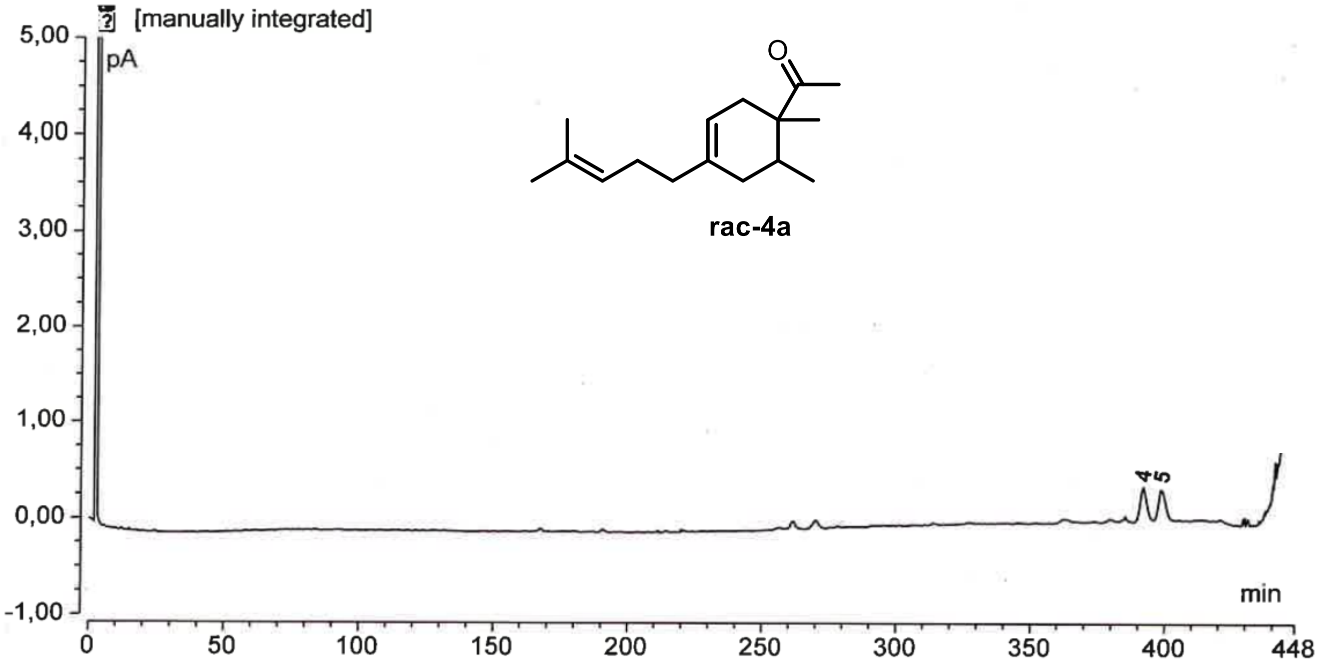

No.	Ret.Time min	Rel.Area %	Peak Name
4	391,67	49,82	**
5	398,57	50,18	**

Instrument parameters:

Column:	30,0 m	BGB-176/BGB-15 0,25/0,25df G/618	
Temperature:	220 / 100, 420 min iso 6/min 240, 5 min iso / 350		
Gas:	0,50 bar	H2	
Sample size:	1,0 µL	Split ratio:	10 : 1

Figure S112. GC traces of racemic and compound **4a**.

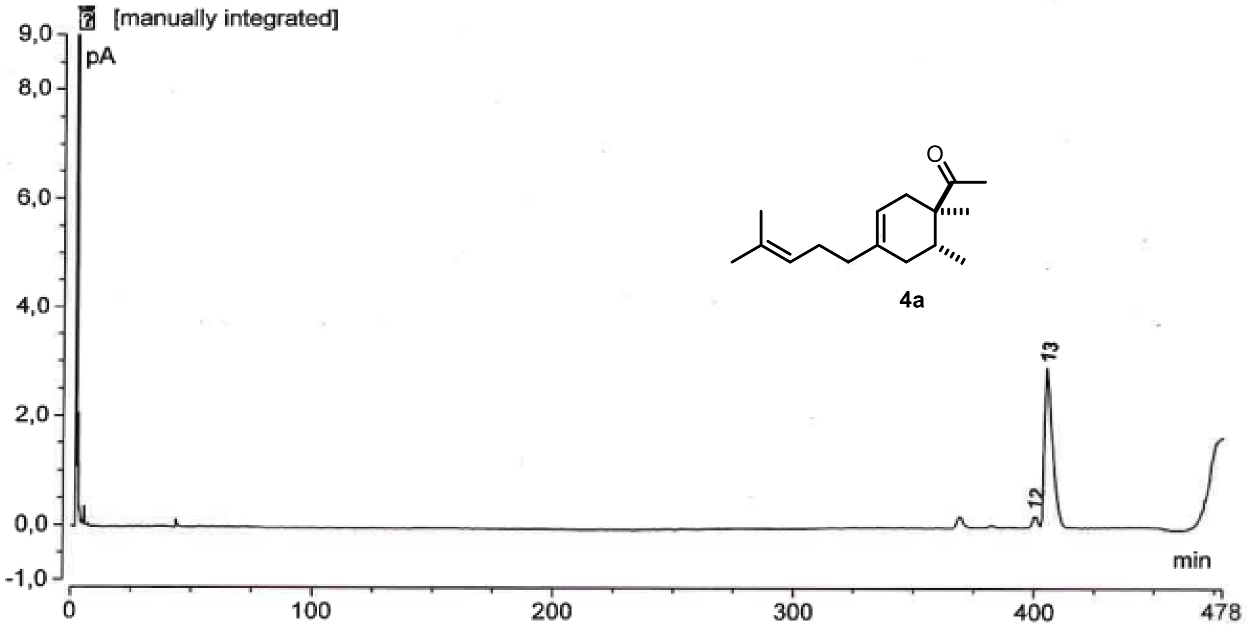

No.	Ret.Time min	Rel.Area %	Peak Name
12	400,39	4,49	..
13	404,76	95,51	..

Instrument parameters:

Column:	30,0 m	BGB-176/BGB-15 0,25/0,25df G/618
Temperature:	220 / 100, 450 min iso 6/min 240, 5 min iso / 350	
Gas:	0,50 bar	H2
Sample size:	0,2 µL	Split ratio: 10 : 1

Figure S113. GC traces of enantioenriched and compound **4a**.

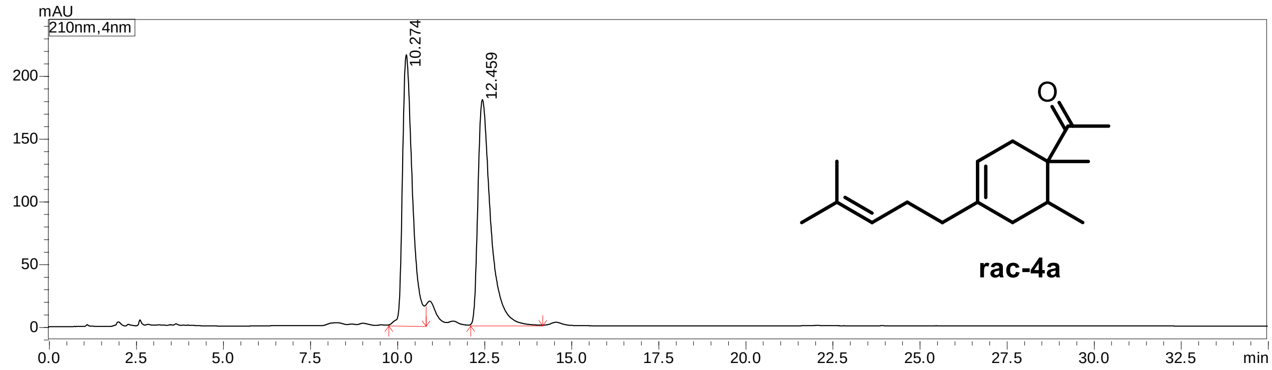

Peak:	Ret. Time [min]	Area [%]	
1	10.27	49.547	E1
2	12.46	50.453	E2

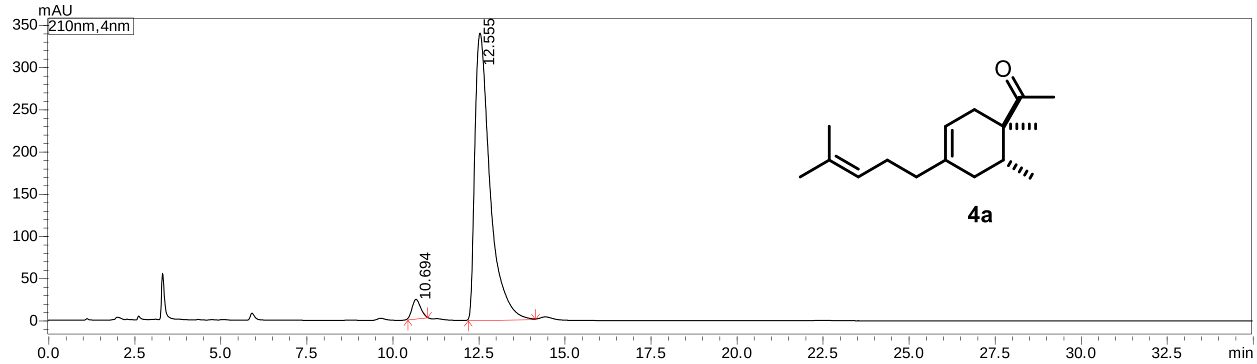

Peak:	Ret. Time [min]	Area [%]	
1	10.69	2.883	E1
2	12.56	97.117	E2

Figure S114. HPLC traces of racemic and enantioenriched compound **4a** (on racemate the corresponding regioisomer is visible).

Conditions: column: AD-3R, eluent (isocratic) 40:60 (v/v) as eluent, flow 1 mL/min, column temperature: 25 °C.

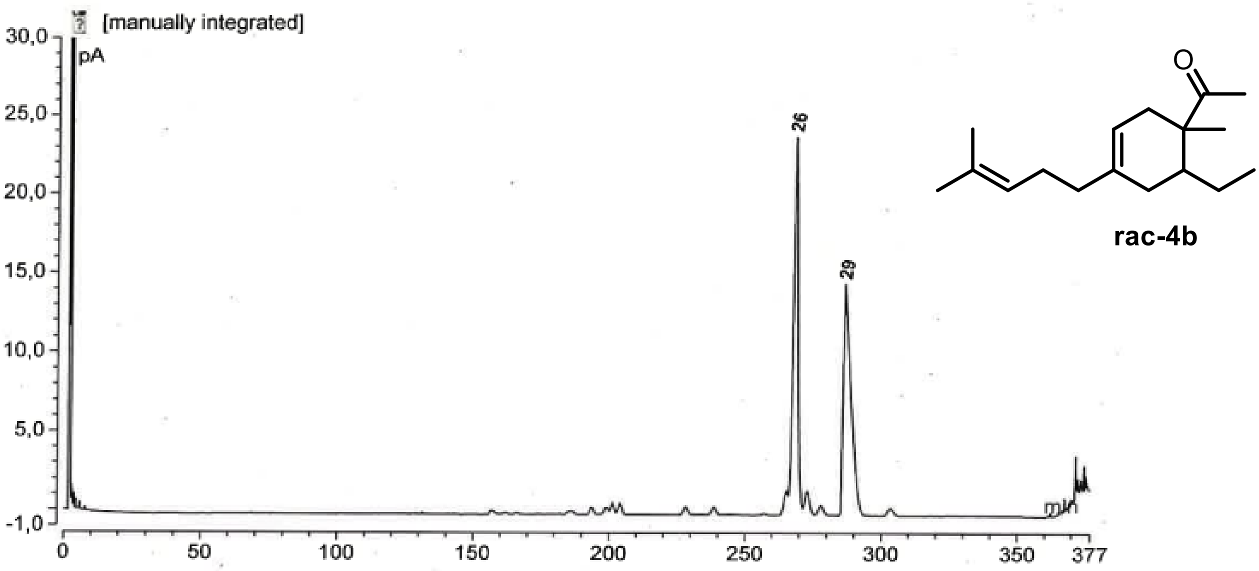

rac-4b

No.	Ret.Time min	Rel.Area %	Peak Name
26	268,12	50,30	.
29	286,30	49,70	.

Instrument parameters:

Column:	30,0 m	BGB-176/BGB-15 0,25/0,25df G/618	
Temperature:	220 / 110, 350 min iso 6/min 240, 5 min iso / 350		
Gas:	0,60 bar	H2	
Sample size:	1,0 µL	Split ratio:	10 : 1

Figure S115. GC traces of racemic compound **4b** (the corresponding regioisomer is visible).

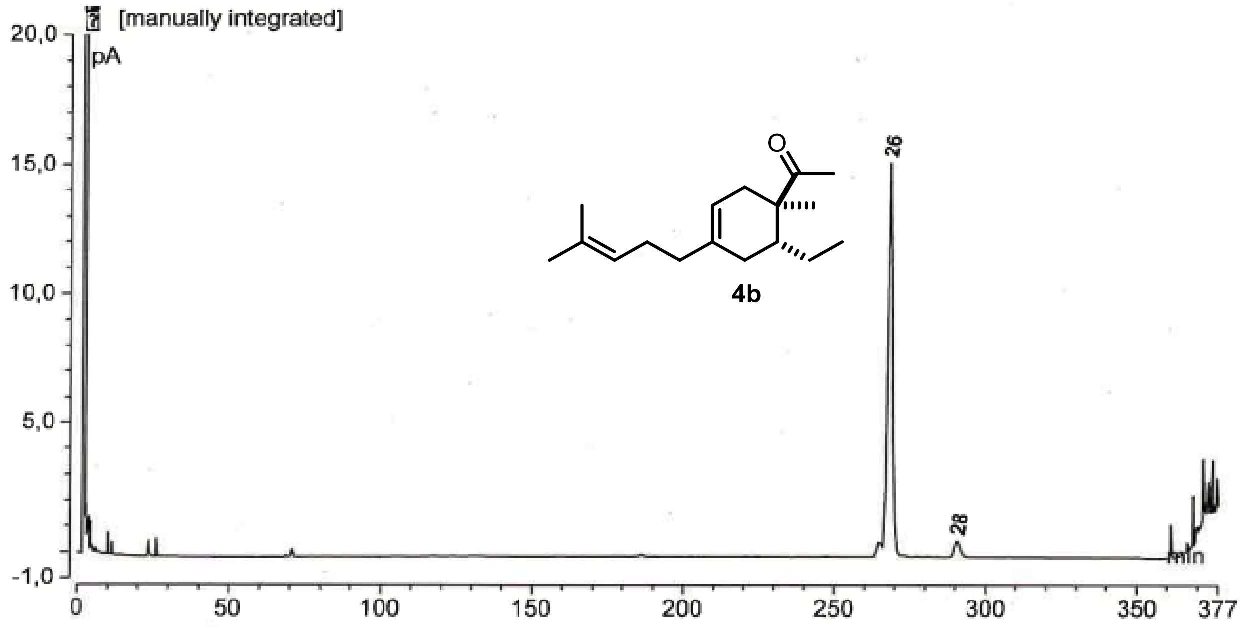

No.	Ret.Time min	Rel.Area %	Peak Name
26	267,94	95,85	.
28	290,61	4,15	.

Instrument parameters:

Column:	30,0 m	BGB-176/BGB-15 0,25/0,25df G/618
Temperature:	220 / 110, 350 min iso 6/min 240, 5 min iso / 350	
Gas:	0,60 bar	H2
Sample size:	1,0 µL	Split ratio: 10 : 1

Figure S116. GC traces of enantioenriched compound **4b**.

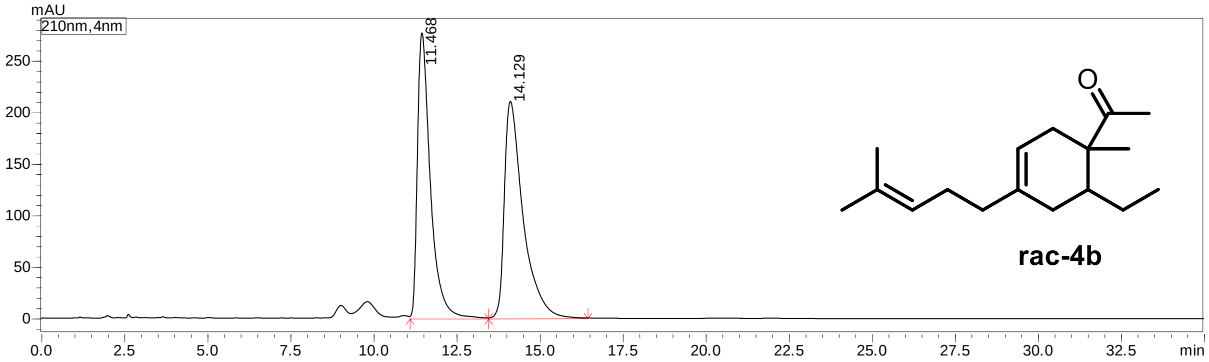

Peak:	Ret. Time [min]	Area [%]	
1	11.47	47.681	E1
2	14.13	52.319	E2

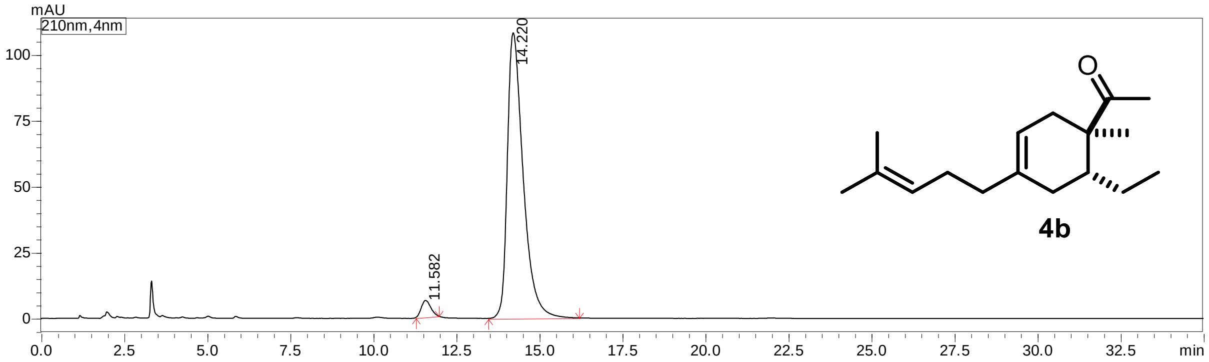

Peak:	Ret. Time [min]	Area [%]	
1	11.58	3.108	E1
2	14.22	96.892	E2

Figure S117. HPLC traces of racemic and enantioenriched compound **4b** (on racemate the corresponding regioisomer is visible)

Conditions: column: AD-3R, eluent (isocratic): water:acetonitrile 40:60 (v/v) as eluent, flow 1 mL/min, column temperature: 25 °C.

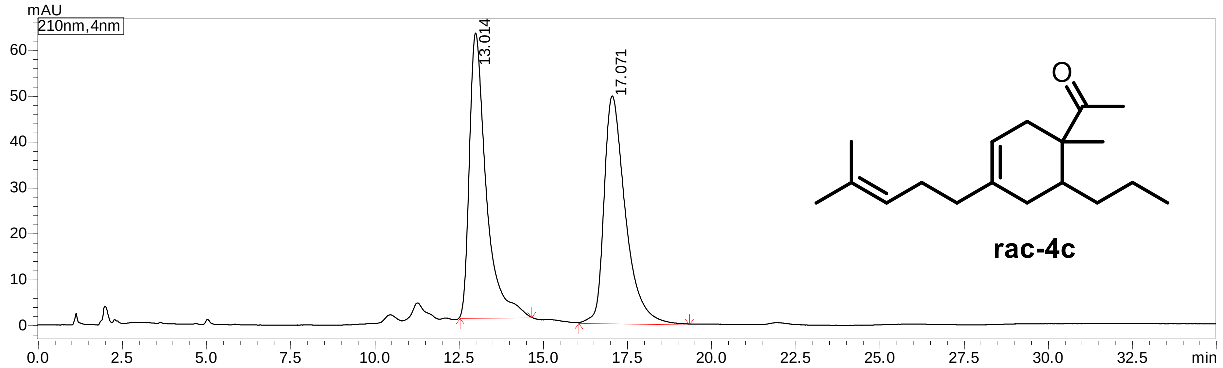

Peak#	Ret. Time	Area%	
1	13.01	49.648	E1
2	17.07	50.352	E2
Total		100.000	

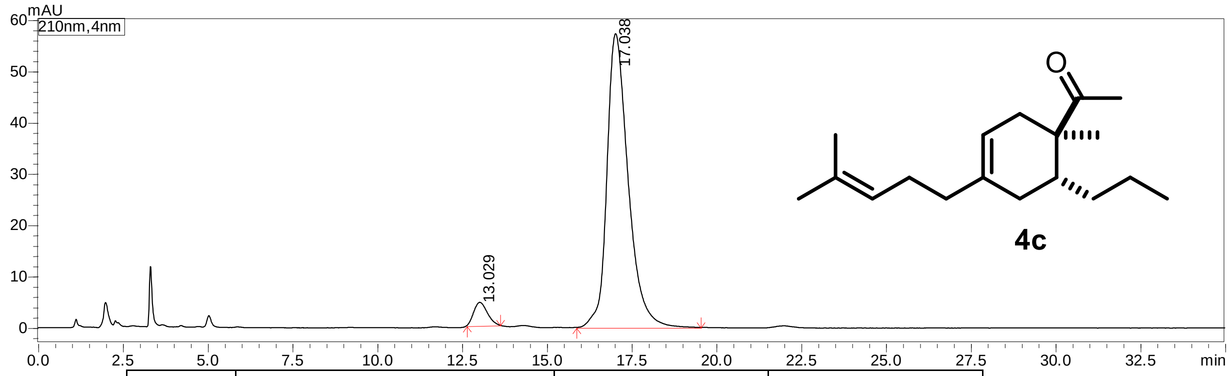

Peak#	Ret. Time	Area%	
1	13.03	4.607	E1
2	17.04	95.393	E2
Total		100.000	

Figure S118. HPLC traces of racemic and enantioenriched compound **4c** (on racemate the corresponding regioisomer is visible).

Conditions: column: AD-3R, eluent (isocratic): water:acetonitrile 40:60 (v/v) as eluent, flow 1 mL/min, column temperature: 25 °C.

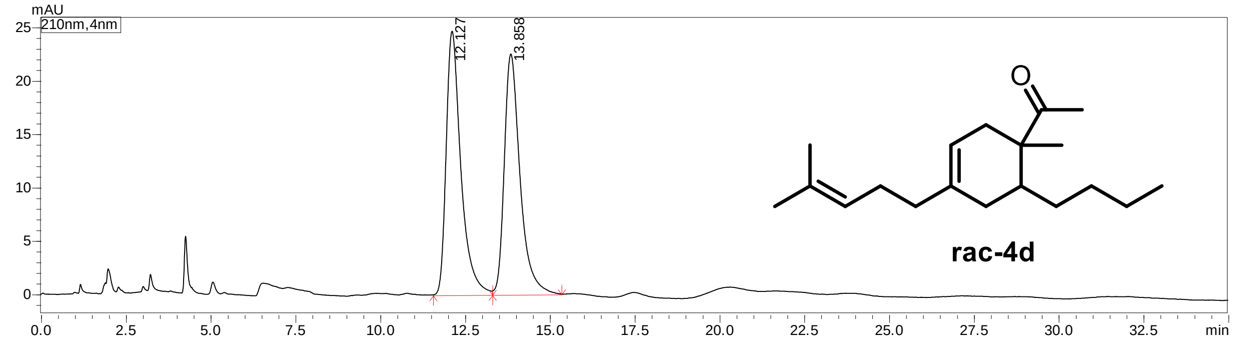

Peak#	Ret. Time	Area%	
1	12.13	50.576	E1
2	13.86	49.424	E2
Total		100.000	

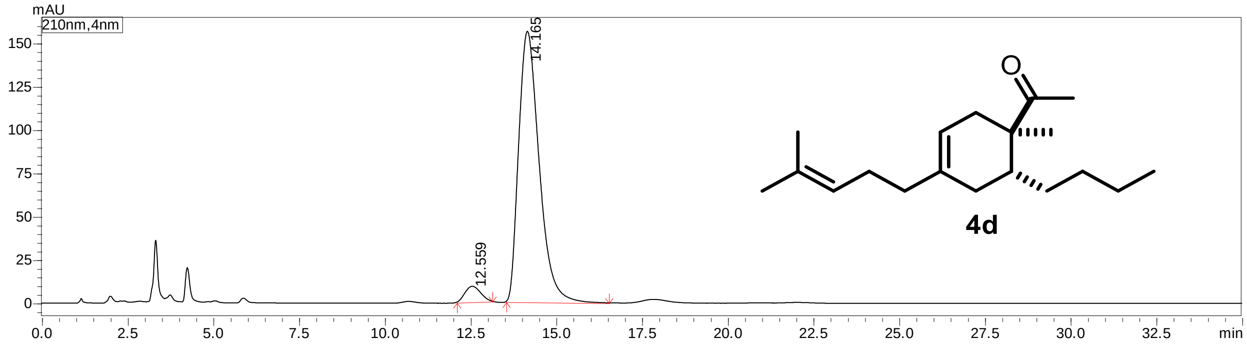

Entry	Ret. Time (Min.)	Area (%)	
1	12.56	4.101	E1
2	14.17	95.899	E2
Total		100.000	

Figure S119. HPLC traces of racemic and enantioenriched compound **4d**.

Conditions: column AD-3R, eluent (isocratic): water:acetonitrile 40:60 (v/v) as eluent, flow 1 mL/min, column temperature: 25 °C.

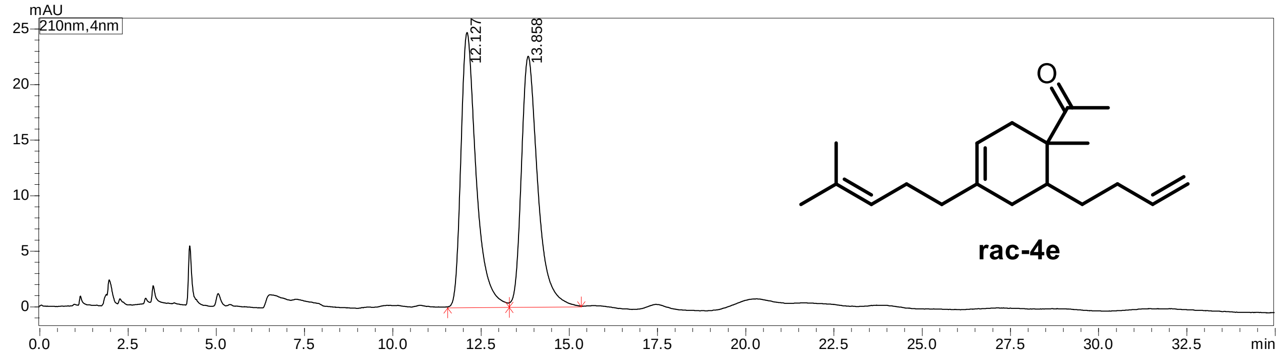

Peak#	Ret. Time	Area%	
1	12.13	50.576	E1
2	13.86	49.424	E2
Total		100.000	

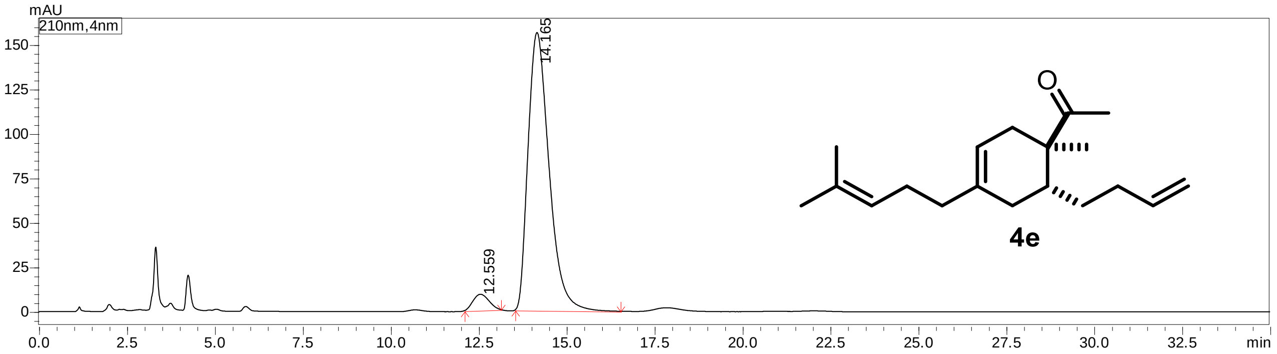

Entry	Ret. Time (Min.)	Area (%)	
1	12.56	4.101	E1
2	14.17	95.899	E2
Total		100.000	

Figure S120. HPLC traces of racemic and enantioenriched compound **4e**.

Conditions: column AD-3R, eluent (isocratic): water:acetonitrile 40:60 (v/v) as eluent, flow 1 mL/min, column temperature: 25 °C.

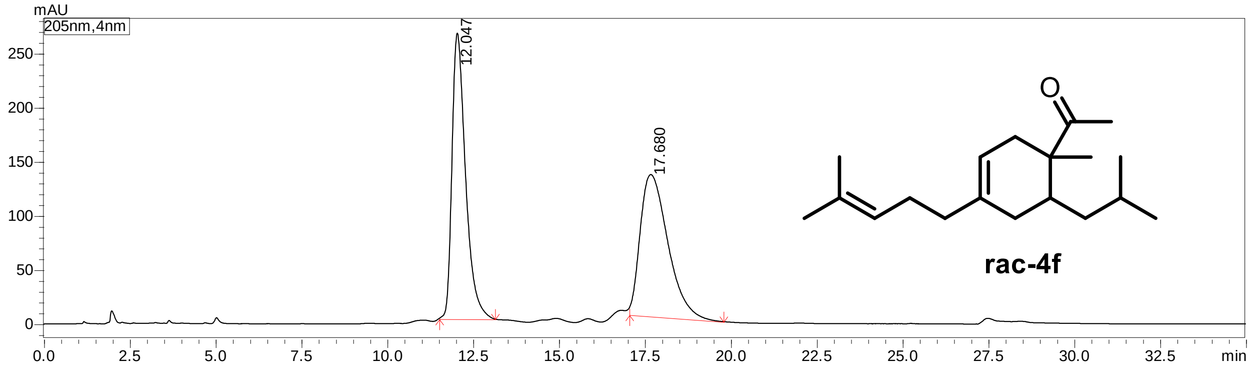

Peak#	Ret. Time	Area%	
1	12.05	49.680	E1
2	17.68	50.320	E2
Total		100.000	

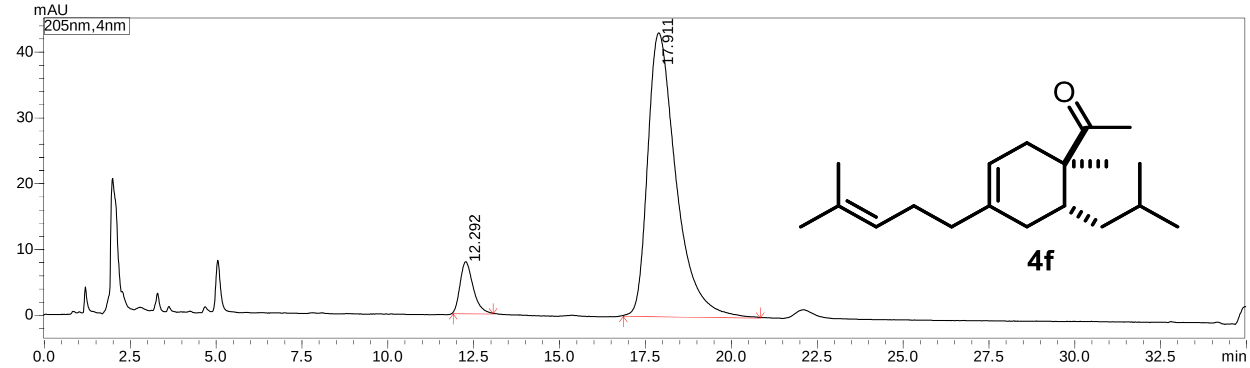

Peak#	Ret. Time	Area%	
1	12.29	7.340	E1
2	17.91	92.660	E2
Total		100.000	

Figure S121. HPLC traces of racemic and enantioenriched compound **4f** (on racemate the corresponding regioisomer is visible).

Conditions: column AD-3R, eluent (isocratic): water:acetonitrile 40:60 (v/v) as eluent, flow 1 mL/min, column temperature: 25 °C

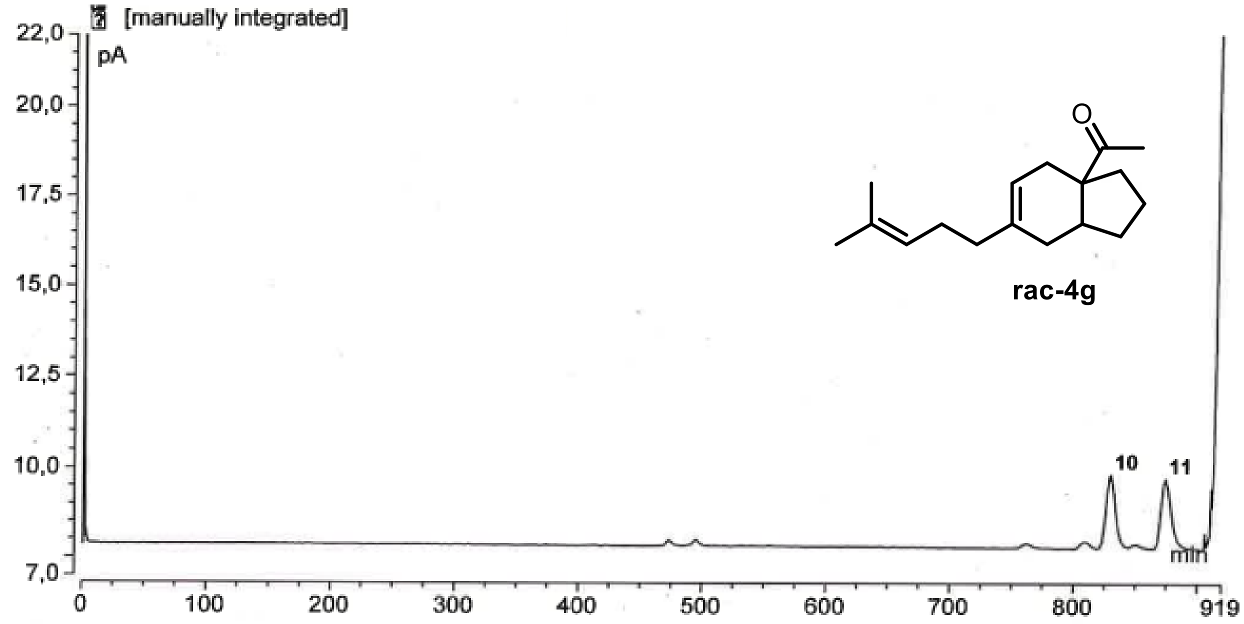

No.	Ret.Time min	Rel.Area %	Peak Name
10	829,89	49,33	.
11	874,16	50,67	.

Instrument parameters:

Column:	25,0 m	Lipodex-E 0,25/?df G/645
Temperature:	220 / 90, 900 min iso 8/min 220, 3 min iso / 350	
Gas:	0,50 bar	Hydrogen
Sample size:	1,0 µL	Split ratio: 10 : 1

Figure S122. GC traces of racemic compound **4g** (corresponding regioisomer is visible).

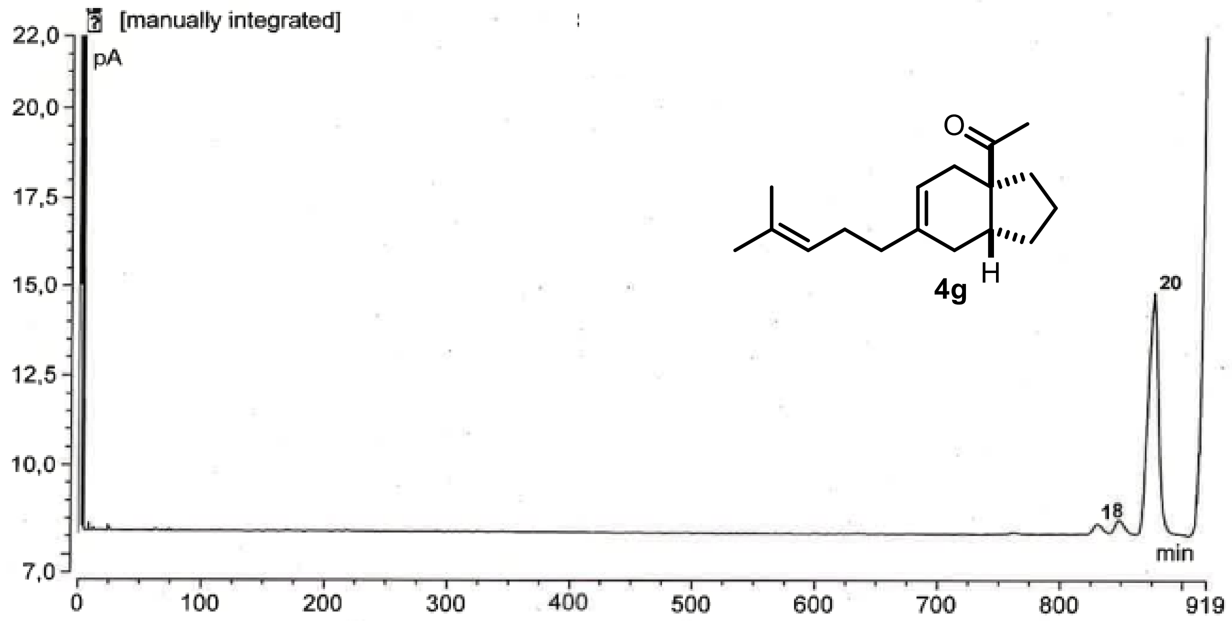

No.	Ret.Time min	Rel.Area %	Peak Name
18	829,98	4,03	.
20	875,69	95,97	.

Instrument parameters:

Column:	25,0 m	Lipodex-E 0,25/?df G/645		
Temperature:	220 / 90, 900 min iso 8/min 220, 3 min iso / 350			
Gas:	0,50 bar	Hydrogen		
Sample size:	1,0 µL	Split ratio:	10 : 1	

Figure S123. GC traces of enantioenriched compound **4g**.

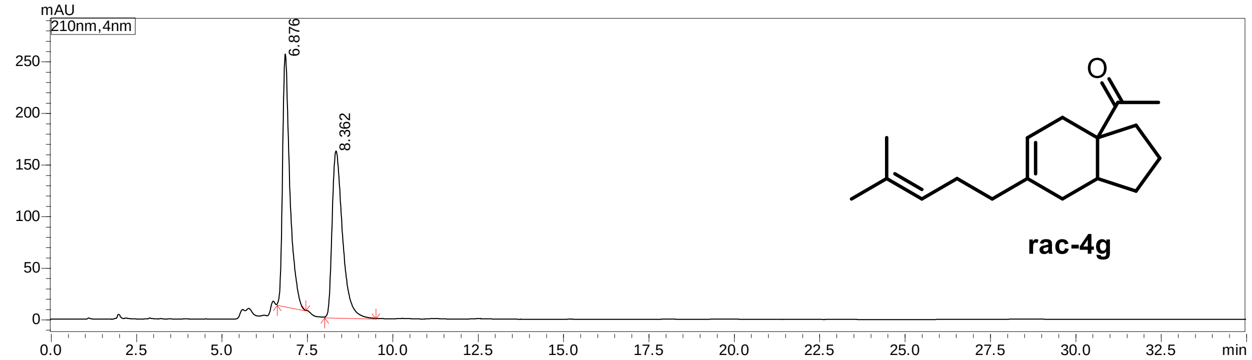

Peak#	Ret. Time	Area%	
1	6.88	51.466	E1
2	8.36	48.534	E2
Total		100.000	

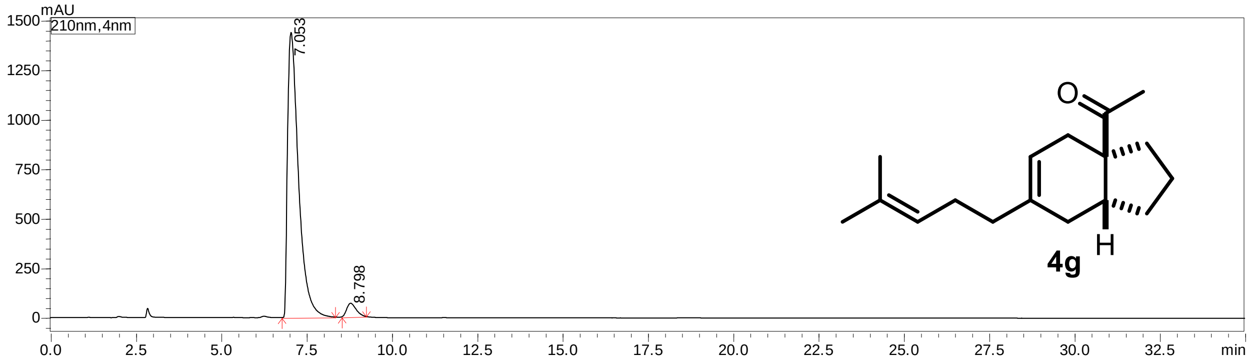

Peak#	Ret. Time	Area%	
1	7.05	96.486	E1
2	8.80	3.514	E2
Total		100.000	

Figure S124. HPLC traces of racemic and enantioenriched compound **4g** (on racemate the corresponding regioisomer is visible).

Conditions: column AD-3R, eluent (isocratic): water:acetonitrile 30:70 (v/v) as eluent, flow 1 mL/min, column temperature: 25 °C.

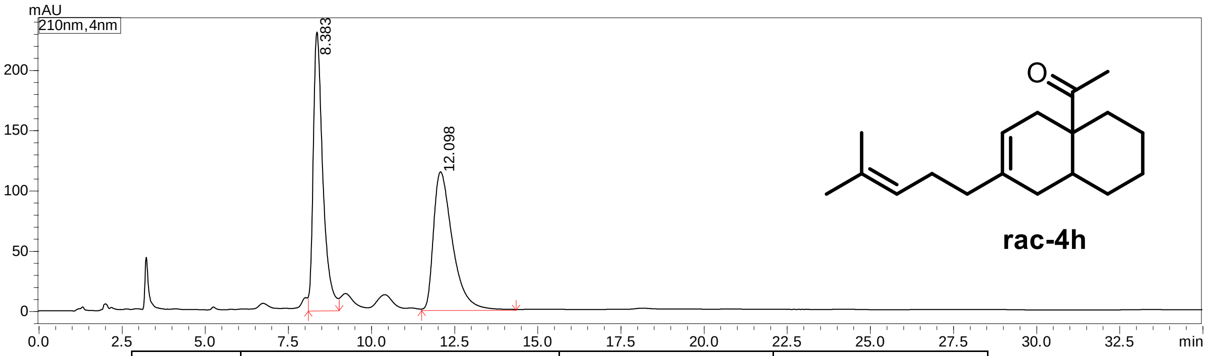

Entry	Ret. Time (Min.)	Area (%)	
1	8.38	50.880	E1
2	12.10	49.120	E2
Total		100.000	

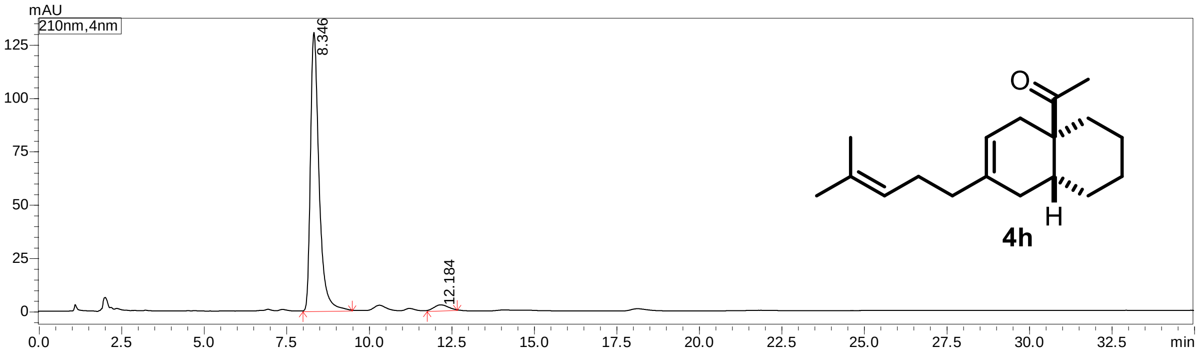

Entry	Ret. Time (Min.)	Area (%)	
1	8.35	97.370	E1
2	12.18	2.630	E2
Total		100.000	

Figure S125. HPLC traces of racemic and enantioenriched compound **4h** (corresponding regioisomer is visible).

Conditions: column AD-3R, eluent (isocratic): water:acetonitrile 30:70 (v/v) as eluent, flow 1 mL/min, column temperature: 25 °C.

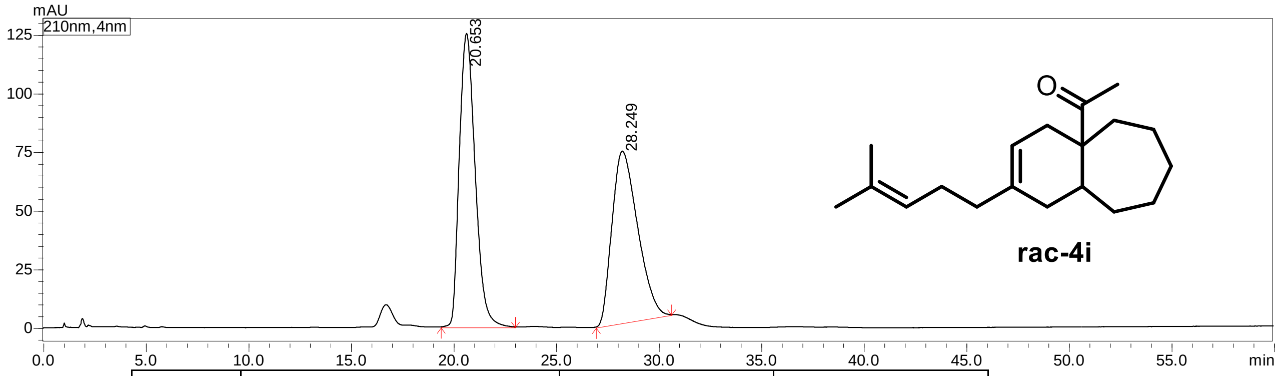

Entry	Ret. Time (Min.)	Area (%)	
1	20.65	50.830	E1
2	28.25	49.170	E2
Total		100.000	

HPLC Trace of Enantiopure (4i)

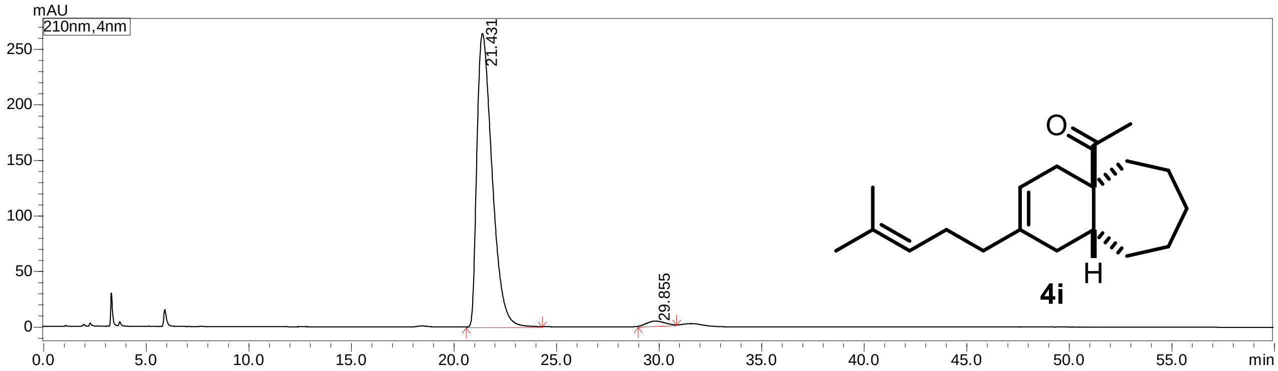

Entry	Ret. Time (Min.)	Area (%)	
1	21.43	98.270	E1
2	29.86	1.730	E2
Total		100.000	

Figure S126. HPLC traces of racemic and enantioenriched compound **4i** (on racemate the corresponding regioisomer is visible).

Conditions: column AD-3R, eluent (isocratic): water:acetonitrile 40:60 (v/v) as eluent, flow 1 mL/min, column temperature: 25 °C.

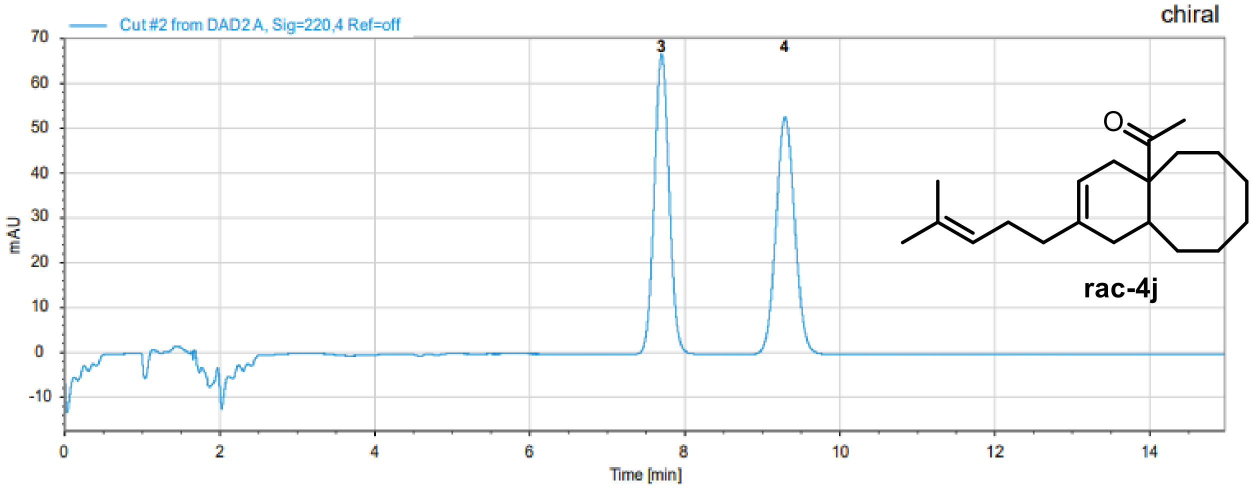

Entry	Ret. Time (Min.)	Area (%)	
1	7.71	50.016	E1(3)
2	9.30	49.984	E2(4)
Total		100.000	

Figure S127. 2D-HPLC traces of racemic compound **4j**.

Conditions: 2D-RP-HPLC: 1D: column: 50 mm Eclipse PAH 1.8 µm 4.6 mm i.D. eluent (isocratic): acetonitrile:water 70:30, flow: 1 mL/min, column temperature 35 °C, chromatogram observed at 220 nm; 2D: column: Chiralcel OJ-3R 4.6 i.D., eluent (isocratic): methanol:water 85:15 (v/v), flow: 1 mL/min, column oven temperature: 25 °C, chromatograms were monitored at 220 nm; retention times (2D).

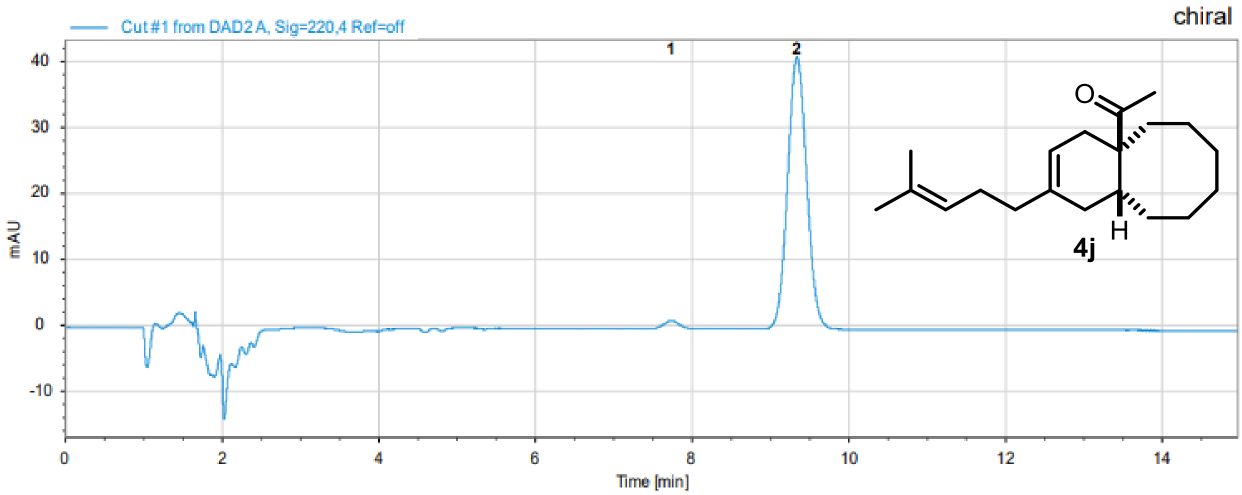

Entry	Ret. Time (Min.)	Area (%)	
1	7.74	2.159	E1
2	9.34	97.841	E2
Total		100.000	

Figure S128. 2D-HPLC traces of enantioenriched compound **4j**.

Conditions: 2D-RP-HPLC: 1D: column: 50 mm Eclipse PAH 1.8 µm 4.6 mm i.D. eluent (isocratic): acetonitrile:water 70:30, flow: 1 mL/min, column temperature 35 °C, chromatogram observed at 220 nm; 2D: column: Chiralcel OJ-3R 4.6 i.D., eluent (isocratic): methanol:water 85:15 (v/v), flow: 1 mL/min, column oven temperature: 25 °C, chromatograms were monitored at 220 nm; retention times (2D).

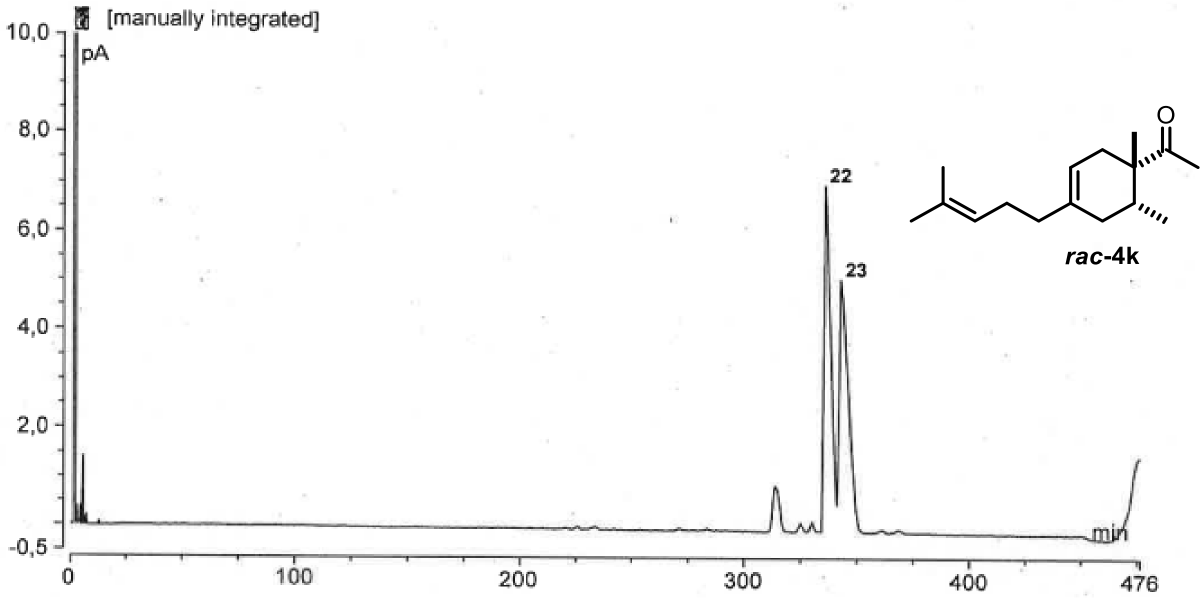

No.	Ret.Time min	Rel.Area %	Peak Name
22	336,02	49,89	.
23	342,92	50,11	.

Instrument parameters:

Column:	30,0 m	BGB-176/BGB-15 0,25/0,25df G/618
Temperature:	220 / 100, 450 min iso 6/min 240, 3 min iso / 350	
Gas:	0,60 bar	H2
Sample size:	0,2 µL	

Figure S129. GC traces of racemic compound **4k** (corresponding regioisomer is visible).

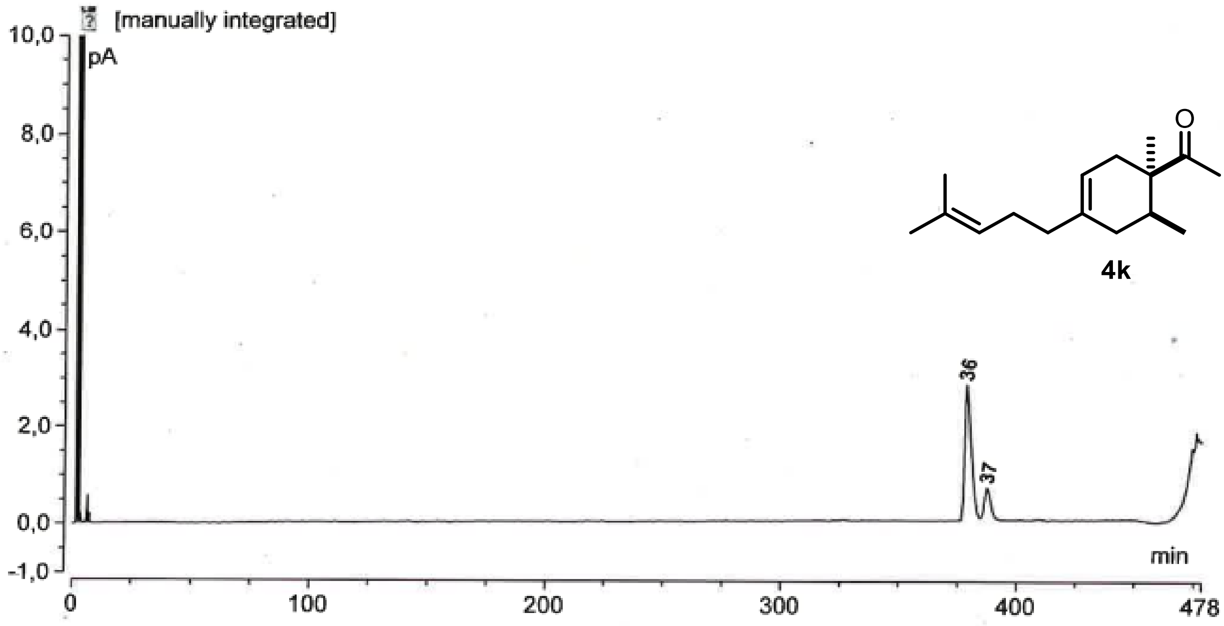

No.	Ret.Time min	Rel.Area %	Peak Name
36	378,47	80,41 .	
37	387,40	19,59 .	

Instrument parameters:

Column:	30,0 m	BGB-176/BGB-15 0,25/0,25df G/618		
Temperature:	220 / 100, 450 min iso 6/min 240, 5 min iso / 350			
Gas:	0,50 bar	H2		
Sample size:	0,2 µL	Split ratio:	80 : 1	

Figure S130. GC traces of enantioenriched compound **4k**.

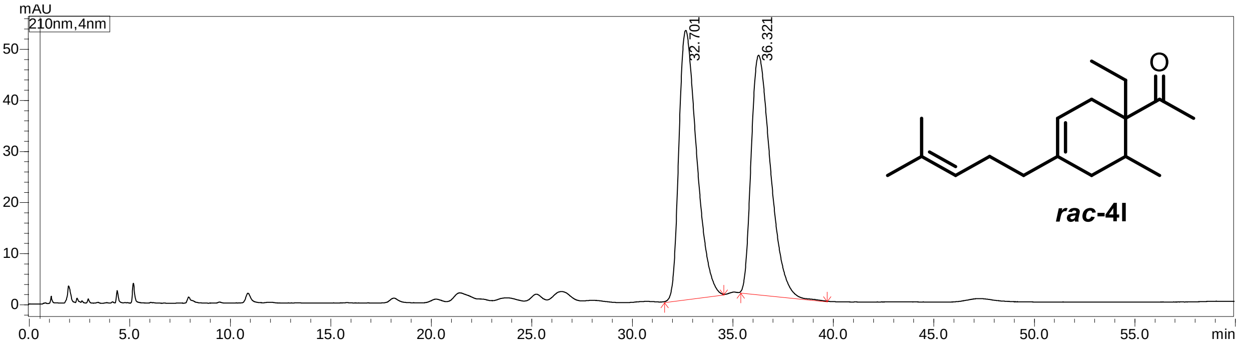

Entry	Ret. Time (Min.)	Area (%)	
1	32.70	50.855	E1
2	36.32	49.145	E2
Total		100.000	

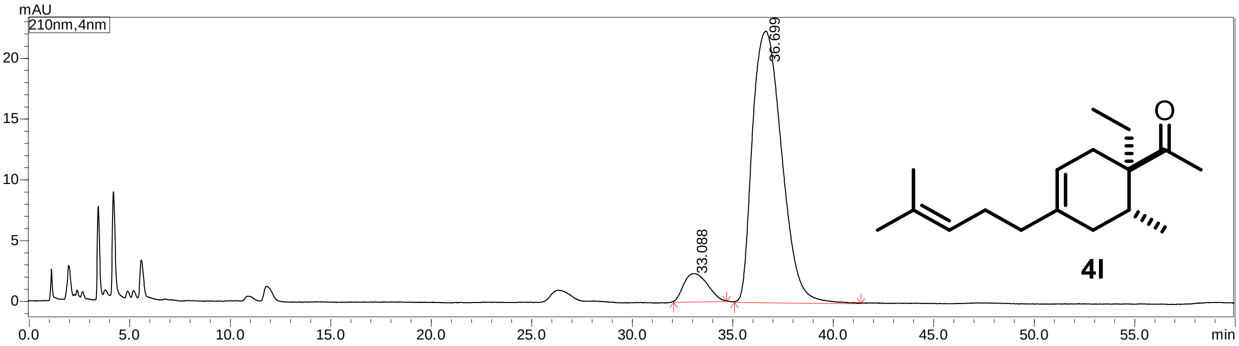

Entry	Ret. Time (Min.)	Area (%)	
1	33.09	7.268	E1
2	36.70	92.732	E2
Total		100.000	

Figure S131. HPLC traces of racemic and enantioenriched compound **4l** (on racemate the corresponding regioisomer is visible).

Conditions: column AD-3R, eluent (isocratic): water: acetonitrile 50:50 (v/v) as eluent, flow 1 mL/min, column temperature: 25 °C.

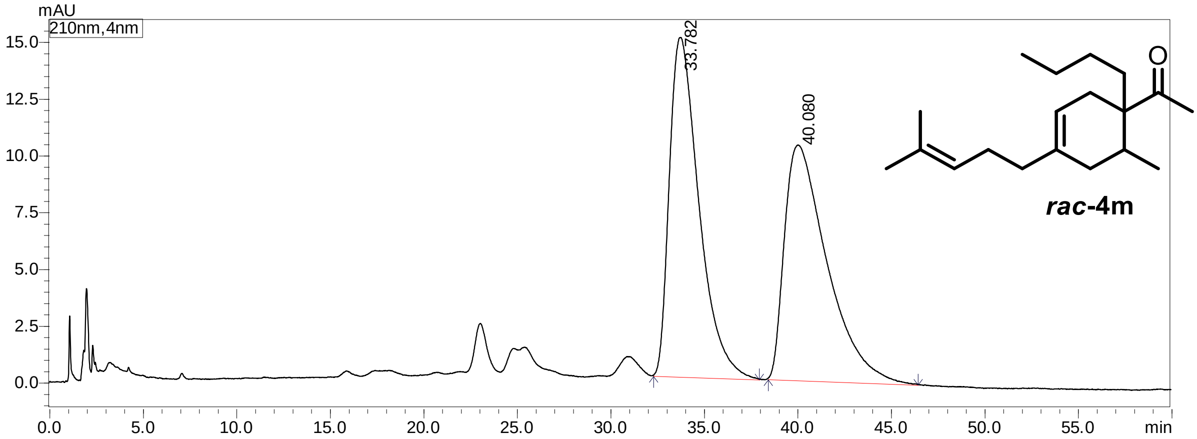

Peak#	Ret. Time	Area%	
1	33.78	50.069	E1
2	40.08	49.931	E2
Total		100.000	

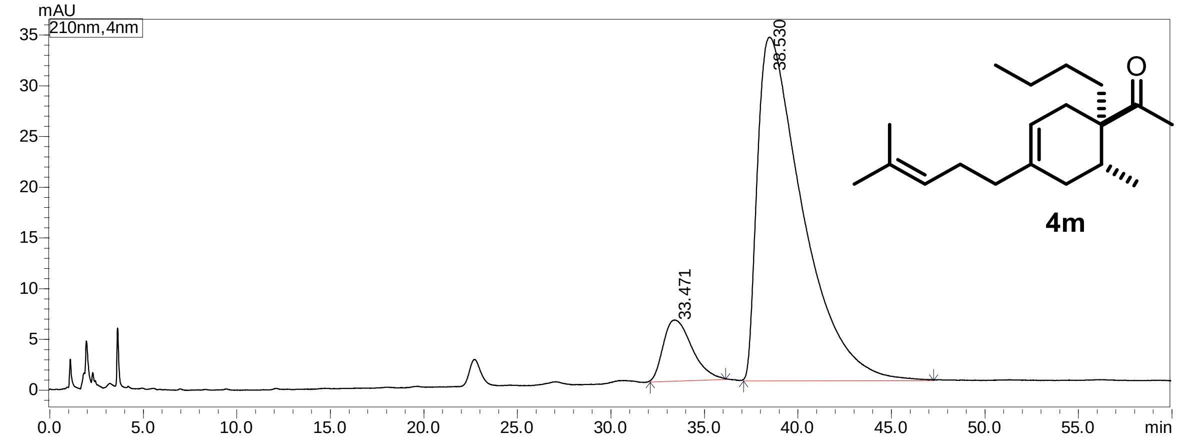

Peak#	Ret. Time	Area%	
1	33.47	9.508	E1
2	38.53	90.492	E2
Total		100.000	

Figure S132. HPLC traces of racemic and enantioenriched compound **4m** (the corresponding regioisomer is visible between 20 and 30 minutes).

Conditions: column AD-3R, eluent (isocratic): water:acetonitrile 45:55 (v/v) as eluent, flow 1 mL/min, column temperature: 25 °C.

150 mm Chiralpak IG-3, 4.6 mm i.D.
Acetonitril / Wasser = 60:40
1.0 ml/min, 17.0 MPa, 298 K
UV 220 nm

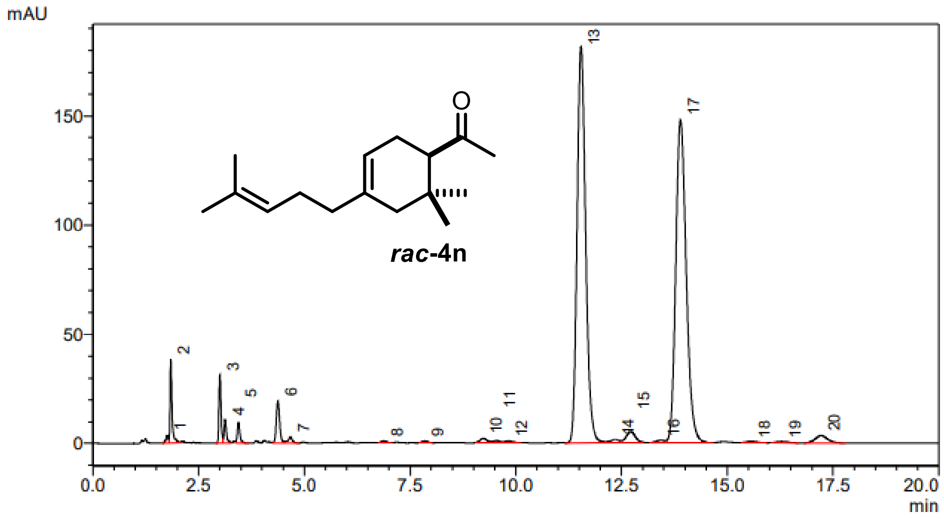

1 210nm,4nm

PDA Ch1 210nm

Peak #	Ret. Time	Area %	Name
1	1.75	0.26	
2	1.84	2.12	
3	3.00	1.84	
4	3.13	0.74	
5	3.44	0.72	
6	4.37	1.84	
7	4.67	0.36	
8	6.88	0.12	
9	7.84	0.14	
10	9.23	0.46	
11	9.55	0.21	
12	9.83	0.18	
13	11.54	43.67	1st enantiomer 1st isomer
14	12.35	0.42	
15	12.71	1.42	1st enantiomer 2nd isomer
16	13.43	0.31	
17	13.89	43.50	2nd enantiomer 1st isomer
18	15.57	0.19	
19	16.29	0.20	
20	17.22	1.30	2nd enantiomer 2nd isomer
Total		100.00	

Figure S133. HPLC traces of racemic compound **4n** (regioisomer is visible).

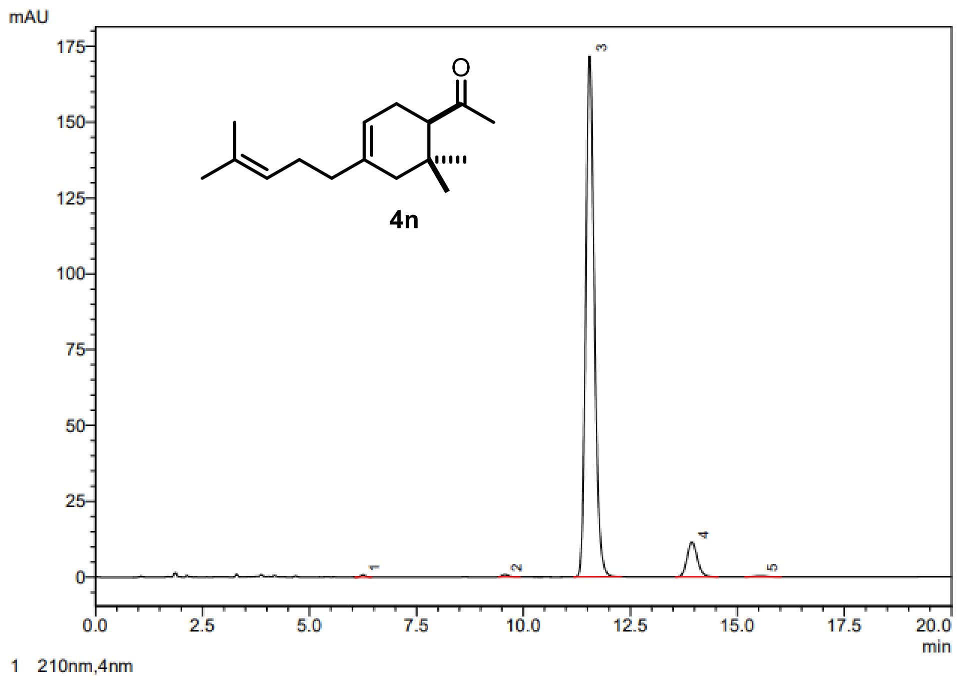

Entry	Ret. Time (Min.)	Area (%)	
1	11.55	92.605	E1
2	13.94	7.395	E2
Total		100.000	

Figure S134. HPLC traces of enantioenriched compound **4n**.

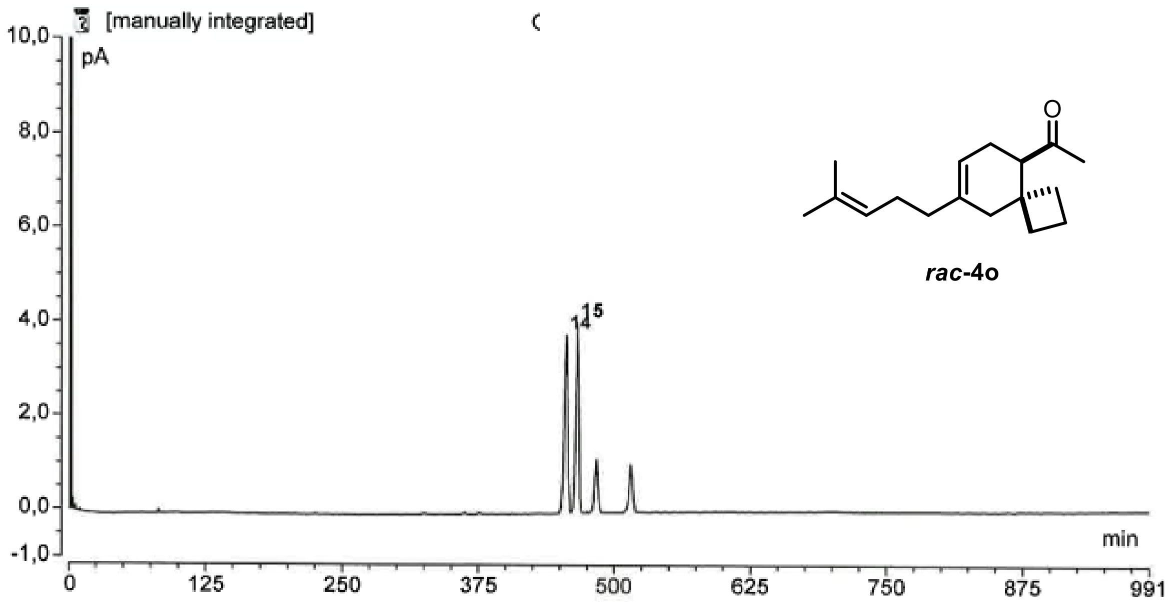

No.	Ret.Time min	Rel.Area %	Peak Name
14	456,08	49,86	MG:246
15	466,48	50,14	MG:246

Instrument parameters:

Column:	29,5 m	BGB-178/BGB-15 0,25/0,25df G/615
Temperature:	220/100 iso/350	
Gas:	0,60 bar	H2
Sample size:	0,2 µL	

Figure S135. GC traces of racemic compound **4o**. On the traces the regioisomer is visible. Racemate which was synthesized according to the general racemate preparation method, contained always significant and irremovable regioisomer 'meta'.

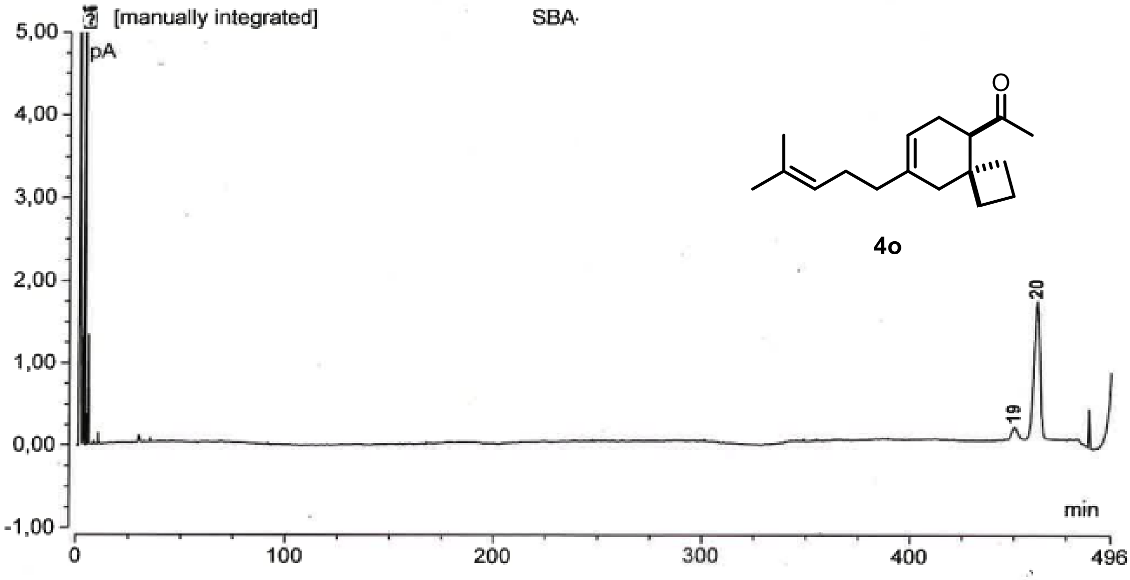

No.	Ret.Time min	Rel.Area %	Peak Name
19	449,98	9,36	MG:246
20	460,57	90,64	MG:246

Instrument parameters:

Column:	29,5 m	BGB-178/BGB-15 0,25/0,25df G/615		
Temperature:	220/100 480min iso 8/min 230/350			
Gas:	0,60 bar	H2		
Sample size:	1,0 µL	Split ratio:	20 : 1	

Figure S136. GC traces of enantioenriched compound **4o**.

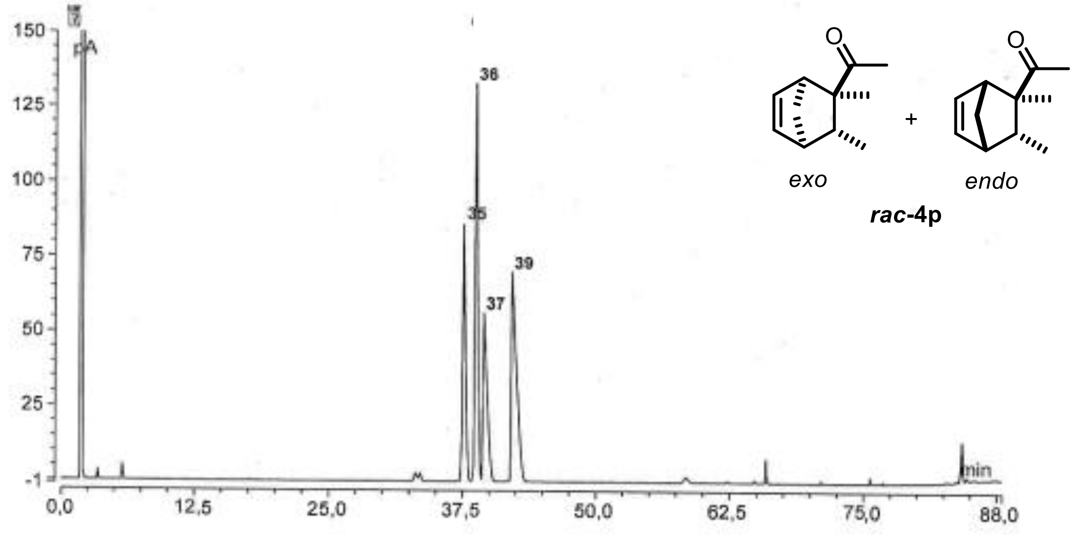

No.	Ret.Time min	Rel.Area %	Peak Name
35	37,56	19,86	.
36	38,69	30,14	..
37	39,46	19,89	.
39	42,12	30,11	..

Instrument parameters:

Column:	29,5 m	BGB-178/BGB-15 0,25/0,25df G/615
Temperature:	220 / 80, 60 min iso 6/min 230, 3 min iso / 350	
Gas:	0,50 bar	H2
Sample size:	0,2 µL	

Figure S137. GC traces of racemic (*endo* and *exo* product mixture) compound **4p**.

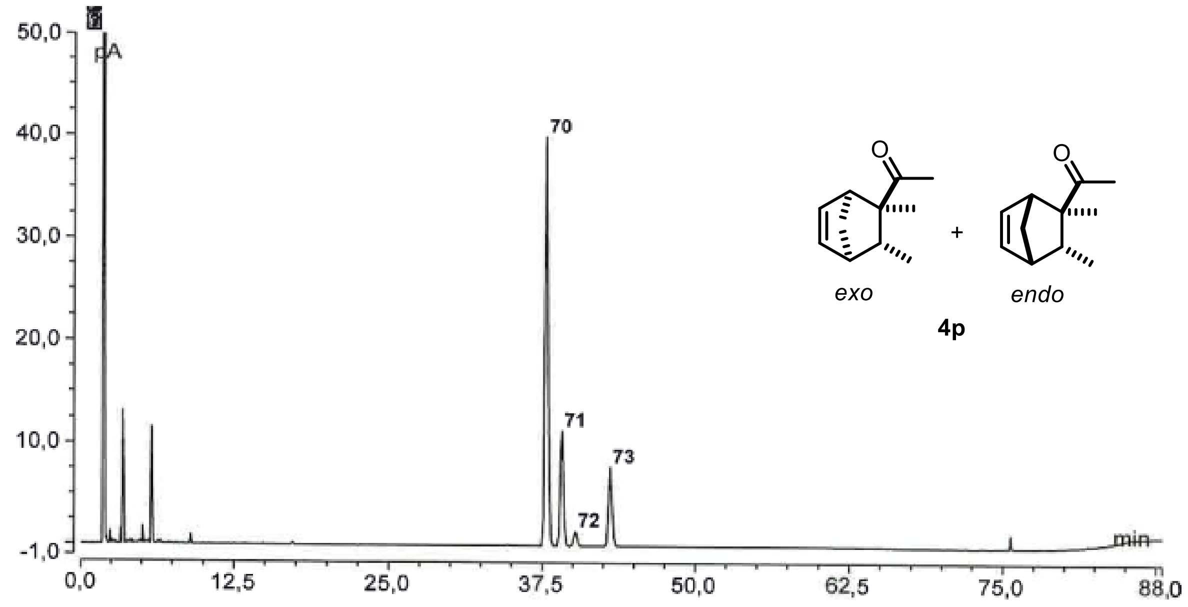

No.	Ret.Time min	Rel.Area %	Peak Name
70	37,79	64,75	.
71	39,09	18,62	..
72	40,21	2,41	.
73	43,04	14,22	..

Instrument parameters:

Column:	29,5 m	BGB-178/BGB-15 0,25/0,25df G/615
Temperature:	2220 / 80, 60 min iso 6/min 230, 3 min iso / 350	
Gas:	0,50 bar	H2
Sample size:	0,2 µL	

Figure S138. GC traces of enantioenriched (*endo* and *exo* product mixture) compound **4p**.

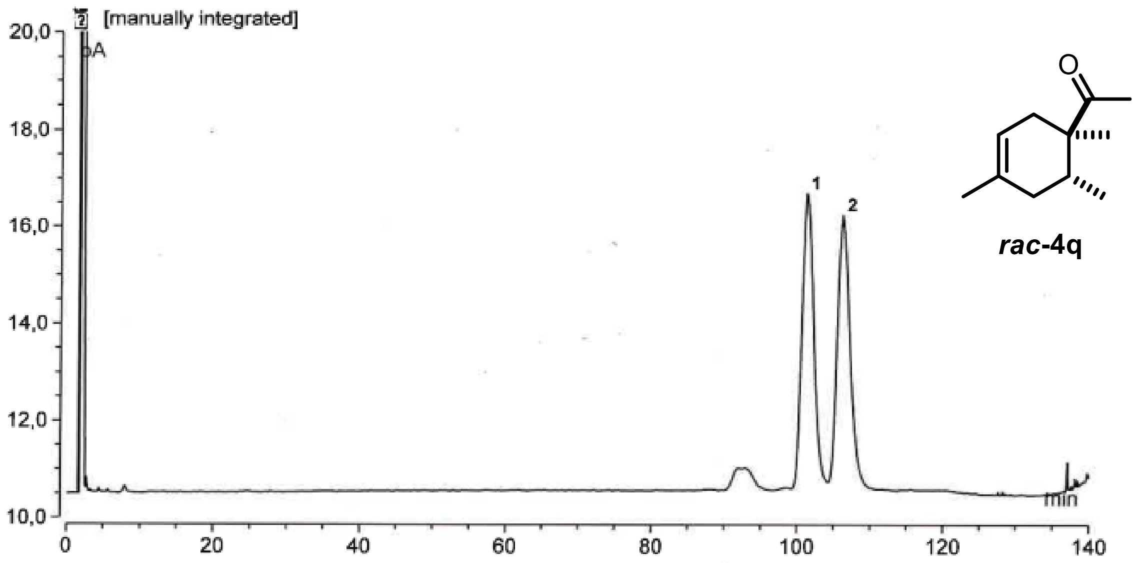

No.	Ret.Time min	Rel.Area %	Peak Name
1	101,17	50,06	.
2	106,07	49,94	.

Instrument parameters:

Column:	25,0 m	Hydrodex-beta-TBDAc-CD 0,25/?df G/681	
Temperature:	220/60 120min iso 8/min 220/350		
Gas:	0,50 bar	Hydrogen	
Sample size:	0,2 µL	Split ratio:	40 : 1

Figure S139. GC traces of racemic compound **4q**.

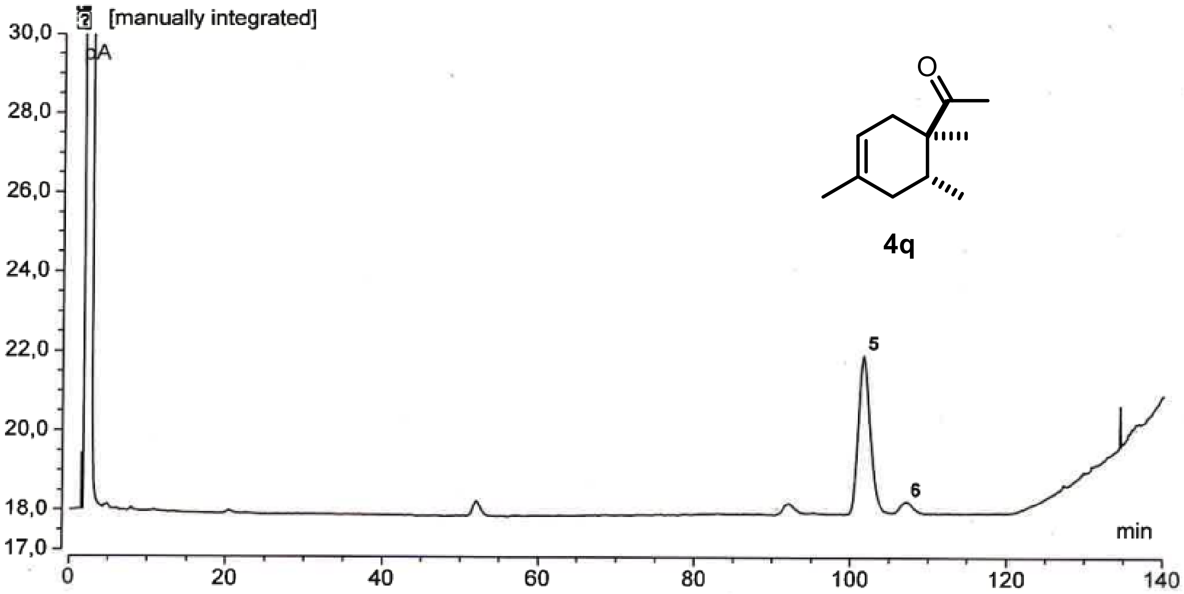

No.	Ret.Time min	Rel.Area %	Peak Name
5	101,57	93,42	.
6	107,11	6,58	.

Instrument parameters:

Column:	25,0 m	Hydrodex-beta-TBDAc-CD 0,25/?df G/681	
Temperature:	220/60 120min iso 8/min 220/350		
Gas:	0,50 bar	Hydrogen	
Sample size:	1,0 µL	Split ratio:	20 : 1

Figure S140. GC traces of enantioenriched compound **4q**.

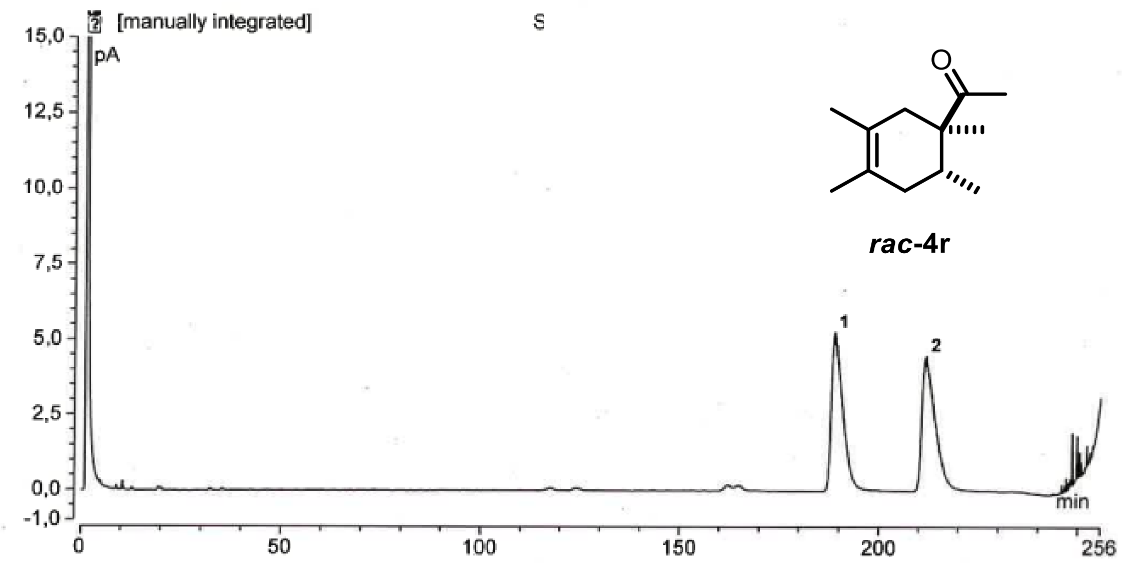

No.	Ret.Time min	Rel.Area %	Peak Name
1	188,82	50,27	.
2	211,79	49,73	.

Instrument parameters:

Column:	25,0 m	Hydrodex-gamma-TBDAc 0,25/?df G/586	
Temperature:	220/65 235min iso 8/min 230/350		
Gas:	0,60 bar	H2	
Sample size:	1,0 µL	Split ratio:	20 : 1

Figure S141. GC traces of racemic compound **4r**.

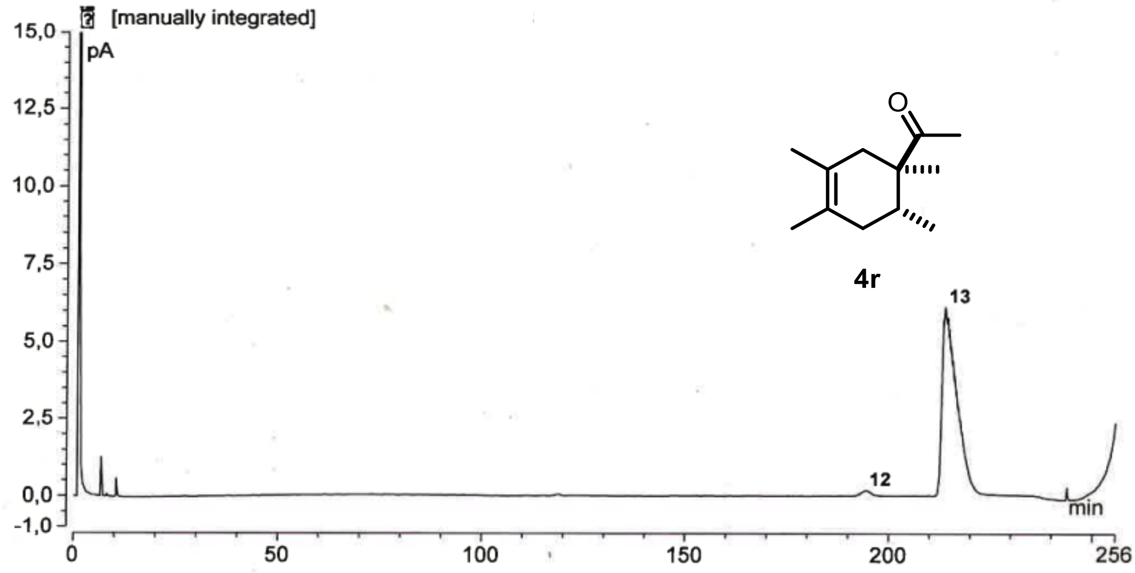

No.	Ret.Time min	Rel.Area %	Peak Name
12	194,37	1,79	.
13	213,66	98,21	.

Instrument parameters:

Column:	25,0 m	Hydrodex-gamma-TBDAc 0,25/?df G/586	
Temperature:	220/65 235min iso 8/min 230/350		
Gas:	0,60 bar	H2	
Sample size:	1,0 µL	Split ratio:	40 : 1

Figure S142. GC traces of enantioenriched compound **4r**.

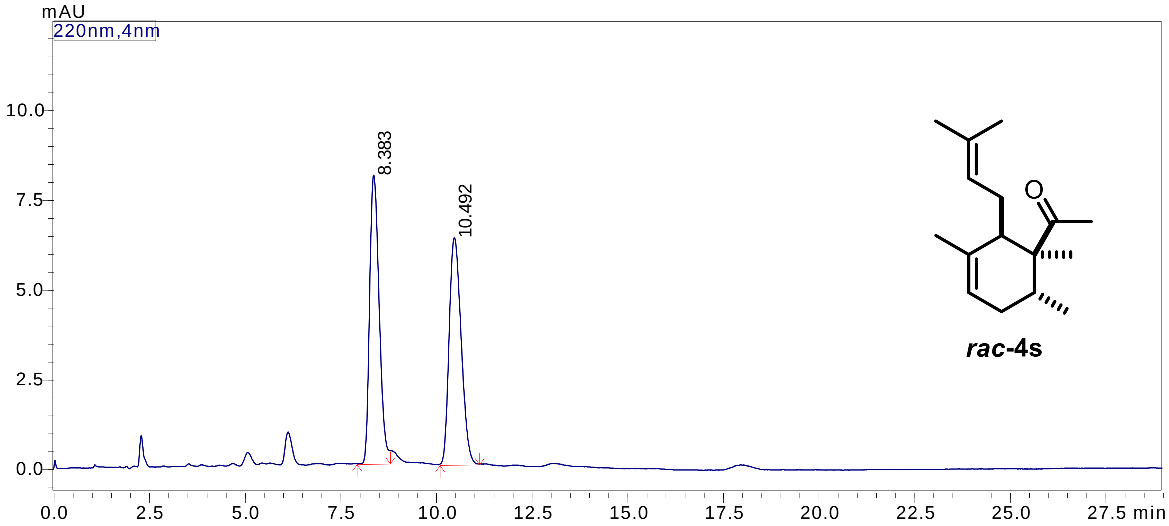

Entry	Ret. Time (Min.)	Area (%)	
1	8.38	50.988	E1
2	10.49	49.012	E2
Total		100	

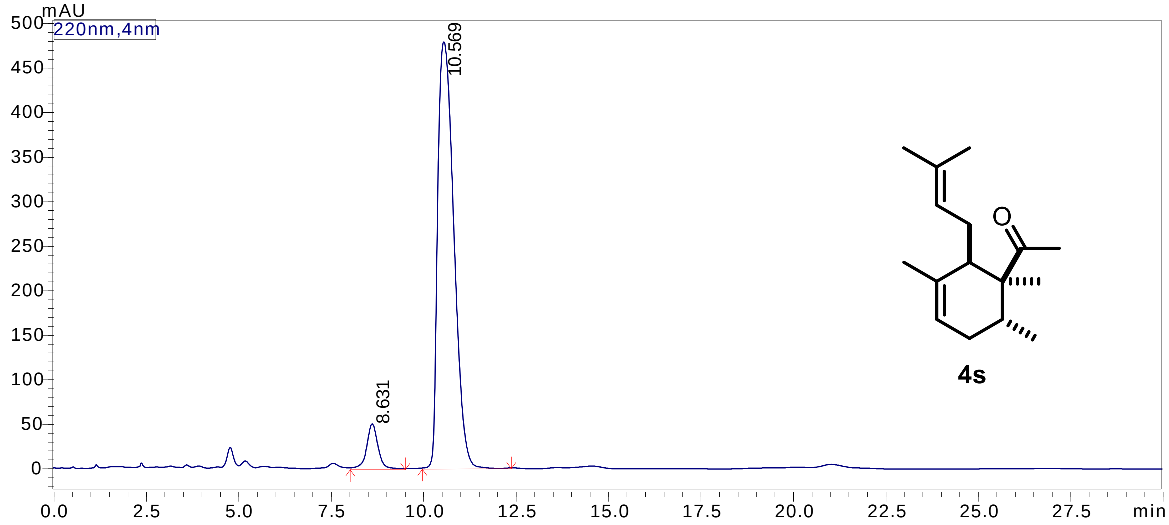

Entry	Ret. Time (Min.)	Area (%)	
1	8.60	6.783	E1
2	10.57	93.217	E2
Total		100	

Figure S143. HPLC traces of racemic and enantioenriched compound **4s** (on racemate the corresponding regioisomer is visible).

Conditions: column IG-3R, solvent system: acetonitrile:water 60:40 (v/v), flow rate: 1.0 mL/min., temp.: 25 °C.

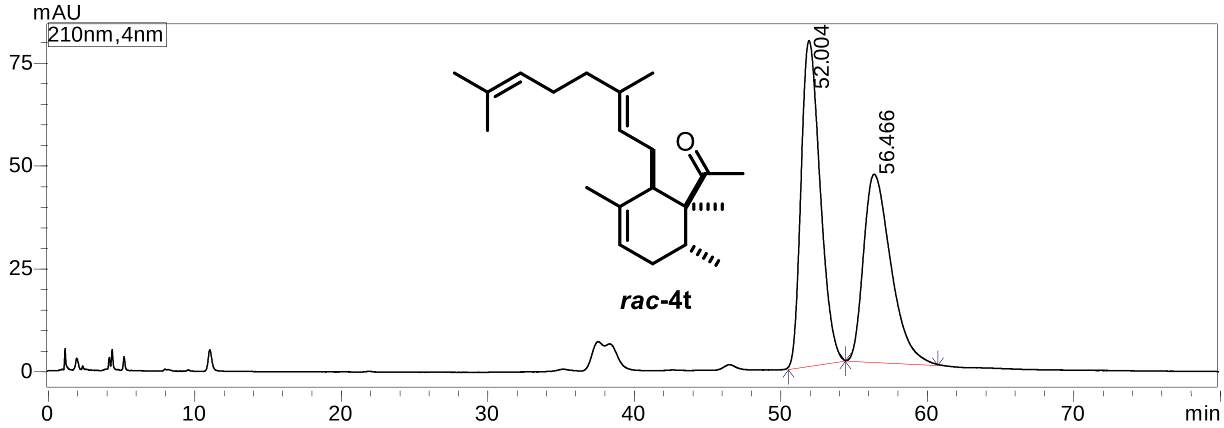

Peak#	Ret. Time	Area%	
1	52.00	54.790	E1
2	56.47	45.210	E2
Total		100.000	

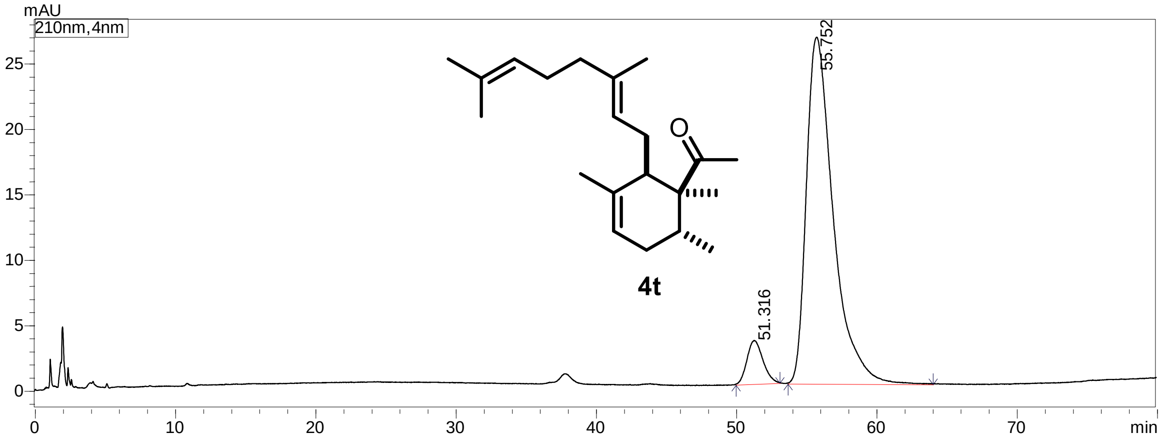

Peak#	Ret. Time	Area%	
1	51.32	6.850	E1
2	55.75	93.150	E2
Total		100.000	

Figure S144. HPLC traces of racemic and enantioenriched compound **4t** (on racemate the corresponding regioisomer is visible).

Conditions: column AD-3R, solvent system: acetonitrile:water 50:50 (v/v), flow rate: 1.0 mL/min., temp.: 25 °C.

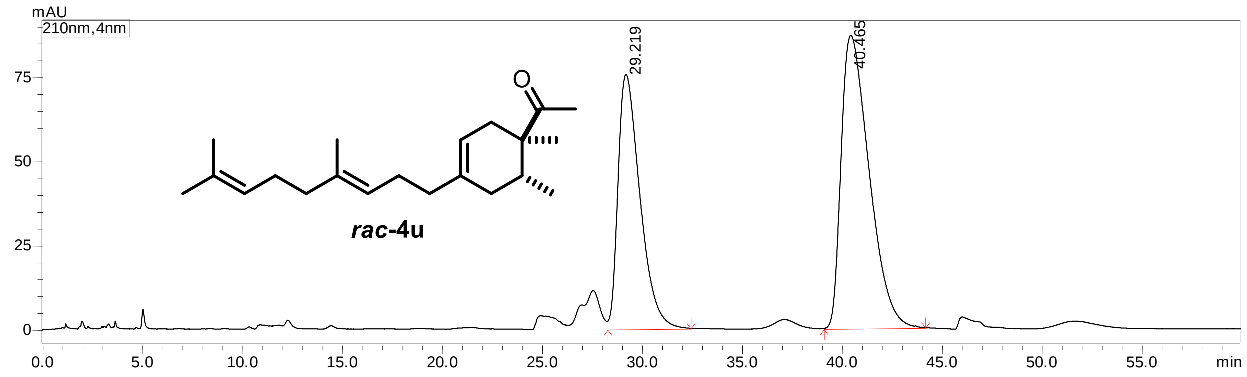

Peak#	Ret. Time	Area%	
1	29.22	40.515	E1
2	40.47	59.485	E2
Total		100.000	

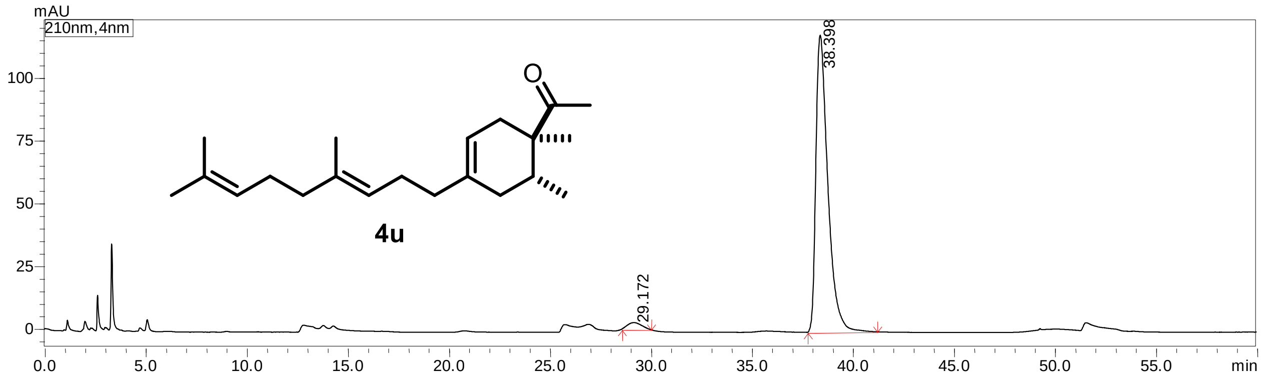

Peak#	Ret. Time	Area%	
1	29.17	2.631	E1
2	38.40	97.369	E2
Total		100.000	

Figure S145. HPLC traces of racemic and enantioenriched compound **4u** (on racemate the corresponding regioisomer is visible).

Conditions: column AD-3R, solvent system: acetonitrile:water 50:50 (v/v), flow rate: 1.0 mL/min., temp.: 25 °C.

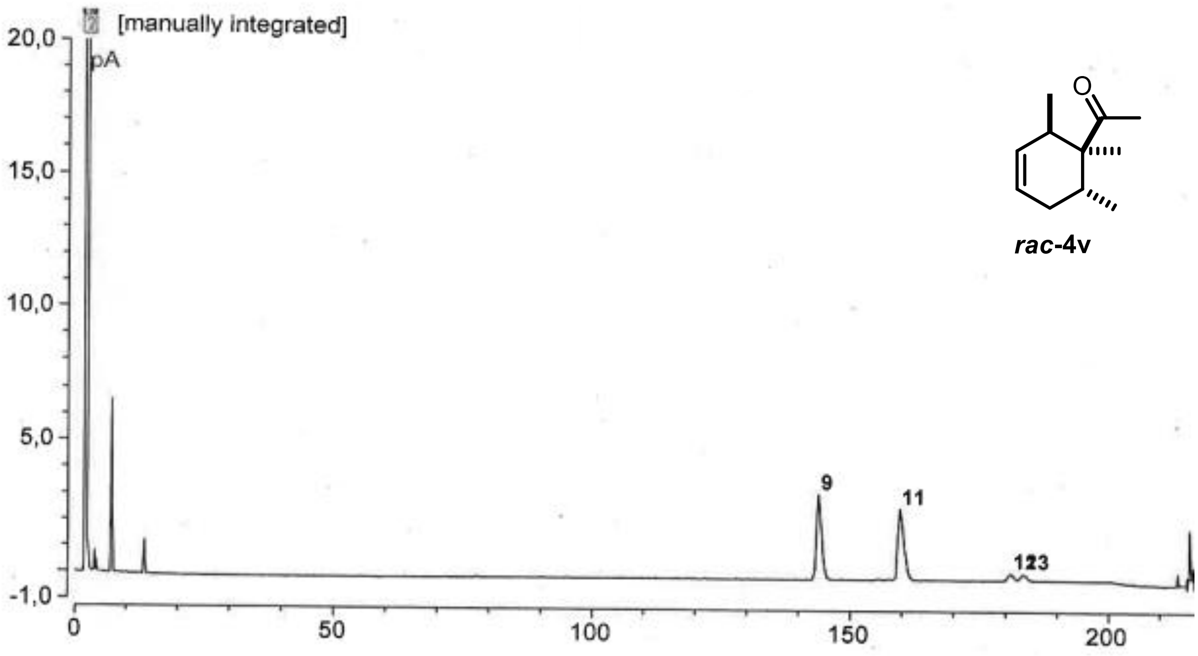

rac-**4v**

No.	Ret.Time min	Rel.Area %	Peak Name
9	143,81	45,64	.
11	159,56	45,57	.
12	180,97	4,42	..
13	183,32	4,38	..

Instrument parameters:

Column:	29,5 m	BGB-178/BGB-15 0,25/0,25df G/615
Temperature:	220 / 55, 200 min iso 8/min 230, 3 min iso / 350	
Gas:	0,50 bar	H2
Sample size:	1,0 µL	

Figure S146. GC traces of racemic compound **4v** (corresponding regio- ans stereoisomers are visible)

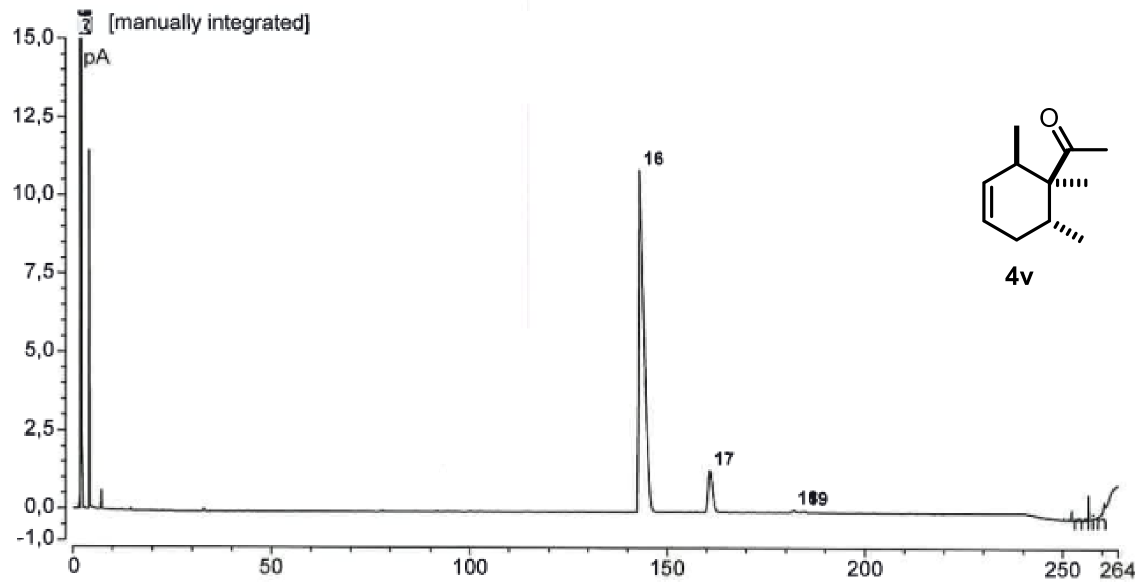

No.	Ret.Time min	Rel.Area %	Peak Name
16	142,92	90,48	.
17	160,78	8,92	.
18	181,93	0,38	..
19	184,48	0,22	..

Instrument parameters:

Column:	29,5 m	BGB-178/BGB-15 0,25/0,25df G/615
Temperature:	220 / 55, 240 min iso 8/min 230, 3 min iso / 350	
Gas:	0,50 bar	H2
Sample size:	0,2 µL	

Figure S147. GC traces of enantioenriched compound **4v** (corresponding regio- ans stereoisomers are visible).

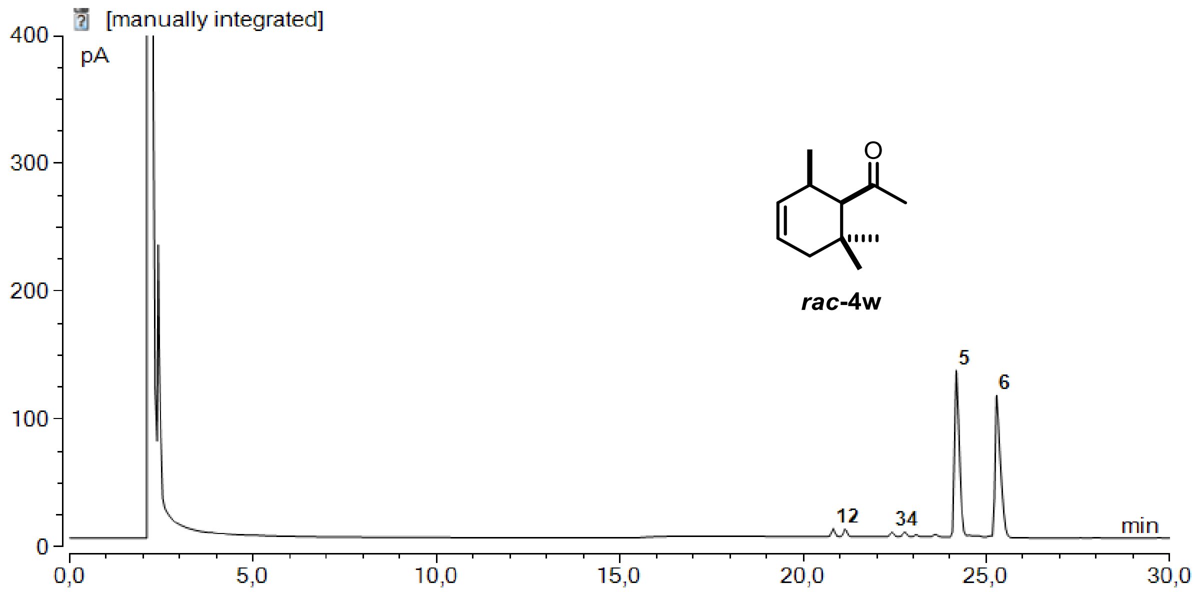

No.	Ret.Time min	area-% %	Peak Name
1	20,82	1,74	
2	21,15	1,21	
3	22,42	1,19	
4	22,77	1,17	
5	24,18	47,41	
6	25,27	47,28	

Instrument parameters:

Column:	30 m	BGB-176
Temperature:	220/80 1/min 220 5min iso/350	
Gas:	0,50 bar	Helium
Sample size:	5,0 µL	

Figure S148. GC traces of racemic compound **4w** (corresponding regio- ans stereoisomers are visible).

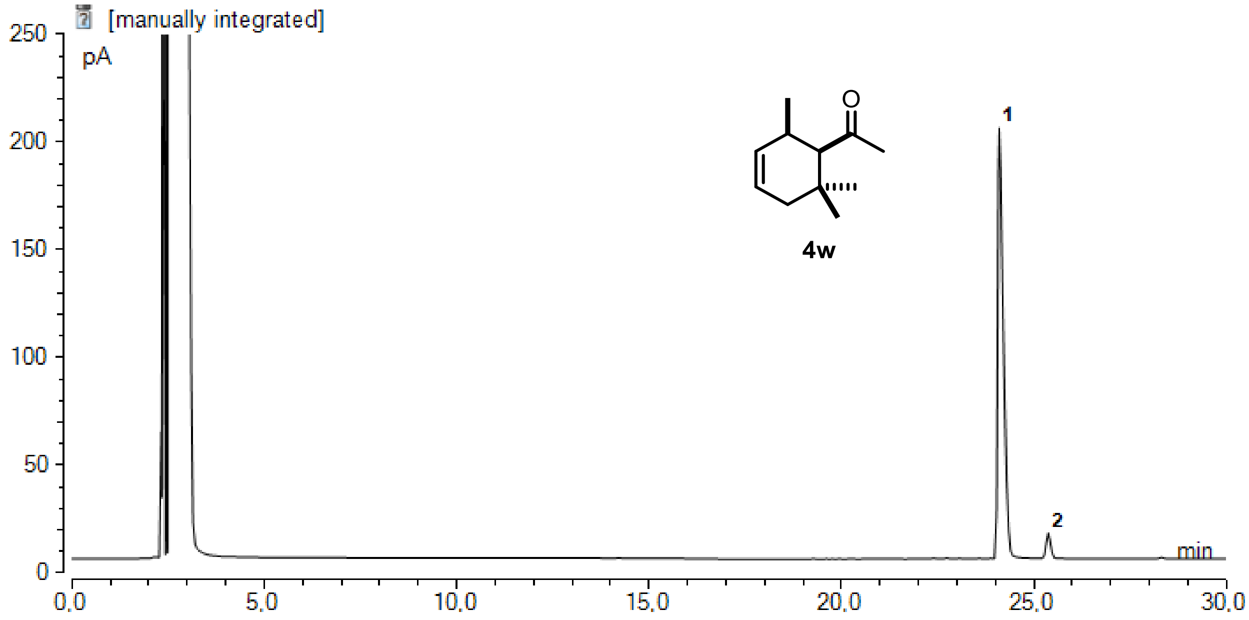

No.	Ret.Time min	area-% %	Peak Name
1	24,10	95,53	
2	25,37	4,47	

Instrument parameters:

Column:	30 m	BGB-176
Temperature:	220/80 1/min 220 5min iso/350	
Gas:	0,50 bar	Helium
Sample size:	5,0 µL	

Figure S149. GC traces of enantioenriched compound **4w**.

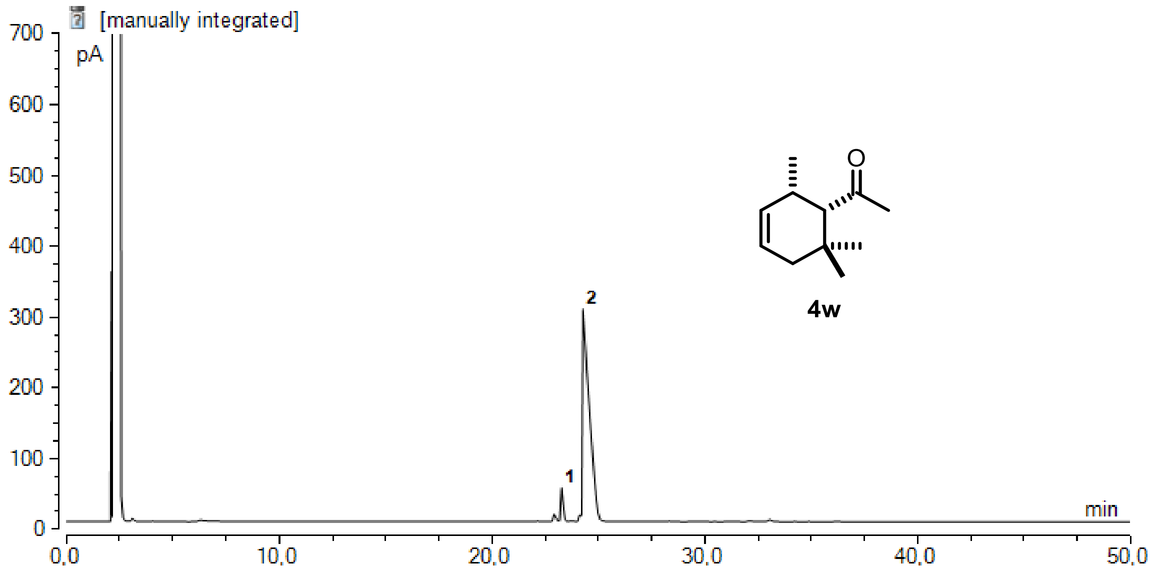

No.	Ret.Time min	area-% %	Peak Name
1	23,28	5,14	
2	24,27	94,86	

Instrument parameters:

Column:	30 m	n.a.
Temperature:	220/80 1/min 220 5min iso/350	
Gas:	0,50 bar	Helium
Sample size:	3,0 µL	

Figure S150. GC traces of enantioenriched compound ***ent*-4w**.

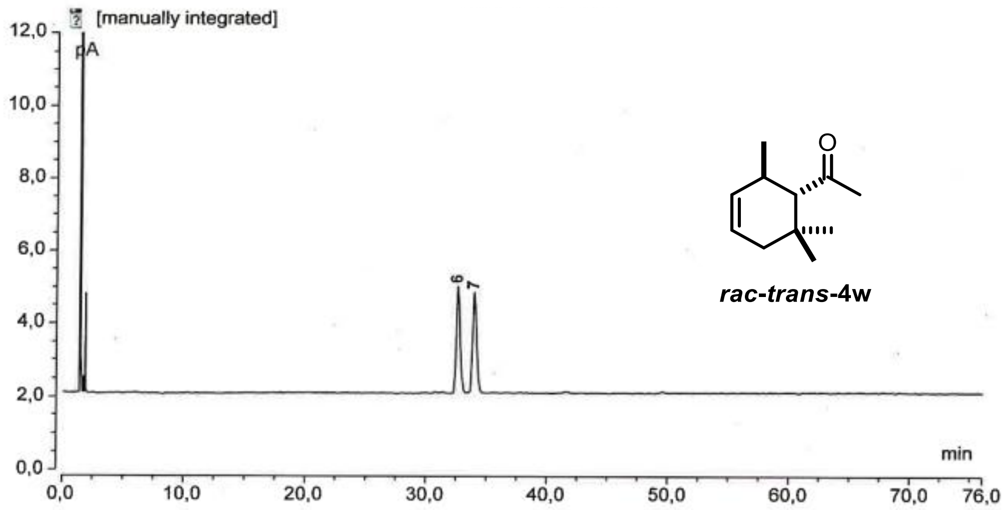

No.	Ret.Time min	Rel.Area %	Peak Name
6	32,58	49,92	.
7	33,93	50,08	.

Instrument parameters:

Column:	25,0 m	Hydrodex-gamma DiMOM 0,25/?df G/652
Temperature:	220 / 70 iso / 350	
Gas:	0,50 bar	Hydrogen
Sample size:	0,2 µL	Split ratio: 80 : 1

Figure S151. GC traces of racemic compound ***trans*-4w**.

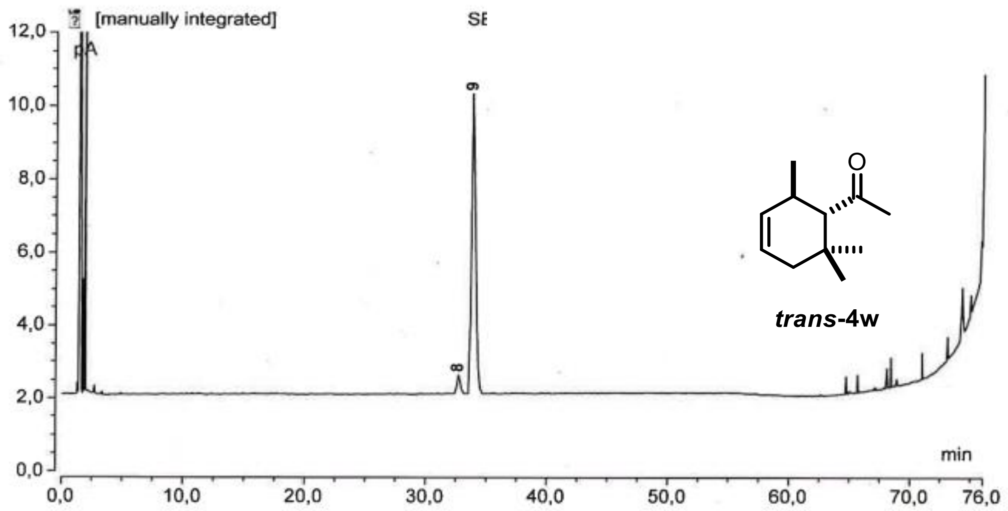

No.	Ret.Time min	Rel.Area %	Peak Name
8	32,68	5,32	.
9	33,76	94,68	.

Instrument parameters:

Column:	25,0 m	Hydrodex-gamma DiMOM 0,25/?df G/652	
Temperature:	220 / 70, 55 min iso 8/min 240 / 350		
Gas:	0,50 bar	Hydrogen	
Sample size:	0,2 µL	Split ratio:	10 : 1

Figure S152. GC traces of enantioenriched compound ***trans*-4w**.

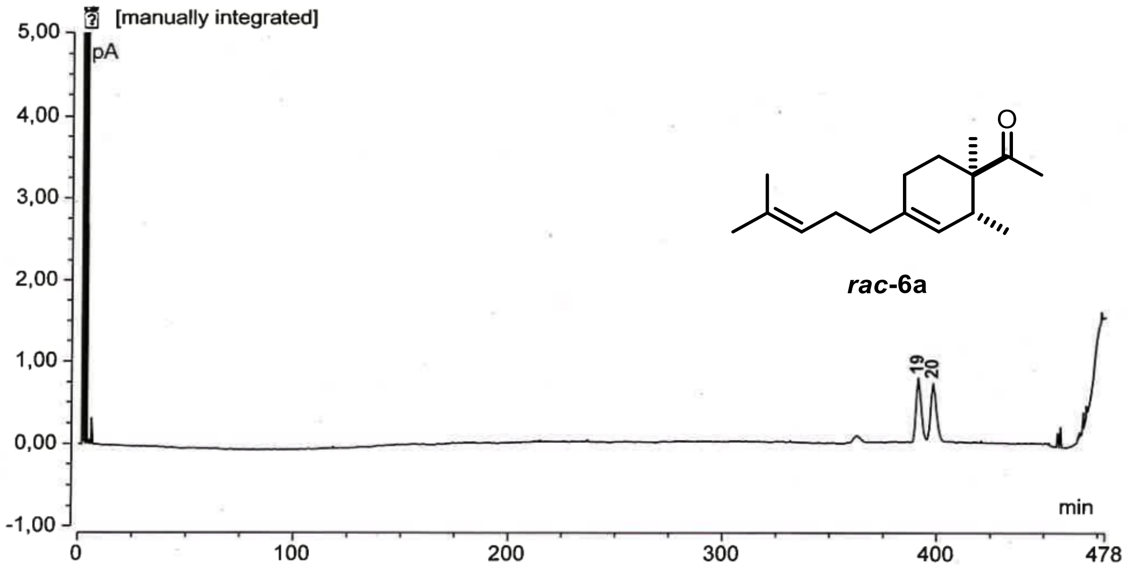

No.	Ret.Time min	Rel.Area %	Peak Name
19	390,92	49,39	..
20	397,93	50,61	..

Instrument parameters:

Column:	30,0 m	BGB-176/BGB-15 0,25/0,25df G/618		
Temperature:	220 / 100, 450 min iso 6/min 240, 5 min iso / 350			
Gas:	0,50 bar	H2		
Sample size:	0,2 µL	Split ratio:	100 : 1	

Figure S153. GC traces of racemic compound **6a**.

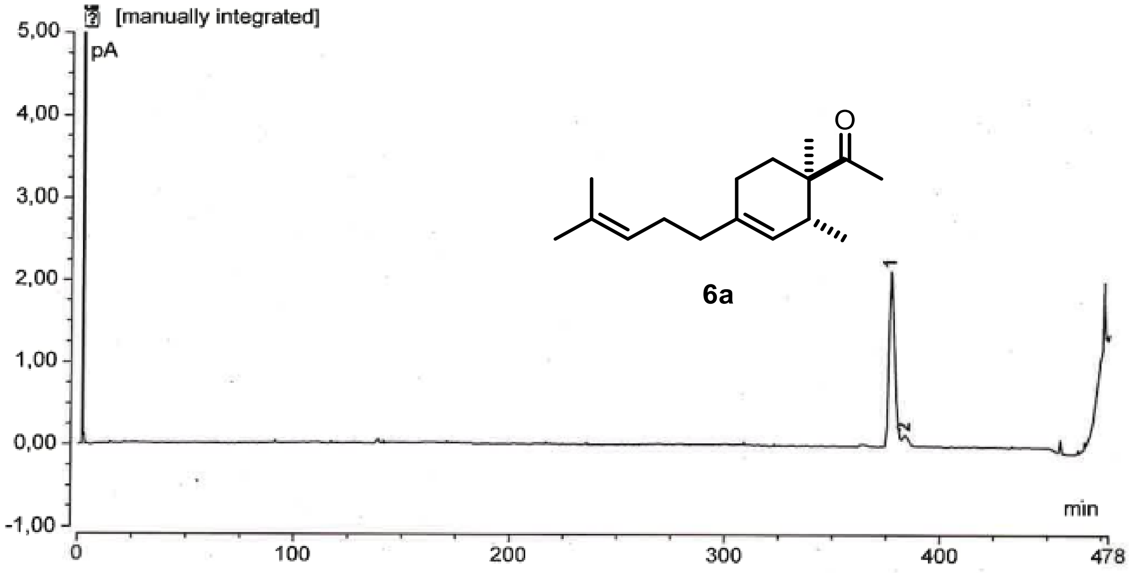

No.	Ret.Time min	Rel.Area %	Peak Name
1	376,65	93,64	.
2	383,72	6,36	.

Instrument parameters:

Column:	30,0 m	BGB-176/BGB-15 0,25/0,25df G/618		
Temperature:	220 / 100, 450 min iso 6/min 240, 5 min iso / 350			
Gas:	0,50 bar	H2		
Sample size:	1,0 µL	Split ratio:	60 : 1	

Figure S154. GC traces of enantioenriched compound **6a**.

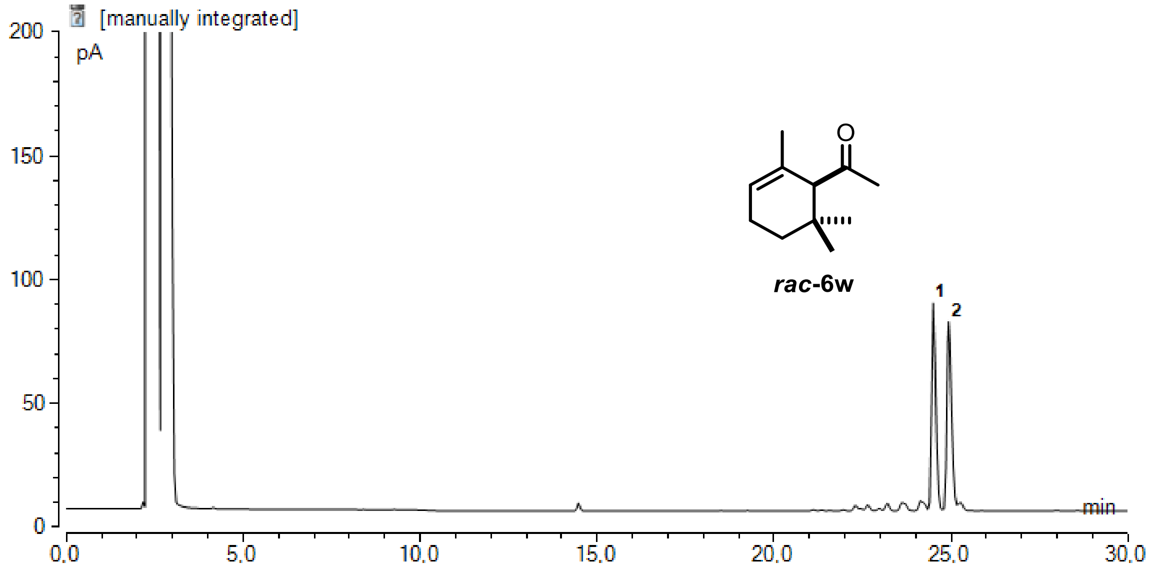

No.	Ret.Time min	area-% %	Peak Name
1	24,49	50,56	
2	24,93	49,44	

Instrument parameters:

Column:	30 m	BGB-176
Temperature:	220/80 1/min 220 5min iso/350	
Gas:	0,50 bar	Helium
Sample size:	5,0 µL	

Figure S155. GC traces of racemic compound **6w**.

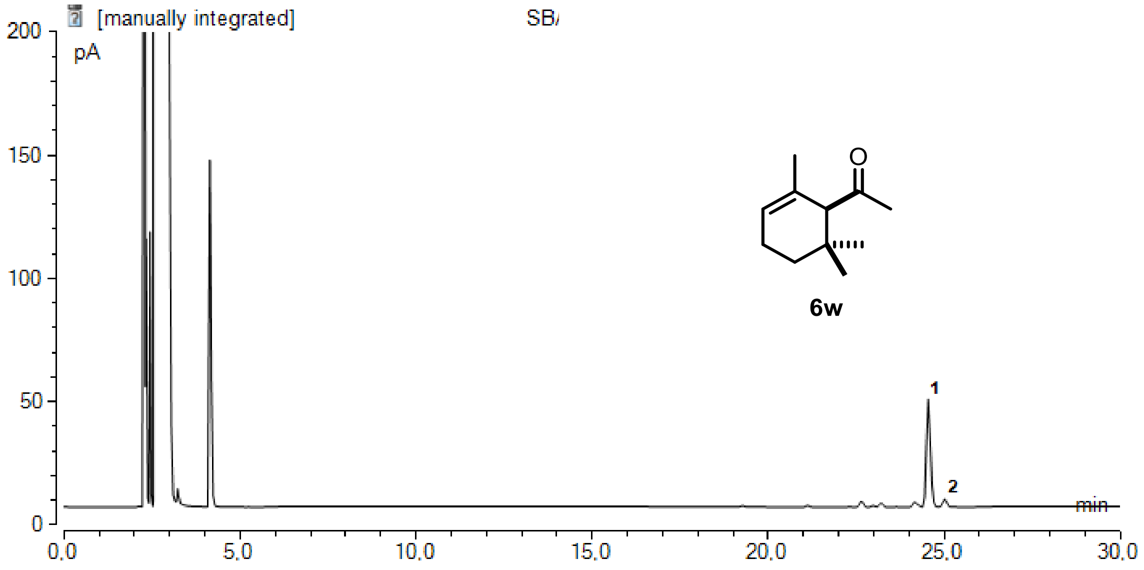

No.	Ret.Time min	area-% %	Peak Name
1	24,54	94,64	
2	25,01	5,36	

Instrument parameters:

Column:	30 m	BGB-176
Temperature:	220/80 1/min 220 5min iso/350	
Gas:	0,50 bar	Helium
Sample size:	1,0 µL	

Figure S156. GC traces of enantioenriched compound **6w**.

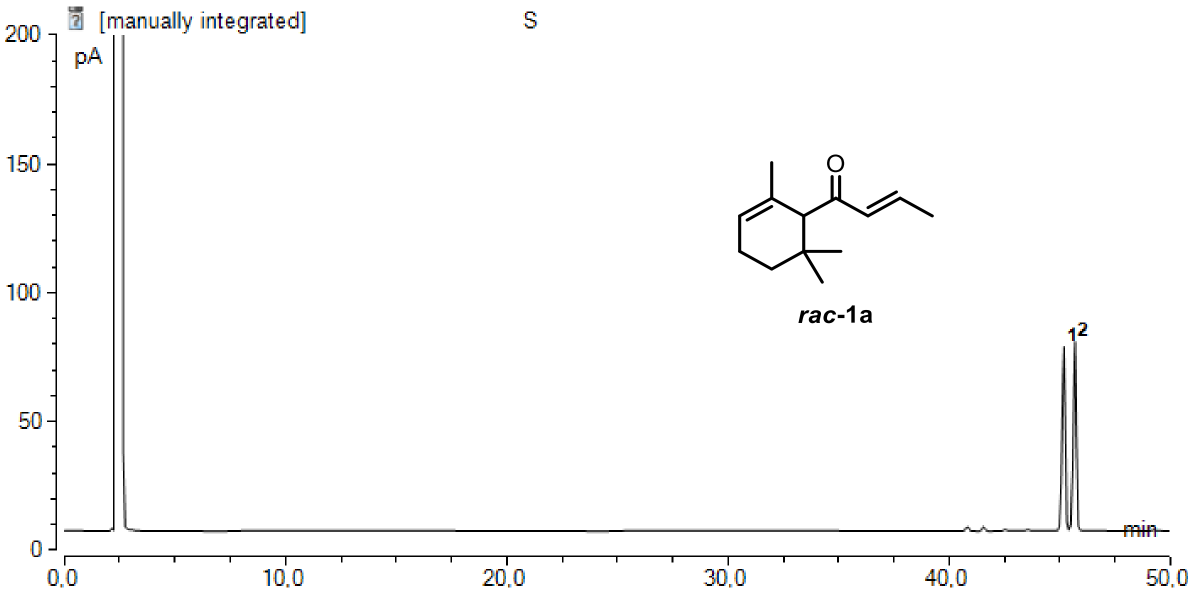

No.	Ret.Time min	area-% %	Peak Name
1	45,19	49,86	
2	45,69	50,14	

Instrument parameters:

Column:	30 m	BGB-176
Temperature:	220/80 1/min 220 5min iso/350	
Gas:	0,50 bar	Helium
Sample size:	1,0 µL	

Figure S157. GC traces of racemic α-Damascone **(*rac*)-1a**.

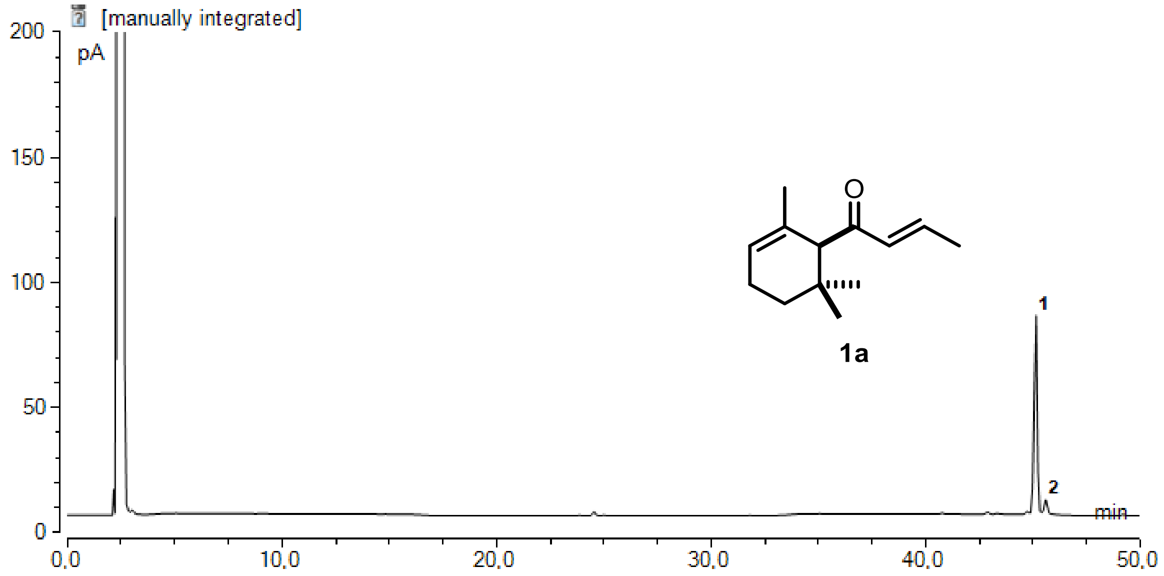

No.	Ret.Time min	area-% %	Peak Name
1	45,14	94,55	
2	45,60	5,45	

Instrument parameters:

Column:	30 m	BGB-176
Temperature:	220/80 1/min 220 5min iso/350	
Gas:	0,50 bar	Helium
Sample size:	3,0 µL	

Figure S158. GC traces of enantioenriched (*S*)-α-Damascone **(-)-1a**.

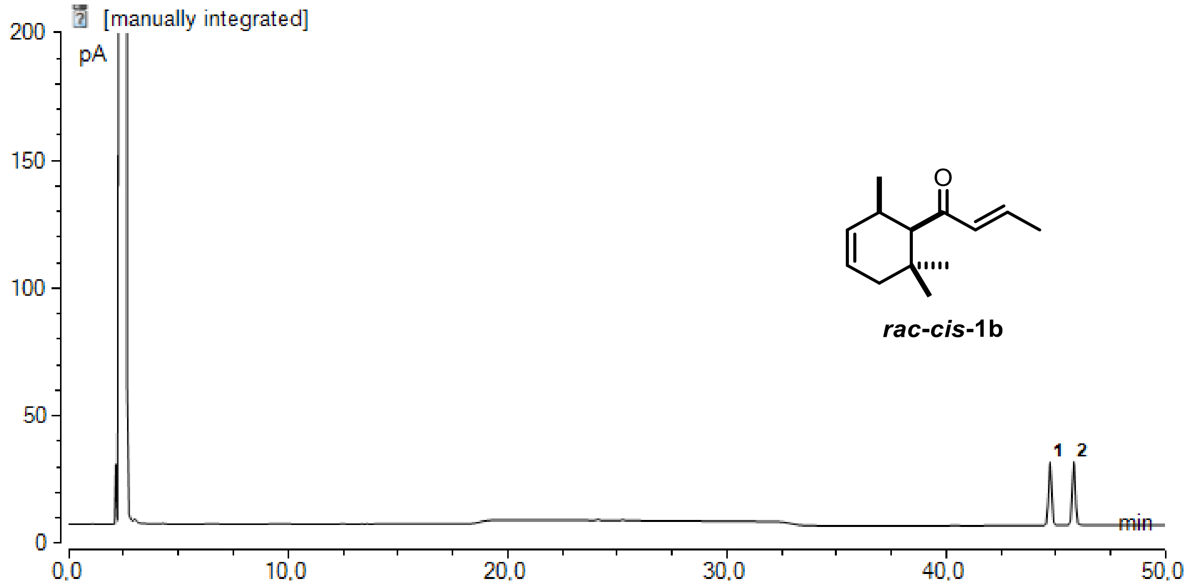

No.	Ret.Time min	area-% %	Peak Name
1	44,73	49,80	
2	45,81	50,20	

Instrument parameters:

Column:	30 m	BGB-176
Temperature:	220/80 1/min 220 5min iso/350	
Gas:	0,50 bar	Helium
Sample size:	3,0 µL	

Figure S159. GC traces of racemic *cis*-δ-Damascone **(*rac*)-*cis*-1b**.

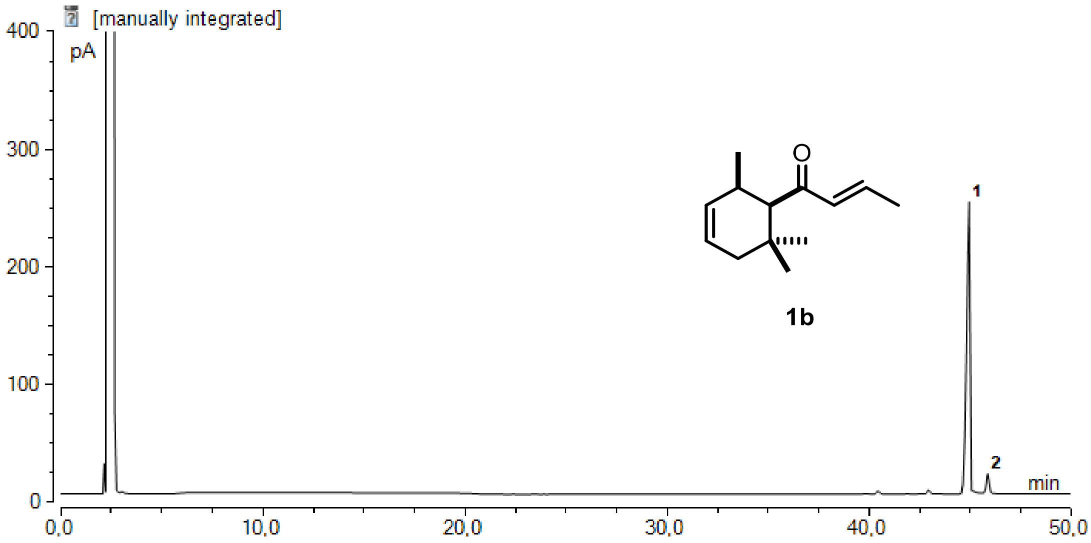

No.	Ret.Time min	area-% %	Peak Name
1	44,94	95,22	
2	45,86	4,78	

Instrument parameters:

Column:	30 m	BGB-176
Temperature:	220/80 1/min 220 5min iso/350	
Gas:	0,50 bar	Helium
Sample size:	3,0 µL	

Figure S160. GC traces of enantioenriched (*R*,*R*)-*cis*-δ-Damascone **(-)-*cis*-1b**.

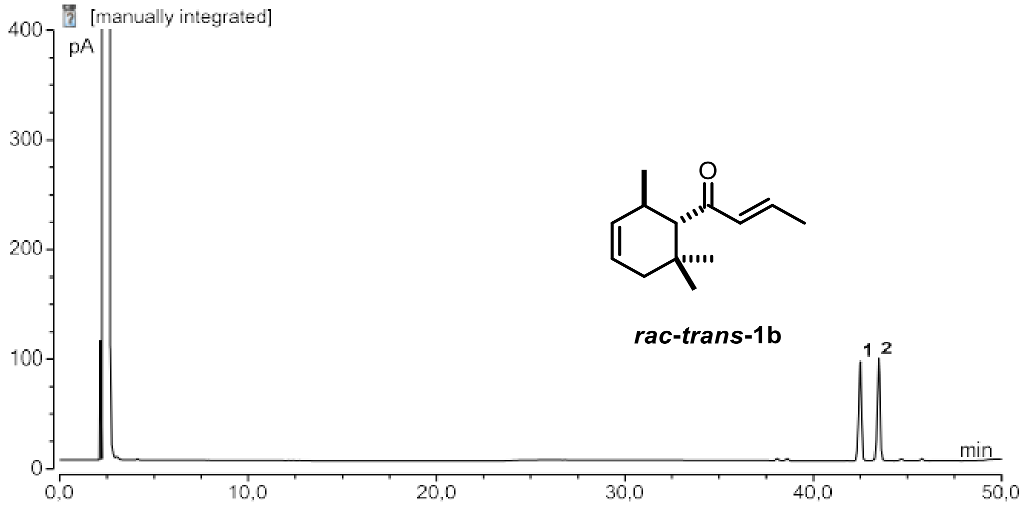

No.	Ret.Time min	area-% %	Peak Name
1	42,51	50,24	
2	43,48	49,76	

Instrument parameters:

Column:	30 m	n.a.
Temperature:	220/80 1/min 220 5min iso/350	
Gas:	0,50 bar	Helium
Sample size:	3,0 µL	

Figure S161. GC traces of racemic *trans*-δ-Damascone **(*rac*)-*trans*-1b**.

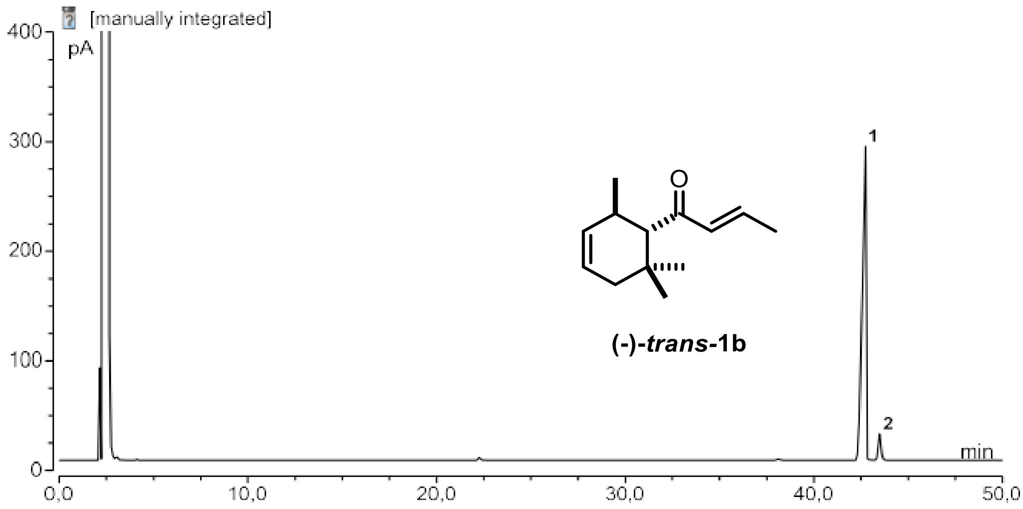

No.	Ret.Time min	area-% %	Peak Name
1	42,73	95,20	
2	43,49	4,80	

Instrument parameters:

Column:	30 m	n.a.
Temperature:	220/80 1/min 220 5min iso/350	
Gas:	0,50 bar	Helium
Sample size:	3,0 µL	

Figure S162. GC traces of enantioenriched (1*S*,2*R*)-*trans*-δ-Damascone **(-)-*trans*-1b**.

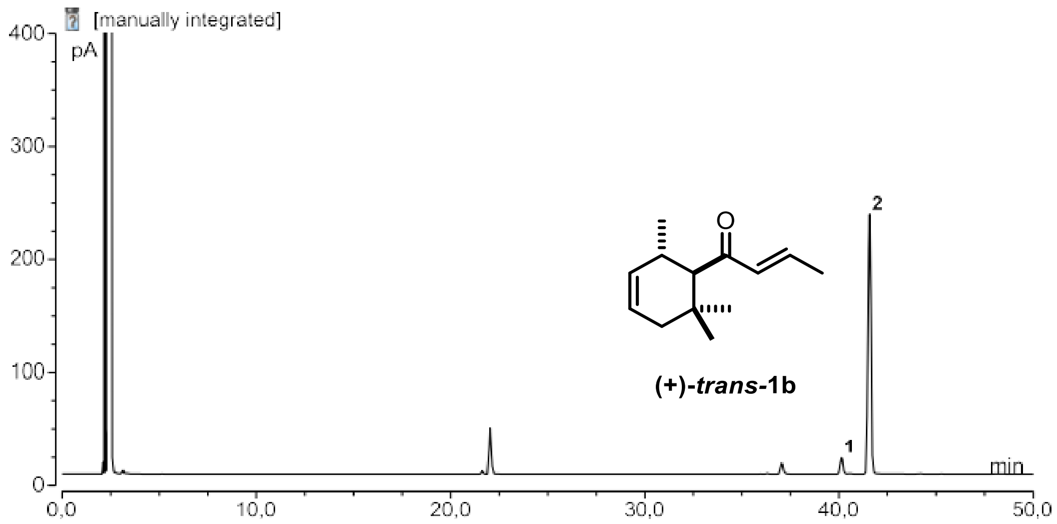

No.	Ret.Time<br>min	area-%<br>%	Peak Name
1	40,13	5,51	
2	41,58	94,49	

Instrument parameters:

Column:	30 m	n.a.
Temperature:	220/80 1/min 220 5min iso/350	
Gas:	0,50 bar	Helium
Sample size:	1,0 µL	

Figure S163. GC traces of enantioenriched (1*R*,2*S*)-*trans*-δ-Damascone **(+)-*trans*-1b**.

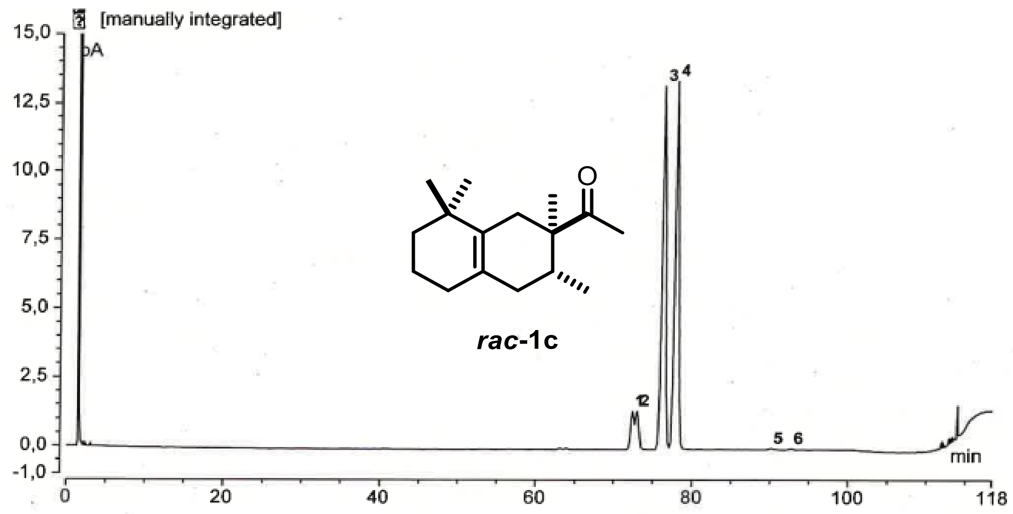

No.	Ret.Time min	Rel.Area %	Peak Name
1	72,37	4,35	***
2	72,99	4,49	***
3	76,27	45,19	isoESuper
4	77,91	45,47	isoESuper
5	90,07	0,26	isogamma
6	92,66	0,23	isogamma

Instrument parameters:

Column:	30,0 m	BGB-176/BGB-15 0,25/0,25df G/618		
Temperature:	220 / 117, 100 min iso 8/min 240, 3 min iso / 350			
Gas:	0,70 bar	H2		
Sample size:	0,2 µL	Split ratio:	60 : 1	

Figure S164. GC traces of racemic compound **1c**.

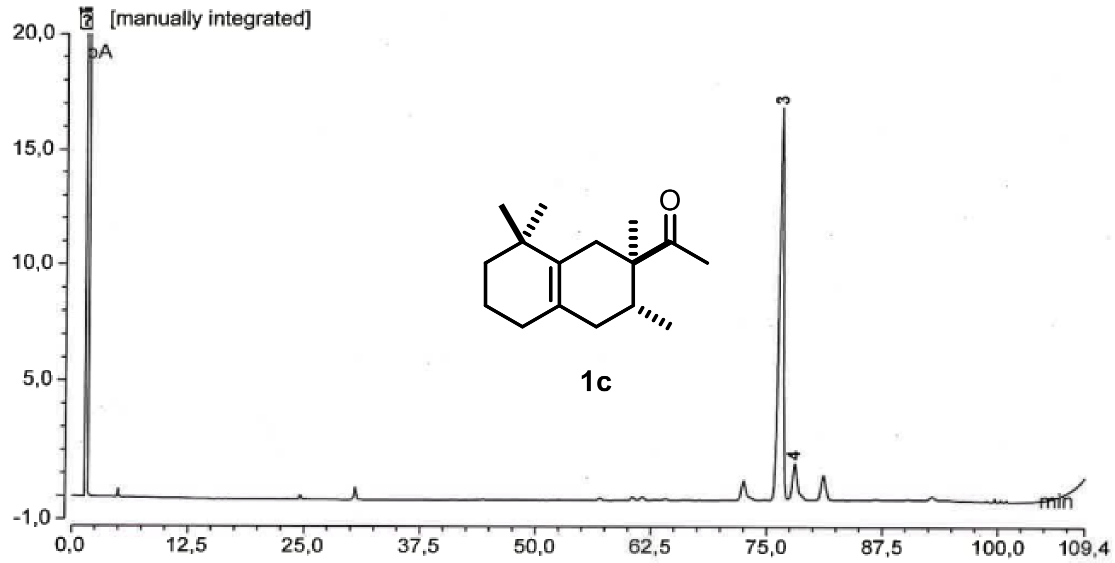

No.	Ret.Time min	Rel.Area %	Peak Name
3	76,47	91,05 .	
4	78,04	8,95 .	

Instrument parameters:

Column:	30,0 m	BGB-176/BGB-15 0,25/0,25df G/618	
Temperature:	220 / 117, 94 min iso 8/min 240 / 350		
Gas:	0,70 bar	H2	
Sample size:	0,2 µL	Split ratio:	60 : 1

Figure S165. GC traces of enantioenriched compound **1c**.

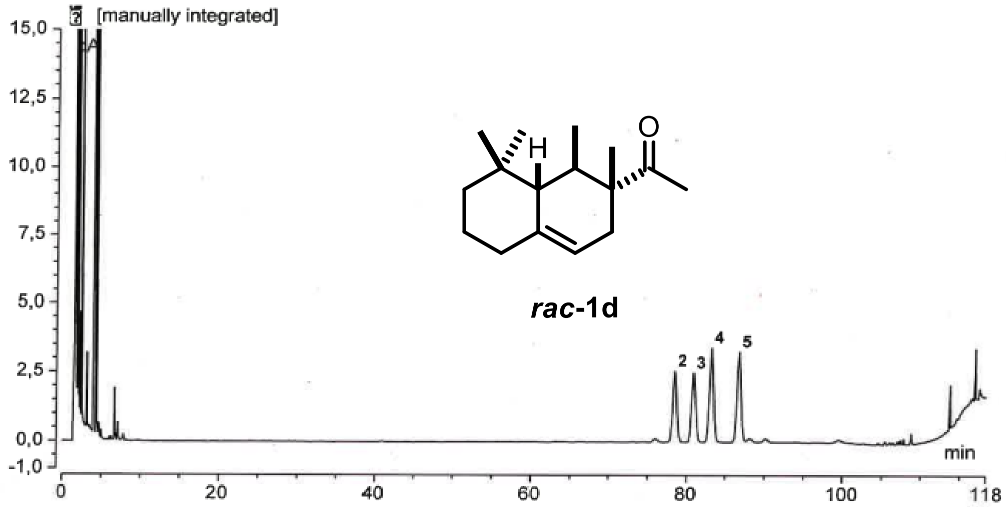

No.	Ret.Time min	Rel.Area %	Peak Name
2	78,42	20,89	*
3	80,85	20,80	*
4	83,20	29,21	**
5	86,71	29,10	**

Instrument parameters:

Column:	30,0 m	BGB-176/BGB-15 0,25/0,25df G/618	
Temperature:	220 / 117, 100 min iso 8/min 240, 3 min iso / 350		
Gas:	0,70 bar	H2	
Sample size:	0,2 µL	Split ratio:	20 : 1

Figure S166. GC traces of racemic compound **1d** in mixture with **1e**

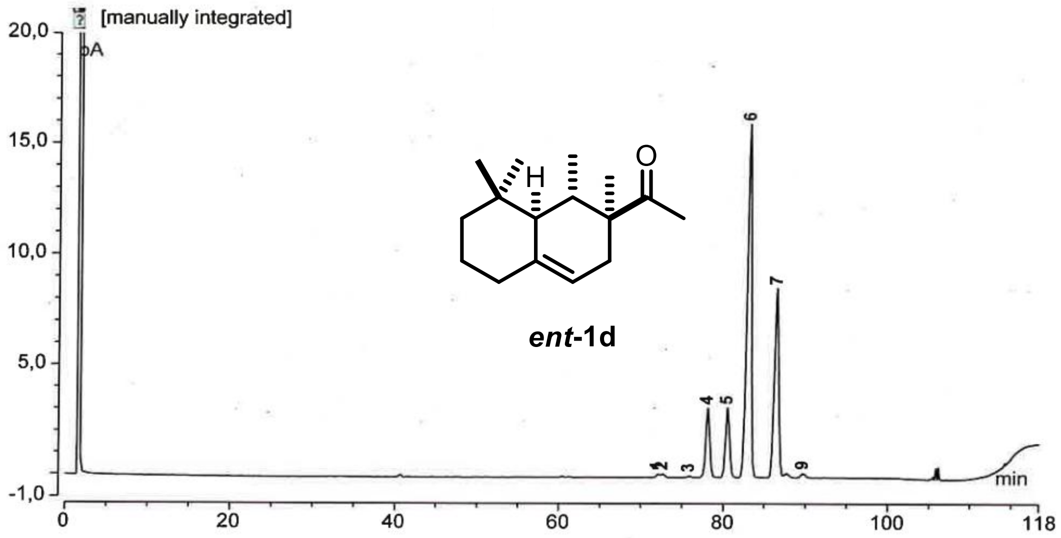

No.	Ret.Time min	Rel.Area %	Peak Name
1	72,08	0,41	***
2	72,63	0,46	***
3	75,87	0,27	isoESuper + isoalpha
4	78,06	9,50	isoESuper + isoalpha + *'
5	80,47	9,66	*
6	83,06	51,63	**
7	86,41	27,35	**
9	89,68	0,72	isogamma

Instrument parameters:

Column:	30,0 m	BGB-176/BGB-15 0,25/0,25df G/618		
Temperature:	220 / 117, 100 min iso 8/min 240, 3 min iso / 350			
Gas:	0,70 bar	H2		
Sample size:	0,2 µL	Split ratio:	20 : 1	

Figure S167. GC traces of enantioenriched compound **1d** obtained by cyclisation of racemic mixture of **4a** and **6a** catalyzed by **IDPi-5c**.

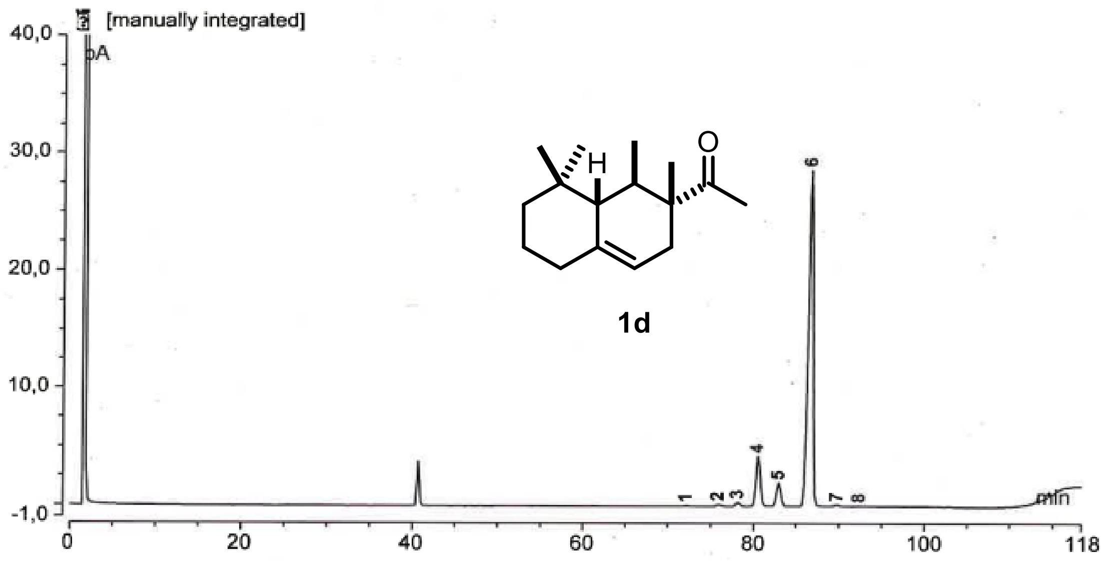

No.	Ret.Time min	Rel.Area %	Peak Name
1	72,17	0,15	***
2	75,92	0,52	isoESuper + isoalpha
3	78,18	0,87	isoESuper + isoalpha + *?
4	80,49	11,18	*
5	82,96	5,41	isoESuper plus
6	86,63	81,22	isoESuper plus
7	89,77	0,54	isogamma
8	92,43	0,10	isogamma

Instrument parameters:

Column:	30,0 m	BGB-176/BGB-15 0,25/0,25df G/618
Temperature:	220 / 117, 100 min iso 8/min 240, 3 min iso / 350	
Gas:	0,70 bar	H2
Sample size:	0,2 µL	Split ratio: 10 : 1

Figure S168. GC traces of enantioenriched compound **1d**.

20. Data for structural analyses of the single crystals of osmate esters

20.1. X-ray Crystal Structure Analysis of 4qa:

C_{17} H_{34} N_2 O_5 Os, M_r = 536.66 g mol^{-1}, yellow plate, crystal size 0.112 x 0.061 x 0.031 mm^3, monoclinic, space group $P2_1$ [4], a = 7.6404(2) Å, b = 11.0961(3) Å, c = 12.0511(3) Å, β= 97.6687(15)°, V = 1012.54(5) Å^3, T = 100(2) K, Z = 2, D_{calc} = 1.760 g·cm^3, λ = 0.71073 Å, $\mu(Mo\text{-}K_\alpha)$ = 6.324 mm^{-1}, Numerical correction ($T_{\min}$ = 0.64829, $T_{\max}$ = 0.86543), Bruker-AXS D8 Venture with Photon III detector and IμS Diamond microfocus Mo-anode X-ray source, 2.505 < θ < 32.630°, 155580 measured reflections, 7427 independent reflections, 7186 reflections with $I > 2\sigma(I)$, R_{int} = 0.0593. The structure was solved by *SHELXT* and refined by full-matrix least-squares (*SHELXL*) against F^2 to R_1 = 0.0154 [$I > 2\sigma(I)$], wR_2 = 0.0362 [all data], 234 parameters, 1 restraints and absolute structure parameter x = -0.013(3).

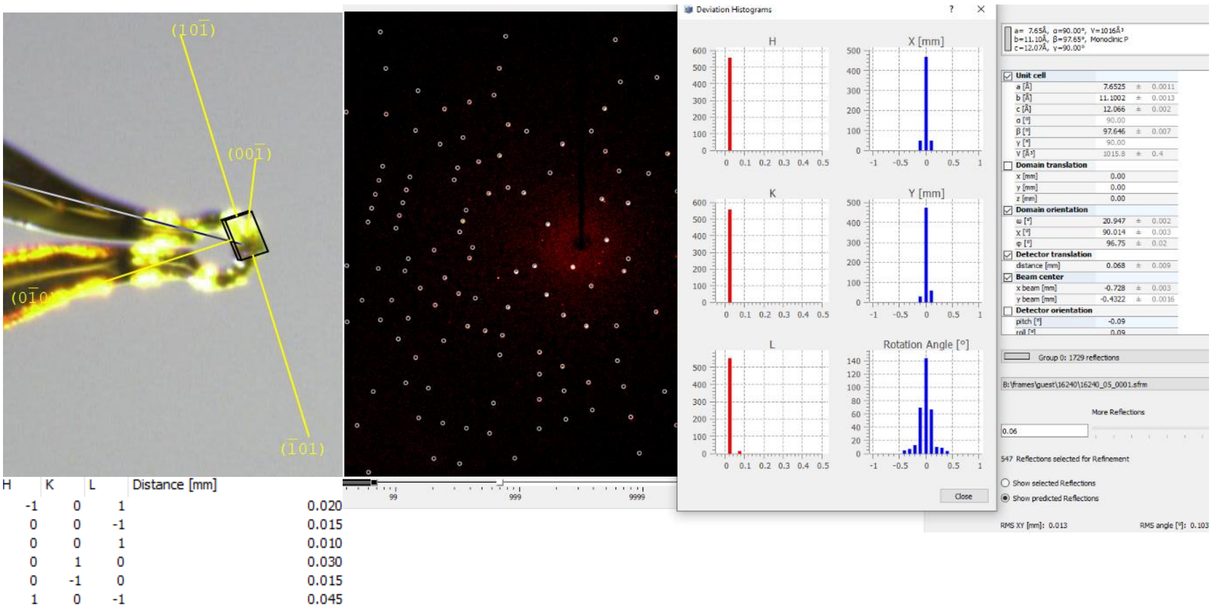

Figure S169. Crystal faces and unit cell determination/refinement of 4qa.

INTENSITY STATISTICS FOR DATASET

Resolution	#Data	#Theory	%Complete	Redundancy	Mean I	Mean I/s	Rmerge	Rsigma
Inf - 2.62	114	119	95.8	25.73	204.07	120.30	0.0278	0.0121
2.62 - 1.78	267	267	100.0	31.69	145.94	114.26	0.0290	0.0072
1.78 - 1.42	369	369	100.0	29.66	117.16	102.90	0.0345	0.0082
1.42 - 1.23	385	385	100.0	25.47	93.38	85.70	0.0394	0.0097
1.23 - 1.12	366	366	100.0	24.21	73.03	73.20	0.0467	0.0113
1.12 - 1.04	393	393	100.0	23.42	61.40	65.76	0.0527	0.0127
1.04 - 0.98	367	367	100.0	22.95	55.77	60.93	0.0583	0.0138
0.98 - 0.93	392	392	100.0	21.94	50.13	54.22	0.0640	0.0151
0.93 - 0.89	363	363	100.0	21.80	45.62	51.23	0.0692	0.0164
0.89 - 0.85	437	437	100.0	20.78	39.65	44.85	0.0782	0.0185
0.85 - 0.83	271	271	100.0	19.89	36.98	41.74	0.0847	0.0202
0.83 - 0.80	436	436	100.0	19.80	36.11	39.85	0.0859	0.0212
0.80 - 0.78	317	317	100.0	19.29	30.68	34.64	0.0962	0.0240
0.78 - 0.75	544	544	100.0	18.89	28.40	31.75	0.1052	0.0264
0.75 - 0.74	220	220	100.0	18.57	26.48	29.30	0.1110	0.0283
0.74 - 0.72	452	452	100.0	18.21	28.17	29.87	0.1136	0.0287

0.72 - 0.70	492	492	100.0	17.73	22.59	24.58	0.1314	0.0344
0.70 - 0.69	263	263	100.0	16.00	19.49	20.58	0.1468	0.0407
0.69 - 0.68	324	324	100.0	16.39	22.30	22.65	0.1422	0.0385
0.68 - 0.66	669	672	99.6	15.47	18.92	18.72	0.1580	0.0476
0.76 - 0.66	2613	2616	99.9	17.07	22.78	24.13	0.1312	0.0360
Inf - 0.66	7441	7449	99.9	20.91	49.98	48.90	0.0590	0.0167

Complete .cif-data are available under the CCDC number CCDC-**2472884**.

20.2. X-ray Crystal Structure Analysis of 4ra:

$C_{18}H_{36}N_2O_5Os$, M_r = 550.69 g mol^{-1}, yellow prism, crystal size 0.151 x 0.142 x 0.02 mm^3, monoclinic, space group $P2_1$ [4], a = 8.5320(7) Å, b = 12.0721(10) Å, c = 10.6708(9) Å, β= 103.863(3)°, V = 1067.07(15) Å^3, T = 100(2) K, Z = 2, D_{calc} = 1.714 g·cm^3, λ = 0.71073 Å, $\mu(Mo\text{-}K_\alpha)$ = 6.004 mm^{-1}, Numerical correction (T_{min} = 0.57702, T_{max} = 0.89087), Bruker-AXS Mach3 Goniometer with APEXII detector and IμS microfocus Mo-anode X-ray source, 1.966 < θ < 31.943°, 36553 measured reflections, 7319 independent reflections, 6862 reflections with $I > 2\sigma(I)$, R_{int} = 0.0354. The structure was solved by *SHELXT* and refined by full-matrix least-squares (*SHELXL*) against F^2 to R_1 = 0.0183 [$I > 2\sigma(I)$], wR_2 = 0.0337 [all data], 303 parameters, 73 restraints and absolute structure parameter x = 0.011(4).

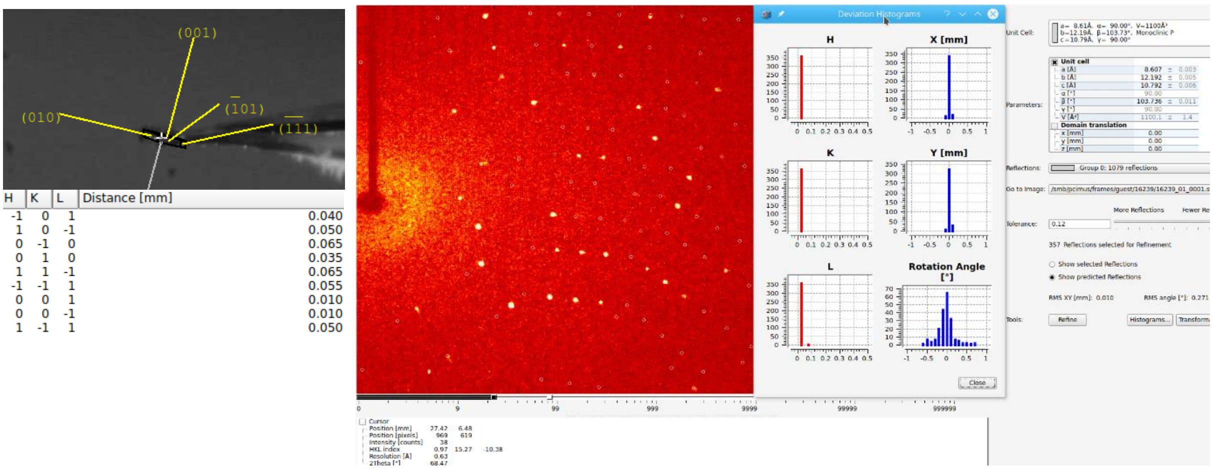

Figure S170. Crystal faces and unit cell determination/refinement of **4ra**.

INTENSITY STATISTICS FOR DATASET

Resolution	#Data	#Theory	%Complete	Redundancy	Mean I	Mean I/s	Rmerge	Rsigma
Inf - 2.71	111	115	96.5	8.82	186.90	55.42	0.0284	0.0169
2.71 - 1.83	259	260	99.6	9.28	137.56	53.14	0.0259	0.0170
1.83 - 1.45	368	368	100.0	9.36	105.75	50.36	0.0286	0.0179
1.45 - 1.27	364	364	100.0	9.28	79.11	45.99	0.0316	0.0191
1.27 - 1.15	371	371	100.0	8.83	67.26	41.65	0.0325	0.0209
1.15 - 1.06	395	396	99.7	7.33	54.77	35.16	0.0370	0.0250
1.06 - 1.00	361	361	100.0	5.63	47.31	29.34	0.0373	0.0306
1.00 - 0.95	374	374	100.0	4.99	42.53	26.22	0.0412	0.0349
0.95 - 0.91	368	369	99.7	4.30	41.76	24.52	0.0435	0.0379
0.91 - 0.87	428	428	100.0	4.07	32.78	20.43	0.0455	0.0434
0.87 - 0.84	368	368	100.0	3.76	30.09	19.08	0.0485	0.0487
0.84 - 0.82	300	300	100.0	3.74	29.70	18.59	0.0493	0.0504
0.82 - 0.79	484	485	99.8	3.64	25.92	16.84	0.0503	0.0551
0.79 - 0.77	328	328	100.0	3.41	24.10	15.54	0.0563	0.0624
0.77 - 0.75	429	429	100.0	3.32	21.74	14.43	0.0599	0.0674
0.75 - 0.74	214	214	100.0	3.36	20.38	13.42	0.0633	0.0700
0.74 - 0.72	477	477	100.0	3.23	18.74	12.60	0.0653	0.0774
0.72 - 0.71	231	232	99.6	3.00	19.33	12.02	0.0615	0.0787
0.71 - 0.69	589	589	100.0	2.96	16.42	10.71	0.0737	0.0916
0.69 - 0.68	315	315	100.0	2.95	14.49	10.02	0.0801	0.1002
0.68 - 0.67	203	227	89.4	2.30	14.24	8.93	0.0777	0.1122
0.77 - 0.67	2458	2483	99.0	3.05	17.99	11.85	0.0673	0.0824

```
Inf - 0.67    7337   7370     99.6     4.97     43.30   24.21  0.0353  0.0357
--------------------------------------------------------------------------------
```

Complete .cif-data are available under the CCDC number CCDC-**2472883**.

The carbon atoms of the TMEDA unit are disordered over two positions. The occupancy of each part was freely refined with an FVAR and found to be 66:34%. The atomic displacement parameters of the disordered atoms were treated using the ISOR instruction.

21. Appendix 1: Screening Table

Table S34. Screening of the catalysts and reaction conditions with **4a** synthesis as a model.

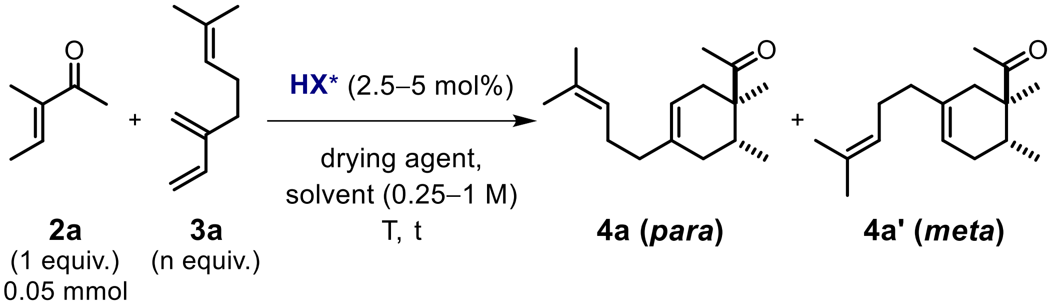

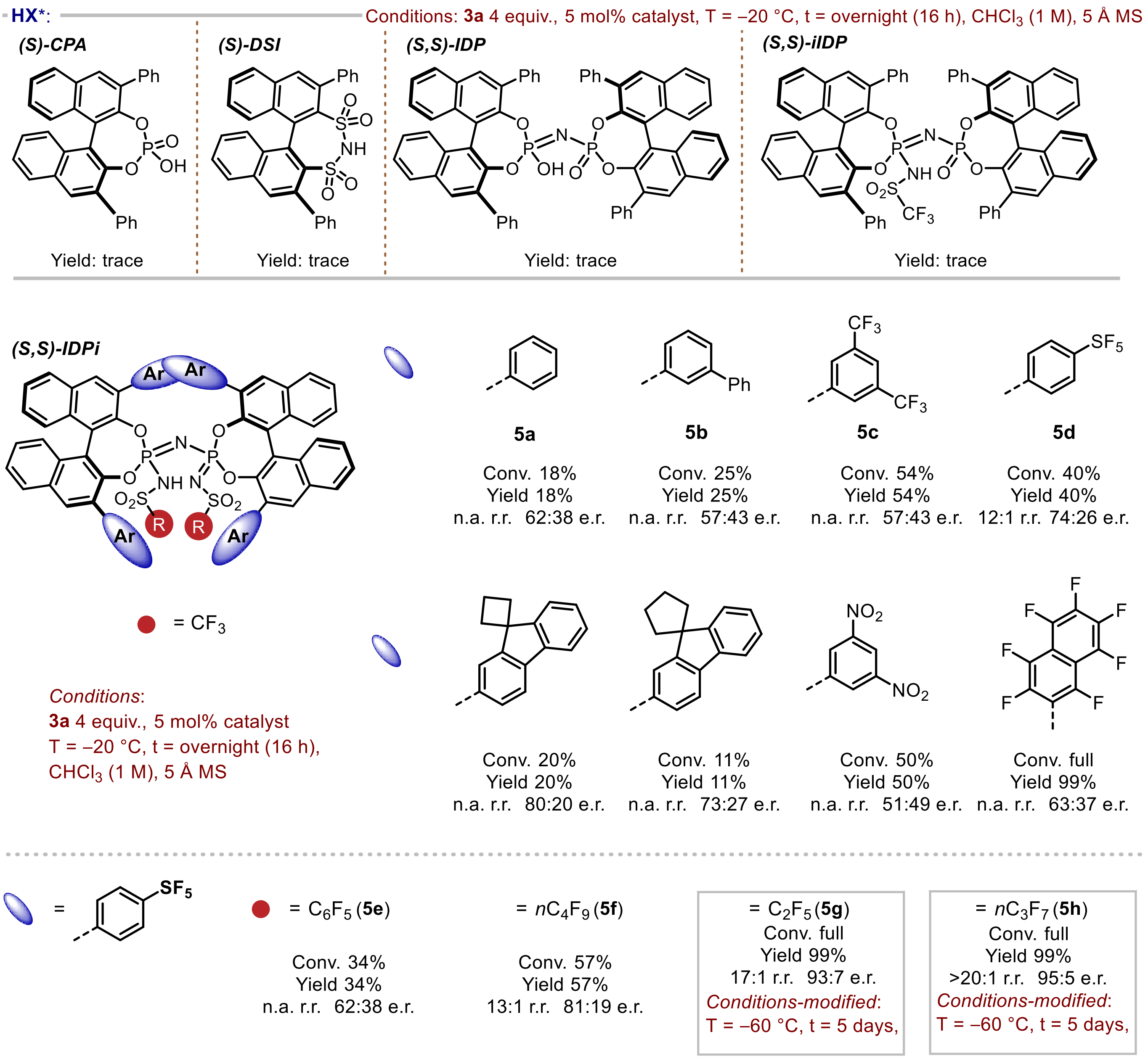

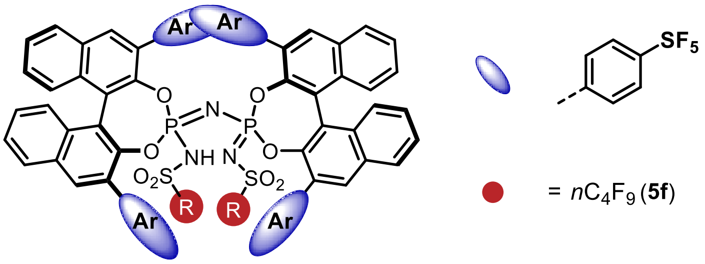

Conditions:
3a 4 equiv., 5 mol% catalyst
T = –20 °C, t = overnight (16 h),
$CHCl_3$ (1 M), 5 Å MS

Solvent Screening

**Solvent**	**CH_2Cl_2**	**$CHCl_3$**	**Et_2O**	**neat (3a)**	**MeCy**	**nC_6H_{14}**	**EtOAc**	**PhMe**
**Result**	Conv. 47%<br>Yield 47%<br>n.a. r.r.<br>78:22 e.r.	Conv. 64%<br>Yield 64%<br>n.a. r.r.<br>81:19 e.r.	Conv. 70%<br>Yield 70%<br>n.a. r.r.<br>60:40 e.r.	Conv. full<br>Yield 99%<br>n.a. r.r.<br>75:25 e.r.	Conv. full<br>Yield 99%<br>n.a. r.r.<br>78:22 e.r.	Conv. 89%<br>Yield 89%<br>n.a. r.r.<br>78:22 e.r.	Conv. 66%<br>Yield 66%<br>n.a. r.r.<br>61:39 e.r.	Conv. 90%<br>Yield 90%<br>n.a. r.r.<br>73:27 e.r.
				T = –20 °C				

Temperature Screening

**T**	**–10**	**–65**	**–80**
**Result**	Conv. 64%<br>Yield 64%<br>n.a. r.r.<br>81:19 e.r.	Conv. full<br>Yield 99%<br>17:1 r.r.<br>93:7 e.r.	Conv. 59%<br>Yield 59%<br>18:1 r.r.<br>95:5 e.r.
	t = 24 h	***t = 5 days***	***t = 6 days***

Diene Equivalent Screening

3a equiv.	**2**	**3**	**4**
**Result**	Conv. 75%<br>Yield 75%<br>17:1 r.r.<br>92:8 e.r.	Conv. 94%<br>Yield 94%<br>17:1 r.r.<br>92:8 e.r.	Conv. full<br>Yield 99%<br>17:1 r.r.<br>92:8 e.r.

Additive Screening

**Solvent**	**3 Å M.S.**	**4 Å M.S.**	**5 Å M.S.**	***$MgSO_4$***	***Na_2SO_4***	***no additive***
**Result**	trace	Conv. 27%<br>Yield 27%<br>n.a. r.r.<br>92:8 e.r.	Conv. full<br>Yield 99%<br>17:1 r.r.<br>92:8 e.r.	trace	trace	trace

Conversion (Conv.) and regioisomeric ratio (r.r.) were determined by ^1H NMR, e.r. (enantiomeric ratio) was determined by chiral HPLC. Note 1.: Due to the initial problems with **7e** synthesis, the optimizations were performed using catalysts: **5d,g** and **h**.

22. Appendix 2: Space-filling model of the IDPi-5h

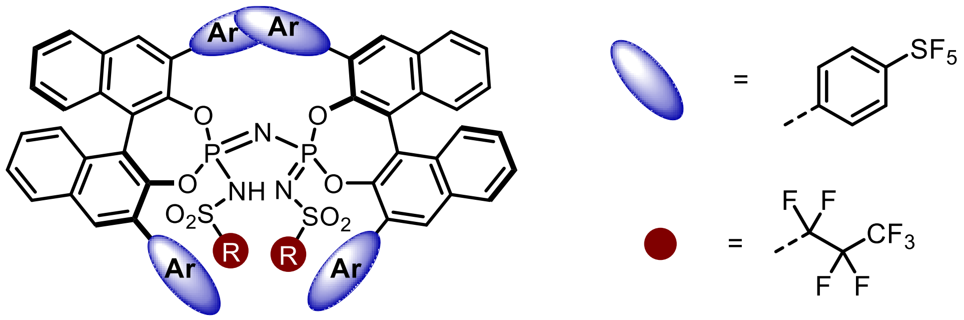

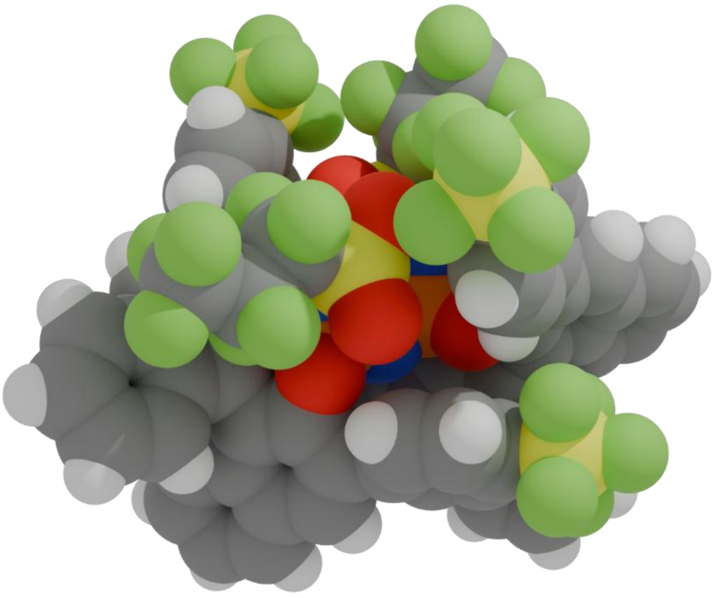

23. Appendix 3: Setup of the Preparative HPLC

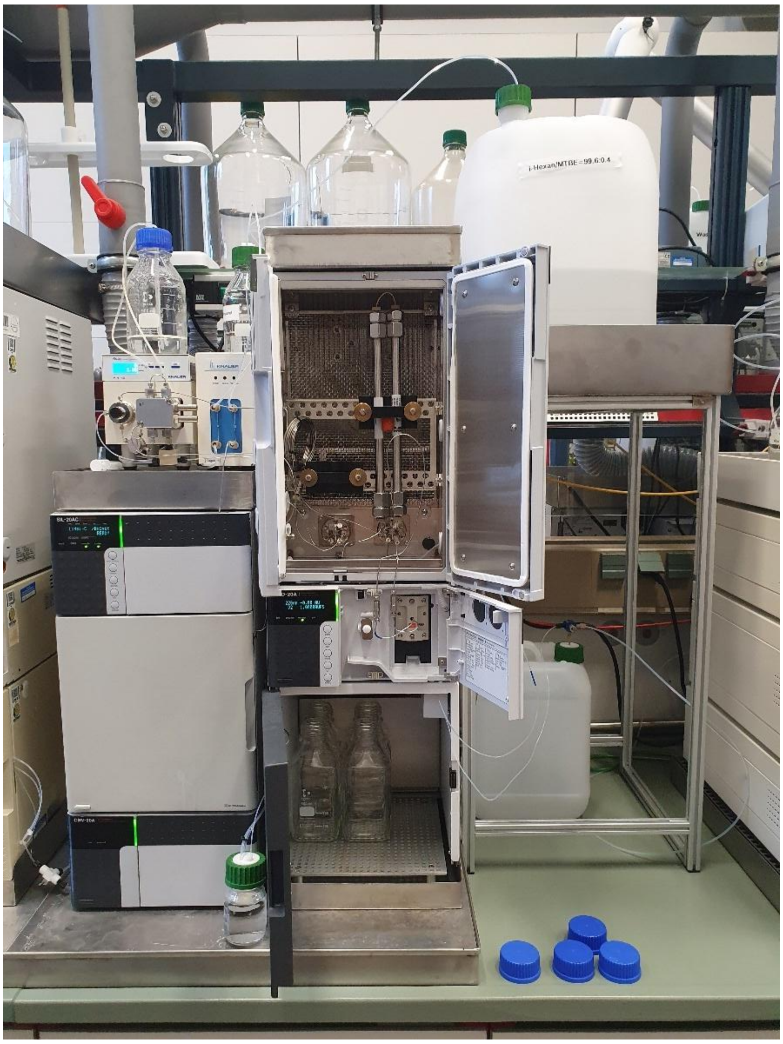

Figure S171. The setup of the preparative HPLC with the recycling system.

24. Appendix 4: Olfactory analysis

Procedure for the olfactory evaluation of the **4q**, *ent*-**4q**, **4r**, *ent*-**4r**:

10% solutions (*m*/*m*) in dipropylene glycol of given compounds were analyzed on paper blotters over a period of 24 h, with smelling fresh and at 4 h, 8 h, 24 h. The solutions were prepared from pure compounds (> 99% GC purity) distilled prior to use.
